# Single-Cell RNA Sequencing of Uterine Tissue from a 70-Day-Old Female Rabbit: Cellular Composition and Fibroblast-Associated Networks

**DOI:** 10.3390/genes17091131

**Published:** 2026-09-16

**Authors:** Tianyou Du, Kerui Xie, Wenli Zhang, Xun Xie, Xinzhong Fan, Dan Wang, Qin Zhang

**Affiliations:** Shandong Provincial Key Laboratory of Animal Biotechnology and Disease Control and Prevention, College of Animal Science and Veterinary Medicine, Shandong Agricultural University, Tai’an 271018, China; dutianyou0728@163.com (T.D.); xiekerui666@163.com (K.X.); 13210621045@163.com (W.Z.); xiexun0612@163.com (X.X.); xzfan@sdau.edu.cn (X.F.)

**Keywords:** rabbit, uterus, single-cell RNA sequencing, single-cell transcriptomic atlas, fibroblast

## Abstract

**Background/Objectives:** The cellular composition and molecular features of the rabbit uterus remain incompletely understood. This study aimed to provide an initial single-cell characterization of the cell populations, transcriptional features, and predicted communication networks identified in one rabbit uterine-body specimen. **Methods:** Single-cell RNA sequencing was performed on uterine-body tissue from one 70-day-old female Kangda V-line rabbit. Following quality control, cells were clustered and annotated using canonical markers. Gene Ontology and Kyoto Encyclopedia of Genes and Genomes enrichment analyses, descriptive functional module scoring, CellChat, and high-dimensional weighted gene co-expression network analysis were used to characterize cell-associated expression patterns, predicted intercellular communication, and fibroblast co-expression modules. **Results:** A total of 9251 cells from this single specimen were retained and resolved into 16 preliminary clusters; 15 clusters were assigned to nine major cell types, whereas one small cluster remained unclassified. Fibroblasts were the predominant cell population among the analyzed cells (76.6%), followed by smooth muscle cells (10.9%). Functional analyses highlighted expression programs related to extracellular matrix organization, contractility, vascular function, epithelial barrier maintenance, and immune responses. CellChat analysis using an unadjusted empirical permutation *p* value < 0.05 predicted prominent COLLAGEN, LAMININ, CD99, MIF, and PTN signaling. Integration with fibroblast co-expression modules identified 21 candidate genes, including *COL1A1*, *COL1A2*, *COL6A2*, *LAMC1*, *PTN*, *BMP2*, and *PDGFRA*, linking fibroblast-associated expression programs with predicted extracellular-matrix, adhesion, vascular, and developmental relationships. **Conclusions:** This single-specimen study provides a preliminary cell-level reference for rabbit uterine tissue. The relatively high proportion of fibroblasts and the predicted communication patterns generate testable hypotheses for future studies using independent animals and functional validation.

## 1. Introduction

The domestic rabbit (*Oryctolagus cuniculus*) is an important meat-producing animal and is also widely used as a classical model organism for studies of mammalian embryo implantation, reproductive development, and endometrial repair [1,2]. Reproductive performance in female rabbits directly affects the economic efficiency of rabbit production. As the central organ involved in embryo recognition, implantation, and early development, the uterus plays a critical role in determining female reproductive performance. Therefore, analysis of gene expression in uterine tissue is essential for understanding the genetic basis of female reproductive performance in rabbits. However, the uterus comprises diverse epithelial, stromal, smooth muscle, endothelial, and immune cell populations that exhibit substantial cellular and transcriptional heterogeneity across different reproductive states [3,4]. Conventional bulk RNA-seq only reflects average gene expression across mixed cell populations and cannot resolve cell-type-specific expression patterns and intercellular interactions [5]. Characterizing the cellular composition, molecular expression features, and cell–cell communication patterns of the uterus is therefore fundamental for understanding reproductive function in female rabbits [6].

Single-cell RNA sequencing (scRNA-seq) enables transcriptomic profiling at single-cell resolution and can be used to identify cell populations, detect cluster-enriched marker genes, characterize transcriptional states, and infer potential intercellular communication networks [7,8,9]. Rabbit single-cell studies have begun to characterize embryonic differentiation and tissue-specific cellular heterogeneity, including developmental comparisons with mice and the developing rabbit sclera [10,11]. Single-cell transcriptomic studies of the uterus and other reproductive tissues have been reported in cattle [12], pigs [13], humans [14], and mice [15], revealing the important roles of epithelial, stromal, immune, and vascular-associated populations in endometrial remodeling and reproductive function. However, no comparable single-cell atlas of the rabbit uterus has yet been reported.

Rabbits are important livestock and experimental animals, but single-cell transcriptomic information on the rabbit uterus remains limited. Characterizing uterine cellular composition and gene-expression patterns at single-cell resolution can provide a foundation for investigating uterine development and reproductive biology in this species. In the present study, uterine tissue from one healthy 70-day-old female Kangda V-line rabbit was analyzed using high-throughput scRNA-seq. We generated an initial cell-type-resolved transcriptomic reference for this uterine-body specimen and integrated functional enrichment, CellChat, hdWGCNA, and functional module scoring to characterize the recovered cell populations and fibroblast-associated expression networks.

## 2. Materials and Methods

### 2.1. Experimental Animal and Tissue Collection

One healthy 70-day-old female Kangda V-line rabbit weighing 2.5 kg was used in this study. The rabbit was weaned at 35 days of age and maintained at an ambient temperature of 18–24 °C, a relative humidity of 55–65%, and a daily photoperiod of 8–10 h. The animal was fed a complete pelleted diet formulated for growing meat rabbits and had ad libitum access to feed and water. At 70 days of age, the animal was considered to be in a juvenile developmental period preceding regular reproductive activity. The rabbit was euthanized by intravenous administration of an overdose of pentobarbital sodium (100 mg/kg body weight) via the marginal ear vein. Immediately after euthanasia, the uterus was aseptically excised, and the surrounding adipose and connective tissues were removed. All tissue used for single-cell RNA sequencing (scRNA-seq) was collected exclusively from the uterine body. The endometrium and myometrium were processed together, and tissues from the uterine horns, cervix, or other anatomical regions were not pooled before dissociation. The specimen was immediately placed in serum-free Minimum Essential Medium pre-cooled to 4 °C, transported to the laboratory on ice, and processed within 4 h of collection.

### 2.2. Preparation of Single-Cell Suspensions

Fresh uterine tissue was washed with pre-cooled phosphate-buffered saline (PBS), cut into approximately 2-mm pieces, and incubated in a dissociation solution containing collagenase type II (Sigma-Aldrich, St. Louis, MO, USA), DNase I (Sigma-Aldrich, St. Louis, MO, USA), and 2% fetal bovine serum (FBS; Gibco, Waltham, MA, USA) at 37 °C for 20–45 min with gentle rotation. After dissociation, the cell suspension was filtered through a 70-μm SmartStrainer (Miltenyi Biotec, Bergisch Gladbach, Germany), centrifuged at 300× *g* for 10 min at 4 °C, and resuspended in 1 mL of PBS. The cell suspension was mixed with Red Cell Lysis Solution (Solarbio, Beijing, China) at a ratio of 1:3 and incubated for 3–5 min at room temperature. The suspension was then centrifuged at 300× *g* for 5 min at 4 °C, and the resulting cell pellet was resuspended in PBS containing 0.04% bovine serum albumin (BSA). Cell concentration and viability were evaluated using an automated cell counter, while cell morphology, aggregation, and debris levels were examined microscopically. Only single-cell suspensions with a viability of ≥85% were retained, and the concentration of viable cells was adjusted to 300–600 cells/μL for subsequent library construction [12].

### 2.3. Library Construction and Single-Cell RNA Sequencing

The uterine single-cell suspension was loaded onto a Chromium Single Cell Controller (10x Genomics, Pleasanton, CA, USA). Single-cell 3′ gene expression libraries were generated using the Chromium Single Cell 3′ Library and Gel Bead Kit v3.1 and the Chromium Single Cell G Chip Kit (10x Genomics) [16]. Individual cells were partitioned into gel bead-in-emulsions (GEMs), in which the cells were lysed and the released mRNA molecules were captured and barcoded during reverse transcription. Reverse transcription was performed using an S1000 Touch Thermal Cycler (Bio-Rad, Hercules, CA, USA) at 53 °C for 45 min, followed by 85 °C for 5 min, after which the reactions were maintained at 4 °C. The resulting cDNA molecules, containing cell-specific barcodes and unique molecular identifiers (UMIs), were amplified and used for library construction. The quality and fragment-size distributions of the amplified cDNA and sequencing libraries were assessed using an Agilent 4200 TapeStation system (Agilent Technologies, Santa Clara, CA, USA). Library preparation and sequencing were performed by LC-Bio Technologies (Hangzhou) Co., Ltd. (Hangzhou, China). Qualified libraries were sequenced on an Illumina NovaSeq 6000 platform (Illumina, San Diego, CA, USA) using a paired-end 150-bp sequencing strategy, with a sequencing depth of at least 100,000 reads per cell.

### 2.4. Preprocessing, Dimensionality Reduction, and Clustering of scRNA-Seq Data

Raw sequencing reads in FASTQ format were processed using Cell Ranger (version 7.0.1; 10x Genomics, Pleasanton, CA, USA), aligned to the rabbit reference genome assembly mOryCun1.1 (NCBI accession no. GCF_964237555.1), and converted into a gene-by-cell UMI count matrix. Subsequent analyses were conducted using Seurat (version 5.4.0) [17]. Initial cell-level quality-control filtering excluded genes detected in fewer than three cells, and cells were retained if they contained >500 UMIs and >250 detected genes, had a log10GenesPerUMI value > 0.8, and had a mitochondrial transcript proportion < 10%, with log10GenesPerUMI calculated as log10(nGene)/log10(nUMI). To prepare the Seurat object required for DoubletFinder, the quality-filtered expression matrix was normalized using the LogNormalize method implemented in NormalizeData with a scale factor of 10,000, and the 2000 most highly variable genes were identified using FindVariableFeatures. The data were then centered and scaled using ScaleData, followed by principal component analysis using RunPCA. A shared nearest-neighbor graph was constructed using FindNeighbors based on the first 30 principal components, and preliminary clustering was performed using FindClusters at a resolution of 0.5; these preliminary cluster assignments were used only to support doublet detection and estimation of the homotypic doublet proportion. Potential doublets were subsequently identified using DoubletFinder [18]. A parameter sweep was performed using the first 10 principal components with pN = 0.25, and the optimal pK value of 0.005 was selected according to the maximum mean–variance-normalized bimodality coefficient (BCmvn). The expected number of doublets was calculated as round (N^2^ × 8 × 10^−6^), where N represents the number of cells subjected to doublet detection, and was adjusted according to the estimated homotypic doublet proportion based on the preliminary cluster assignments. Cells classified as doublets were excluded, and the retained singlets were subsequently visualized using RunUMAP based on the first 30 principal components and used for all downstream analyses.

### 2.5. Cell-Type Annotation

Positive cluster-enriched marker candidates were identified using FindAllMarkers in Seurat (version 5.4.0) with only.pos = TRUE, min.pct = 0.10, logfc.threshold = 0.25, and test.use = “wilcox”. Thus, genes detected in at least 10% of cells in either comparison group and showing an average log2 fold change of at least 0.25 were evaluated using the Wilcoxon rank-sum test. Seurat calculated Bonferroni-adjusted *p* values across all tested features, and an adjusted *p* value < 0.05 was considered statistically significant. A hierarchical tree of cell clusters was generated using BuildClusterTree, and representative marker expression was visualized using VlnPlot. Cell types were manually annotated using rabbit gene symbols generated from alignment to the mOryCun1.1 reference genome, established canonical mammalian markers, published single-cell studies, and CZ CELLxGENE Discover (accessed on 17 July 2026). No genome-wide human-to-rabbit one-to-one ortholog conversion was performed. The complete marker output and annotated cluster assignments are provided in Table S7.

### 2.6. Cell–Cell Communication Analysis

Cell–cell communication was inferred using CellChat (version 1.6.1) [19,20,21] based on the normalized RNA expression matrix and cell-type annotations in the Seurat object. Because a rabbit-specific CellChat database was unavailable, the human ligand–receptor interaction database was used, and rabbit genes matching entries in the human database were retained. Overexpressed signaling genes and ligand–receptor pairs were identified using identifyOverExpressedGenes and identifyOverExpressedInteractions, respectively. Communication probabilities and empirical *p* values were calculated using the default trimean method and one-sided permutation test implemented in computeCommunProb. Interactions involving cell populations containing fewer than 10 cells were excluded. Predicted ligand–receptor interactions with an unadjusted permutation *p* value < 0.05 were retained and subsequently aggregated to construct pathway-level communication networks. The numbers and strengths of predicted interactions, pathway-level communication networks, and outgoing and incoming signaling patterns were then evaluated.

### 2.7. High-Dimensional Weighted Gene Co-Expression Network Analysis

High-dimensional weighted gene co-expression network analysis was performed using hdWGCNA (version 0.4.6) [22] within the fibroblast subset containing 7088 cells, and all network-construction steps used the fibroblast-specific expression matrix. Genes expressed in at least 5% of fibroblasts were retained, and transcriptionally similar fibroblasts were aggregated into metacells using 25 nearest neighbors, with a maximum overlap of 10 cells between metacells. A signed fibroblast-specific co-expression network was constructed using a soft-thresholding power of 3 selected from scale-free topology diagnostics. Module detection, module eigengenes, and eigengene-based connectivity (kME) were calculated from the fibroblast-specific expression matrix. The grey module was excluded, the 10 genes with the highest kME values in each module were designated candidate hub genes, and UCell scores based on the top 25 hub genes were used to visualize the distribution of module signatures across the major cell types.

### 2.8. Functional Enrichment Analysis

Functional annotation was performed using the DAVID Bioinformatics Resources platform for marker genes identified by FindAllMarkers and module genes identified by hdWGCNA [23]. Official rabbit gene symbols were used, with Oryctolagus cuniculus selected as the background species. GO [24] and KEGG [25] enrichment analyses were performed. For GO analysis, term size was defined as the number of genes assigned to a term in the annotated background. Terms were retained only when they contained 10–500 background genes, included at least three input genes, and had a Benjamini-adjusted *p* value < 0.05. Redundancy was reduced within each ontology using the Jaccard similarity of input-gene sets: when similarity was at least 0.70, the term with the smaller background gene set was retained, with the lower adjusted *p* value used to break ties. The 10 most significant nonredundant terms per ontology were displayed. KEGG pathways with a Benjamini-adjusted *p* value < 0.05 were considered significant. Bubble plots show gene ratio, input-gene count, and −log10(Benjamini-adjusted *p*).

### 2.9. Integrated CellChat and hdWGCNA Analysis

Ligand–receptor-related genes retained by CellChat at an unadjusted empirical permutation *p* value < 0.05 were intersected with genes assigned to each of the five fibroblast hdWGCNA modules. Overlapping genes were summarized by module membership, signaling pathway, interaction number, communication probability, and predicted sender-receiver role.

### 2.10. Functional State Scoring of Major Cell Types

Nine predefined gene sets representing ECM remodeling, collagen synthesis, cell adhesion, angiogenesis, smooth muscle contraction, epithelial barrier function, inflammatory response, cell cycle, and hormone response were scored in individual cells using AddModuleScore in Seurat. Functional module scoring summarized the coordinated expression of these predefined transcriptional programs and evaluated their correspondence with the assigned cell-type annotations. Cell-level distributions and cell-type means were visualized using heatmaps, UMAP plots, and violin plots. Because all cells were derived from one biological specimen, the scores were summarized descriptively without inferential *p* values or multiple-comparison correction. Gene-set construction and the genes retained for scoring are documented in Table S5, and cell-type mean scores are reported in Table S6.

## 3. Results

### 3.1. Cell Quality Control

To establish a high-quality single-cell dataset for characterizing the rabbit uterine tissue, low-quality cells and predicted doublets were removed before downstream analysis. After initial quality control and removal of 682 predicted doublets, 9251 cells from the uterine-body specimen were retained for subsequent analyses. Quality-control distributions, principal component evaluation, and DoubletFinder results are provided in Figure S1.

### 3.2. Dimensionality Reduction, Clustering, and Cell-Type Identification

**Figure 1:**
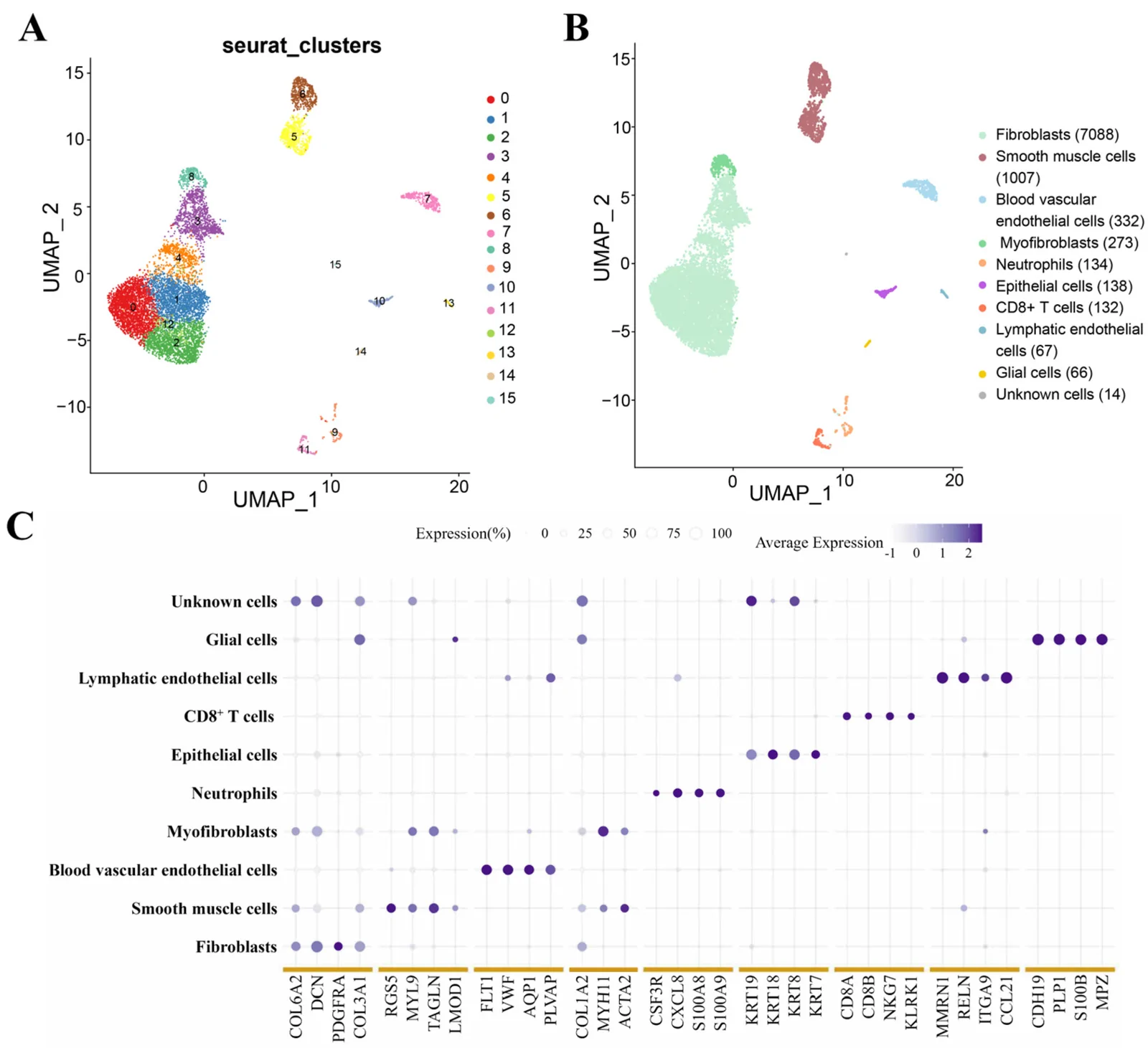
Clustering and cell-type annotation of cells recovered from the single rabbit uterine-body specimen. (**A**) UMAP of 16 preliminary Seurat clusters, with colors indicating cluster identity; (**B**) UMAP of nine annotated cell types and the 14-cell unclassified population, with colors indicating annotation and numbers in the legend indicating recovered cell counts; (**C**) dot plot of representative marker genes, in which dot size represents the percentage of cells expressing each gene and color represents average scaled expression.

Based on the Seurat dimensionality reduction results, UMAP was used to visualize single-cell clustering. A total of 16 cell clusters were identified and finally annotated into nine cell types, whereas one small cluster remained unclassified (Figure 1) according to marker genes: fibroblasts (*COL6A2*, *DCN*, *PDGFRA*, and *COL3A1*), smooth muscle cells (*RGS5*, *MYL9*, *TAGLN,* and *LMOD1*), blood vascular endothelial cells (*FLT1*, *VWF*, *AQP1*, and *PLVAP*), myofibroblasts (*COL1A2*, *MYH11*, and *ACTA2*), neutrophils (*CSF3R*, *CXCL8*, *S100A8*, and *S100A9*), epithelial cells (*KRT19*, *KRT18*, *KRT8*, and *KRT7*), CD8^+^ T cells (*CD8A*, *CD8B*, *NKG7*, and *KLRK1*), lymphatic endothelial cells (*MMRN1*, *RELN*, *ITGA9*, and *CCL21*), and glial cells (*CDH19*, *PLP1*, *S100B*, and *MPZ*). Additional annotation evidence is shown in Figure S2, and the complete positive marker results are provided in Table S7.

### 3.3. Cellular Composition of Uterine Tissue

To clarify the cellular composition of the rabbit uterine tissue, the numbers and proportions of annotated cell types were calculated (Table 1). A total of 9251 high-quality cells were obtained from the uterine sample, and clear differences in cell-type distribution were observed. Fibroblasts were the most abundant population, with 7088 cells, accounting for 76.6% of all cells, and represented the dominant cellular component in this sample. Smooth muscle cells were the second largest population, with 1007 cells (10.9%). Blood vascular endothelial cells, myofibroblasts, epithelial cells, neutrophils, and CD8^+^ T cells were less abundant, accounting for 3.6%, 3.0%, 1.5%, 1.4%, and 1.4% of all cells, respectively. Lymphatic endothelial cells and glial cells each accounted for approximately 0.7%, whereas unknown cells accounted for only 0.2%. In this specimen, mesenchyme-derived populations, particularly fibroblasts and smooth muscle cells, accounted for most of the analyzed cells. Fibroblasts represented the largest population, whereas smooth muscle cells represented the second largest population. Their transcriptional features were further examined using functional enrichment, hdWGCNA, and CellChat analyses.

### 3.4. Functional Enrichment Analysis of Cell-Type-Specific Genes

The numbers of the cell-type-specific genes are shown in Table 2; complete results are provided in Table S7.

Functional enrichment summarized coordinated transcriptional features across the annotated cell populations and provided functional context for the subsequent CellChat and hdWGCNA analyses. Using the predefined significance and term-filtering criteria, enrichment results were identified for fibroblasts, smooth muscle cells, blood vascular endothelial cells, neutrophils, and epithelial cells, although no KEGG pathway remained significant for epithelial cells. Myofibroblasts and glial cells yielded no significant terms, whereas lymphatic endothelial cells and CD8^+^ T cells produced limited MF or KEGG results. The main text therefore focuses on fibroblast-associated results related to the major compositional feature of this uterine specimen, while the supporting enrichment results for the other cell populations are provided in Figures S3–S6.

#### 3.4.1. Functional Enrichment Analysis of the Fibroblast-Specific Genes

**Figure 2:**
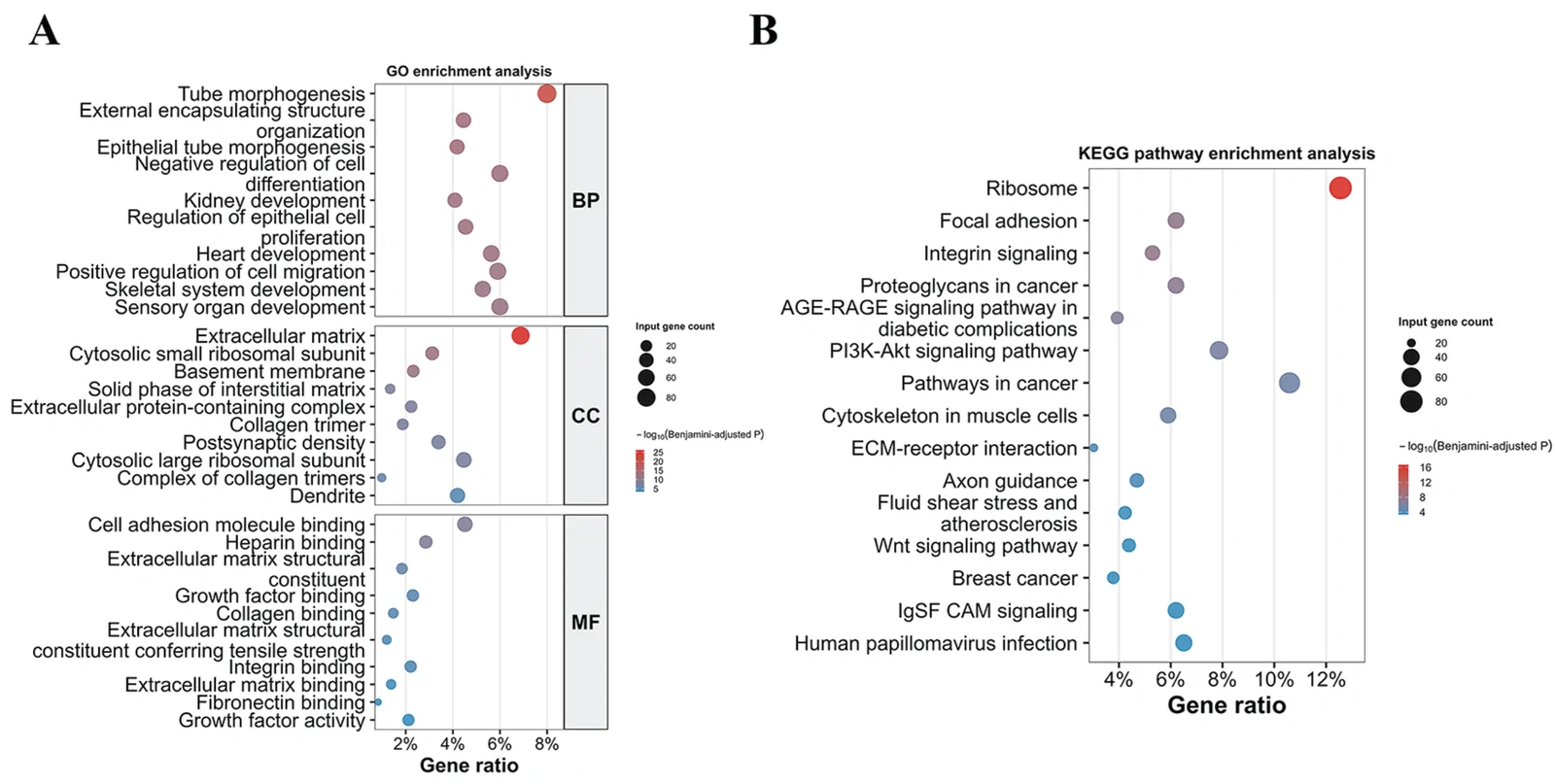
GO and KEGG enrichment analyses of fibroblast-specific genes. (**A**) GO terms separated into Biological Process (BP), Cellular Component (CC), and Molecular Function (MF); (**B**) KEGG pathways. In both panels, the *x*-axis shows the gene ratio and the *y*-axis lists enriched terms or pathways. Bubble size represents the number of input genes assigned to a term, and color represents −log10(Benjamini-adjusted *p*), with warmer colors indicating stronger statistical enrichment.

To characterize the transcriptional programs associated with the high proportion of fibroblasts in this uterine specimen, GO and KEGG enrichment analyses were performed using fibroblast-enriched marker genes. After term-size and redundancy filtering, representative BP terms included tube morphogenesis, external encapsulating structure organization, epithelial tube morphogenesis, regulation of epithelial cell proliferation, and positive regulation of cell migration (Figure 2A). Representative CC terms included extracellular matrix, basement membrane, collagen trimer, complex of collagen trimers, and extracellular protein-containing complex. Enriched MF terms included cell adhesion molecule binding, extracellular matrix structural constituent, collagen binding, integrin binding, extracellular matrix binding, and growth factor binding. Collectively, these terms identified fibroblast-associated transcriptional features related to extracellular matrix organization, cell–matrix adhesion, and tissue morphogenesis.

KEGG enrichment identified focal adhesion, integrin signaling, ECM–receptor interaction, PI3K–Akt signaling, Wnt signaling, and cytoskeleton in muscle cells among the representative pathways (Figure 2B). These enrichment patterns further connected the fibroblast population with matrix–cell interactions, cellular adhesion, cytoskeletal organization, and developmental signaling in the uterine tissue.

#### 3.4.2. Functional Features of the Other Uterine Cell Populations

The enrichment results for the other cell populations provided complementary information on uterine tissue organization (Figures S3–S6). Smooth muscle cell markers were associated with cytoskeletal organization, actin binding, focal adhesion, vascular smooth muscle contraction, cGMP–PKG signaling, oxytocin signaling, and regulation of the actin cytoskeleton. Blood vascular endothelial cell markers were associated with cell junctions, vesicle transport, Ras, PI3K–Akt, MAPK, Rap1, adherens-junction, and relaxin signaling. Neutrophil markers highlighted immune and defense responses, chemokine signaling, phagosomal and lysosomal processes, and endocytosis. Epithelial cell markers were enriched for cell-junction organization, intracellular organelles, molecular-adaptor activity, and transcriptional coregulation. Together, these results summarized contractile, vascular, immune, and epithelial transcriptional features in the uterine tissue.

### 3.5. Analysis of Cell–Cell Communication Among Different Cell Types

To characterize the predicted signaling relationships among uterine cell types and determine how fibroblasts were connected with the other populations, CellChat was used to infer directional ligand–receptor interactions and pathway-level communication patterns. Using the standard CellChat empirical permutation threshold, 1847 of the 2067 tested source–target–ligand–receptor combinations were retained at an unadjusted permutation *p* value < 0.05.

**Figure 3:**
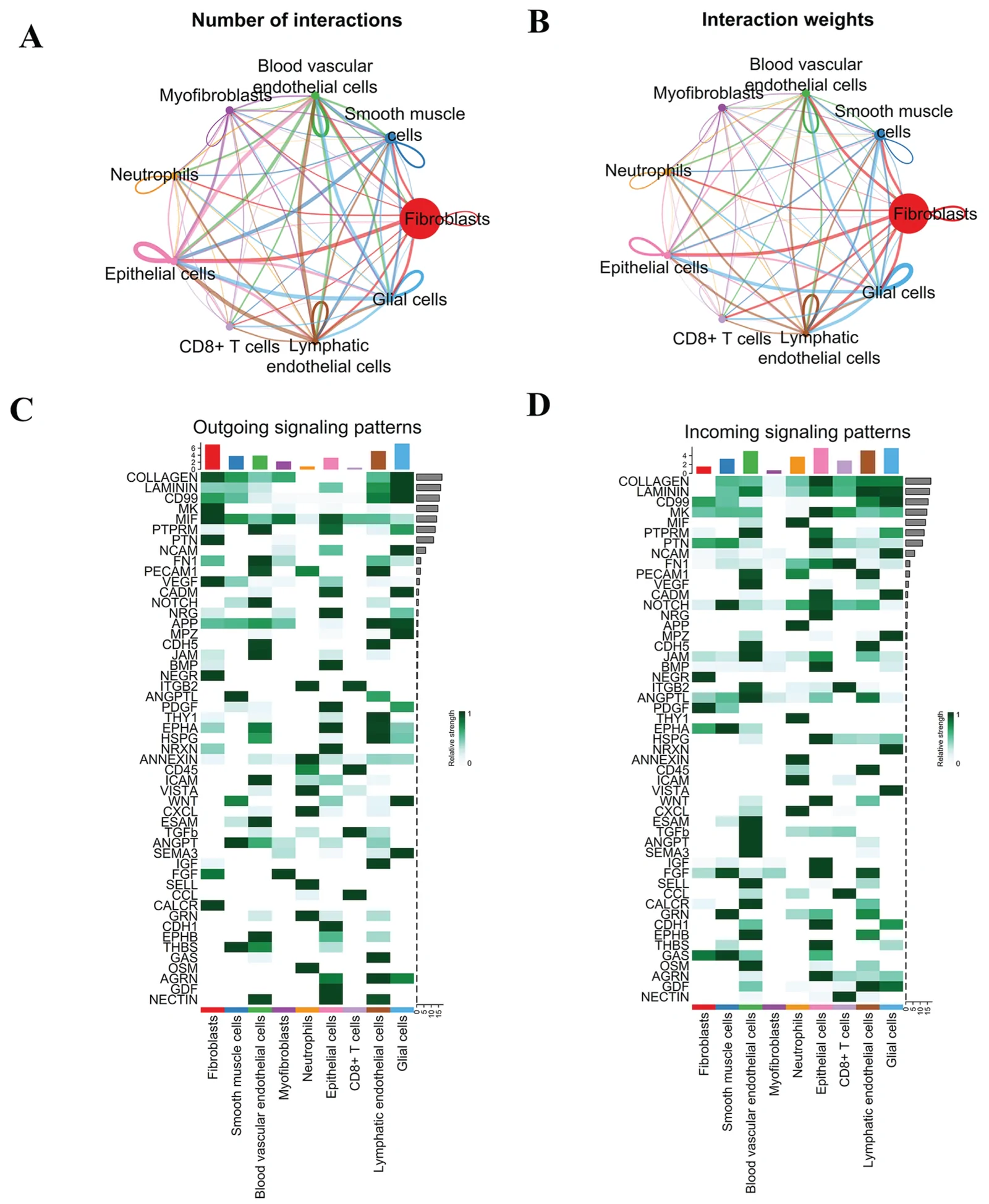
Predicted communication networks and signaling roles among the nine annotated cell types. (**A**) Directed network showing the number of predicted ligand–receptor interactions; (**B**) directed network showing the summed model-derived interaction strength. In panels (**A**,**B**), node color indicates cell type, node size represents the number of analyzed cells, edge direction indicates the predicted sender-to-receiver relationship, edge width represents the number or strength of interactions, and self-loops indicate predicted autocrine relationships. (**C**,**D**) Heatmaps showing relative outgoing and incoming signaling strength, respectively. Rows represent signaling pathways, columns represent cell types, green intensity indicates relative pathway strength, and the marginal bar plots summarize the total signaling strength.

The resulting network showed extensive predicted communication among the annotated cell populations (Figure 3A,B), with notable directional relationships involving fibroblasts, epithelial cells, and glial cells. Specifically, 59 fibroblast-to-epithelial and 9 epithelial-to-fibroblast interactions were identified, together with 62 glial-to-epithelial and 38 epithelial-to-glial interactions. These patterns highlighted predicted stromal–epithelial and glial–epithelial signaling relationships in the uterine tissue.

Outgoing and incoming signaling patterns identified COLLAGEN and LAMININ as prominent predicted pathways (Figure 3C,D; Table S1), connecting stromal cells with multiple uterine cell types through matrix- and basement-membrane-associated ligand–receptor relationships. MIF signaling linked fibroblasts with immune cell populations, indicating a predicted stromal–immune signaling component. PTN, MK, BMP, and FGF signaling represented developmental and stromal relationships potentially associated with tissue growth and maturation, whereas PDGF and VEGF signaling highlighted predicted stromal–vascular relationships. Collectively, the CellChat results identified matrix-associated, stromal–epithelial, stromal–immune, developmental, and stromal–vascular components of the predicted uterine communication network.

### 3.6. High-Dimensional Weighted Co-Expression Network Analysis of Fibroblasts

**Figure 4:**
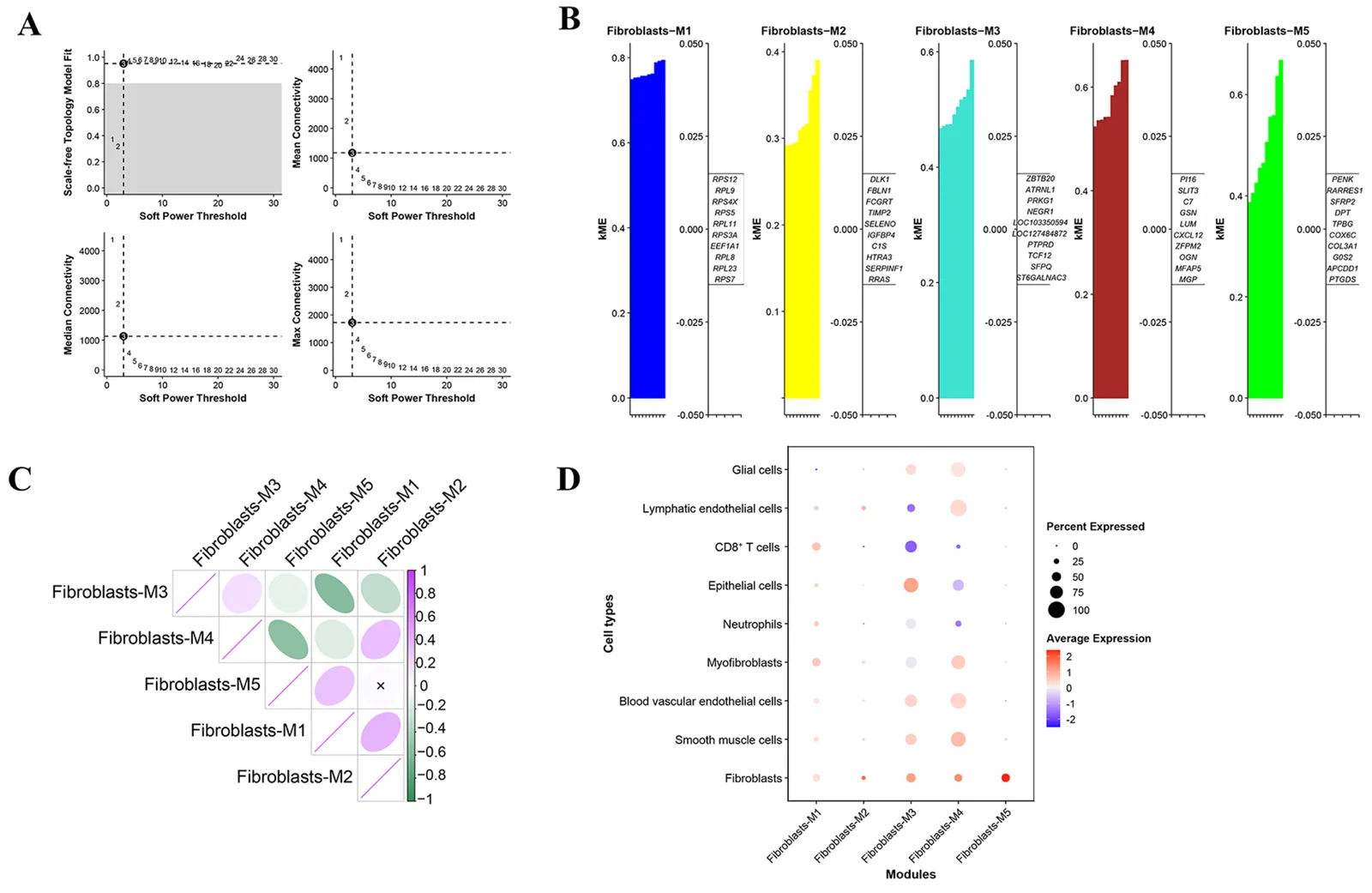
Construction and characterization of the fibroblast high-dimensional weighted gene co-expression network (hdWGCNA). (**A**) Scale-free topology model fit and mean, median, and maximum connectivity across tested soft-thresholding powers; dashed lines and labels indicate the selected power of 3. (**B**) kME values of the top hub genes in Fibroblasts-M1 to Fibroblasts-M5, with bar colors denoting module colors. (**C**) Correlations among module eigengenes, with ellipse color and orientation representing correlation direction and magnitude. (**D**) Distribution of module signatures across cell types; dot size represents the percentage of cells expressing the signature and color represents average scaled expression.

To characterize transcriptional heterogeneity within fibroblasts and identify coordinated gene-expression programs, hdWGCNA was applied to the fibroblast subset. Using a soft-thresholding power of 3 (Figure 4A), five fibroblast co-expression modules were identified and designated Fibroblasts-M1, Fibroblasts-M2, Fibroblasts-M3, Fibroblasts-M4, andFibroblasts-M5 (Figure 4B).

Hub genes were identified according to intramodular connectivity kME values. Fibroblasts-M1 was characterized primarily by ribosomal and translation-related genes, including *RPS12*, *RPL9*, *RPS4X*, *RPS5,* and *EEF1A1*. Fibroblasts-M2 contained extracellular-matrix- and growth-associated genes, including *FBLN1*, *TIMP2*, *IGFBP4*, and *SERPINF1*. Fibroblasts-M4 contained stromal and matrix-associated genes such as *LUM, OGN, MFAP5*, *CXCL12*, and *SLIT3*. Fibroblasts-M5 included *SFRP2*, *DPT*, *COL3A1*, *RARRES1*, and *APCDD1*, highlighting collagen-, matrix-remodeling-, and Wnt-associated transcriptional features. The complete module composition and the top 10 hub genes in each module are provided in Tables S2 and S3.

Module correlation analysis revealed coordinated and opposing expression patterns among the fibroblast modules (Figure 4C). Fibroblasts-M1 showed positive correlation patterns with Fibroblasts-M2 and Fibroblasts-M5, whereas Fibroblasts-M3 showed negative correlation patterns with Fibroblasts-M1 and Fibroblasts-M2. The cross-cell-type signature plot further illustrates the distribution of the five fibroblast-derived module signatures among the uterine cell types (Figure 4D). These module-level patterns provided the basis for integrating fibroblast co-expression programs with the CellChat communication network.

### 3.7. Integrated CellChat and hdWGCNA Analysis of Fibroblasts

**Figure 5:**
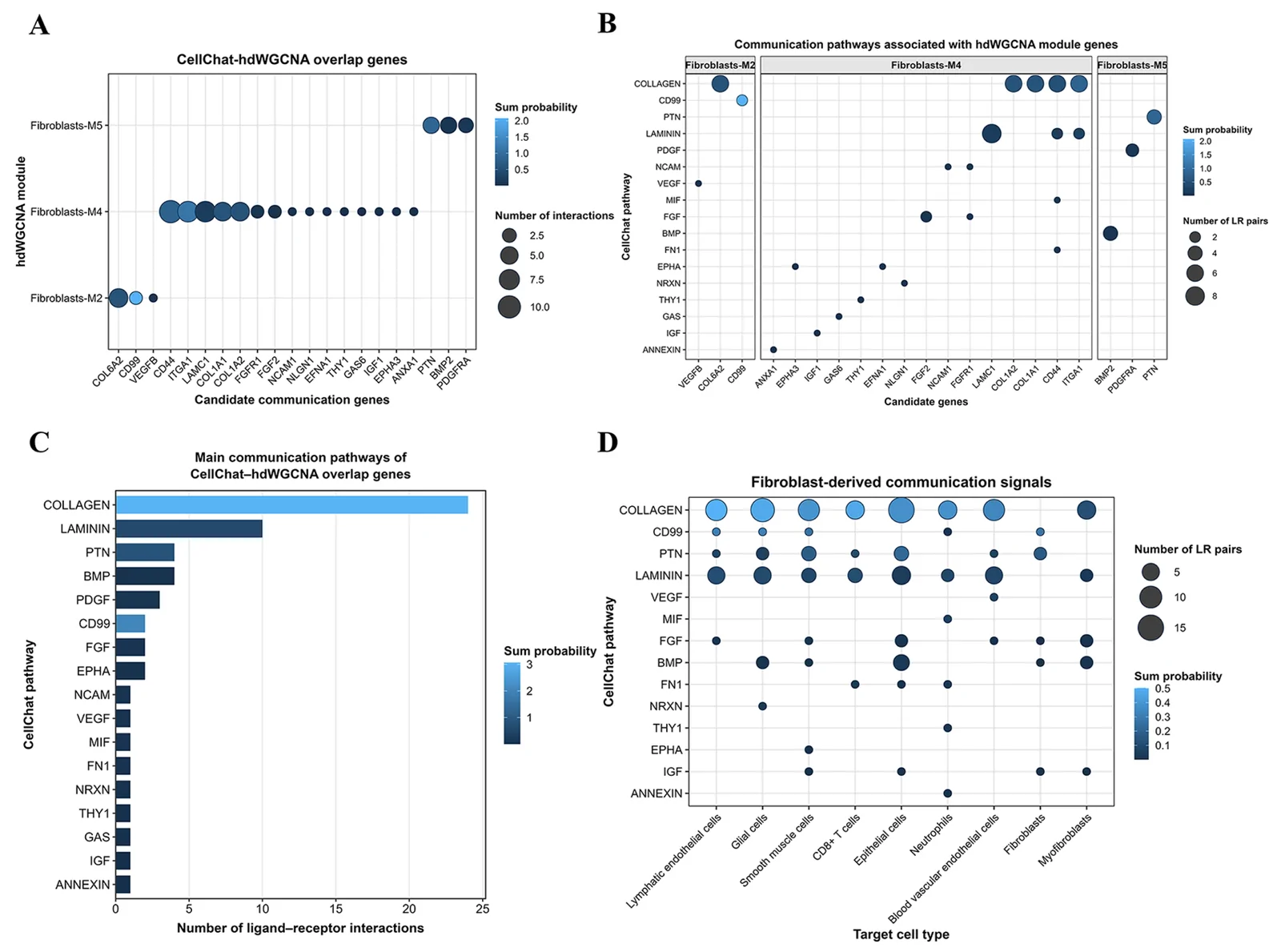
Communication-related candidate genes identified through integrated CellChat and hdWGCNA analyses. (**A**) Numbers and module assignments of genes overlapping between fibroblast hdWGCNA modules and CellChat ligand–receptor-related genes; (**B**) predicted signaling pathways associated with each candidate gene; (**C**) numbers of predicted ligand–receptor interactions across candidate-associated signaling pathways; and (**D**) predicted target-cell distributions with fibroblasts designated as signaling senders. Bubble size represents the number of predicted ligand–receptor interactions, and color represents the summed model-derived communication probability.

To connect fibroblast co-expression programs with predicted cell–cell communication, ligand–receptor-related genes retained in the CellChat analysis at *p* < 0.05 were intersected with genes from the five fibroblast hdWGCNA modules. The resulting overlapping genes were distributed across Fibroblasts-M2, Fibroblasts-M4, and Fibroblasts-M5. A total of 21 overlapping genes were identified across these three modules (Figure 5A). Fibroblasts-M4 contained 15 genes, including *CD44*, *ITGA1*, *LAMC1*, *COL1A1*, *COL1A2*, *FGFR1*, *FGF2*, *NCAM1*, *NLGN1*, *EFNA1*, *THY1*, *GAS6*, *IGF1*, *EPHA3*, and *ANXA1*. Fibroblasts-M2 contained *COL6A2*, *CD99*, and *VEGFB*, whereas Fibroblasts-M5 contained *PTN*, *BMP2*, and *PDGFRA*. Detailed module assignments and predicted signaling partners are provided in Table S4.

To characterize the coordinated functions represented by these communication-associated modules, genes from M2, M4, and M5 were combined for functional enrichment analysis. The filtered GO terms included positive regulation of cell migration, blood vessel morphogenesis, regulation of angiogenesis, extracellular and interstitial matrix structures, basement membrane, collagen trimer, heparin binding, collagen binding, extracellular matrix binding, and integrin binding (Figure S7A). KEGG enrichment included cytoskeleton in muscle cells, focal adhesion, integrin signaling, ECM–receptor interaction, PI3K–Akt signaling, Wnt signaling, and regulation of the actin cytoskeleton (Figure S7B).

Integration with CellChat linked M4 genes to predicted COLLAGEN, LAMININ, FGF, FN1, MIF, NCAM, EPHA, IGF, GAS, THY1, and ANNEXIN signaling; M2 genes to COLLAGEN, CD99, and VEGF signaling; and M5 genes to PTN, BMP, and PDGF signaling (Figure 5B). Among the overlapping genes, *CD44*, *ITGA1*, *LAMC1*, *COL1A1*, *COL1A2*, *COL6A2*, and *PTN* were involved in multiple predicted ligand–receptor interactions or interactions with relatively high communication probabilities and were used to represent the connections between fibroblast co-expression modules and the predicted communication network.

COLLAGEN signaling contained the largest number of ligand–receptor interactions involving these representative genes, followed by LAMININ signaling. PTN, BMP, PDGF, FGF, EPHA, CD99, and VEGF signaling were also represented (Figure 5C). With fibroblasts as predicted signaling senders, COLLAGEN and LAMININ signaling connected fibroblasts with epithelial cells, smooth muscle cells, blood vascular endothelial cells, lymphatic endothelial cells, glial cells, myofibroblasts, CD8^+^ T cells, and neutrophils (Figure 5D).

Together, the integrated CellChat and hdWGCNA analysis showed that communication-associated genes were primarily distributed across Fibroblasts-M4, Fibroblasts-M2, and Fibroblasts-M5. M4 was associated mainly with extracellular matrix- and adhesion-related signaling, M2 with COLLAGEN, CD99, and VEGF signaling, and M5 with PTN, BMP, and PDGF signaling. These patterns connected fibroblast co-expression programs with predicted extracellular matrix, epithelial, immune, developmental, and vascular signaling relationships in the uterine tissue.

### 3.8. Functional State Scoring Analysis of Major Cell Types

**Figure 6:**
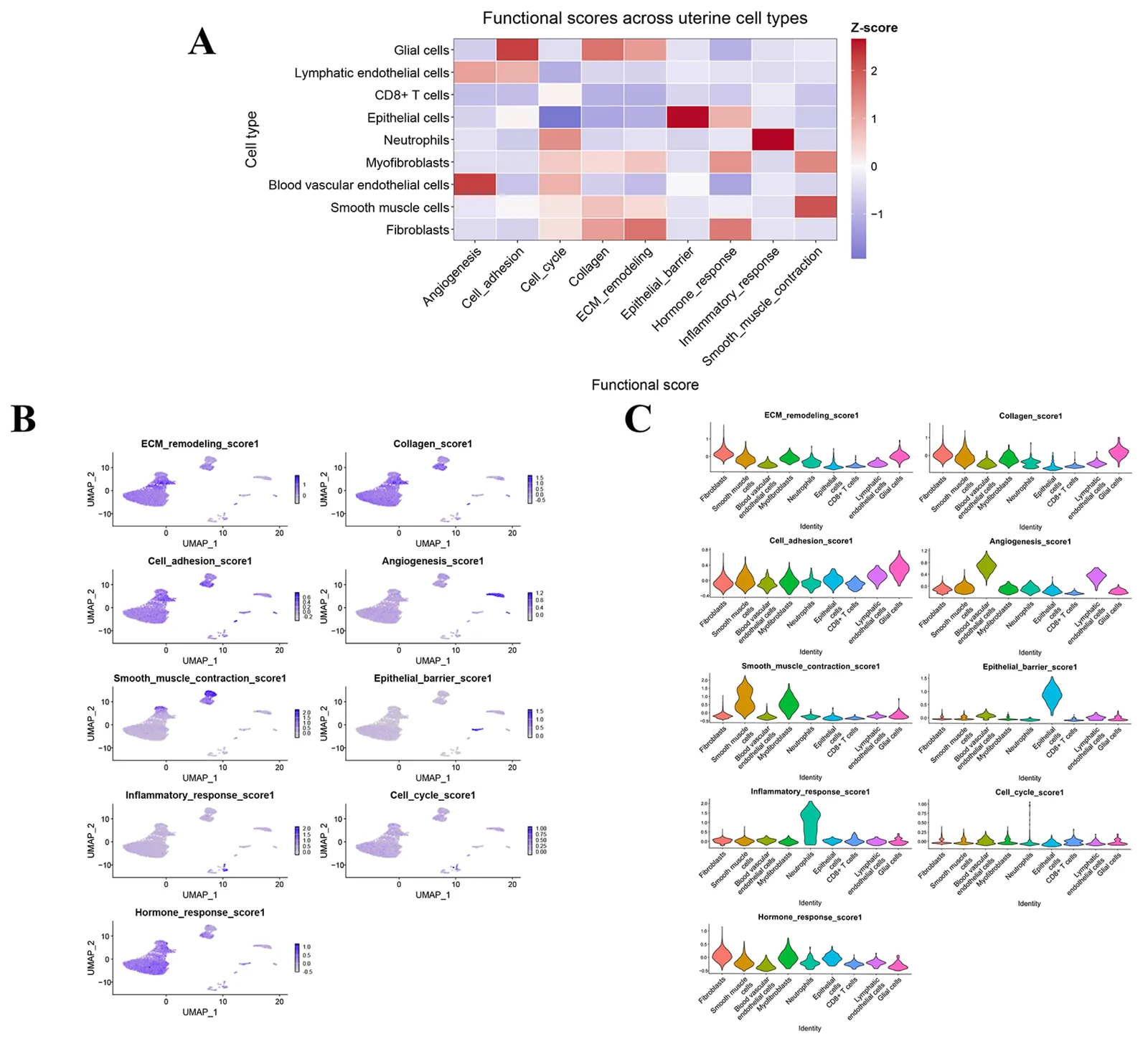
Functional state scoring analysis of major uterine cell types. (**A**) Heatmap of cell-type mean scores; rows represent predefined gene sets, columns represent cell types, and color indicates relative score. (**B**) UMAP maps showing cell-level score distributions in the single specimen. (**C**) Violin plots showing the distribution of cell-level scores within each annotated population.

To compare coordinated functional transcriptional programs among the major uterine cell types, nine predefined gene sets were evaluated at the single-cell level using AddModuleScore. Score distributions across individual cells and annotated cell types were summarized using heatmaps, UMAP plots, and violin plots (Figure 6).

Blood vascular endothelial cells had the highest angiogenesis score, while lymphatic endothelial cells also exhibited relatively high angiogenesis-related scores, highlighting vascular-development- and organization-associated transcriptional features. Smooth muscle cells had the highest smooth muscle contraction score, and myofibroblasts also showed elevated scores, reflecting their contractile and tissue-tension-associated characteristics. Epithelial cells had the highest epithelial barrier score, highlighting cell-junction- and epithelial-integrity-associated transcriptional features. Neutrophils exhibited the highest inflammatory-response score, reflecting their immune- and inflammation-associated expression program.

Fibroblasts exhibited relatively high ECM-remodeling, collagen, and hormone-response scores, consistent with the matrix- and signaling-associated patterns identified by the enrichment, hdWGCNA, and CellChat analyses. The UMAP and violin-plot distributions further showed that angiogenesis, smooth muscle contraction, epithelial barrier, and inflammatory-response programs were concentrated primarily in blood vascular endothelial cells, smooth muscle cells, epithelial cells, and neutrophils, respectively. Collectively, the functional scores summarized coordinated transcriptional programs among the uterine cell types and corresponded closely with their marker-based annotations. The gene sets used for scoring and the corresponding cell-type mean scores are provided in Tables S5 and S6, respectively.

## 4. Discussion

### 4.1. Major Findings and Their Biological Significance

This study used scRNA-seq to characterize the cellular composition and transcriptional features of uterine tissue from a 70-day-old Kangda V-line female rabbit. The most prominent feature of the observed cellular composition was the relatively high proportion of fibroblasts, which substantially exceeded that of the other cell types and represented the predominant population in this specimen. Fibroblasts are major components of connective tissue and the stromal microenvironment, with established functions in extracellular matrix synthesis, collagen deposition, tissue support, cell adhesion, and tissue remodeling. Previous studies have shown that the extracellular matrix is closely associated with pregnancy establishment, maintenance of uterine structural integrity, embryo adhesion, and regulation of trophoblast invasion [26]. In the present dataset, the high relative abundance of fibroblasts, together with their extracellular matrix- and collagen-associated transcriptional features and predicted signaling relationships, highlighted their potential involvement in uterine stromal organization and microenvironmental regulation through extracellular matrix deposition, cell–matrix interactions, and developmental signaling.

From a biological perspective, the uterus is a highly dynamic organ that undergoes marked structural changes during development, sexual maturation, estrus, mating, embryo implantation, pregnancy expansion, and parturition. In highly prolific, multiparous species such as rabbits, the uterus requires substantial tissue extensibility, matrix-remodeling capacity, and vascular support to accommodate the simultaneous implantation and development of multiple embryos [27]. Therefore, the predominance of fibroblasts observed in this study may indicate that the uterus of this 70-day-old Kangda V-line female rabbit was at a developmental stage characterized by stromal framework establishment, extracellular matrix deposition, and tissue structural maturation. Collectively, the functional enrichment, hdWGCNA, and CellChat analyses provided complementary information about fibroblast-associated transcriptional features in this uterine specimen. Functional enrichment indicated associations with extracellular matrix organization, collagen structure, cell adhesion, tissue development, and vascular regulation, while Fibroblasts-M2, M4, and M5 contained genes overlapping with ligand–receptor-related genes retained at an unadjusted empirical permutation *p* value < 0.05. Consistently, CellChat predicted fibroblast-associated communication through COLLAGEN, LAMININ, PTN, BMP, PDGF, FGF, and VEGF signaling. Candidate genes including *COL1A1*, *COL1A2*, *COL6A2*, *ITGA1*, *LAMC1*, *CD44*, *PTN*, *BMP2*, and *PDGFRA* may be involved in collagen deposition, basement-membrane formation, cell adhesion, vascular regulation, and developmental signaling. Taken together, these convergent findings provide candidate transcriptional and communication features consistent with stromal framework establishment, extracellular matrix remodeling, and tissue structural maturation in this uterine specimen.

### 4.2. Comparison with Single-Cell Transcriptomic Studies of the Uterus in Other Mammals

Cross-species comparisons provide a useful framework for understanding the conservation and species-specific characteristics of uterine cellular composition among mammals. Table 3 summarizes the uterine cell types and their relative proportions reported in rabbits (this study), cattle, pigs, humans, and mice. The resolution and nomenclature of cell-type classification vary among studies. For example, stromal cells represent a broad category of supporting cells that may encompass fibroblasts, smooth muscle-related cells, and other mesenchymal populations. Overall, stromal/fibroblast cells, epithelial cells, smooth muscle cells, blood vascular endothelial cells, lymphatic endothelial cells, and immune cells represent the major cell lineages commonly identified in mammalian uterine tissues. Most of the cell types identified in the rabbit uterus in the present study had broadly comparable counterparts in other mammalian species. For example, the porcine Stromal 1 population broadly corresponds to the fibroblast populations identified in rabbits and cattle, whereas the Stromal 2 population, which exhibits contractile and differentiation-associated characteristics, closely resembles the myofibroblast population identified in the present study. Similarly, the myocyte population reported in the developing mouse uterus is comparable to the smooth muscle cell population identified in the rabbit uterus. These findings suggest that, despite interspecies differences in uterine anatomy, reproductive strategies, and pregnancy characteristics, the principal cellular framework of the mammalian uterus is relatively conserved.

In terms of cellular composition proportions, the most prominent feature of the rabbit uterus in the present study was the high proportion of fibroblasts, which accounted for 76.6% of all cells, whereas epithelial and immune cells were relatively less abundant. This finding may be related to the developmental stage of the rabbit examined. Previous reproductive studies have shown that female rabbits generally reach puberty at approximately 3.5 months of age and become capable of pregnancy at approximately 4–4.5 months of age [28]. Therefore, 70 days of age represents a critical prepubertal developmental window during which the endometrium may not yet have been exposed to regular cyclical stimulation by sex hormones. In comparison, a detectable population of undifferentiated mesothelial cells (1.3%) was identified in the neonatal/developing mouse uterus, reflecting the incomplete maturation of organ morphogenesis during embryonic development. In contrast, mesothelial cells were not identified as a distinct cell population in the 70-day-old prepubertal rabbit examined in the present study or in the adult human, bovine, and porcine uteri. As individuals progress toward sexual maturity and undergo estrous or menstrual cycles, epithelial cells become more finely differentiated in the adult bovine, porcine, and human uterus, with specialized ciliated and glandular epithelial populations being identified. In adult humans and cattle, repeated cyclical stimulation by progesterone and estrogen promotes the recruitment of highly specialized immune cells into the endometrium, contributing to the local immune barrier during the estrous or menstrual cycle and preparing for maternal–fetal immune tolerance before embryo implantation [3,29]. By contrast, the 70-day-old rabbit examined in the present study was in the transitional stage preceding puberty, and its hypothalamic–pituitary–gonadal axis was likely still undergoing maturation. Consequently, uterine immune cells at this stage may primarily perform basal immune-surveillance functions, and the complex, specialized immune microenvironment characteristic of sexually mature individuals may not yet have developed. The relatively high proportion of fibroblasts in this specimen may therefore be associated with stromal-framework formation and extracellular matrix deposition during this juvenile developmental stage, whereas epithelial specialization and immune-cell recruitment may be less pronounced than in sexually mature or pregnant uteri.

From phylogenetic and evolutionary perspectives, the pronounced activity of fibroblast-associated signaling may reflect distinctive aspects of lagomorph reproductive physiology. Domestic rabbits and mice both belong to the superorder Glires and are typical litter-bearing mammals with elongated tubular duplex or bicornuate uteri [30,31]. In the mouse atlas, stromal cells and myocytes together accounted for 62.9% of the cells, broadly corresponding to the combined proportion of stromal fibroblasts and smooth muscle cells in the rabbit uterus in the present study (87.5%). This similarity may reflect a cellular organization that supports multiple-embryo implantation, placental attachment, and extensive uterine stretching and remodeling during pregnancy. More importantly, unlike humans, which have regular menstrual cycles, and mice, which undergo spontaneous estrous cycles, domestic rabbits are induced ovulators, in which mating-associated stimulation triggers the neuroendocrine cascade leading to ovulation [32,33]. Before mating stimulation, the uterus of the female rabbit may remain poised to respond to subsequent reproductive signals and initiate remodeling, suggesting that its stromal fibroblasts possess substantial transcriptional plasticity. The pronounced activity of Wnt and PI3K–Akt signaling in fibroblasts observed in the present study may be associated with endometrial remodeling and the establishment of receptivity following reproductive activation. In addition, evolutionary differences in placental type among species may also be reflected in uterine cellular composition. Humans possess a highly invasive discoid hemochorial placenta characterized by extensive maternal–fetal interactions, which may be associated with the maintenance of a substantial stromal cell compartment capable of decidualization [34,35]. In contrast, cattle and pigs possess cotyledonary synepitheliochorial and diffuse epitheliochorial placentas, respectively; their embryos do not deeply invade the endometrium, and nutrient exchange relies substantially on interactions between microvilli on the epithelial surfaces [36,37]. These characteristics may partly explain the higher proportions of epithelial cell lineages in cattle and pigs than in the developing rabbit uterus. In summary, the high proportion of fibroblast-associated cell populations and their distinctive molecular expression profiles in the uterus of Kangda V-line rabbits may represent a physiological feature of this developmental stage and may also be related to the reproductive characteristics of litter-bearing, induced-ovulating lagomorphs.

### 4.3. Limitations and Future Studies

This study provides an initial single-cell characterization of uterine-body tissue from one 70-day-old female rabbit and describes its cellular composition, functional transcriptional features, predicted cell–cell communication patterns, and fibroblast co-expression modules. Several limitations remain. First, this study analyzed the single-cell transcriptome from a single biological specimen collected at only one developmental time point, 70 days of age, and therefore cannot reveal dynamic changes during rabbit uterine development. The observed cell-type proportions may also have been influenced by the anatomical sampling location, tissue dissociation and cell-recovery efficiency, and other individual-specific factors. Although 70 days may approximate the prepubertal stage, it is not equivalent to complete sexual maturity or an active reproductive stage. Future studies should include samples from multiple developmental and physiological stages to systematically characterize changes in cellular composition from uterine development and maturation to pregnancy. Particular attention should be given to whether the proportion of fibroblasts decreases or undergoes functional transformation with age and physiological state, and whether epithelial cells and immune cells increase markedly during estrus or embryo implantation.

Second, this study analyzed only the uterine tissue and thus a systematic comparison with other tissues was not feasible. Therefore, it remains unclear whether the candidate genes identified here are specific to uterine tissue. For example, *COL1A1*, *COL1A2*, *COL6A2*, *LAMC1*, *ITGA1*, and *CD44* are broadly involved in extracellular matrix, basement membrane, and cell adhesion functions and may also be highly expressed in matrix-rich tissues such as skin, muscle, blood vessels, lung, and intestine. *BMP2*, *PDGFRA*, *PTN*, *FGF2*, *FGFR1*, and *VEGFB* may also participate in the development, angiogenesis, and mesenchymal regulation of multiple tissues. Future studies should integrate rabbit multi-tissue bulk RNA-seq, single-cell transcriptomic, or spatial transcriptomic data [4,38] to compare the expression patterns of these candidate genes across tissues and cell types.

Finally, the uterine single-cell transcriptomics should be further integrated with the phenotypes of reproductive traits in rabbits. The Kangda V-line rabbit is a meat rabbit line with practical production value. Uterine developmental status, embryo implantation capacity, pregnancy maintenance, and litter size may all be associated with uterine cellular composition and intercellular communication. Future studies should be conducted to compare uterine single-cell atlases from high- and low-prolificacy individuals, different parities, different pregnancy stages, and different rabbit lines to identify key cell types and candidate genes associated with reproductive performance. Integration of GWAS, eQTL, bulk RNA-seq, spatial transcriptomics, and phenotypic data may further clarify the molecular mechanisms underlying rabbit reproductive performance from the perspective of cell types, candidate genes, regulatory pathways, and reproductive traits.

## 5. Conclusions

In summary, this study provides a preliminary characterization of the cellular composition, fibroblast-associated functional features, and predicted intercellular communication patterns of uterine tissue from a 70-day-old Kangda V-line female rabbit. Fibroblasts accounted for 76.6% of the analyzed cells and represented the predominant cell population in this specimen, with expression patterns associated with extracellular matrix remodeling, cell adhesion, vascular regulation, and developmental signaling. Compared with published studies in humans, mice, cattle, and pigs, the major cell-type composition showed broad conservation, whereas the relatively high proportion of fibroblasts may be related to the developmental stage, reproductive characteristics, and stromal remodeling requirements of the rabbit uterus. This study provides a preliminary single-cell transcriptomic resource for future investigations of rabbit uterine development, embryo implantation, pregnancy maintenance, and the regulatory mechanisms underlying reproductive traits.

## Figures and Tables

**Table 1:** Numbers and proportions of major cell types identified in the rabbit uterus.

Cell Type	Number of Cells	Proportion (%)
Fibroblasts	7088	76.6
Smooth muscle cells	1007	10.9
Blood vascular endothelial cells	332	3.6
Myofibroblasts	273	3.0
Epithelial cells	138	1.5
Neutrophils	134	1.4
CD8^+^ T cells	132	1.4
Lymphatic endothelial cells	67	0.7
Glial cells	66	0.7
Unknown cells	14	0.2

**Table 2:** Numbers of cell-type-specific genes in different cell types.

Cell Type	Number of Cell-Type-Specific Genes
Fibroblasts	1899
Smooth muscle cells	1972
Blood vascular endothelial cells	1659
Myofibroblasts	820
Epithelial cells	2069
Neutrophils	1909
CD8^+^ T cells	1921
Lymphatic endothelial cells	1774
Glial cells	737

**Table 3:** Comparison of rabbit uterine cell types with cell types reported in other mammals.

Rabbit	Cattle * [12]	Pig [13]	Human [14]	Mouse [15]
Fibroblasts (76.6%)	Fibroblasts	Stromal cell 1 (25.7%)	Stromal (62.8%)	Stromal (35.3%)
Smooth muscle cells (10.9%)	Smooth muscle cells	Stromal cell 2 (6.8%)	Smooth muscle cells (1.8%)	Myocytes (27.6%)
Blood vascular endothelial cells (3.6%)	Blood vascular endothelial cells	Epithelial cell_rising estrogen level (22.8%)	Endothelial (1.0%)	Endothelial (5.9%)
Lymphatic endothelial cells (0.7%)	Lymphatic endothelial cells	Luminal cells (39.5%)	Lymphatic (0.2%)	Epithelial (23.4%)
Myofibroblasts (3.0%)	Other epithelial cells	Ciliated cells (0.4%)	Epithelium (25.6%)	Myeloid (0.6%)
Epithelial cells (1.5%)	Neutrophils	CD8 T cells (0.6%)	Immune myeloid (2.5%)	Lymphocytes (1.7%)
Neutrophils (1.4%)	Macrophages	Unknown (4.1%)	Immune lymphoid (5.8%)	Pericytes (4.2%)
CD8^+^ T cells (1.4%)	Mast cells		Undefined (0.3%)	Mesothelial (1.3%)
Glial cells (0.7%)	CD8^+^ T cells			
Unknown (0.2%)	Cytotoxic CD8^+^ T cells			
	WC1− γδ T cells			
	Proliferative T cells			
	NK T cells			
	NK cells			
	B cells			
	Plasma cells			
	Pericytes			
	Glial cells			

Note: The ages of the individuals from which the uterus was sampled in the referenced studies were as follows: rabbit, 70 days; cattle, 3.5 years; pig, 6 months; human, adult; mouse, postnatal day 12. * The cattle study did not provide complete cell-type proportions.

## Data Availability

The datasets generated and analyzed during the current study are not publicly available because they are part of an ongoing research project. Data may be made available from the corresponding authors upon reasonable request and with permission from the project investigators.

## Supplementary Materials

The following supporting information can be downloaded at: https://www.mdpi.com/article/10.3390/genes17091131/s1, Figure S1: Quality control, principal-component evaluation, and doublet identification in the single rabbit uterine-body dataset; Figure S2: Marker-gene evidence supporting cell-type annotation; Figure S3: GO and KEGG enrichment analyses of smooth muscle cell marker genes; Figure S4: GO and KEGG enrichment analyses of blood vascular endothelial cell marker genes; Figure S5: GO and KEGG enrichment analyses of neutrophil marker genes; Figure S6: GO enrichment analysis of epithelial cell marker genes; Figure S7: GO and KEGG enrichment analyses of communication-associated fibroblast co-expression modules; Table S1: Signaling pathways predicted by CellChat using an unadjusted empirical permutation *p* value < 0.05; Table S2: Summary of fibroblast hdWGCNA modules; Table S3: Top 10 hub genes in each fibroblast hdWGCNA module; Table S4: Communication-associated candidate genes identified by integrating CellChat interactions retained at an unadjusted empirical permutation *p* value < 0.05 with fibroblast hdWGCNA results; Table S5: Gene sets used for descriptive functional scoring with Seurat AddModuleScore; Table S6: Mean descriptive functional module scores for each uterine cell type; Table S7: Comprehensive positive marker output for the nine annotated cell populations.
