# Supplementary material for: Single-Cell RNA Sequencing of Uterine Tissue from a 70-Day-Old Female Rabbit: Cellular Composition and Fibroblast-Associated Networks
Source: Genes (Basel). 2026 Sep 16;17(9):1131. doi: 10.3390/genes17091131 (PMC13606278; doi:10.3390/genes17091131)
Supplement: Supplementary file 1 [file genes-17-01131-s001.zip › Table S7.pdf]

Table S7. Comprehensive positive marker output for the nine annotated cell populations.

Original cluster	Annotated cell type	Gene	avg_log2FC	pct.1	pct.2	Raw P value	Bonferroni-adjusted P value
12	**Fibroblasts**	*LOC138848040*	3.6197	0.972	0.899	2.214e-45	5.113e-41
4	Fibroblasts	*IGFBP5*	2.8958	0.995	0.608	5.336e-274	1.232e-269
3	Fibroblasts	*LUM*	2.7308	0.584	0.160	4.048e-284	9.348e-280
3	Fibroblasts	*GSN*	2.4919	0.825	0.492	4.489e-226	1.037e-221
3	Fibroblasts	*PI16*	2.3482	0.549	0.034	0	0
2	Fibroblasts	*GABRG3*	2.0334	0.727	0.170	0	0
3	Fibroblasts	*C7*	2.0284	0.456	0.019	0	0
3	Fibroblasts	*RSPO3*	2.0206	0.688	0.109	0	0
3	Fibroblasts	*OGN*	1.8587	0.943	0.544	0	0
3	Fibroblasts	*ZFPM2*	1.7134	0.638	0.148	0	0
2	Fibroblasts	*NRXN3*	1.6815	0.714	0.241	0	0
1	Fibroblasts	*NTM*	1.6702	0.605	0.153	0	0
1	Fibroblasts	*TENM2*	1.6696	0.495	0.074	0	0
4	Fibroblasts	*RALYL*	1.6171	0.545	0.094	3.928e-221	9.070e-217
4	Fibroblasts	*IGFBP6*	1.5745	0.784	0.310	4.640e-155	1.072e-150
1	Fibroblasts	*ANO4*	1.5645	0.805	0.291	0	0
3	Fibroblasts	*PLOD2*	1.5561	0.515	0.120	1.458e-253	3.368e-249
2	Fibroblasts	*ST6GALNAC3*	1.5262	0.958	0.585	0	0
3	Fibroblasts	*CCDC102B*	1.5261	0.246	0.037	5.282e-160	1.220e-155
3	Fibroblasts	*BNC2*	1.5089	0.717	0.299	2.720e-214	6.280e-210
3	Fibroblasts	*SLIT3*	1.4923	0.527	0.122	1.674e-235	3.865e-231
4	Fibroblasts	*SPHKAP*	1.4921	0.527	0.091	9.623e-220	2.222e-215
3	Fibroblasts	*PLSCR4*	1.4792	0.905	0.597	3.028e-250	6.993e-246
4	Fibroblasts	*SEMA3C*	1.4675	0.432	0.055	2.910e-232	6.719e-228
3	Fibroblasts	*THBS1*	1.4289	0.418	0.118	1.126e-152	2.599e-148
3	Fibroblasts	*MGP*	1.4160	0.557	0.332	2.549e-63	5.886e-59
2	Fibroblasts	*LOC138846189*	1.4085	0.823	0.381	1.234e-284	2.849e-280
2	Fibroblasts	*PGR*	1.4073	0.872	0.446	1.050e-262	2.426e-258
3	Fibroblasts	*LOC100357148*	1.4023	0.647	0.283	2.577e-173	5.950e-169
3	Fibroblasts	*NLGN1*	1.3884	0.293	0.106	4.710e-70	1.088e-65
3	Fibroblasts	*MAST4*	1.3785	0.589	0.285	5.993e-133	1.384e-128
3	Fibroblasts	*CXCL12*	1.3627	0.413	0.027	0	0
3	Fibroblasts	*DCN*	1.3465	0.988	0.838	3.659e-261	8.450e-257
3	Fibroblasts	*NAALADL2*	1.3292	0.843	0.586	3.432e-132	7.925e-128
3	Fibroblasts	*NCAM1*	1.3241	0.541	0.155	6.994e-213	1.615e-208
2	Fibroblasts	*GALNTL6*	1.3060	0.325	0.103	9.007e-123	2.080e-118
4	Fibroblasts	*GPC5*	1.2959	0.345	0.109	3.772e-63	8.710e-59
3	Fibroblasts	*AKAP12*	1.2859	0.643	0.316	2.228e-142	5.145e-138

Original cluster	Annotated cell type	Gene	avg_log2FC	pct.1	pct.2	Raw P value	Bonferroni-adjusted P value
3	Fibroblasts	*COL14A1*	1.2777	0.892	0.671	4.601e-171	1.063e-166
2	Fibroblasts	*SYT1*	1.2728	0.921	0.529	1.716e-273	3.962e-269
1	Fibroblasts	*NTNG1*	1.2508	0.528	0.140	6.650e-284	1.536e-279
1	Fibroblasts	*CYP7B1*	1.2425	0.886	0.512	2.626e-244	6.064e-240
3	Fibroblasts	*IGFBP7*	1.2291	0.822	0.483	2.403e-158	5.549e-154
2	Fibroblasts	*LUZP2*	1.2226	0.409	0.133	1.920e-150	4.434e-146
0	Fibroblasts	*PENK*	1.2093	0.952	0.541	0	0
4	Fibroblasts	*DLK1*	1.2007	0.461	0.091	1.189e-161	2.745e-157
3	Fibroblasts	*CELF2*	1.2003	0.831	0.572	1.729e-135	3.993e-131
3	Fibroblasts	*EPHA3*	1.1994	0.405	0.089	2.614e-184	6.036e-180
2	Fibroblasts	*G0S2*	1.1839	0.728	0.321	5.109e-215	1.180e-210
3	Fibroblasts	*DCLK1*	1.1796	0.547	0.228	3.977e-126	9.183e-122
12	Fibroblasts	*PRKCA*	1.1730	0.653	0.431	1.860e-15	4.295e-11
2	Fibroblasts	*NEGR1*	1.1463	0.974	0.734	7.632e-272	1.762e-267
2	Fibroblasts	*LAMA2*	1.1454	0.926	0.511	4.394e-300	1.015e-295
2	Fibroblasts	*DPT*	1.1429	0.816	0.290	0	0
3	Fibroblasts	*TXNIP*	1.1331	0.949	0.829	2.010e-174	4.642e-170
3	Fibroblasts	*MFAP5*	1.1323	0.291	0.027	3.590e-268	8.291e-264
2	Fibroblasts	*PTPRD*	1.1313	0.922	0.619	8.997e-235	2.078e-230
3	Fibroblasts	*CDH18*	1.1309	0.190	0.012	5.786e-206	1.336e-201
3	Fibroblasts	*LTBP4*	1.1127	0.768	0.488	4.205e-137	9.711e-133
3	Fibroblasts	*ABI3BP*	1.1027	0.509	0.196	9.529e-128	2.200e-123
2	Fibroblasts	*EPHA5*	1.1025	0.401	0.092	1.599e-219	3.692e-215
4	Fibroblasts	*CEBPD*	1.1013	0.861	0.703	8.487e-43	1.960e-38
2	Fibroblasts	*RORB*	1.0977	0.435	0.142	7.461e-165	1.723e-160
1	Fibroblasts	*NKAIN2*	1.0843	0.284	0.074	7.231e-140	1.670e-135
2	Fibroblasts	*TPBG*	1.0661	0.678	0.295	1.754e-197	4.051e-193
4	Fibroblasts	*CFD*	1.0579	0.859	0.592	3.817e-58	8.814e-54
1	Fibroblasts	*RXFP1*	1.0556	0.704	0.321	4.387e-207	1.013e-202
12	Fibroblasts	*COL3A1*	1.0533	0.833	0.681	3.523e-17	8.135e-13
3	Fibroblasts	*LSAMP*	1.0366	0.305	0.090	1.890e-97	4.365e-93
3	Fibroblasts	*NFIA*	1.0294	0.831	0.611	9.787e-137	2.260e-132
3	Fibroblasts	*HDAC9*	1.0246	0.562	0.300	4.637e-82	1.071e-77
3	Fibroblasts	*ADAMTS19*	1.0223	0.229	0.033	1.490e-151	3.440e-147
12	Fibroblasts	*LOC127484872*	1.0201	0.799	0.651	3.666e-18	8.466e-14
2	Fibroblasts	*DOCK10*	1.0182	0.587	0.213	2.756e-201	6.365e-197
4	Fibroblasts	*C1R*	1.0177	0.646	0.266	5.978e-94	1.380e-89
12	Fibroblasts	*SEMA6D*	1.0171	0.736	0.528	4.118e-15	9.508e-11
3	Fibroblasts	*SVEP1*	1.0163	0.262	0.048	7.566e-143	1.747e-138
2	Fibroblasts	*TMTC2*	1.0123	0.547	0.214	3.638e-171	8.401e-167

Original cluster	Annotated cell type	Gene	avg_log2FC	pct.1	pct.2	Raw P value	Bonferroni-adjusted P value
3	Fibroblasts	*C1H11orf96*	1.0065	0.549	0.257	8.636e-88	1.994e-83
2	Fibroblasts	*PLEKHA5*	1.0061	0.837	0.484	8.066e-205	1.863e-200
2	Fibroblasts	*SAMD5*	1.0055	0.822	0.436	4.458e-193	1.029e-188
12	Fibroblasts	*ROBO2*	0.9903	0.604	0.414	2.662e-11	6.148e-07
3	Fibroblasts	*MEIS1*	0.9874	0.613	0.297	8.681e-119	2.005e-114
3	Fibroblasts	*GPC3*	0.9820	0.271	0.038	2.513e-183	5.802e-179
4	Fibroblasts	*PLPP3*	0.9817	0.993	0.783	3.035e-109	7.009e-105
3	Fibroblasts	*IGF1*	0.9689	0.635	0.460	3.005e-54	6.940e-50
3	Fibroblasts	*GHR*	0.9647	0.681	0.515	9.496e-56	2.193e-51
12	Fibroblasts	*FAP*	0.9631	0.458	0.277	3.129e-11	7.225e-07
3	Fibroblasts	*ASPN*	0.9624	0.280	0.031	8.428e-226	1.946e-221
2	Fibroblasts	*ZNF804A*	0.9595	0.179	0.033	7.228e-112	1.669e-107
4	Fibroblasts	*HOXA13*	0.9555	0.389	0.033	2.895e-283	6.685e-279
3	Fibroblasts	*UNC5C*	0.9539	0.591	0.490	1.569e-26	3.623e-22
12	Fibroblasts	*OGT*	0.9480	0.556	0.357	2.760e-14	6.373e-10
4	Fibroblasts	*HMOX1*	0.9480	0.743	0.441	8.931e-57	2.062e-52
2	Fibroblasts	*RARRES1*	0.9469	0.992	0.728	2.062e-260	4.762e-256
4	Fibroblasts	*RARRES2*	0.9373	0.882	0.525	2.197e-91	5.074e-87
12	Fibroblasts	*ZNF385D*	0.9332	0.653	0.508	7.905e-10	1.825e-05
4	Fibroblasts	*PID1*	0.9293	0.936	0.577	2.902e-89	6.702e-85
12	Fibroblasts	*LOC127491587*	0.9272	0.222	0.069	4.875e-14	1.126e-09
2	Fibroblasts	*FREM1*	0.9245	0.791	0.398	1.999e-198	4.617e-194
2	Fibroblasts	*LOC103345489*	0.9209	0.515	0.161	3.651e-200	8.430e-196
3	Fibroblasts	*ADAMTSL1*	0.9151	0.476	0.311	3.554e-40	8.206e-36
2	Fibroblasts	*ROBO1*	0.9141	0.660	0.319	4.671e-156	1.079e-151
3	Fibroblasts	*SLIT2*	0.9127	0.443	0.229	1.117e-58	2.579e-54
3	Fibroblasts	*RNASE4*	0.9058	0.899	0.747	1.889e-125	4.362e-121
3	Fibroblasts	*FGF14*	0.9053	0.309	0.174	3.201e-30	7.391e-26
3	Fibroblasts	*GAS1*	0.9042	0.727	0.520	9.889e-67	2.284e-62
4	Fibroblasts	*BST2*	0.9032	0.541	0.298	3.529e-38	8.148e-34
3	Fibroblasts	*LHFPL6*	0.9018	0.751	0.475	6.232e-102	1.439e-97
3	Fibroblasts	*SPARCL1*	0.9017	0.932	0.767	1.883e-144	4.348e-140
3	Fibroblasts	*DKK2*	0.8975	0.217	0.036	4.830e-125	1.115e-120
2	Fibroblasts	*RBMS3*	0.8929	0.885	0.609	1.913e-177	4.419e-173
2	Fibroblasts	*METTL24*	0.8890	0.702	0.361	4.499e-147	1.039e-142
3	Fibroblasts	*LINGO2*	0.8882	0.138	0.045	1.781e-36	4.113e-32
2	Fibroblasts	*GRID2*	0.8844	0.458	0.249	4.323e-72	9.982e-68
4	Fibroblasts	*MAFB*	0.8825	0.491	0.243	1.103e-39	2.547e-35
4	Fibroblasts	*LOC100343802*	0.8810	0.443	0.253	7.173e-22	1.656e-17
4	Fibroblasts	*ITM2A*	0.8720	0.857	0.589	1.683e-53	3.887e-49

Original cluster	Annotated cell type	Gene	avg_log2FC	pct.1	pct.2	Raw P value	Bonferroni-adjusted P value
0	Fibroblasts	*PTGDS*	0.8679	0.785	0.532	9.449e-147	2.182e-142
12	Fibroblasts	*PREX2*	0.8662	0.618	0.453	7.926e-10	1.830e-05
4	Fibroblasts	*MARCKS*	0.8600	0.932	0.750	7.909e-67	1.826e-62
4	Fibroblasts	*S100A6*	0.8596	0.946	0.786	1.798e-58	4.152e-54
3	Fibroblasts	*GLI3*	0.8573	0.360	0.121	2.805e-100	6.478e-96
2	Fibroblasts	*PPP2R2B*	0.8569	0.749	0.395	2.490e-147	5.749e-143
2	Fibroblasts	*EPHA7*	0.8560	0.545	0.263	7.296e-115	1.685e-110
4	Fibroblasts	*LOC100343299*	0.8548	0.259	0.123	1.107e-20	2.555e-16
12	Fibroblasts	*LOC138843476*	0.8545	0.507	0.309	1.858e-11	4.290e-07
1	Fibroblasts	*PDE11A*	0.8500	0.471	0.187	1.568e-141	3.621e-137
3	Fibroblasts	*DDIT4L*	0.8469	0.292	0.137	8.090e-45	1.868e-40
0	Fibroblasts	*DIO3*	0.8413	0.600	0.403	5.106e-74	1.179e-69
3	Fibroblasts	*TGFBI*	0.8359	0.315	0.084	1.447e-117	3.342e-113
3	Fibroblasts	*PTPRK*	0.8333	0.558	0.339	1.150e-58	2.657e-54
3	Fibroblasts	*RYR3*	0.8333	0.267	0.075	2.677e-88	6.183e-84
4	Fibroblasts	*PRSS23*	0.8300	0.520	0.167	3.196e-93	7.381e-89
0	Fibroblasts	*SFRP2*	0.8269	0.947	0.589	5.500e-230	1.270e-225
4	Fibroblasts	*GALNT13*	0.8198	0.459	0.169	1.115e-63	2.575e-59
3	Fibroblasts	*PRELP*	0.8150	0.230	0.016	2.826e-244	6.525e-240
3	Fibroblasts	*GYPC*	0.8149	0.565	0.352	3.501e-65	8.086e-61
3	Fibroblasts	*NTRK2*	0.8128	0.449	0.235	9.757e-63	2.253e-58
3	Fibroblasts	*LOC103350594*	0.8098	0.813	0.697	9.552e-52	2.206e-47
3	Fibroblasts	*ITM2C*	0.8091	0.392	0.226	2.097e-44	4.843e-40
4	Fibroblasts	*LOC138848296*	0.8083	0.488	0.216	1.823e-51	4.209e-47
3	Fibroblasts	*COL1A2*	0.8083	0.678	0.557	3.511e-42	8.108e-38
3	Fibroblasts	*RERG*	0.8063	0.340	0.180	9.947e-41	2.297e-36
3	Fibroblasts	*FGF2*	0.8049	0.413	0.192	7.135e-71	1.648e-66
3	Fibroblasts	*KCNB2*	0.8038	0.168	0.020	5.598e-123	1.293e-118
3	Fibroblasts	*FOXP2*	0.7997	0.221	0.076	3.997e-53	9.230e-49
3	Fibroblasts	*LOC100357917*	0.7991	0.479	0.244	5.612e-78	1.296e-73
3	Fibroblasts	*FBLN1*	0.7978	0.845	0.698	2.582e-61	5.963e-57
12	Fibroblasts	*RORA*	0.7965	0.819	0.698	2.980e-14	6.881e-10
2	Fibroblasts	*CSMD3*	0.7963	0.299	0.116	5.121e-79	1.183e-74
3	Fibroblasts	*COL1A1*	0.7962	0.766	0.610	1.488e-65	3.435e-61
2	Fibroblasts	*CPED1*	0.7955	0.572	0.265	1.068e-122	2.467e-118
12	Fibroblasts	*NBEA*	0.7945	0.688	0.572	4.157e-09	9.600e-05
2	Fibroblasts	*PRR16*	0.7917	0.686	0.381	4.974e-119	1.149e-114
2	Fibroblasts	*ATRNL1*	0.7897	0.858	0.549	3.146e-142	7.265e-138
4	Fibroblasts	*SERPINE1*	0.7889	0.250	0.071	4.978e-52	1.149e-47
1	Fibroblasts	*CNKSR2*	0.7885	0.401	0.174	1.275e-98	2.944e-94

Original cluster	Annotated cell type	Gene	avg_log2FC	pct.1	pct.2	Raw P value	Bonferroni-adjusted P value
3	Fibroblasts	*SERPING1*	0.7850	0.549	0.339	1.736e-62	4.008e-58
12	Fibroblasts	*WASF1*	0.7845	0.625	0.489	2.943e-07	6.797e-03
3	Fibroblasts	*MFAP4*	0.7831	0.352	0.133	7.869e-81	1.817e-76
4	Fibroblasts	*FBLN2*	0.7830	0.537	0.212	1.559e-69	3.601e-65
2	Fibroblasts	*GPC6*	0.7829	0.561	0.267	3.914e-116	9.039e-112
12	Fibroblasts	*HGF*	0.7816	0.410	0.267	8.140e-08	1.880e-03
2	Fibroblasts	*PRKD1*	0.7801	0.539	0.282	4.191e-94	9.677e-90
12	Fibroblasts	*SLC8A1*	0.7792	0.556	0.401	3.560e-07	8.220e-03
4	Fibroblasts	*RTP4*	0.7747	0.443	0.173	3.440e-58	7.945e-54
4	Fibroblasts	*TIMP2*	0.7712	0.882	0.584	2.424e-66	5.599e-62
2	Fibroblasts	*ALCAM*	0.7706	0.403	0.165	6.981e-102	1.612e-97
2	Fibroblasts	*FANCC*	0.7697	0.487	0.239	1.598e-92	3.690e-88
2	Fibroblasts	*LOC103350560*	0.7690	0.616	0.315	8.386e-113	1.936e-108
1	Fibroblasts	*PTN*	0.7636	0.886	0.577	3.042e-155	7.024e-151
12	Fibroblasts	*COL15A1*	0.7634	0.431	0.233	5.463e-11	1.261e-06
12	Fibroblasts	*KEF51-p06*	0.7625	0.993	0.982	2.069e-29	4.778e-25
4	Fibroblasts	*GFPT2*	0.7586	0.329	0.133	2.446e-37	5.649e-33
12	Fibroblasts	*ATXN1*	0.7571	0.604	0.519	1.563e-07	3.610e-03
1	Fibroblasts	*BCL2*	0.7560	0.945	0.737	1.237e-150	2.858e-146
4	Fibroblasts	*NRXN1*	0.7552	0.243	0.046	7.481e-84	1.727e-79
3	Fibroblasts	*ADAMTSL3*	0.7552	0.195	0.024	5.218e-143	1.205e-138
3	Fibroblasts	*COL17A1*	0.7525	0.157	0.016	1.155e-133	2.666e-129
2	Fibroblasts	*BICC1*	0.7524	0.660	0.346	7.580e-123	1.750e-118
4	Fibroblasts	*IGFBP3*	0.7461	1.000	0.899	2.503e-84	5.780e-80
3	Fibroblasts	*PCDH7*	0.7457	0.536	0.368	1.717e-39	3.965e-35
3	Fibroblasts	*MEDAG*	0.7445	0.191	0.017	3.076e-175	7.103e-171
3	Fibroblasts	*GAB2*	0.7429	0.414	0.288	7.737e-26	1.787e-21
3	Fibroblasts	*LOX*	0.7396	0.280	0.102	3.219e-65	7.433e-61
2	Fibroblasts	*NOVA1*	0.7387	0.405	0.169	1.133e-96	2.616e-92
3	Fibroblasts	*GPX3*	0.7380	0.550	0.361	2.399e-52	5.541e-48
2	Fibroblasts	*SOX6*	0.7379	0.670	0.383	1.431e-103	3.304e-99
3	Fibroblasts	*CACNA1C*	0.7342	0.346	0.183	1.400e-38	3.233e-34
0	Fibroblasts	*COX6C*	0.7305	0.963	0.843	1.423e-227	3.286e-223
4	Fibroblasts	*S100A13*	0.7284	0.616	0.295	7.535e-61	1.740e-56
2	Fibroblasts	*PLA2R1*	0.7280	0.589	0.288	7.446e-120	1.719e-115
3	Fibroblasts	*JUN*	0.7262	0.902	0.850	1.290e-59	2.979e-55
4	Fibroblasts	*ZFHX4*	0.7261	0.420	0.146	2.114e-65	4.882e-61
3	Fibroblasts	*MED13L*	0.7260	0.757	0.648	1.470e-54	3.394e-50
4	Fibroblasts	*C1S*	0.7254	0.604	0.304	2.607e-51	6.020e-47
4	Fibroblasts	*LOC127486901*	0.7252	0.495	0.229	2.862e-43	6.608e-39

Original cluster	Annotated cell type	Gene	avg_log2FC	pct.1	pct.2	Raw P value	Bonferroni-adjusted P value
3	Fibroblasts	*LOC138850424*	0.7226	0.875	0.792	2.890e-59	6.674e-55
3	Fibroblasts	*YBX3*	0.7181	0.722	0.570	8.471e-59	1.956e-54
2	Fibroblasts	*PDGFD*	0.7149	0.354	0.147	5.583e-85	1.289e-80
2	Fibroblasts	*LOC100008920*	0.7125	0.345	0.117	2.811e-109	6.490e-105
12	Fibroblasts	*PLXDC2*	0.7120	0.722	0.567	5.606e-10	1.294e-05
1	Fibroblasts	*SULF1*	0.7078	0.742	0.482	1.355e-98	3.130e-94
3	Fibroblasts	*THY1*	0.7066	0.450	0.283	2.459e-41	5.679e-37
3	Fibroblasts	*CTSK*	0.7042	0.448	0.254	1.302e-53	3.007e-49
4	Fibroblasts	*PPP1R15A*	0.7041	0.746	0.513	2.221e-43	5.130e-39
3	Fibroblasts	*LTBP1*	0.7039	0.610	0.435	4.594e-46	1.061e-41
4	Fibroblasts	*EFEMP1*	0.7014	0.825	0.452	3.538e-63	8.171e-59
2	Fibroblasts	*NID1*	0.7013	0.814	0.540	9.864e-120	2.278e-115
4	Fibroblasts	*AOX1*	0.7002	0.345	0.102	5.516e-69	1.274e-64
3	Fibroblasts	*CDON*	0.7001	0.321	0.128	1.067e-64	2.464e-60
2	Fibroblasts	*CADPS2*	0.6996	0.306	0.093	3.595e-116	8.303e-112
12	Fibroblasts	*SEMA3A*	0.6992	0.167	0.070	1.841e-06	4.250e-02
1	Fibroblasts	*PLK2*	0.6987	0.566	0.357	4.126e-66	9.527e-62
3	Fibroblasts	*HPGD*	0.6974	0.176	0.012	1.069e-179	2.469e-175
3	Fibroblasts	*SFRP1*	0.6949	0.227	0.051	7.549e-99	1.743e-94
12	Fibroblasts	*ADHFE1*	0.6941	0.340	0.205	2.795e-07	6.453e-03
3	Fibroblasts	*GAS6*	0.6924	0.406	0.193	1.653e-65	3.817e-61
2	Fibroblasts	*BMP2*	0.6895	0.450	0.198	6.524e-97	1.507e-92
1	Fibroblasts	*ZNF804B*	0.6886	0.183	0.041	9.075e-100	2.096e-95
2	Fibroblasts	*FHIT*	0.6876	0.553	0.355	5.052e-56	1.167e-51
3	Fibroblasts	*CYP1B1*	0.6864	0.197	0.020	4.300e-165	9.929e-161
4	Fibroblasts	*COLEC12*	0.6842	0.595	0.275	9.308e-58	2.149e-53
3	Fibroblasts	*GRM8*	0.6840	0.166	0.050	4.388e-49	1.013e-44
2	Fibroblasts	*RAB27B*	0.6837	0.342	0.112	2.429e-114	5.610e-110
12	Fibroblasts	*KEF51-p03*	0.6809	0.729	0.673	1.625e-08	3.752e-04
4	Fibroblasts	*SCARA5*	0.6800	0.377	0.090	1.315e-96	3.037e-92
3	Fibroblasts	*LOC100340719*	0.6789	0.559	0.423	1.371e-36	3.165e-32
1	Fibroblasts	*NEBL*	0.6785	0.606	0.315	2.160e-111	4.988e-107
12	Fibroblasts	*KEF51-p08*	0.6756	1.000	0.994	4.600e-34	1.062e-29
3	Fibroblasts	*FZD1*	0.6740	0.259	0.083	3.098e-72	7.155e-68
2	Fibroblasts	*PRDM1*	0.6735	0.595	0.294	5.510e-113	1.272e-108
3	Fibroblasts	*TENM3*	0.6718	0.558	0.386	2.011e-39	4.645e-35
4	Fibroblasts	*CRISPLD1*	0.6704	0.550	0.364	2.455e-16	5.668e-12
4	Fibroblasts	*LOC138843198*	0.6686	0.246	0.020	3.781e-180	8.732e-176
4	Fibroblasts	*SERPINF1*	0.6670	0.293	0.051	1.724e-108	3.981e-104
1	Fibroblasts	*ESR1*	0.6669	0.938	0.748	8.396e-118	1.939e-113

Original cluster	Annotated cell type	Gene	avg_log2FC	pct.1	pct.2	Raw P value	Bonferroni-adjusted P value
4	Fibroblasts	*LOC103346136*	0.6665	0.500	0.244	9.572e-42	2.210e-37
3	Fibroblasts	*PGM5*	0.6655	0.205	0.044	3.594e-91	8.300e-87
12	Fibroblasts	*PAM*	0.6653	0.812	0.742	5.370e-11	1.240e-06
12	Fibroblasts	*KEF51-p13*	0.6648	1.000	0.986	1.547e-29	3.573e-25
1	Fibroblasts	*MAGI2*	0.6595	0.728	0.511	2.475e-72	5.715e-68
2	Fibroblasts	*MATN2*	0.6586	0.639	0.338	1.058e-107	2.442e-103
3	Fibroblasts	*CDK14*	0.6561	0.416	0.260	1.007e-33	2.326e-29
12	Fibroblasts	*BICRAL*	0.6560	0.410	0.271	1.676e-07	3.869e-03
4	Fibroblasts	*FCGRT*	0.6556	0.848	0.566	6.220e-54	1.436e-49
4	Fibroblasts	*NFIX*	0.6544	0.823	0.524	1.296e-46	2.994e-42
3	Fibroblasts	*LAMB1*	0.6530	0.257	0.079	1.696e-75	3.916e-71
0	Fibroblasts	*IGFBP2*	0.6514	0.768	0.539	9.696e-123	2.239e-118
3	Fibroblasts	*DEPP1*	0.6500	0.160	0.059	4.689e-34	1.083e-29
3	Fibroblasts	*OLFML1*	0.6491	0.210	0.026	1.298e-151	2.998e-147
3	Fibroblasts	*FNBP1L*	0.6470	0.424	0.259	2.553e-38	5.896e-34
3	Fibroblasts	*RUNX1T1*	0.6443	0.705	0.557	4.203e-45	9.705e-41
4	Fibroblasts	*OLFML3*	0.6441	0.382	0.109	2.943e-76	6.795e-72
3	Fibroblasts	*COL5A2*	0.6433	0.521	0.344	2.303e-41	5.317e-37
12	Fibroblasts	*CNTN3*	0.6422	0.160	0.061	3.214e-07	7.422e-03
3	Fibroblasts	*MMP23B*	0.6416	0.259	0.083	1.103e-72	2.547e-68
4	Fibroblasts	*RAMP1*	0.6413	0.661	0.310	4.289e-59	9.903e-55
1	Fibroblasts	*TMTC1*	0.6408	0.378	0.193	1.279e-66	2.953e-62
2	Fibroblasts	*PCDH9*	0.6399	0.195	0.080	4.947e-43	1.142e-38
1	Fibroblasts	*LOC138847232*	0.6395	0.529	0.282	8.507e-90	1.965e-85
4	Fibroblasts	*LY6E*	0.6393	0.518	0.220	5.350e-57	1.235e-52
0	Fibroblasts	*RBP4*	0.6380	0.641	0.425	6.415e-104	1.481e-99
3	Fibroblasts	*DOK6*	0.6371	0.254	0.134	9.791e-28	2.261e-23
3	Fibroblasts	*LRP6*	0.6354	0.467	0.318	3.592e-35	8.294e-31
3	Fibroblasts	*SMC5*	0.6306	0.637	0.513	9.095e-36	2.100e-31
3	Fibroblasts	*ZFP36L1*	0.6300	0.968	0.948	1.097e-73	2.533e-69
3	Fibroblasts	*OSR1*	0.6285	0.199	0.051	9.593e-73	2.215e-68
4	Fibroblasts	*BTG2*	0.6263	0.973	0.881	9.329e-29	2.154e-24
3	Fibroblasts	*ASXL3*	0.6239	0.238	0.079	3.709e-60	8.564e-56
12	Fibroblasts	*TTC3*	0.6203	0.819	0.759	2.465e-07	5.693e-03
4	Fibroblasts	*EPB41L2*	0.6202	0.589	0.311	1.002e-42	2.313e-38
2	Fibroblasts	*PLCL1*	0.6187	0.452	0.215	1.219e-79	2.814e-75
4	Fibroblasts	*HTRA3*	0.6185	0.623	0.335	3.309e-42	7.641e-38
2	Fibroblasts	*TIAM1*	0.6183	0.355	0.118	5.357e-118	1.237e-113
4	Fibroblasts	*RTN4*	0.6156	0.807	0.522	1.479e-48	3.416e-44
3	Fibroblasts	*SLC4A4*	0.6154	0.208	0.058	3.031e-68	6.998e-64

Original cluster	Annotated cell type	Gene	avg_log2FC	pct.1	pct.2	Raw P value	Bonferroni-adjusted P value
1	Fibroblasts	*ZBTB20*	0.6142	0.990	0.906	3.602e-148	8.319e-144
4	Fibroblasts	*LGALS3*	0.6124	0.286	0.067	6.992e-74	1.615e-69
0	Fibroblasts	*RPS12*	0.6113	1.000	0.988	0	0
3	Fibroblasts	*TGFBR3*	0.6103	0.683	0.553	6.041e-35	1.395e-30
3	Fibroblasts	*IMMP2L*	0.6100	0.587	0.458	1.138e-23	2.628e-19
12	Fibroblasts	*KEF51-p12*	0.6094	0.979	0.945	4.719e-16	1.090e-11
12	Fibroblasts	*EHBP1*	0.6093	0.694	0.581	1.747e-09	4.033e-05
12	Fibroblasts	*ZEB2*	0.6075	0.618	0.467	4.516e-08	1.043e-03
2	Fibroblasts	*INTS6*	0.6059	0.673	0.471	1.046e-60	2.416e-56
3	Fibroblasts	*PTGS2*	0.6054	0.247	0.145	6.620e-19	1.529e-14
2	Fibroblasts	*ARHGAP28*	0.6032	0.450	0.217	3.626e-81	8.374e-77
3	Fibroblasts	*FZD7*	0.6014	0.257	0.092	8.835e-60	2.040e-55
4	Fibroblasts	*LOC100359112*	0.5989	0.357	0.123	8.265e-53	1.909e-48
3	Fibroblasts	*FBLN5*	0.5983	0.355	0.201	5.892e-37	1.361e-32
0	Fibroblasts	*FTL*	0.5979	0.999	0.985	0	0
1	Fibroblasts	*DPYD*	0.5971	0.273	0.103	6.947e-78	1.604e-73
3	Fibroblasts	*RHOBTB1*	0.5967	0.321	0.207	1.204e-21	2.781e-17
3	Fibroblasts	*AR*	0.5963	0.370	0.233	2.815e-26	6.500e-22
4	Fibroblasts	*RRAS*	0.5951	0.650	0.379	3.698e-39	8.540e-35
0	Fibroblasts	*MDK*	0.5950	0.803	0.598	6.965e-103	1.608e-98
1	Fibroblasts	*KLF12*	0.5926	0.870	0.643	3.289e-95	7.595e-91
3	Fibroblasts	*CCN2*	0.5923	0.146	0.063	1.312e-23	3.031e-19
3	Fibroblasts	*COL8A1*	0.5919	0.136	0.039	2.545e-42	5.877e-38
0	Fibroblasts	*STMN1*	0.5915	0.742	0.565	8.331e-93	1.924e-88
0	Fibroblasts	*RPL5*	0.5913	1.000	0.972	0	0
3	Fibroblasts	*ITGA1*	0.5903	0.329	0.150	2.239e-49	5.170e-45
2	Fibroblasts	*LOC127493124*	0.5864	0.333	0.135	6.697e-79	1.546e-74
3	Fibroblasts	*CLEC3B*	0.5859	0.539	0.224	5.355e-111	1.237e-106
1	Fibroblasts	*MOXD1*	0.5856	0.858	0.585	2.404e-94	5.550e-90
4	Fibroblasts	*BCHE*	0.5856	0.320	0.114	4.105e-45	9.478e-41
0	Fibroblasts	*RBP1*	0.5851	0.627	0.426	5.657e-96	1.306e-91
1	Fibroblasts	*SLC35F1*	0.5837	0.621	0.376	4.545e-81	1.050e-76
4	Fibroblasts	*AHNAK*	0.5822	0.827	0.619	5.032e-34	1.162e-29
3	Fibroblasts	*CP*	0.5819	0.116	0.013	5.912e-92	1.365e-87
3	Fibroblasts	*MEST*	0.5812	0.184	0.072	3.514e-35	8.114e-31
0	Fibroblasts	*AMER2*	0.5803	0.342	0.167	1.811e-82	4.182e-78
12	Fibroblasts	*VPS13B*	0.5767	0.660	0.581	5.167e-07	1.193e-02
1	Fibroblasts	*CADM2*	0.5741	0.436	0.130	3.731e-194	8.615e-190
4	Fibroblasts	*PRRX1*	0.5734	0.809	0.522	1.455e-42	3.361e-38
2	Fibroblasts	*CKAP2*	0.5725	0.322	0.193	4.696e-34	1.084e-29

Original cluster	Annotated cell type	Gene	avg_log2FC	pct.1	pct.2	Raw P value	Bonferroni-adjusted P value
0	Fibroblasts	*RPS5*	0.5725	1.000	0.986	0	0
4	Fibroblasts	*CCDC85A*	0.5722	0.232	0.061	3.100e-52	7.158e-48
4	Fibroblasts	*LOC127488699*	0.5720	0.325	0.130	9.017e-39	2.082e-34
4	Fibroblasts	*DDIT4*	0.5716	0.714	0.531	4.946e-21	1.142e-16
3	Fibroblasts	*LPL*	0.5711	0.225	0.098	7.972e-34	1.841e-29
2	Fibroblasts	*GULP1*	0.5711	0.642	0.413	3.427e-67	7.914e-63
4	Fibroblasts	*GSTP1*	0.5706	0.805	0.564	6.208e-36	1.434e-31
3	Fibroblasts	*PODN*	0.5706	0.232	0.089	4.854e-48	1.121e-43
2	Fibroblasts	*IL1RAPL1*	0.5688	0.273	0.135	6.131e-43	1.416e-38
2	Fibroblasts	*ZC3H12B*	0.5671	0.512	0.326	1.243e-46	2.869e-42
4	Fibroblasts	*DNAJB1*	0.5667	0.582	0.443	3.229e-16	7.457e-12
12	Fibroblasts	*KEF51-p04*	0.5664	1.000	0.990	4.774e-22	1.102e-17
0	Fibroblasts	*NACA*	0.5664	1.000	0.974	0	0
2	Fibroblasts	*ROR1*	0.5660	0.550	0.296	2.392e-82	5.524e-78
2	Fibroblasts	*TDRD15*	0.5651	0.191	0.033	5.500e-126	1.270e-121
4	Fibroblasts	*BSG*	0.5644	0.891	0.699	1.164e-36	2.689e-32
3	Fibroblasts	*ECRG4*	0.5640	0.151	0.047	7.025e-41	1.622e-36
2	Fibroblasts	*BMP3*	0.5638	0.333	0.111	1.122e-105	2.591e-101
3	Fibroblasts	*ADAMTS5*	0.5632	0.213	0.071	2.880e-54	6.651e-50
0	Fibroblasts	*FTH1*	0.5630	1.000	0.992	2.019e-274	4.663e-270
0	Fibroblasts	*PDGFRA*	0.5629	0.624	0.422	1.948e-86	4.498e-82
2	Fibroblasts	*JAZF1*	0.5623	0.548	0.303	4.108e-75	9.486e-71
2	Fibroblasts	*CPA6*	0.5617	0.207	0.066	2.415e-69	5.577e-65
12	Fibroblasts	*EGFLAM*	0.5589	0.236	0.120	1.245e-06	2.874e-02
1	Fibroblasts	*MEIS2*	0.5589	0.900	0.690	7.313e-101	1.689e-96
1	Fibroblasts	*HECW1*	0.5586	0.362	0.150	1.462e-89	3.375e-85
3	Fibroblasts	*SLC16A9*	0.5578	0.276	0.165	5.768e-22	1.332e-17
4	Fibroblasts	*ANGPTL1*	0.5575	0.288	0.072	1.193e-67	2.754e-63
3	Fibroblasts	*DEPTOR*	0.5554	0.228	0.089	1.905e-45	4.400e-41
3	Fibroblasts	*LDAF1*	0.5541	0.271	0.129	1.201e-38	2.774e-34
0	Fibroblasts	*RPL8*	0.5538	1.000	0.981	0	0
0	Fibroblasts	*LOC138849180*	0.5511	0.999	0.975	0	0
0	Fibroblasts	*LOC127485429*	0.5491	0.699	0.549	2.788e-77	6.438e-73
4	Fibroblasts	*ANTXR1*	0.5484	0.573	0.394	1.069e-13	2.469e-09
4	Fibroblasts	*ID3*	0.5482	0.920	0.803	6.541e-22	1.510e-17
0	Fibroblasts	*RPL10A*	0.5472	0.999	0.966	1.133e-265	2.616e-261
3	Fibroblasts	*PBX1*	0.5438	0.892	0.805	1.027e-44	2.373e-40
2	Fibroblasts	*KIF26B*	0.5431	0.382	0.179	1.763e-68	4.071e-64
3	Fibroblasts	*UST*	0.5421	0.285	0.162	7.367e-26	1.701e-21
2	Fibroblasts	*ABCC4*	0.5418	0.408	0.188	1.248e-78	2.881e-74

Original cluster	Annotated cell type	Gene	avg_log2FC	pct.1	pct.2	Raw P value	Bonferroni-adjusted P value
0	Fibroblasts	*RPSA*	0.5418	1.000	0.970	0	0
2	Fibroblasts	*LHFPL3*	0.5414	0.202	0.073	2.985e-55	6.893e-51
3	Fibroblasts	*PHACTR1*	0.5400	0.194	0.084	7.414e-32	1.712e-27
3	Fibroblasts	*FBN1*	0.5393	0.386	0.274	8.831e-21	2.039e-16
0	Fibroblasts	*RPL11*	0.5377	1.000	0.986	0	0
3	Fibroblasts	*FHL1*	0.5371	0.285	0.134	2.382e-39	5.501e-35
0	Fibroblasts	*RPS4X*	0.5368	1.000	0.987	0	0
4	Fibroblasts	*ANXA1*	0.5353	0.739	0.462	2.916e-44	6.733e-40
3	Fibroblasts	*PDE1A*	0.5346	0.496	0.403	1.331e-14	3.074e-10
3	Fibroblasts	*PTCHD1*	0.5345	0.187	0.041	1.319e-81	3.045e-77
4	Fibroblasts	*S100A11*	0.5343	0.945	0.740	4.978e-55	1.149e-50
2	Fibroblasts	*ENPP2*	0.5340	0.478	0.232	3.664e-80	8.461e-76
2	Fibroblasts	*WSB1*	0.5340	0.670	0.482	2.303e-45	5.318e-41
4	Fibroblasts	*LOC127493068*	0.5335	0.457	0.349	2.097e-09	4.843e-05
1	Fibroblasts	*COL4A6*	0.5333	0.372	0.177	2.105e-71	4.860e-67
3	Fibroblasts	*SSBP2*	0.5324	0.547	0.441	6.561e-24	1.515e-19
4	Fibroblasts	*VEGFB*	0.5319	0.730	0.475	5.125e-35	1.183e-30
4	Fibroblasts	*KLF4*	0.5314	0.918	0.764	1.293e-28	2.986e-24
1	Fibroblasts	*IKZF2*	0.5308	0.354	0.159	4.198e-76	9.694e-72
0	Fibroblasts	*RPS27A*	0.5301	1.000	0.984	0	0
0	Fibroblasts	*RPS20*	0.5298	1.000	0.984	0	0
4	Fibroblasts	*PDGFRL*	0.5286	0.302	0.075	1.721e-71	3.974e-67
3	Fibroblasts	*LRRC17*	0.5284	0.365	0.202	2.021e-36	4.667e-32
0	Fibroblasts	*RPL9*	0.5277	1.000	0.987	0	0
4	Fibroblasts	*TIMP3*	0.5273	0.927	0.746	1.940e-38	4.480e-34
0	Fibroblasts	*OSR2*	0.5272	0.712	0.522	1.461e-76	3.373e-72
4	Fibroblasts	*CHRNE*	0.5271	0.571	0.298	6.597e-37	1.523e-32
2	Fibroblasts	*SYNPO2*	0.5271	0.583	0.344	7.185e-66	1.659e-61
0	Fibroblasts	*RPL35A*	0.5262	1.000	0.982	0	0
1	Fibroblasts	*VEGFA*	0.5260	0.623	0.417	7.516e-61	1.736e-56
2	Fibroblasts	*DYNC2H1*	0.5245	0.704	0.480	2.086e-66	4.816e-62
2	Fibroblasts	*RFX2*	0.5245	0.296	0.153	2.606e-41	6.018e-37
0	Fibroblasts	*RPL7*	0.5243	1.000	0.973	6.118e-303	1.413e-298
4	Fibroblasts	*EMP3*	0.5238	0.734	0.451	4.531e-37	1.046e-32
0	Fibroblasts	*RPL37*	0.5236	1.000	0.981	0	0
3	Fibroblasts	*GLUL*	0.5233	0.521	0.408	1.348e-23	3.112e-19
0	Fibroblasts	*DUSP26*	0.5228	0.339	0.191	4.161e-61	9.609e-57
2	Fibroblasts	*RYR2*	0.5226	0.459	0.255	9.423e-55	2.176e-50
3	Fibroblasts	*CDKN1C*	0.5216	0.507	0.367	3.150e-27	7.273e-23
4	Fibroblasts	*PRDX6*	0.5203	0.786	0.547	4.687e-35	1.082e-30

Original cluster	Annotated cell type	Gene	avg_log2FC	pct.1	pct.2	Raw P value	Bonferroni-adjusted P value
0	Fibroblasts	*APCDD1*	0.5192	0.513	0.346	4.473e-63	1.033e-58
2	Fibroblasts	*LOC103349704*	0.5187	0.780	0.516	3.048e-88	7.039e-84
2	Fibroblasts	*FLRT3*	0.5187	0.446	0.226	3.182e-66	7.349e-62
4	Fibroblasts	*CSRP2*	0.5182	0.439	0.237	4.352e-28	1.005e-23
0	Fibroblasts	*RPS15A*	0.5176	1.000	0.983	0	0
0	Fibroblasts	*RPS3A*	0.5157	0.999	0.983	0	0
3	Fibroblasts	*ADD3*	0.5148	0.555	0.446	5.150e-23	1.189e-18
4	Fibroblasts	*FGF10*	0.5148	0.386	0.171	2.803e-34	6.473e-30
0	Fibroblasts	*DBP*	0.5137	0.670	0.554	2.908e-62	6.714e-58
4	Fibroblasts	*ABCA6*	0.5134	0.207	0.038	1.046e-70	2.416e-66
0	Fibroblasts	*CARD6*	0.5132	0.980	0.922	2.143e-158	4.949e-154
4	Fibroblasts	*PCBP1*	0.5122	0.855	0.635	1.361e-33	3.144e-29
3	Fibroblasts	*PARD3B*	0.5122	0.661	0.565	1.885e-22	4.353e-18
0	Fibroblasts	*RPS25*	0.5121	0.999	0.974	8.659e-293	2.000e-288
4	Fibroblasts	*IGSF10*	0.5107	0.418	0.170	2.493e-44	5.756e-40
3	Fibroblasts	*THBS2*	0.5098	0.160	0.038	2.790e-64	6.442e-60
0	Fibroblasts	*EIF3F*	0.5093	0.985	0.922	1.401e-172	3.235e-168
2	Fibroblasts	*PRKCQ*	0.5086	0.259	0.079	2.151e-91	4.968e-87
1	Fibroblasts	*NALF1*	0.5085	0.166	0.071	9.094e-37	2.100e-32
3	Fibroblasts	*DLG2*	0.5085	0.450	0.334	8.575e-18	1.980e-13
3	Fibroblasts	*ARID5B*	0.5073	0.610	0.461	3.011e-31	6.953e-27
1	Fibroblasts	*LRMDA*	0.5070	0.628	0.391	8.453e-70	1.952e-65
3	Fibroblasts	*THRB*	0.5070	0.378	0.276	5.010e-17	1.157e-12
4	Fibroblasts	*ADM*	0.5064	0.539	0.314	2.140e-25	4.942e-21
0	Fibroblasts	*RPL22*	0.5055	0.996	0.961	5.038e-204	1.163e-199
3	Fibroblasts	*FMNL2*	0.5051	0.314	0.187	5.343e-26	1.234e-21
0	Fibroblasts	*RPS6*	0.5050	0.999	0.963	7.567e-226	1.747e-221
3	Fibroblasts	*ASPH*	0.5043	0.589	0.471	1.371e-26	3.165e-22
3	Fibroblasts	*PEG3*	0.5035	0.273	0.155	1.615e-24	3.728e-20
3	Fibroblasts	*NFIB*	0.5028	0.480	0.353	4.560e-24	1.053e-19
2	Fibroblasts	*PRDM5*	0.5026	0.406	0.194	1.240e-69	2.862e-65
3	Fibroblasts	*RHOB*	0.5020	0.828	0.693	3.796e-44	8.765e-40
2	Fibroblasts	*RASD1*	0.5014	0.396	0.246	2.642e-33	6.101e-29
1	Fibroblasts	*CEP126*	0.5014	0.292	0.129	3.099e-62	7.156e-58
12	Fibroblasts	*KEF51-p07*	0.5008	1.000	0.994	5.814e-24	1.343e-19
3	Fibroblasts	*GSTM3*	0.4993	0.462	0.351	2.761e-23	6.375e-19
3	Fibroblasts	*LOC127484919*	0.4992	0.121	0.036	6.071e-36	1.402e-31
2	Fibroblasts	*GLIS3*	0.4972	0.545	0.318	1.115e-60	2.574e-56
3	Fibroblasts	*PDZRN3*	0.4972	0.582	0.468	1.498e-20	3.460e-16
0	Fibroblasts	*LOC127489420*	0.4962	0.967	0.888	3.805e-137	8.787e-133

Original cluster	Annotated cell type	Gene	avg_log2FC	pct.1	pct.2	Raw P value	Bonferroni-adjusted P value
0	Fibroblasts	*RPL19*	0.4954	1.000	0.980	1.949e-298	4.500e-294
1	Fibroblasts	*STK33*	0.4946	0.389	0.207	2.822e-58	6.516e-54
1	Fibroblasts	*LOC138850492*	0.4934	0.343	0.191	1.570e-44	3.626e-40
0	Fibroblasts	*RPS7*	0.4933	1.000	0.984	0	0
4	Fibroblasts	*CYBRD1*	0.4929	0.516	0.268	2.767e-34	6.389e-30
3	Fibroblasts	*TMEM158*	0.4927	0.188	0.090	1.602e-25	3.700e-21
2	Fibroblasts	*MMP16*	0.4927	0.283	0.149	1.008e-38	2.328e-34
2	Fibroblasts	*HOMER1*	0.4901	0.379	0.215	1.185e-42	2.736e-38
1	Fibroblasts	*LOC138849567*	0.4892	0.326	0.180	1.538e-40	3.552e-36
0	Fibroblasts	*RPL27A*	0.4889	1.000	0.983	0	0
2	Fibroblasts	*VEGFC*	0.4888	0.319	0.149	7.377e-58	1.703e-53
0	Fibroblasts	*GABARAP*	0.4876	0.996	0.970	2.115e-190	4.883e-186
4	Fibroblasts	*LAMC1*	0.4871	0.505	0.290	1.836e-26	4.241e-22
4	Fibroblasts	*SPON2*	0.4869	0.414	0.244	3.846e-18	8.880e-14
0	Fibroblasts	*LOC138843493*	0.4866	0.915	0.832	4.252e-114	9.819e-110
3	Fibroblasts	*CAP2*	0.4856	0.231	0.119	5.926e-26	1.368e-21
4	Fibroblasts	*ALDH2*	0.4843	0.639	0.391	1.888e-28	4.359e-24
0	Fibroblasts	*FIBIN*	0.4838	0.499	0.358	5.402e-47	1.247e-42
0	Fibroblasts	*RPL23*	0.4830	1.000	0.988	0	0
0	Fibroblasts	*RSL24D1*	0.4827	0.900	0.796	4.378e-98	1.011e-93
0	Fibroblasts	*RPL14*	0.4823	1.000	0.982	0	0
1	Fibroblasts	*CLU*	0.4823	0.411	0.216	7.791e-64	1.799e-59
0	Fibroblasts	*RPS26*	0.4813	1.000	0.980	4.054e-298	9.362e-294
3	Fibroblasts	*EDA*	0.4801	0.287	0.182	3.668e-17	8.469e-13
0	Fibroblasts	*RACK1*	0.4799	0.999	0.973	5.319e-229	1.228e-224
2	Fibroblasts	*TNFRSF19*	0.4797	0.425	0.203	3.706e-71	8.558e-67
2	Fibroblasts	*PAPPA*	0.4796	0.449	0.250	5.629e-54	1.300e-49
0	Fibroblasts	*LOC138847495*	0.4782	0.911	0.824	2.355e-106	5.437e-102
2	Fibroblasts	*LDAH*	0.4781	0.415	0.225	5.721e-54	1.321e-49
2	Fibroblasts	*RFX3*	0.4775	0.659	0.467	9.227e-47	2.131e-42
3	Fibroblasts	*IGFBP4*	0.4771	0.815	0.680	4.412e-35	1.019e-30
3	Fibroblasts	*ADRA1A*	0.4769	0.149	0.051	1.141e-35	2.634e-31
4	Fibroblasts	*ANGPTL4*	0.4768	0.359	0.208	1.735e-16	4.007e-12
3	Fibroblasts	*EXT1*	0.4765	0.401	0.320	2.270e-13	5.241e-09
3	Fibroblasts	*PDE7B*	0.4756	0.185	0.053	4.423e-56	1.021e-51
4	Fibroblasts	*TLE2*	0.4753	0.250	0.065	4.462e-56	1.030e-51
2	Fibroblasts	*TOX*	0.4751	0.216	0.053	1.057e-98	2.440e-94
4	Fibroblasts	*EYA1*	0.4749	0.188	0.035	8.010e-64	1.850e-59
3	Fibroblasts	*FGF7*	0.4744	0.138	0.036	1.318e-48	3.044e-44
2	Fibroblasts	*LCORL*	0.4743	0.538	0.365	1.847e-39	4.264e-35

Original cluster	Annotated cell type	Gene	avg_log2FC	pct.1	pct.2	Raw P value	Bonferroni-adjusted P value
4	Fibroblasts	*POSTN*	0.4737	0.288	0.150	6.091e-17	1.407e-12
3	Fibroblasts	*PIP5K1B*	0.4724	0.220	0.102	8.426e-29	1.946e-24
2	Fibroblasts	*ARHGAP42*	0.4723	0.494	0.274	1.855e-61	4.284e-57
0	Fibroblasts	*RPL6*	0.4713	1.000	0.974	8.580e-231	1.981e-226
2	Fibroblasts	*ENOX1*	0.4708	0.226	0.083	2.230e-59	5.148e-55
0	Fibroblasts	*RPL27*	0.4708	0.996	0.954	2.407e-168	5.558e-164
0	Fibroblasts	*ENHO*	0.4706	0.410	0.263	2.891e-56	6.675e-52
0	Fibroblasts	*RPS24*	0.4706	1.000	0.986	0	0
0	Fibroblasts	*PEBP1*	0.4700	0.745	0.631	2.274e-75	5.251e-71
1	Fibroblasts	*LOC100356485*	0.4694	0.143	0.042	3.503e-57	8.089e-53
1	Fibroblasts	*COL4A5*	0.4688	0.374	0.200	5.251e-56	1.213e-51
4	Fibroblasts	*AFF2*	0.4672	0.296	0.108	1.335e-38	3.083e-34
3	Fibroblasts	*PLEKHH2*	0.4666	0.172	0.066	1.648e-33	3.807e-29
0	Fibroblasts	*RPL24*	0.4656	1.000	0.985	0	0
1	Fibroblasts	*RGS7*	0.4653	0.186	0.064	1.863e-58	4.302e-54
0	Fibroblasts	*RPL30*	0.4653	0.999	0.975	3.678e-250	8.493e-246
3	Fibroblasts	*SLC40A1*	0.4645	0.492	0.392	2.549e-17	5.885e-13
0	Fibroblasts	*H3-3B*	0.4643	0.994	0.970	1.801e-129	4.158e-125
3	Fibroblasts	*COL6A3*	0.4635	0.239	0.114	5.360e-31	1.238e-26
2	Fibroblasts	*P2RY1*	0.4632	0.203	0.061	6.146e-74	1.419e-69
0	Fibroblasts	*LOC100349792*	0.4630	0.384	0.284	9.821e-29	2.268e-24
4	Fibroblasts	*SPARC*	0.4623	0.714	0.505	1.301e-22	3.003e-18
3	Fibroblasts	*LOC100340483*	0.4616	0.728	0.667	5.771e-21	1.333e-16
3	Fibroblasts	*FLRT2*	0.4602	0.282	0.176	5.466e-19	1.262e-14
3	Fibroblasts	*CILP*	0.4595	0.141	0.018	1.066e-99	2.462e-95
0	Fibroblasts	*LOC103352391*	0.4594	0.949	0.870	3.286e-100	7.589e-96
0	Fibroblasts	*LAPTM4B*	0.4591	0.863	0.748	3.438e-83	7.940e-79
3	Fibroblasts	*IGDCC4*	0.4591	0.296	0.179	1.718e-22	3.967e-18
0	Fibroblasts	*SSR2*	0.4583	0.944	0.864	4.471e-108	1.033e-103
2	Fibroblasts	*LOC138845366*	0.4578	0.317	0.149	9.782e-54	2.259e-49
2	Fibroblasts	*PTCH1*	0.4573	0.276	0.132	3.246e-46	7.496e-42
2	Fibroblasts	*COL6A6*	0.4567	0.164	0.026	2.707e-116	6.250e-112
2	Fibroblasts	*FBN2*	0.4558	0.240	0.093	1.165e-59	2.691e-55
3	Fibroblasts	*RTL3*	0.4557	0.121	0.010	6.290e-114	1.452e-109
4	Fibroblasts	*LOC138848072*	0.4551	0.286	0.115	8.543e-32	1.973e-27
4	Fibroblasts	*ZFP36*	0.4548	0.825	0.739	5.901e-14	1.363e-09
3	Fibroblasts	*ZFP36L2*	0.4543	0.692	0.630	1.745e-18	4.029e-14
3	Fibroblasts	*FXYD1*	0.4542	0.845	0.741	2.614e-41	6.035e-37
0	Fibroblasts	*RPL29*	0.4541	0.933	0.865	6.562e-103	1.515e-98
0	Fibroblasts	*SSBP4*	0.4541	0.485	0.371	2.191e-42	5.059e-38

Original cluster	Annotated cell type	Gene	avg_log2FC	pct.1	pct.2	Raw P value	Bonferroni-adjusted P value
0	Fibroblasts	*MAGED2*	0.4537	0.686	0.588	2.797e-53	6.459e-49
3	Fibroblasts	*PTBP3*	0.4532	0.289	0.208	1.649e-12	3.809e-08
3	Fibroblasts	*PDE5A*	0.4523	0.358	0.298	1.398e-09	3.229e-05
4	Fibroblasts	*KIF22*	0.4521	0.580	0.344	1.604e-26	3.705e-22
0	Fibroblasts	*LOC103350713*	0.4518	0.936	0.838	2.331e-93	5.382e-89
4	Fibroblasts	*LOC100008606*	0.4504	0.364	0.168	4.671e-30	1.079e-25
2	Fibroblasts	*LOC127483154*	0.4498	0.595	0.403	5.805e-41	1.341e-36
1	Fibroblasts	*RALGPS2*	0.4490	0.543	0.351	1.252e-51	2.890e-47
3	Fibroblasts	*ADAMTS1*	0.4484	0.422	0.294	6.634e-21	1.532e-16
0	Fibroblasts	*RPS27*	0.4482	1.000	0.982	1.259e-249	2.907e-245
4	Fibroblasts	*RHOU*	0.4478	0.312	0.144	5.790e-26	1.337e-21
4	Fibroblasts	*EFNA5*	0.4477	0.552	0.328	1.872e-24	4.322e-20
4	Fibroblasts	*MMP2*	0.4473	0.445	0.257	7.772e-22	1.795e-17
0	Fibroblasts	*HAND2*	0.4465	0.315	0.186	1.315e-46	3.035e-42
3	Fibroblasts	*ZNF521*	0.4456	0.356	0.225	8.341e-23	1.926e-18
0	Fibroblasts	*RPL38*	0.4450	0.999	0.975	1.737e-197	4.012e-193
2	Fibroblasts	*CHRM2*	0.4450	0.251	0.120	4.004e-40	9.247e-36
2	Fibroblasts	*CAMK2D*	0.4446	0.782	0.591	3.423e-56	7.905e-52
2	Fibroblasts	*TCF4*	0.4441	0.916	0.737	3.403e-83	7.857e-79
1	Fibroblasts	*ACSS3*	0.4440	0.250	0.101	3.553e-61	8.205e-57
2	Fibroblasts	*OSBPL8*	0.4439	0.652	0.466	3.042e-48	7.024e-44
0	Fibroblasts	*EEF1A1*	0.4420	1.000	0.987	4.193e-301	9.681e-297
0	Fibroblasts	*LOC138849940*	0.4419	0.978	0.919	1.290e-127	2.979e-123
0	Fibroblasts	*RPS8*	0.4416	0.999	0.984	3.668e-285	8.470e-281
4	Fibroblasts	*AEBP1*	0.4410	0.364	0.173	7.931e-28	1.831e-23
2	Fibroblasts	*NFKBIZ*	0.4407	0.546	0.429	5.944e-17	1.373e-12
0	Fibroblasts	*NDP*	0.4396	0.264	0.148	2.539e-43	5.863e-39
1	Fibroblasts	*CPQ*	0.4392	0.566	0.416	1.717e-34	3.965e-30
3	Fibroblasts	*CCDC181*	0.4391	0.207	0.106	7.857e-23	1.814e-18
2	Fibroblasts	*SFPQ*	0.4388	0.834	0.634	7.927e-61	1.830e-56
4	Fibroblasts	*RBMS1*	0.4386	0.766	0.530	7.064e-26	1.631e-21
2	Fibroblasts	*MAOB*	0.4378	0.424	0.228	1.465e-53	3.382e-49
0	Fibroblasts	*RPL3*	0.4376	0.997	0.963	7.481e-166	1.728e-161
4	Fibroblasts	*ZCCHC24*	0.4363	0.523	0.304	8.191e-25	1.891e-20
4	Fibroblasts	*LOC127488889*	0.4362	0.398	0.280	9.997e-10	2.309e-05
12	Fibroblasts	*KEF51-p10*	0.4357	1.000	0.994	3.330e-19	7.689e-15
0	Fibroblasts	*LOC138846222*	0.4343	0.829	0.730	3.606e-71	8.327e-67
2	Fibroblasts	*KSR1*	0.4342	0.226	0.109	4.717e-37	1.089e-32
3	Fibroblasts	*CD83*	0.4339	0.120	0.051	9.532e-19	2.201e-14
0	Fibroblasts	*RPL21*	0.4338	0.999	0.974	1.192e-214	2.752e-210

Original cluster	Annotated cell type	Gene	avg_log2FC	pct.1	pct.2	Raw P value	Bonferroni-adjusted P value
0	Fibroblasts	*ATP1A2*	0.4329	0.442	0.297	3.215e-51	7.423e-47
3	Fibroblasts	*ICAM1*	0.4328	0.173	0.069	1.164e-31	2.687e-27
0	Fibroblasts	*LOC127487801*	0.4324	0.717	0.608	9.026e-56	2.084e-51
4	Fibroblasts	*EIF1*	0.4323	0.996	0.971	1.887e-49	4.358e-45
0	Fibroblasts	*RPL32*	0.4315	0.995	0.963	2.673e-170	6.173e-166
4	Fibroblasts	*LOC100343557*	0.4306	0.425	0.267	4.856e-14	1.121e-09
2	Fibroblasts	*TTC28*	0.4301	0.752	0.552	3.738e-49	8.632e-45
4	Fibroblasts	*SDC2*	0.4298	0.857	0.644	1.930e-25	4.458e-21
3	Fibroblasts	*ECM2*	0.4296	0.155	0.045	5.951e-47	1.374e-42
2	Fibroblasts	*IL15*	0.4296	0.261	0.109	1.571e-56	3.628e-52
2	Fibroblasts	*TCF12*	0.4293	0.862	0.703	1.039e-58	2.400e-54
2	Fibroblasts	*WASF3*	0.4290	0.307	0.142	2.498e-54	5.769e-50
3	Fibroblasts	*TCF21*	0.4289	0.283	0.163	1.255e-23	2.898e-19
2	Fibroblasts	*TRPS1*	0.4285	0.470	0.277	5.935e-50	1.371e-45
2	Fibroblasts	*PTPN13*	0.4283	0.490	0.277	2.428e-56	5.606e-52
4	Fibroblasts	*JDP2*	0.4283	0.391	0.180	1.004e-31	2.318e-27
3	Fibroblasts	*KALRN*	0.4280	0.221	0.143	1.378e-12	3.183e-08
2	Fibroblasts	*KIF16B*	0.4279	0.334	0.157	5.640e-59	1.302e-54
4	Fibroblasts	*TMSB10*	0.4263	0.998	0.958	1.085e-74	2.504e-70
4	Fibroblasts	*DPYSL2*	0.4256	0.536	0.326	8.845e-22	2.042e-17
2	Fibroblasts	*PRTG*	0.4231	0.309	0.134	3.059e-61	7.063e-57
3	Fibroblasts	*PRSS12*	0.4228	0.153	0.051	3.359e-38	7.757e-34
1	Fibroblasts	*SDK1*	0.4227	0.346	0.202	3.145e-39	7.263e-35
0	Fibroblasts	*UBB*	0.4227	0.999	0.966	2.619e-100	6.048e-96
1	Fibroblasts	*ADAMTS12*	0.4227	0.438	0.284	7.801e-36	1.801e-31
2	Fibroblasts	*PI15*	0.4225	0.354	0.199	1.504e-37	3.473e-33
0	Fibroblasts	*EIF3K*	0.4218	0.916	0.857	5.334e-84	1.232e-79
2	Fibroblasts	*STAG1*	0.4213	0.800	0.608	3.370e-52	7.782e-48
3	Fibroblasts	*TSPAN15*	0.4212	0.310	0.201	5.756e-19	1.329e-14
3	Fibroblasts	*DOCK9*	0.4203	0.316	0.191	7.396e-23	1.708e-18
0	Fibroblasts	*FAU*	0.4197	0.999	0.987	4.697e-271	1.085e-266
4	Fibroblasts	*LGALS1*	0.4194	0.764	0.520	6.969e-25	1.609e-20
2	Fibroblasts	*BCKDHB*	0.4191	0.418	0.269	4.303e-32	9.935e-28
3	Fibroblasts	*SYNE1*	0.4191	0.537	0.438	7.860e-18	1.815e-13
2	Fibroblasts	*BTBD3*	0.4177	0.466	0.304	1.669e-34	3.855e-30
2	Fibroblasts	*FRZB*	0.4176	0.257	0.106	4.327e-55	9.991e-51
4	Fibroblasts	*CCND2*	0.4171	0.662	0.473	4.843e-18	1.118e-13
3	Fibroblasts	*PMP22*	0.4171	0.552	0.454	3.900e-21	9.005e-17
4	Fibroblasts	*LOC127482730*	0.4169	0.486	0.324	7.715e-16	1.782e-11
0	Fibroblasts	*RPL12*	0.4166	1.000	0.984	7.159e-222	1.653e-217

Original cluster	Annotated cell type	Gene	avg_log2FC	pct.1	pct.2	Raw P value	Bonferroni-adjusted P value
0	Fibroblasts	*RPS2*	0.4166	1.000	0.982	2.133e-227	4.926e-223
3	Fibroblasts	*COL6A2*	0.4165	0.696	0.582	3.636e-24	8.397e-20
2	Fibroblasts	*SH3RF1*	0.4158	0.331	0.169	6.097e-47	1.408e-42
2	Fibroblasts	*CNTLN*	0.4139	0.578	0.397	2.175e-41	5.023e-37
0	Fibroblasts	*RPS15*	0.4138	1.000	0.986	1.317e-235	3.041e-231
0	Fibroblasts	*RPL37A*	0.4136	1.000	0.978	5.670e-216	1.309e-211
0	Fibroblasts	*RPS14*	0.4130	1.000	0.986	9.431e-300	2.178e-295
2	Fibroblasts	*SPRY2*	0.4126	0.372	0.244	4.039e-24	9.328e-20
4	Fibroblasts	*NUPR1*	0.4115	0.596	0.373	1.569e-22	3.623e-18
0	Fibroblasts	*RPLP1*	0.4115	1.000	0.988	1.500e-239	3.463e-235
0	Fibroblasts	*RPL34*	0.4115	0.940	0.869	8.088e-95	1.868e-90
0	Fibroblasts	*RPS29*	0.4114	1.000	0.970	2.546e-173	5.879e-169
0	Fibroblasts	*RCN3*	0.4113	0.591	0.454	5.938e-50	1.371e-45
4	Fibroblasts	*MTCH1*	0.4113	0.789	0.590	6.777e-23	1.565e-18
3	Fibroblasts	*VCAN*	0.4110	0.246	0.134	6.723e-24	1.553e-19
0	Fibroblasts	*RPS9*	0.4109	1.000	0.992	0	0
4	Fibroblasts	*IER2*	0.4101	0.927	0.810	4.324e-19	9.986e-15
1	Fibroblasts	*TUFT1*	0.4100	0.203	0.097	1.696e-36	3.917e-32
3	Fibroblasts	*HERC3*	0.4079	0.268	0.167	4.378e-18	1.011e-13
0	Fibroblasts	*RPLP0*	0.4074	1.000	0.977	2.821e-194	6.513e-190
0	Fibroblasts	*RPL10*	0.4073	1.000	0.976	2.099e-196	4.847e-192
2	Fibroblasts	*ANK2*	0.4071	0.508	0.303	9.362e-52	2.162e-47
0	Fibroblasts	*BTF3*	0.4068	0.989	0.946	2.180e-123	5.034e-119
2	Fibroblasts	*CHD9*	0.4065	0.713	0.544	1.096e-34	2.531e-30
4	Fibroblasts	*SELENOP*	0.4058	0.936	0.797	7.997e-31	1.847e-26
3	Fibroblasts	*CD99*	0.4054	0.891	0.854	3.229e-33	7.457e-29
3	Fibroblasts	*CCDC80*	0.4048	0.210	0.138	1.941e-11	4.482e-07
3	Fibroblasts	*CENPW*	0.4047	0.157	0.059	5.990e-33	1.383e-28
1	Fibroblasts	*HSP90AA1*	0.4047	0.840	0.708	7.075e-44	1.634e-39
0	Fibroblasts	*RPS28*	0.4037	1.000	0.981	7.722e-224	1.783e-219
0	Fibroblasts	*RPL7A*	0.4032	1.000	0.985	4.378e-250	1.011e-245
0	Fibroblasts	*TLE5*	0.4032	0.741	0.641	1.456e-50	3.362e-46
3	Fibroblasts	*DISP1*	0.4029	0.248	0.163	2.353e-13	5.433e-09
1	Fibroblasts	*SAMD12*	0.4023	0.188	0.084	3.442e-38	7.948e-34
4	Fibroblasts	*IRX3*	0.4019	0.143	0.011	2.760e-110	6.374e-106
1	Fibroblasts	*NMNAT1*	0.4015	0.238	0.148	9.359e-20	2.161e-15
2	Fibroblasts	*FBXL7*	0.4008	0.624	0.454	3.490e-36	8.059e-32
1	Fibroblasts	*LRRC4C*	0.4007	0.173	0.094	5.678e-22	1.311e-17
2	Fibroblasts	*SUPT3H*	0.4003	0.539	0.378	2.452e-32	5.662e-28
1	Fibroblasts	*FAT4*	0.4000	0.363	0.223	6.937e-34	1.602e-29

Original cluster	Annotated cell type	Gene	avg_log2FC	pct.1	pct.2	Raw P value	Bonferroni-adjusted P value
4	Fibroblasts	*IRX5*	0.3997	0.136	0.003	4.197e-179	9.692e-175
2	Fibroblasts	*GPHN*	0.3995	0.675	0.459	1.770e-53	4.088e-49
4	Fibroblasts	*ARRDC3*	0.3989	0.570	0.422	1.764e-11	4.073e-07
2	Fibroblasts	*REV3L*	0.3989	0.689	0.495	2.676e-43	6.180e-39
3	Fibroblasts	*PALLD*	0.3980	0.453	0.364	1.116e-14	2.578e-10
12	Fibroblasts	*KEF51-p11*	0.3979	1.000	0.993	1.377e-13	3.180e-09
0	Fibroblasts	*LOC108175433*	0.3973	0.999	0.968	5.570e-123	1.286e-118
2	Fibroblasts	*SERPINB9*	0.3971	0.347	0.206	5.129e-32	1.184e-27
1	Fibroblasts	*CADM1*	0.3970	0.339	0.183	2.568e-47	5.931e-43
3	Fibroblasts	*LMO4*	0.3969	0.625	0.591	5.013e-12	1.158e-07
0	Fibroblasts	*LOC100352968*	0.3965	0.966	0.908	5.292e-96	1.222e-91
4	Fibroblasts	*PEG10*	0.3963	0.357	0.262	8.149e-07	1.882e-02
0	Fibroblasts	*PTK7*	0.3960	0.425	0.300	1.153e-42	2.663e-38
3	Fibroblasts	*SETBP1*	0.3954	0.462	0.398	1.316e-10	3.040e-06
0	Fibroblasts	*KRT19*	0.3947	0.163	0.084	1.230e-27	2.840e-23
4	Fibroblasts	*PRRX2*	0.3939	0.491	0.278	1.215e-21	2.805e-17
1	Fibroblasts	*TEX14*	0.3935	0.549	0.402	1.042e-32	2.406e-28
4	Fibroblasts	*HMGCLL1*	0.3935	0.157	0.040	2.522e-36	5.824e-32
2	Fibroblasts	*PTPRG*	0.3934	0.562	0.323	6.243e-65	1.442e-60
4	Fibroblasts	*HEBP1*	0.3934	0.634	0.421	1.150e-18	2.655e-14
4	Fibroblasts	*IRF9*	0.3933	0.329	0.169	7.026e-21	1.623e-16
3	Fibroblasts	*F3*	0.3930	0.114	0.034	3.234e-33	7.469e-29
0	Fibroblasts	*EIF3G*	0.3928	0.889	0.804	3.400e-72	7.851e-68
3	Fibroblasts	*DAB2*	0.3928	0.210	0.103	4.110e-25	9.491e-21
4	Fibroblasts	*POLE4*	0.3927	0.561	0.346	1.748e-21	4.038e-17
0	Fibroblasts	*PLAT*	0.3925	0.408	0.277	2.095e-40	4.838e-36
3	Fibroblasts	*HTRA1*	0.3925	0.429	0.323	5.809e-18	1.342e-13
3	Fibroblasts	*COL6A1*	0.3923	0.505	0.405	4.899e-16	1.131e-11
1	Fibroblasts	*PPM1L*	0.3923	0.444	0.304	6.022e-30	1.390e-25
3	Fibroblasts	*VIM*	0.3919	0.956	0.932	1.115e-77	2.575e-73
0	Fibroblasts	*GCAT*	0.3910	0.416	0.292	1.314e-42	3.035e-38
0	Fibroblasts	*RPL35*	0.3908	1.000	0.984	3.331e-223	7.691e-219
4	Fibroblasts	*LOC138843080*	0.3907	0.759	0.559	1.458e-19	3.367e-15
3	Fibroblasts	*SLC5A3*	0.3906	0.231	0.154	1.903e-12	4.395e-08
2	Fibroblasts	*NCOA1*	0.3900	0.605	0.430	6.226e-37	1.438e-32
2	Fibroblasts	*SEMA3D*	0.3892	0.156	0.046	3.138e-57	7.247e-53
2	Fibroblasts	*ADAMTS3*	0.3891	0.232	0.095	4.758e-49	1.099e-44
3	Fibroblasts	*COL4A2*	0.3887	0.209	0.091	7.360e-32	1.699e-27
3	Fibroblasts	*KLF2*	0.3884	0.606	0.475	3.980e-22	9.190e-18
4	Fibroblasts	*HSPA8*	0.3875	0.979	0.895	5.744e-25	1.326e-20

Original cluster	Annotated cell type	Gene	avg_log2FC	pct.1	pct.2	Raw P value	Bonferroni-adjusted P value
3	Fibroblasts	*ANXA2*	0.3869	0.297	0.160	9.042e-29	2.088e-24
4	Fibroblasts	*SRPX*	0.3868	0.498	0.298	4.222e-20	9.749e-16
1	Fibroblasts	*AFF3*	0.3867	0.497	0.304	2.970e-47	6.859e-43
4	Fibroblasts	*TCEA3*	0.3866	0.471	0.256	3.674e-24	8.485e-20
4	Fibroblasts	*TIMP1*	0.3865	0.629	0.424	4.869e-20	1.124e-15
4	Fibroblasts	*MYLIP*	0.3855	0.512	0.312	4.257e-21	9.831e-17
2	Fibroblasts	*INHBA*	0.3848	0.324	0.170	6.279e-42	1.450e-37
3	Fibroblasts	*NCOA7*	0.3846	0.340	0.262	1.089e-11	2.516e-07
0	Fibroblasts	*H1-0*	0.3845	0.286	0.198	4.827e-24	1.115e-19
4	Fibroblasts	*JUNB*	0.3841	0.966	0.891	1.485e-13	3.428e-09
4	Fibroblasts	*FIS1*	0.3841	0.729	0.537	1.518e-19	3.506e-15
0	Fibroblasts	*H1-10*	0.3841	0.707	0.625	3.581e-41	8.270e-37
3	Fibroblasts	*ADRB1*	0.3841	0.106	0.022	6.201e-49	1.432e-44
3	Fibroblasts	*CMKLR2*	0.3839	0.106	0.011	1.959e-85	4.523e-81
2	Fibroblasts	*GLRX*	0.3837	0.148	0.102	1.253e-07	2.894e-03
2	Fibroblasts	*DLG1*	0.3837	0.560	0.413	1.156e-28	2.670e-24
2	Fibroblasts	*BTRC*	0.3834	0.439	0.281	2.908e-33	6.715e-29
4	Fibroblasts	*EPHX1*	0.3826	0.868	0.661	1.639e-20	3.784e-16
0	Fibroblasts	*SNX5*	0.3824	0.616	0.528	1.444e-39	3.335e-35
0	Fibroblasts	*LDHB*	0.3816	0.693	0.605	3.376e-48	7.796e-44
4	Fibroblasts	*MYC*	0.3813	0.421	0.268	3.476e-14	8.027e-10
0	Fibroblasts	*LOC108176887*	0.3809	0.354	0.264	1.163e-30	2.685e-26
2	Fibroblasts	*ARID4A*	0.3806	0.478	0.361	4.680e-21	1.081e-16
1	Fibroblasts	*LOC100353783*	0.3801	0.309	0.217	3.061e-17	7.068e-13
0	Fibroblasts	*LOC100346272*	0.3799	0.705	0.619	5.634e-48	1.301e-43
4	Fibroblasts	*CLIC1*	0.3797	0.709	0.528	5.166e-19	1.193e-14
1	Fibroblasts	*TENM4*	0.3792	0.475	0.260	8.568e-65	1.978e-60
3	Fibroblasts	*NRK*	0.3792	0.120	0.020	1.618e-66	3.736e-62
0	Fibroblasts	*EIF3M*	0.3791	0.764	0.682	7.728e-53	1.785e-48
0	Fibroblasts	*RPL39*	0.3787	0.978	0.940	1.926e-55	4.447e-51
4	Fibroblasts	*CMTM3*	0.3785	0.398	0.221	9.727e-20	2.246e-15
3	Fibroblasts	*ISLR*	0.3784	0.430	0.327	6.226e-15	1.438e-10
4	Fibroblasts	*UBE2S*	0.3781	0.636	0.490	2.319e-11	5.355e-07
2	Fibroblasts	*NPAS3*	0.3779	0.487	0.324	9.072e-35	2.095e-30
3	Fibroblasts	*VAV3*	0.3779	0.203	0.110	1.373e-18	3.169e-14
2	Fibroblasts	*MBD5*	0.3775	0.651	0.478	3.421e-36	7.899e-32
0	Fibroblasts	*CREG1*	0.3773	0.639	0.544	1.022e-44	2.359e-40
2	Fibroblasts	*SCMH1*	0.3772	0.521	0.345	2.773e-36	6.404e-32
2	Fibroblasts	*COL23A1*	0.3769	0.227	0.097	1.418e-45	3.273e-41
2	Fibroblasts	*HMCN1*	0.3769	0.221	0.085	1.490e-54	3.441e-50

Original cluster	Annotated cell type	Gene	avg_log2FC	pct.1	pct.2	Raw P value	Bonferroni-adjusted P value
2	Fibroblasts	*KIFAP3*	0.3763	0.520	0.347	1.678e-37	3.875e-33
2	Fibroblasts	*UBE4B*	0.3751	0.355	0.254	1.641e-16	3.790e-12
0	Fibroblasts	*RPL28*	0.3751	1.000	0.983	1.599e-196	3.693e-192
2	Fibroblasts	*ZEB1*	0.3748	0.595	0.416	2.724e-38	6.290e-34
3	Fibroblasts	*RNF19A*	0.3741	0.429	0.324	2.886e-16	6.665e-12
0	Fibroblasts	*RPS19*	0.3739	1.000	0.981	2.442e-177	5.639e-173
1	Fibroblasts	*PTGER3*	0.3738	0.222	0.102	1.545e-41	3.568e-37
3	Fibroblasts	*LGR4*	0.3735	0.141	0.062	1.345e-21	3.106e-17
3	Fibroblasts	*OSTF1*	0.3735	0.414	0.324	3.193e-15	7.373e-11
2	Fibroblasts	*LOC103352191*	0.3732	0.530	0.342	4.419e-40	1.020e-35
3	Fibroblasts	*FUNDC2*	0.3728	0.401	0.322	6.820e-13	1.575e-08
4	Fibroblasts	*TWIST1*	0.3728	0.370	0.267	4.701e-07	1.086e-02
0	Fibroblasts	*LOC100345694*	0.3725	0.899	0.848	2.811e-65	6.491e-61
0	Fibroblasts	*RPS10*	0.3725	1.000	0.977	1.988e-185	4.591e-181
3	Fibroblasts	*AGTR1*	0.3723	0.157	0.054	2.347e-37	5.420e-33
3	Fibroblasts	*CLIC4*	0.3720	0.418	0.356	3.381e-08	7.806e-04
3	Fibroblasts	*TP53INP1*	0.3719	0.175	0.104	4.027e-13	9.299e-09
4	Fibroblasts	*TMEM35B*	0.3716	0.489	0.299	2.543e-18	5.872e-14
3	Fibroblasts	*PTGIS*	0.3713	0.129	0.047	1.063e-28	2.455e-24
2	Fibroblasts	*DIP2B*	0.3711	0.376	0.227	9.577e-35	2.211e-30
1	Fibroblasts	*ANKS1B*	0.3709	0.329	0.227	1.281e-20	2.958e-16
3	Fibroblasts	*NMRK1*	0.3708	0.194	0.106	1.580e-18	3.648e-14
2	Fibroblasts	*PHF21A*	0.3699	0.490	0.337	4.751e-29	1.097e-24
3	Fibroblasts	*EMILIN2*	0.3699	0.169	0.083	3.230e-20	7.459e-16
0	Fibroblasts	*CHRAC1*	0.3690	0.601	0.499	1.381e-43	3.188e-39
4	Fibroblasts	*BGN*	0.3688	0.512	0.299	5.697e-22	1.316e-17
2	Fibroblasts	*TBX3*	0.3688	0.585	0.419	2.269e-32	5.239e-28
3	Fibroblasts	*GJB3*	0.3681	0.104	0.007	7.561e-108	1.746e-103
4	Fibroblasts	*IDS*	0.3680	0.475	0.311	3.911e-16	9.030e-12
0	Fibroblasts	*IL11RA*	0.3677	0.427	0.317	2.953e-38	6.820e-34
3	Fibroblasts	*FAM171B*	0.3674	0.318	0.221	3.805e-15	8.786e-11
3	Fibroblasts	*IGF1R*	0.3660	0.382	0.294	6.304e-13	1.456e-08
0	Fibroblasts	*RPS3*	0.3658	1.000	0.980	4.702e-199	1.086e-194
2	Fibroblasts	*SLC24A3*	0.3654	0.625	0.447	1.584e-34	3.658e-30
4	Fibroblasts	*EGR1*	0.3651	0.979	0.915	5.696e-19	1.315e-14
0	Fibroblasts	*RPS17*	0.3651	1.000	0.978	1.225e-165	2.829e-161
1	Fibroblasts	*TMTC4*	0.3647	0.249	0.113	1.384e-48	3.196e-44
4	Fibroblasts	*WNT2B*	0.3646	0.154	0.028	1.038e-53	2.397e-49
1	Fibroblasts	*MYL11*	0.3644	0.228	0.095	3.834e-51	8.853e-47
4	Fibroblasts	*CNTN1*	0.3632	0.396	0.209	6.014e-22	1.389e-17

Original cluster	Annotated cell type	Gene	avg_log2FC	pct.1	pct.2	Raw P value	Bonferroni-adjusted P value
3	Fibroblasts	*WNT5B*	0.3632	0.139	0.055	5.175e-26	1.195e-21
4	Fibroblasts	*COPRS*	0.3628	0.311	0.145	1.848e-24	4.268e-20
3	Fibroblasts	*FGFR1*	0.3628	0.423	0.350	3.643e-11	8.412e-07
1	Fibroblasts	*TMEM135*	0.3626	0.506	0.371	4.796e-27	1.107e-22
4	Fibroblasts	*SELENBP1*	0.3620	0.239	0.097	4.644e-25	1.072e-20
2	Fibroblasts	*NCOA2*	0.3619	0.661	0.486	7.220e-35	1.667e-30
12	Fibroblasts	*KEF51-p01*	0.3617	0.993	0.990	1.127e-09	2.604e-05
4	Fibroblasts	*PAFAH1B3*	0.3616	0.655	0.449	2.322e-18	5.361e-14
4	Fibroblasts	*CD81*	0.3610	0.991	0.946	3.593e-31	8.297e-27
3	Fibroblasts	*ARHGAP20*	0.3609	0.302	0.224	2.061e-10	4.759e-06
0	Fibroblasts	*LOC100343018*	0.3609	0.547	0.472	1.499e-33	3.461e-29
4	Fibroblasts	*PTGES*	0.3609	0.164	0.041	6.390e-40	1.476e-35
4	Fibroblasts	*S100A10*	0.3604	0.521	0.341	3.248e-15	7.499e-11
0	Fibroblasts	*SLC2A4RG*	0.3603	0.461	0.360	4.237e-35	9.784e-31
2	Fibroblasts	*RNF217*	0.3602	0.395	0.248	4.552e-32	1.051e-27
2	Fibroblasts	*FNDC3A*	0.3602	0.615	0.463	2.172e-30	5.015e-26
0	Fibroblasts	*LOC138843036*	0.3602	0.528	0.448	6.266e-32	1.447e-27
0	Fibroblasts	*RPL18*	0.3596	1.000	0.978	8.126e-152	1.877e-147
4	Fibroblasts	*HSPA2*	0.3594	0.575	0.380	5.733e-17	1.324e-12
3	Fibroblasts	*GPAT3*	0.3588	0.171	0.110	2.038e-09	4.706e-05
0	Fibroblasts	*HMGN1*	0.3586	0.708	0.609	1.597e-45	3.688e-41
0	Fibroblasts	*HSP90B1*	0.3579	0.869	0.784	3.298e-46	7.615e-42
0	Fibroblasts	*HNRNPA1*	0.3577	0.971	0.929	1.814e-72	4.190e-68
3	Fibroblasts	*PGER5*	0.3572	0.370	0.284	6.362e-12	1.469e-07
2	Fibroblasts	*RUNX2*	0.3571	0.204	0.073	1.634e-55	3.773e-51
4	Fibroblasts	*ARSJ*	0.3571	0.262	0.128	1.158e-18	2.673e-14
3	Fibroblasts	*LOC108176990*	0.3569	0.146	0.065	1.301e-20	3.005e-16
0	Fibroblasts	*NPW*	0.3567	0.213	0.157	9.266e-13	2.140e-08
0	Fibroblasts	*SLC25A4*	0.3566	0.623	0.547	1.936e-37	4.470e-33
3	Fibroblasts	*RDH14*	0.3566	0.236	0.140	2.314e-18	5.343e-14
0	Fibroblasts	*RPL17*	0.3563	0.745	0.672	4.660e-47	1.076e-42
0	Fibroblasts	*LOC103352291*	0.3561	0.801	0.734	3.003e-45	6.935e-41
0	Fibroblasts	*ECHS1*	0.3558	0.569	0.479	2.065e-38	4.768e-34
0	Fibroblasts	*RPL18A*	0.3556	1.000	0.988	1.909e-218	4.409e-214
0	Fibroblasts	*SELENOM*	0.3555	0.554	0.453	4.031e-38	9.307e-34
2	Fibroblasts	*CALCRL*	0.3549	0.520	0.314	1.903e-46	4.394e-42
3	Fibroblasts	*RND3*	0.3546	0.415	0.339	5.528e-10	1.277e-05
0	Fibroblasts	*HOXA10*	0.3544	0.376	0.289	1.799e-23	4.154e-19
4	Fibroblasts	*SPTBN1*	0.3541	0.832	0.651	3.022e-20	6.978e-16
0	Fibroblasts	*GDF7*	0.3539	0.204	0.114	1.145e-31	2.644e-27

Original cluster	Annotated cell type	Gene	avg_log2FC	pct.1	pct.2	Raw P value	Bonferroni-adjusted P value
1	Fibroblasts	*NRG4*	0.3536	0.368	0.237	2.540e-29	5.866e-25
0	Fibroblasts	*RPL13*	0.3536	1.000	0.984	5.962e-169	1.377e-164
3	Fibroblasts	*CLEC11A*	0.3531	0.215	0.144	6.124e-11	1.414e-06
0	Fibroblasts	*APOE*	0.3520	0.783	0.593	1.594e-68	3.682e-64
3	Fibroblasts	*ARHGAP5*	0.3510	0.573	0.506	2.890e-13	6.673e-09
1	Fibroblasts	*HS2ST1*	0.3509	0.351	0.236	1.756e-25	4.055e-21
4	Fibroblasts	*DNLZ*	0.3506	0.662	0.467	4.146e-18	9.575e-14
3	Fibroblasts	*COX6B2*	0.3498	0.103	0.026	2.292e-37	5.293e-33
3	Fibroblasts	*UBL3*	0.3498	0.399	0.329	7.110e-11	1.642e-06
3	Fibroblasts	*LOC103351750*	0.3496	0.143	0.073	3.864e-16	8.923e-12
2	Fibroblasts	*LRRC58*	0.3495	0.551	0.404	4.927e-27	1.138e-22
0	Fibroblasts	*EEF1G*	0.3489	0.976	0.917	7.465e-78	1.724e-73
0	Fibroblasts	*UQCRH*	0.3486	0.927	0.861	1.886e-58	4.356e-54
2	Fibroblasts	*LOC103351035*	0.3486	0.242	0.108	2.911e-44	6.723e-40
0	Fibroblasts	*RPS13*	0.3483	0.999	0.979	6.925e-163	1.599e-158
3	Fibroblasts	*H2AJ*	0.3482	0.424	0.348	1.230e-11	2.841e-07
2	Fibroblasts	*CTNNB1*	0.3479	0.578	0.492	7.007e-12	1.618e-07
1	Fibroblasts	*KIRREL3*	0.3475	0.175	0.078	1.085e-34	2.506e-30
2	Fibroblasts	*MLLT3*	0.3468	0.505	0.350	1.865e-29	4.306e-25
0	Fibroblasts	*RPS23*	0.3468	1.000	0.980	6.556e-183	1.514e-178
2	Fibroblasts	*NUBPL*	0.3459	0.381	0.248	3.736e-27	8.628e-23
1	Fibroblasts	*MAPK10*	0.3455	0.114	0.027	6.919e-58	1.598e-53
2	Fibroblasts	*ULK2*	0.3452	0.336	0.219	1.419e-21	3.276e-17
3	Fibroblasts	*GRIA3*	0.3449	0.102	0.026	1.264e-36	2.918e-32
3	Fibroblasts	*ITGA2*	0.3448	0.142	0.064	1.681e-20	3.882e-16
0	Fibroblasts	*EIF1B*	0.3447	0.755	0.681	9.722e-41	2.245e-36
4	Fibroblasts	*GABARAPL1*	0.3444	0.407	0.257	2.338e-14	5.399e-10
2	Fibroblasts	*LEF1*	0.3443	0.300	0.156	4.390e-39	1.014e-34
3	Fibroblasts	*RFTN1*	0.3441	0.228	0.137	1.693e-16	3.908e-12
3	Fibroblasts	*CD34*	0.3440	0.203	0.084	4.384e-33	1.012e-28
3	Fibroblasts	*PDK4*	0.3439	0.209	0.157	7.267e-07	1.678e-02
4	Fibroblasts	*NME2*	0.3436	0.855	0.687	3.370e-19	7.782e-15
1	Fibroblasts	*SNED1*	0.3432	0.374	0.219	5.059e-39	1.168e-34
3	Fibroblasts	*CAST*	0.3430	0.337	0.256	8.092e-12	1.869e-07
0	Fibroblasts	*TECR*	0.3428	0.467	0.377	7.743e-33	1.788e-28
2	Fibroblasts	*NF1*	0.3426	0.605	0.446	2.240e-31	5.174e-27
4	Fibroblasts	*TNFAIP8*	0.3423	0.321	0.205	8.507e-11	1.964e-06
2	Fibroblasts	*WNT2*	0.3418	0.284	0.130	6.261e-46	1.446e-41
4	Fibroblasts	*PTGFR*	0.3416	0.182	0.046	5.060e-42	1.168e-37
2	Fibroblasts	*SASH1*	0.3415	0.405	0.265	9.823e-28	2.268e-23

Original cluster	Annotated cell type	Gene	avg_log2FC	pct.1	pct.2	Raw P value	Bonferroni-adjusted P value
2	Fibroblasts	*RSRC1*	0.3403	0.657	0.508	2.408e-29	5.560e-25
2	Fibroblasts	*TASP1*	0.3401	0.422	0.289	6.956e-26	1.606e-21
3	Fibroblasts	*ACTN1*	0.3400	0.302	0.228	1.789e-10	4.131e-06
0	Fibroblasts	*RPL15*	0.3399	0.853	0.789	1.829e-49	4.223e-45
0	Fibroblasts	*NPM1*	0.3396	0.993	0.950	4.143e-91	9.566e-87
0	Fibroblasts	*PAQR7*	0.3396	0.280	0.199	1.451e-21	3.352e-17
4	Fibroblasts	*SQSTM1*	0.3384	0.891	0.741	1.807e-19	4.173e-15
4	Fibroblasts	*DDAH2*	0.3380	0.832	0.658	2.848e-17	6.578e-13
3	Fibroblasts	*LOC138843145*	0.3380	0.236	0.154	8.200e-13	1.894e-08
2	Fibroblasts	*IVNS1ABP*	0.3375	0.352	0.230	6.804e-25	1.571e-20
0	Fibroblasts	*PFDN5*	0.3374	0.965	0.921	3.272e-69	7.556e-65
4	Fibroblasts	*AQP1*	0.3374	0.182	0.062	1.039e-26	2.399e-22
2	Fibroblasts	*TBC1D32*	0.3366	0.411	0.264	2.627e-30	6.065e-26
2	Fibroblasts	*TBC1D5*	0.3359	0.656	0.504	8.216e-29	1.897e-24
4	Fibroblasts	*PROS1*	0.3355	0.318	0.146	2.794e-24	6.451e-20
3	Fibroblasts	*ACVR2A*	0.3352	0.261	0.198	1.330e-08	3.071e-04
2	Fibroblasts	*SBF2*	0.3347	0.557	0.400	7.532e-28	1.739e-23
0	Fibroblasts	*RPS18*	0.3347	1.000	0.987	6.482e-229	1.497e-224
0	Fibroblasts	*LOC103351575*	0.3347	0.600	0.529	8.517e-32	1.967e-27
3	Fibroblasts	*TRMT9B*	0.3341	0.103	0.026	1.321e-38	3.052e-34
2	Fibroblasts	*ZCCHC7*	0.3338	0.519	0.387	9.464e-23	2.185e-18
0	Fibroblasts	*IER5L*	0.3327	0.643	0.550	1.772e-27	4.092e-23
4	Fibroblasts	*SNAPIN*	0.3327	0.438	0.260	3.673e-17	8.482e-13
1	Fibroblasts	*MYO1E*	0.3326	0.382	0.288	5.238e-16	1.209e-11
4	Fibroblasts	*LOC138844731*	0.3321	0.227	0.095	1.429e-22	3.300e-18
0	Fibroblasts	*LDOC1*	0.3321	0.484	0.403	3.760e-27	8.682e-23
1	Fibroblasts	*CEBPB*	0.3321	0.826	0.715	6.305e-22	1.456e-17
0	Fibroblasts	*PPIB*	0.3315	0.829	0.769	2.953e-46	6.819e-42
3	Fibroblasts	*CYB5A*	0.3314	0.529	0.455	8.864e-14	2.047e-09
0	Fibroblasts	*LOC100351480*	0.3314	0.560	0.502	2.543e-28	5.873e-24
3	Fibroblasts	*CCDC178*	0.3313	0.104	0.037	1.830e-22	4.225e-18
3	Fibroblasts	*MITF*	0.3306	0.664	0.621	3.746e-10	8.650e-06
0	Fibroblasts	*EEF1D*	0.3304	0.942	0.906	1.881e-59	4.343e-55
3	Fibroblasts	*TCN2*	0.3303	0.229	0.146	3.339e-14	7.710e-10
1	Fibroblasts	*AMMECR1*	0.3303	0.302	0.203	7.370e-20	1.702e-15
3	Fibroblasts	*PPIC*	0.3302	0.411	0.342	1.279e-10	2.954e-06
0	Fibroblasts	*KEL*	0.3300	0.778	0.706	5.469e-38	1.263e-33
2	Fibroblasts	*GNA14*	0.3299	0.206	0.089	2.587e-39	5.974e-35
0	Fibroblasts	*EEF1B2*	0.3293	0.994	0.943	4.482e-94	1.035e-89
4	Fibroblasts	*PSAP*	0.3292	0.787	0.605	1.102e-15	2.545e-11

Original cluster	Annotated cell type	Gene	avg_log2FC	pct.1	pct.2	Raw P value	Bonferroni-adjusted P value
3	Fibroblasts	*RNASET2*	0.3284	0.299	0.219	2.188e-11	5.053e-07
2	Fibroblasts	*UGGT2*	0.3276	0.333	0.205	2.879e-27	6.648e-23
2	Fibroblasts	*LPAR1*	0.3273	0.327	0.176	2.047e-37	4.726e-33
3	Fibroblasts	*LPAR4*	0.3268	0.121	0.055	4.809e-17	1.110e-12
2	Fibroblasts	*GOPC*	0.3264	0.723	0.553	2.038e-35	4.705e-31
2	Fibroblasts	*KCNJ3*	0.3264	0.142	0.050	6.618e-40	1.528e-35
1	Fibroblasts	*AIG1*	0.3262	0.625	0.494	1.349e-26	3.115e-22
2	Fibroblasts	*SMYD3*	0.3255	0.547	0.402	9.522e-27	2.199e-22
2	Fibroblasts	*ATL2*	0.3249	0.375	0.260	8.596e-20	1.985e-15
2	Fibroblasts	*RERE*	0.3246	0.585	0.428	6.652e-29	1.536e-24
3	Fibroblasts	*BOC*	0.3245	0.176	0.094	1.139e-16	2.631e-12
2	Fibroblasts	*LRP1B*	0.3244	0.190	0.106	2.626e-20	6.063e-16
4	Fibroblasts	*CSTB*	0.3243	0.798	0.631	3.181e-16	7.347e-12
3	Fibroblasts	*PBX3*	0.3240	0.273	0.216	7.867e-07	1.817e-02
1	Fibroblasts	*DZIP3*	0.3240	0.516	0.379	9.198e-26	2.124e-21
3	Fibroblasts	*ATF7IP*	0.3238	0.508	0.443	1.211e-10	2.797e-06
3	Fibroblasts	*CITED2*	0.3230	0.505	0.475	4.261e-07	9.840e-03
0	Fibroblasts	*MFAP2*	0.3224	0.472	0.370	1.162e-29	2.683e-25
3	Fibroblasts	*LOC138844593*	0.3223	0.325	0.246	9.201e-11	2.125e-06
2	Fibroblasts	*MPDZ*	0.3222	0.348	0.224	1.377e-24	3.180e-20
4	Fibroblasts	*COL5A1*	0.3209	0.366	0.208	3.699e-16	8.541e-12
1	Fibroblasts	*EFCAB2*	0.3208	0.459	0.332	5.199e-22	1.201e-17
4	Fibroblasts	*LSM4*	0.3208	0.588	0.414	3.077e-14	7.106e-10
0	Fibroblasts	*HMGB2*	0.3206	0.809	0.719	3.988e-53	9.209e-49
2	Fibroblasts	*SPRED1*	0.3205	0.354	0.206	2.365e-35	5.461e-31
1	Fibroblasts	*PACRG*	0.3204	0.185	0.097	1.243e-25	2.871e-21
2	Fibroblasts	*TSHZ3*	0.3200	0.349	0.199	7.628e-34	1.762e-29
0	Fibroblasts	*RPS16*	0.3199	1.000	0.974	2.251e-118	5.199e-114
3	Fibroblasts	*FXYD6*	0.3182	0.408	0.322	4.755e-12	1.098e-07
3	Fibroblasts	*LOC100343872*	0.3179	0.164	0.068	1.667e-27	3.850e-23
1	Fibroblasts	*SMOC2*	0.3179	0.258	0.126	3.447e-41	7.961e-37
4	Fibroblasts	*LOC100347254*	0.3178	0.216	0.084	2.648e-24	6.116e-20
0	Fibroblasts	*GADD45G*	0.3176	0.492	0.400	7.184e-21	1.659e-16
2	Fibroblasts	*TFPI*	0.3171	0.375	0.197	6.783e-45	1.566e-40
4	Fibroblasts	*DNAJA1*	0.3169	0.786	0.697	1.365e-07	3.152e-03
2	Fibroblasts	*MCC*	0.3167	0.289	0.163	6.999e-31	1.616e-26
3	Fibroblasts	*LOC100349757*	0.3167	0.323	0.264	3.481e-08	8.039e-04
1	Fibroblasts	*DACH2*	0.3165	0.123	0.050	5.059e-29	1.168e-24
0	Fibroblasts	*RPL36A*	0.3163	0.984	0.936	8.132e-70	1.878e-65
2	Fibroblasts	*NELL1*	0.3158	0.206	0.105	1.803e-27	4.164e-23

Original cluster	Annotated cell type	Gene	avg_log2FC	pct.1	pct.2	Raw P value	Bonferroni-adjusted P value
4	Fibroblasts	*TMBIM1*	0.3157	0.248	0.109	4.158e-22	9.603e-18
4	Fibroblasts	*SOD3*	0.3151	0.462	0.275	1.258e-16	2.904e-12
4	Fibroblasts	*MGMT*	0.3150	0.257	0.110	1.839e-23	4.246e-19
1	Fibroblasts	*EPHA4*	0.3146	0.260	0.150	4.737e-28	1.094e-23
0	Fibroblasts	*HMGN2*	0.3140	0.930	0.861	7.857e-53	1.814e-48
2	Fibroblasts	*EXOC6B*	0.3138	0.631	0.452	6.223e-33	1.437e-28
0	Fibroblasts	*HSPE1*	0.3137	0.857	0.795	2.279e-42	5.262e-38
2	Fibroblasts	*CAMKMT*	0.3133	0.441	0.343	3.787e-15	8.745e-11
3	Fibroblasts	*RBPMS*	0.3130	0.556	0.477	1.304e-13	3.010e-09
3	Fibroblasts	*SVIL*	0.3126	0.441	0.374	1.989e-08	4.592e-04
0	Fibroblasts	*TGM2*	0.3123	0.286	0.205	1.404e-22	3.242e-18
0	Fibroblasts	*TPT1*	0.3118	1.000	0.983	1.683e-127	3.887e-123
3	Fibroblasts	*ARHGAP12*	0.3118	0.215	0.160	1.340e-07	3.093e-03
2	Fibroblasts	*NDNF*	0.3117	0.135	0.027	7.948e-76	1.835e-71
0	Fibroblasts	*LOC100348918*	0.3116	0.653	0.593	5.266e-31	1.216e-26
3	Fibroblasts	*MEX3B*	0.3112	0.242	0.159	9.714e-13	2.243e-08
0	Fibroblasts	*PINK1*	0.3109	0.505	0.436	8.847e-25	2.043e-20
3	Fibroblasts	*GPSM2*	0.3107	0.129	0.053	3.232e-22	7.462e-18
2	Fibroblasts	*CEP112*	0.3106	0.519	0.375	2.078e-24	4.799e-20
2	Fibroblasts	*LOC138849697*	0.3106	0.263	0.144	2.776e-29	6.410e-25
2	Fibroblasts	*PCM1*	0.3106	0.672	0.521	2.017e-23	4.658e-19
3	Fibroblasts	*APP*	0.3103	0.673	0.649	2.328e-10	5.376e-06
0	Fibroblasts	*EIF2D*	0.3103	0.348	0.286	7.788e-19	1.798e-14
2	Fibroblasts	*PCSK5*	0.3101	0.259	0.122	2.943e-41	6.795e-37
3	Fibroblasts	*MMUT*	0.3096	0.335	0.280	4.763e-07	1.100e-02
0	Fibroblasts	*BEX3*	0.3096	0.441	0.368	1.654e-23	3.819e-19
3	Fibroblasts	*SLC1A3*	0.3093	0.113	0.040	4.901e-25	1.132e-20
2	Fibroblasts	*PSD3*	0.3089	0.385	0.259	4.266e-23	9.851e-19
4	Fibroblasts	*HEXB*	0.3088	0.354	0.223	3.346e-12	7.725e-08
0	Fibroblasts	*SSR4*	0.3087	0.834	0.770	4.740e-40	1.095e-35
3	Fibroblasts	*SPRY1*	0.3079	0.144	0.075	7.646e-15	1.766e-10
1	Fibroblasts	*FAT3*	0.3076	0.242	0.132	1.923e-28	4.441e-24
0	Fibroblasts	*ATP5PO*	0.3075	0.703	0.650	1.372e-32	3.168e-28
4	Fibroblasts	*LOC100125981*	0.3074	0.380	0.236	1.908e-13	4.405e-09
0	Fibroblasts	*RTRAF*	0.3074	0.713	0.655	1.041e-33	2.404e-29
0	Fibroblasts	*CENPV*	0.3070	0.441	0.359	5.869e-27	1.355e-22
2	Fibroblasts	*CDKAL1*	0.3068	0.583	0.422	3.084e-29	7.121e-25
3	Fibroblasts	*MAPK14*	0.3053	0.259	0.196	3.011e-08	6.953e-04
4	Fibroblasts	*ITM2B*	0.3052	0.996	0.975	2.863e-32	6.610e-28
0	Fibroblasts	*RNF151*	0.3049	0.449	0.379	4.680e-21	1.081e-16

Original cluster	Annotated cell type	Gene	avg_log2FC	pct.1	pct.2	Raw P value	Bonferroni-adjusted P value
4	Fibroblasts	*TUBB4B*	0.3048	0.816	0.676	9.337e-14	2.156e-09
1	Fibroblasts	*AHI1*	0.3047	0.400	0.295	1.227e-18	2.833e-14
2	Fibroblasts	*MEF2A*	0.3043	0.431	0.323	4.089e-15	9.442e-11
3	Fibroblasts	*WNK3*	0.3043	0.162	0.112	2.497e-07	5.765e-03
1	Fibroblasts	*KHDRBS3*	0.3040	0.245	0.161	2.061e-16	4.759e-12
2	Fibroblasts	*STXBP5*	0.3038	0.399	0.258	1.613e-28	3.724e-24
2	Fibroblasts	*ESRRG*	0.3033	0.190	0.099	2.093e-24	4.834e-20
0	Fibroblasts	*UBL5*	0.3031	0.893	0.830	4.066e-47	9.390e-43
0	Fibroblasts	*HSP90AB1*	0.3031	0.980	0.938	1.095e-55	2.530e-51
3	Fibroblasts	*MTSS1*	0.3026	0.325	0.251	1.697e-09	3.918e-05
2	Fibroblasts	*CTTNBP2*	0.3025	0.209	0.099	1.829e-33	4.224e-29
2	Fibroblasts	*MAPK8*	0.3023	0.402	0.296	1.439e-15	3.324e-11
2	Fibroblasts	*ENAH*	0.3022	0.362	0.235	2.951e-25	6.814e-21
2	Fibroblasts	*FAF1*	0.3020	0.538	0.391	4.065e-27	9.387e-23
1	Fibroblasts	*MAMDC2*	0.3020	0.317	0.199	9.987e-25	2.306e-20
2	Fibroblasts	*BAZ2B*	0.3017	0.738	0.562	1.815e-33	4.192e-29
0	Fibroblasts	*MIF*	0.3017	0.784	0.712	8.245e-36	1.904e-31
0	Fibroblasts	*SH3BP5*	0.3016	0.359	0.296	2.995e-18	6.915e-14
3	Fibroblasts	*C1QTNF7*	0.3015	0.360	0.303	9.554e-07	2.206e-02
0	Fibroblasts	*LOC100008830*	0.3012	0.583	0.489	4.004e-30	9.245e-26
3	Fibroblasts	*TSC22D1*	0.3011	0.668	0.604	6.826e-12	1.576e-07
3	Fibroblasts	*FAM13A*	0.3009	0.253	0.190	5.546e-08	1.281e-03
2	Fibroblasts	*MAP1B*	0.3009	0.385	0.244	6.926e-29	1.599e-24
4	Fibroblasts	*TNFAIP6*	0.3000	0.114	0.020	1.055e-42	2.435e-38
2	Fibroblasts	*RBM20*	0.2998	0.168	0.065	3.144e-40	7.260e-36
3	Fibroblasts	*NEXN*	0.2998	0.173	0.089	8.369e-18	1.933e-13
0	Fibroblasts	*LOC138845301*	0.2996	0.432	0.363	2.586e-19	5.971e-15
3	Fibroblasts	*CHPT1*	0.2994	0.226	0.152	3.415e-11	7.885e-07
0	Fibroblasts	*LOC138843475*	0.2993	0.854	0.784	9.002e-37	2.079e-32
2	Fibroblasts	*LOC103345486*	0.2992	0.227	0.104	1.228e-37	2.837e-33
4	Fibroblasts	*POLR2E*	0.2992	0.643	0.495	1.665e-11	3.846e-07
2	Fibroblasts	*SH3D19*	0.2989	0.379	0.267	1.372e-19	3.169e-15
4	Fibroblasts	*CLDN11*	0.2989	0.227	0.101	3.367e-19	7.775e-15
2	Fibroblasts	*SOBP*	0.2985	0.206	0.097	9.466e-33	2.186e-28
1	Fibroblasts	*AASS*	0.2983	0.339	0.218	4.020e-27	9.284e-23
0	Fibroblasts	*EIF4A3*	0.2983	0.518	0.464	1.979e-24	4.571e-20
4	Fibroblasts	*OAT*	0.2981	0.554	0.387	3.142e-13	7.256e-09
3	Fibroblasts	*PPM1K*	0.2981	0.380	0.299	2.177e-11	5.028e-07
4	Fibroblasts	*ZBTB16*	0.2980	0.343	0.211	4.308e-12	9.949e-08
2	Fibroblasts	*HS3ST3A1*	0.2976	0.118	0.029	8.140e-53	1.880e-48

Original cluster	Annotated cell type	Gene	avg_log2FC	pct.1	pct.2	Raw P value	Bonferroni-adjusted P value
1	Fibroblasts	*PRKG1*	0.2975	0.934	0.712	9.511e-83	2.196e-78
2	Fibroblasts	*RUFY3*	0.2975	0.503	0.370	3.004e-22	6.937e-18
3	Fibroblasts	*RBM25*	0.2973	0.648	0.615	3.794e-09	8.762e-05
4	Fibroblasts	*TOB1*	0.2973	0.832	0.669	5.886e-12	1.359e-07
4	Fibroblasts	*TEX22*	0.2972	0.238	0.100	4.310e-22	9.954e-18
0	Fibroblasts	*FZD2*	0.2972	0.262	0.181	4.976e-23	1.149e-18
2	Fibroblasts	*RARB*	0.2972	0.222	0.103	4.283e-36	9.891e-32
4	Fibroblasts	*CD63*	0.2968	0.979	0.888	3.282e-20	7.578e-16
3	Fibroblasts	*MSRB3*	0.2967	0.323	0.242	4.780e-10	1.104e-05
1	Fibroblasts	*FOXP1*	0.2966	0.657	0.528	5.466e-24	1.262e-19
3	Fibroblasts	*TPST1*	0.2964	0.216	0.144	8.609e-11	1.988e-06
3	Fibroblasts	*PDE3A*	0.2962	0.518	0.273	3.511e-53	8.107e-49
3	Fibroblasts	*PLA2G4A*	0.2958	0.159	0.095	1.881e-11	4.344e-07
0	Fibroblasts	*PDLIM4*	0.2955	0.300	0.224	2.809e-21	6.487e-17
2	Fibroblasts	*FTO*	0.2954	0.503	0.339	1.432e-30	3.306e-26
3	Fibroblasts	*DST*	0.2950	0.629	0.574	1.487e-09	3.434e-05
3	Fibroblasts	*NPC2*	0.2948	0.383	0.331	5.444e-07	1.257e-02
4	Fibroblasts	*C5H6orf62*	0.2946	0.530	0.364	1.380e-13	3.186e-09
2	Fibroblasts	*SHPRH*	0.2945	0.431	0.300	2.378e-23	5.491e-19
0	Fibroblasts	*LOC100355379*	0.2945	0.297	0.234	6.681e-17	1.543e-12
2	Fibroblasts	*CDK12*	0.2945	0.236	0.177	3.895e-08	8.994e-04
0	Fibroblasts	*ACYP1*	0.2941	0.481	0.410	8.642e-24	1.996e-19
4	Fibroblasts	*EIF4EBP2*	0.2939	0.714	0.537	4.614e-13	1.066e-08
0	Fibroblasts	*OCIAD1*	0.2938	0.697	0.633	8.689e-31	2.007e-26
0	Fibroblasts	*GAPDH*	0.2938	0.803	0.755	1.677e-30	3.872e-26
0	Fibroblasts	*LOC100341365*	0.2937	0.496	0.448	1.262e-19	2.914e-15
4	Fibroblasts	*MRC2*	0.2934	0.245	0.099	1.206e-24	2.784e-20
2	Fibroblasts	*ZMYND11*	0.2934	0.655	0.528	1.209e-21	2.793e-17
2	Fibroblasts	*DPH6*	0.2933	0.433	0.304	9.928e-22	2.293e-17
0	Fibroblasts	*BRK1*	0.2930	0.861	0.816	1.466e-38	3.386e-34
2	Fibroblasts	*CARMIL1*	0.2930	0.313	0.181	2.388e-29	5.513e-25
3	Fibroblasts	*GRIN3A*	0.2925	0.110	0.043	9.247e-21	2.135e-16
3	Fibroblasts	*AKT3*	0.2924	0.577	0.548	2.153e-06	4.972e-02
0	Fibroblasts	*RCN1*	0.2920	0.337	0.267	2.287e-19	5.280e-15
2	Fibroblasts	*PHIP*	0.2914	0.774	0.621	4.351e-28	1.005e-23
3	Fibroblasts	*MORC4*	0.2905	0.190	0.131	2.223e-08	5.133e-04
3	Fibroblasts	*MYCBP2*	0.2905	0.491	0.453	5.292e-07	1.222e-02
1	Fibroblasts	*LOC138846070*	0.2905	0.192	0.120	2.606e-15	6.018e-11
2	Fibroblasts	*TRPC4*	0.2899	0.313	0.176	5.105e-31	1.179e-26
4	Fibroblasts	*NRG2*	0.2896	0.161	0.041	2.579e-36	5.954e-32

Original cluster	Annotated cell type	Gene	avg_log2FC	pct.1	pct.2	Raw P value	Bonferroni-adjusted P value
1	Fibroblasts	*CACNA2D1*	0.2896	0.420	0.306	4.082e-20	9.427e-16
4	Fibroblasts	*COL16A1*	0.2895	0.255	0.116	1.129e-19	2.607e-15
3	Fibroblasts	*TMEM26*	0.2890	0.134	0.066	1.697e-15	3.919e-11
3	Fibroblasts	*MAF*	0.2890	0.317	0.243	1.075e-08	2.482e-04
3	Fibroblasts	*AK3*	0.2888	0.457	0.423	5.481e-07	1.266e-02
0	Fibroblasts	*BRI3*	0.2886	0.724	0.668	1.355e-34	3.128e-30
2	Fibroblasts	*PCF11*	0.2886	0.647	0.502	4.897e-22	1.131e-17
4	Fibroblasts	*TENT5A*	0.2883	0.225	0.122	2.349e-12	5.424e-08
3	Fibroblasts	*CLMP*	0.2881	0.231	0.170	6.470e-08	1.494e-03
2	Fibroblasts	*MAT2A*	0.2880	0.550	0.431	8.332e-17	1.924e-12
2	Fibroblasts	*AP1S2*	0.2877	0.409	0.286	1.511e-20	3.488e-16
2	Fibroblasts	*LOC138844525*	0.2875	0.182	0.075	9.777e-38	2.258e-33
0	Fibroblasts	*TADA3*	0.2872	0.344	0.288	4.157e-16	9.600e-12
4	Fibroblasts	*NDRG1*	0.2872	0.355	0.195	5.884e-17	1.359e-12
3	Fibroblasts	*TLN2*	0.2871	0.180	0.114	1.673e-10	3.863e-06
3	Fibroblasts	*MTARC2*	0.2870	0.377	0.322	1.746e-07	4.033e-03
3	Fibroblasts	*MNDA*	0.2865	0.383	0.321	8.925e-08	2.061e-03
2	Fibroblasts	*PDS5B*	0.2855	0.555	0.419	5.257e-23	1.214e-18
3	Fibroblasts	*FAM210B*	0.2855	0.174	0.102	2.191e-13	5.060e-09
4	Fibroblasts	*LOC100353437*	0.2851	0.243	0.114	3.129e-18	7.224e-14
3	Fibroblasts	*CREBRF*	0.2848	0.573	0.541	1.783e-07	4.118e-03
4	Fibroblasts	*EPB41L3*	0.2848	0.327	0.174	2.986e-17	6.894e-13
2	Fibroblasts	*ITPR2*	0.2841	0.400	0.242	6.969e-33	1.609e-28
0	Fibroblasts	*ZNF503*	0.2841	0.525	0.453	8.259e-20	1.907e-15
3	Fibroblasts	*VKORC1*	0.2838	0.606	0.560	5.650e-10	1.305e-05
3	Fibroblasts	*GATA2*	0.2835	0.490	0.439	1.100e-07	2.539e-03
1	Fibroblasts	*EFNB2*	0.2833	0.318	0.214	4.023e-19	9.290e-15
0	Fibroblasts	*GATD3*	0.2832	0.470	0.417	1.714e-19	3.957e-15
2	Fibroblasts	*LOC100353508*	0.2830	0.637	0.506	5.168e-21	1.193e-16
0	Fibroblasts	*GNPDA1*	0.2828	0.211	0.154	1.333e-14	3.077e-10
2	Fibroblasts	*LOC138843509*	0.2828	0.325	0.213	4.085e-21	9.434e-17
0	Fibroblasts	*TXN2*	0.2822	0.369	0.316	1.054e-15	2.435e-11
2	Fibroblasts	*PPP2R3A*	0.2821	0.397	0.276	7.508e-21	1.734e-16
0	Fibroblasts	*CDK4*	0.2820	0.389	0.334	1.868e-18	4.314e-14
2	Fibroblasts	*PTPRM*	0.2820	0.412	0.238	4.608e-38	1.064e-33
2	Fibroblasts	*NBAS*	0.2819	0.413	0.284	8.743e-23	2.019e-18
1	Fibroblasts	*FOSB*	0.2816	0.912	0.840	3.038e-20	7.015e-16
2	Fibroblasts	*DDR2*	0.2815	0.563	0.403	2.994e-25	6.914e-21
2	Fibroblasts	*CACNA1D*	0.2815	0.158	0.053	4.755e-48	1.098e-43
2	Fibroblasts	*KCTD12*	0.2815	0.430	0.264	6.420e-34	1.483e-29

Original cluster	Annotated cell type	Gene	avg_log2FC	pct.1	pct.2	Raw P value	Bonferroni-adjusted P value
0	Fibroblasts	*TST*	0.2813	0.253	0.199	1.620e-13	3.741e-09
4	Fibroblasts	*ENPEP*	0.2813	0.161	0.047	5.335e-30	1.232e-25
1	Fibroblasts	*RB1CC1*	0.2810	0.475	0.415	1.326e-07	3.063e-03
2	Fibroblasts	*GABRB1*	0.2809	0.118	0.065	3.833e-13	8.851e-09
2	Fibroblasts	*TRA2A*	0.2809	0.697	0.540	1.072e-25	2.476e-21
0	Fibroblasts	*PSENEN*	0.2806	0.651	0.593	1.542e-28	3.561e-24
0	Fibroblasts	*RPL13A*	0.2805	1.000	0.983	1.734e-101	4.005e-97
2	Fibroblasts	*ANK3*	0.2804	0.400	0.225	7.080e-41	1.635e-36
3	Fibroblasts	*EMX2*	0.2804	0.445	0.381	1.609e-08	3.715e-04
2	Fibroblasts	*STAU2*	0.2802	0.642	0.501	6.506e-25	1.502e-20
2	Fibroblasts	*RAB28*	0.2800	0.379	0.268	8.963e-19	2.070e-14
4	Fibroblasts	*PAK3*	0.2800	0.175	0.062	1.976e-23	4.563e-19
4	Fibroblasts	*ISG15*	0.2798	0.109	0.041	3.305e-14	7.631e-10
3	Fibroblasts	*TACC1*	0.2797	0.290	0.225	5.326e-08	1.230e-03
3	Fibroblasts	*LOC138845283*	0.2795	0.291	0.218	3.862e-09	8.918e-05
0	Fibroblasts	*LOC100351756*	0.2795	0.381	0.323	4.147e-17	9.575e-13
3	Fibroblasts	*CALD1*	0.2794	0.771	0.661	8.205e-26	1.895e-21
4	Fibroblasts	*CHAD*	0.2793	0.182	0.060	8.641e-28	1.995e-23
4	Fibroblasts	*VAT1*	0.2791	0.359	0.208	1.080e-14	2.494e-10
2	Fibroblasts	*ELP4*	0.2788	0.329	0.229	4.303e-16	9.938e-12
1	Fibroblasts	*WFIKKN2*	0.2785	0.187	0.084	5.537e-36	1.279e-31
2	Fibroblasts	*VPS13C*	0.2785	0.492	0.353	4.044e-22	9.338e-18
2	Fibroblasts	*TUSC3*	0.2782	0.340	0.219	2.749e-21	6.348e-17
2	Fibroblasts	*EYA4*	0.2779	0.144	0.074	4.178e-19	9.647e-15
3	Fibroblasts	*FGL2*	0.2778	0.309	0.162	5.966e-33	1.378e-28
2	Fibroblasts	*FUS*	0.2777	0.756	0.614	2.720e-26	6.281e-22
4	Fibroblasts	*SYNGR1*	0.2777	0.307	0.163	2.447e-16	5.650e-12
2	Fibroblasts	*PLA2G7*	0.2776	0.277	0.141	5.654e-36	1.306e-31
1	Fibroblasts	*ABCA5*	0.2774	0.489	0.352	1.960e-25	4.525e-21
1	Fibroblasts	*SLC25A21*	0.2771	0.206	0.135	7.635e-15	1.763e-10
2	Fibroblasts	*LOC103348945*	0.2769	0.142	0.072	7.461e-20	1.723e-15
3	Fibroblasts	*CAMK1D*	0.2766	0.123	0.065	8.880e-13	2.051e-08
2	Fibroblasts	*ZNF704*	0.2761	0.418	0.316	7.890e-15	1.822e-10
4	Fibroblasts	*SAP18*	0.2755	0.898	0.794	1.087e-12	2.511e-08
2	Fibroblasts	*PRICKLE1*	0.2754	0.292	0.165	1.245e-28	2.874e-24
2	Fibroblasts	*HIVEP2*	0.2752	0.566	0.436	6.775e-19	1.564e-14
2	Fibroblasts	*SESN3*	0.2752	0.606	0.443	6.351e-27	1.467e-22
3	Fibroblasts	*SNCAIP*	0.2746	0.177	0.116	4.315e-09	9.964e-05
3	Fibroblasts	*PDE10A*	0.2746	0.130	0.075	6.980e-10	1.612e-05
2	Fibroblasts	*TAF1*	0.2744	0.405	0.275	2.755e-22	6.363e-18

Original cluster	Annotated cell type	Gene	avg_log2FC	pct.1	pct.2	Raw P value	Bonferroni-adjusted P value
2	Fibroblasts	*XPO1*	0.2743	0.571	0.430	2.377e-23	5.490e-19
1	Fibroblasts	*ST7*	0.2737	0.253	0.166	4.021e-18	9.286e-14
3	Fibroblasts	*TMEM178A*	0.2732	0.132	0.071	1.727e-12	3.988e-08
0	Fibroblasts	*LOC138849307*	0.2726	0.267	0.208	1.972e-15	4.553e-11
2	Fibroblasts	*PIK3R1*	0.2725	0.616	0.458	1.589e-26	3.668e-22
4	Fibroblasts	*AVPR1A*	0.2724	0.150	0.052	1.286e-21	2.970e-17
1	Fibroblasts	*CEP170*	0.2724	0.509	0.403	1.039e-17	2.400e-13
1	Fibroblasts	*RBFOX2*	0.2718	0.620	0.501	4.684e-21	1.082e-16
1	Fibroblasts	*CCDC148*	0.2717	0.127	0.062	3.687e-21	8.514e-17
2	Fibroblasts	*TEAD1*	0.2716	0.582	0.422	6.239e-25	1.441e-20
0	Fibroblasts	*PRDX1*	0.2715	0.771	0.718	3.562e-31	8.226e-27
4	Fibroblasts	*ARNT*	0.2712	0.423	0.264	3.431e-14	7.924e-10
2	Fibroblasts	*PRKN*	0.2707	0.467	0.358	7.776e-17	1.796e-12
2	Fibroblasts	*TUT4*	0.2703	0.587	0.440	1.168e-25	2.698e-21
0	Fibroblasts	*LOC100359139*	0.2703	0.490	0.455	2.616e-16	6.041e-12
1	Fibroblasts	*CERS6*	0.2701	0.223	0.116	1.834e-30	4.236e-26
3	Fibroblasts	*RBPJ*	0.2691	0.496	0.456	1.516e-07	3.500e-03
0	Fibroblasts	*ADISSP*	0.2689	0.340	0.268	7.651e-20	1.767e-15
4	Fibroblasts	*ID1*	0.2688	0.909	0.809	1.325e-10	3.060e-06
2	Fibroblasts	*ARHGAP32*	0.2688	0.422	0.289	8.129e-22	1.877e-17
2	Fibroblasts	*SPIDR*	0.2685	0.440	0.334	1.949e-15	4.501e-11
4	Fibroblasts	*SIRT5*	0.2684	0.546	0.368	9.319e-13	2.152e-08
4	Fibroblasts	*SMIM1*	0.2683	0.229	0.103	1.790e-18	4.134e-14
0	Fibroblasts	*PIGP*	0.2683	0.560	0.515	1.304e-19	3.010e-15
4	Fibroblasts	*SERF2*	0.2682	0.995	0.949	1.313e-19	3.033e-15
3	Fibroblasts	*PLCL2*	0.2681	0.146	0.075	1.530e-15	3.533e-11
2	Fibroblasts	*ARB2A*	0.2681	0.681	0.530	1.796e-25	4.147e-21
0	Fibroblasts	*RPL36*	0.2677	0.991	0.965	9.146e-66	2.112e-61
0	Fibroblasts	*RPL4*	0.2672	0.804	0.747	4.463e-29	1.031e-24
0	Fibroblasts	*LOC103348041*	0.2672	0.727	0.660	7.234e-26	1.670e-21
4	Fibroblasts	*PPL*	0.2670	0.116	0.023	6.191e-37	1.430e-32
2	Fibroblasts	*XRCC5*	0.2670	0.413	0.300	1.149e-17	2.653e-13
4	Fibroblasts	*CCNG1*	0.2667	0.855	0.703	2.008e-10	4.636e-06
4	Fibroblasts	*LOC103346390*	0.2666	0.186	0.079	2.110e-17	4.873e-13
0	Fibroblasts	*SMIM20*	0.2666	0.361	0.313	3.718e-14	8.585e-10
2	Fibroblasts	*TOX3*	0.2661	0.216	0.118	2.012e-23	4.647e-19
2	Fibroblasts	*CEP76*	0.2658	0.167	0.095	8.213e-17	1.897e-12
3	Fibroblasts	*PLBD1*	0.2657	0.101	0.027	8.626e-35	1.992e-30
4	Fibroblasts	*LOC100353245*	0.2657	0.457	0.307	1.519e-12	3.508e-08
0	Fibroblasts	*NDUFV1*	0.2655	0.617	0.577	1.393e-22	3.218e-18

Original cluster	Annotated cell type	Gene	avg_log2FC	pct.1	pct.2	Raw P value	Bonferroni-adjusted P value
3	Fibroblasts	*MAP7D3*	0.2651	0.213	0.151	3.642e-08	8.409e-04
0	Fibroblasts	*GPX7*	0.2651	0.351	0.287	9.632e-17	2.224e-12
4	Fibroblasts	*LOC100359014*	0.2649	0.175	0.072	7.444e-18	1.719e-13
4	Fibroblasts	*UBC*	0.2648	0.988	0.947	3.950e-14	9.121e-10
4	Fibroblasts	*ROMO1*	0.2648	0.682	0.510	1.589e-11	3.670e-07
0	Fibroblasts	*MSRB1*	0.2641	0.338	0.281	1.264e-15	2.918e-11
2	Fibroblasts	*OXR1*	0.2641	0.572	0.443	2.566e-20	5.926e-16
2	Fibroblasts	*LOC103351793*	0.2639	0.203	0.092	1.269e-35	2.931e-31
0	Fibroblasts	*TMEM59*	0.2633	0.817	0.772	2.625e-32	6.063e-28
3	Fibroblasts	*MAML2*	0.2633	0.536	0.467	1.830e-08	4.225e-04
0	Fibroblasts	*LOC138843093*	0.2631	0.212	0.147	8.424e-18	1.945e-13
3	Fibroblasts	*KLF9*	0.2626	0.331	0.264	5.200e-08	1.201e-03
3	Fibroblasts	*LOC138845008*	0.2625	0.102	0.036	1.601e-22	3.696e-18
0	Fibroblasts	*ATP5F1D*	0.2625	0.543	0.497	9.236e-20	2.133e-15
1	Fibroblasts	*SSBP3*	0.2622	0.545	0.414	8.747e-23	2.020e-18
2	Fibroblasts	*CRYBG1*	0.2622	0.152	0.074	5.834e-23	1.347e-18
0	Fibroblasts	*RPL22L1*	0.2621	0.820	0.774	2.502e-26	5.778e-22
3	Fibroblasts	*PLP2*	0.2618	0.193	0.125	1.398e-10	3.229e-06
2	Fibroblasts	*RBM5*	0.2617	0.699	0.561	4.136e-24	9.551e-20
2	Fibroblasts	*ZNF609*	0.2615	0.444	0.316	2.582e-21	5.962e-17
2	Fibroblasts	*NPNT*	0.2614	0.261	0.166	3.396e-18	7.842e-14
2	Fibroblasts	*ZDHHC17*	0.2613	0.389	0.273	6.621e-20	1.529e-15
4	Fibroblasts	*COL21A1*	0.2611	0.279	0.152	7.884e-14	1.821e-09
0	Fibroblasts	*LOC108178711*	0.2608	0.416	0.377	1.613e-13	3.724e-09
2	Fibroblasts	*HGSNAT*	0.2607	0.582	0.435	7.616e-23	1.759e-18
0	Fibroblasts	*EDF1*	0.2605	0.770	0.722	1.822e-30	4.207e-26
0	Fibroblasts	*MRPL36*	0.2604	0.440	0.396	4.625e-15	1.068e-10
4	Fibroblasts	*UAP1*	0.2604	0.218	0.124	2.897e-10	6.689e-06
1	Fibroblasts	*PRKCE*	0.2602	0.312	0.221	5.487e-16	1.267e-11
2	Fibroblasts	*PTBP2*	0.2597	0.538	0.409	9.220e-19	2.129e-14
0	Fibroblasts	*NENF*	0.2596	0.585	0.539	1.969e-20	4.546e-16
3	Fibroblasts	*TMEM141*	0.2584	0.184	0.121	1.280e-09	2.956e-05
0	Fibroblasts	*SCPEP1*	0.2578	0.469	0.418	1.285e-17	2.967e-13
2	Fibroblasts	*C11H12orf42*	0.2573	0.155	0.084	1.046e-17	2.415e-13
2	Fibroblasts	*AFF1*	0.2572	0.335	0.235	2.300e-16	5.311e-12
0	Fibroblasts	*KRT10*	0.2570	0.506	0.439	4.559e-20	1.053e-15
0	Fibroblasts	*LOC100338677*	0.2569	0.417	0.378	2.914e-14	6.730e-10
1	Fibroblasts	*HFM1*	0.2568	0.166	0.092	2.514e-19	5.805e-15
0	Fibroblasts	*TALDO1*	0.2568	0.653	0.594	2.284e-25	5.274e-21
2	Fibroblasts	*KAT6B*	0.2559	0.522	0.387	7.035e-21	1.624e-16

Original cluster	Annotated cell type	Gene	avg_log2FC	pct.1	pct.2	Raw P value	Bonferroni-adjusted P value
1	Fibroblasts	*RAD51B*	0.2557	0.264	0.196	7.449e-11	1.720e-06
0	Fibroblasts	*DDX39B*	0.2556	0.538	0.498	2.171e-17	5.013e-13
1	Fibroblasts	*KPNA5*	0.2553	0.350	0.243	1.003e-18	2.317e-14
4	Fibroblasts	*XBP1*	0.2550	0.388	0.265	5.671e-09	1.310e-04
2	Fibroblasts	*RGS6*	0.2549	0.282	0.165	7.100e-25	1.639e-20
0	Fibroblasts	*CYP27A1*	0.2549	0.311	0.250	2.230e-15	5.151e-11
2	Fibroblasts	*LOC100338139*	0.2548	0.623	0.472	3.927e-22	9.067e-18
0	Fibroblasts	*LOC138847174*	0.2548	0.326	0.284	5.952e-12	1.374e-07
2	Fibroblasts	*DIP2C*	0.2545	0.527	0.375	8.554e-25	1.975e-20
1	Fibroblasts	*LOC103349250*	0.2541	0.283	0.181	3.253e-21	7.513e-17
3	Fibroblasts	*NPEPPS*	0.2540	0.340	0.282	2.101e-07	4.851e-03
2	Fibroblasts	*ACYP2*	0.2537	0.311	0.220	2.455e-14	5.669e-10
0	Fibroblasts	*SPINT2*	0.2536	0.494	0.430	8.303e-20	1.917e-15
0	Fibroblasts	*COX5B*	0.2528	0.669	0.616	5.279e-24	1.219e-19
1	Fibroblasts	*L3MBTL4*	0.2528	0.106	0.045	1.866e-22	4.308e-18
2	Fibroblasts	*LOC127482834*	0.2527	0.130	0.044	1.602e-38	3.698e-34
4	Fibroblasts	*IFI44L*	0.2524	0.334	0.203	7.853e-12	1.814e-07
2	Fibroblasts	*TBCK*	0.2523	0.250	0.162	2.535e-16	5.855e-12
4	Fibroblasts	*SOCS1*	0.2519	0.418	0.271	2.803e-11	6.472e-07
0	Fibroblasts	*UBA52*	0.2517	0.998	0.975	9.997e-78	2.309e-73
2	Fibroblasts	*LOC103351270*	0.2513	0.493	0.370	2.895e-17	6.685e-13
1	Fibroblasts	*FAM81A*	0.2510	0.186	0.098	8.255e-25	1.906e-20
2	Fibroblasts	*STK3*	0.2506	0.522	0.377	2.959e-23	6.832e-19
0	Fibroblasts	*ATP5F1E*	0.2501	0.834	0.785	1.631e-26	3.765e-22
1	Fibroblasts	*LOC103351686*	0.2500	0.108	0.061	3.570e-12	8.244e-08
6	**Smooth muscle cells**	*TAGLN*	4.6299	0.985	0.104	0	0
5	Smooth muscle cells	*ANO3*	4.0453	0.800	0.099	0	0
6	Smooth muscle cells	*CTNNA3*	3.8559	0.841	0.021	0	0
6	Smooth muscle cells	*ACTA2*	3.6663	0.948	0.041	0	0
6	Smooth muscle cells	*RRAD*	3.1644	0.882	0.096	0	0
6	Smooth muscle cells	*NR4A1*	3.1072	0.919	0.315	2.976e-272	6.872e-268
6	Smooth muscle cells	*CRIP1*	2.9793	0.969	0.173	0	0
6	Smooth muscle cells	*RERGL*	2.9203	0.640	0.003	0	0
6	Smooth muscle cells	*SGCZ*	2.8814	0.690	0.019	0	0
6	Smooth muscle cells	*TPM1*	2.8634	0.983	0.364	0	0
6	Smooth muscle cells	*PDE3A*	2.8225	0.969	0.264	0	0
6	Smooth muscle cells	*DMD*	2.7519	0.810	0.153	0	0
6	Smooth muscle cells	*MGP*	2.7376	0.983	0.323	7.149e-292	1.651e-287
5	Smooth muscle cells	*RGS5*	2.7016	0.515	0.052	0	0
6	Smooth muscle cells	*COL25A1*	2.6802	0.797	0.057	0	0

Original cluster	Annotated cell type	Gene	avg_log2FC	pct.1	pct.2	Raw P value	Bonferroni-adjusted P value
5	Smooth muscle cells	*RGS4*	2.6771	0.647	0.064	0	0
6	Smooth muscle cells	*MYH11*	2.6385	0.858	0.058	0	0
6	Smooth muscle cells	*FRY*	2.6292	0.834	0.117	0	0
6	Smooth muscle cells	*SNCG*	2.5549	0.777	0.014	0	0
5	Smooth muscle cells	*SOX5*	2.5488	0.867	0.365	1.988e-239	4.591e-235
6	Smooth muscle cells	*NR4A3*	2.4990	0.766	0.114	0	0
5	Smooth muscle cells	*EBF1*	2.4729	0.699	0.141	7.103e-281	1.640e-276
6	Smooth muscle cells	*MUSTN1*	2.4581	0.550	0.011	0	0
6	Smooth muscle cells	*SORBS2*	2.4251	0.764	0.056	0	0
6	Smooth muscle cells	*BCAM*	2.3841	0.917	0.089	0	0
6	Smooth muscle cells	*SLIT3*	2.3813	0.902	0.127	0	0
6	Smooth muscle cells	*MEF2C*	2.3747	0.908	0.093	0	0
6	Smooth muscle cells	*NR4A2*	2.3702	0.797	0.114	0	0
6	Smooth muscle cells	*GADD45B*	2.3321	0.934	0.225	0	0
6	Smooth muscle cells	*C1H11orf96*	2.3279	0.961	0.253	2.523e-296	5.826e-292
6	Smooth muscle cells	*MYOCD*	2.2976	0.675	0.027	0	0
6	Smooth muscle cells	*KCNAB1*	2.2933	0.812	0.221	4.667e-242	1.078e-237
6	Smooth muscle cells	*ZFHX3*	2.2506	0.845	0.165	0	0
6	Smooth muscle cells	*ID4*	2.2464	0.828	0.169	1.095e-295	2.528e-291
5	Smooth muscle cells	*EPHA3*	2.2288	0.583	0.094	4.940e-276	1.141e-271
6	Smooth muscle cells	*PRKAR2B*	2.2263	0.830	0.072	0	0
5	Smooth muscle cells	*GRIP1*	2.2204	0.659	0.174	4.099e-206	9.465e-202
5	Smooth muscle cells	*MYO1B*	2.2142	0.696	0.183	4.930e-246	1.139e-241
6	Smooth muscle cells	*IL33*	2.2074	0.745	0.028	0	0
6	Smooth muscle cells	*LOC100343557*	2.1957	0.539	0.263	3.300e-52	7.621e-48
6	Smooth muscle cells	*COX4I2*	2.1821	0.764	0.027	0	0
5	Smooth muscle cells	*ADGRL3*	2.1625	0.761	0.308	7.961e-173	1.838e-168
5	Smooth muscle cells	*LPL*	2.1600	0.636	0.079	0	0
6	Smooth muscle cells	*TPM2*	2.1588	0.961	0.457	3.874e-237	8.947e-233
5	Smooth muscle cells	*GUCY1A2*	2.1475	0.423	0.012	0	0
5	Smooth muscle cells	*DLC1*	2.1311	0.836	0.510	1.397e-146	3.226e-142
6	Smooth muscle cells	*ERRFI1*	2.1005	0.793	0.308	1.713e-154	3.955e-150
6	Smooth muscle cells	*AKAP6*	2.0926	0.644	0.018	0	0
5	Smooth muscle cells	*INPP4B*	2.0798	0.710	0.210	4.756e-203	1.098e-198
6	Smooth muscle cells	*ADIPOQ*	2.0788	0.633	0.009	0	0
6	Smooth muscle cells	*SDC4*	2.0743	0.563	0.044	0	0
6	Smooth muscle cells	*NET1*	2.0640	0.836	0.190	6.078e-305	1.403e-300
6	Smooth muscle cells	*ATF3*	2.0625	0.928	0.490	9.554e-161	2.206e-156
6	Smooth muscle cells	*MYL6*	2.0585	0.991	0.720	2.969e-237	6.857e-233
6	Smooth muscle cells	*IGFBP7*	2.0043	0.985	0.496	5.361e-221	1.238e-216

Original cluster	Annotated cell type	Gene	avg_log2FC	pct.1	pct.2	Raw P value	Bonferroni-adjusted P value
5	Smooth muscle cells	*CHSY3*	2.0010	0.565	0.161	6.661e-154	1.538e-149
5	Smooth muscle cells	*FHL5*	1.9954	0.603	0.045	0	0
6	Smooth muscle cells	*DSTN*	1.9770	0.945	0.548	3.216e-188	7.427e-184
5	Smooth muscle cells	*CCN1*	1.9638	0.494	0.175	3.071e-91	7.092e-87
6	Smooth muscle cells	*MYL9*	1.9465	0.854	0.192	7.752e-302	1.790e-297
6	Smooth muscle cells	*TINAGL1*	1.9437	0.773	0.040	0	0
6	Smooth muscle cells	*RELN*	1.9301	0.672	0.022	0	0
5	Smooth muscle cells	*ZEB2*	1.9203	0.843	0.446	1.549e-164	3.576e-160
6	Smooth muscle cells	*SPARCL1*	1.9203	0.989	0.774	2.939e-214	6.787e-210
6	Smooth muscle cells	*ETS2*	1.9086	0.723	0.148	1.222e-253	2.823e-249
6	Smooth muscle cells	*FRMD4B*	1.9078	0.838	0.181	1.327e-292	3.064e-288
5	Smooth muscle cells	*SEMA5A*	1.9075	0.778	0.355	2.162e-172	4.991e-168
6	Smooth muscle cells	*ADAMTS1*	1.8905	0.721	0.286	5.800e-115	1.339e-110
6	Smooth muscle cells	*ROCK1*	1.8898	0.921	0.402	4.754e-203	1.098e-198
6	Smooth muscle cells	*NOTCH3*	1.8877	0.773	0.048	0	0
6	Smooth muscle cells	*CREM*	1.8692	0.596	0.131	8.220e-176	1.898e-171
6	Smooth muscle cells	*PDLIM5*	1.8490	0.821	0.210	5.168e-239	1.193e-234
5	Smooth muscle cells	*KCNMB2*	1.8449	0.464	0.109	1.147e-146	2.649e-142
6	Smooth muscle cells	*MCAM*	1.8160	0.692	0.039	0	0
6	Smooth muscle cells	*DGKB*	1.8022	0.823	0.288	4.184e-174	9.661e-170
5	Smooth muscle cells	*ITGA1*	1.8015	0.605	0.142	2.450e-205	5.659e-201
6	Smooth muscle cells	*TMEM51*	1.7907	0.762	0.045	0	0
6	Smooth muscle cells	*PRKG1*	1.7700	0.956	0.742	6.904e-119	1.594e-114
5	Smooth muscle cells	*ISYNA1*	1.7666	0.829	0.397	2.209e-177	5.101e-173
6	Smooth muscle cells	*CAV1*	1.7626	0.932	0.216	1.418e-307	3.274e-303
6	Smooth muscle cells	*UTRN*	1.7570	0.943	0.530	1.995e-170	4.607e-166
5	Smooth muscle cells	*CPNE8*	1.7350	0.579	0.138	4.998e-199	1.154e-194
5	Smooth muscle cells	*MYADM*	1.7091	0.758	0.523	9.756e-90	2.253e-85
5	Smooth muscle cells	*PARM1*	1.7028	0.585	0.157	4.407e-172	1.018e-167
6	Smooth muscle cells	*CSRNP1*	1.6933	0.806	0.189	2.101e-238	4.853e-234
6	Smooth muscle cells	*LOC100343802*	1.6865	0.541	0.250	2.220e-54	5.127e-50
6	Smooth muscle cells	*CACNB2*	1.6834	0.849	0.327	1.004e-160	2.318e-156
6	Smooth muscle cells	*RASL11A*	1.6764	0.509	0.070	1.535e-229	3.546e-225
6	Smooth muscle cells	*FILIP1*	1.6430	0.607	0.052	0	0
6	Smooth muscle cells	*KLF2*	1.6379	0.919	0.466	8.377e-138	1.934e-133
6	Smooth muscle cells	*EDNRA*	1.6313	0.755	0.107	0	0
5	Smooth muscle cells	*EPAS1*	1.6296	0.863	0.506	3.692e-155	8.526e-151
6	Smooth muscle cells	*PPP1R12A*	1.6182	0.963	0.637	2.072e-161	4.784e-157
6	Smooth muscle cells	*LRRTM3*	1.5880	0.496	0.009	0	0
6	Smooth muscle cells	*LBH*	1.5873	0.640	0.045	0	0

Original cluster	Annotated cell type	Gene	avg_log2FC	pct.1	pct.2	Raw P value	Bonferroni-adjusted P value
6	Smooth muscle cells	*RGS6*	1.5860	0.679	0.158	5.862e-186	1.354e-181
6	Smooth muscle cells	*COQ10B*	1.5840	0.740	0.235	9.839e-161	2.272e-156
6	Smooth muscle cells	*RHOB*	1.5809	0.963	0.694	8.580e-139	1.981e-134
6	Smooth muscle cells	*LOC108175368*	1.5759	0.445	0.019	0	0
5	Smooth muscle cells	*IRF1*	1.5749	0.599	0.362	1.162e-57	2.683e-53
5	Smooth muscle cells	*CALD1*	1.5575	0.905	0.658	4.416e-151	1.020e-146
5	Smooth muscle cells	*PEAK1*	1.5520	0.519	0.178	5.524e-111	1.276e-106
5	Smooth muscle cells	*SERPINE2*	1.5507	0.368	0.055	4.490e-177	1.037e-172
5	Smooth muscle cells	*STAC*	1.5416	0.315	0.019	6.709e-292	1.549e-287
6	Smooth muscle cells	*CH25H*	1.5296	0.360	0.034	3.395e-214	7.839e-210
5	Smooth muscle cells	*NCKAP5*	1.5265	0.359	0.096	5.501e-92	1.270e-87
5	Smooth muscle cells	*RCAN2*	1.5164	0.461	0.123	5.806e-127	1.341e-122
5	Smooth muscle cells	*TSHR*	1.5163	0.319	0.031	4.653e-212	1.074e-207
6	Smooth muscle cells	*MSRB3*	1.5128	0.714	0.227	4.947e-147	1.142e-142
6	Smooth muscle cells	*VIM*	1.5041	0.989	0.932	2.910e-112	6.720e-108
6	Smooth muscle cells	*CSRP1*	1.5017	0.764	0.323	4.543e-120	1.049e-115
6	Smooth muscle cells	*PLCL1*	1.4946	0.670	0.232	1.603e-111	3.702e-107
5	Smooth muscle cells	*PDGFRB*	1.4943	0.581	0.199	7.321e-135	1.691e-130
6	Smooth muscle cells	*DBNDD2*	1.4931	0.600	0.024	0	0
6	Smooth muscle cells	*DGKH*	1.4855	0.747	0.220	8.546e-173	1.973e-168
6	Smooth muscle cells	*MYLK*	1.4791	0.670	0.050	0	0
6	Smooth muscle cells	*CAVIN1*	1.4770	0.838	0.228	1.206e-217	2.784e-213
6	Smooth muscle cells	*RNF122*	1.4748	0.480	0.090	6.478e-161	1.496e-156
5	Smooth muscle cells	*LOC138843476*	1.4618	0.601	0.294	1.420e-86	3.280e-82
6	Smooth muscle cells	*CRYAB*	1.4589	0.579	0.054	0	0
5	Smooth muscle cells	*EPS8*	1.4553	0.552	0.248	1.970e-86	4.549e-82
5	Smooth muscle cells	*SGIP1*	1.4476	0.304	0.015	0	0
6	Smooth muscle cells	*CLDN10*	1.4449	0.509	0.019	0	0
6	Smooth muscle cells	*PPARGC1A*	1.4341	0.378	0.031	6.246e-257	1.442e-252
5	Smooth muscle cells	*LOC103350563*	1.4320	0.333	0.062	7.569e-127	1.748e-122
6	Smooth muscle cells	*CAV2*	1.4311	0.793	0.209	1.127e-210	2.603e-206
6	Smooth muscle cells	*PIP5K1B*	1.4268	0.679	0.085	0	0
6	Smooth muscle cells	*MARK1*	1.4213	0.600	0.146	9.989e-149	2.307e-144
6	Smooth muscle cells	*KLF10*	1.4168	0.714	0.226	4.681e-147	1.081e-142
5	Smooth muscle cells	*GSN*	1.4168	0.843	0.508	2.040e-124	4.710e-120
5	Smooth muscle cells	*HIGD1B*	1.4077	0.268	0.011	8.998e-297	2.078e-292
6	Smooth muscle cells	*BMP5*	1.4049	0.452	0.013	0	0
6	Smooth muscle cells	*SLC8A1*	1.3867	0.810	0.382	6.475e-101	1.495e-96
6	Smooth muscle cells	*MAP3K7CL*	1.3844	0.454	0.005	0	0
6	Smooth muscle cells	*NRARP*	1.3751	0.474	0.030	0	0

Original cluster	Annotated cell type	Gene	avg_log2FC	pct.1	pct.2	Raw P value	Bonferroni-adjusted P value
6	Smooth muscle cells	*RYR2*	1.3728	0.731	0.265	7.814e-121	1.804e-116
5	Smooth muscle cells	*LOC103352191*	1.3652	0.630	0.356	1.220e-72	2.817e-68
6	Smooth muscle cells	*TBX2*	1.3616	0.548	0.032	0	0
6	Smooth muscle cells	*MAP3K5*	1.3585	0.552	0.040	0	0
5	Smooth muscle cells	*LOC100346101*	1.3573	0.415	0.125	1.088e-98	2.514e-94
6	Smooth muscle cells	*TM4SF1*	1.3421	0.603	0.044	0	0
5	Smooth muscle cells	*ADGRF5*	1.3416	0.342	0.049	1.094e-159	2.527e-155
6	Smooth muscle cells	*SOX6*	1.3348	0.784	0.411	3.683e-83	8.506e-79
6	Smooth muscle cells	*PGF*	1.3309	0.531	0.069	1.537e-247	3.548e-243
5	Smooth muscle cells	*SLC24A3*	1.3254	0.752	0.459	7.105e-84	1.641e-79
5	Smooth muscle cells	*APOE*	1.3249	0.825	0.626	5.884e-63	1.359e-58
6	Smooth muscle cells	*CAVIN3*	1.3203	0.937	0.535	8.282e-130	1.912e-125
6	Smooth muscle cells	*FABP3*	1.3174	0.445	0.011	0	0
6	Smooth muscle cells	*WTIP*	1.3159	0.611	0.093	7.110e-258	1.642e-253
6	Smooth muscle cells	*PALM2AKAP2*	1.3142	0.679	0.110	1.359e-254	3.139e-250
5	Smooth muscle cells	*NR2F1*	1.3087	0.681	0.373	1.559e-90	3.601e-86
6	Smooth muscle cells	*NRP1*	1.3077	0.659	0.114	3.798e-239	8.770e-235
5	Smooth muscle cells	*PTEN*	1.3034	0.668	0.439	1.213e-65	2.802e-61
6	Smooth muscle cells	*ATP2A2*	1.3004	0.686	0.162	9.610e-187	2.219e-182
5	Smooth muscle cells	*ARHGEF17*	1.2972	0.423	0.073	2.264e-178	5.228e-174
6	Smooth muscle cells	*TCAP*	1.2938	0.450	0.009	0	0
5	Smooth muscle cells	*GUCY1B1*	1.2936	0.344	0.040	2.754e-203	6.360e-199
6	Smooth muscle cells	*HTRA4*	1.2859	0.443	0.024	0	0
6	Smooth muscle cells	*TIPARP*	1.2843	0.705	0.320	6.883e-76	1.589e-71
6	Smooth muscle cells	*DCC*	1.2841	0.380	0.031	6.083e-255	1.405e-250
5	Smooth muscle cells	*LAMA4*	1.2833	0.455	0.192	9.756e-68	2.253e-63
6	Smooth muscle cells	*PLCE1*	1.2828	0.627	0.132	1.074e-184	2.481e-180
6	Smooth muscle cells	*RASGEF1B*	1.2808	0.566	0.115	1.559e-171	3.600e-167
5	Smooth muscle cells	*GUCY1A1*	1.2803	0.306	0.050	5.502e-132	1.271e-127
5	Smooth muscle cells	*KLHL23*	1.2753	0.357	0.087	3.350e-103	7.736e-99
5	Smooth muscle cells	*FAM162B*	1.2748	0.366	0.061	4.229e-158	9.766e-154
6	Smooth muscle cells	*UBA2*	1.2696	0.697	0.175	1.480e-179	3.418e-175
6	Smooth muscle cells	*FBXO33*	1.2689	0.544	0.198	1.104e-77	2.548e-73
6	Smooth muscle cells	*UGDH*	1.2682	0.563	0.217	7.114e-74	1.643e-69
5	Smooth muscle cells	*PHLDA2*	1.2677	0.230	0.108	3.186e-21	7.356e-17
6	Smooth muscle cells	*PPP1R12B*	1.2661	0.657	0.145	4.297e-190	9.923e-186
5	Smooth muscle cells	*STK39*	1.2650	0.437	0.213	4.587e-52	1.059e-47
5	Smooth muscle cells	*FIGN*	1.2533	0.348	0.097	1.136e-81	2.624e-77
5	Smooth muscle cells	*LAMA2*	1.2511	0.685	0.571	7.834e-32	1.809e-27
5	Smooth muscle cells	*JUND*	1.2498	0.922	0.960	2.202e-81	5.086e-77

Original cluster	Annotated cell type	Gene	avg_log2FC	pct.1	pct.2	Raw P value	Bonferroni-adjusted P value
6	Smooth muscle cells	*LMNA*	1.2443	0.727	0.242	3.195e-128	7.378e-124
6	Smooth muscle cells	*OAZ2*	1.2420	0.775	0.295	9.277e-125	2.142e-120
6	Smooth muscle cells	*LGALS1*	1.2319	0.952	0.513	1.041e-134	2.404e-130
6	Smooth muscle cells	*CALM1*	1.2294	0.978	0.735	1.614e-123	3.728e-119
5	Smooth muscle cells	*GEM*	1.2290	0.357	0.100	2.192e-85	5.062e-81
5	Smooth muscle cells	*HDAC9*	1.2131	0.572	0.313	2.845e-56	6.569e-52
5	Smooth muscle cells	*KCNH1*	1.2125	0.308	0.021	2.728e-264	6.299e-260
6	Smooth muscle cells	*COL4A1*	1.2119	0.627	0.185	3.210e-119	7.412e-115
6	Smooth muscle cells	*HOMER1*	1.2084	0.491	0.228	5.434e-45	1.255e-40
5	Smooth muscle cells	*CCDC80*	1.2082	0.392	0.130	5.973e-79	1.379e-74
5	Smooth muscle cells	*ABI3BP*	1.2080	0.517	0.211	6.191e-80	1.430e-75
6	Smooth muscle cells	*NEXN*	1.2034	0.548	0.074	7.617e-243	1.759e-238
5	Smooth muscle cells	*UACA*	1.2024	0.692	0.493	2.528e-58	5.839e-54
6	Smooth muscle cells	*PDLIM3*	1.2009	0.544	0.103	1.260e-169	2.910e-165
6	Smooth muscle cells	*PDGFA*	1.1981	0.561	0.042	0	0
6	Smooth muscle cells	*ITGB1*	1.1973	0.873	0.437	1.070e-127	2.471e-123
5	Smooth muscle cells	*BGN*	1.1872	0.565	0.296	4.610e-68	1.065e-63
6	Smooth muscle cells	*TPM4*	1.1866	0.734	0.330	3.374e-90	7.790e-86
5	Smooth muscle cells	*PTPRG*	1.1840	0.565	0.349	1.737e-48	4.012e-44
5	Smooth muscle cells	*TRIO*	1.1818	0.519	0.252	1.922e-66	4.438e-62
6	Smooth muscle cells	*C6H8orf34*	1.1805	0.373	0.029	8.012e-261	1.850e-256
6	Smooth muscle cells	*HIP1*	1.1758	0.559	0.091	7.042e-213	1.626e-208
6	Smooth muscle cells	*CRIM1*	1.1738	0.773	0.429	3.361e-68	7.762e-64
5	Smooth muscle cells	*FNDC3B*	1.1738	0.719	0.535	6.754e-61	1.560e-56
6	Smooth muscle cells	*ESAM*	1.1676	0.557	0.044	0	0
6	Smooth muscle cells	*ITGA4*	1.1668	0.483	0.027	0	0
5	Smooth muscle cells	*COBLL1*	1.1609	0.519	0.256	6.575e-61	1.518e-56
5	Smooth muscle cells	*PAWR*	1.1591	0.415	0.174	1.422e-60	3.283e-56
6	Smooth muscle cells	*ATOSA*	1.1578	0.631	0.276	4.257e-72	9.829e-68
5	Smooth muscle cells	*ZEB1*	1.1538	0.583	0.437	2.413e-32	5.573e-28
6	Smooth muscle cells	*PHLDB2*	1.1533	0.681	0.235	1.342e-114	3.099e-110
5	Smooth muscle cells	*DTD1*	1.1500	0.546	0.360	2.325e-43	5.369e-39
6	Smooth muscle cells	*DUSP14*	1.1387	0.314	0.029	1.338e-190	3.090e-186
5	Smooth muscle cells	*NR2F2*	1.1378	0.916	0.778	9.581e-94	2.213e-89
6	Smooth muscle cells	*SMARCA5*	1.1368	0.697	0.326	2.401e-85	5.544e-81
5	Smooth muscle cells	*CACNA1C*	1.1361	0.517	0.180	6.946e-93	1.604e-88
5	Smooth muscle cells	*STOM*	1.1345	0.485	0.188	2.682e-86	6.194e-82
6	Smooth muscle cells	*CCNL1*	1.1272	0.904	0.657	5.538e-80	1.279e-75
5	Smooth muscle cells	*CC2D2A*	1.1249	0.488	0.265	1.267e-50	2.925e-46
5	Smooth muscle cells	*P2RY14*	1.1197	0.291	0.065	6.650e-89	1.536e-84

Original cluster	Annotated cell type	Gene	avg_log2FC	pct.1	pct.2	Raw P value	Bonferroni-adjusted P value
6	Smooth muscle cells	*ITGA8*	1.1185	0.465	0.059	1.376e-218	3.177e-214
6	Smooth muscle cells	*ARHGAP44*	1.1160	0.441	0.047	3.171e-240	7.322e-236
6	Smooth muscle cells	*MAP2*	1.1118	0.555	0.110	1.073e-165	2.477e-161
6	Smooth muscle cells	*SAMD4A*	1.1056	0.607	0.186	6.073e-113	1.402e-108
6	Smooth muscle cells	*TBC1D1*	1.1037	0.513	0.065	5.065e-245	1.170e-240
6	Smooth muscle cells	*PLP2*	1.0973	0.576	0.109	6.912e-186	1.596e-181
6	Smooth muscle cells	*MFGE8*	1.0919	0.598	0.138	5.568e-156	1.286e-151
6	Smooth muscle cells	*B3GNT2*	1.0916	0.485	0.055	3.457e-252	7.983e-248
6	Smooth muscle cells	*LOC100343299*	1.0903	0.290	0.123	7.179e-27	1.658e-22
5	Smooth muscle cells	*CTSC*	1.0879	0.545	0.316	2.436e-53	5.626e-49
6	Smooth muscle cells	*ASTN2*	1.0857	0.430	0.040	3.350e-258	7.735e-254
6	Smooth muscle cells	*OLFM1*	1.0817	0.509	0.049	2.964e-301	6.845e-297
6	Smooth muscle cells	*AFAP1L2*	1.0747	0.480	0.087	1.301e-159	3.005e-155
6	Smooth muscle cells	*MYO6*	1.0706	0.284	0.104	1.541e-34	3.560e-30
6	Smooth muscle cells	*PEX3*	1.0649	0.463	0.200	4.438e-45	1.025e-40
5	Smooth muscle cells	*GPC6*	1.0623	0.490	0.304	7.034e-31	1.624e-26
5	Smooth muscle cells	*REM1*	1.0620	0.370	0.115	4.501e-80	1.039e-75
6	Smooth muscle cells	*ACTG1*	1.0619	0.974	0.814	1.566e-89	3.617e-85
5	Smooth muscle cells	*PLD1*	1.0603	0.455	0.256	8.343e-40	1.926e-35
6	Smooth muscle cells	*LAMA3*	1.0573	0.360	0.019	0	0
6	Smooth muscle cells	*SORT1*	1.0549	0.616	0.163	6.598e-139	1.524e-134
5	Smooth muscle cells	*FAT1*	1.0546	0.410	0.215	7.057e-40	1.630e-35
6	Smooth muscle cells	*JAG1*	1.0506	0.526	0.083	5.791e-200	1.337e-195
6	Smooth muscle cells	*ITGA7*	1.0496	0.430	0.008	0	0
5	Smooth muscle cells	*KCNJ8*	1.0487	0.259	0.026	8.262e-168	1.908e-163
5	Smooth muscle cells	*FOXS1*	1.0482	0.231	0.017	9.294e-181	2.146e-176
5	Smooth muscle cells	*LOC108176990*	1.0429	0.277	0.061	7.026e-83	1.622e-78
6	Smooth muscle cells	*HSPB8*	1.0384	0.367	0.021	0	0
6	Smooth muscle cells	*LOC138849590*	1.0383	0.688	0.272	4.897e-89	1.131e-84
6	Smooth muscle cells	*VCL*	1.0325	0.705	0.261	5.093e-105	1.176e-100
6	Smooth muscle cells	*AOPEP*	1.0324	0.779	0.360	1.385e-91	3.198e-87
5	Smooth muscle cells	*DIAPH2*	1.0315	0.599	0.409	4.977e-38	1.149e-33
5	Smooth muscle cells	*PDLIM1*	1.0301	0.264	0.043	4.708e-114	1.087e-109
5	Smooth muscle cells	*AGAP1*	1.0299	0.366	0.125	4.273e-68	9.866e-64
6	Smooth muscle cells	*CDH6*	1.0288	0.456	0.031	0	0
5	Smooth muscle cells	*ADAM9*	1.0269	0.441	0.251	2.714e-39	6.268e-35
5	Smooth muscle cells	*ABCC9*	1.0265	0.306	0.056	6.463e-116	1.492e-111
5	Smooth muscle cells	*MAP4K4*	1.0247	0.550	0.394	7.018e-35	1.621e-30
6	Smooth muscle cells	*SLCO1C1*	1.0246	0.373	0.026	1.322e-278	3.053e-274
5	Smooth muscle cells	*TNFRSF12A*	1.0231	0.240	0.023	1.764e-158	4.074e-154

Original cluster	Annotated cell type	Gene	avg_log2FC	pct.1	pct.2	Raw P value	Bonferroni-adjusted P value
5	Smooth muscle cells	*LOC103350379*	1.0220	0.182	0.017	6.645e-125	1.534e-120
6	Smooth muscle cells	*FAM241A*	1.0195	0.496	0.058	1.220e-248	2.818e-244
6	Smooth muscle cells	*CRIP2*	1.0188	0.535	0.068	2.908e-250	6.716e-246
6	Smooth muscle cells	*PLCB4*	1.0097	0.517	0.068	3.753e-227	8.667e-223
6	Smooth muscle cells	*ATP10A*	1.0077	0.386	0.023	0	0
6	Smooth muscle cells	*LMOD1*	1.0040	0.439	0.029	0	0
5	Smooth muscle cells	*GHR*	1.0036	0.696	0.522	2.948e-43	6.808e-39
6	Smooth muscle cells	*DES*	1.0011	0.404	0.016	0	0
6	Smooth muscle cells	*ACTN1*	0.9980	0.642	0.215	1.403e-104	3.239e-100
6	Smooth muscle cells	*SIK1*	0.9972	0.448	0.090	4.344e-129	1.003e-124
6	Smooth muscle cells	*EDA*	0.9970	0.522	0.177	4.310e-75	9.952e-71
5	Smooth muscle cells	*MACROD2*	0.9967	0.505	0.345	2.720e-24	6.282e-20
6	Smooth muscle cells	*CNNM2*	0.9949	0.450	0.105	1.014e-111	2.342e-107
6	Smooth muscle cells	*TOB2*	0.9928	0.849	0.484	7.414e-87	1.712e-82
5	Smooth muscle cells	*PDE1A*	0.9909	0.559	0.404	6.263e-28	1.446e-23
6	Smooth muscle cells	*TES*	0.9891	0.509	0.070	2.067e-219	4.773e-215
5	Smooth muscle cells	*GTDC1*	0.9880	0.475	0.275	3.367e-38	7.775e-34
6	Smooth muscle cells	*DOCK4*	0.9879	0.441	0.087	3.854e-128	8.901e-124
6	Smooth muscle cells	*MAP3K20*	0.9794	0.758	0.327	8.920e-94	2.060e-89
6	Smooth muscle cells	*GJA4*	0.9793	0.352	0.019	0	0
6	Smooth muscle cells	*TANK*	0.9773	0.524	0.155	1.261e-94	2.912e-90
6	Smooth muscle cells	*TMEM200B*	0.9769	0.478	0.064	2.441e-208	5.636e-204
6	Smooth muscle cells	*TTLL7*	0.9749	0.386	0.013	0	0
6	Smooth muscle cells	*LMO7*	0.9745	0.456	0.039	1.944e-294	4.490e-290
5	Smooth muscle cells	*ARHGDIB*	0.9738	0.355	0.083	5.633e-102	1.301e-97
6	Smooth muscle cells	*RETREG1*	0.9693	0.487	0.078	6.238e-182	1.441e-177
6	Smooth muscle cells	*MAPRE2*	0.9675	0.672	0.254	1.097e-92	2.533e-88
5	Smooth muscle cells	*SOCS3*	0.9666	0.743	0.603	8.404e-41	1.941e-36
6	Smooth muscle cells	*MAP1LC3A*	0.9654	0.766	0.384	1.917e-79	4.426e-75
6	Smooth muscle cells	*ANGPT1*	0.9652	0.500	0.127	1.467e-107	3.387e-103
5	Smooth muscle cells	*LOC138849286*	0.9646	0.140	0.013	1.290e-92	2.978e-88
6	Smooth muscle cells	*PTPRK*	0.9619	0.712	0.345	1.538e-63	3.552e-59
6	Smooth muscle cells	*EFHD2*	0.9609	0.469	0.096	2.764e-133	6.382e-129
6	Smooth muscle cells	*ELOVL5*	0.9607	0.483	0.118	7.809e-111	1.803e-106
6	Smooth muscle cells	*TIMP1*	0.9594	0.627	0.427	7.163e-19	1.654e-14
5	Smooth muscle cells	*CD83*	0.9593	0.195	0.050	1.347e-45	3.110e-41
6	Smooth muscle cells	*USP37*	0.9568	0.533	0.208	8.071e-66	1.864e-61
5	Smooth muscle cells	*PRSS23*	0.9545	0.361	0.177	9.824e-35	2.268e-30
5	Smooth muscle cells	*EGFLAM*	0.9466	0.335	0.108	1.459e-64	3.368e-60
5	Smooth muscle cells	*PLD5*	0.9455	0.180	0.017	1.908e-120	4.407e-116

Original cluster	Annotated cell type	Gene	avg_log2FC	pct.1	pct.2	Raw P value	Bonferroni-adjusted P value
6	Smooth muscle cells	*ID1*	0.9454	0.972	0.806	2.832e-62	6.541e-58
5	Smooth muscle cells	*PGER5*	0.9453	0.552	0.277	3.255e-62	7.517e-58
6	Smooth muscle cells	*LOC103344972*	0.9410	0.496	0.094	1.795e-148	4.144e-144
6	Smooth muscle cells	*CAP2*	0.9390	0.552	0.109	2.118e-160	4.890e-156
6	Smooth muscle cells	*SIK3*	0.9387	0.614	0.283	2.697e-59	6.227e-55
5	Smooth muscle cells	*TANC1*	0.9349	0.341	0.163	2.026e-35	4.679e-31
6	Smooth muscle cells	*ELL2*	0.9339	0.428	0.141	2.192e-63	5.063e-59
5	Smooth muscle cells	*LHFPL6*	0.9327	0.678	0.494	2.738e-40	6.322e-36
6	Smooth muscle cells	*SLC25A5*	0.9268	0.799	0.375	5.540e-85	1.279e-80
5	Smooth muscle cells	*PAMR1*	0.9256	0.246	0.027	1.463e-144	3.379e-140
6	Smooth muscle cells	*LOC100340483*	0.9252	0.900	0.661	1.558e-49	3.598e-45
6	Smooth muscle cells	*UBC*	0.9225	0.991	0.947	3.518e-85	8.124e-81
5	Smooth muscle cells	*VWA2*	0.9186	0.259	0.044	1.580e-104	3.650e-100
5	Smooth muscle cells	*GPRIN3*	0.9178	0.233	0.055	1.178e-64	2.721e-60
6	Smooth muscle cells	*FOSL2*	0.9172	0.742	0.397	5.788e-58	1.337e-53
5	Smooth muscle cells	*NHSL2*	0.9158	0.311	0.145	5.391e-33	1.245e-28
5	Smooth muscle cells	*ADAMTS12*	0.9150	0.486	0.302	5.724e-33	1.322e-28
5	Smooth muscle cells	*ADGRB3*	0.9150	0.164	0.041	1.814e-40	4.188e-36
6	Smooth muscle cells	*ANGPT2*	0.9113	0.465	0.079	6.525e-161	1.507e-156
6	Smooth muscle cells	*IDH2*	0.9109	0.524	0.143	5.643e-107	1.303e-102
6	Smooth muscle cells	*ARPC2*	0.9080	0.771	0.388	3.193e-77	7.374e-73
6	Smooth muscle cells	*PLS3*	0.9077	0.552	0.127	2.639e-134	6.095e-130
6	Smooth muscle cells	*WNT6*	0.9068	0.445	0.072	1.100e-159	2.541e-155
6	Smooth muscle cells	*SIPA1L2*	0.9044	0.502	0.118	2.292e-119	5.293e-115
5	Smooth muscle cells	*RBPMS*	0.9039	0.634	0.476	3.198e-37	7.385e-33
5	Smooth muscle cells	*C1QTNF7*	0.8992	0.443	0.300	1.149e-23	2.653e-19
6	Smooth muscle cells	*PICALM*	0.8959	0.651	0.270	2.489e-79	5.747e-75
5	Smooth muscle cells	*ARHGAP42*	0.8958	0.439	0.301	5.111e-22	1.180e-17
6	Smooth muscle cells	*NFKBIZ*	0.8943	0.793	0.430	2.650e-55	6.120e-51
6	Smooth muscle cells	*HOPX*	0.8927	0.384	0.025	3.051e-294	7.044e-290
6	Smooth muscle cells	*LOC138843527*	0.8919	0.930	0.648	5.204e-77	1.202e-72
5	Smooth muscle cells	*TRAK1*	0.8899	0.335	0.141	4.712e-44	1.088e-39
6	Smooth muscle cells	*AVPI1*	0.8895	0.448	0.112	1.752e-98	4.046e-94
6	Smooth muscle cells	*AOC3*	0.8890	0.419	0.030	1.881e-302	4.343e-298
6	Smooth muscle cells	*LAMA5*	0.8884	0.413	0.034	3.662e-277	8.455e-273
5	Smooth muscle cells	*RORA*	0.8875	0.791	0.694	6.576e-38	1.519e-33
6	Smooth muscle cells	*HES1*	0.8858	0.570	0.162	9.367e-97	2.163e-92
6	Smooth muscle cells	*FOXC1*	0.8840	0.319	0.020	6.383e-254	1.474e-249
6	Smooth muscle cells	*SNX18*	0.8831	0.513	0.137	2.898e-106	6.693e-102
6	Smooth muscle cells	*ESYT2*	0.8801	0.539	0.221	1.079e-58	2.491e-54

Original cluster	Annotated cell type	Gene	avg_log2FC	pct.1	pct.2	Raw P value	Bonferroni-adjusted P value
5	Smooth muscle cells	*TNS1*	0.8700	0.541	0.349	2.583e-41	5.966e-37
5	Smooth muscle cells	*RNLS*	0.8689	0.257	0.075	1.153e-52	2.662e-48
6	Smooth muscle cells	*RBM24*	0.8651	0.467	0.072	4.833e-172	1.116e-167
6	Smooth muscle cells	*LRRC32*	0.8650	0.410	0.047	6.214e-209	1.435e-204
5	Smooth muscle cells	*SLC7A2*	0.8621	0.248	0.098	1.338e-32	3.089e-28
5	Smooth muscle cells	*GGTA1*	0.8616	0.390	0.174	1.187e-47	2.742e-43
5	Smooth muscle cells	*TNFRSF21*	0.8577	0.253	0.071	1.367e-57	3.156e-53
5	Smooth muscle cells	*KLF6*	0.8570	0.545	0.501	9.163e-11	2.116e-06
6	Smooth muscle cells	*CHCHD2*	0.8556	0.858	0.602	7.786e-50	1.798e-45
5	Smooth muscle cells	*PLAU*	0.8554	0.189	0.013	8.351e-157	1.928e-152
6	Smooth muscle cells	*CCND1*	0.8530	0.489	0.152	6.234e-81	1.440e-76
5	Smooth muscle cells	*SYTL2*	0.8515	0.195	0.023	1.083e-108	2.500e-104
5	Smooth muscle cells	*IFRD1*	0.8456	0.395	0.208	1.918e-35	4.429e-31
5	Smooth muscle cells	*TIMP3*	0.8441	0.832	0.752	2.623e-41	6.057e-37
6	Smooth muscle cells	*SLC7A8*	0.8433	0.384	0.053	1.134e-161	2.619e-157
6	Smooth muscle cells	*EHD4*	0.8407	0.386	0.051	3.555e-173	8.208e-169
6	Smooth muscle cells	*ANAPC7*	0.8403	0.419	0.070	3.263e-145	7.535e-141
5	Smooth muscle cells	*PELI1*	0.8361	0.352	0.245	1.834e-14	4.236e-10
6	Smooth muscle cells	*USP2*	0.8337	0.338	0.023	4.310e-253	9.952e-249
6	Smooth muscle cells	*ARHGEF7*	0.8336	0.452	0.090	4.782e-132	1.104e-127
6	Smooth muscle cells	*GLS*	0.8321	0.729	0.478	1.545e-36	3.567e-32
5	Smooth muscle cells	*ARID5B*	0.8287	0.608	0.469	2.262e-28	5.223e-24
6	Smooth muscle cells	*PDE10A*	0.8274	0.255	0.072	3.467e-45	8.006e-41
5	Smooth muscle cells	*CDC42BPA*	0.8273	0.561	0.413	2.672e-30	6.170e-26
5	Smooth muscle cells	*FGF13*	0.8272	0.193	0.070	6.670e-28	1.540e-23
6	Smooth muscle cells	*GABRA3*	0.8264	0.271	0.006	0	0
6	Smooth muscle cells	*VASN*	0.8258	0.437	0.141	1.002e-64	2.313e-60
5	Smooth muscle cells	*DACH1*	0.8240	0.357	0.266	3.739e-11	8.635e-07
6	Smooth muscle cells	*CDKN2D*	0.8239	0.432	0.113	3.293e-87	7.605e-83
6	Smooth muscle cells	*HEYL*	0.8232	0.321	0.014	0	0
6	Smooth muscle cells	*SERTAD1*	0.8228	0.900	0.589	6.886e-65	1.590e-60
6	Smooth muscle cells	*SNTB1*	0.8216	0.343	0.062	1.552e-107	3.583e-103
6	Smooth muscle cells	*TNFAIP8*	0.8212	0.596	0.192	1.103e-89	2.548e-85
5	Smooth muscle cells	*FRMD4A*	0.8170	0.352	0.188	5.012e-28	1.157e-23
5	Smooth muscle cells	*GCNT2*	0.8143	0.213	0.041	8.278e-73	1.912e-68
6	Smooth muscle cells	*PTMA*	0.8137	0.998	0.981	5.244e-127	1.211e-122
6	Smooth muscle cells	*RASAL2*	0.8119	0.504	0.125	8.633e-107	1.993e-102
6	Smooth muscle cells	*RAP1A*	0.8117	0.725	0.330	3.274e-73	7.560e-69
6	Smooth muscle cells	*CD151*	0.8090	0.524	0.138	6.697e-107	1.546e-102
6	Smooth muscle cells	*PPP1R9A*	0.8052	0.469	0.182	1.527e-49	3.527e-45

Original cluster	Annotated cell type	Gene	avg_log2FC	pct.1	pct.2	Raw P value	Bonferroni-adjusted P value
5	Smooth muscle cells	*IER5L*	0.8031	0.592	0.570	1.926e-12	4.448e-08
5	Smooth muscle cells	*FRMD3*	0.8023	0.222	0.041	1.434e-80	3.311e-76
6	Smooth muscle cells	*ANGPTL4*	0.8009	0.437	0.206	2.284e-32	5.274e-28
6	Smooth muscle cells	*LOC100353865*	0.7987	0.550	0.162	4.218e-97	9.740e-93
5	Smooth muscle cells	*ADAMTS3*	0.7986	0.204	0.112	2.219e-13	5.124e-09
6	Smooth muscle cells	*GNG2*	0.7984	0.448	0.179	2.623e-46	6.057e-42
5	Smooth muscle cells	*THBS1*	0.7980	0.288	0.142	1.685e-22	3.890e-18
6	Smooth muscle cells	*SLC25A3*	0.7975	0.819	0.467	8.276e-63	1.911e-58
5	Smooth muscle cells	*ERF*	0.7975	0.273	0.124	2.682e-29	6.193e-25
6	Smooth muscle cells	*CNN1*	0.7956	0.297	0.014	1.351e-272	3.121e-268
5	Smooth muscle cells	*PLEKHG1*	0.7953	0.202	0.076	1.183e-27	2.732e-23
5	Smooth muscle cells	*LRCH1*	0.7943	0.332	0.183	5.883e-24	1.359e-19
5	Smooth muscle cells	*ENOX1*	0.7938	0.202	0.101	2.999e-16	6.925e-12
5	Smooth muscle cells	*LOC138845366*	0.7928	0.284	0.170	5.002e-16	1.155e-11
6	Smooth muscle cells	*CSRP2*	0.7906	0.474	0.238	1.067e-31	2.464e-27
6	Smooth muscle cells	*SYNM*	0.7882	0.356	0.034	6.052e-205	1.398e-200
5	Smooth muscle cells	*FAM13A*	0.7864	0.348	0.187	1.743e-27	4.024e-23
6	Smooth muscle cells	*HSPB2*	0.7853	0.345	0.002	0	0
5	Smooth muscle cells	*SPARC*	0.7846	0.650	0.509	5.838e-33	1.348e-28
6	Smooth muscle cells	*CD55*	0.7838	0.605	0.207	1.420e-85	3.278e-81
5	Smooth muscle cells	*ATXN7L1*	0.7834	0.308	0.185	6.509e-19	1.503e-14
6	Smooth muscle cells	*FARP1*	0.7824	0.594	0.231	1.977e-70	4.566e-66
6	Smooth muscle cells	*CDKL2*	0.7817	0.400	0.074	5.393e-123	1.245e-118
5	Smooth muscle cells	*RBMS3*	0.7816	0.747	0.648	1.905e-26	4.398e-22
5	Smooth muscle cells	*NDUFA4L2*	0.7813	0.146	0.003	2.551e-202	5.892e-198
6	Smooth muscle cells	*MAT2A*	0.7810	0.707	0.437	1.186e-34	2.738e-30
5	Smooth muscle cells	*TSPAN15*	0.7777	0.377	0.203	8.796e-32	2.031e-27
5	Smooth muscle cells	*ANKRD12*	0.7739	0.577	0.511	6.919e-16	1.598e-11
6	Smooth muscle cells	*COX4I1*	0.7714	0.985	0.914	6.824e-96	1.576e-91
6	Smooth muscle cells	*C7H1orf54*	0.7694	0.474	0.093	9.520e-134	2.198e-129
6	Smooth muscle cells	*ESYT1*	0.7672	0.432	0.078	1.483e-136	3.426e-132
5	Smooth muscle cells	*FRAS1*	0.7666	0.260	0.156	3.354e-15	7.746e-11
5	Smooth muscle cells	*SFMBT2*	0.7654	0.266	0.172	1.494e-12	3.451e-08
6	Smooth muscle cells	*PACSIN2*	0.7645	0.426	0.102	1.367e-97	3.157e-93
5	Smooth muscle cells	*SPECC1*	0.7642	0.206	0.061	3.096e-41	7.148e-37
5	Smooth muscle cells	*ATRNL1*	0.7639	0.661	0.595	6.910e-15	1.596e-10
6	Smooth muscle cells	*CBLB*	0.7633	0.583	0.399	3.812e-17	8.803e-13
6	Smooth muscle cells	*NPY1R*	0.7619	0.376	0.056	6.596e-143	1.523e-138
6	Smooth muscle cells	*MICU3*	0.7570	0.651	0.288	3.934e-62	9.083e-58
6	Smooth muscle cells	*TACC1*	0.7563	0.552	0.215	9.031e-64	2.085e-59

Original cluster	Annotated cell type	Gene	avg_log2FC	pct.1	pct.2	Raw P value	Bonferroni-adjusted P value
5	Smooth muscle cells	*FGFR1*	0.7547	0.475	0.351	1.303e-21	3.009e-17
5	Smooth muscle cells	*ASXL1*	0.7543	0.444	0.333	1.399e-18	3.232e-14
6	Smooth muscle cells	*ANO1*	0.7520	0.358	0.040	1.947e-178	4.495e-174
6	Smooth muscle cells	*ADCY9*	0.7518	0.443	0.102	1.220e-103	2.818e-99
5	Smooth muscle cells	*FOXO3*	0.7517	0.421	0.326	4.117e-14	9.506e-10
6	Smooth muscle cells	*TESC*	0.7516	0.336	0.011	0	0
6	Smooth muscle cells	*NDRG2*	0.7515	0.572	0.186	2.762e-84	6.379e-80
5	Smooth muscle cells	*TASP1*	0.7509	0.384	0.306	2.679e-11	6.186e-07
6	Smooth muscle cells	*TENM1*	0.7479	0.354	0.054	1.392e-134	3.214e-130
5	Smooth muscle cells	*DTX3*	0.7474	0.741	0.666	5.019e-34	1.159e-29
6	Smooth muscle cells	*SLC20A1*	0.7468	0.397	0.125	8.232e-63	1.901e-58
6	Smooth muscle cells	*YJEFN3*	0.7465	0.703	0.367	6.939e-52	1.602e-47
6	Smooth muscle cells	*LPP*	0.7463	0.686	0.364	3.670e-50	8.475e-46
5	Smooth muscle cells	*AKAP11*	0.7452	0.321	0.182	1.792e-22	4.137e-18
5	Smooth muscle cells	*ETV6*	0.7450	0.355	0.176	1.317e-32	3.040e-28
5	Smooth muscle cells	*TCIM*	0.7445	0.135	0.047	1.527e-20	3.527e-16
6	Smooth muscle cells	*DNAJB4*	0.7442	0.404	0.151	8.470e-46	1.956e-41
6	Smooth muscle cells	*FRYL*	0.7440	0.544	0.226	1.303e-55	3.010e-51
5	Smooth muscle cells	*AUTS2*	0.7438	0.676	0.562	1.164e-25	2.687e-21
6	Smooth muscle cells	*LOC138844084*	0.7429	0.251	0.004	0	0
6	Smooth muscle cells	*ADAMTS9*	0.7406	0.260	0.044	5.828e-87	1.346e-82
6	Smooth muscle cells	*KCNQ5*	0.7402	0.330	0.042	1.742e-143	4.022e-139
5	Smooth muscle cells	*FHL1*	0.7386	0.286	0.142	1.305e-24	3.014e-20
6	Smooth muscle cells	*SEPTIN5*	0.7381	0.321	0.015	3.663e-303	8.459e-299
6	Smooth muscle cells	*PTP4A3*	0.7376	0.303	0.015	1.115e-284	2.574e-280
5	Smooth muscle cells	*JUNB*	0.7366	0.827	0.900	1.079e-32	2.493e-28
6	Smooth muscle cells	*RASSF3*	0.7338	0.432	0.091	6.891e-115	1.591e-110
5	Smooth muscle cells	*NBEA*	0.7320	0.614	0.572	2.606e-14	6.019e-10
6	Smooth muscle cells	*OXR1*	0.7318	0.731	0.450	1.830e-40	4.225e-36
6	Smooth muscle cells	*NFE2L1*	0.7295	0.581	0.246	1.068e-56	2.465e-52
5	Smooth muscle cells	*ISM1*	0.7292	0.138	0.059	6.683e-15	1.543e-10
6	Smooth muscle cells	*SYNE1*	0.7285	0.764	0.433	1.357e-53	3.134e-49
5	Smooth muscle cells	*FAT3*	0.7284	0.286	0.144	3.364e-23	7.769e-19
5	Smooth muscle cells	*MITF*	0.7283	0.740	0.619	9.420e-31	2.175e-26
5	Smooth muscle cells	*NCALD*	0.7269	0.197	0.041	4.972e-61	1.148e-56
6	Smooth muscle cells	*EEF2*	0.7267	0.987	0.878	7.568e-74	1.748e-69
5	Smooth muscle cells	*PITPNB*	0.7262	0.455	0.330	1.723e-21	3.979e-17
5	Smooth muscle cells	*TBC1D4*	0.7255	0.270	0.127	2.073e-25	4.787e-21
6	Smooth muscle cells	*NTF3*	0.7244	0.271	0.005	0	0
5	Smooth muscle cells	*KLHL29*	0.7243	0.191	0.056	1.499e-39	3.460e-35

Original cluster	Annotated cell type	Gene	avg_log2FC	pct.1	pct.2	Raw P value	Bonferroni-adjusted P value
5	Smooth muscle cells	*FILIP1L*	0.7232	0.335	0.236	3.654e-13	8.438e-09
6	Smooth muscle cells	*EHD2*	0.7217	0.487	0.122	7.555e-102	1.745e-97
5	Smooth muscle cells	*KALRN*	0.7205	0.262	0.145	1.738e-17	4.013e-13
5	Smooth muscle cells	*STAT3*	0.7204	0.572	0.477	7.749e-21	1.789e-16
6	Smooth muscle cells	*PPIA*	0.7203	0.790	0.463	1.165e-53	2.691e-49
6	Smooth muscle cells	*MYO1D*	0.7187	0.352	0.054	2.154e-132	4.974e-128
5	Smooth muscle cells	*SEPTIN7*	0.7179	0.741	0.691	5.045e-32	1.165e-27
5	Smooth muscle cells	*SH3RF1*	0.7175	0.284	0.189	8.701e-12	2.009e-07
6	Smooth muscle cells	*PTMS*	0.7170	0.860	0.602	1.401e-49	3.235e-45
6	Smooth muscle cells	*LOC100343872*	0.7168	0.395	0.062	7.073e-142	1.633e-137
6	Smooth muscle cells	*COL13A1*	0.7159	0.365	0.072	1.109e-102	2.560e-98
6	Smooth muscle cells	*PPM1L*	0.7140	0.620	0.315	1.106e-39	2.554e-35
6	Smooth muscle cells	*HSPB1*	0.7135	0.976	0.863	1.533e-54	3.540e-50
5	Smooth muscle cells	*NFIL3*	0.7111	0.284	0.143	5.629e-24	1.300e-19
6	Smooth muscle cells	*ATP5MC2*	0.7094	0.943	0.747	1.792e-58	4.138e-54
6	Smooth muscle cells	*HPSE2*	0.7085	0.155	0.023	4.319e-61	9.973e-57
5	Smooth muscle cells	*CREB5*	0.7085	0.255	0.172	2.658e-10	6.139e-06
6	Smooth muscle cells	*PRRX1*	0.7074	0.812	0.525	2.816e-44	6.502e-40
5	Smooth muscle cells	*ZFP36*	0.7041	0.743	0.744	7.531e-17	1.739e-12
6	Smooth muscle cells	*MYOM1*	0.7041	0.336	0.042	1.821e-153	4.206e-149
5	Smooth muscle cells	*MYH9*	0.7021	0.373	0.209	4.690e-28	1.083e-23
6	Smooth muscle cells	*COL24A1*	0.7005	0.245	0.046	1.101e-72	2.542e-68
6	Smooth muscle cells	*CARHSP1*	0.6984	0.421	0.120	9.040e-76	2.087e-71
6	Smooth muscle cells	*NOLC1*	0.6977	0.483	0.182	7.344e-55	1.696e-50
5	Smooth muscle cells	*SLC40A1*	0.6974	0.475	0.399	2.176e-12	5.024e-08
6	Smooth muscle cells	*ACTN4*	0.6963	0.535	0.184	1.771e-72	4.089e-68
5	Smooth muscle cells	*ARHGEF12*	0.6962	0.415	0.298	2.124e-17	4.905e-13
6	Smooth muscle cells	*LOC100357092*	0.6957	0.262	0.001	0	0
5	Smooth muscle cells	*NFAT5*	0.6947	0.563	0.480	4.697e-17	1.085e-12
5	Smooth muscle cells	*RFTN1*	0.6940	0.293	0.138	4.972e-29	1.148e-24
6	Smooth muscle cells	*COL4A3*	0.6927	0.199	0.013	4.051e-152	9.354e-148
6	Smooth muscle cells	*YWHAZ*	0.6915	0.878	0.634	1.167e-51	2.694e-47
5	Smooth muscle cells	*MAML2*	0.6905	0.572	0.468	6.353e-19	1.467e-14
5	Smooth muscle cells	*TRPC6*	0.6879	0.135	0.041	2.468e-25	5.699e-21
6	Smooth muscle cells	*ITPRID2*	0.6877	0.487	0.137	4.916e-85	1.135e-80
5	Smooth muscle cells	*PPP2R2A*	0.6848	0.506	0.387	2.915e-20	6.731e-16
6	Smooth muscle cells	*MAGI1*	0.6848	0.465	0.117	6.274e-93	1.449e-88
6	Smooth muscle cells	*RALGAPA2*	0.6829	0.467	0.182	4.375e-50	1.010e-45
5	Smooth muscle cells	*CPED1*	0.6814	0.428	0.308	6.590e-15	1.522e-10
5	Smooth muscle cells	*COL4A2*	0.6812	0.248	0.094	2.111e-33	4.874e-29

Original cluster	Annotated cell type	Gene	avg_log2FC	pct.1	pct.2	Raw P value	Bonferroni-adjusted P value
6	Smooth muscle cells	*ACTB*	0.6808	0.996	0.970	6.632e-74	1.532e-69
6	Smooth muscle cells	*ARHGAP10*	0.6781	0.600	0.320	4.706e-37	1.087e-32
6	Smooth muscle cells	*CSPG4*	0.6759	0.297	0.023	7.011e-203	1.619e-198
6	Smooth muscle cells	*ANKRD10*	0.6747	0.515	0.201	5.414e-54	1.250e-49
5	Smooth muscle cells	*DNAJC6*	0.6745	0.164	0.031	6.977e-58	1.611e-53
6	Smooth muscle cells	*KCNMB1*	0.6743	0.308	0.008	0	0
5	Smooth muscle cells	*ENPP2*	0.6743	0.342	0.268	2.769e-09	6.395e-05
5	Smooth muscle cells	*ARHGAP18*	0.6705	0.219	0.087	2.132e-27	4.923e-23
6	Smooth muscle cells	*CSNK1A1*	0.6700	0.675	0.409	2.762e-38	6.377e-34
6	Smooth muscle cells	*CNN3*	0.6694	0.902	0.679	7.435e-47	1.717e-42
5	Smooth muscle cells	*REV1*	0.6686	0.330	0.238	1.044e-12	2.412e-08
6	Smooth muscle cells	*AMPH*	0.6682	0.271	0.018	7.635e-203	1.763e-198
6	Smooth muscle cells	*ESRRG*	0.6680	0.378	0.100	4.130e-71	9.538e-67
5	Smooth muscle cells	*ANTXR1*	0.6659	0.503	0.399	1.442e-16	3.330e-12
6	Smooth muscle cells	*SORBS3*	0.6651	0.424	0.095	1.934e-100	4.466e-96
5	Smooth muscle cells	*BASP1*	0.6646	0.177	0.043	6.417e-46	1.482e-41
6	Smooth muscle cells	*SYNE2*	0.6630	0.535	0.200	7.243e-61	1.673e-56
5	Smooth muscle cells	*SPECC1L*	0.6624	0.342	0.203	1.460e-22	3.371e-18
5	Smooth muscle cells	*COL6A3*	0.6623	0.251	0.119	4.855e-23	1.121e-18
5	Smooth muscle cells	*BACH2*	0.6586	0.319	0.193	8.064e-17	1.862e-12
6	Smooth muscle cells	*SMTN*	0.6568	0.400	0.099	6.304e-84	1.456e-79
5	Smooth muscle cells	*AJUBA*	0.6544	0.197	0.062	6.991e-37	1.614e-32
6	Smooth muscle cells	*NBEAL1*	0.6537	0.603	0.339	3.016e-33	6.965e-29
5	Smooth muscle cells	*APBB2*	0.6535	0.286	0.161	6.228e-19	1.438e-14
6	Smooth muscle cells	*ARID5A*	0.6533	0.334	0.044	5.203e-145	1.202e-140
6	Smooth muscle cells	*CPEB4*	0.6523	0.389	0.115	1.232e-64	2.845e-60
6	Smooth muscle cells	*TXN*	0.6516	0.934	0.772	5.567e-41	1.286e-36
6	Smooth muscle cells	*ACTG2*	0.6510	0.290	0.026	2.834e-172	6.544e-168
6	Smooth muscle cells	*LAPTM4A*	0.6504	0.830	0.646	7.803e-24	1.802e-19
5	Smooth muscle cells	*DYRK1A*	0.6498	0.322	0.256	2.388e-07	5.515e-03
6	Smooth muscle cells	*TSPAN7*	0.6493	0.539	0.219	4.870e-53	1.125e-48
6	Smooth muscle cells	*IL1RAPL2*	0.6488	0.164	0.020	1.293e-76	2.987e-72
6	Smooth muscle cells	*KEF51-p11*	0.6479	0.998	0.993	1.191e-85	2.750e-81
6	Smooth muscle cells	*KIF5B*	0.6475	0.607	0.292	4.068e-46	9.393e-42
5	Smooth muscle cells	*CDK6*	0.6468	0.410	0.298	7.906e-16	1.826e-11
6	Smooth muscle cells	*GMDS*	0.6466	0.434	0.139	1.848e-63	4.267e-59
5	Smooth muscle cells	*SLC30A4*	0.6457	0.209	0.127	3.755e-11	8.670e-07
6	Smooth muscle cells	*RASL12*	0.6445	0.288	0.014	3.879e-263	8.958e-259
6	Smooth muscle cells	*PRXL2A*	0.6444	0.476	0.168	1.053e-59	2.432e-55
5	Smooth muscle cells	*SH2D3C*	0.6442	0.209	0.085	3.726e-26	8.603e-22

Original cluster	Annotated cell type	Gene	avg_log2FC	pct.1	pct.2	Raw P value	Bonferroni-adjusted P value
5	Smooth muscle cells	*DENND2B*	0.6429	0.308	0.168	2.006e-22	4.632e-18
6	Smooth muscle cells	*PELI2*	0.6420	0.517	0.217	6.551e-49	1.513e-44
5	Smooth muscle cells	*ELF2*	0.6416	0.446	0.398	3.142e-08	7.256e-04
6	Smooth muscle cells	*SMAP2*	0.6401	0.336	0.051	1.613e-128	3.724e-124
6	Smooth muscle cells	*SRSF2*	0.6400	0.908	0.685	5.660e-43	1.307e-38
5	Smooth muscle cells	*C1QTNF2*	0.6387	0.246	0.103	1.675e-29	3.869e-25
6	Smooth muscle cells	*CST3*	0.6386	0.716	0.360	1.194e-51	2.758e-47
6	Smooth muscle cells	*IL13RA1*	0.6383	0.349	0.060	1.150e-114	2.656e-110
5	Smooth muscle cells	*INPP5A*	0.6377	0.233	0.082	1.146e-37	2.646e-33
6	Smooth muscle cells	*HEY2*	0.6368	0.275	0.016	7.567e-230	1.747e-225
5	Smooth muscle cells	*LOC138849658*	0.6362	0.204	0.061	6.490e-41	1.499e-36
6	Smooth muscle cells	*CCNG1*	0.6354	0.891	0.703	6.232e-38	1.439e-33
6	Smooth muscle cells	*AZIN1*	0.6345	0.627	0.309	3.869e-45	8.934e-41
5	Smooth muscle cells	*PPFIBP1*	0.6335	0.299	0.166	1.812e-20	4.185e-16
5	Smooth muscle cells	*STK3*	0.6331	0.477	0.396	4.016e-14	9.274e-10
5	Smooth muscle cells	*MOB3B*	0.6329	0.255	0.130	3.622e-20	8.365e-16
6	Smooth muscle cells	*RIGI*	0.6325	0.493	0.225	4.457e-39	1.029e-34
6	Smooth muscle cells	*RELL1*	0.6318	0.356	0.072	2.912e-97	6.723e-93
5	Smooth muscle cells	*LIMS1*	0.6315	0.412	0.298	1.458e-16	3.367e-12
6	Smooth muscle cells	*PXDNL*	0.6307	0.240	0.027	3.203e-122	7.396e-118
5	Smooth muscle cells	*SERPINI1*	0.6304	0.166	0.054	5.999e-29	1.385e-24
5	Smooth muscle cells	*LOC100349021*	0.6302	0.483	0.423	3.688e-10	8.517e-06
6	Smooth muscle cells	*PHLDA1*	0.6287	0.271	0.072	1.295e-51	2.991e-47
6	Smooth muscle cells	*SLC14A1*	0.6272	0.172	0.009	9.728e-158	2.246e-153
5	Smooth muscle cells	*RGS3*	0.6258	0.277	0.210	2.169e-07	5.008e-03
5	Smooth muscle cells	*CEP85L*	0.6255	0.337	0.216	2.596e-17	5.994e-13
5	Smooth muscle cells	*BAMBI*	0.6254	0.160	0.060	2.843e-22	6.565e-18
5	Smooth muscle cells	*TRPC1*	0.6252	0.310	0.204	1.860e-13	4.296e-09
6	Smooth muscle cells	*SHC4*	0.6233	0.260	0.010	3.545e-272	8.185e-268
6	Smooth muscle cells	*CHST8*	0.6219	0.218	0.004	9.160e-300	2.115e-295
6	Smooth muscle cells	*RNF150*	0.6218	0.303	0.086	8.110e-52	1.873e-47
6	Smooth muscle cells	*SCHIP1*	0.6215	0.360	0.073	1.248e-97	2.882e-93
6	Smooth muscle cells	*TGFB2*	0.6200	0.299	0.080	7.567e-55	1.747e-50
6	Smooth muscle cells	*ARVCF*	0.6199	0.301	0.018	9.043e-240	2.088e-235
5	Smooth muscle cells	*TRA2B*	0.6195	0.572	0.491	1.039e-17	2.399e-13
6	Smooth muscle cells	*S1PR3*	0.6188	0.192	0.002	1.800e-294	4.157e-290
6	Smooth muscle cells	*UBE2E2*	0.6187	0.417	0.149	1.342e-49	3.098e-45
6	Smooth muscle cells	*MYO10*	0.6181	0.306	0.049	8.508e-108	1.965e-103
6	Smooth muscle cells	*DUSP2*	0.6175	0.408	0.157	2.982e-39	6.886e-35
6	Smooth muscle cells	*ELMO1*	0.6170	0.352	0.103	2.221e-57	5.129e-53

Original cluster	Annotated cell type	Gene	avg_log2FC	pct.1	pct.2	Raw P value	Bonferroni-adjusted P value
6	Smooth muscle cells	*CAMTA1*	0.6147	0.624	0.297	3.543e-47	8.182e-43
6	Smooth muscle cells	*ETV1*	0.6130	0.255	0.030	2.638e-123	6.091e-119
5	Smooth muscle cells	*LDAF1*	0.6124	0.259	0.137	1.059e-19	2.445e-15
6	Smooth muscle cells	*AMD1*	0.6122	0.675	0.394	1.361e-31	3.143e-27
6	Smooth muscle cells	*IRS2*	0.6110	0.570	0.324	1.190e-28	2.749e-24
6	Smooth muscle cells	*FBXL22*	0.6108	0.308	0.037	1.991e-145	4.598e-141
5	Smooth muscle cells	*AGTR1*	0.6099	0.148	0.060	6.679e-18	1.542e-13
6	Smooth muscle cells	*CKB*	0.6098	0.384	0.121	7.273e-56	1.680e-51
5	Smooth muscle cells	*CYTH3*	0.6095	0.224	0.101	3.297e-23	7.612e-19
5	Smooth muscle cells	*LOC138846057*	0.6078	0.138	0.019	8.031e-66	1.854e-61
5	Smooth muscle cells	*IL6ST*	0.6069	0.494	0.360	2.175e-20	5.022e-16
5	Smooth muscle cells	*MAML3*	0.6068	0.348	0.235	1.111e-13	2.564e-09
6	Smooth muscle cells	*WWP2*	0.6061	0.321	0.057	7.728e-103	1.785e-98
6	Smooth muscle cells	*TMEM141*	0.6061	0.391	0.114	6.614e-65	1.527e-60
5	Smooth muscle cells	*ROCK2*	0.6055	0.423	0.343	4.092e-12	9.449e-08
6	Smooth muscle cells	*EIF4A1*	0.6053	0.876	0.647	7.581e-39	1.751e-34
5	Smooth muscle cells	*BTG1*	0.6042	0.707	0.662	2.578e-19	5.954e-15
5	Smooth muscle cells	*RNF19B*	0.6038	0.255	0.192	2.522e-07	5.825e-03
5	Smooth muscle cells	*UBR1*	0.6037	0.357	0.274	5.758e-11	1.330e-06
6	Smooth muscle cells	*LURAP1L*	0.6033	0.393	0.124	9.490e-57	2.191e-52
6	Smooth muscle cells	*DUSP8*	0.6023	0.216	0.005	2.233e-277	5.157e-273
6	Smooth muscle cells	*PPP1R14A*	0.6020	0.336	0.062	1.669e-100	3.854e-96
6	Smooth muscle cells	*IQGAP1*	0.6016	0.491	0.204	2.616e-47	6.042e-43
6	Smooth muscle cells	*RGS7BP*	0.6011	0.253	0.014	7.118e-213	1.644e-208
5	Smooth muscle cells	*SPAG9*	0.6005	0.388	0.297	8.941e-12	2.065e-07
6	Smooth muscle cells	*ADRA1A*	0.5996	0.290	0.050	1.509e-93	3.486e-89
6	Smooth muscle cells	*CYBA*	0.5974	0.483	0.169	3.161e-60	7.298e-56
6	Smooth muscle cells	*CSK*	0.5971	0.424	0.150	3.298e-52	7.616e-48
5	Smooth muscle cells	*PCOLCE2*	0.5964	0.151	0.039	2.291e-36	5.290e-32
5	Smooth muscle cells	*DENND5A*	0.5959	0.332	0.254	1.546e-10	3.571e-06
6	Smooth muscle cells	*CD93*	0.5957	0.301	0.042	4.091e-120	9.447e-116
5	Smooth muscle cells	*SLC12A2*	0.5934	0.189	0.095	5.022e-15	1.160e-10
5	Smooth muscle cells	*ROR2*	0.5915	0.255	0.163	6.129e-12	1.415e-07
6	Smooth muscle cells	*AMIGO2*	0.5913	0.297	0.030	4.341e-161	1.002e-156
6	Smooth muscle cells	*PDZRN4*	0.5901	0.271	0.026	9.237e-152	2.133e-147
6	Smooth muscle cells	*CLDN5*	0.5896	0.332	0.042	1.553e-146	3.585e-142
6	Smooth muscle cells	*SUSD5*	0.5888	0.249	0.001	0	0
6	Smooth muscle cells	*GPRC5A*	0.5883	0.210	0.039	2.568e-62	5.929e-58
5	Smooth muscle cells	*SEPTIN11*	0.5882	0.282	0.155	2.449e-20	5.654e-16
5	Smooth muscle cells	*LRRC36*	0.5868	0.171	0.070	2.068e-20	4.774e-16

Original cluster	Annotated cell type	Gene	avg_log2FC	pct.1	pct.2	Raw P value	Bonferroni-adjusted P value
6	Smooth muscle cells	*EIF5*	0.5868	0.790	0.554	5.938e-33	1.371e-28
6	Smooth muscle cells	*ATP1B2*	0.5865	0.365	0.093	1.690e-72	3.903e-68
6	Smooth muscle cells	*STK38L*	0.5855	0.286	0.069	3.800e-62	8.776e-58
6	Smooth muscle cells	*NWD2*	0.5845	0.133	0.021	1.641e-46	3.789e-42
6	Smooth muscle cells	*SH3BGRL*	0.5826	0.648	0.336	4.336e-41	1.001e-36
5	Smooth muscle cells	*PLAC9*	0.5824	0.146	0.017	3.556e-80	8.211e-76
6	Smooth muscle cells	*LGALSL*	0.5819	0.284	0.024	4.841e-182	1.118e-177
6	Smooth muscle cells	*BRD2*	0.5817	0.679	0.432	1.668e-30	3.853e-26
6	Smooth muscle cells	*ATP2B1*	0.5815	0.697	0.390	1.022e-39	2.359e-35
6	Smooth muscle cells	*TGFB1I1*	0.5801	0.500	0.214	1.231e-42	2.843e-38
6	Smooth muscle cells	*LTBP1*	0.5799	0.697	0.441	8.213e-26	1.896e-21
6	Smooth muscle cells	*PURB*	0.5799	0.572	0.276	5.900e-40	1.362e-35
6	Smooth muscle cells	*ASB14*	0.5798	0.242	0.024	4.970e-135	1.148e-130
6	Smooth muscle cells	*MAFF*	0.5795	0.290	0.076	7.748e-57	1.789e-52
5	Smooth muscle cells	*TLE4*	0.5784	0.424	0.375	1.271e-07	2.935e-03
6	Smooth muscle cells	*XKR4*	0.5775	0.157	0.010	1.540e-118	3.557e-114
5	Smooth muscle cells	*YWHAH*	0.5765	0.171	0.082	2.791e-15	6.445e-11
6	Smooth muscle cells	*LOC100340711*	0.5759	0.245	0.026	2.513e-128	5.803e-124
5	Smooth muscle cells	*SPRED1*	0.5745	0.288	0.226	5.591e-07	1.291e-02
6	Smooth muscle cells	*DOCK8*	0.5745	0.306	0.035	1.738e-150	4.013e-146
5	Smooth muscle cells	*GLUL*	0.5735	0.448	0.418	7.726e-07	1.784e-02
5	Smooth muscle cells	*ASAP1*	0.5727	0.412	0.312	1.455e-13	3.360e-09
6	Smooth muscle cells	*BRIP1*	0.5727	0.288	0.047	1.337e-98	3.089e-94
5	Smooth muscle cells	*LOC103348141*	0.5716	0.424	0.347	4.990e-10	1.152e-05
6	Smooth muscle cells	*ITIH5*	0.5715	0.343	0.089	5.535e-68	1.278e-63
5	Smooth muscle cells	*ARHGAP32*	0.5713	0.361	0.308	8.873e-07	2.049e-02
5	Smooth muscle cells	*EPC1*	0.5710	0.506	0.451	3.180e-10	7.342e-06
6	Smooth muscle cells	*EIF3A*	0.5706	0.596	0.301	1.053e-36	2.432e-32
6	Smooth muscle cells	*VIPR2*	0.5706	0.249	0.039	3.323e-91	7.673e-87
5	Smooth muscle cells	*SETBP1*	0.5701	0.483	0.400	3.234e-12	7.469e-08
6	Smooth muscle cells	*PPP2CA*	0.5696	0.480	0.197	5.009e-45	1.157e-40
5	Smooth muscle cells	*ANGPTL2*	0.5687	0.182	0.063	2.600e-28	6.004e-24
5	Smooth muscle cells	*LAMB2*	0.5676	0.279	0.179	2.045e-13	4.721e-09
5	Smooth muscle cells	*LONRF1*	0.5671	0.268	0.175	8.573e-12	1.980e-07
5	Smooth muscle cells	*SPIRE1*	0.5661	0.262	0.170	1.619e-11	3.740e-07
6	Smooth muscle cells	*CFL1*	0.5657	0.777	0.502	1.384e-34	3.197e-30
5	Smooth muscle cells	*ASPN*	0.5652	0.148	0.052	9.865e-22	2.278e-17
6	Smooth muscle cells	*REXO5*	0.5648	0.404	0.164	1.461e-37	3.373e-33
6	Smooth muscle cells	*NGF*	0.5638	0.273	0.042	9.629e-98	2.224e-93
5	Smooth muscle cells	*SBF2*	0.5624	0.466	0.423	8.907e-08	2.057e-03

Original cluster	Annotated cell type	Gene	avg_log2FC	pct.1	pct.2	Raw P value	Bonferroni-adjusted P value
5	Smooth muscle cells	*ZSWIM6*	0.5609	0.444	0.371	2.738e-10	6.323e-06
5	Smooth muscle cells	*ETS1*	0.5603	0.259	0.115	1.014e-25	2.342e-21
6	Smooth muscle cells	*GNG12*	0.5597	0.356	0.098	6.133e-64	1.416e-59
6	Smooth muscle cells	*MYO1C*	0.5593	0.367	0.097	1.001e-69	2.312e-65
6	Smooth muscle cells	*MSN*	0.5583	0.515	0.193	1.057e-55	2.441e-51
5	Smooth muscle cells	*GAB1*	0.5577	0.357	0.251	4.772e-13	1.102e-08
6	Smooth muscle cells	*MPRIP*	0.5559	0.419	0.158	1.861e-45	4.298e-41
6	Smooth muscle cells	*ATP5F1B*	0.5558	0.651	0.357	7.758e-36	1.791e-31
5	Smooth muscle cells	*PLA2G7*	0.5557	0.257	0.157	2.890e-13	6.675e-09
5	Smooth muscle cells	*CDK14*	0.5555	0.361	0.271	7.037e-11	1.625e-06
6	Smooth muscle cells	*KCNMB4*	0.5550	0.319	0.062	8.883e-89	2.051e-84
5	Smooth muscle cells	*MLLT3*	0.5549	0.448	0.370	1.599e-09	3.691e-05
6	Smooth muscle cells	*KANK2*	0.5542	0.572	0.281	4.552e-38	1.051e-33
5	Smooth muscle cells	*BRAF*	0.5541	0.426	0.330	5.688e-13	1.313e-08
6	Smooth muscle cells	*TLN1*	0.5540	0.620	0.321	6.816e-39	1.574e-34
6	Smooth muscle cells	*SERTAD3*	0.5532	0.509	0.223	8.820e-41	2.037e-36
6	Smooth muscle cells	*MAP3K2*	0.5529	0.570	0.354	1.868e-21	4.313e-17
5	Smooth muscle cells	*ODAD3*	0.5518	0.128	0.010	1.113e-93	2.570e-89
5	Smooth muscle cells	*PARD3*	0.5515	0.295	0.218	4.264e-09	9.847e-05
5	Smooth muscle cells	*PHLPP1*	0.5513	0.239	0.143	4.063e-12	9.382e-08
6	Smooth muscle cells	*PAIP2B*	0.5512	0.306	0.132	3.671e-25	8.478e-21
5	Smooth muscle cells	*C1QTNF5*	0.5483	0.217	0.095	7.814e-23	1.804e-18
6	Smooth muscle cells	*MAGI3*	0.5475	0.448	0.262	2.916e-18	6.733e-14
5	Smooth muscle cells	*LPIN2*	0.5467	0.219	0.152	7.934e-08	1.832e-03
6	Smooth muscle cells	*PTK2*	0.5464	0.474	0.228	5.959e-32	1.376e-27
6	Smooth muscle cells	*UBE2S*	0.5453	0.751	0.485	5.324e-28	1.229e-23
6	Smooth muscle cells	*NFIB*	0.5452	0.646	0.352	8.931e-34	2.062e-29
5	Smooth muscle cells	*LOC100344919*	0.5441	0.126	0.012	1.850e-83	4.273e-79
6	Smooth muscle cells	*MED12L*	0.5435	0.229	0.034	1.459e-86	3.369e-82
6	Smooth muscle cells	*COX5A*	0.5428	0.675	0.389	1.998e-30	4.613e-26
5	Smooth muscle cells	*LOC138845174*	0.5418	0.193	0.100	3.167e-14	7.313e-10
5	Smooth muscle cells	*FAS*	0.5407	0.164	0.049	4.637e-32	1.071e-27
5	Smooth muscle cells	*PPP1R2*	0.5406	0.390	0.338	1.216e-07	2.808e-03
6	Smooth muscle cells	*VDAC2*	0.5392	0.773	0.483	6.419e-36	1.482e-31
5	Smooth muscle cells	*DDR2*	0.5385	0.474	0.426	3.959e-08	9.141e-04
6	Smooth muscle cells	*FERMT2*	0.5383	0.629	0.323	2.402e-36	5.547e-32
6	Smooth muscle cells	*AKAP5*	0.5370	0.229	0.016	1.215e-168	2.805e-164
5	Smooth muscle cells	*TRIQK*	0.5363	0.257	0.184	3.214e-08	7.422e-04
5	Smooth muscle cells	*PDE8A*	0.5356	0.146	0.037	3.814e-36	8.807e-32
6	Smooth muscle cells	*CRTAP*	0.5356	0.445	0.178	3.133e-42	7.235e-38

Original cluster	Annotated cell type	Gene	avg_log2FC	pct.1	pct.2	Raw P value	Bonferroni-adjusted P value
6	Smooth muscle cells	*SLC25A24*	0.5355	0.408	0.141	2.878e-50	6.646e-46
6	Smooth muscle cells	*ITGA3*	0.5355	0.229	0.016	8.983e-172	2.074e-167
6	Smooth muscle cells	*LCLAT1*	0.5332	0.382	0.152	7.814e-37	1.804e-32
6	Smooth muscle cells	*CHST9*	0.5329	0.162	0.011	1.312e-120	3.030e-116
6	Smooth muscle cells	*YBX1*	0.5324	0.906	0.685	1.338e-36	3.089e-32
6	Smooth muscle cells	*EPHA2*	0.5322	0.190	0.018	3.530e-113	8.152e-109
6	Smooth muscle cells	*GPATCH11*	0.5319	0.345	0.117	1.326e-44	3.062e-40
5	Smooth muscle cells	*LOC103345192*	0.5316	0.510	0.451	6.232e-13	1.439e-08
5	Smooth muscle cells	*RIPOR2*	0.5301	0.237	0.112	2.395e-21	5.530e-17
6	Smooth muscle cells	*NHS*	0.5300	0.299	0.098	7.742e-40	1.788e-35
5	Smooth muscle cells	*WDR47*	0.5299	0.208	0.111	9.586e-15	2.214e-10
6	Smooth muscle cells	*KDM6B*	0.5296	0.524	0.271	2.947e-30	6.806e-26
5	Smooth muscle cells	*UBXN6*	0.5270	0.368	0.313	2.201e-08	5.083e-04
6	Smooth muscle cells	*CLASP2*	0.5270	0.509	0.331	2.650e-15	6.120e-11
5	Smooth muscle cells	*RRBP1*	0.5266	0.373	0.292	3.524e-10	8.137e-06
6	Smooth muscle cells	*CYCS*	0.5253	0.334	0.096	5.771e-56	1.333e-51
5	Smooth muscle cells	*RNF19A*	0.5245	0.395	0.331	2.680e-08	6.188e-04
6	Smooth muscle cells	*DDX3X*	0.5236	0.808	0.586	8.732e-29	2.016e-24
5	Smooth muscle cells	*NAP1L1*	0.5235	0.761	0.744	1.340e-19	3.093e-15
5	Smooth muscle cells	*MMD*	0.5233	0.146	0.091	2.014e-06	4.650e-02
6	Smooth muscle cells	*LOC108177235*	0.5223	0.915	0.685	1.380e-39	3.186e-35
6	Smooth muscle cells	*TRPC4*	0.5219	0.421	0.187	7.102e-32	1.640e-27
6	Smooth muscle cells	*TOMM20*	0.5218	0.657	0.397	4.206e-28	9.712e-24
6	Smooth muscle cells	*CLIC4*	0.5213	0.635	0.348	1.055e-34	2.436e-30
5	Smooth muscle cells	*LAMC1*	0.5209	0.375	0.299	2.314e-09	5.343e-05
5	Smooth muscle cells	*CTNNA1*	0.5208	0.415	0.346	5.689e-10	1.314e-05
5	Smooth muscle cells	*DDX11*	0.5206	0.186	0.097	1.228e-13	2.835e-09
5	Smooth muscle cells	*LOC138847739*	0.5204	0.377	0.265	2.607e-15	6.020e-11
6	Smooth muscle cells	*KLF9*	0.5204	0.496	0.260	2.337e-26	5.398e-22
5	Smooth muscle cells	*RUNX1T1*	0.5201	0.601	0.572	1.119e-08	2.584e-04
5	Smooth muscle cells	*YAP1*	0.5196	0.464	0.409	6.178e-09	1.427e-04
6	Smooth muscle cells	*SGCD*	0.5194	0.430	0.246	5.784e-17	1.336e-12
5	Smooth muscle cells	*PIK3CA*	0.5193	0.361	0.312	1.170e-06	2.702e-02
5	Smooth muscle cells	*LACTB*	0.5193	0.228	0.126	4.392e-15	1.014e-10
6	Smooth muscle cells	*ATF4*	0.5191	0.841	0.631	2.033e-29	4.694e-25
5	Smooth muscle cells	*CAMSAP2*	0.5173	0.353	0.282	8.615e-09	1.989e-04
5	Smooth muscle cells	*KDM6A*	0.5168	0.444	0.383	6.873e-09	1.587e-04
5	Smooth muscle cells	*PATL1*	0.5165	0.189	0.116	4.188e-09	9.671e-05
5	Smooth muscle cells	*FUOM*	0.5160	0.353	0.269	1.091e-10	2.519e-06
5	Smooth muscle cells	*DUSP16*	0.5158	0.235	0.156	2.039e-09	4.709e-05

Original cluster	Annotated cell type	Gene	avg_log2FC	pct.1	pct.2	Raw P value	Bonferroni-adjusted P value
5	Smooth muscle cells	*KPNA4*	0.5157	0.335	0.244	1.049e-11	2.423e-07
5	Smooth muscle cells	*MAST4*	0.5155	0.377	0.314	1.626e-07	3.755e-03
6	Smooth muscle cells	*TP53INP2*	0.5150	0.216	0.016	1.138e-150	2.628e-146
5	Smooth muscle cells	*DST*	0.5146	0.616	0.578	8.495e-11	1.962e-06
6	Smooth muscle cells	*BHLHE40*	0.5136	0.428	0.158	3.757e-46	8.675e-42
6	Smooth muscle cells	*TPST2*	0.5134	0.358	0.096	3.828e-65	8.838e-61
5	Smooth muscle cells	*PDE4A*	0.5125	0.157	0.040	1.289e-37	2.976e-33
5	Smooth muscle cells	*PDZD2*	0.5121	0.173	0.079	1.354e-16	3.127e-12
5	Smooth muscle cells	*UBR3*	0.5113	0.399	0.328	5.738e-10	1.325e-05
5	Smooth muscle cells	*ADAP2*	0.5110	0.158	0.054	1.413e-25	3.262e-21
6	Smooth muscle cells	*CSNK1E*	0.5101	0.410	0.158	2.982e-41	6.886e-37
5	Smooth muscle cells	*MRTFA*	0.5099	0.255	0.151	2.546e-14	5.879e-10
6	Smooth muscle cells	*HSPD1*	0.5099	0.638	0.421	1.609e-21	3.715e-17
6	Smooth muscle cells	*MGST3*	0.5098	0.725	0.455	2.245e-29	5.183e-25
6	Smooth muscle cells	*EIF4G2*	0.5093	0.801	0.583	1.706e-28	3.939e-24
6	Smooth muscle cells	*MSI2*	0.5091	0.467	0.220	2.660e-33	6.142e-29
5	Smooth muscle cells	*RERG*	0.5088	0.304	0.191	3.716e-13	8.582e-09
5	Smooth muscle cells	*LOC138848505*	0.5083	0.155	0.078	6.408e-12	1.480e-07
5	Smooth muscle cells	*NUFIP2*	0.5079	0.488	0.432	7.560e-10	1.746e-05
6	Smooth muscle cells	*GLRX2*	0.5075	0.306	0.097	1.759e-44	4.061e-40
6	Smooth muscle cells	*NAV2*	0.5049	0.290	0.080	3.738e-52	8.633e-48
6	Smooth muscle cells	*SLC4A7*	0.5048	0.408	0.159	5.038e-41	1.163e-36
6	Smooth muscle cells	*GNB4*	0.5037	0.445	0.172	8.697e-45	2.008e-40
5	Smooth muscle cells	*PDGFRL*	0.5034	0.182	0.083	4.125e-17	9.525e-13
6	Smooth muscle cells	*CLTB*	0.5029	0.500	0.220	1.656e-38	3.824e-34
5	Smooth muscle cells	*PARD3B*	0.5029	0.648	0.571	3.434e-14	7.929e-10
5	Smooth muscle cells	*MAMLD1*	0.5017	0.177	0.077	3.051e-18	7.045e-14
5	Smooth muscle cells	*MAFK*	0.5005	0.219	0.117	1.469e-15	3.392e-11
6	Smooth muscle cells	*SOD3*	0.5001	0.544	0.273	4.889e-32	1.129e-27
6	Smooth muscle cells	*STXBP6*	0.5000	0.330	0.110	1.426e-42	3.292e-38
5	Smooth muscle cells	*TENT4B*	0.4999	0.199	0.130	6.602e-08	1.524e-03
6	Smooth muscle cells	*PFN1*	0.4997	0.841	0.547	1.758e-43	4.059e-39
5	Smooth muscle cells	*CLASP1*	0.4991	0.302	0.243	4.729e-07	1.092e-02
5	Smooth muscle cells	*SSBP2*	0.4987	0.492	0.450	3.425e-08	7.909e-04
6	Smooth muscle cells	*SDHA*	0.4986	0.513	0.252	6.497e-31	1.500e-26
6	Smooth muscle cells	*NDUFB9*	0.4955	0.537	0.266	3.973e-32	9.176e-28
5	Smooth muscle cells	*DACT1*	0.4953	0.135	0.033	3.321e-34	7.668e-30
6	Smooth muscle cells	*NAF1*	0.4949	0.417	0.152	2.963e-46	6.841e-42
6	Smooth muscle cells	*INF2*	0.4945	0.253	0.044	8.634e-83	1.994e-78
6	Smooth muscle cells	*COL4A4*	0.4944	0.229	0.026	5.897e-115	1.362e-110

Original cluster	Annotated cell type	Gene	avg_log2FC	pct.1	pct.2	Raw P value	Bonferroni-adjusted P value
6	Smooth muscle cells	*BTG2*	0.4944	0.967	0.882	9.295e-36	2.146e-31
6	Smooth muscle cells	*GANC*	0.4936	0.273	0.084	1.934e-40	4.466e-36
5	Smooth muscle cells	*OLFML1*	0.4935	0.157	0.039	1.554e-38	3.589e-34
5	Smooth muscle cells	*ADK*	0.4934	0.337	0.278	1.196e-06	2.761e-02
5	Smooth muscle cells	*IGSF10*	0.4933	0.266	0.179	3.003e-10	6.934e-06
5	Smooth muscle cells	*IVNS1ABP*	0.4930	0.311	0.246	2.933e-07	6.774e-03
6	Smooth muscle cells	*DIPK1A*	0.4927	0.264	0.039	5.530e-102	1.277e-97
6	Smooth muscle cells	*CDK2AP2*	0.4924	0.312	0.094	2.159e-48	4.987e-44
5	Smooth muscle cells	*PLXND1*	0.4922	0.200	0.095	9.555e-18	2.206e-13
5	Smooth muscle cells	*SETD5*	0.4917	0.421	0.381	4.447e-07	1.027e-02
6	Smooth muscle cells	*KCNMA1*	0.4913	0.362	0.114	2.792e-51	6.447e-47
6	Smooth muscle cells	*DMPK*	0.4907	0.214	0.023	1.331e-112	3.073e-108
6	Smooth muscle cells	*PPP1R14B*	0.4902	0.533	0.235	1.115e-39	2.575e-35
6	Smooth muscle cells	*C1QTNF1*	0.4895	0.240	0.033	1.009e-97	2.330e-93
6	Smooth muscle cells	*CDKN1A*	0.4880	0.317	0.107	6.810e-40	1.573e-35
6	Smooth muscle cells	*DDX5*	0.4878	0.956	0.893	3.856e-33	8.905e-29
6	Smooth muscle cells	*IRAG1*	0.4871	0.312	0.067	1.028e-75	2.375e-71
6	Smooth muscle cells	*EMP2*	0.4870	0.579	0.327	4.367e-27	1.009e-22
6	Smooth muscle cells	*TMSB4X*	0.4864	0.993	0.974	1.717e-64	3.966e-60
6	Smooth muscle cells	*ARF4*	0.4863	0.764	0.512	1.384e-28	3.196e-24
6	Smooth muscle cells	*NDUFB7*	0.4861	0.668	0.388	3.867e-31	8.929e-27
6	Smooth muscle cells	*RAN*	0.4858	0.424	0.165	8.043e-42	1.857e-37
6	Smooth muscle cells	*LOC100342119*	0.4849	0.146	0.006	5.186e-150	1.198e-145
5	Smooth muscle cells	*IER2*	0.4840	0.767	0.820	1.983e-09	4.580e-05
5	Smooth muscle cells	*FYN*	0.4840	0.328	0.256	2.132e-08	4.923e-04
6	Smooth muscle cells	*BAG1*	0.4836	0.338	0.109	7.429e-48	1.715e-43
6	Smooth muscle cells	*ZCCHC2*	0.4832	0.277	0.096	1.007e-34	2.326e-30
6	Smooth muscle cells	*PCP4*	0.4819	0.144	0.011	4.295e-97	9.919e-93
5	Smooth muscle cells	*CCNYL1*	0.4818	0.169	0.087	2.134e-12	4.928e-08
5	Smooth muscle cells	*ENPP1*	0.4814	0.146	0.069	7.074e-13	1.634e-08
6	Smooth muscle cells	*COX7A2*	0.4812	0.823	0.604	1.972e-27	4.554e-23
5	Smooth muscle cells	*LOC138843459*	0.4801	0.135	0.058	3.894e-14	8.991e-10
6	Smooth muscle cells	*SKIL*	0.4792	0.380	0.155	8.087e-35	1.867e-30
5	Smooth muscle cells	*LOC138849600*	0.4783	0.133	0.058	1.030e-13	2.379e-09
5	Smooth muscle cells	*SHOC2*	0.4766	0.432	0.379	3.535e-08	8.164e-04
5	Smooth muscle cells	*LOC103349547*	0.4760	0.107	0.034	4.538e-19	1.048e-14
5	Smooth muscle cells	*LUZP1*	0.4749	0.151	0.068	5.062e-15	1.169e-10
5	Smooth muscle cells	*FREM2*	0.4742	0.129	0.063	1.475e-10	3.405e-06
5	Smooth muscle cells	*SIPA1L1*	0.4741	0.333	0.250	4.015e-09	9.271e-05
5	Smooth muscle cells	*CD248*	0.4734	0.155	0.070	5.465e-15	1.262e-10

Original cluster	Annotated cell type	Gene	avg_log2FC	pct.1	pct.2	Raw P value	Bonferroni-adjusted P value
5	Smooth muscle cells	*STARD13*	0.4733	0.308	0.217	7.737e-10	1.787e-05
5	Smooth muscle cells	*RAB3IP*	0.4724	0.162	0.081	1.386e-12	3.200e-08
5	Smooth muscle cells	*RNF152*	0.4722	0.124	0.026	7.688e-38	1.775e-33
5	Smooth muscle cells	*ICAM1*	0.4715	0.155	0.075	8.109e-13	1.873e-08
5	Smooth muscle cells	*SSR3*	0.4713	0.625	0.581	2.547e-14	5.882e-10
5	Smooth muscle cells	*RAB8B*	0.4713	0.384	0.303	3.991e-10	9.217e-06
5	Smooth muscle cells	*RCSD1*	0.4710	0.202	0.092	4.239e-19	9.789e-15
5	Smooth muscle cells	*ARHGAP24*	0.4708	0.131	0.053	2.019e-15	4.662e-11
6	Smooth muscle cells	*NDUFS7*	0.4702	0.349	0.121	8.575e-43	1.980e-38
5	Smooth muscle cells	*FSTL3*	0.4691	0.109	0.012	5.803e-62	1.340e-57
5	Smooth muscle cells	*MACF1*	0.4683	0.532	0.482	2.038e-08	4.705e-04
5	Smooth muscle cells	*LOC138843958*	0.4678	0.186	0.110	7.892e-10	1.823e-05
6	Smooth muscle cells	*ATP5IF1*	0.4677	0.692	0.459	1.014e-22	2.342e-18
5	Smooth muscle cells	*PDE5A*	0.4674	0.392	0.299	1.611e-10	3.719e-06
6	Smooth muscle cells	*OLFM3*	0.4673	0.170	0.023	3.328e-70	7.684e-66
5	Smooth muscle cells	*CFAP210*	0.4671	0.135	0.082	1.861e-06	4.298e-02
5	Smooth muscle cells	*SRSF10*	0.4662	0.505	0.449	4.355e-11	1.006e-06
5	Smooth muscle cells	*TSHZ2*	0.4662	0.497	0.449	3.025e-08	6.985e-04
6	Smooth muscle cells	*BTBD9*	0.4660	0.450	0.249	3.297e-20	7.614e-16
6	Smooth muscle cells	*SORBS1*	0.4659	0.269	0.064	2.966e-57	6.849e-53
6	Smooth muscle cells	*DEK*	0.4652	0.712	0.464	1.393e-24	3.218e-20
6	Smooth muscle cells	*TJP1*	0.4629	0.491	0.217	8.860e-37	2.046e-32
6	Smooth muscle cells	*MTMR7*	0.4623	0.240	0.027	9.980e-120	2.305e-115
6	Smooth muscle cells	*GPX4*	0.4620	0.777	0.537	6.823e-24	1.576e-19
6	Smooth muscle cells	*SKP1*	0.4618	0.670	0.407	8.411e-25	1.942e-20
6	Smooth muscle cells	*MAP4*	0.4614	0.520	0.266	6.277e-28	1.450e-23
6	Smooth muscle cells	*BZW2*	0.4611	0.376	0.145	3.415e-37	7.887e-33
5	Smooth muscle cells	*RAPGEF2*	0.4604	0.339	0.280	1.350e-06	3.118e-02
5	Smooth muscle cells	*PLEKHA5*	0.4589	0.563	0.540	1.343e-06	3.101e-02
5	Smooth muscle cells	*GJC1*	0.4570	0.129	0.023	1.828e-48	4.222e-44
5	Smooth muscle cells	*COL3A1*	0.4562	0.714	0.682	2.903e-10	6.703e-06
6	Smooth muscle cells	*SMARCA1*	0.4561	0.378	0.203	1.404e-17	3.243e-13
6	Smooth muscle cells	*CIAO2B*	0.4557	0.611	0.351	7.580e-26	1.750e-21
6	Smooth muscle cells	*RASGRP2*	0.4557	0.203	0.016	1.811e-137	4.183e-133
6	Smooth muscle cells	*CYB5R3*	0.4533	0.579	0.326	1.556e-25	3.594e-21
6	Smooth muscle cells	*AKAP1*	0.4529	0.242	0.048	3.378e-66	7.801e-62
5	Smooth muscle cells	*NUMB*	0.4522	0.368	0.315	3.482e-07	8.040e-03
6	Smooth muscle cells	*PCDH19*	0.4522	0.183	0.030	5.254e-63	1.213e-58
5	Smooth muscle cells	*TOP1*	0.4514	0.566	0.571	1.975e-06	4.560e-02
6	Smooth muscle cells	*CS*	0.4508	0.469	0.222	3.002e-30	6.931e-26

Original cluster	Annotated cell type	Gene	avg_log2FC	pct.1	pct.2	Raw P value	Bonferroni-adjusted P value
5	Smooth muscle cells	*GRK5*	0.4501	0.177	0.088	6.162e-14	1.423e-09
6	Smooth muscle cells	*NDRG4*	0.4494	0.210	0.019	4.093e-124	9.452e-120
6	Smooth muscle cells	*EML2*	0.4491	0.266	0.068	3.528e-52	8.147e-48
5	Smooth muscle cells	*SPRY1*	0.4486	0.157	0.078	2.393e-12	5.525e-08
6	Smooth muscle cells	*ADGRA2*	0.4468	0.376	0.115	3.860e-53	8.914e-49
5	Smooth muscle cells	*THBD*	0.4463	0.149	0.050	1.348e-23	3.113e-19
6	Smooth muscle cells	*RILPL1*	0.4462	0.373	0.127	4.158e-45	9.602e-41
6	Smooth muscle cells	*ADSS1*	0.4462	0.186	0.011	4.614e-150	1.066e-145
5	Smooth muscle cells	*AMOTL2*	0.4462	0.158	0.083	4.111e-11	9.493e-07
6	Smooth muscle cells	*PPP2R5A*	0.4441	0.469	0.216	1.401e-31	3.234e-27
6	Smooth muscle cells	*HINT1*	0.4428	0.633	0.390	7.632e-22	1.762e-17
6	Smooth muscle cells	*CD9*	0.4428	0.336	0.068	2.357e-87	5.442e-83
6	Smooth muscle cells	*HUNK*	0.4427	0.197	0.033	1.751e-65	4.044e-61
6	Smooth muscle cells	*KCTD10*	0.4422	0.253	0.071	1.270e-43	2.934e-39
6	Smooth muscle cells	*ZBTB1*	0.4420	0.253	0.090	2.070e-29	4.780e-25
6	Smooth muscle cells	*NCL*	0.4415	0.814	0.589	2.764e-22	6.383e-18
6	Smooth muscle cells	*PDLIM7*	0.4400	0.288	0.092	6.030e-40	1.392e-35
5	Smooth muscle cells	*GALNT18*	0.4397	0.169	0.070	8.417e-19	1.944e-14
5	Smooth muscle cells	*LOC103347881*	0.4385	0.135	0.064	1.403e-11	3.241e-07
6	Smooth muscle cells	*PTP4A1*	0.4376	0.456	0.269	3.622e-18	8.365e-14
5	Smooth muscle cells	*MAPK6*	0.4371	0.189	0.123	1.102e-07	2.544e-03
6	Smooth muscle cells	*FGF1*	0.4367	0.201	0.012	4.732e-162	1.093e-157
6	Smooth muscle cells	*FAM184A*	0.4366	0.118	0.060	4.130e-07	9.538e-03
6	Smooth muscle cells	*PHTF2*	0.4352	0.306	0.105	1.376e-37	3.177e-33
5	Smooth muscle cells	*PRICKLE2*	0.4339	0.220	0.144	6.687e-09	1.544e-04
6	Smooth muscle cells	*NEURL2*	0.4319	0.229	0.035	5.707e-83	1.318e-78
6	Smooth muscle cells	*BAG3*	0.4316	0.328	0.124	2.406e-33	5.556e-29
6	Smooth muscle cells	*LOC103349248*	0.4311	0.192	0.005	1.777e-239	4.104e-235
6	Smooth muscle cells	*DCUN1D3*	0.4304	0.223	0.048	8.165e-55	1.886e-50
5	Smooth muscle cells	*FRMD6*	0.4297	0.171	0.107	1.280e-07	2.956e-03
6	Smooth muscle cells	*SEMA4C*	0.4294	0.216	0.050	3.073e-49	7.097e-45
6	Smooth muscle cells	*MFHAS1*	0.4290	0.258	0.068	3.378e-48	7.800e-44
5	Smooth muscle cells	*PTPN9*	0.4287	0.209	0.132	1.850e-09	4.271e-05
6	Smooth muscle cells	*TUBB6*	0.4285	0.310	0.122	3.283e-29	7.581e-25
6	Smooth muscle cells	*COX6A1*	0.4283	0.793	0.561	3.237e-22	7.475e-18
6	Smooth muscle cells	*GSPT1*	0.4280	0.489	0.239	3.128e-28	7.223e-24
5	Smooth muscle cells	*CCDC102B*	0.4276	0.120	0.056	2.182e-10	5.040e-06
6	Smooth muscle cells	*PDE4DIP*	0.4275	0.260	0.057	1.525e-62	3.522e-58
5	Smooth muscle cells	*HUWE1*	0.4271	0.397	0.335	3.415e-08	7.885e-04
6	Smooth muscle cells	*BIRC3*	0.4268	0.109	0.022	1.801e-30	4.159e-26

Original cluster	Annotated cell type	Gene	avg_log2FC	pct.1	pct.2	Raw P value	Bonferroni-adjusted P value
5	Smooth muscle cells	*RAPGEF5*	0.4265	0.122	0.039	2.630e-21	6.074e-17
5	Smooth muscle cells	*IGFBP6*	0.4261	0.410	0.335	5.967e-08	1.378e-03
6	Smooth muscle cells	*ITPR1*	0.4258	0.330	0.101	3.059e-48	7.063e-44
5	Smooth muscle cells	*SLC25A33*	0.4253	0.148	0.056	3.647e-20	8.422e-16
6	Smooth muscle cells	*CCT6A*	0.4250	0.657	0.416	2.154e-22	4.975e-18
6	Smooth muscle cells	*HEATR5B*	0.4247	0.273	0.074	1.382e-48	3.190e-44
5	Smooth muscle cells	*CISH*	0.4246	0.135	0.072	3.163e-09	7.303e-05
6	Smooth muscle cells	*OGDH*	0.4244	0.397	0.165	6.595e-34	1.523e-29
6	Smooth muscle cells	*MTSS2*	0.4225	0.299	0.090	7.437e-46	1.717e-41
6	Smooth muscle cells	*ATP5F1A*	0.4224	0.603	0.364	1.904e-20	4.396e-16
5	Smooth muscle cells	*PLEKHA2*	0.4215	0.169	0.099	3.279e-09	7.573e-05
6	Smooth muscle cells	*LOC100350425*	0.4214	0.175	0.036	9.948e-46	2.297e-41
6	Smooth muscle cells	*HNRNPA0*	0.4212	0.537	0.295	1.381e-22	3.189e-18
5	Smooth muscle cells	*TLE1*	0.4211	0.299	0.228	1.621e-07	3.743e-03
5	Smooth muscle cells	*SRSF7*	0.4210	0.760	0.743	5.159e-12	1.191e-07
6	Smooth muscle cells	*PRKAG2*	0.4200	0.330	0.202	7.323e-11	1.691e-06
5	Smooth muscle cells	*AKAP12*	0.4196	0.432	0.346	1.536e-09	3.547e-05
6	Smooth muscle cells	*KEF51-p01*	0.4195	0.998	0.989	8.267e-50	1.909e-45
6	Smooth muscle cells	*ALDOA*	0.4192	0.672	0.456	1.762e-17	4.069e-13
6	Smooth muscle cells	*RHOA*	0.4186	0.843	0.602	7.615e-21	1.758e-16
6	Smooth muscle cells	*NDUFB10*	0.4180	0.878	0.684	1.477e-21	3.410e-17
6	Smooth muscle cells	*APPL1*	0.4163	0.408	0.195	3.870e-25	8.937e-21
6	Smooth muscle cells	*RHOJ*	0.4158	0.483	0.216	6.442e-36	1.488e-31
5	Smooth muscle cells	*GPBP1*	0.4153	0.574	0.547	3.577e-09	8.260e-05
6	Smooth muscle cells	*PDLIM2*	0.4152	0.247	0.072	4.913e-40	1.134e-35
6	Smooth muscle cells	*ADAMTS4*	0.4152	0.107	0.019	4.679e-34	1.080e-29
5	Smooth muscle cells	*FBXO30*	0.4148	0.191	0.127	3.236e-07	7.472e-03
6	Smooth muscle cells	*CASQ2*	0.4142	0.168	0.001	6.091e-278	1.407e-273
6	Smooth muscle cells	*PDGFB*	0.4139	0.188	0.019	2.818e-103	6.507e-99
6	Smooth muscle cells	*PPP2R5E*	0.4130	0.487	0.291	3.829e-17	8.841e-13
6	Smooth muscle cells	*EID1*	0.4128	0.725	0.505	2.467e-17	5.696e-13
6	Smooth muscle cells	*HSPA5*	0.4125	0.729	0.462	3.355e-22	7.748e-18
6	Smooth muscle cells	*KSR1*	0.4113	0.271	0.120	4.310e-20	9.952e-16
6	Smooth muscle cells	*MBOAT1*	0.4107	0.258	0.061	1.980e-54	4.572e-50
6	Smooth muscle cells	*LOC100345591*	0.4107	0.513	0.288	6.007e-22	1.387e-17
6	Smooth muscle cells	*CRTC3*	0.4099	0.365	0.168	6.165e-24	1.424e-19
5	Smooth muscle cells	*AKT3*	0.4087	0.583	0.549	6.698e-09	1.547e-04
5	Smooth muscle cells	*LZTS2*	0.4082	0.277	0.217	9.211e-07	2.127e-02
5	Smooth muscle cells	*ZNF214*	0.4081	0.146	0.067	2.726e-13	6.295e-09
6	Smooth muscle cells	*PLAAT3*	0.4081	0.450	0.148	2.372e-54	5.477e-50

Original cluster	Annotated cell type	Gene	avg_log2FC	pct.1	pct.2	Raw P value	Bonferroni-adjusted P value
5	Smooth muscle cells	*PNRC1*	0.4070	0.831	0.836	4.048e-12	9.348e-08
6	Smooth muscle cells	*DHRS3*	0.4064	0.221	0.054	6.273e-47	1.448e-42
5	Smooth muscle cells	*UBE2B*	0.4058	0.630	0.675	8.308e-07	1.918e-02
5	Smooth muscle cells	*CHD1*	0.4057	0.341	0.281	8.897e-07	2.055e-02
6	Smooth muscle cells	*GMPS*	0.4055	0.371	0.183	1.922e-21	4.437e-17
5	Smooth muscle cells	*NXT1*	0.4053	0.144	0.070	3.087e-12	7.128e-08
5	Smooth muscle cells	*TCF12*	0.4052	0.729	0.729	2.041e-07	4.714e-03
6	Smooth muscle cells	*LGI4*	0.4051	0.162	0.010	2.493e-127	5.756e-123
5	Smooth muscle cells	*SLC2A1*	0.4048	0.113	0.035	2.031e-20	4.690e-16
6	Smooth muscle cells	*PSMB6*	0.4036	0.585	0.334	4.111e-23	9.493e-19
5	Smooth muscle cells	*ATP1B3*	0.4036	0.244	0.175	1.730e-07	3.995e-03
6	Smooth muscle cells	*ACTR3*	0.4029	0.513	0.260	3.228e-29	7.454e-25
6	Smooth muscle cells	*LOC103345647*	0.4029	0.415	0.206	3.421e-23	7.899e-19
6	Smooth muscle cells	*CKM*	0.4016	0.124	0.007	8.322e-106	1.922e-101
6	Smooth muscle cells	*FHL3*	0.4016	0.216	0.031	1.991e-83	4.598e-79
6	Smooth muscle cells	*RYBP*	0.4014	0.474	0.317	3.015e-11	6.963e-07
6	Smooth muscle cells	*SYAP1*	0.4007	0.297	0.139	3.545e-20	8.186e-16
6	Smooth muscle cells	*NEURL1B*	0.4007	0.188	0.025	4.339e-79	1.002e-74
6	Smooth muscle cells	*NTHL1*	0.4006	0.395	0.181	1.146e-26	2.646e-22
6	Smooth muscle cells	*KEF51-p10*	0.3994	0.998	0.994	1.929e-53	4.454e-49
5	Smooth muscle cells	*MAF*	0.3992	0.310	0.247	1.386e-06	3.200e-02
6	Smooth muscle cells	*DRAP1*	0.3989	0.476	0.254	2.078e-21	4.800e-17
6	Smooth muscle cells	*SNTA1*	0.3988	0.376	0.177	8.160e-24	1.884e-19
6	Smooth muscle cells	*BOD1L1*	0.3986	0.675	0.452	9.552e-19	2.206e-14
6	Smooth muscle cells	*HOOK3*	0.3981	0.666	0.428	9.406e-20	2.172e-15
6	Smooth muscle cells	*ATP5MG*	0.3970	0.686	0.453	3.463e-18	7.997e-14
6	Smooth muscle cells	*CDS2*	0.3969	0.312	0.108	2.095e-37	4.837e-33
6	Smooth muscle cells	*ST6GAL1*	0.3966	0.269	0.060	2.050e-62	4.734e-58
5	Smooth muscle cells	*LOC103349873*	0.3954	0.148	0.083	1.051e-08	2.427e-04
6	Smooth muscle cells	*NDUFA6*	0.3950	0.533	0.318	1.697e-18	3.918e-14
5	Smooth muscle cells	*KIF27*	0.3941	0.171	0.088	1.966e-12	4.540e-08
6	Smooth muscle cells	*BUD31*	0.3940	0.570	0.335	1.369e-20	3.162e-16
6	Smooth muscle cells	*HSPB6*	0.3930	0.236	0.060	4.757e-45	1.098e-40
6	Smooth muscle cells	*NAPA*	0.3929	0.266	0.083	3.045e-38	7.031e-34
6	Smooth muscle cells	*ADAMTS8*	0.3927	0.181	0.027	3.673e-68	8.482e-64
6	Smooth muscle cells	*FAM81A*	0.3922	0.286	0.105	3.456e-30	7.981e-26
5	Smooth muscle cells	*VSTM4*	0.3917	0.164	0.077	1.230e-14	2.840e-10
6	Smooth muscle cells	*TTC19*	0.3915	0.306	0.123	1.142e-27	2.637e-23
6	Smooth muscle cells	*CTPS2*	0.3913	0.317	0.170	3.632e-15	8.386e-11
6	Smooth muscle cells	*JPT1*	0.3894	0.317	0.128	4.727e-28	1.092e-23

Original cluster	Annotated cell type	Gene	avg_log2FC	pct.1	pct.2	Raw P value	Bonferroni-adjusted P value
6	Smooth muscle cells	*ATP5MC3*	0.3885	0.753	0.533	1.889e-18	4.363e-14
6	Smooth muscle cells	*RPS27L*	0.3880	0.797	0.586	3.347e-18	7.730e-14
6	Smooth muscle cells	*LRRFIP1*	0.3875	0.408	0.195	5.794e-25	1.338e-20
5	Smooth muscle cells	*LUC7L2*	0.3873	0.386	0.326	3.197e-07	7.382e-03
5	Smooth muscle cells	*LRP12*	0.3860	0.158	0.100	1.154e-06	2.665e-02
6	Smooth muscle cells	*LOC138850734*	0.3831	0.197	0.033	1.197e-64	2.763e-60
6	Smooth muscle cells	*MRPS6*	0.3831	0.295	0.103	1.107e-33	2.557e-29
6	Smooth muscle cells	*CDH13*	0.3830	0.227	0.047	5.215e-58	1.204e-53
6	Smooth muscle cells	*LOC108176367*	0.3829	0.107	0.005	1.180e-99	2.724e-95
6	Smooth muscle cells	*LOC108177422*	0.3826	0.417	0.231	2.497e-18	5.766e-14
6	Smooth muscle cells	*FLNA*	0.3824	0.214	0.036	4.668e-70	1.078e-65
6	Smooth muscle cells	*DNAJB9*	0.3824	0.515	0.322	7.609e-16	1.757e-11
6	Smooth muscle cells	*RBM3*	0.3804	0.793	0.600	3.682e-16	8.503e-12
5	Smooth muscle cells	*PNPLA2*	0.3793	0.153	0.076	4.296e-12	9.920e-08
6	Smooth muscle cells	*KHDRBS3*	0.3791	0.332	0.168	6.114e-17	1.412e-12
5	Smooth muscle cells	*HEY1*	0.3756	0.126	0.050	5.305e-15	1.225e-10
6	Smooth muscle cells	*LOC108177962*	0.3747	0.194	0.040	4.147e-51	9.576e-47
6	Smooth muscle cells	*BIRC2*	0.3747	0.408	0.232	4.567e-16	1.055e-11
6	Smooth muscle cells	*ACSL3*	0.3743	0.317	0.117	8.868e-33	2.048e-28
5	Smooth muscle cells	*ITPRIP*	0.3739	0.120	0.056	8.063e-11	1.862e-06
6	Smooth muscle cells	*CAMK2G*	0.3738	0.336	0.114	7.804e-40	1.802e-35
5	Smooth muscle cells	*LRRC8C*	0.3736	0.135	0.058	1.227e-13	2.834e-09
5	Smooth muscle cells	*ECRG4*	0.3732	0.115	0.055	2.638e-09	6.091e-05
6	Smooth muscle cells	*INKA2*	0.3729	0.155	0.009	6.232e-129	1.439e-124
6	Smooth muscle cells	*SOX4*	0.3727	0.633	0.457	9.199e-12	2.124e-07
6	Smooth muscle cells	*OLFML2A*	0.3722	0.212	0.034	3.274e-72	7.561e-68
6	Smooth muscle cells	*CD109*	0.3714	0.221	0.052	9.396e-48	2.170e-43
6	Smooth muscle cells	*PLEC*	0.3709	0.321	0.123	2.748e-31	6.346e-27
5	Smooth muscle cells	*SMURF2*	0.3708	0.310	0.241	5.816e-07	1.343e-02
6	Smooth muscle cells	*THRAP3*	0.3695	0.603	0.423	5.953e-13	1.375e-08
6	Smooth muscle cells	*HAPSTR1*	0.3691	0.395	0.197	2.330e-22	5.381e-18
5	Smooth muscle cells	*BCLAF1*	0.3683	0.550	0.520	3.888e-08	8.979e-04
6	Smooth muscle cells	*RNF149*	0.3678	0.362	0.168	7.345e-24	1.696e-19
6	Smooth muscle cells	*GLO1*	0.3674	0.485	0.273	2.152e-19	4.969e-15
6	Smooth muscle cells	*LOC103347958*	0.3671	0.133	0.016	9.303e-63	2.148e-58
6	Smooth muscle cells	*LOC127483303*	0.3667	0.148	0.005	2.247e-169	5.189e-165
6	Smooth muscle cells	*MEF2D*	0.3665	0.251	0.073	3.278e-40	7.570e-36
6	Smooth muscle cells	*MAPRE1*	0.3664	0.500	0.276	2.476e-20	5.717e-16
6	Smooth muscle cells	*TCF7L1*	0.3660	0.496	0.254	2.137e-24	4.935e-20
5	Smooth muscle cells	*TIAM2*	0.3649	0.128	0.074	5.450e-07	1.258e-02

Original cluster	Annotated cell type	Gene	avg_log2FC	pct.1	pct.2	Raw P value	Bonferroni-adjusted P value
6	Smooth muscle cells	*BCAS2*	0.3645	0.445	0.284	9.123e-12	2.107e-07
5	Smooth muscle cells	*GPR63*	0.3638	0.126	0.064	3.789e-09	8.750e-05
6	Smooth muscle cells	*HNRNPAB*	0.3634	0.644	0.410	5.971e-18	1.379e-13
6	Smooth muscle cells	*TOB1*	0.3626	0.821	0.672	7.394e-13	1.707e-08
5	Smooth muscle cells	*POLR1D*	0.3625	0.780	0.779	4.550e-11	1.051e-06
6	Smooth muscle cells	*THRB*	0.3624	0.480	0.277	2.981e-18	6.883e-14
6	Smooth muscle cells	*GRIN2B*	0.3610	0.118	0.009	1.013e-81	2.339e-77
6	Smooth muscle cells	*LOC108177469*	0.3606	0.107	0.003	6.400e-138	1.478e-133
6	Smooth muscle cells	*PSMB3*	0.3606	0.380	0.204	1.374e-17	3.173e-13
5	Smooth muscle cells	*LGALS3*	0.3596	0.155	0.075	2.907e-12	6.713e-08
6	Smooth muscle cells	*PCP4L1*	0.3594	0.144	0.002	1.108e-208	2.559e-204
6	Smooth muscle cells	*GPR21*	0.3588	0.146	0.018	1.746e-68	4.032e-64
6	Smooth muscle cells	*KEF51-p07*	0.3584	0.998	0.994	1.696e-38	3.917e-34
6	Smooth muscle cells	*SMDT1*	0.3569	0.382	0.180	3.122e-23	7.210e-19
6	Smooth muscle cells	*ATG13*	0.3566	0.227	0.094	1.164e-19	2.687e-15
5	Smooth muscle cells	*LOC138844523*	0.3561	0.117	0.041	2.114e-17	4.881e-13
6	Smooth muscle cells	*PPP1CB*	0.3560	0.662	0.434	3.755e-19	8.671e-15
6	Smooth muscle cells	*SMARCD3*	0.3556	0.188	0.031	2.082e-62	4.808e-58
5	Smooth muscle cells	*COL1A1*	0.3553	0.628	0.627	8.350e-07	1.928e-02
6	Smooth muscle cells	*KCNJ12*	0.3552	0.142	0.001	5.901e-246	1.363e-241
5	Smooth muscle cells	*CAVIN2*	0.3552	0.148	0.053	3.188e-20	7.361e-16
6	Smooth muscle cells	*RGS2*	0.3542	0.397	0.215	1.004e-16	2.320e-12
6	Smooth muscle cells	*ATP5PB*	0.3538	0.686	0.479	5.339e-15	1.233e-10
6	Smooth muscle cells	*MRGPRF*	0.3535	0.264	0.091	9.287e-31	2.145e-26
6	Smooth muscle cells	*IPO7*	0.3533	0.378	0.188	7.569e-21	1.748e-16
5	Smooth muscle cells	*HMG20B*	0.3532	0.184	0.124	7.298e-07	1.685e-02
6	Smooth muscle cells	*IPO5*	0.3524	0.330	0.148	4.311e-23	9.955e-19
6	Smooth muscle cells	*MAOA*	0.3522	0.229	0.058	3.275e-44	7.563e-40
6	Smooth muscle cells	*AXL*	0.3517	0.465	0.241	9.368e-21	2.163e-16
6	Smooth muscle cells	*TMEM38B*	0.3514	0.266	0.105	4.760e-25	1.099e-20
5	Smooth muscle cells	*SAT1*	0.3508	0.472	0.419	1.997e-06	4.610e-02
6	Smooth muscle cells	*ARIH1*	0.3500	0.441	0.265	1.538e-14	3.551e-10
6	Smooth muscle cells	*SEPTIN8*	0.3491	0.253	0.075	2.877e-38	6.643e-34
6	Smooth muscle cells	*CCT4*	0.3485	0.524	0.317	1.003e-16	2.317e-12
6	Smooth muscle cells	*MN1*	0.3485	0.170	0.031	2.375e-51	5.484e-47
6	Smooth muscle cells	*GPRC5C*	0.3476	0.225	0.081	1.812e-25	4.184e-21
6	Smooth muscle cells	*UCHL5*	0.3470	0.295	0.133	4.170e-21	9.628e-17
6	Smooth muscle cells	*ATP6V0A1*	0.3466	0.277	0.099	1.968e-31	4.543e-27
6	Smooth muscle cells	*SLC6A9*	0.3461	0.146	0.006	1.153e-145	2.662e-141
6	Smooth muscle cells	*PABPC1*	0.3459	0.956	0.872	6.367e-22	1.470e-17

Original cluster	Annotated cell type	Gene	avg_log2FC	pct.1	pct.2	Raw P value	Bonferroni-adjusted P value
6	Smooth muscle cells	*NAGK*	0.3452	0.277	0.102	1.615e-29	3.730e-25
5	Smooth muscle cells	*ENPEP*	0.3451	0.107	0.051	2.029e-09	4.685e-05
6	Smooth muscle cells	*STRAP*	0.3447	0.463	0.243	1.345e-20	3.105e-16
6	Smooth muscle cells	*HIVEP2*	0.3447	0.618	0.449	1.016e-10	2.346e-06
5	Smooth muscle cells	*S100A11*	0.3444	0.801	0.749	3.449e-20	7.965e-16
6	Smooth muscle cells	*NDUFC2*	0.3440	0.386	0.192	2.227e-20	5.143e-16
6	Smooth muscle cells	*LOC103349722*	0.3436	0.511	0.314	2.020e-15	4.666e-11
6	Smooth muscle cells	*ASAP2*	0.3429	0.284	0.103	4.302e-31	9.933e-27
6	Smooth muscle cells	*ZFPM2*	0.3428	0.443	0.188	6.529e-34	1.508e-29
6	Smooth muscle cells	*LOC138846541*	0.3427	0.144	0.009	2.218e-117	5.122e-113
6	Smooth muscle cells	*TAF13*	0.3422	0.277	0.111	5.963e-25	1.377e-20
6	Smooth muscle cells	*NDUFS2*	0.3420	0.592	0.369	3.764e-17	8.692e-13
6	Smooth muscle cells	*RAB7A*	0.3418	0.515	0.323	8.552e-15	1.975e-10
6	Smooth muscle cells	*TCP1*	0.3417	0.456	0.242	6.488e-20	1.498e-15
6	Smooth muscle cells	*MEOX2*	0.3406	0.120	0.006	1.106e-109	2.554e-105
6	Smooth muscle cells	*DZIP1*	0.3401	0.290	0.114	6.901e-26	1.593e-21
6	Smooth muscle cells	*FCHSD2*	0.3400	0.400	0.236	4.487e-14	1.036e-09
6	Smooth muscle cells	*EHD1*	0.3400	0.303	0.127	1.340e-24	3.093e-20
6	Smooth muscle cells	*HIPK3*	0.3399	0.528	0.337	2.084e-13	4.813e-09
6	Smooth muscle cells	*ADCY6*	0.3397	0.229	0.075	2.121e-30	4.898e-26
6	Smooth muscle cells	*ETV3*	0.3395	0.282	0.121	6.354e-22	1.467e-17
6	Smooth muscle cells	*CALM3*	0.3389	0.644	0.434	7.954e-15	1.837e-10
6	Smooth muscle cells	*CCDC127*	0.3389	0.406	0.210	2.529e-19	5.840e-15
6	Smooth muscle cells	*INPP5F*	0.3384	0.258	0.073	3.101e-42	7.160e-38
6	Smooth muscle cells	*DTNA*	0.3372	0.164	0.016	4.530e-89	1.046e-84
6	Smooth muscle cells	*KIF1B*	0.3370	0.437	0.243	2.921e-18	6.745e-14
6	Smooth muscle cells	*ENAH*	0.3367	0.421	0.247	1.414e-14	3.266e-10
6	Smooth muscle cells	*PON2*	0.3357	0.325	0.153	2.742e-20	6.333e-16
6	Smooth muscle cells	*KANK1*	0.3356	0.242	0.071	6.073e-38	1.402e-33
6	Smooth muscle cells	*GALNT11*	0.3353	0.181	0.103	3.576e-07	8.257e-03
6	Smooth muscle cells	*ALX1*	0.3347	0.120	0.007	1.214e-100	2.802e-96
6	Smooth muscle cells	*KLF8*	0.3343	0.181	0.059	2.012e-24	4.646e-20
6	Smooth muscle cells	*BCL2L1*	0.3342	0.199	0.060	1.037e-30	2.395e-26
6	Smooth muscle cells	*CHMP1B*	0.3337	0.253	0.121	9.828e-16	2.269e-11
6	Smooth muscle cells	*NDUFA7*	0.3336	0.426	0.217	1.101e-20	2.542e-16
6	Smooth muscle cells	*CHD4*	0.3333	0.454	0.264	7.215e-16	1.666e-11
5	Smooth muscle cells	*TRIM47*	0.3331	0.138	0.064	1.012e-12	2.337e-08
6	Smooth muscle cells	*NPFFR2*	0.3329	0.116	0.005	1.313e-119	3.033e-115
6	Smooth muscle cells	*CCT8*	0.3328	0.522	0.330	2.545e-13	5.878e-09
6	Smooth muscle cells	*MACO1*	0.3328	0.493	0.274	4.502e-19	1.039e-14

Original cluster	Annotated cell type	Gene	avg_log2FC	pct.1	pct.2	Raw P value	Bonferroni-adjusted P value
6	Smooth muscle cells	*MAGOH*	0.3327	0.454	0.249	9.836e-19	2.271e-14
6	Smooth muscle cells	*VASP*	0.3320	0.288	0.099	7.264e-35	1.677e-30
6	Smooth muscle cells	*AFF4*	0.3318	0.524	0.320	8.848e-16	2.043e-11
6	Smooth muscle cells	*LOC127492083*	0.3313	0.114	0.006	6.763e-95	1.562e-90
6	Smooth muscle cells	*CCSER1*	0.3311	0.275	0.132	7.498e-17	1.731e-12
6	Smooth muscle cells	*AVPR1A*	0.3307	0.201	0.051	3.289e-39	7.596e-35
6	Smooth muscle cells	*CACNB4*	0.3305	0.118	0.022	3.332e-35	7.693e-31
6	Smooth muscle cells	*TANC2*	0.3304	0.325	0.182	1.355e-13	3.129e-09
6	Smooth muscle cells	*EFR3A*	0.3303	0.424	0.234	7.083e-18	1.636e-13
6	Smooth muscle cells	*YWHAQ*	0.3301	0.823	0.616	5.259e-16	1.214e-11
6	Smooth muscle cells	*TLCD5*	0.3300	0.181	0.035	2.507e-50	5.790e-46
6	Smooth muscle cells	*TMEM134*	0.3298	0.293	0.119	2.906e-25	6.711e-21
6	Smooth muscle cells	*PGM5*	0.3296	0.253	0.051	2.712e-64	6.263e-60
6	Smooth muscle cells	*RNF180*	0.3291	0.183	0.059	3.461e-25	7.993e-21
6	Smooth muscle cells	*IMPA2*	0.3291	0.175	0.030	1.714e-57	3.957e-53
6	Smooth muscle cells	*BPNT2*	0.3290	0.358	0.186	6.355e-18	1.468e-13
6	Smooth muscle cells	*RPL36A*	0.3283	0.989	0.945	2.251e-24	5.199e-20
6	Smooth muscle cells	*CHMP3*	0.3268	0.496	0.313	8.335e-14	1.925e-09
6	Smooth muscle cells	*STRN*	0.3254	0.373	0.215	8.629e-14	1.993e-09
6	Smooth muscle cells	*IFNGR1*	0.3251	0.349	0.158	2.686e-23	6.203e-19
6	Smooth muscle cells	*P3H2*	0.3251	0.214	0.062	1.318e-33	3.044e-29
6	Smooth muscle cells	*MAPRE3*	0.3251	0.251	0.098	4.726e-24	1.091e-19
6	Smooth muscle cells	*CCDC3*	0.3246	0.258	0.102	3.749e-23	8.657e-19
6	Smooth muscle cells	*CTSK*	0.3232	0.472	0.265	9.869e-18	2.279e-13
6	Smooth muscle cells	*SRSF4*	0.3219	0.557	0.354	1.928e-14	4.453e-10
6	Smooth muscle cells	*ANKRD50*	0.3215	0.528	0.322	4.066e-15	9.388e-11
6	Smooth muscle cells	*LIN7A*	0.3212	0.172	0.053	1.118e-25	2.582e-21
6	Smooth muscle cells	*PTH1R*	0.3211	0.277	0.112	1.243e-23	2.870e-19
6	Smooth muscle cells	*LDLRAD3*	0.3202	0.253	0.098	3.963e-24	9.152e-20
6	Smooth muscle cells	*LOC127490703*	0.3198	0.153	0.014	6.387e-91	1.475e-86
6	Smooth muscle cells	*PLEKHO1*	0.3192	0.214	0.054	7.159e-42	1.653e-37
6	Smooth muscle cells	*SIAH2*	0.3189	0.216	0.096	4.578e-16	1.057e-11
6	Smooth muscle cells	*TRIR*	0.3184	0.734	0.540	1.259e-12	2.907e-08
6	Smooth muscle cells	*PTGIR*	0.3184	0.120	0.003	2.890e-149	6.674e-145
6	Smooth muscle cells	*UBE2A*	0.3166	0.251	0.103	7.305e-22	1.687e-17
6	Smooth muscle cells	*DAAM2*	0.3164	0.199	0.051	1.354e-37	3.128e-33
6	Smooth muscle cells	*G3BP2*	0.3161	0.504	0.298	3.720e-16	8.591e-12
6	Smooth muscle cells	*RAB33B*	0.3160	0.192	0.061	4.130e-27	9.537e-23
6	Smooth muscle cells	*LOC138844924*	0.3157	0.168	0.025	2.396e-64	5.533e-60
6	Smooth muscle cells	*BCL9L*	0.3152	0.225	0.078	7.629e-27	1.762e-22

Original cluster	Annotated cell type	Gene	avg_log2FC	pct.1	pct.2	Raw P value	Bonferroni-adjusted P value
6	Smooth muscle cells	*TRPC3*	0.3152	0.144	0.013	6.173e-88	1.425e-83
6	Smooth muscle cells	*PFDN2*	0.3144	0.513	0.312	1.971e-14	4.551e-10
6	Smooth muscle cells	*DYNC1LI2*	0.3140	0.410	0.217	7.552e-19	1.744e-14
6	Smooth muscle cells	*HCFC1R1*	0.3135	0.384	0.199	7.737e-18	1.787e-13
6	Smooth muscle cells	*CHTOP*	0.3129	0.430	0.243	3.457e-16	7.984e-12
6	Smooth muscle cells	*MAP6*	0.3125	0.225	0.065	1.645e-35	3.798e-31
6	Smooth muscle cells	*OXCT1*	0.3123	0.234	0.095	2.010e-20	4.641e-16
6	Smooth muscle cells	*MTUS1*	0.3106	0.334	0.139	4.626e-26	1.068e-21
6	Smooth muscle cells	*RHOQ*	0.3101	0.299	0.122	1.439e-24	3.322e-20
6	Smooth muscle cells	*PLN*	0.3101	0.162	0.034	2.074e-40	4.789e-36
6	Smooth muscle cells	*IRF2BPL*	0.3101	0.321	0.186	5.484e-12	1.266e-07
6	Smooth muscle cells	*HIF1A*	0.3100	0.330	0.187	7.251e-13	1.674e-08
6	Smooth muscle cells	*BANF1*	0.3094	0.858	0.678	3.788e-15	8.747e-11
6	Smooth muscle cells	*SGCA*	0.3089	0.155	0.002	6.174e-231	1.426e-226
6	Smooth muscle cells	*SFPQ*	0.3088	0.808	0.660	3.164e-12	7.305e-08
6	Smooth muscle cells	*ISCA1*	0.3084	0.566	0.347	6.388e-17	1.475e-12
6	Smooth muscle cells	*UBR5*	0.3081	0.513	0.331	6.151e-13	1.420e-08
6	Smooth muscle cells	*RBPMS2*	0.3078	0.135	0.010	9.647e-93	2.228e-88
6	Smooth muscle cells	*PDCL3*	0.3076	0.452	0.241	8.485e-19	1.959e-14
6	Smooth muscle cells	*MORF4L1*	0.3075	0.760	0.570	1.749e-13	4.038e-09
6	Smooth muscle cells	*ESD*	0.3073	0.461	0.272	7.116e-14	1.643e-09
6	Smooth muscle cells	*LOC138843879*	0.3070	0.162	0.013	1.212e-107	2.800e-103
6	Smooth muscle cells	*MLEC*	0.3066	0.389	0.215	2.998e-15	6.924e-11
6	Smooth muscle cells	*RAB9A*	0.3043	0.583	0.369	9.804e-14	2.264e-09
5	Smooth muscle cells	*FAM222A*	0.3042	0.109	0.053	4.600e-09	1.062e-04
6	Smooth muscle cells	*ZNF131*	0.3034	0.306	0.175	4.775e-12	1.103e-07
6	Smooth muscle cells	*WFDC1*	0.3027	0.133	0.032	5.754e-29	1.329e-24
6	Smooth muscle cells	*CCDC110*	0.3024	0.138	0.015	1.186e-71	2.738e-67
5	Smooth muscle cells	*PEAR1*	0.3024	0.104	0.042	2.205e-12	5.093e-08
6	Smooth muscle cells	*RASL11B*	0.3018	0.238	0.096	1.181e-20	2.728e-16
6	Smooth muscle cells	*LOC100345985*	0.3013	0.417	0.250	1.505e-12	3.475e-08
6	Smooth muscle cells	*OTUD1*	0.3006	0.323	0.212	2.253e-07	5.204e-03
6	Smooth muscle cells	*ARF1*	0.3005	0.557	0.343	1.213e-14	2.800e-10
6	Smooth muscle cells	*KAZN*	0.3004	0.168	0.055	3.978e-23	9.187e-19
6	Smooth muscle cells	*GTF2IRD1*	0.2998	0.188	0.065	8.792e-23	2.030e-18
6	Smooth muscle cells	*LOC138844589*	0.2998	0.410	0.238	4.793e-15	1.107e-10
6	Smooth muscle cells	*ITGA6*	0.2995	0.190	0.048	1.577e-37	3.642e-33
6	Smooth muscle cells	*PLCB1*	0.2995	0.603	0.388	5.768e-18	1.332e-13
6	Smooth muscle cells	*SPSB3*	0.2992	0.402	0.210	1.527e-17	3.526e-13
6	Smooth muscle cells	*MT3*	0.2981	0.146	0.019	1.498e-64	3.458e-60

Original cluster	Annotated cell type	Gene	avg_log2FC	pct.1	pct.2	Raw P value	Bonferroni-adjusted P value
6	Smooth muscle cells	*SNW1*	0.2981	0.500	0.324	1.018e-11	2.351e-07
6	Smooth muscle cells	*NABP1*	0.2980	0.378	0.206	6.701e-16	1.547e-11
6	Smooth muscle cells	*ENTPD1*	0.2965	0.192	0.066	2.316e-22	5.348e-18
6	Smooth muscle cells	*LOC138850269*	0.2964	0.301	0.139	2.753e-19	6.357e-15
6	Smooth muscle cells	*RSU1*	0.2957	0.522	0.319	1.867e-15	4.311e-11
6	Smooth muscle cells	*PGK1*	0.2955	0.437	0.240	1.776e-16	4.102e-12
6	Smooth muscle cells	*SBSPON*	0.2946	0.131	0.014	1.288e-67	2.974e-63
6	Smooth muscle cells	*EVA1B*	0.2943	0.421	0.224	1.895e-17	4.375e-13
6	Smooth muscle cells	*SIAH1*	0.2932	0.362	0.219	6.984e-12	1.613e-07
6	Smooth muscle cells	*EIF5B*	0.2924	0.448	0.266	3.835e-13	8.856e-09
6	Smooth muscle cells	*GRIA3*	0.2924	0.133	0.029	2.775e-32	6.407e-28
6	Smooth muscle cells	*COX7B*	0.2917	0.410	0.235	1.382e-14	3.192e-10
6	Smooth muscle cells	*C6H1orf35*	0.2914	0.546	0.359	2.243e-12	5.181e-08
6	Smooth muscle cells	*TAGLN2*	0.2910	0.570	0.406	1.073e-09	2.479e-05
6	Smooth muscle cells	*RB1*	0.2905	0.443	0.265	2.678e-14	6.183e-10
6	Smooth muscle cells	*CAPSL*	0.2905	0.114	0.001	6.851e-192	1.582e-187
6	Smooth muscle cells	*TPST1*	0.2903	0.275	0.145	9.471e-13	2.187e-08
6	Smooth muscle cells	*ECH1*	0.2898	0.236	0.081	6.929e-28	1.600e-23
6	Smooth muscle cells	*CD82*	0.2897	0.262	0.134	1.140e-13	2.634e-09
6	Smooth muscle cells	*ARL5A*	0.2894	0.605	0.407	3.395e-12	7.840e-08
6	Smooth muscle cells	*CYTH2*	0.2893	0.275	0.126	3.457e-18	7.983e-14
6	Smooth muscle cells	*LOC103346524*	0.2885	0.306	0.152	8.913e-17	2.058e-12
5	Smooth muscle cells	*CD44*	0.2881	0.118	0.056	7.833e-10	1.809e-05
6	Smooth muscle cells	*HNRNPA3*	0.2877	0.948	0.860	6.303e-14	1.456e-09
6	Smooth muscle cells	*RAB2A*	0.2873	0.688	0.480	4.666e-13	1.077e-08
6	Smooth muscle cells	*IL1R1*	0.2866	0.138	0.020	5.114e-54	1.181e-49
6	Smooth muscle cells	*HS3ST1*	0.2864	0.162	0.036	9.910e-39	2.289e-34
6	Smooth muscle cells	*DNAJC7*	0.2864	0.391	0.193	2.516e-20	5.811e-16
6	Smooth muscle cells	*FAM3C*	0.2859	0.419	0.237	2.606e-15	6.018e-11
6	Smooth muscle cells	*LOC138848813*	0.2851	0.655	0.475	3.345e-10	7.724e-06
6	Smooth muscle cells	*PPARD*	0.2851	0.212	0.072	1.516e-25	3.502e-21
5	Smooth muscle cells	*CBL*	0.2848	0.129	0.077	1.463e-06	3.378e-02
6	Smooth muscle cells	*CHMP2A*	0.2846	0.467	0.296	1.247e-11	2.879e-07
6	Smooth muscle cells	*BZW1*	0.2846	0.459	0.278	1.181e-13	2.727e-09
6	Smooth muscle cells	*MYLK4*	0.2843	0.146	0.031	3.420e-37	7.897e-33
6	Smooth muscle cells	*GDNF*	0.2839	0.122	0.017	3.536e-50	8.165e-46
6	Smooth muscle cells	*LOC138843475*	0.2837	0.934	0.793	3.482e-14	8.041e-10
6	Smooth muscle cells	*DNAJB2*	0.2835	0.238	0.090	2.249e-23	5.193e-19
6	Smooth muscle cells	*C3H2orf69*	0.2833	0.157	0.044	1.419e-27	3.277e-23
6	Smooth muscle cells	*BBLN*	0.2829	0.240	0.096	4.312e-22	9.958e-18

Original cluster	Annotated cell type	Gene	avg_log2FC	pct.1	pct.2	Raw P value	Bonferroni-adjusted P value
6	Smooth muscle cells	*ANTKMT*	0.2828	0.207	0.071	1.892e-25	4.370e-21
6	Smooth muscle cells	*MVP*	0.2827	0.245	0.098	1.063e-21	2.454e-17
6	Smooth muscle cells	*LPCAT1*	0.2820	0.207	0.070	4.128e-26	9.532e-22
6	Smooth muscle cells	*PPM1G*	0.2818	0.295	0.141	9.370e-18	2.164e-13
6	Smooth muscle cells	*LOC127489568*	0.2817	0.478	0.318	4.107e-10	9.483e-06
6	Smooth muscle cells	*MARVELD1*	0.2816	0.286	0.140	6.495e-16	1.500e-11
6	Smooth muscle cells	*HSPA12A*	0.2815	0.148	0.025	6.751e-49	1.559e-44
6	Smooth muscle cells	*ATP5MF*	0.2812	0.465	0.282	8.636e-14	1.994e-09
6	Smooth muscle cells	*CDH11*	0.2803	0.454	0.262	3.254e-15	7.515e-11
6	Smooth muscle cells	*UQCRC1*	0.2801	0.266	0.120	1.067e-18	2.464e-14
6	Smooth muscle cells	*KEF51-p04*	0.2799	0.996	0.989	8.940e-23	2.064e-18
6	Smooth muscle cells	*TUBA4A*	0.2799	0.142	0.036	1.284e-28	2.966e-24
6	Smooth muscle cells	*EEF1A1*	0.2798	0.998	0.990	5.649e-27	1.304e-22
6	Smooth muscle cells	*PSMG3*	0.2795	0.260	0.104	4.533e-23	1.047e-18
6	Smooth muscle cells	*ATP11B*	0.2792	0.354	0.182	6.367e-18	1.470e-13
5	Smooth muscle cells	*RAP2A*	0.2791	0.113	0.058	3.955e-08	9.133e-04
6	Smooth muscle cells	*MON2*	0.2785	0.384	0.219	2.127e-13	4.913e-09
6	Smooth muscle cells	*KLHL15*	0.2780	0.264	0.126	2.080e-16	4.803e-12
6	Smooth muscle cells	*MICOS13*	0.2774	0.441	0.271	1.127e-11	2.602e-07
6	Smooth muscle cells	*DEPP1*	0.2772	0.199	0.064	1.005e-26	2.322e-22
6	Smooth muscle cells	*MFAP3L*	0.2771	0.127	0.004	1.734e-150	4.005e-146
6	Smooth muscle cells	*MIF4GD*	0.2770	0.341	0.173	6.867e-17	1.586e-12
6	Smooth muscle cells	*RPL15*	0.2767	0.919	0.798	1.261e-11	2.912e-07
6	Smooth muscle cells	*HNRNPR*	0.2766	0.646	0.444	5.819e-12	1.344e-07
6	Smooth muscle cells	*LOC138848792*	0.2765	0.107	0.001	2.314e-179	5.342e-175
6	Smooth muscle cells	*FBXO15*	0.2762	0.144	0.026	2.353e-43	5.433e-39
6	Smooth muscle cells	*CCT3*	0.2759	0.356	0.196	2.275e-14	5.253e-10
6	Smooth muscle cells	*ACADL*	0.2757	0.349	0.176	2.532e-17	5.846e-13
6	Smooth muscle cells	*ACADVL*	0.2756	0.223	0.078	1.255e-25	2.898e-21
6	Smooth muscle cells	*SLC5A3*	0.2746	0.306	0.155	1.005e-15	2.321e-11
6	Smooth muscle cells	*EIF1AX*	0.2743	0.491	0.306	2.008e-12	4.637e-08
6	Smooth muscle cells	*EIF4A2*	0.2742	0.928	0.821	2.081e-12	4.806e-08
6	Smooth muscle cells	*FMNL3*	0.2742	0.138	0.030	5.164e-34	1.193e-29
6	Smooth muscle cells	*HSPA2*	0.2739	0.566	0.383	1.166e-11	2.694e-07
6	Smooth muscle cells	*FBXL2*	0.2738	0.120	0.006	2.073e-105	4.787e-101
5	Smooth muscle cells	*IDH3A*	0.2737	0.117	0.058	4.851e-09	1.120e-04
6	Smooth muscle cells	*EPHX3*	0.2734	0.105	0.005	1.234e-92	2.850e-88
6	Smooth muscle cells	*NRP2*	0.2723	0.192	0.090	9.540e-13	2.203e-08
6	Smooth muscle cells	*SLC39A1*	0.2718	0.448	0.267	8.916e-13	2.059e-08
6	Smooth muscle cells	*EHBP1L1*	0.2717	0.140	0.021	5.464e-52	1.262e-47

Original cluster	Annotated cell type	Gene	avg_log2FC	pct.1	pct.2	Raw P value	Bonferroni-adjusted P value
6	Smooth muscle cells	*ITPRIPL1*	0.2716	0.131	0.014	7.978e-68	1.842e-63
6	Smooth muscle cells	*CAPZA2*	0.2715	0.747	0.552	1.154e-11	2.666e-07
6	Smooth muscle cells	*BACE2*	0.2714	0.236	0.120	9.940e-13	2.295e-08
6	Smooth muscle cells	*ARPC5*	0.2711	0.485	0.293	4.563e-14	1.054e-09
6	Smooth muscle cells	*BTAF1*	0.2706	0.295	0.174	2.199e-10	5.077e-06
5	Smooth muscle cells	*NRM*	0.2702	0.113	0.064	1.143e-06	2.639e-02
6	Smooth muscle cells	*RABGAP1L*	0.2696	0.550	0.359	4.640e-12	1.072e-07
6	Smooth muscle cells	*COPZ2*	0.2691	0.194	0.060	1.886e-27	4.356e-23
6	Smooth muscle cells	*DNAJC12*	0.2685	0.166	0.049	6.896e-26	1.593e-21
6	Smooth muscle cells	*ARC*	0.2683	0.140	0.064	9.021e-10	2.083e-05
6	Smooth muscle cells	*MED13*	0.2681	0.583	0.393	4.859e-12	1.122e-07
6	Smooth muscle cells	*STK38*	0.2678	0.279	0.135	4.417e-16	1.020e-11
6	Smooth muscle cells	*COL18A1*	0.2671	0.201	0.067	3.402e-25	7.857e-21
6	Smooth muscle cells	*PLK3*	0.2669	0.131	0.027	1.966e-34	4.541e-30
6	Smooth muscle cells	*SNTB2*	0.2668	0.334	0.187	2.273e-12	5.249e-08
6	Smooth muscle cells	*ANKRD55*	0.2668	0.135	0.009	1.709e-101	3.947e-97
6	Smooth muscle cells	*HSPA9*	0.2663	0.410	0.219	4.883e-17	1.128e-12
6	Smooth muscle cells	*HSD17B12*	0.2661	0.319	0.180	4.951e-12	1.143e-07
6	Smooth muscle cells	*CIRBP*	0.2660	0.450	0.281	1.796e-11	4.146e-07
6	Smooth muscle cells	*LOC100344472*	0.2656	0.517	0.349	3.734e-10	8.623e-06
6	Smooth muscle cells	*FAM162A*	0.2646	0.229	0.111	6.282e-14	1.451e-09
6	Smooth muscle cells	*PHF1*	0.2641	0.384	0.224	1.590e-12	3.671e-08
6	Smooth muscle cells	*LOC108176480*	0.2639	0.122	0.011	1.532e-72	3.538e-68
6	Smooth muscle cells	*REEP3*	0.2639	0.450	0.265	4.986e-14	1.151e-09
6	Smooth muscle cells	*CAPZB*	0.2635	0.537	0.347	4.764e-12	1.100e-07
6	Smooth muscle cells	*HARBI1*	0.2624	0.194	0.072	1.566e-20	3.617e-16
6	Smooth muscle cells	*RP9*	0.2622	0.321	0.170	2.403e-14	5.549e-10
6	Smooth muscle cells	*EIF4B*	0.2621	0.662	0.467	6.890e-11	1.591e-06
6	Smooth muscle cells	*PPP4R3A*	0.2618	0.415	0.261	3.412e-11	7.879e-07
6	Smooth muscle cells	*LARP1B*	0.2616	0.258	0.126	6.909e-15	1.596e-10
6	Smooth muscle cells	*SERBP1*	0.2616	0.803	0.615	3.711e-11	8.570e-07
6	Smooth muscle cells	*ING3*	0.2611	0.229	0.105	3.401e-15	7.853e-11
6	Smooth muscle cells	*DCUN1D1*	0.2610	0.264	0.126	6.143e-16	1.418e-11
6	Smooth muscle cells	*UBQLN1*	0.2606	0.365	0.192	7.601e-16	1.755e-11
6	Smooth muscle cells	*ABR*	0.2606	0.199	0.073	3.852e-21	8.895e-17
6	Smooth muscle cells	*ACSL4*	0.2601	0.214	0.077	6.232e-24	1.439e-19
6	Smooth muscle cells	*LOC100345355*	0.2600	0.607	0.412	8.915e-11	2.059e-06
6	Smooth muscle cells	*CCN3*	0.2598	0.118	0.037	3.267e-17	7.543e-13
6	Smooth muscle cells	*ENDOD1*	0.2598	0.245	0.083	6.435e-29	1.486e-24
6	Smooth muscle cells	*AURKAIP1*	0.2598	0.611	0.431	1.540e-09	3.556e-05

Original cluster	Annotated cell type	Gene	avg_log2FC	pct.1	pct.2	Raw P value	Bonferroni-adjusted P value
6	Smooth muscle cells	*AKIRIN1*	0.2598	0.214	0.085	1.067e-19	2.465e-15
6	Smooth muscle cells	*EIF3D*	0.2598	0.334	0.174	1.595e-15	3.683e-11
6	Smooth muscle cells	*SUCLA2*	0.2597	0.310	0.164	2.697e-14	6.228e-10
6	Smooth muscle cells	*RHEB*	0.2595	0.633	0.464	5.242e-09	1.211e-04
6	Smooth muscle cells	*MRPS17*	0.2594	0.299	0.166	5.825e-12	1.345e-07
6	Smooth muscle cells	*BNIP3*	0.2591	0.474	0.347	1.750e-06	4.042e-02
6	Smooth muscle cells	*AGFG1*	0.2578	0.382	0.228	5.249e-12	1.212e-07
6	Smooth muscle cells	*RBM7*	0.2576	0.362	0.210	6.051e-12	1.397e-07
6	Smooth muscle cells	*TNK2*	0.2576	0.177	0.056	8.700e-25	2.009e-20
6	Smooth muscle cells	*MCRIP2*	0.2571	0.155	0.062	1.573e-14	3.632e-10
6	Smooth muscle cells	*BEND7*	0.2570	0.172	0.065	7.358e-18	1.699e-13
6	Smooth muscle cells	*LOC100358069*	0.2567	0.817	0.656	2.266e-09	5.232e-05
6	Smooth muscle cells	*HSPA13*	0.2566	0.179	0.061	1.579e-22	3.646e-18
6	Smooth muscle cells	*ADARB1*	0.2564	0.203	0.077	2.334e-20	5.389e-16
6	Smooth muscle cells	*EPN2*	0.2560	0.251	0.100	3.780e-22	8.728e-18
6	Smooth muscle cells	*OAT*	0.2559	0.557	0.389	1.838e-09	4.244e-05
6	Smooth muscle cells	*EGR3*	0.2555	0.107	0.031	2.051e-18	4.737e-14
6	Smooth muscle cells	*GNB1*	0.2555	0.620	0.421	2.707e-11	6.250e-07
6	Smooth muscle cells	*COX5B*	0.2549	0.793	0.620	2.110e-09	4.872e-05
6	Smooth muscle cells	*AARS1*	0.2544	0.249	0.100	1.870e-21	4.318e-17
6	Smooth muscle cells	*STK11*	0.2543	0.349	0.168	4.162e-19	9.612e-15
6	Smooth muscle cells	*PABPC4*	0.2543	0.400	0.234	3.553e-12	8.205e-08
6	Smooth muscle cells	*PSMA2*	0.2540	0.439	0.290	3.405e-10	7.862e-06
6	Smooth muscle cells	*PPFIBP2*	0.2538	0.133	0.027	4.672e-36	1.079e-31
6	Smooth muscle cells	*MARCHF3*	0.2536	0.207	0.070	3.440e-26	7.944e-22
6	Smooth muscle cells	*IRF2BP2*	0.2534	0.526	0.363	1.468e-09	3.390e-05
6	Smooth muscle cells	*MAP7D1*	0.2534	0.229	0.084	3.354e-24	7.746e-20
6	Smooth muscle cells	*CTNNB1*	0.2533	0.740	0.493	2.693e-16	6.218e-12
6	Smooth muscle cells	*IGSF5*	0.2529	0.116	0.012	1.783e-64	4.117e-60
6	Smooth muscle cells	*PSMD8*	0.2527	0.493	0.313	2.120e-11	4.895e-07
6	Smooth muscle cells	*SCLT1*	0.2526	0.251	0.135	7.000e-11	1.616e-06
6	Smooth muscle cells	*SLC44A2*	0.2521	0.260	0.119	1.014e-16	2.342e-12
6	Smooth muscle cells	*LOC100354169*	0.2519	0.299	0.155	2.844e-14	6.567e-10
6	Smooth muscle cells	*LRIG1*	0.2514	0.177	0.062	9.756e-21	2.253e-16
6	Smooth muscle cells	*AKIRIN2*	0.2511	0.288	0.149	1.494e-13	3.451e-09
6	Smooth muscle cells	*ATG101*	0.2511	0.299	0.156	4.904e-14	1.132e-09
6	Smooth muscle cells	*RASSF9*	0.2510	0.105	0.023	1.546e-26	3.571e-22
6	Smooth muscle cells	*PKIG*	0.2510	0.255	0.116	4.548e-17	1.050e-12
6	Smooth muscle cells	*PPME1*	0.2510	0.225	0.108	1.447e-13	3.341e-09
6	Smooth muscle cells	*NDUFS8*	0.2507	0.148	0.033	1.541e-35	3.558e-31

Original cluster	Annotated cell type	Gene	avg_log2FC	pct.1	pct.2	Raw P value	Bonferroni-adjusted P value
6	Smooth muscle cells	*MRPL54*	0.2507	0.341	0.189	8.968e-13	2.071e-08
6	Smooth muscle cells	*ETF1*	0.2506	0.308	0.160	8.143e-15	1.880e-10
6	Smooth muscle cells	*AKT1*	0.2501	0.310	0.160	7.210e-15	1.665e-10
7	**Blood vascular endothelial cells**	*LOC103351679*	4.5889	0.991	0.057	0	0
7	Blood vascular endothelial cells	*CLEC3B*	3.7879	0.994	0.231	0	0
7	Blood vascular endothelial cells	*EMCN*	3.6966	0.982	0.069	0	0
7	Blood vascular endothelial cells	*ADGRL4*	3.6463	0.955	0.008	0	0
7	Blood vascular endothelial cells	*GNG11*	3.5294	0.979	0.338	1.022e-257	2.360e-253
7	Blood vascular endothelial cells	*ABCB1*	3.3544	0.919	0.021	0	0
7	Blood vascular endothelial cells	*RAMP2*	3.1914	0.976	0.193	0	0
7	Blood vascular endothelial cells	*EGFL7*	3.1510	0.955	0.013	0	0
7	Blood vascular endothelial cells	*CAV1*	3.1321	0.985	0.225	0	0
7	Blood vascular endothelial cells	*ENTPD1*	2.8392	0.880	0.042	0	0
7	Blood vascular endothelial cells	*FLT1*	2.8311	0.852	0.006	0	0
7	Blood vascular endothelial cells	*MECOM*	2.8238	0.889	0.024	0	0
7	Blood vascular endothelial cells	*CYYR1*	2.8064	0.901	0.006	0	0
7	Blood vascular endothelial cells	*GRO-A*	2.7863	0.479	0.025	0	0
7	Blood vascular endothelial cells	*SOX18*	2.7683	0.831	0.010	0	0
7	Blood vascular endothelial cells	*LEPR*	2.7130	0.867	0.042	0	0
7	Blood vascular endothelial cells	*GPIHBP1*	2.6780	0.777	0.007	0	0
7	Blood vascular endothelial cells	*ADGRF5*	2.6633	0.907	0.035	0	0
7	Blood vascular endothelial cells	*PLCB4*	2.6625	0.849	0.062	0	0
7	Blood vascular endothelial cells	*SOX17*	2.5995	0.729	0.014	0	0
7	Blood vascular endothelial cells	*LMO2*	2.5621	0.889	0.024	0	0
7	Blood vascular endothelial cells	*LOC100358984*	2.5466	0.744	0.003	0	0
7	Blood vascular endothelial cells	*RHOJ*	2.5355	0.919	0.204	5.970e-274	1.379e-269
7	Blood vascular endothelial cells	*PLVAP*	2.4021	0.762	0.006	0	0
7	Blood vascular endothelial cells	*RASAL2*	2.3723	0.883	0.116	0	0
7	Blood vascular endothelial cells	*VWF*	2.3418	0.810	0.020	0	0
7	Blood vascular endothelial cells	*PRKCH*	2.3136	0.834	0.039	0	0
7	Blood vascular endothelial cells	*CCSER1*	2.2669	0.789	0.115	2.321e-282	5.361e-278
7	Blood vascular endothelial cells	*CD34*	2.2621	0.801	0.070	0	0
7	Blood vascular endothelial cells	*MAGI1*	2.2555	0.810	0.110	0	0
7	Blood vascular endothelial cells	*VIM*	2.2492	1.000	0.932	6.767e-179	1.563e-174
7	Blood vascular endothelial cells	*ARL15*	2.2479	0.813	0.319	1.327e-109	3.065e-105
7	Blood vascular endothelial cells	*IL6ST*	2.2172	0.819	0.352	2.775e-113	6.408e-109
7	Blood vascular endothelial cells	*CD55*	2.2163	0.861	0.203	5.045e-217	1.165e-212
7	Blood vascular endothelial cells	*SAT1*	2.2123	0.949	0.403	1.259e-156	2.907e-152
7	Blood vascular endothelial cells	*C13H1orf115*	2.2121	0.810	0.010	0	0
7	Blood vascular endothelial cells	*LOC138846556*	2.1908	0.747	0.006	0	0

Original cluster	Annotated cell type	Gene	avg_log2FC	pct.1	pct.2	Raw P value	Bonferroni-adjusted P value
7	Blood vascular endothelial cells	*ARHGAP31*	2.1822	0.813	0.081	0	0
7	Blood vascular endothelial cells	*LDB2*	2.1609	0.979	0.529	1.769e-164	4.084e-160
7	Blood vascular endothelial cells	*BCR*	2.1562	0.684	0.046	0	0
7	Blood vascular endothelial cells	*CALCRL*	2.1505	0.934	0.326	5.991e-181	1.383e-176
7	Blood vascular endothelial cells	*TMOD3*	2.1495	0.864	0.222	3.030e-207	6.997e-203
7	Blood vascular endothelial cells	*EMP1*	2.1387	0.654	0.027	0	0
7	Blood vascular endothelial cells	*KLF2*	2.1321	0.922	0.473	7.904e-118	1.825e-113
7	Blood vascular endothelial cells	*SNRK*	2.1288	0.774	0.105	4.999e-307	1.154e-302
7	Blood vascular endothelial cells	*DNM3*	2.1234	0.804	0.100	0	0
7	Blood vascular endothelial cells	*MRTFB*	2.1199	0.889	0.200	7.508e-243	1.734e-238
7	Blood vascular endothelial cells	*CD93*	2.1168	0.807	0.027	0	0
7	Blood vascular endothelial cells	*PTPRB*	2.1138	0.789	0.015	0	0
7	Blood vascular endothelial cells	*CLDN5*	2.0981	0.738	0.031	0	0
7	Blood vascular endothelial cells	*PPP1R9A*	2.0640	0.864	0.171	1.536e-246	3.548e-242
7	Blood vascular endothelial cells	*FLI1*	2.0343	0.798	0.026	0	0
7	Blood vascular endothelial cells	*JCAD*	2.0339	0.834	0.050	0	0
7	Blood vascular endothelial cells	*PALMD*	2.0308	0.750	0.002	0	0
7	Blood vascular endothelial cells	*PLCB1*	2.0220	0.849	0.382	1.913e-100	4.417e-96
7	Blood vascular endothelial cells	*LOC100008640*	2.0191	0.877	0.182	1.443e-257	3.333e-253
7	Blood vascular endothelial cells	*CDH5*	2.0105	0.801	0.008	0	0
7	Blood vascular endothelial cells	*PECAM1*	2.0067	0.852	0.020	0	0
7	Blood vascular endothelial cells	*KANK3*	1.9916	0.807	0.010	0	0
7	Blood vascular endothelial cells	*ERG*	1.9839	0.807	0.015	0	0
7	Blood vascular endothelial cells	*SRGN*	1.9663	0.768	0.036	0	0
7	Blood vascular endothelial cells	*PODXL*	1.9594	0.657	0.013	0	0
7	Blood vascular endothelial cells	*PLAAT3*	1.9552	0.858	0.137	0	0
7	Blood vascular endothelial cells	*CD9*	1.9410	0.711	0.057	0	0
7	Blood vascular endothelial cells	*ZNF521*	1.9354	0.846	0.217	8.299e-178	1.916e-173
7	Blood vascular endothelial cells	*ACKR1*	1.9301	0.259	0.003	0	0
7	Blood vascular endothelial cells	*LOC138847739*	1.9183	0.858	0.250	7.923e-180	1.830e-175
7	Blood vascular endothelial cells	*TPD52L1*	1.9022	0.861	0.264	4.268e-172	9.857e-168
7	Blood vascular endothelial cells	*AQP1*	1.8992	0.753	0.044	0	0
7	Blood vascular endothelial cells	*THBD*	1.8935	0.711	0.031	0	0
7	Blood vascular endothelial cells	*TJP1*	1.8720	0.873	0.207	5.605e-227	1.294e-222
7	Blood vascular endothelial cells	*RASGRP3*	1.8715	0.663	0.020	0	0
7	Blood vascular endothelial cells	*PTPRM*	1.8672	0.870	0.244	8.640e-178	1.995e-173
7	Blood vascular endothelial cells	*PALM2AKAP2*	1.8652	0.810	0.114	0	0
7	Blood vascular endothelial cells	*MEF2C*	1.8515	0.837	0.107	0	0
7	Blood vascular endothelial cells	*ATP8B1*	1.8496	0.696	0.048	0	0
7	Blood vascular endothelial cells	*LIMCH1*	1.8466	0.744	0.098	5.223e-286	1.206e-281

Original cluster	Annotated cell type	Gene	avg_log2FC	pct.1	pct.2	Raw P value	Bonferroni-adjusted P value
7	Blood vascular endothelial cells	*TSPAN5*	1.8421	0.786	0.073	0	0
7	Blood vascular endothelial cells	*GARRE1*	1.8329	0.849	0.156	1.864e-267	4.304e-263
7	Blood vascular endothelial cells	*GADD45A*	1.8266	0.744	0.258	7.280e-100	1.681e-95
7	Blood vascular endothelial cells	*P3H2*	1.8253	0.708	0.046	0	0
7	Blood vascular endothelial cells	*ENG*	1.8207	0.798	0.068	0	0
7	Blood vascular endothelial cells	*BCAM*	1.8177	0.864	0.102	0	0
7	Blood vascular endothelial cells	*SCARB1*	1.8050	0.687	0.046	0	0
7	Blood vascular endothelial cells	*RFLNB*	1.8001	0.726	0.038	0	0
7	Blood vascular endothelial cells	*CD200*	1.7987	0.795	0.155	1.003e-228	2.317e-224
7	Blood vascular endothelial cells	*TCF4*	1.7925	0.991	0.758	3.319e-148	7.663e-144
7	Blood vascular endothelial cells	*FAM171A1*	1.7922	0.705	0.098	1.547e-258	3.573e-254
7	Blood vascular endothelial cells	*ZFPM2*	1.7872	0.777	0.179	6.271e-159	1.448e-154
7	Blood vascular endothelial cells	*SHANK3*	1.7491	0.747	0.010	0	0
7	Blood vascular endothelial cells	*TNFSF10*	1.7445	0.720	0.045	0	0
7	Blood vascular endothelial cells	*TSPAN13*	1.7440	0.747	0.133	6.459e-226	1.492e-221
7	Blood vascular endothelial cells	*SPARC*	1.7347	0.916	0.503	8.150e-128	1.882e-123
7	Blood vascular endothelial cells	*PTPRG*	1.7293	0.822	0.344	2.664e-101	6.152e-97
7	Blood vascular endothelial cells	*CCDC85B*	1.7246	0.807	0.111	0	0
7	Blood vascular endothelial cells	*MAGI3*	1.7235	0.756	0.253	4.329e-101	9.997e-97
7	Blood vascular endothelial cells	*CAV2*	1.7224	0.849	0.215	6.550e-196	1.512e-191
7	Blood vascular endothelial cells	*PLEKHG1*	1.6878	0.720	0.060	0	0
7	Blood vascular endothelial cells	*IRAK2*	1.6874	0.654	0.096	4.300e-229	9.929e-225
7	Blood vascular endothelial cells	*ZNF385B*	1.6871	0.494	0.010	0	0
7	Blood vascular endothelial cells	*SMAD1*	1.6797	0.795	0.183	4.247e-183	9.807e-179
7	Blood vascular endothelial cells	*TM4SF1*	1.6742	0.684	0.048	0	0
7	Blood vascular endothelial cells	*DACH1*	1.6742	0.843	0.250	2.541e-138	5.867e-134
7	Blood vascular endothelial cells	*DTL*	1.6696	0.596	0.009	0	0
7	Blood vascular endothelial cells	*LOC100349085*	1.6639	0.883	0.282	7.493e-161	1.730e-156
7	Blood vascular endothelial cells	*SDCBP*	1.6444	0.943	0.630	4.492e-129	1.037e-124
7	Blood vascular endothelial cells	*ADAMTS9*	1.6384	0.419	0.041	6.391e-195	1.476e-190
7	Blood vascular endothelial cells	*LOC127491011*	1.6369	0.696	0.039	0	0
7	Blood vascular endothelial cells	*CAVIN2*	1.6321	0.693	0.035	0	0
7	Blood vascular endothelial cells	*WWTR1*	1.6315	0.907	0.318	1.666e-149	3.848e-145
7	Blood vascular endothelial cells	*ICAM2*	1.6252	0.723	0.016	0	0
7	Blood vascular endothelial cells	*ECSCR*	1.6217	0.759	0.010	0	0
7	Blood vascular endothelial cells	*TMEM88*	1.6173	0.645	0.010	0	0
7	Blood vascular endothelial cells	*NPDC1*	1.6153	0.801	0.103	0	0
7	Blood vascular endothelial cells	*ESAM*	1.6074	0.753	0.044	0	0
7	Blood vascular endothelial cells	*B2M*	1.6049	0.985	0.874	2.103e-136	4.857e-132
7	Blood vascular endothelial cells	*TEK*	1.6038	0.672	0.009	0	0

Original cluster	Annotated cell type	Gene	avg_log2FC	pct.1	pct.2	Raw P value	Bonferroni-adjusted P value
7	Blood vascular endothelial cells	*CRACR2B*	1.5954	0.714	0.020	0	0
7	Blood vascular endothelial cells	*ELMO1*	1.5931	0.774	0.091	0	0
7	Blood vascular endothelial cells	*RAPGEF4*	1.5915	0.608	0.012	0	0
7	Blood vascular endothelial cells	*SELENOP*	1.5840	0.955	0.800	4.737e-87	1.094e-82
7	Blood vascular endothelial cells	*PKP4*	1.5811	0.795	0.240	7.381e-134	1.704e-129
7	Blood vascular endothelial cells	*LOC100328967*	1.5780	0.861	0.258	1.907e-157	4.405e-153
7	Blood vascular endothelial cells	*BST2*	1.5701	0.846	0.293	1.193e-127	2.754e-123
7	Blood vascular endothelial cells	*LOC138844296*	1.5697	0.726	0.060	0	0
7	Blood vascular endothelial cells	*LIFR*	1.5617	0.735	0.070	0	0
7	Blood vascular endothelial cells	*LOC100125981*	1.5573	0.735	0.226	2.984e-122	6.890e-118
7	Blood vascular endothelial cells	*HIF1A*	1.5564	0.714	0.175	8.809e-147	2.034e-142
7	Blood vascular endothelial cells	*ELK3*	1.5444	0.771	0.096	0	0
7	Blood vascular endothelial cells	*LRRC8C*	1.5325	0.620	0.042	0	0
7	Blood vascular endothelial cells	*LOC100340954*	1.5250	0.898	0.438	2.658e-112	6.139e-108
7	Blood vascular endothelial cells	*ST6GAL1*	1.5221	0.623	0.050	0	0
7	Blood vascular endothelial cells	*LOC100354804*	1.5035	0.358	0.013	0	0
7	Blood vascular endothelial cells	*SOX7*	1.5017	0.620	0.014	0	0
7	Blood vascular endothelial cells	*ADAM10*	1.4888	0.822	0.278	3.565e-126	8.231e-122
7	Blood vascular endothelial cells	*SOCS3*	1.4865	0.901	0.600	1.652e-80	3.814e-76
7	Blood vascular endothelial cells	*PLXND1*	1.4861	0.744	0.077	0	0
7	Blood vascular endothelial cells	*TFPI*	1.4825	0.780	0.206	5.532e-145	1.278e-140
7	Blood vascular endothelial cells	*ENPP3*	1.4780	0.596	0.007	0	0
7	Blood vascular endothelial cells	*CDK17*	1.4687	0.747	0.150	1.804e-187	4.165e-183
7	Blood vascular endothelial cells	*CAVIN1*	1.4681	0.825	0.237	1.145e-152	2.644e-148
7	Blood vascular endothelial cells	*EDN1*	1.4574	0.274	0.001	0	0
7	Blood vascular endothelial cells	*SELE*	1.4484	0.250	0.001	0	0
7	Blood vascular endothelial cells	*DOCK9*	1.4457	0.804	0.182	1.042e-177	2.405e-173
7	Blood vascular endothelial cells	*ARL5B*	1.4448	0.696	0.168	2.594e-135	5.990e-131
7	Blood vascular endothelial cells	*YES1*	1.4435	0.807	0.211	9.839e-161	2.272e-156
7	Blood vascular endothelial cells	*NEDD9*	1.4428	0.678	0.097	5.492e-231	1.268e-226
7	Blood vascular endothelial cells	*NFIB*	1.4362	0.880	0.348	1.631e-115	3.766e-111
7	Blood vascular endothelial cells	*THSD7A*	1.4361	0.581	0.038	0	0
7	Blood vascular endothelial cells	*ITPR2*	1.4326	0.765	0.249	1.772e-114	4.092e-110
7	Blood vascular endothelial cells	*PGER5*	1.4305	0.825	0.273	9.791e-133	2.261e-128
7	Blood vascular endothelial cells	*ACE*	1.4301	0.557	0.005	0	0
7	Blood vascular endothelial cells	*COL4A1*	1.4202	0.735	0.188	7.371e-134	1.702e-129
7	Blood vascular endothelial cells	*RALGAPA1*	1.4179	0.858	0.445	6.061e-81	1.400e-76
7	Blood vascular endothelial cells	*ST8SIA4*	1.4168	0.651	0.058	0	0
7	Blood vascular endothelial cells	*DOCK4*	1.4124	0.642	0.085	2.598e-230	5.999e-226
7	Blood vascular endothelial cells	*NR5A2*	1.4118	0.596	0.002	0	0

Original cluster	Annotated cell type	Gene	avg_log2FC	pct.1	pct.2	Raw P value	Bonferroni-adjusted P value
7	Blood vascular endothelial cells	*ETS1*	1.4110	0.660	0.104	3.831e-204	8.847e-200
7	Blood vascular endothelial cells	*CRIM1*	1.4025	0.870	0.430	1.024e-76	2.365e-72
7	Blood vascular endothelial cells	*B3GNT5*	1.3951	0.515	0.031	0	0
7	Blood vascular endothelial cells	*ANXA2*	1.3906	0.777	0.153	8.282e-195	1.912e-190
7	Blood vascular endothelial cells	*ADAMTS1*	1.3830	0.687	0.294	1.999e-61	4.617e-57
7	Blood vascular endothelial cells	*SEMA6A*	1.3770	0.705	0.070	0	0
7	Blood vascular endothelial cells	*KLF6*	1.3761	0.867	0.490	9.900e-63	2.286e-58
7	Blood vascular endothelial cells	*NUAK1*	1.3707	0.569	0.024	0	0
7	Blood vascular endothelial cells	*TMSB10*	1.3690	0.994	0.959	2.004e-136	4.628e-132
7	Blood vascular endothelial cells	*MACF1*	1.3687	0.916	0.469	4.576e-116	1.057e-111
7	Blood vascular endothelial cells	*ITGA6*	1.3684	0.596	0.035	0	0
7	Blood vascular endothelial cells	*CRYBG3*	1.3660	0.623	0.081	1.299e-230	3.000e-226
7	Blood vascular endothelial cells	*MGAT4A*	1.3623	0.605	0.062	6.272e-279	1.448e-274
7	Blood vascular endothelial cells	*CTNNAL1*	1.3597	0.605	0.095	1.790e-186	4.134e-182
7	Blood vascular endothelial cells	*SPTBN1*	1.3595	0.952	0.651	3.926e-97	9.066e-93
7	Blood vascular endothelial cells	*PDE4D*	1.3571	0.669	0.164	1.671e-119	3.860e-115
7	Blood vascular endothelial cells	*RNF122*	1.3561	0.584	0.091	5.883e-176	1.359e-171
7	Blood vascular endothelial cells	*MSN*	1.3547	0.780	0.188	7.212e-164	1.666e-159
7	Blood vascular endothelial cells	*MFNG*	1.3544	0.711	0.024	0	0
7	Blood vascular endothelial cells	*GALNT18*	1.3505	0.623	0.056	0	0
7	Blood vascular endothelial cells	*MTARC2*	1.3475	0.867	0.308	2.800e-130	6.465e-126
7	Blood vascular endothelial cells	*ACKR3*	1.3461	0.524	0.182	6.392e-60	1.476e-55
7	Blood vascular endothelial cells	*HES1*	1.3455	0.590	0.167	8.593e-88	1.984e-83
7	Blood vascular endothelial cells	*EPB41*	1.3431	0.801	0.241	8.745e-125	2.019e-120
7	Blood vascular endothelial cells	*SLC39A8*	1.3378	0.605	0.014	0	0
7	Blood vascular endothelial cells	*GPM6A*	1.3280	0.404	0.108	5.750e-60	1.328e-55
7	Blood vascular endothelial cells	*BANK1*	1.3239	0.557	0.022	0	0
7	Blood vascular endothelial cells	*KDR*	1.3204	0.584	0.019	0	0
7	Blood vascular endothelial cells	*ABLIM1*	1.3188	0.765	0.249	1.011e-113	2.335e-109
7	Blood vascular endothelial cells	*ROBO4*	1.3148	0.627	0.005	0	0
7	Blood vascular endothelial cells	*LOC103351793*	1.3130	0.452	0.098	6.889e-91	1.591e-86
7	Blood vascular endothelial cells	*LOC138844494*	1.3106	0.437	0.029	2.154e-273	4.974e-269
7	Blood vascular endothelial cells	*LOC103348410*	1.3094	0.494	0.001	0	0
7	Blood vascular endothelial cells	*LOC103350144*	1.3051	0.539	0.000	0	0
7	Blood vascular endothelial cells	*PIK3R3*	1.3048	0.524	0.139	3.973e-84	9.175e-80
7	Blood vascular endothelial cells	*LOC103348788*	1.3002	0.642	0.078	3.699e-247	8.541e-243
7	Blood vascular endothelial cells	*RAP1B*	1.2998	0.720	0.173	1.213e-145	2.802e-141
7	Blood vascular endothelial cells	*TNFAIP3*	1.2958	0.404	0.062	6.602e-119	1.525e-114
7	Blood vascular endothelial cells	*MCF2L*	1.2945	0.560	0.004	0	0
7	Blood vascular endothelial cells	*IRF1*	1.2931	0.795	0.360	3.397e-82	7.844e-78

Original cluster	Annotated cell type	Gene	avg_log2FC	pct.1	pct.2	Raw P value	Bonferroni-adjusted P value
7	Blood vascular endothelial cells	*FGD6*	1.2903	0.602	0.040	0	0
7	Blood vascular endothelial cells	*PPFIBP1*	1.2890	0.753	0.152	1.106e-181	2.554e-177
7	Blood vascular endothelial cells	*JMJD1C*	1.2859	0.925	0.697	5.628e-60	1.300e-55
7	Blood vascular endothelial cells	*IPO11*	1.2802	0.696	0.159	2.290e-140	5.289e-136
7	Blood vascular endothelial cells	*EFNA1*	1.2792	0.620	0.039	0	0
7	Blood vascular endothelial cells	*RASIP1*	1.2787	0.623	0.011	0	0
7	Blood vascular endothelial cells	*EBF1*	1.2779	0.816	0.151	3.530e-195	8.151e-191
7	Blood vascular endothelial cells	*ATF3*	1.2765	0.880	0.498	7.165e-62	1.655e-57
7	Blood vascular endothelial cells	*APOLD1*	1.2736	0.536	0.155	5.493e-77	1.268e-72
7	Blood vascular endothelial cells	*ETS2*	1.2690	0.708	0.156	7.882e-149	1.820e-144
7	Blood vascular endothelial cells	*S100A16*	1.2682	0.825	0.250	5.267e-138	1.216e-133
7	Blood vascular endothelial cells	*LY6E*	1.2643	0.741	0.219	1.017e-110	2.347e-106
7	Blood vascular endothelial cells	*RIGI*	1.2587	0.663	0.223	1.043e-79	2.409e-75
7	Blood vascular endothelial cells	*RAPGEF2*	1.2582	0.756	0.266	1.109e-93	2.561e-89
7	Blood vascular endothelial cells	*SLC39A10*	1.2482	0.783	0.287	3.655e-95	8.440e-91
7	Blood vascular endothelial cells	*ADGRG1*	1.2436	0.578	0.004	0	0
7	Blood vascular endothelial cells	*MPZL2*	1.2408	0.636	0.035	0	0
7	Blood vascular endothelial cells	*LOC138843459*	1.2387	0.545	0.045	1.356e-297	3.130e-293
7	Blood vascular endothelial cells	*SCHIP1*	1.2336	0.581	0.068	5.435e-230	1.255e-225
7	Blood vascular endothelial cells	*STIM2*	1.2299	0.684	0.127	7.746e-174	1.789e-169
7	Blood vascular endothelial cells	*RALGAPA2*	1.2295	0.602	0.181	2.938e-80	6.785e-76
7	Blood vascular endothelial cells	*CHN1*	1.2287	0.738	0.255	5.784e-90	1.336e-85
7	Blood vascular endothelial cells	*FNBP1L*	1.2271	0.783	0.258	1.279e-103	2.953e-99
7	Blood vascular endothelial cells	*STT3B*	1.2248	0.765	0.212	1.280e-126	2.955e-122
7	Blood vascular endothelial cells	*LCORL*	1.2227	0.819	0.377	1.003e-72	2.315e-68
7	Blood vascular endothelial cells	*FAM110D*	1.2183	0.539	0.075	9.819e-183	2.267e-178
7	Blood vascular endothelial cells	*CST3*	1.2158	0.840	0.361	2.034e-96	4.697e-92
7	Blood vascular endothelial cells	*CLU*	1.2146	0.702	0.235	8.544e-87	1.973e-82
7	Blood vascular endothelial cells	*MYH9*	1.2130	0.747	0.200	5.786e-130	1.336e-125
7	Blood vascular endothelial cells	*TF*	1.2108	0.590	0.024	0	0
7	Blood vascular endothelial cells	*UBC*	1.2042	0.979	0.948	3.185e-83	7.354e-79
7	Blood vascular endothelial cells	*SRGAP2*	1.2035	0.780	0.288	3.145e-95	7.261e-91
7	Blood vascular endothelial cells	*S1PR1*	1.2005	0.515	0.009	0	0
7	Blood vascular endothelial cells	*LOC100343802*	1.1945	0.407	0.259	5.125e-11	1.183e-06
7	Blood vascular endothelial cells	*EPAS1*	1.1932	0.940	0.512	6.431e-96	1.485e-91
7	Blood vascular endothelial cells	*MYCT1*	1.1915	0.563	0.003	0	0
7	Blood vascular endothelial cells	*CHP2*	1.1851	0.355	0.007	0	0
7	Blood vascular endothelial cells	*RLA-A3*	1.1826	0.877	0.439	7.485e-87	1.728e-82
7	Blood vascular endothelial cells	*SNED1*	1.1814	0.729	0.229	1.831e-99	4.228e-95
7	Blood vascular endothelial cells	*CREM*	1.1808	0.627	0.136	7.210e-128	1.665e-123

Original cluster	Annotated cell type	Gene	avg_log2FC	pct.1	pct.2	Raw P value	Bonferroni-adjusted P value
7	Blood vascular endothelial cells	*MGLL*	1.1782	0.497	0.006	0	0
7	Blood vascular endothelial cells	*SH3YL1*	1.1670	0.587	0.044	0	0
7	Blood vascular endothelial cells	*CRIP2*	1.1652	0.672	0.070	4.343e-297	1.003e-292
7	Blood vascular endothelial cells	*DIAPH2*	1.1647	0.877	0.403	1.632e-83	3.769e-79
7	Blood vascular endothelial cells	*TAGLN2*	1.1638	0.792	0.400	2.811e-70	6.492e-66
7	Blood vascular endothelial cells	*TSC22D1*	1.1625	0.895	0.600	3.975e-57	9.179e-53
7	Blood vascular endothelial cells	*HS3ST1*	1.1616	0.488	0.025	0	0
7	Blood vascular endothelial cells	*KCTD12*	1.1583	0.759	0.274	1.075e-90	2.483e-86
7	Blood vascular endothelial cells	*SPAAR*	1.1568	0.536	0.010	0	0
7	Blood vascular endothelial cells	*DNAJC13*	1.1556	0.620	0.209	5.652e-75	1.305e-70
7	Blood vascular endothelial cells	*FRYL*	1.1551	0.732	0.223	7.832e-103	1.808e-98
7	Blood vascular endothelial cells	*PLS3*	1.1474	0.660	0.129	3.447e-154	7.959e-150
7	Blood vascular endothelial cells	*KDM6B*	1.1437	0.693	0.268	7.070e-76	1.633e-71
7	Blood vascular endothelial cells	*PXDN*	1.1419	0.593	0.049	0	0
7	Blood vascular endothelial cells	*DLL4*	1.1387	0.476	0.004	0	0
7	Blood vascular endothelial cells	*BSG*	1.1386	0.955	0.702	1.963e-88	4.534e-84
7	Blood vascular endothelial cells	*NRP1*	1.1337	0.690	0.120	2.923e-180	6.750e-176
7	Blood vascular endothelial cells	*TM4SF18*	1.1330	0.503	0.002	0	0
7	Blood vascular endothelial cells	*COL4A2*	1.1326	0.560	0.086	2.968e-170	6.855e-166
7	Blood vascular endothelial cells	*FAM120B*	1.1304	0.617	0.052	0	0
7	Blood vascular endothelial cells	*CLEC14A*	1.1303	0.545	0.026	0	0
7	Blood vascular endothelial cells	*C4H21orf91*	1.1294	0.648	0.219	2.793e-76	6.449e-72
7	Blood vascular endothelial cells	*ANGPT2*	1.1204	0.428	0.086	2.955e-96	6.824e-92
7	Blood vascular endothelial cells	*CRYAB*	1.1141	0.593	0.061	1.312e-262	3.031e-258
7	Blood vascular endothelial cells	*ZFP36*	1.1127	0.892	0.738	1.332e-36	3.076e-32
7	Blood vascular endothelial cells	*FGD5*	1.1114	0.569	0.040	0	0
7	Blood vascular endothelial cells	*TIE1*	1.1078	0.572	0.033	0	0
7	Blood vascular endothelial cells	*BTNL9*	1.1060	0.331	0.001	0	0
7	Blood vascular endothelial cells	*PICALM*	1.1031	0.756	0.272	2.361e-84	5.452e-80
7	Blood vascular endothelial cells	*CTNNA1*	1.1008	0.831	0.332	5.697e-93	1.316e-88
7	Blood vascular endothelial cells	*MAPK6*	1.0991	0.584	0.110	1.423e-140	3.287e-136
7	Blood vascular endothelial cells	*IFNAR1*	1.0966	0.605	0.108	3.083e-158	7.119e-154
7	Blood vascular endothelial cells	*PTPN14*	1.0856	0.596	0.092	1.085e-177	2.505e-173
7	Blood vascular endothelial cells	*ARHGAP29*	1.0842	0.861	0.456	1.361e-75	3.142e-71
7	Blood vascular endothelial cells	*COL18A1*	1.0825	0.581	0.055	3.417e-279	7.891e-275
7	Blood vascular endothelial cells	*SYNE2*	1.0822	0.732	0.198	2.801e-117	6.468e-113
7	Blood vascular endothelial cells	*RNF138*	1.0794	0.744	0.227	1.688e-100	3.898e-96
7	Blood vascular endothelial cells	*PPP2R5A*	1.0749	0.717	0.211	3.724e-105	8.600e-101
7	Blood vascular endothelial cells	*AVPI1*	1.0740	0.518	0.114	2.345e-98	5.416e-94
7	Blood vascular endothelial cells	*RALB*	1.0715	0.666	0.105	3.509e-196	8.104e-192

Original cluster	Annotated cell type	Gene	avg_log2FC	pct.1	pct.2	Raw P value	Bonferroni-adjusted P value
7	Blood vascular endothelial cells	*TMSB4X*	1.0713	0.994	0.974	3.547e-114	8.191e-110
7	Blood vascular endothelial cells	*PPP3CA*	1.0688	0.834	0.420	8.652e-59	1.998e-54
7	Blood vascular endothelial cells	*SGK1*	1.0646	0.645	0.145	2.137e-125	4.935e-121
7	Blood vascular endothelial cells	*FLNB*	1.0643	0.669	0.116	4.512e-178	1.042e-173
7	Blood vascular endothelial cells	*CALM1*	1.0615	0.961	0.739	3.332e-79	7.695e-75
7	Blood vascular endothelial cells	*HDAC7*	1.0612	0.678	0.125	2.866e-167	6.617e-163
7	Blood vascular endothelial cells	*PAM*	1.0602	0.961	0.735	1.241e-67	2.865e-63
7	Blood vascular endothelial cells	*MET*	1.0601	0.506	0.018	0	0
7	Blood vascular endothelial cells	*RLIM*	1.0600	0.608	0.211	3.810e-67	8.798e-63
7	Blood vascular endothelial cells	*ITPRID2*	1.0552	0.575	0.138	4.705e-97	1.086e-92
7	Blood vascular endothelial cells	*ANKS1A*	1.0513	0.675	0.149	5.361e-133	1.238e-128
7	Blood vascular endothelial cells	*CPNE8*	1.0500	0.690	0.144	3.122e-143	7.210e-139
7	Blood vascular endothelial cells	*IQGAP1*	1.0488	0.723	0.200	3.995e-111	9.226e-107
7	Blood vascular endothelial cells	*CNTN5*	1.0469	0.160	0.017	2.329e-67	5.378e-63
7	Blood vascular endothelial cells	*TNFAIP8L1*	1.0456	0.497	0.006	0	0
7	Blood vascular endothelial cells	*TGFBR2*	1.0427	0.756	0.219	4.456e-112	1.029e-107
7	Blood vascular endothelial cells	*TRIM47*	1.0409	0.536	0.051	3.996e-254	9.226e-250
7	Blood vascular endothelial cells	*LOC138849320*	1.0374	0.383	0.018	0	0
7	Blood vascular endothelial cells	*ETV6*	1.0361	0.672	0.169	2.056e-114	4.747e-110
7	Blood vascular endothelial cells	*MYZAP*	1.0358	0.533	0.053	9.966e-242	2.301e-237
7	Blood vascular endothelial cells	*SPTLC2*	1.0340	0.530	0.076	2.644e-169	6.106e-165
7	Blood vascular endothelial cells	*CLIC4*	1.0309	0.765	0.348	6.535e-60	1.509e-55
7	Blood vascular endothelial cells	*LOC100347536*	1.0304	0.545	0.025	0	0
7	Blood vascular endothelial cells	*KALRN*	1.0298	0.527	0.138	4.207e-83	9.715e-79
7	Blood vascular endothelial cells	*CADPS2*	1.0276	0.584	0.110	3.222e-137	7.441e-133
7	Blood vascular endothelial cells	*MYRIP*	1.0233	0.443	0.026	0	0
7	Blood vascular endothelial cells	*PCDH7*	1.0223	0.804	0.371	4.739e-65	1.094e-60
7	Blood vascular endothelial cells	*GASK1B*	1.0223	0.503	0.017	0	0
7	Blood vascular endothelial cells	*MTUS1*	1.0206	0.578	0.133	4.478e-107	1.034e-102
7	Blood vascular endothelial cells	*MAN1A1*	1.0184	0.639	0.144	1.087e-121	2.510e-117
7	Blood vascular endothelial cells	*MINDY2*	1.0137	0.828	0.349	5.260e-75	1.215e-70
7	Blood vascular endothelial cells	*PDE2A*	1.0132	0.506	0.011	0	0
7	Blood vascular endothelial cells	*ARL4A*	1.0126	0.702	0.280	8.240e-60	1.903e-55
7	Blood vascular endothelial cells	*LOC138846666*	1.0087	0.476	0.002	0	0
7	Blood vascular endothelial cells	*LOC100349257*	1.0074	0.422	0.011	0	0
7	Blood vascular endothelial cells	*TBC1D9*	1.0071	0.575	0.087	9.628e-172	2.223e-167
7	Blood vascular endothelial cells	*DIPK2B*	1.0051	0.506	0.003	0	0
7	Blood vascular endothelial cells	*LAP3*	1.0035	0.729	0.236	5.856e-94	1.352e-89
7	Blood vascular endothelial cells	*SSH2*	0.9982	0.714	0.179	8.080e-122	1.866e-117
7	Blood vascular endothelial cells	*TINAGL1*	0.9978	0.593	0.058	4.438e-267	1.025e-262

Original cluster	Annotated cell type	Gene	avg_log2FC	pct.1	pct.2	Raw P value	Bonferroni-adjusted P value
7	Blood vascular endothelial cells	*ZBTB38*	0.9950	0.584	0.151	4.698e-91	1.085e-86
7	Blood vascular endothelial cells	*N4BP3*	0.9907	0.515	0.007	0	0
7	Blood vascular endothelial cells	*BAZ1A*	0.9876	0.542	0.046	4.337e-280	1.001e-275
7	Blood vascular endothelial cells	*SHROOM3*	0.9827	0.434	0.061	4.750e-142	1.097e-137
7	Blood vascular endothelial cells	*CEMIP2*	0.9820	0.455	0.133	1.583e-56	3.655e-52
7	Blood vascular endothelial cells	*CD300LG*	0.9817	0.413	0.000	0	0
7	Blood vascular endothelial cells	*IL33*	0.9796	0.479	0.048	7.540e-211	1.741e-206
7	Blood vascular endothelial cells	*CHRM3*	0.9689	0.169	0.013	9.012e-94	2.081e-89
7	Blood vascular endothelial cells	*TOP1*	0.9634	0.898	0.558	3.091e-53	7.138e-49
7	Blood vascular endothelial cells	*APLP2*	0.9614	0.934	0.670	1.580e-62	3.649e-58
7	Blood vascular endothelial cells	*SPTY2D1*	0.9606	0.648	0.187	9.316e-93	2.151e-88
7	Blood vascular endothelial cells	*MYO1B*	0.9605	0.717	0.195	3.321e-109	7.668e-105
7	Blood vascular endothelial cells	*CTBP2*	0.9599	0.627	0.184	9.641e-89	2.226e-84
7	Blood vascular endothelial cells	*DENND5A*	0.9595	0.756	0.240	2.861e-93	6.608e-89
7	Blood vascular endothelial cells	*STARD13*	0.9592	0.684	0.205	7.242e-88	1.672e-83
7	Blood vascular endothelial cells	*CNOT6L*	0.9573	0.771	0.367	3.597e-55	8.307e-51
7	Blood vascular endothelial cells	*GMDS*	0.9569	0.639	0.136	7.816e-128	1.805e-123
7	Blood vascular endothelial cells	*NDRG1*	0.9567	0.657	0.188	3.485e-92	8.048e-88
7	Blood vascular endothelial cells	*TLR4*	0.9559	0.530	0.015	0	0
7	Blood vascular endothelial cells	*LOC100340719*	0.9542	0.819	0.423	1.731e-54	3.996e-50
7	Blood vascular endothelial cells	*CCNYL1*	0.9537	0.482	0.077	1.484e-136	3.428e-132
7	Blood vascular endothelial cells	*GAB1*	0.9530	0.620	0.244	1.120e-50	2.586e-46
7	Blood vascular endothelial cells	*GPX1*	0.9506	0.861	0.500	9.928e-55	2.292e-50
7	Blood vascular endothelial cells	*PCMTD1*	0.9490	0.807	0.397	4.360e-56	1.007e-51
7	Blood vascular endothelial cells	*REL*	0.9479	0.473	0.054	6.128e-187	1.415e-182
7	Blood vascular endothelial cells	*THSD1*	0.9415	0.479	0.008	0	0
7	Blood vascular endothelial cells	*ADGRG6*	0.9393	0.238	0.024	1.254e-106	2.896e-102
7	Blood vascular endothelial cells	*CPD*	0.9376	0.759	0.269	7.357e-84	1.699e-79
7	Blood vascular endothelial cells	*SHE*	0.9367	0.506	0.004	0	0
7	Blood vascular endothelial cells	*CRACD*	0.9364	0.413	0.009	0	0
7	Blood vascular endothelial cells	*LOC100347786*	0.9361	0.443	0.004	0	0
7	Blood vascular endothelial cells	*TSPAN18*	0.9356	0.494	0.018	0	0
7	Blood vascular endothelial cells	*SLC38A2*	0.9346	0.654	0.186	1.164e-92	2.689e-88
7	Blood vascular endothelial cells	*GEM*	0.9340	0.425	0.103	1.335e-69	3.083e-65
7	Blood vascular endothelial cells	*DENND4A*	0.9279	0.672	0.242	3.444e-68	7.952e-64
7	Blood vascular endothelial cells	*JAG2*	0.9264	0.398	0.003	0	0
7	Blood vascular endothelial cells	*AKAP12*	0.9256	0.536	0.345	7.668e-15	1.771e-10
7	Blood vascular endothelial cells	*MAML2*	0.9245	0.843	0.461	6.692e-48	1.545e-43
7	Blood vascular endothelial cells	*SLC25A5*	0.9236	0.789	0.381	1.947e-65	4.496e-61
7	Blood vascular endothelial cells	*KMT2E*	0.9210	0.913	0.645	6.404e-51	1.479e-46

Original cluster	Annotated cell type	Gene	avg_log2FC	pct.1	pct.2	Raw P value	Bonferroni-adjusted P value
7	Blood vascular endothelial cells	*PTGS2*	0.9208	0.277	0.151	7.494e-10	1.730e-05
7	Blood vascular endothelial cells	*NFAT5*	0.9204	0.840	0.472	8.964e-46	2.070e-41
7	Blood vascular endothelial cells	*SLC7A5*	0.9183	0.416	0.088	1.159e-84	2.675e-80
7	Blood vascular endothelial cells	*PELI1*	0.9160	0.657	0.237	7.705e-69	1.779e-64
7	Blood vascular endothelial cells	*ELF1*	0.9137	0.705	0.220	1.390e-84	3.209e-80
7	Blood vascular endothelial cells	*PIM3*	0.9135	0.810	0.427	8.617e-55	1.990e-50
7	Blood vascular endothelial cells	*LOC100358539*	0.9121	0.274	0.008	3.159e-284	7.294e-280
7	Blood vascular endothelial cells	*TMBIM6*	0.9112	0.910	0.613	2.607e-65	6.021e-61
7	Blood vascular endothelial cells	*MBNL1*	0.9105	0.864	0.659	4.790e-22	1.106e-17
7	Blood vascular endothelial cells	*BCL3*	0.9088	0.434	0.027	1.407e-283	3.248e-279
7	Blood vascular endothelial cells	*ARHGEF3*	0.9057	0.503	0.058	5.568e-196	1.286e-191
7	Blood vascular endothelial cells	*LRCH1*	0.9035	0.633	0.176	2.715e-88	6.269e-84
7	Blood vascular endothelial cells	*TSC22D2*	0.9020	0.681	0.278	4.745e-53	1.096e-48
7	Blood vascular endothelial cells	*GGTA1*	0.9018	0.630	0.170	1.010e-91	2.332e-87
7	Blood vascular endothelial cells	*DLL1*	0.8966	0.443	0.016	0	0
7	Blood vascular endothelial cells	*MTSS1*	0.8958	0.693	0.243	3.346e-71	7.727e-67
7	Blood vascular endothelial cells	*ARAP3*	0.8934	0.518	0.015	0	0
7	Blood vascular endothelial cells	*HIVEP2*	0.8928	0.804	0.444	1.062e-44	2.453e-40
7	Blood vascular endothelial cells	*TMSB15A*	0.8927	0.864	0.477	1.089e-58	2.515e-54
7	Blood vascular endothelial cells	*EFNB2*	0.8923	0.572	0.221	2.044e-49	4.719e-45
7	Blood vascular endothelial cells	*SRPK2*	0.8910	0.771	0.409	1.668e-45	3.851e-41
7	Blood vascular endothelial cells	*DEPTOR*	0.8908	0.539	0.088	4.347e-145	1.004e-140
7	Blood vascular endothelial cells	*CEBPG*	0.8904	0.557	0.121	2.213e-110	5.109e-106
7	Blood vascular endothelial cells	*NOTCH1*	0.8859	0.521	0.043	3.919e-277	9.049e-273
7	Blood vascular endothelial cells	*SNAI1*	0.8853	0.458	0.026	0	0
7	Blood vascular endothelial cells	*NXN*	0.8845	0.587	0.129	1.774e-113	4.097e-109
7	Blood vascular endothelial cells	*CCN1*	0.8844	0.515	0.182	9.037e-45	2.087e-40
7	Blood vascular endothelial cells	*TES*	0.8830	0.518	0.076	2.923e-159	6.751e-155
7	Blood vascular endothelial cells	*WDFY1*	0.8818	0.572	0.086	6.204e-170	1.433e-165
7	Blood vascular endothelial cells	*EHD4*	0.8802	0.491	0.052	3.586e-209	8.280e-205
7	Blood vascular endothelial cells	*PSME4*	0.8754	0.554	0.178	1.840e-64	4.249e-60
7	Blood vascular endothelial cells	*KLHL4*	0.8742	0.328	0.008	0	0
7	Blood vascular endothelial cells	*S100A10*	0.8732	0.780	0.336	7.821e-62	1.806e-57
7	Blood vascular endothelial cells	*TIPARP*	0.8729	0.669	0.327	6.904e-42	1.594e-37
7	Blood vascular endothelial cells	*QKI*	0.8715	0.804	0.405	1.761e-57	4.067e-53
7	Blood vascular endothelial cells	*ZSWIM6*	0.8693	0.750	0.362	2.574e-45	5.945e-41
7	Blood vascular endothelial cells	*SLC52A2*	0.8675	0.578	0.145	3.364e-96	7.769e-92
7	Blood vascular endothelial cells	*CASP10*	0.8654	0.416	0.019	0	0
7	Blood vascular endothelial cells	*PCNA*	0.8650	0.551	0.207	1.039e-46	2.400e-42
7	Blood vascular endothelial cells	*LOC100357058*	0.8627	0.633	0.136	2.058e-124	4.752e-120

Original cluster	Annotated cell type	Gene	avg_log2FC	pct.1	pct.2	Raw P value	Bonferroni-adjusted P value
7	Blood vascular endothelial cells	*HSPG2*	0.8615	0.572	0.110	3.732e-127	8.617e-123
7	Blood vascular endothelial cells	*RAB11A*	0.8605	0.596	0.111	1.431e-139	3.304e-135
7	Blood vascular endothelial cells	*PHLDA1*	0.8602	0.467	0.068	3.846e-143	8.881e-139
7	Blood vascular endothelial cells	*SHROOM4*	0.8593	0.464	0.070	2.465e-134	5.693e-130
7	Blood vascular endothelial cells	*CSRNP1*	0.8587	0.663	0.203	6.653e-81	1.536e-76
7	Blood vascular endothelial cells	*SNCAIP*	0.8568	0.548	0.106	1.776e-117	4.100e-113
7	Blood vascular endothelial cells	*SGMS1*	0.8555	0.732	0.298	1.244e-57	2.873e-53
7	Blood vascular endothelial cells	*DACH2*	0.8542	0.349	0.053	9.053e-101	2.090e-96
7	Blood vascular endothelial cells	*CDC42EP2*	0.8535	0.461	0.036	1.144e-251	2.642e-247
7	Blood vascular endothelial cells	*MYC*	0.8530	0.654	0.263	1.424e-50	3.289e-46
7	Blood vascular endothelial cells	*KIAA1217*	0.8514	0.636	0.168	2.153e-95	4.971e-91
7	Blood vascular endothelial cells	*FYN*	0.8503	0.681	0.245	5.200e-66	1.201e-61
7	Blood vascular endothelial cells	*NFKBIA*	0.8483	0.910	0.761	9.522e-29	2.199e-24
7	Blood vascular endothelial cells	*CCDC85A*	0.8480	0.464	0.057	9.468e-167	2.186e-162
7	Blood vascular endothelial cells	*ARHGEF12*	0.8430	0.705	0.290	4.786e-55	1.105e-50
7	Blood vascular endothelial cells	*JAM2*	0.8428	0.584	0.217	6.928e-52	1.600e-47
7	Blood vascular endothelial cells	*IL4R*	0.8425	0.470	0.051	4.931e-194	1.139e-189
7	Blood vascular endothelial cells	*USHBP1*	0.8418	0.446	0.003	0	0
7	Blood vascular endothelial cells	*CASKIN2*	0.8414	0.479	0.037	7.743e-268	1.788e-263
7	Blood vascular endothelial cells	*ODC1*	0.8370	0.530	0.356	8.262e-12	1.908e-07
7	Blood vascular endothelial cells	*ARHGAP26*	0.8334	0.440	0.058	1.881e-149	4.344e-145
7	Blood vascular endothelial cells	*VAMP5*	0.8327	0.464	0.048	1.360e-203	3.141e-199
7	Blood vascular endothelial cells	*SVIL*	0.8325	0.756	0.367	4.379e-47	1.011e-42
7	Blood vascular endothelial cells	*YWHAB*	0.8320	0.792	0.396	3.603e-54	8.320e-50
7	Blood vascular endothelial cells	*ARRDC3*	0.8314	0.738	0.419	6.878e-37	1.588e-32
7	Blood vascular endothelial cells	*RAPGEF5*	0.8290	0.440	0.029	1.377e-275	3.179e-271
7	Blood vascular endothelial cells	*SLC66A3*	0.8289	0.530	0.092	4.884e-133	1.128e-128
7	Blood vascular endothelial cells	*FOSL2*	0.8285	0.744	0.402	9.471e-40	2.187e-35
7	Blood vascular endothelial cells	*TPST2*	0.8275	0.566	0.092	3.203e-153	7.396e-149
7	Blood vascular endothelial cells	*CXCL12*	0.8269	0.253	0.062	1.049e-39	2.422e-35
7	Blood vascular endothelial cells	*TSPO*	0.8231	0.648	0.198	2.252e-79	5.199e-75
7	Blood vascular endothelial cells	*TMEM40*	0.8230	0.407	0.004	0	0
7	Blood vascular endothelial cells	*FAM117B*	0.8162	0.401	0.082	1.668e-80	3.852e-76
7	Blood vascular endothelial cells	*SIPA1L1*	0.8123	0.699	0.239	3.042e-73	7.024e-69
7	Blood vascular endothelial cells	*TLL1*	0.8113	0.292	0.061	5.605e-59	1.294e-54
7	Blood vascular endothelial cells	*SFRP1*	0.8105	0.352	0.059	4.423e-90	1.021e-85
7	Blood vascular endothelial cells	*BTG1*	0.8091	0.922	0.655	3.296e-44	7.610e-40
7	Blood vascular endothelial cells	*ETNK1*	0.8080	0.768	0.380	3.505e-46	8.094e-42
7	Blood vascular endothelial cells	*SPRY4*	0.8079	0.380	0.015	0	0
7	Blood vascular endothelial cells	*SORBS1*	0.8064	0.461	0.060	7.464e-159	1.724e-154

Original cluster	Annotated cell type	Gene	avg_log2FC	pct.1	pct.2	Raw P value	Bonferroni-adjusted P value
7	Blood vascular endothelial cells	*LUZP1*	0.8046	0.455	0.058	1.287e-159	2.972e-155
7	Blood vascular endothelial cells	*NAV1*	0.8042	0.494	0.107	1.305e-92	3.015e-88
7	Blood vascular endothelial cells	*IFI44L*	0.8008	0.605	0.196	7.533e-67	1.739e-62
7	Blood vascular endothelial cells	*FXYD5*	0.8003	0.476	0.032	1.704e-292	3.935e-288
7	Blood vascular endothelial cells	*FAM124B*	0.7999	0.340	0.006	0	0
7	Blood vascular endothelial cells	*EHD2*	0.7991	0.575	0.123	1.086e-109	2.508e-105
7	Blood vascular endothelial cells	*DNAJB4*	0.7980	0.431	0.154	2.506e-38	5.788e-34
7	Blood vascular endothelial cells	*UBE2Q2*	0.7962	0.566	0.193	4.077e-55	9.414e-51
7	Blood vascular endothelial cells	*ABLIM3*	0.7957	0.446	0.022	0	0
7	Blood vascular endothelial cells	*UBN2*	0.7933	0.515	0.209	8.623e-38	1.991e-33
7	Blood vascular endothelial cells	*PTPN12*	0.7932	0.663	0.214	9.698e-73	2.239e-68
7	Blood vascular endothelial cells	*AHR*	0.7905	0.422	0.021	0	0
7	Blood vascular endothelial cells	*UCK2*	0.7872	0.383	0.038	3.234e-174	7.468e-170
7	Blood vascular endothelial cells	*PHACTR2*	0.7849	0.720	0.255	1.188e-70	2.744e-66
7	Blood vascular endothelial cells	*C7H1orf226*	0.7829	0.410	0.047	7.895e-159	1.823e-154
7	Blood vascular endothelial cells	*ARHGAP18*	0.7804	0.431	0.082	1.203e-95	2.778e-91
7	Blood vascular endothelial cells	*TJP2*	0.7799	0.413	0.033	1.706e-221	3.940e-217
7	Blood vascular endothelial cells	*RUNDC3B*	0.7797	0.352	0.030	8.663e-179	2.000e-174
7	Blood vascular endothelial cells	*PHACTR1*	0.7793	0.370	0.085	1.735e-64	4.007e-60
7	Blood vascular endothelial cells	*RNF125*	0.7792	0.587	0.140	1.211e-95	2.796e-91
7	Blood vascular endothelial cells	*TCIM*	0.7753	0.304	0.043	1.477e-94	3.412e-90
7	Blood vascular endothelial cells	*NAV3*	0.7715	0.398	0.068	1.361e-100	3.143e-96
7	Blood vascular endothelial cells	*PNP*	0.7689	0.542	0.137	9.433e-82	2.178e-77
7	Blood vascular endothelial cells	*SMURF2*	0.7680	0.623	0.231	1.144e-55	2.642e-51
7	Blood vascular endothelial cells	*EVA1C*	0.7680	0.416	0.034	4.123e-222	9.520e-218
7	Blood vascular endothelial cells	*MYO10*	0.7666	0.449	0.048	1.772e-188	4.092e-184
7	Blood vascular endothelial cells	*PDCD6*	0.7663	0.717	0.255	4.696e-68	1.084e-63
7	Blood vascular endothelial cells	*EXOC6*	0.7653	0.509	0.091	5.372e-122	1.240e-117
7	Blood vascular endothelial cells	*MS4A15*	0.7641	0.169	0.000	0	0
7	Blood vascular endothelial cells	*LXN*	0.7629	0.467	0.095	9.747e-95	2.251e-90
7	Blood vascular endothelial cells	*PDE8A*	0.7624	0.425	0.029	5.934e-261	1.370e-256
7	Blood vascular endothelial cells	*RNF19A*	0.7611	0.696	0.322	1.649e-43	3.807e-39
7	Blood vascular endothelial cells	*FGD4*	0.7611	0.488	0.074	1.780e-141	4.111e-137
7	Blood vascular endothelial cells	*TANC1*	0.7597	0.575	0.159	1.682e-77	3.884e-73
7	Blood vascular endothelial cells	*IL10RB*	0.7577	0.416	0.025	4.358e-279	1.006e-274
7	Blood vascular endothelial cells	*ERRFI1*	0.7563	0.648	0.320	5.762e-36	1.331e-31
7	Blood vascular endothelial cells	*BAG3*	0.7556	0.524	0.120	8.861e-94	2.046e-89
7	Blood vascular endothelial cells	*CHD1*	0.7554	0.645	0.271	2.146e-46	4.956e-42
7	Blood vascular endothelial cells	*HHEX*	0.7526	0.343	0.011	0	0
7	Blood vascular endothelial cells	*MARCHF3*	0.7506	0.440	0.063	1.703e-136	3.933e-132

Original cluster	Annotated cell type	Gene	avg_log2FC	pct.1	pct.2	Raw P value	Bonferroni-adjusted P value
7	Blood vascular endothelial cells	*SCARA3*	0.7501	0.449	0.043	8.569e-208	1.979e-203
7	Blood vascular endothelial cells	*DDX3X*	0.7484	0.883	0.586	2.475e-43	5.716e-39
7	Blood vascular endothelial cells	*TLE1*	0.7479	0.623	0.218	1.616e-60	3.732e-56
7	Blood vascular endothelial cells	*CD109*	0.7472	0.431	0.047	3.143e-175	7.258e-171
7	Blood vascular endothelial cells	*BIRC6*	0.7469	0.747	0.385	1.368e-38	3.159e-34
7	Blood vascular endothelial cells	*TBC1D8*	0.7458	0.401	0.059	9.304e-121	2.148e-116
7	Blood vascular endothelial cells	*CREB3L2*	0.7445	0.578	0.160	2.195e-77	5.070e-73
7	Blood vascular endothelial cells	*GMFG*	0.7425	0.473	0.030	3.480e-301	8.036e-297
7	Blood vascular endothelial cells	*USP6NL*	0.7412	0.461	0.058	8.079e-164	1.866e-159
7	Blood vascular endothelial cells	*STK38L*	0.7410	0.464	0.066	1.383e-144	3.193e-140
7	Blood vascular endothelial cells	*DEPP1*	0.7408	0.352	0.060	1.350e-89	3.117e-85
7	Blood vascular endothelial cells	*LOC100338015*	0.7394	0.428	0.119	3.463e-57	7.996e-53
7	Blood vascular endothelial cells	*SEC14L1*	0.7375	0.494	0.075	1.764e-140	4.073e-136
7	Blood vascular endothelial cells	*LOC108176804*	0.7373	0.383	0.004	0	0
7	Blood vascular endothelial cells	*ARHGEF15*	0.7370	0.377	0.003	0	0
7	Blood vascular endothelial cells	*SWAP70*	0.7366	0.518	0.124	5.824e-86	1.345e-81
7	Blood vascular endothelial cells	*RASSF3*	0.7360	0.506	0.093	1.699e-117	3.922e-113
7	Blood vascular endothelial cells	*SAMD9L*	0.7353	0.479	0.085	8.743e-115	2.019e-110
7	Blood vascular endothelial cells	*ELOVL7*	0.7333	0.377	0.014	0	0
7	Blood vascular endothelial cells	*CAP1*	0.7332	0.602	0.200	1.323e-65	3.055e-61
7	Blood vascular endothelial cells	*PEAK1*	0.7320	0.627	0.182	1.493e-76	3.449e-72
7	Blood vascular endothelial cells	*DISC1*	0.7294	0.358	0.020	6.137e-254	1.417e-249
7	Blood vascular endothelial cells	*MYO1C*	0.7294	0.512	0.095	4.592e-117	1.060e-112
7	Blood vascular endothelial cells	*MMRN2*	0.7283	0.346	0.003	0	0
7	Blood vascular endothelial cells	*SH3BGRL2*	0.7275	0.283	0.004	0	0
7	Blood vascular endothelial cells	*BCL6B*	0.7269	0.364	0.002	0	0
7	Blood vascular endothelial cells	*UTRN*	0.7266	0.880	0.539	9.845e-43	2.273e-38
7	Blood vascular endothelial cells	*HMCN1*	0.7264	0.428	0.095	2.894e-77	6.682e-73
7	Blood vascular endothelial cells	*HIP1*	0.7263	0.476	0.101	6.132e-94	1.416e-89
7	Blood vascular endothelial cells	*CD82*	0.7246	0.530	0.126	2.060e-87	4.757e-83
7	Blood vascular endothelial cells	*IGF2BP2*	0.7197	0.467	0.101	1.703e-87	3.933e-83
7	Blood vascular endothelial cells	*HOXD4*	0.7197	0.623	0.187	6.901e-73	1.594e-68
7	Blood vascular endothelial cells	*MBNL2*	0.7189	0.807	0.487	1.389e-35	3.207e-31
7	Blood vascular endothelial cells	*INKA1*	0.7188	0.425	0.034	9.274e-228	2.142e-223
7	Blood vascular endothelial cells	*CFL1*	0.7176	0.852	0.503	4.507e-47	1.041e-42
7	Blood vascular endothelial cells	*CTSS*	0.7171	0.443	0.026	9.973e-298	2.303e-293
7	Blood vascular endothelial cells	*LOC108175735*	0.7168	0.247	0.010	1.380e-218	3.187e-214
7	Blood vascular endothelial cells	*PLPP1*	0.7163	0.479	0.187	1.123e-35	2.593e-31
7	Blood vascular endothelial cells	*LOC103345766*	0.7135	0.301	0.012	4.948e-269	1.143e-264
7	Blood vascular endothelial cells	*RAPGEF3*	0.7125	0.407	0.010	0	0

Original cluster	Annotated cell type	Gene	avg_log2FC	pct.1	pct.2	Raw P value	Bonferroni-adjusted P value
7	Blood vascular endothelial cells	*SRGAP1*	0.7105	0.434	0.053	2.570e-157	5.935e-153
7	Blood vascular endothelial cells	*TACC1*	0.7100	0.678	0.215	2.424e-74	5.597e-70
7	Blood vascular endothelial cells	*ZCCHC2*	0.7082	0.509	0.090	1.866e-122	4.308e-118
7	Blood vascular endothelial cells	*ELN*	0.7077	0.395	0.053	1.055e-131	2.436e-127
7	Blood vascular endothelial cells	*CHMP3*	0.7073	0.762	0.306	5.294e-58	1.223e-53
7	Blood vascular endothelial cells	*ZBTB11*	0.7049	0.428	0.115	7.103e-58	1.640e-53
7	Blood vascular endothelial cells	*CHST15*	0.7047	0.392	0.012	0	0
7	Blood vascular endothelial cells	*CDS2*	0.7037	0.515	0.104	1.017e-106	2.349e-102
7	Blood vascular endothelial cells	*BMPR2*	0.7035	0.756	0.358	1.407e-42	3.248e-38
7	Blood vascular endothelial cells	*PPP3R1*	0.7033	0.873	0.580	1.235e-37	2.851e-33
7	Blood vascular endothelial cells	*GLS*	0.7031	0.813	0.479	1.875e-25	4.330e-21
7	Blood vascular endothelial cells	*C11H12orf75*	0.7025	0.301	0.009	0	0
7	Blood vascular endothelial cells	*LOC138843080*	0.7024	0.867	0.560	1.458e-35	3.366e-31
7	Blood vascular endothelial cells	*CD2AP*	0.6991	0.762	0.336	9.620e-47	2.221e-42
7	Blood vascular endothelial cells	*MYO1E*	0.6933	0.530	0.297	8.217e-16	1.898e-11
7	Blood vascular endothelial cells	*PLEKHO1*	0.6928	0.395	0.050	6.503e-140	1.502e-135
7	Blood vascular endothelial cells	*LOC127484663*	0.6918	0.289	0.007	0	0
7	Blood vascular endothelial cells	*ATP11C*	0.6918	0.614	0.245	2.135e-45	4.929e-41
7	Blood vascular endothelial cells	*RHOC*	0.6916	0.590	0.168	1.318e-73	3.044e-69
7	Blood vascular endothelial cells	*PPP2R2A*	0.6915	0.732	0.382	5.740e-37	1.325e-32
7	Blood vascular endothelial cells	*IL18*	0.6904	0.331	0.007	0	0
7	Blood vascular endothelial cells	*SOCS2*	0.6903	0.569	0.161	1.412e-69	3.259e-65
7	Blood vascular endothelial cells	*SNTB2*	0.6891	0.533	0.182	4.525e-52	1.045e-47
7	Blood vascular endothelial cells	*RAI14*	0.6885	0.410	0.050	1.540e-148	3.557e-144
7	Blood vascular endothelial cells	*HOOK3*	0.6863	0.795	0.426	1.519e-43	3.509e-39
7	Blood vascular endothelial cells	*MOB2*	0.6862	0.675	0.198	8.933e-80	2.063e-75
7	Blood vascular endothelial cells	*TBPL1*	0.6856	0.581	0.242	6.876e-35	1.588e-30
7	Blood vascular endothelial cells	*PREX2*	0.6852	0.804	0.442	1.552e-33	3.584e-29
7	Blood vascular endothelial cells	*SPATS2*	0.6845	0.479	0.105	5.109e-89	1.180e-84
7	Blood vascular endothelial cells	*APBB2*	0.6841	0.575	0.153	1.558e-80	3.598e-76
7	Blood vascular endothelial cells	*ARID5A*	0.6839	0.401	0.046	9.292e-157	2.146e-152
7	Blood vascular endothelial cells	*FKBP1A*	0.6812	0.605	0.204	3.509e-57	8.104e-53
7	Blood vascular endothelial cells	*TSPAN12*	0.6802	0.355	0.013	0	0
7	Blood vascular endothelial cells	*MFGE8*	0.6800	0.542	0.147	4.737e-72	1.094e-67
7	Blood vascular endothelial cells	*NR4A2*	0.6794	0.566	0.132	5.246e-93	1.211e-88
7	Blood vascular endothelial cells	*NT5E*	0.6771	0.298	0.102	3.136e-28	7.241e-24
7	Blood vascular endothelial cells	*NFATC2*	0.6769	0.545	0.138	8.774e-81	2.026e-76
7	Blood vascular endothelial cells	*APC*	0.6761	0.657	0.345	2.782e-32	6.424e-28
7	Blood vascular endothelial cells	*THBS1*	0.6760	0.352	0.143	1.566e-22	3.617e-18
7	Blood vascular endothelial cells	*CCNT1*	0.6753	0.503	0.115	5.514e-89	1.273e-84

Original cluster	Annotated cell type	Gene	avg_log2FC	pct.1	pct.2	Raw P value	Bonferroni-adjusted P value
7	Blood vascular endothelial cells	*RELL1*	0.6735	0.425	0.074	4.114e-106	9.501e-102
7	Blood vascular endothelial cells	*CSGALNACT1*	0.6729	0.361	0.034	1.432e-169	3.306e-165
7	Blood vascular endothelial cells	*TMEM255B*	0.6722	0.398	0.009	0	0
7	Blood vascular endothelial cells	*RIPK2*	0.6719	0.274	0.064	1.171e-46	2.705e-42
7	Blood vascular endothelial cells	*FAM184A*	0.6712	0.289	0.054	3.916e-65	9.043e-61
7	Blood vascular endothelial cells	*FNIP2*	0.6677	0.416	0.069	5.058e-109	1.168e-104
7	Blood vascular endothelial cells	*DUSP5*	0.6673	0.380	0.063	1.940e-99	4.481e-95
7	Blood vascular endothelial cells	*STXBP6*	0.6668	0.422	0.109	4.573e-61	1.056e-56
7	Blood vascular endothelial cells	*LY6S*	0.6667	0.364	0.012	0	0
7	Blood vascular endothelial cells	*ZNRF1*	0.6667	0.533	0.141	1.077e-74	2.486e-70
7	Blood vascular endothelial cells	*FOXC1*	0.6654	0.340	0.024	3.295e-205	7.609e-201
7	Blood vascular endothelial cells	*SEC23B*	0.6652	0.398	0.070	2.057e-96	4.749e-92
7	Blood vascular endothelial cells	*LOC100350928*	0.6651	0.413	0.077	3.575e-93	8.256e-89
7	Blood vascular endothelial cells	*CEP85L*	0.6650	0.554	0.211	9.610e-42	2.219e-37
7	Blood vascular endothelial cells	*TMEM123*	0.6648	0.633	0.207	7.081e-65	1.635e-60
7	Blood vascular endothelial cells	*ZNF326*	0.6646	0.798	0.496	1.455e-29	3.360e-25
7	Blood vascular endothelial cells	*DYSF*	0.6645	0.392	0.011	0	0
7	Blood vascular endothelial cells	*GPR182*	0.6609	0.334	0.006	0	0
7	Blood vascular endothelial cells	*SLC30A1*	0.6589	0.431	0.045	7.601e-184	1.755e-179
7	Blood vascular endothelial cells	*KLF10*	0.6583	0.587	0.237	1.728e-41	3.991e-37
7	Blood vascular endothelial cells	*TTC28*	0.6583	0.837	0.575	6.118e-23	1.413e-18
7	Blood vascular endothelial cells	*RGCC*	0.6578	0.355	0.043	3.031e-129	6.998e-125
7	Blood vascular endothelial cells	*GFOD2*	0.6556	0.401	0.052	7.552e-138	1.744e-133
7	Blood vascular endothelial cells	*ZNF277*	0.6554	0.864	0.549	3.980e-37	9.191e-33
7	Blood vascular endothelial cells	*STAT1*	0.6551	0.491	0.132	2.970e-67	6.858e-63
7	Blood vascular endothelial cells	*PITPNC1*	0.6549	0.521	0.129	1.041e-79	2.404e-75
7	Blood vascular endothelial cells	*MSANTD2*	0.6537	0.512	0.220	1.528e-30	3.529e-26
7	Blood vascular endothelial cells	*GRK5*	0.6508	0.452	0.080	2.243e-106	5.179e-102
7	Blood vascular endothelial cells	*SPCS3*	0.6507	0.681	0.257	2.536e-52	5.856e-48
7	Blood vascular endothelial cells	*EIF4E3*	0.6505	0.645	0.269	1.714e-42	3.958e-38
7	Blood vascular endothelial cells	*AFF4*	0.6505	0.660	0.317	1.625e-34	3.752e-30
7	Blood vascular endothelial cells	*FBXL7*	0.6452	0.768	0.471	4.710e-24	1.088e-19
7	Blood vascular endothelial cells	*HM13*	0.6445	0.557	0.149	9.248e-77	2.135e-72
7	Blood vascular endothelial cells	*ITGA1*	0.6442	0.542	0.156	3.372e-66	7.786e-62
7	Blood vascular endothelial cells	*STK17A*	0.6436	0.566	0.096	1.218e-141	2.812e-137
7	Blood vascular endothelial cells	*PAK2*	0.6428	0.768	0.362	2.135e-44	4.930e-40
7	Blood vascular endothelial cells	*PDLIM5*	0.6424	0.633	0.225	2.720e-52	6.282e-48
7	Blood vascular endothelial cells	*CMIP*	0.6419	0.458	0.092	3.257e-94	7.522e-90
7	Blood vascular endothelial cells	*GPD2*	0.6417	0.407	0.061	3.162e-117	7.302e-113
7	Blood vascular endothelial cells	*SPRING1*	0.6408	0.443	0.070	3.542e-120	8.179e-116

Original cluster	Annotated cell type	Gene	avg_log2FC	pct.1	pct.2	Raw P value	Bonferroni-adjusted P value
7	Blood vascular endothelial cells	*HYAL2*	0.6391	0.395	0.075	2.211e-85	5.105e-81
7	Blood vascular endothelial cells	*SH3BGRL3*	0.6385	0.663	0.197	8.353e-78	1.929e-73
7	Blood vascular endothelial cells	*CD151*	0.6378	0.533	0.143	1.504e-72	3.473e-68
7	Blood vascular endothelial cells	*PLEC*	0.6365	0.479	0.120	5.272e-73	1.217e-68
7	Blood vascular endothelial cells	*MAP3K11*	0.6352	0.395	0.026	1.180e-244	2.725e-240
7	Blood vascular endothelial cells	*SORBS2*	0.6352	0.491	0.076	2.569e-131	5.933e-127
7	Blood vascular endothelial cells	*LOC100328942*	0.6336	0.620	0.201	3.501e-61	8.085e-57
7	Blood vascular endothelial cells	*HLX*	0.6329	0.331	0.003	0	0
7	Blood vascular endothelial cells	*LOC108175429*	0.6328	0.226	0.010	3.715e-188	8.580e-184
7	Blood vascular endothelial cells	*SNAP23*	0.6325	0.524	0.108	6.105e-102	1.410e-97
7	Blood vascular endothelial cells	*XRN2*	0.6323	0.651	0.256	1.197e-47	2.765e-43
7	Blood vascular endothelial cells	*CPEB4*	0.6314	0.488	0.116	8.870e-80	2.048e-75
7	Blood vascular endothelial cells	*MCU*	0.6266	0.533	0.190	1.727e-47	3.987e-43
7	Blood vascular endothelial cells	*ASAP1*	0.6266	0.696	0.304	9.450e-45	2.182e-40
7	Blood vascular endothelial cells	*MTMR2*	0.6262	0.461	0.123	2.657e-62	6.135e-58
7	Blood vascular endothelial cells	*H2AZ1*	0.6258	0.892	0.710	5.923e-26	1.368e-21
7	Blood vascular endothelial cells	*LOC100346112*	0.6231	0.855	0.579	3.154e-33	7.284e-29
7	Blood vascular endothelial cells	*AFDN*	0.6230	0.364	0.027	7.204e-207	1.664e-202
7	Blood vascular endothelial cells	*TNS1*	0.6220	0.738	0.346	1.658e-44	3.829e-40
7	Blood vascular endothelial cells	*NRP2*	0.6215	0.461	0.082	2.072e-109	4.786e-105
7	Blood vascular endothelial cells	*KIF26A*	0.6210	0.340	0.003	0	0
7	Blood vascular endothelial cells	*DOCK6*	0.6195	0.413	0.034	6.897e-213	1.593e-208
7	Blood vascular endothelial cells	*ACVRL1*	0.6189	0.358	0.025	1.701e-216	3.928e-212
7	Blood vascular endothelial cells	*PDE10A*	0.6188	0.352	0.071	1.122e-71	2.590e-67
7	Blood vascular endothelial cells	*LTC4S*	0.6163	0.377	0.013	0	0
7	Blood vascular endothelial cells	*SPTAN1*	0.6156	0.569	0.212	3.028e-45	6.993e-41
7	Blood vascular endothelial cells	*PLA2G2D*	0.6149	0.343	0.001	0	0
7	Blood vascular endothelial cells	*LRMDA*	0.6149	0.702	0.424	6.920e-22	1.598e-17
7	Blood vascular endothelial cells	*JAG1*	0.6149	0.383	0.095	2.727e-57	6.297e-53
7	Blood vascular endothelial cells	*ACTN4*	0.6146	0.587	0.187	6.445e-61	1.488e-56
7	Blood vascular endothelial cells	*TSHZ2*	0.6146	0.750	0.441	3.562e-27	8.225e-23
7	Blood vascular endothelial cells	*MCTP1*	0.6137	0.331	0.029	1.299e-164	3.000e-160
7	Blood vascular endothelial cells	*PTBP3*	0.6135	0.633	0.202	2.959e-64	6.834e-60
7	Blood vascular endothelial cells	*CAVIN3*	0.6110	0.837	0.545	1.225e-27	2.829e-23
7	Blood vascular endothelial cells	*SH2D3C*	0.6104	0.446	0.079	4.617e-104	1.066e-99
7	Blood vascular endothelial cells	*SIPA1L2*	0.6103	0.503	0.124	6.839e-77	1.579e-72
7	Blood vascular endothelial cells	*SH3TC2*	0.6095	0.310	0.003	0	0
7	Blood vascular endothelial cells	*FOXP1*	0.6087	0.825	0.542	3.640e-24	8.406e-20
7	Blood vascular endothelial cells	*ANKRD42*	0.6082	0.455	0.149	6.029e-46	1.392e-41
7	Blood vascular endothelial cells	*LOC138845330*	0.6080	0.241	0.002	0	0

Original cluster	Annotated cell type	Gene	avg_log2FC	pct.1	pct.2	Raw P value	Bonferroni-adjusted P value
7	Blood vascular endothelial cells	*MAP3K3*	0.6067	0.551	0.209	8.510e-43	1.965e-38
7	Blood vascular endothelial cells	*SUSD6*	0.6063	0.428	0.078	9.062e-98	2.093e-93
7	Blood vascular endothelial cells	*NFKBIZ*	0.6053	0.831	0.434	1.630e-39	3.764e-35
7	Blood vascular endothelial cells	*UBAP2*	0.6034	0.575	0.208	1.240e-47	2.863e-43
7	Blood vascular endothelial cells	*MAP4*	0.6019	0.651	0.265	1.216e-43	2.808e-39
7	Blood vascular endothelial cells	*NRARP*	0.6003	0.370	0.040	9.450e-149	2.182e-144
7	Blood vascular endothelial cells	*EZH2*	0.5987	0.370	0.052	4.726e-114	1.091e-109
7	Blood vascular endothelial cells	*LOC100342069*	0.5977	0.238	0.003	0	0
7	Blood vascular endothelial cells	*CDKN1A*	0.5977	0.425	0.106	6.963e-65	1.608e-60
7	Blood vascular endothelial cells	*TSPAN2*	0.5976	0.419	0.048	7.982e-160	1.843e-155
7	Blood vascular endothelial cells	*ANKRD17*	0.5968	0.717	0.404	4.116e-26	9.505e-22
7	Blood vascular endothelial cells	*NOD1*	0.5951	0.340	0.021	3.614e-220	8.345e-216
7	Blood vascular endothelial cells	*KIF5B*	0.5940	0.651	0.295	2.856e-36	6.595e-32
7	Blood vascular endothelial cells	*ARHGEF9*	0.5931	0.370	0.072	4.736e-80	1.094e-75
7	Blood vascular endothelial cells	*DYRK1A*	0.5930	0.542	0.249	3.183e-28	7.350e-24
7	Blood vascular endothelial cells	*LOC100358417*	0.5922	0.343	0.073	7.534e-65	1.740e-60
7	Blood vascular endothelial cells	*HINT1*	0.5917	0.753	0.389	4.581e-33	1.058e-28
7	Blood vascular endothelial cells	*CYSLTR2*	0.5909	0.223	0.006	8.489e-244	1.960e-239
7	Blood vascular endothelial cells	*LRRFIP1*	0.5897	0.572	0.192	8.344e-54	1.927e-49
7	Blood vascular endothelial cells	*LOC100355470*	0.5885	0.663	0.252	1.130e-49	2.610e-45
7	Blood vascular endothelial cells	*UBE2D3*	0.5881	0.771	0.488	5.842e-27	1.349e-22
7	Blood vascular endothelial cells	*SIK3*	0.5879	0.623	0.287	2.824e-33	6.521e-29
7	Blood vascular endothelial cells	*FBXO11*	0.5875	0.747	0.469	4.548e-19	1.050e-14
7	Blood vascular endothelial cells	*SNX25*	0.5867	0.358	0.056	3.852e-100	8.895e-96
7	Blood vascular endothelial cells	*CCDC125*	0.5861	0.337	0.018	1.302e-247	3.007e-243
7	Blood vascular endothelial cells	*NEDD4*	0.5849	0.455	0.094	3.653e-87	8.436e-83
7	Blood vascular endothelial cells	*PTPRE*	0.5823	0.325	0.036	7.850e-130	1.813e-125
7	Blood vascular endothelial cells	*PXN*	0.5820	0.452	0.094	3.541e-86	8.177e-82
7	Blood vascular endothelial cells	*RHOA*	0.5819	0.870	0.604	4.625e-36	1.068e-31
7	Blood vascular endothelial cells	*IRF2BP2*	0.5817	0.717	0.358	1.008e-31	2.328e-27
7	Blood vascular endothelial cells	*MAP3K8*	0.5811	0.337	0.021	1.280e-223	2.957e-219
7	Blood vascular endothelial cells	*RNF19B*	0.5810	0.506	0.184	5.549e-40	1.281e-35
7	Blood vascular endothelial cells	*ZNF366*	0.5808	0.304	0.001	0	0
7	Blood vascular endothelial cells	*ABHD17C*	0.5805	0.358	0.039	8.239e-146	1.902e-141
7	Blood vascular endothelial cells	*LOC138846330*	0.5804	0.160	0.012	6.493e-93	1.499e-88
7	Blood vascular endothelial cells	*HEG1*	0.5803	0.361	0.061	4.581e-92	1.058e-87
7	Blood vascular endothelial cells	*PHLPP1*	0.5797	0.467	0.137	4.735e-54	1.093e-49
7	Blood vascular endothelial cells	*LOC100339318*	0.5796	0.401	0.067	3.188e-102	7.361e-98
7	Blood vascular endothelial cells	*DAB2*	0.5796	0.464	0.102	1.275e-82	2.944e-78
7	Blood vascular endothelial cells	*TOPORS*	0.5788	0.596	0.226	2.249e-43	5.194e-39

Original cluster	Annotated cell type	Gene	avg_log2FC	pct.1	pct.2	Raw P value	Bonferroni-adjusted P value
7	Blood vascular endothelial cells	*JARID2*	0.5779	0.482	0.172	1.402e-41	3.237e-37
7	Blood vascular endothelial cells	*SLC3A2*	0.5762	0.714	0.376	2.156e-29	4.978e-25
7	Blood vascular endothelial cells	*RC3H1*	0.5757	0.551	0.206	5.383e-42	1.243e-37
7	Blood vascular endothelial cells	*FERMT2*	0.5737	0.681	0.325	5.506e-33	1.271e-28
7	Blood vascular endothelial cells	*CDC42BPB*	0.5721	0.497	0.123	1.456e-74	3.363e-70
7	Blood vascular endothelial cells	*GNG12*	0.5713	0.440	0.098	4.529e-77	1.046e-72
7	Blood vascular endothelial cells	*CALCOCO2*	0.5695	0.645	0.275	1.557e-37	3.595e-33
7	Blood vascular endothelial cells	*LARP1B*	0.5688	0.470	0.120	7.630e-68	1.762e-63
7	Blood vascular endothelial cells	*TGIF1*	0.5669	0.630	0.300	3.813e-31	8.805e-27
7	Blood vascular endothelial cells	*MCL1*	0.5647	0.780	0.419	5.680e-33	1.312e-28
7	Blood vascular endothelial cells	*TPM3*	0.5645	0.587	0.175	5.611e-65	1.296e-60
7	Blood vascular endothelial cells	*LOC100351475*	0.5636	0.304	0.005	0	0
7	Blood vascular endothelial cells	*PARVA*	0.5631	0.584	0.194	1.927e-54	4.449e-50
7	Blood vascular endothelial cells	*TSPAN4*	0.5626	0.527	0.162	5.382e-55	1.243e-50
7	Blood vascular endothelial cells	*GTF2I*	0.5617	0.581	0.221	1.477e-42	3.410e-38
7	Blood vascular endothelial cells	*LOC138844642*	0.5600	0.292	0.004	0	0
7	Blood vascular endothelial cells	*FEZ2*	0.5598	0.461	0.118	6.515e-65	1.504e-60
7	Blood vascular endothelial cells	*GADD45G*	0.5586	0.645	0.413	2.346e-16	5.417e-12
7	Blood vascular endothelial cells	*CARD10*	0.5582	0.316	0.005	0	0
7	Blood vascular endothelial cells	*SPTSSA*	0.5580	0.467	0.123	5.318e-63	1.228e-58
7	Blood vascular endothelial cells	*CTNND1*	0.5578	0.527	0.154	2.502e-61	5.778e-57
7	Blood vascular endothelial cells	*TCN2*	0.5574	0.497	0.143	3.394e-59	7.838e-55
7	Blood vascular endothelial cells	*PPP1R13B*	0.5573	0.512	0.140	2.577e-65	5.950e-61
7	Blood vascular endothelial cells	*CD36*	0.5565	0.120	0.004	3.916e-111	9.042e-107
7	Blood vascular endothelial cells	*CHST7*	0.5559	0.404	0.080	6.456e-82	1.491e-77
7	Blood vascular endothelial cells	*TC2N*	0.5555	0.373	0.106	3.016e-46	6.964e-42
7	Blood vascular endothelial cells	*ELL2*	0.5549	0.470	0.144	2.491e-50	5.752e-46
7	Blood vascular endothelial cells	*MCC*	0.5548	0.461	0.173	2.991e-35	6.907e-31
7	Blood vascular endothelial cells	*KCMF1*	0.5546	0.566	0.210	1.145e-43	2.645e-39
7	Blood vascular endothelial cells	*ITGB1*	0.5529	0.780	0.446	1.075e-28	2.483e-24
7	Blood vascular endothelial cells	*PTP4A3*	0.5524	0.268	0.020	4.811e-153	1.111e-148
7	Blood vascular endothelial cells	*DNAJC10*	0.5520	0.494	0.196	6.252e-33	1.444e-28
7	Blood vascular endothelial cells	*GNAI2*	0.5516	0.834	0.531	5.766e-28	1.332e-23
7	Blood vascular endothelial cells	*PPP1R2*	0.5511	0.630	0.330	5.436e-24	1.255e-19
7	Blood vascular endothelial cells	*LOC138844436*	0.5503	0.328	0.006	0	0
7	Blood vascular endothelial cells	*AGPAT4*	0.5502	0.259	0.016	2.503e-166	5.779e-162
7	Blood vascular endothelial cells	*ARHGAP25*	0.5467	0.286	0.014	4.387e-218	1.013e-213
7	Blood vascular endothelial cells	*NOSTRIN*	0.5464	0.271	0.002	0	0
7	Blood vascular endothelial cells	*BAG1*	0.5461	0.467	0.108	1.377e-77	3.180e-73
7	Blood vascular endothelial cells	*NOS3*	0.5454	0.304	0.004	0	0

Original cluster	Annotated cell type	Gene	avg_log2FC	pct.1	pct.2	Raw P value	Bonferroni-adjusted P value
7	Blood vascular endothelial cells	*IFNAR2*	0.5448	0.428	0.088	1.965e-83	4.539e-79
7	Blood vascular endothelial cells	*YWHAZ*	0.5440	0.880	0.638	4.238e-29	9.787e-25
7	Blood vascular endothelial cells	*ELF2*	0.5436	0.696	0.390	2.226e-22	5.141e-18
7	Blood vascular endothelial cells	*SH3GLB1*	0.5430	0.666	0.308	7.100e-36	1.640e-31
7	Blood vascular endothelial cells	*DDAH1*	0.5418	0.196	0.014	5.918e-113	1.367e-108
7	Blood vascular endothelial cells	*LYL1*	0.5411	0.334	0.035	2.754e-141	6.361e-137
7	Blood vascular endothelial cells	*LOC103347814*	0.5407	0.262	0.007	3.124e-282	7.214e-278
7	Blood vascular endothelial cells	*CHN2*	0.5399	0.410	0.108	1.128e-55	2.606e-51
7	Blood vascular endothelial cells	*FOXF1*	0.5389	0.280	0.001	0	0
7	Blood vascular endothelial cells	*C9H12orf57*	0.5379	0.726	0.339	3.875e-34	8.949e-30
7	Blood vascular endothelial cells	*EIF3A*	0.5379	0.654	0.303	3.388e-34	7.824e-30
7	Blood vascular endothelial cells	*LOC127489568*	0.5373	0.687	0.312	1.385e-37	3.198e-33
7	Blood vascular endothelial cells	*LNX2*	0.5367	0.187	0.031	4.729e-48	1.092e-43
7	Blood vascular endothelial cells	*ARID4B*	0.5352	0.867	0.614	5.092e-24	1.176e-19
7	Blood vascular endothelial cells	*IFRD1*	0.5334	0.584	0.205	4.727e-48	1.092e-43
7	Blood vascular endothelial cells	*PURB*	0.5321	0.627	0.278	5.123e-35	1.183e-30
7	Blood vascular endothelial cells	*SELP*	0.5309	0.114	0.000	1.973e-207	4.556e-203
7	Blood vascular endothelial cells	*REEP3*	0.5306	0.605	0.262	2.617e-35	6.044e-31
7	Blood vascular endothelial cells	*DOCK1*	0.5299	0.455	0.135	2.273e-50	5.250e-46
7	Blood vascular endothelial cells	*PDGFB*	0.5281	0.319	0.017	4.587e-237	1.059e-232
7	Blood vascular endothelial cells	*MBNL3*	0.5277	0.289	0.005	0	0
7	Blood vascular endothelial cells	*KITLG*	0.5273	0.271	0.039	1.644e-83	3.796e-79
7	Blood vascular endothelial cells	*IFI44*	0.5268	0.364	0.061	5.802e-93	1.340e-88
7	Blood vascular endothelial cells	*APLNR*	0.5259	0.265	0.001	0	0
7	Blood vascular endothelial cells	*HSPH1*	0.5250	0.416	0.163	4.459e-28	1.030e-23
7	Blood vascular endothelial cells	*SKAP2*	0.5250	0.334	0.035	1.377e-140	3.180e-136
7	Blood vascular endothelial cells	*CYTH1*	0.5242	0.512	0.158	4.036e-52	9.319e-48
7	Blood vascular endothelial cells	*MAFB*	0.5241	0.575	0.246	1.118e-31	2.581e-27
7	Blood vascular endothelial cells	*TSPAN6*	0.5214	0.666	0.326	9.724e-29	2.245e-24
7	Blood vascular endothelial cells	*CYFIP1*	0.5212	0.527	0.147	2.811e-62	6.490e-58
7	Blood vascular endothelial cells	*FBXO33*	0.5204	0.533	0.203	3.661e-38	8.454e-34
7	Blood vascular endothelial cells	*MAPK3*	0.5203	0.470	0.118	2.529e-67	5.839e-63
7	Blood vascular endothelial cells	*DST*	0.5194	0.852	0.570	5.063e-26	1.169e-21
7	Blood vascular endothelial cells	*VPS37B*	0.5193	0.389	0.058	4.848e-112	1.119e-107
7	Blood vascular endothelial cells	*LRRC8D*	0.5185	0.340	0.059	4.530e-84	1.046e-79
7	Blood vascular endothelial cells	*LPGAT1*	0.5182	0.361	0.108	2.892e-41	6.678e-37
7	Blood vascular endothelial cells	*SNAPC1*	0.5182	0.458	0.174	1.002e-32	2.313e-28
7	Blood vascular endothelial cells	*SHISA5*	0.5181	0.515	0.164	8.404e-51	1.941e-46
7	Blood vascular endothelial cells	*GADD45B*	0.5176	0.639	0.246	1.040e-46	2.402e-42
7	Blood vascular endothelial cells	*RGL2*	0.5176	0.410	0.106	8.021e-58	1.852e-53

Original cluster	Annotated cell type	Gene	avg_log2FC	pct.1	pct.2	Raw P value	Bonferroni-adjusted P value
7	Blood vascular endothelial cells	*TAMALIN*	0.5174	0.482	0.129	8.835e-63	2.040e-58
7	Blood vascular endothelial cells	*SYNJ1*	0.5172	0.377	0.100	1.312e-51	3.029e-47
7	Blood vascular endothelial cells	*ACSL4*	0.5168	0.419	0.071	5.227e-103	1.207e-98
7	Blood vascular endothelial cells	*GALNT15*	0.5165	0.307	0.002	0	0
7	Blood vascular endothelial cells	*EFCC1*	0.5159	0.310	0.020	2.092e-200	4.830e-196
7	Blood vascular endothelial cells	*SMTN*	0.5147	0.467	0.100	1.536e-83	3.548e-79
7	Blood vascular endothelial cells	*LY96*	0.5145	0.319	0.035	1.569e-126	3.622e-122
7	Blood vascular endothelial cells	*DENND2C*	0.5141	0.358	0.047	6.559e-119	1.515e-114
7	Blood vascular endothelial cells	*LOC103348141*	0.5140	0.666	0.339	8.157e-26	1.884e-21
7	Blood vascular endothelial cells	*TTC39B*	0.5139	0.401	0.081	1.855e-79	4.282e-75
7	Blood vascular endothelial cells	*SLC39A1*	0.5135	0.596	0.264	1.295e-31	2.991e-27
7	Blood vascular endothelial cells	*MYO1D*	0.5132	0.307	0.060	6.203e-65	1.432e-60
7	Blood vascular endothelial cells	*LOC100349347*	0.5131	0.404	0.077	5.457e-86	1.260e-81
7	Blood vascular endothelial cells	*RNF152*	0.5120	0.286	0.023	4.035e-154	9.318e-150
7	Blood vascular endothelial cells	*RIPOR1*	0.5119	0.358	0.047	6.970e-119	1.610e-114
7	Blood vascular endothelial cells	*LOC138849409*	0.5110	0.346	0.076	4.234e-62	9.777e-58
7	Blood vascular endothelial cells	*GFOD1*	0.5096	0.259	0.023	9.768e-126	2.256e-121
7	Blood vascular endothelial cells	*CDC42SE2*	0.5095	0.434	0.080	9.368e-97	2.163e-92
7	Blood vascular endothelial cells	*CSNK1A1*	0.5094	0.756	0.409	2.522e-28	5.823e-24
7	Blood vascular endothelial cells	*AFAP1L1*	0.5088	0.259	0.004	0	0
7	Blood vascular endothelial cells	*F11R*	0.5076	0.313	0.013	3.186e-269	7.357e-265
7	Blood vascular endothelial cells	*PCDH12*	0.5076	0.265	0.004	0	0
7	Blood vascular endothelial cells	*TM6SF1*	0.5073	0.322	0.017	1.798e-241	4.152e-237
7	Blood vascular endothelial cells	*TRIO*	0.5071	0.611	0.256	1.091e-35	2.519e-31
7	Blood vascular endothelial cells	*FLT4*	0.5062	0.328	0.009	0	0
7	Blood vascular endothelial cells	*CBL*	0.5054	0.389	0.069	8.086e-92	1.867e-87
7	Blood vascular endothelial cells	*NIN*	0.5049	0.358	0.072	4.089e-73	9.443e-69
7	Blood vascular endothelial cells	*ATXN7L1*	0.5043	0.527	0.179	1.779e-46	4.109e-42
7	Blood vascular endothelial cells	*RB1*	0.5038	0.608	0.261	8.650e-33	1.997e-28
7	Blood vascular endothelial cells	*CD274*	0.5037	0.304	0.041	3.278e-97	7.570e-93
7	Blood vascular endothelial cells	*LOC127487360*	0.5035	0.370	0.111	1.007e-41	2.325e-37
7	Blood vascular endothelial cells	*ICA1*	0.5022	0.401	0.068	2.296e-98	5.303e-94
7	Blood vascular endothelial cells	*BCL10*	0.5014	0.386	0.089	2.818e-63	6.508e-59
7	Blood vascular endothelial cells	*CSRP2*	0.5008	0.506	0.240	6.678e-23	1.542e-18
7	Blood vascular endothelial cells	*PCSK6*	0.5007	0.416	0.093	4.976e-70	1.149e-65
7	Blood vascular endothelial cells	*FOXN3*	0.5005	0.620	0.269	8.132e-34	1.878e-29
7	Blood vascular endothelial cells	*HMGCLL1*	0.4995	0.166	0.043	7.172e-25	1.656e-20
7	Blood vascular endothelial cells	*CDC14A*	0.4993	0.352	0.069	8.454e-74	1.952e-69
7	Blood vascular endothelial cells	*LMCD1*	0.4989	0.310	0.043	2.073e-97	4.786e-93
7	Blood vascular endothelial cells	*RAP1A*	0.4983	0.687	0.337	3.477e-29	8.029e-25

Original cluster	Annotated cell type	Gene	avg_log2FC	pct.1	pct.2	Raw P value	Bonferroni-adjusted P value
7	Blood vascular endothelial cells	*RAC1*	0.4982	0.801	0.485	4.002e-24	9.240e-20
7	Blood vascular endothelial cells	*PLEKHG2*	0.4982	0.349	0.057	1.989e-91	4.593e-87
7	Blood vascular endothelial cells	*SERP1*	0.4977	0.771	0.418	1.414e-27	3.266e-23
7	Blood vascular endothelial cells	*NFKB1*	0.4970	0.545	0.203	5.480e-40	1.265e-35
7	Blood vascular endothelial cells	*FGL2*	0.4960	0.485	0.166	8.393e-43	1.938e-38
7	Blood vascular endothelial cells	*IER2*	0.4952	0.898	0.814	2.922e-18	6.748e-14
7	Blood vascular endothelial cells	*MMD*	0.4951	0.364	0.084	4.790e-61	1.106e-56
7	Blood vascular endothelial cells	*CILP*	0.4950	0.292	0.022	4.178e-164	9.649e-160
7	Blood vascular endothelial cells	*CD14*	0.4936	0.295	0.009	3.737e-293	8.630e-289
7	Blood vascular endothelial cells	*PROM1*	0.4903	0.322	0.020	8.956e-213	2.068e-208
7	Blood vascular endothelial cells	*PPP1R3B*	0.4901	0.310	0.040	1.741e-105	4.020e-101
7	Blood vascular endothelial cells	*PGK1*	0.4899	0.581	0.237	1.687e-35	3.896e-31
7	Blood vascular endothelial cells	*ECE1*	0.4895	0.479	0.168	5.028e-40	1.161e-35
7	Blood vascular endothelial cells	*FCHSD2*	0.4890	0.515	0.234	1.494e-25	3.449e-21
7	Blood vascular endothelial cells	*MLF2*	0.4890	0.479	0.123	4.272e-66	9.864e-62
7	Blood vascular endothelial cells	*TPM4*	0.4878	0.666	0.338	4.338e-27	1.002e-22
7	Blood vascular endothelial cells	*ARF4*	0.4876	0.774	0.515	1.750e-21	4.041e-17
7	Blood vascular endothelial cells	*NEURL1B*	0.4874	0.313	0.023	4.397e-179	1.015e-174
7	Blood vascular endothelial cells	*NRGN*	0.4872	0.247	0.007	2.694e-257	6.221e-253
7	Blood vascular endothelial cells	*MERTK*	0.4867	0.301	0.048	1.100e-80	2.541e-76
7	Blood vascular endothelial cells	*ZFC3H1*	0.4866	0.545	0.221	2.549e-33	5.886e-29
7	Blood vascular endothelial cells	*GSPT1*	0.4865	0.590	0.239	1.596e-36	3.685e-32
7	Blood vascular endothelial cells	*EMC10*	0.4861	0.530	0.191	2.648e-40	6.115e-36
7	Blood vascular endothelial cells	*C12H14orf119*	0.4858	0.437	0.092	7.539e-80	1.741e-75
7	Blood vascular endothelial cells	*INSR*	0.4857	0.611	0.340	4.774e-19	1.102e-14
7	Blood vascular endothelial cells	*BNIP2*	0.4855	0.461	0.149	3.459e-44	7.988e-40
7	Blood vascular endothelial cells	*HOXB7*	0.4855	0.262	0.014	1.684e-194	3.889e-190
7	Blood vascular endothelial cells	*KANK1*	0.4848	0.334	0.070	7.175e-64	1.657e-59
7	Blood vascular endothelial cells	*CYB5R3*	0.4840	0.681	0.326	2.331e-29	5.382e-25
7	Blood vascular endothelial cells	*CHCHD2*	0.4837	0.846	0.606	9.761e-25	2.254e-20
7	Blood vascular endothelial cells	*TMEM140*	0.4828	0.328	0.031	6.972e-151	1.610e-146
7	Blood vascular endothelial cells	*ITGB4*	0.4826	0.226	0.006	8.289e-255	1.914e-250
7	Blood vascular endothelial cells	*STARD7*	0.4825	0.410	0.084	1.909e-78	4.407e-74
7	Blood vascular endothelial cells	*EXOC5*	0.4819	0.449	0.150	6.990e-42	1.614e-37
7	Blood vascular endothelial cells	*RRBP1*	0.4814	0.596	0.286	1.077e-24	2.487e-20
7	Blood vascular endothelial cells	*ADGRL2*	0.4813	0.542	0.243	7.018e-26	1.621e-21
7	Blood vascular endothelial cells	*TPD52*	0.4809	0.262	0.048	1.812e-60	4.184e-56
7	Blood vascular endothelial cells	*TMEM170A*	0.4808	0.473	0.130	2.331e-59	5.383e-55
7	Blood vascular endothelial cells	*CDC42EP1*	0.4802	0.386	0.067	7.888e-92	1.822e-87
7	Blood vascular endothelial cells	*MTA2*	0.4794	0.355	0.048	4.826e-115	1.114e-110

Original cluster	Annotated cell type	Gene	avg_log2FC	pct.1	pct.2	Raw P value	Bonferroni-adjusted P value
7	Blood vascular endothelial cells	*GLRX2*	0.4791	0.413	0.096	1.291e-66	2.980e-62
7	Blood vascular endothelial cells	*YTHDF2*	0.4790	0.548	0.219	7.367e-35	1.701e-30
7	Blood vascular endothelial cells	*LOC100347754*	0.4790	0.340	0.049	1.332e-102	3.076e-98
7	Blood vascular endothelial cells	*MFSD6*	0.4789	0.271	0.024	1.538e-132	3.551e-128
7	Blood vascular endothelial cells	*AKIRIN1*	0.4787	0.410	0.080	3.302e-84	7.626e-80
7	Blood vascular endothelial cells	*CYB561A3*	0.4786	0.289	0.055	1.601e-63	3.697e-59
7	Blood vascular endothelial cells	*LOC103345647*	0.4786	0.548	0.204	8.017e-40	1.851e-35
7	Blood vascular endothelial cells	*PPARG*	0.4783	0.223	0.009	3.169e-194	7.317e-190
7	Blood vascular endothelial cells	*CCNL1*	0.4779	0.880	0.661	5.498e-22	1.270e-17
7	Blood vascular endothelial cells	*SMG1*	0.4764	0.587	0.267	1.317e-28	3.040e-24
7	Blood vascular endothelial cells	*EGLN3*	0.4756	0.235	0.030	7.821e-80	1.806e-75
7	Blood vascular endothelial cells	*TMEM204*	0.4744	0.392	0.108	1.743e-48	4.024e-44
7	Blood vascular endothelial cells	*ZNRF2*	0.4743	0.377	0.099	7.690e-52	1.776e-47
7	Blood vascular endothelial cells	*LOC138847146*	0.4740	0.217	0.000	0	0
7	Blood vascular endothelial cells	*PLXNA2*	0.4739	0.274	0.010	7.461e-258	1.723e-253
7	Blood vascular endothelial cells	*BHLHE40*	0.4732	0.482	0.160	1.283e-44	2.963e-40
7	Blood vascular endothelial cells	*SERTAD2*	0.4729	0.434	0.140	1.251e-40	2.888e-36
7	Blood vascular endothelial cells	*LOC108175596*	0.4724	0.367	0.060	1.196e-94	2.762e-90
7	Blood vascular endothelial cells	*RAB11FIP5*	0.4719	0.352	0.039	2.866e-140	6.619e-136
7	Blood vascular endothelial cells	*ITSN2*	0.4716	0.551	0.229	1.440e-32	3.324e-28
7	Blood vascular endothelial cells	*FAM124A*	0.4712	0.256	0.008	2.745e-262	6.339e-258
7	Blood vascular endothelial cells	*PPP1CA*	0.4710	0.569	0.225	1.584e-36	3.658e-32
7	Blood vascular endothelial cells	*ADH1A*	0.4703	0.133	0.001	2.256e-209	5.210e-205
7	Blood vascular endothelial cells	*SRRM2*	0.4691	0.955	0.730	4.708e-33	1.087e-28
7	Blood vascular endothelial cells	*OCIAD2*	0.4690	0.602	0.260	1.322e-32	3.054e-28
7	Blood vascular endothelial cells	*DBI*	0.4681	0.611	0.288	5.224e-28	1.206e-23
7	Blood vascular endothelial cells	*CLEC1A*	0.4672	0.232	0.001	0	0
7	Blood vascular endothelial cells	*RNF144B*	0.4670	0.280	0.025	3.704e-138	8.553e-134
7	Blood vascular endothelial cells	*PSMB8*	0.4664	0.328	0.030	9.003e-154	2.079e-149
7	Blood vascular endothelial cells	*UBR5*	0.4662	0.648	0.328	5.285e-26	1.220e-21
7	Blood vascular endothelial cells	*GRB10*	0.4662	0.367	0.098	2.031e-49	4.690e-45
7	Blood vascular endothelial cells	*PPM1H*	0.4655	0.301	0.027	1.748e-146	4.036e-142
7	Blood vascular endothelial cells	*TSPAN14*	0.4653	0.346	0.039	8.534e-135	1.971e-130
7	Blood vascular endothelial cells	*INPP1*	0.4644	0.605	0.251	1.340e-34	3.095e-30
7	Blood vascular endothelial cells	*RASSF9*	0.4642	0.262	0.018	3.219e-159	7.433e-155
7	Blood vascular endothelial cells	*ARHGAP20*	0.4630	0.485	0.223	5.215e-23	1.204e-18
7	Blood vascular endothelial cells	*NCEH1*	0.4627	0.274	0.064	8.828e-45	2.039e-40
7	Blood vascular endothelial cells	*TRIB1*	0.4621	0.274	0.027	5.257e-121	1.214e-116
7	Blood vascular endothelial cells	*LPAR6*	0.4614	0.340	0.033	6.988e-150	1.614e-145
7	Blood vascular endothelial cells	*MYO6*	0.4610	0.425	0.101	6.180e-67	1.427e-62

Original cluster	Annotated cell type	Gene	avg_log2FC	pct.1	pct.2	Raw P value	Bonferroni-adjusted P value
7	Blood vascular endothelial cells	*MAML3*	0.4609	0.542	0.231	8.547e-31	1.974e-26
7	Blood vascular endothelial cells	*TRIR*	0.4609	0.840	0.539	4.720e-24	1.090e-19
7	Blood vascular endothelial cells	*TMEM9B*	0.4605	0.358	0.068	1.227e-77	2.834e-73
7	Blood vascular endothelial cells	*IFIT5*	0.4602	0.268	0.032	5.327e-100	1.230e-95
7	Blood vascular endothelial cells	*RAB13*	0.4598	0.563	0.221	3.122e-34	7.208e-30
7	Blood vascular endothelial cells	*PIK3C2B*	0.4596	0.298	0.029	4.916e-133	1.135e-128
7	Blood vascular endothelial cells	*GNAS*	0.4585	0.946	0.879	1.357e-28	3.135e-24
7	Blood vascular endothelial cells	*ELK4*	0.4579	0.482	0.166	5.266e-41	1.216e-36
7	Blood vascular endothelial cells	*CTNNB1*	0.4578	0.834	0.493	9.633e-31	2.224e-26
7	Blood vascular endothelial cells	*TOMM20*	0.4575	0.711	0.399	2.579e-23	5.956e-19
7	Blood vascular endothelial cells	*CEP68*	0.4574	0.473	0.143	1.531e-50	3.535e-46
7	Blood vascular endothelial cells	*BTBD3*	0.4574	0.627	0.320	4.347e-23	1.004e-18
7	Blood vascular endothelial cells	*SAR1A*	0.4567	0.452	0.199	2.307e-22	5.328e-18
7	Blood vascular endothelial cells	*VPS13D*	0.4557	0.449	0.158	1.231e-35	2.842e-31
7	Blood vascular endothelial cells	*LFNG*	0.4554	0.235	0.015	2.627e-147	6.066e-143
7	Blood vascular endothelial cells	*AZIN1*	0.4554	0.648	0.313	1.363e-28	3.148e-24
7	Blood vascular endothelial cells	*LNPEP*	0.4551	0.395	0.108	3.736e-51	8.628e-47
7	Blood vascular endothelial cells	*CTTNBP2NL*	0.4547	0.343	0.051	1.803e-99	4.164e-95
7	Blood vascular endothelial cells	*RBKS*	0.4546	0.295	0.056	5.026e-64	1.160e-59
7	Blood vascular endothelial cells	*ACER2*	0.4545	0.262	0.031	1.389e-96	3.207e-92
7	Blood vascular endothelial cells	*PXDC1*	0.4544	0.389	0.075	1.128e-80	2.605e-76
7	Blood vascular endothelial cells	*MEF2A*	0.4541	0.672	0.328	2.011e-28	4.644e-24
7	Blood vascular endothelial cells	*CCDC88C*	0.4541	0.298	0.009	7.736e-306	1.786e-301
7	Blood vascular endothelial cells	*PCNX1*	0.4526	0.560	0.241	3.503e-29	8.088e-25
7	Blood vascular endothelial cells	*SPAG9*	0.4519	0.578	0.292	6.660e-22	1.538e-17
7	Blood vascular endothelial cells	*FZD6*	0.4518	0.343	0.062	1.016e-78	2.345e-74
7	Blood vascular endothelial cells	*CHST6*	0.4516	0.217	0.004	1.709e-269	3.945e-265
7	Blood vascular endothelial cells	*DCLK2*	0.4515	0.259	0.022	1.726e-130	3.986e-126
7	Blood vascular endothelial cells	*BBLN*	0.4512	0.431	0.091	8.963e-79	2.070e-74
7	Blood vascular endothelial cells	*REXO2*	0.4510	0.464	0.147	1.094e-45	2.525e-41
7	Blood vascular endothelial cells	*HID1*	0.4507	0.259	0.007	4.594e-291	1.061e-286
7	Blood vascular endothelial cells	*TRPM6*	0.4500	0.175	0.010	1.647e-124	3.803e-120
7	Blood vascular endothelial cells	*NUDT14*	0.4497	0.340	0.065	9.512e-73	2.196e-68
7	Blood vascular endothelial cells	*RAB5C*	0.4480	0.506	0.169	6.328e-44	1.461e-39
7	Blood vascular endothelial cells	*PLEKHF2*	0.4474	0.349	0.106	5.191e-38	1.199e-33
7	Blood vascular endothelial cells	*PTK2*	0.4474	0.599	0.227	1.452e-41	3.354e-37
7	Blood vascular endothelial cells	*EIF2AK2*	0.4473	0.328	0.068	1.068e-62	2.467e-58
7	Blood vascular endothelial cells	*GNB4*	0.4462	0.494	0.174	1.546e-38	3.569e-34
7	Blood vascular endothelial cells	*PKN3*	0.4462	0.238	0.004	0	0
7	Blood vascular endothelial cells	*TSPAN9*	0.4460	0.352	0.047	4.074e-114	9.407e-110

Original cluster	Annotated cell type	Gene	avg_log2FC	pct.1	pct.2	Raw P value	Bonferroni-adjusted P value
7	Blood vascular endothelial cells	*LOC100346985*	0.4455	0.247	0.025	3.598e-107	8.308e-103
7	Blood vascular endothelial cells	*ATP5F1B*	0.4449	0.684	0.360	1.368e-22	3.158e-18
7	Blood vascular endothelial cells	*GOLGA7*	0.4445	0.428	0.143	4.522e-38	1.044e-33
7	Blood vascular endothelial cells	*AOX1*	0.4433	0.416	0.106	3.948e-58	9.117e-54
7	Blood vascular endothelial cells	*HIVEP1*	0.4432	0.364	0.088	2.841e-56	6.561e-52
7	Blood vascular endothelial cells	*VSIR*	0.4429	0.340	0.056	9.956e-87	2.299e-82
7	Blood vascular endothelial cells	*NLK*	0.4427	0.422	0.125	4.567e-47	1.055e-42
7	Blood vascular endothelial cells	*ITGA5*	0.4427	0.274	0.018	1.017e-172	2.348e-168
7	Blood vascular endothelial cells	*ABI1*	0.4426	0.608	0.271	1.347e-29	3.109e-25
7	Blood vascular endothelial cells	*PKIG*	0.4426	0.416	0.112	2.142e-53	4.947e-49
7	Blood vascular endothelial cells	*STAU1*	0.4426	0.452	0.150	2.836e-41	6.549e-37
7	Blood vascular endothelial cells	*CHD7*	0.4423	0.319	0.028	3.461e-159	7.991e-155
7	Blood vascular endothelial cells	*HIPK3*	0.4422	0.639	0.336	8.831e-24	2.039e-19
7	Blood vascular endothelial cells	*LARP4*	0.4419	0.527	0.182	4.064e-42	9.386e-38
7	Blood vascular endothelial cells	*SLC16A13*	0.4416	0.238	0.007	2.226e-250	5.141e-246
7	Blood vascular endothelial cells	*ARPC1B*	0.4414	0.518	0.115	1.079e-88	2.492e-84
7	Blood vascular endothelial cells	*LOC100346553*	0.4411	0.229	0.006	1.156e-254	2.669e-250
7	Blood vascular endothelial cells	*RAB2A*	0.4407	0.780	0.479	3.161e-22	7.299e-18
7	Blood vascular endothelial cells	*LOC108178130*	0.4404	0.154	0.001	9.988e-263	2.306e-258
7	Blood vascular endothelial cells	*TGFB1*	0.4399	0.346	0.062	2.564e-80	5.920e-76
7	Blood vascular endothelial cells	*ATP1B3*	0.4397	0.509	0.166	2.245e-47	5.185e-43
7	Blood vascular endothelial cells	*ABHD17B*	0.4396	0.440	0.148	1.314e-38	3.035e-34
7	Blood vascular endothelial cells	*LRP5*	0.4393	0.367	0.061	5.136e-93	1.186e-88
7	Blood vascular endothelial cells	*BACC1*	0.4386	0.627	0.269	2.258e-31	5.213e-27
7	Blood vascular endothelial cells	*SNX9*	0.4383	0.473	0.178	6.787e-33	1.567e-28
7	Blood vascular endothelial cells	*SERTAD1*	0.4383	0.816	0.596	5.600e-22	1.293e-17
7	Blood vascular endothelial cells	*EGR3*	0.4379	0.271	0.026	4.741e-125	1.095e-120
7	Blood vascular endothelial cells	*PIK3R4*	0.4378	0.401	0.095	1.232e-62	2.845e-58
7	Blood vascular endothelial cells	*CHMP2A*	0.4370	0.639	0.292	1.487e-29	3.434e-25
7	Blood vascular endothelial cells	*HAPSTR1*	0.4366	0.524	0.195	6.487e-37	1.498e-32
7	Blood vascular endothelial cells	*HMG20B*	0.4357	0.437	0.116	3.005e-57	6.939e-53
7	Blood vascular endothelial cells	*NCKAP1*	0.4353	0.608	0.268	7.505e-29	1.733e-24
7	Blood vascular endothelial cells	*RFC3*	0.4353	0.377	0.119	1.412e-37	3.260e-33
7	Blood vascular endothelial cells	*SERINC3*	0.4349	0.578	0.269	1.053e-24	2.431e-20
7	Blood vascular endothelial cells	*STAB1*	0.4349	0.289	0.008	0	0
7	Blood vascular endothelial cells	*GYPC*	0.4349	0.642	0.365	6.200e-19	1.432e-14
7	Blood vascular endothelial cells	*TPR*	0.4345	0.681	0.381	1.487e-21	3.433e-17
7	Blood vascular endothelial cells	*GJA1*	0.4339	0.286	0.104	3.562e-23	8.226e-19
7	Blood vascular endothelial cells	*LOC138844116*	0.4337	0.193	0.046	1.408e-31	3.252e-27
7	Blood vascular endothelial cells	*DEGS1*	0.4333	0.470	0.166	1.739e-35	4.015e-31

Original cluster	Annotated cell type	Gene	avg_log2FC	pct.1	pct.2	Raw P value	Bonferroni-adjusted P value
7	Blood vascular endothelial cells	*SPRY1*	0.4328	0.334	0.073	6.835e-59	1.578e-54
7	Blood vascular endothelial cells	*WWC2*	0.4321	0.590	0.279	6.532e-25	1.508e-20
7	Blood vascular endothelial cells	*VASP*	0.4321	0.422	0.097	3.391e-69	7.831e-65
7	Blood vascular endothelial cells	*TAOK2*	0.4319	0.343	0.058	4.236e-85	9.781e-81
7	Blood vascular endothelial cells	*SIPA1*	0.4318	0.307	0.031	1.227e-133	2.834e-129
7	Blood vascular endothelial cells	*ITIH5*	0.4309	0.328	0.093	7.094e-40	1.638e-35
7	Blood vascular endothelial cells	*LOC138844757*	0.4309	0.247	0.033	4.650e-81	1.074e-76
7	Blood vascular endothelial cells	*KIAA0040*	0.4305	0.298	0.028	5.000e-136	1.155e-131
7	Blood vascular endothelial cells	*FUCA1*	0.4299	0.494	0.196	2.094e-31	4.835e-27
7	Blood vascular endothelial cells	*CISH*	0.4296	0.355	0.065	8.098e-80	1.870e-75
7	Blood vascular endothelial cells	*FLOT2*	0.4287	0.386	0.092	2.778e-60	6.414e-56
7	Blood vascular endothelial cells	*SYPL1*	0.4283	0.551	0.214	4.445e-36	1.026e-31
7	Blood vascular endothelial cells	*PDK4*	0.4281	0.316	0.157	2.808e-12	6.484e-08
7	Blood vascular endothelial cells	*RNASEK*	0.4275	0.684	0.340	7.434e-27	1.717e-22
7	Blood vascular endothelial cells	*TRAK2*	0.4261	0.322	0.083	2.486e-46	5.740e-42
7	Blood vascular endothelial cells	*IQSEC1*	0.4258	0.373	0.085	8.083e-62	1.867e-57
7	Blood vascular endothelial cells	*RABGAP1L*	0.4245	0.657	0.358	6.773e-21	1.564e-16
7	Blood vascular endothelial cells	*SLC20A1*	0.4240	0.389	0.129	8.543e-36	1.973e-31
7	Blood vascular endothelial cells	*MPZL1*	0.4239	0.542	0.177	2.912e-48	6.724e-44
7	Blood vascular endothelial cells	*NTAN1*	0.4237	0.563	0.250	3.048e-26	7.038e-22
7	Blood vascular endothelial cells	*LOC100358813*	0.4234	0.886	0.713	1.297e-22	2.995e-18
7	Blood vascular endothelial cells	*PRR5L*	0.4234	0.262	0.022	1.151e-133	2.657e-129
7	Blood vascular endothelial cells	*CAMSAP2*	0.4232	0.572	0.275	3.069e-24	7.086e-20
7	Blood vascular endothelial cells	*GNG5*	0.4232	0.783	0.474	5.777e-19	1.334e-14
7	Blood vascular endothelial cells	*HUWE1*	0.4228	0.660	0.326	3.897e-25	9.000e-21
7	Blood vascular endothelial cells	*LOC100358069*	0.4228	0.880	0.656	3.276e-17	7.566e-13
7	Blood vascular endothelial cells	*GPRC5B*	0.4221	0.277	0.017	9.427e-181	2.177e-176
7	Blood vascular endothelial cells	*NECTIN2*	0.4218	0.346	0.066	5.927e-74	1.369e-69
7	Blood vascular endothelial cells	*PSMB3*	0.4217	0.548	0.200	6.039e-39	1.395e-34
7	Blood vascular endothelial cells	*PRCP*	0.4214	0.226	0.026	1.212e-86	2.799e-82
7	Blood vascular endothelial cells	*TET2*	0.4214	0.542	0.233	1.984e-29	4.582e-25
7	Blood vascular endothelial cells	*UNC13B*	0.4210	0.307	0.044	2.590e-94	5.981e-90
7	Blood vascular endothelial cells	*LRRC32*	0.4205	0.337	0.054	5.356e-88	1.237e-83
7	Blood vascular endothelial cells	*DUSP1*	0.4200	0.705	0.447	2.182e-18	5.039e-14
7	Blood vascular endothelial cells	*MAP4K4*	0.4197	0.717	0.392	2.605e-23	6.016e-19
7	Blood vascular endothelial cells	*SET*	0.4196	0.672	0.319	7.415e-28	1.712e-23
7	Blood vascular endothelial cells	*DTNBP1*	0.4194	0.343	0.096	2.786e-42	6.433e-38
7	Blood vascular endothelial cells	*FAM53B*	0.4187	0.259	0.030	1.030e-97	2.377e-93
7	Blood vascular endothelial cells	*RIPPLY3*	0.4179	0.235	0.015	6.977e-147	1.611e-142
7	Blood vascular endothelial cells	*UGCG*	0.4178	0.491	0.185	5.222e-33	1.206e-28

Original cluster	Annotated cell type	Gene	avg_log2FC	pct.1	pct.2	Raw P value	Bonferroni-adjusted P value
7	Blood vascular endothelial cells	*PNPLA2*	0.4176	0.346	0.071	4.762e-67	1.100e-62
7	Blood vascular endothelial cells	*FUT8*	0.4168	0.431	0.157	1.776e-33	4.100e-29
7	Blood vascular endothelial cells	*LRBA*	0.4167	0.614	0.349	4.885e-15	1.128e-10
7	Blood vascular endothelial cells	*LOC100347604*	0.4167	0.196	0.001	0	0
7	Blood vascular endothelial cells	*LOC138843475*	0.4167	0.925	0.796	4.383e-21	1.012e-16
7	Blood vascular endothelial cells	*LPAR4*	0.4151	0.316	0.053	1.625e-78	3.753e-74
7	Blood vascular endothelial cells	*TBC1D1*	0.4145	0.355	0.078	3.135e-63	7.239e-59
7	Blood vascular endothelial cells	*ST3GAL2*	0.4144	0.392	0.095	2.415e-59	5.577e-55
7	Blood vascular endothelial cells	*ZNF438*	0.4144	0.428	0.122	8.613e-50	1.989e-45
7	Blood vascular endothelial cells	*COQ10B*	0.4141	0.596	0.248	1.575e-34	3.637e-30
7	Blood vascular endothelial cells	*NETO2*	0.4138	0.340	0.099	2.739e-39	6.324e-35
7	Blood vascular endothelial cells	*ANXA7*	0.4137	0.533	0.204	6.871e-34	1.587e-29
7	Blood vascular endothelial cells	*NDST1*	0.4135	0.322	0.044	4.281e-103	9.885e-99
7	Blood vascular endothelial cells	*HIGD1A*	0.4133	0.298	0.039	2.802e-99	6.471e-95
7	Blood vascular endothelial cells	*VPS36*	0.4125	0.464	0.142	4.725e-48	1.091e-43
7	Blood vascular endothelial cells	*RDH10*	0.4124	0.268	0.058	2.924e-49	6.752e-45
7	Blood vascular endothelial cells	*ATF2*	0.4115	0.488	0.258	5.362e-14	1.238e-09
7	Blood vascular endothelial cells	*SEC31A*	0.4111	0.467	0.179	2.039e-31	4.708e-27
7	Blood vascular endothelial cells	*NR3C1*	0.4104	0.741	0.414	1.314e-23	3.034e-19
7	Blood vascular endothelial cells	*FOXO1*	0.4092	0.651	0.281	8.837e-35	2.041e-30
7	Blood vascular endothelial cells	*LOC127492483*	0.4091	0.343	0.118	4.456e-30	1.029e-25
7	Blood vascular endothelial cells	*KDM7A*	0.4082	0.419	0.134	6.756e-40	1.560e-35
7	Blood vascular endothelial cells	*TEAD4*	0.4082	0.244	0.009	1.845e-221	4.261e-217
7	Blood vascular endothelial cells	*RGS6*	0.4080	0.377	0.177	9.650e-18	2.228e-13
7	Blood vascular endothelial cells	*FRMD4B*	0.4078	0.509	0.202	4.417e-32	1.020e-27
7	Blood vascular endothelial cells	*ZNFX1*	0.4052	0.352	0.077	5.745e-62	1.327e-57
7	Blood vascular endothelial cells	*YBX1*	0.4051	0.861	0.690	1.567e-16	3.619e-12
7	Blood vascular endothelial cells	*DRAM1*	0.4050	0.286	0.034	1.270e-104	2.933e-100
7	Blood vascular endothelial cells	*BCL2L1*	0.4049	0.307	0.058	9.691e-67	2.238e-62
7	Blood vascular endothelial cells	*SAMD9*	0.4044	0.349	0.155	9.383e-19	2.167e-14
7	Blood vascular endothelial cells	*CYB5D1*	0.4044	0.208	0.041	4.348e-44	1.004e-39
7	Blood vascular endothelial cells	*ARHGDIA*	0.4035	0.678	0.362	8.763e-22	2.024e-17
7	Blood vascular endothelial cells	*CTNNBIP1*	0.4034	0.515	0.176	1.629e-41	3.761e-37
7	Blood vascular endothelial cells	*LOC138843527*	0.4031	0.892	0.654	5.126e-21	1.184e-16
7	Blood vascular endothelial cells	*HEY1*	0.4030	0.214	0.049	4.545e-37	1.049e-32
7	Blood vascular endothelial cells	*ZBTB37*	0.4029	0.467	0.164	1.099e-35	2.538e-31
7	Blood vascular endothelial cells	*LOC127483734*	0.4021	0.247	0.016	4.891e-157	1.129e-152
7	Blood vascular endothelial cells	*SMC1A*	0.4017	0.500	0.204	3.113e-29	7.188e-25
7	Blood vascular endothelial cells	*ACER3*	0.4014	0.401	0.150	7.606e-29	1.756e-24
7	Blood vascular endothelial cells	*ADRB2*	0.4012	0.187	0.010	1.249e-138	2.884e-134

Original cluster	Annotated cell type	Gene	avg_log2FC	pct.1	pct.2	Raw P value	Bonferroni-adjusted P value
7	Blood vascular endothelial cells	*MAL*	0.4012	0.205	0.007	5.993e-197	1.384e-192
7	Blood vascular endothelial cells	*YWHAG*	0.4011	0.539	0.206	1.667e-34	3.850e-30
7	Blood vascular endothelial cells	*LOC103350988*	0.4011	0.247	0.036	1.117e-73	2.580e-69
7	Blood vascular endothelial cells	*PSME1*	0.4009	0.545	0.225	1.856e-30	4.287e-26
7	Blood vascular endothelial cells	*STK3*	0.4008	0.675	0.390	7.379e-17	1.704e-12
7	Blood vascular endothelial cells	*RAB1A*	0.4003	0.732	0.425	7.532e-21	1.739e-16
7	Blood vascular endothelial cells	*FAR2*	0.3993	0.214	0.020	7.121e-101	1.644e-96
7	Blood vascular endothelial cells	*UBE2D1*	0.3989	0.452	0.139	1.339e-45	3.092e-41
7	Blood vascular endothelial cells	*IFIT3*	0.3988	0.217	0.014	2.710e-138	6.257e-134
7	Blood vascular endothelial cells	*NR6A1*	0.3983	0.232	0.049	1.576e-44	3.639e-40
7	Blood vascular endothelial cells	*ADAM15*	0.3980	0.283	0.039	3.913e-90	9.036e-86
7	Blood vascular endothelial cells	*DERA*	0.3979	0.485	0.178	1.081e-34	2.496e-30
7	Blood vascular endothelial cells	*ENY2*	0.3979	0.726	0.416	3.378e-21	7.800e-17
7	Blood vascular endothelial cells	*DAZAP2*	0.3976	0.431	0.139	1.906e-40	4.402e-36
7	Blood vascular endothelial cells	*PFN1*	0.3972	0.852	0.551	2.484e-26	5.735e-22
7	Blood vascular endothelial cells	*EPHB4*	0.3958	0.310	0.044	6.129e-94	1.415e-89
7	Blood vascular endothelial cells	*TAX1BP3*	0.3958	0.364	0.066	2.071e-81	4.783e-77
7	Blood vascular endothelial cells	*VEZF1*	0.3955	0.617	0.311	3.706e-22	8.558e-18
7	Blood vascular endothelial cells	*GRPEL2*	0.3955	0.373	0.094	4.806e-53	1.110e-48
7	Blood vascular endothelial cells	*RMND5A*	0.3951	0.566	0.243	1.745e-28	4.030e-24
7	Blood vascular endothelial cells	*KLHL23*	0.3949	0.361	0.093	1.515e-49	3.497e-45
7	Blood vascular endothelial cells	*LIMA1*	0.3949	0.322	0.082	3.259e-46	7.526e-42
7	Blood vascular endothelial cells	*USP3*	0.3949	0.479	0.166	3.967e-39	9.161e-35
7	Blood vascular endothelial cells	*FMNL3*	0.3948	0.280	0.026	1.482e-129	3.423e-125
7	Blood vascular endothelial cells	*H1-4*	0.3948	0.304	0.075	1.075e-45	2.481e-41
7	Blood vascular endothelial cells	*YY1*	0.3947	0.581	0.275	1.664e-24	3.843e-20
7	Blood vascular endothelial cells	*ZNF608*	0.3941	0.259	0.045	2.727e-62	6.297e-58
7	Blood vascular endothelial cells	*USP25*	0.3932	0.530	0.249	1.996e-22	4.608e-18
7	Blood vascular endothelial cells	*ATP5MC2*	0.3932	0.925	0.751	1.726e-18	3.987e-14
7	Blood vascular endothelial cells	*EIF4A1*	0.3919	0.825	0.652	1.838e-12	4.244e-08
7	Blood vascular endothelial cells	*BLTP2*	0.3916	0.377	0.106	4.028e-46	9.302e-42
7	Blood vascular endothelial cells	*PPP4R1*	0.3910	0.539	0.195	1.077e-39	2.486e-35
7	Blood vascular endothelial cells	*PTPN4*	0.3909	0.530	0.222	3.573e-29	8.252e-25
7	Blood vascular endothelial cells	*SLC16A12*	0.3909	0.187	0.008	2.792e-162	6.447e-158
7	Blood vascular endothelial cells	*AAK1*	0.3908	0.455	0.137	6.203e-47	1.432e-42
7	Blood vascular endothelial cells	*MTHFD1L*	0.3903	0.289	0.094	1.671e-28	3.858e-24
7	Blood vascular endothelial cells	*BCLAF1*	0.3892	0.801	0.511	3.163e-18	7.304e-14
7	Blood vascular endothelial cells	*M6PR*	0.3891	0.584	0.269	1.653e-24	3.816e-20
7	Blood vascular endothelial cells	*SOS1*	0.3889	0.488	0.201	1.702e-28	3.930e-24
7	Blood vascular endothelial cells	*UBE2J1*	0.3888	0.404	0.111	1.917e-49	4.428e-45

Original cluster	Annotated cell type	Gene	avg_log2FC	pct.1	pct.2	Raw P value	Bonferroni-adjusted P value
7	Blood vascular endothelial cells	*NR2C2*	0.3885	0.380	0.123	2.546e-35	5.878e-31
7	Blood vascular endothelial cells	*MEMO1*	0.3882	0.373	0.102	1.357e-47	3.133e-43
7	Blood vascular endothelial cells	*IQCN*	0.3880	0.404	0.146	2.617e-30	6.044e-26
7	Blood vascular endothelial cells	*ANXA5*	0.3880	0.768	0.455	1.843e-19	4.255e-15
7	Blood vascular endothelial cells	*ERBIN*	0.3879	0.684	0.381	5.499e-20	1.270e-15
7	Blood vascular endothelial cells	*EIF4G2*	0.3878	0.816	0.586	2.728e-16	6.299e-12
7	Blood vascular endothelial cells	*JAK1*	0.3871	0.774	0.497	5.463e-17	1.262e-12
7	Blood vascular endothelial cells	*MTF2*	0.3862	0.660	0.339	5.203e-22	1.202e-17
7	Blood vascular endothelial cells	*STAT3*	0.3857	0.768	0.472	1.572e-19	3.631e-15
7	Blood vascular endothelial cells	*FAM43A*	0.3856	0.458	0.204	2.743e-23	6.333e-19
7	Blood vascular endothelial cells	*ATF7IP2*	0.3856	0.220	0.025	3.964e-85	9.154e-81
7	Blood vascular endothelial cells	*PDLIM1*	0.3852	0.289	0.047	1.837e-75	4.241e-71
7	Blood vascular endothelial cells	*ERF*	0.3848	0.422	0.122	1.865e-47	4.306e-43
7	Blood vascular endothelial cells	*LITAF*	0.3839	0.325	0.074	3.316e-55	7.657e-51
7	Blood vascular endothelial cells	*SIGIRR*	0.3820	0.241	0.010	2.863e-207	6.611e-203
7	Blood vascular endothelial cells	*PDCL*	0.3818	0.283	0.037	6.878e-94	1.588e-89
7	Blood vascular endothelial cells	*CCM2L*	0.3817	0.217	0.002	0	0
7	Blood vascular endothelial cells	*PARP14*	0.3811	0.295	0.068	2.825e-48	6.523e-44
7	Blood vascular endothelial cells	*SIK1*	0.3805	0.367	0.098	3.546e-48	8.188e-44
7	Blood vascular endothelial cells	*LBR*	0.3801	0.461	0.137	1.193e-48	2.754e-44
7	Blood vascular endothelial cells	*CDKN2AIP*	0.3801	0.575	0.285	2.441e-20	5.637e-16
7	Blood vascular endothelial cells	*DHRS7*	0.3799	0.298	0.049	5.438e-76	1.256e-71
7	Blood vascular endothelial cells	*MAPK1IP1L*	0.3796	0.446	0.152	5.489e-37	1.268e-32
7	Blood vascular endothelial cells	*PPP1R16B*	0.3794	0.205	0.007	2.050e-191	4.733e-187
7	Blood vascular endothelial cells	*PGM2L1*	0.3792	0.337	0.111	1.911e-31	4.413e-27
7	Blood vascular endothelial cells	*PDE4B*	0.3790	0.202	0.031	7.869e-57	1.817e-52
7	Blood vascular endothelial cells	*MMAA*	0.3788	0.286	0.093	6.749e-28	1.559e-23
7	Blood vascular endothelial cells	*G3BP1*	0.3788	0.672	0.312	9.511e-26	2.196e-21
7	Blood vascular endothelial cells	*SLK*	0.3775	0.590	0.279	3.099e-24	7.157e-20
7	Blood vascular endothelial cells	*RHOB*	0.3769	0.886	0.701	2.750e-14	6.351e-10
7	Blood vascular endothelial cells	*IPO7*	0.3768	0.488	0.186	1.749e-32	4.039e-28
7	Blood vascular endothelial cells	*FKBP5*	0.3767	0.262	0.030	1.572e-100	3.629e-96
7	Blood vascular endothelial cells	*LOC108177076*	0.3766	0.205	0.000	0	0
7	Blood vascular endothelial cells	*LOC100351528*	0.3761	0.148	0.000	1.480e-283	3.418e-279
7	Blood vascular endothelial cells	*EIF3J*	0.3760	0.563	0.236	8.195e-30	1.892e-25
7	Blood vascular endothelial cells	*NUFIP2*	0.3759	0.717	0.425	1.808e-18	4.176e-14
7	Blood vascular endothelial cells	*EEA1*	0.3759	0.660	0.367	1.085e-15	2.506e-11
7	Blood vascular endothelial cells	*RBMS2*	0.3758	0.428	0.134	1.351e-40	3.119e-36
7	Blood vascular endothelial cells	*WNK1*	0.3756	0.693	0.327	8.385e-30	1.936e-25
7	Blood vascular endothelial cells	*ERC1*	0.3755	0.578	0.295	3.046e-18	7.034e-14

Original cluster	Annotated cell type	Gene	avg_log2FC	pct.1	pct.2	Raw P value	Bonferroni-adjusted P value
7	Blood vascular endothelial cells	*PCBP2*	0.3755	0.831	0.631	2.569e-16	5.931e-12
7	Blood vascular endothelial cells	*IL12RB2*	0.3748	0.211	0.012	2.184e-142	5.043e-138
7	Blood vascular endothelial cells	*KMT2C*	0.3748	0.780	0.466	9.395e-18	2.169e-13
7	Blood vascular endothelial cells	*STARD9*	0.3741	0.355	0.098	2.330e-43	5.382e-39
7	Blood vascular endothelial cells	*NAA30*	0.3741	0.383	0.087	7.835e-62	1.809e-57
7	Blood vascular endothelial cells	*BRD2*	0.3733	0.699	0.435	6.587e-16	1.521e-11
7	Blood vascular endothelial cells	*HSPA4*	0.3729	0.497	0.200	1.629e-29	3.762e-25
7	Blood vascular endothelial cells	*PAWR*	0.3727	0.500	0.177	1.167e-38	2.694e-34
7	Blood vascular endothelial cells	*NCKAP1L*	0.3724	0.229	0.012	8.346e-168	1.927e-163
7	Blood vascular endothelial cells	*BAZ2B*	0.3723	0.753	0.584	1.134e-08	2.619e-04
7	Blood vascular endothelial cells	*ACAP2*	0.3710	0.630	0.328	1.061e-20	2.450e-16
7	Blood vascular endothelial cells	*TRIOBP*	0.3708	0.377	0.112	2.852e-41	6.586e-37
7	Blood vascular endothelial cells	*ZFAND3*	0.3707	0.581	0.290	1.402e-21	3.238e-17
7	Blood vascular endothelial cells	*TMEM44*	0.3703	0.217	0.007	8.226e-219	1.900e-214
7	Blood vascular endothelial cells	*LOC103345076*	0.3700	0.304	0.039	1.118e-101	2.582e-97
7	Blood vascular endothelial cells	*CAMTA1*	0.3700	0.645	0.301	4.501e-24	1.039e-19
7	Blood vascular endothelial cells	*LOC138849218*	0.3692	0.214	0.004	1.848e-266	4.268e-262
7	Blood vascular endothelial cells	*CYTH2*	0.3691	0.407	0.123	2.044e-42	4.719e-38
7	Blood vascular endothelial cells	*YIPF4*	0.3690	0.581	0.268	5.770e-25	1.332e-20
7	Blood vascular endothelial cells	*CHMP5*	0.3685	0.563	0.240	5.059e-28	1.168e-23
7	Blood vascular endothelial cells	*GPRC5A*	0.3685	0.268	0.040	5.701e-78	1.317e-73
7	Blood vascular endothelial cells	*NFKBID*	0.3684	0.232	0.021	1.421e-112	3.282e-108
7	Blood vascular endothelial cells	*APH1B*	0.3680	0.340	0.079	3.008e-55	6.946e-51
7	Blood vascular endothelial cells	*HOXD9*	0.3679	0.455	0.165	7.025e-33	1.622e-28
7	Blood vascular endothelial cells	*LOC138843111*	0.3678	0.220	0.012	3.933e-156	9.082e-152
7	Blood vascular endothelial cells	*ASAP2*	0.3676	0.358	0.103	5.558e-42	1.284e-37
7	Blood vascular endothelial cells	*LOC138848813*	0.3676	0.735	0.474	8.403e-16	1.940e-11
7	Blood vascular endothelial cells	*CAST*	0.3676	0.557	0.254	1.477e-25	3.410e-21
7	Blood vascular endothelial cells	*GIT2*	0.3675	0.386	0.114	1.961e-41	4.528e-37
7	Blood vascular endothelial cells	*GPR146*	0.3657	0.259	0.046	5.154e-61	1.190e-56
7	Blood vascular endothelial cells	*KLF7*	0.3657	0.518	0.223	1.431e-25	3.305e-21
7	Blood vascular endothelial cells	*KATNBL1*	0.3657	0.416	0.154	2.528e-30	5.838e-26
7	Blood vascular endothelial cells	*CLIC5*	0.3654	0.151	0.001	3.752e-267	8.664e-263
7	Blood vascular endothelial cells	*ITGA2*	0.3652	0.259	0.066	4.035e-37	9.316e-33
7	Blood vascular endothelial cells	*PRRG1*	0.3647	0.301	0.088	6.552e-35	1.513e-30
7	Blood vascular endothelial cells	*ZFAND5*	0.3639	0.377	0.113	2.275e-40	5.253e-36
7	Blood vascular endothelial cells	*SPPL2A*	0.3632	0.476	0.198	5.581e-27	1.289e-22
7	Blood vascular endothelial cells	*KLF3*	0.3623	0.419	0.166	6.524e-26	1.507e-21
7	Blood vascular endothelial cells	*MFHAS1*	0.3620	0.301	0.069	1.379e-49	3.185e-45
7	Blood vascular endothelial cells	*AHNAK*	0.3618	0.813	0.625	4.544e-11	1.049e-06

Original cluster	Annotated cell type	Gene	avg_log2FC	pct.1	pct.2	Raw P value	Bonferroni-adjusted P value
7	Blood vascular endothelial cells	*AMOTL2*	0.3614	0.334	0.079	1.861e-52	4.298e-48
7	Blood vascular endothelial cells	*KLF13*	0.3614	0.482	0.164	3.100e-39	7.158e-35
7	Blood vascular endothelial cells	*RELA*	0.3613	0.458	0.162	7.889e-35	1.822e-30
7	Blood vascular endothelial cells	*NPR1*	0.3612	0.235	0.024	2.146e-99	4.955e-95
7	Blood vascular endothelial cells	*LOC127488967*	0.3610	0.232	0.004	1.790e-306	4.135e-302
7	Blood vascular endothelial cells	*HDAC8*	0.3607	0.440	0.188	1.124e-23	2.596e-19
7	Blood vascular endothelial cells	*DDX21*	0.3601	0.386	0.151	2.514e-25	5.805e-21
7	Blood vascular endothelial cells	*MCF2L2*	0.3594	0.190	0.012	8.117e-123	1.874e-118
7	Blood vascular endothelial cells	*PLEKHA1*	0.3591	0.364	0.095	7.065e-49	1.632e-44
7	Blood vascular endothelial cells	*CERT1*	0.3589	0.437	0.155	2.846e-34	6.571e-30
7	Blood vascular endothelial cells	*LOC127489744*	0.3587	0.491	0.183	1.053e-32	2.432e-28
7	Blood vascular endothelial cells	*PRLR*	0.3587	0.139	0.014	1.845e-62	4.259e-58
7	Blood vascular endothelial cells	*TP53*	0.3587	0.355	0.093	8.699e-47	2.009e-42
7	Blood vascular endothelial cells	*CERS2*	0.3581	0.401	0.107	1.778e-50	4.106e-46
7	Blood vascular endothelial cells	*ZHX3*	0.3581	0.340	0.135	9.705e-22	2.241e-17
7	Blood vascular endothelial cells	*PROCR*	0.3580	0.175	0.008	3.702e-144	8.549e-140
7	Blood vascular endothelial cells	*YBX3*	0.3579	0.813	0.578	4.584e-12	1.059e-07
7	Blood vascular endothelial cells	*DCUN1D3*	0.3576	0.235	0.050	2.506e-44	5.787e-40
7	Blood vascular endothelial cells	*MARCHF7*	0.3570	0.608	0.326	2.567e-17	5.927e-13
7	Blood vascular endothelial cells	*EFNB1*	0.3562	0.386	0.134	1.625e-31	3.753e-27
7	Blood vascular endothelial cells	*EEF2K*	0.3561	0.389	0.147	5.577e-27	1.288e-22
7	Blood vascular endothelial cells	*LOC100353245*	0.3561	0.636	0.304	5.683e-25	1.312e-20
7	Blood vascular endothelial cells	*PITPNB*	0.3559	0.654	0.326	1.133e-23	2.616e-19
7	Blood vascular endothelial cells	*LOC138845996*	0.3558	0.268	0.052	5.062e-56	1.169e-51
7	Blood vascular endothelial cells	*MXD4*	0.3551	0.295	0.057	8.042e-62	1.857e-57
7	Blood vascular endothelial cells	*CLEC4G*	0.3551	0.148	0.008	1.329e-103	3.068e-99
7	Blood vascular endothelial cells	*ARHGAP27*	0.3550	0.211	0.009	2.729e-184	6.302e-180
7	Blood vascular endothelial cells	*ROCK2*	0.3544	0.657	0.336	1.413e-21	3.263e-17
7	Blood vascular endothelial cells	*KIAA1671*	0.3542	0.250	0.029	1.381e-93	3.189e-89
7	Blood vascular endothelial cells	*DYNC1H1*	0.3542	0.458	0.163	2.271e-35	5.245e-31
7	Blood vascular endothelial cells	*PPP2R5C*	0.3537	0.422	0.157	4.917e-30	1.136e-25
7	Blood vascular endothelial cells	*ENTREP1*	0.3535	0.187	0.011	2.478e-129	5.722e-125
7	Blood vascular endothelial cells	*TBL1XR1*	0.3533	0.753	0.507	1.776e-12	4.101e-08
7	Blood vascular endothelial cells	*OAS1*	0.3530	0.202	0.004	2.661e-256	6.145e-252
7	Blood vascular endothelial cells	*IRF7*	0.3526	0.256	0.063	1.276e-39	2.946e-35
7	Blood vascular endothelial cells	*FSD1L*	0.3525	0.316	0.105	8.100e-29	1.870e-24
7	Blood vascular endothelial cells	*SDF4*	0.3524	0.539	0.207	3.329e-33	7.686e-29
7	Blood vascular endothelial cells	*FZD4*	0.3524	0.265	0.027	3.871e-114	8.940e-110
7	Blood vascular endothelial cells	*SLITRK4*	0.3524	0.157	0.006	7.489e-146	1.729e-141
7	Blood vascular endothelial cells	*RNF11*	0.3519	0.370	0.119	1.497e-35	3.458e-31

Original cluster	Annotated cell type	Gene	avg_log2FC	pct.1	pct.2	Raw P value	Bonferroni-adjusted P value
7	Blood vascular endothelial cells	*MYO9A*	0.3516	0.590	0.296	3.236e-21	7.472e-17
7	Blood vascular endothelial cells	*LRRC1*	0.3514	0.211	0.033	1.686e-58	3.892e-54
7	Blood vascular endothelial cells	*EXOC4*	0.3512	0.687	0.421	4.697e-14	1.085e-09
7	Blood vascular endothelial cells	*ZNRF3*	0.3503	0.485	0.179	1.686e-33	3.894e-29
7	Blood vascular endothelial cells	*CHMP1B*	0.3500	0.380	0.118	5.783e-38	1.335e-33
7	Blood vascular endothelial cells	*PLAU*	0.3500	0.214	0.016	6.930e-117	1.600e-112
7	Blood vascular endothelial cells	*PRKAG2*	0.3498	0.473	0.198	1.095e-25	2.528e-21
7	Blood vascular endothelial cells	*ATXN2L*	0.3491	0.470	0.164	2.259e-36	5.217e-32
7	Blood vascular endothelial cells	*RSAD2*	0.3490	0.154	0.006	5.104e-135	1.179e-130
7	Blood vascular endothelial cells	*ATP6AP2*	0.3488	0.500	0.196	1.815e-29	4.191e-25
7	Blood vascular endothelial cells	*PPP4C*	0.3487	0.530	0.230	1.808e-25	4.176e-21
7	Blood vascular endothelial cells	*KRAS*	0.3479	0.440	0.151	3.341e-36	7.714e-32
7	Blood vascular endothelial cells	*INPP5K*	0.3478	0.274	0.043	8.130e-74	1.877e-69
7	Blood vascular endothelial cells	*ARPC3*	0.3470	0.675	0.339	5.564e-22	1.285e-17
7	Blood vascular endothelial cells	*MAP1B*	0.3465	0.530	0.257	9.264e-20	2.139e-15
7	Blood vascular endothelial cells	*PAN3*	0.3464	0.633	0.358	9.360e-17	2.161e-12
7	Blood vascular endothelial cells	*MAGOH*	0.3464	0.569	0.248	2.145e-26	4.952e-22
7	Blood vascular endothelial cells	*VAV3*	0.3461	0.377	0.111	2.232e-42	5.155e-38
7	Blood vascular endothelial cells	*PPIA*	0.3460	0.729	0.470	5.989e-14	1.383e-09
7	Blood vascular endothelial cells	*TP53I11*	0.3460	0.220	0.014	2.174e-141	5.020e-137
7	Blood vascular endothelial cells	*CNDP2*	0.3459	0.343	0.079	1.524e-55	3.519e-51
7	Blood vascular endothelial cells	*UBE2E3*	0.3452	0.623	0.322	8.239e-19	1.903e-14
7	Blood vascular endothelial cells	*GIPC2*	0.3450	0.160	0.012	1.469e-93	3.392e-89
7	Blood vascular endothelial cells	*VAMP8*	0.3448	0.446	0.150	2.583e-37	5.964e-33
7	Blood vascular endothelial cells	*WDR1*	0.3444	0.404	0.122	5.757e-42	1.329e-37
7	Blood vascular endothelial cells	*FXYD6*	0.3444	0.554	0.323	2.506e-12	5.787e-08
7	Blood vascular endothelial cells	*LSM1*	0.3443	0.361	0.108	1.147e-38	2.648e-34
7	Blood vascular endothelial cells	*RAB5A*	0.3441	0.440	0.167	1.494e-28	3.449e-24
7	Blood vascular endothelial cells	*LOC138843958*	0.3440	0.373	0.105	2.811e-44	6.491e-40
7	Blood vascular endothelial cells	*SYNCRIP*	0.3433	0.636	0.337	4.198e-19	9.694e-15
7	Blood vascular endothelial cells	*CD59*	0.3431	0.563	0.284	5.874e-19	1.356e-14
7	Blood vascular endothelial cells	*BABAM2*	0.3431	0.578	0.320	4.439e-14	1.025e-09
7	Blood vascular endothelial cells	*AQP3*	0.3425	0.123	0.003	2.653e-147	6.127e-143
7	Blood vascular endothelial cells	*ANKRD28*	0.3423	0.455	0.185	4.847e-27	1.119e-22
7	Blood vascular endothelial cells	*UBE2G1*	0.3423	0.485	0.213	2.047e-22	4.726e-18
7	Blood vascular endothelial cells	*MPDZ*	0.3421	0.503	0.234	5.691e-21	1.314e-16
7	Blood vascular endothelial cells	*GLCE*	0.3420	0.340	0.118	8.118e-29	1.875e-24
7	Blood vascular endothelial cells	*ARPP19*	0.3420	0.542	0.233	2.792e-26	6.448e-22
7	Blood vascular endothelial cells	*ADAMTS4*	0.3419	0.157	0.018	1.978e-59	4.568e-55
7	Blood vascular endothelial cells	*MBD1*	0.3410	0.458	0.163	4.920e-34	1.136e-29

Original cluster	Annotated cell type	Gene	avg_log2FC	pct.1	pct.2	Raw P value	Bonferroni-adjusted P value
7	Blood vascular endothelial cells	*HIPK1*	0.3408	0.500	0.202	1.119e-28	2.584e-24
7	Blood vascular endothelial cells	*PDLIM2*	0.3405	0.325	0.072	4.433e-56	1.024e-51
7	Blood vascular endothelial cells	*TOP2B*	0.3405	0.599	0.322	1.931e-16	4.458e-12
7	Blood vascular endothelial cells	*CAMK1D*	0.3404	0.286	0.063	1.266e-50	2.922e-46
7	Blood vascular endothelial cells	*NDEL1*	0.3399	0.395	0.145	7.152e-29	1.651e-24
7	Blood vascular endothelial cells	*ACTR1A*	0.3399	0.247	0.038	8.070e-70	1.864e-65
7	Blood vascular endothelial cells	*CEBPD*	0.3397	0.864	0.707	8.990e-12	2.076e-07
7	Blood vascular endothelial cells	*SLC25A21*	0.3397	0.373	0.140	3.828e-27	8.839e-23
7	Blood vascular endothelial cells	*CLIC2*	0.3396	0.325	0.102	4.879e-32	1.127e-27
7	Blood vascular endothelial cells	*RAP1GDS1*	0.3393	0.407	0.140	6.931e-33	1.601e-28
7	Blood vascular endothelial cells	*USP34*	0.3392	0.687	0.432	3.182e-15	7.348e-11
7	Blood vascular endothelial cells	*RRAS2*	0.3392	0.319	0.098	1.338e-33	3.089e-29
7	Blood vascular endothelial cells	*TMEM243*	0.3392	0.358	0.104	1.169e-40	2.699e-36
7	Blood vascular endothelial cells	*PCDH19*	0.3391	0.250	0.030	5.562e-91	1.284e-86
7	Blood vascular endothelial cells	*B4GALNT1*	0.3381	0.241	0.018	4.639e-132	1.071e-127
7	Blood vascular endothelial cells	*RYBP*	0.3380	0.630	0.313	1.551e-21	3.582e-17
7	Blood vascular endothelial cells	*EEF1G*	0.3380	0.958	0.930	1.806e-17	4.170e-13
7	Blood vascular endothelial cells	*ZBTB10*	0.3379	0.422	0.170	1.147e-24	2.650e-20
7	Blood vascular endothelial cells	*LOC138846988*	0.3371	0.250	0.039	1.163e-68	2.686e-64
7	Blood vascular endothelial cells	*AMMECR1*	0.3371	0.479	0.211	2.117e-22	4.889e-18
7	Blood vascular endothelial cells	*SACS*	0.3370	0.274	0.048	5.447e-65	1.258e-60
7	Blood vascular endothelial cells	*EPHA4*	0.3365	0.361	0.164	1.171e-15	2.704e-11
7	Blood vascular endothelial cells	*NIT1*	0.3362	0.380	0.100	6.261e-49	1.446e-44
7	Blood vascular endothelial cells	*PLOD1*	0.3358	0.277	0.041	1.010e-79	2.333e-75
7	Blood vascular endothelial cells	*CAPN2*	0.3354	0.431	0.162	2.739e-29	6.324e-25
7	Blood vascular endothelial cells	*EEPD1*	0.3354	0.211	0.016	1.918e-119	4.429e-115
7	Blood vascular endothelial cells	*SUCLG2*	0.3353	0.491	0.191	4.592e-30	1.060e-25
7	Blood vascular endothelial cells	*SSR3*	0.3348	0.801	0.576	5.879e-15	1.358e-10
7	Blood vascular endothelial cells	*FAM219A*	0.3345	0.265	0.047	2.831e-61	6.538e-57
7	Blood vascular endothelial cells	*TEC*	0.3343	0.331	0.146	4.415e-15	1.020e-10
7	Blood vascular endothelial cells	*COX4I1*	0.3337	0.967	0.916	6.137e-18	1.417e-13
7	Blood vascular endothelial cells	*CDC42BPA*	0.3334	0.669	0.412	3.018e-11	6.968e-07
7	Blood vascular endothelial cells	*LOC138846723*	0.3333	0.410	0.111	1.006e-49	2.323e-45
7	Blood vascular endothelial cells	*SBF2*	0.3332	0.666	0.416	4.878e-12	1.127e-07
7	Blood vascular endothelial cells	*GNB1*	0.3327	0.693	0.421	6.324e-14	1.460e-09
7	Blood vascular endothelial cells	*KIF13B*	0.3323	0.289	0.079	8.448e-37	1.951e-32
7	Blood vascular endothelial cells	*ATP1A1*	0.3313	0.627	0.304	1.010e-22	2.331e-18
7	Blood vascular endothelial cells	*TPK1*	0.3311	0.331	0.104	2.995e-33	6.916e-29
7	Blood vascular endothelial cells	*UBA52*	0.3309	0.979	0.980	2.978e-22	6.876e-18
7	Blood vascular endothelial cells	*GNPTAB*	0.3307	0.286	0.077	1.039e-36	2.399e-32

Original cluster	Annotated cell type	Gene	avg_log2FC	pct.1	pct.2	Raw P value	Bonferroni-adjusted P value
7	Blood vascular endothelial cells	*SERPINH1*	0.3304	0.681	0.391	2.023e-15	4.671e-11
7	Blood vascular endothelial cells	*PCF11*	0.3303	0.738	0.517	2.643e-10	6.102e-06
7	Blood vascular endothelial cells	*HMBOX1*	0.3301	0.524	0.297	2.282e-12	5.270e-08
7	Blood vascular endothelial cells	*WARS1*	0.3297	0.295	0.066	4.513e-50	1.042e-45
7	Blood vascular endothelial cells	*DUSP6*	0.3295	0.205	0.022	1.997e-85	4.612e-81
7	Blood vascular endothelial cells	*MKNK2*	0.3291	0.485	0.178	6.601e-34	1.524e-29
7	Blood vascular endothelial cells	*ECH1*	0.3290	0.331	0.080	7.394e-50	1.707e-45
7	Blood vascular endothelial cells	*CPEB3*	0.3289	0.470	0.174	3.408e-32	7.869e-28
7	Blood vascular endothelial cells	*TRIP12*	0.3288	0.485	0.203	3.120e-26	7.205e-22
7	Blood vascular endothelial cells	*MFSD2A*	0.3288	0.136	0.002	5.338e-192	1.233e-187
7	Blood vascular endothelial cells	*RAPGEF6*	0.3286	0.551	0.269	4.072e-20	9.404e-16
7	Blood vascular endothelial cells	*GATAD2B*	0.3286	0.392	0.146	7.392e-28	1.707e-23
7	Blood vascular endothelial cells	*MYO18A*	0.3286	0.235	0.046	8.530e-50	1.970e-45
7	Blood vascular endothelial cells	*CNKSR3*	0.3284	0.316	0.085	1.620e-40	3.740e-36
7	Blood vascular endothelial cells	*LOC108175891*	0.3278	0.398	0.133	9.510e-34	2.196e-29
7	Blood vascular endothelial cells	*APP*	0.3276	0.858	0.644	5.255e-10	1.214e-05
7	Blood vascular endothelial cells	*MAN1A2*	0.3270	0.446	0.173	3.400e-27	7.852e-23
7	Blood vascular endothelial cells	*PDCD10*	0.3270	0.491	0.221	1.423e-22	3.286e-18
7	Blood vascular endothelial cells	*MPHOSPH9*	0.3268	0.199	0.017	1.552e-99	3.583e-95
7	Blood vascular endothelial cells	*SERBP1*	0.3267	0.840	0.617	2.006e-13	4.632e-09
7	Blood vascular endothelial cells	*SETD5*	0.3264	0.645	0.374	8.127e-15	1.877e-10
7	Blood vascular endothelial cells	*STX10*	0.3260	0.587	0.272	8.365e-23	1.932e-18
7	Blood vascular endothelial cells	*MALT1*	0.3260	0.340	0.086	1.852e-48	4.276e-44
7	Blood vascular endothelial cells	*TWF1*	0.3257	0.392	0.116	4.926e-41	1.137e-36
7	Blood vascular endothelial cells	*RGS19*	0.3252	0.259	0.031	3.899e-95	9.003e-91
7	Blood vascular endothelial cells	*IPCEF1*	0.3251	0.301	0.037	2.705e-106	6.247e-102
7	Blood vascular endothelial cells	*PLP2*	0.3251	0.401	0.122	1.042e-40	2.407e-36
7	Blood vascular endothelial cells	*DHRS1*	0.3250	0.545	0.232	2.257e-26	5.212e-22
7	Blood vascular endothelial cells	*KLHL2*	0.3250	0.419	0.136	7.522e-37	1.737e-32
7	Blood vascular endothelial cells	*PPP1R18*	0.3250	0.298	0.054	8.419e-67	1.944e-62
7	Blood vascular endothelial cells	*ADIPOR1*	0.3245	0.355	0.095	4.735e-46	1.093e-41
7	Blood vascular endothelial cells	*TRAM1*	0.3241	0.678	0.362	1.063e-17	2.455e-13
7	Blood vascular endothelial cells	*RPS27L*	0.3236	0.837	0.588	1.675e-13	3.868e-09
7	Blood vascular endothelial cells	*LOC138849203*	0.3236	0.214	0.033	3.023e-59	6.981e-55
7	Blood vascular endothelial cells	*CPNE2*	0.3233	0.280	0.035	7.090e-97	1.637e-92
7	Blood vascular endothelial cells	*SMARCD2*	0.3232	0.295	0.057	6.249e-62	1.443e-57
7	Blood vascular endothelial cells	*RABEP2*	0.3226	0.253	0.043	5.732e-63	1.324e-58
7	Blood vascular endothelial cells	*MIER1*	0.3224	0.699	0.453	3.401e-12	7.853e-08
7	Blood vascular endothelial cells	*SHC1*	0.3220	0.358	0.098	3.148e-44	7.270e-40
7	Blood vascular endothelial cells	*CNIH1*	0.3220	0.383	0.106	2.457e-45	5.674e-41

Original cluster	Annotated cell type	Gene	avg_log2FC	pct.1	pct.2	Raw P value	Bonferroni-adjusted P value
7	Blood vascular endothelial cells	*BLVRB*	0.3218	0.590	0.247	1.871e-28	4.320e-24
7	Blood vascular endothelial cells	*PEA15*	0.3217	0.352	0.116	3.410e-32	7.875e-28
7	Blood vascular endothelial cells	*HTRA1*	0.3216	0.599	0.324	2.849e-15	6.579e-11
7	Blood vascular endothelial cells	*NOP53*	0.3215	0.726	0.415	2.088e-16	4.821e-12
7	Blood vascular endothelial cells	*MAP7D1*	0.3215	0.331	0.082	3.205e-48	7.401e-44
7	Blood vascular endothelial cells	*PPM1G*	0.3215	0.398	0.139	6.425e-31	1.484e-26
7	Blood vascular endothelial cells	*WDR37*	0.3214	0.343	0.111	8.277e-33	1.911e-28
7	Blood vascular endothelial cells	*TNFAIP1*	0.3214	0.235	0.030	3.312e-79	7.649e-75
7	Blood vascular endothelial cells	*VAT1*	0.3212	0.503	0.207	6.954e-26	1.606e-21
7	Blood vascular endothelial cells	*TNFRSF1B*	0.3211	0.214	0.028	6.823e-72	1.576e-67
7	Blood vascular endothelial cells	*RAPH1*	0.3211	0.307	0.106	6.088e-26	1.406e-21
7	Blood vascular endothelial cells	*ZBTB16*	0.3211	0.443	0.211	2.030e-18	4.688e-14
7	Blood vascular endothelial cells	*LOC108177130*	0.3211	0.301	0.100	1.674e-27	3.865e-23
7	Blood vascular endothelial cells	*ASXL2*	0.3210	0.428	0.196	1.206e-19	2.785e-15
7	Blood vascular endothelial cells	*SASH1*	0.3210	0.581	0.277	3.251e-21	7.508e-17
7	Blood vascular endothelial cells	*INPP4B*	0.3208	0.575	0.227	4.022e-36	9.288e-32
7	Blood vascular endothelial cells	*EEF2*	0.3206	0.955	0.881	1.378e-15	3.181e-11
7	Blood vascular endothelial cells	*ARPC2*	0.3204	0.720	0.395	1.270e-21	2.933e-17
7	Blood vascular endothelial cells	*EGR2*	0.3203	0.190	0.021	4.464e-76	1.031e-71
7	Blood vascular endothelial cells	*LCMT1*	0.3201	0.349	0.123	2.368e-27	5.469e-23
7	Blood vascular endothelial cells	*TMF1*	0.3201	0.470	0.197	1.908e-24	4.407e-20
7	Blood vascular endothelial cells	*RSRP1*	0.3201	0.627	0.342	2.947e-16	6.805e-12
7	Blood vascular endothelial cells	*ADA*	0.3195	0.226	0.020	8.829e-112	2.039e-107
7	Blood vascular endothelial cells	*DPF3*	0.3195	0.187	0.024	5.324e-64	1.229e-59
7	Blood vascular endothelial cells	*ENOX2*	0.3190	0.380	0.123	2.541e-35	5.868e-31
7	Blood vascular endothelial cells	*GPI*	0.3185	0.545	0.258	1.537e-20	3.549e-16
7	Blood vascular endothelial cells	*TRIM38*	0.3185	0.280	0.045	3.156e-73	7.288e-69
7	Blood vascular endothelial cells	*ETF1*	0.3182	0.437	0.158	1.415e-31	3.267e-27
7	Blood vascular endothelial cells	*LOC138845290*	0.3181	0.434	0.174	7.741e-25	1.788e-20
7	Blood vascular endothelial cells	*LOC127492178*	0.3181	0.367	0.125	3.661e-31	8.453e-27
7	Blood vascular endothelial cells	*TNFRSF11A*	0.3180	0.196	0.025	2.383e-69	5.503e-65
7	Blood vascular endothelial cells	*WASF2*	0.3173	0.545	0.240	4.477e-25	1.034e-20
7	Blood vascular endothelial cells	*VCP*	0.3172	0.473	0.171	3.925e-33	9.065e-29
7	Blood vascular endothelial cells	*SMAP1*	0.3171	0.545	0.259	2.220e-20	5.126e-16
7	Blood vascular endothelial cells	*CUL3*	0.3170	0.630	0.405	2.281e-09	5.267e-05
7	Blood vascular endothelial cells	*MVB12A*	0.3167	0.458	0.183	3.726e-27	8.604e-23
7	Blood vascular endothelial cells	*CAPZA2*	0.3166	0.795	0.553	2.446e-12	5.648e-08
7	Blood vascular endothelial cells	*PLCG1*	0.3163	0.328	0.069	5.199e-60	1.201e-55
7	Blood vascular endothelial cells	*TDRP*	0.3161	0.205	0.013	7.365e-135	1.701e-130
7	Blood vascular endothelial cells	*ABI3*	0.3161	0.181	0.005	4.293e-185	9.914e-181

Original cluster	Annotated cell type	Gene	avg_log2FC	pct.1	pct.2	Raw P value	Bonferroni-adjusted P value
7	Blood vascular endothelial cells	*MID2*	0.3160	0.319	0.085	2.122e-41	4.901e-37
7	Blood vascular endothelial cells	*ROCK1*	0.3160	0.753	0.416	3.330e-22	7.690e-18
7	Blood vascular endothelial cells	*POLR2I*	0.3155	0.364	0.122	1.923e-31	4.441e-27
7	Blood vascular endothelial cells	*ZFP91*	0.3154	0.563	0.292	2.773e-17	6.403e-13
7	Blood vascular endothelial cells	*ATOSA*	0.3153	0.479	0.287	2.649e-08	6.117e-04
7	Blood vascular endothelial cells	*SEMA7A*	0.3153	0.181	0.003	6.531e-250	1.508e-245
7	Blood vascular endothelial cells	*SP100*	0.3148	0.178	0.022	5.362e-64	1.238e-59
7	Blood vascular endothelial cells	*BRWD3*	0.3145	0.298	0.118	4.519e-19	1.043e-14
7	Blood vascular endothelial cells	*CBFB*	0.3145	0.343	0.108	1.133e-33	2.615e-29
7	Blood vascular endothelial cells	*BCAR1*	0.3143	0.241	0.036	1.770e-68	4.088e-64
7	Blood vascular endothelial cells	*EDNRB*	0.3142	0.190	0.035	4.298e-43	9.926e-39
7	Blood vascular endothelial cells	*KLF9*	0.3134	0.503	0.263	5.359e-16	1.238e-11
7	Blood vascular endothelial cells	*MAP7D3*	0.3132	0.425	0.148	9.431e-33	2.178e-28
7	Blood vascular endothelial cells	*SMYD2*	0.3116	0.274	0.068	9.732e-40	2.247e-35
7	Blood vascular endothelial cells	*GNGT2*	0.3113	0.196	0.005	1.751e-220	4.043e-216
7	Blood vascular endothelial cells	*FER*	0.3110	0.539	0.280	1.582e-16	3.654e-12
7	Blood vascular endothelial cells	*BRWD1*	0.3108	0.509	0.223	2.468e-23	5.699e-19
7	Blood vascular endothelial cells	*SERINC5*	0.3106	0.277	0.061	2.444e-48	5.644e-44
7	Blood vascular endothelial cells	*LOC103348448*	0.3094	0.223	0.040	5.368e-51	1.240e-46
7	Blood vascular endothelial cells	*ADARB1*	0.3088	0.313	0.075	5.004e-48	1.156e-43
7	Blood vascular endothelial cells	*MRPL58*	0.3086	0.340	0.082	6.767e-51	1.563e-46
7	Blood vascular endothelial cells	*ACVR1*	0.3084	0.340	0.109	1.502e-32	3.469e-28
7	Blood vascular endothelial cells	*RAN*	0.3083	0.452	0.168	8.155e-30	1.883e-25
7	Blood vascular endothelial cells	*ETV3*	0.3082	0.352	0.121	6.951e-30	1.605e-25
7	Blood vascular endothelial cells	*LUC7L2*	0.3082	0.599	0.320	1.499e-15	3.462e-11
7	Blood vascular endothelial cells	*LAMA4*	0.3080	0.464	0.198	1.301e-23	3.004e-19
7	Blood vascular endothelial cells	*PRKD3*	0.3080	0.509	0.239	5.554e-20	1.283e-15
7	Blood vascular endothelial cells	*RASSF1*	0.3065	0.410	0.154	7.863e-28	1.816e-23
7	Blood vascular endothelial cells	*LOC100344388*	0.3064	0.596	0.286	3.321e-20	7.668e-16
7	Blood vascular endothelial cells	*AMMECR1L*	0.3064	0.271	0.057	2.729e-51	6.303e-47
7	Blood vascular endothelial cells	*PIK3C2A*	0.3064	0.482	0.206	5.993e-23	1.384e-18
7	Blood vascular endothelial cells	*SMC4*	0.3062	0.352	0.134	2.105e-24	4.861e-20
7	Blood vascular endothelial cells	*PRELID1*	0.3060	0.473	0.203	6.471e-23	1.494e-18
7	Blood vascular endothelial cells	*BTBD1*	0.3057	0.461	0.193	6.975e-24	1.611e-19
7	Blood vascular endothelial cells	*DHRS4*	0.3056	0.247	0.049	8.726e-51	2.015e-46
7	Blood vascular endothelial cells	*RPS11*	0.3056	0.979	0.981	4.951e-11	1.143e-06
7	Blood vascular endothelial cells	*IPMK*	0.3055	0.292	0.067	4.171e-48	9.633e-44
7	Blood vascular endothelial cells	*NDFIP1*	0.3053	0.855	0.648	1.260e-13	2.909e-09
7	Blood vascular endothelial cells	*NGLY1*	0.3051	0.455	0.182	4.067e-25	9.392e-21
7	Blood vascular endothelial cells	*CORO1B*	0.3049	0.419	0.155	4.121e-29	9.515e-25

Original cluster	Annotated cell type	Gene	avg_log2FC	pct.1	pct.2	Raw P value	Bonferroni-adjusted P value
7	Blood vascular endothelial cells	*PTMS*	0.3048	0.822	0.607	1.120e-09	2.586e-05
7	Blood vascular endothelial cells	*TGFB2*	0.3047	0.187	0.088	1.423e-08	3.287e-04
7	Blood vascular endothelial cells	*LOC127484640*	0.3038	0.175	0.001	0	0
7	Blood vascular endothelial cells	*PRRG2*	0.3033	0.247	0.032	5.242e-84	1.211e-79
7	Blood vascular endothelial cells	*ADAM17*	0.3031	0.271	0.051	4.428e-59	1.023e-54
7	Blood vascular endothelial cells	*CCT6A*	0.3030	0.708	0.418	7.685e-15	1.775e-10
7	Blood vascular endothelial cells	*OAZ2*	0.3030	0.569	0.310	2.497e-15	5.766e-11
7	Blood vascular endothelial cells	*KIF1B*	0.3029	0.530	0.242	6.739e-22	1.556e-17
7	Blood vascular endothelial cells	*PNRC1*	0.3023	0.943	0.832	9.312e-15	2.150e-10
7	Blood vascular endothelial cells	*PIEZO1*	0.3019	0.244	0.030	3.186e-87	7.357e-83
7	Blood vascular endothelial cells	*SCAF4*	0.3018	0.343	0.123	8.793e-26	2.030e-21
7	Blood vascular endothelial cells	*ACP1*	0.3017	0.446	0.157	1.934e-33	4.466e-29
7	Blood vascular endothelial cells	*TMCC1*	0.3017	0.340	0.127	2.587e-24	5.974e-20
7	Blood vascular endothelial cells	*ANXA4*	0.3014	0.199	0.044	6.368e-35	1.470e-30
7	Blood vascular endothelial cells	*KDM6A*	0.3013	0.630	0.378	5.351e-12	1.236e-07
7	Blood vascular endothelial cells	*GPD1L*	0.3010	0.226	0.039	2.274e-55	5.252e-51
7	Blood vascular endothelial cells	*MASTL*	0.3010	0.211	0.040	7.409e-46	1.711e-41
7	Blood vascular endothelial cells	*CUL1*	0.3008	0.467	0.198	2.193e-24	5.063e-20
7	Blood vascular endothelial cells	*LOC138849238*	0.3006	0.157	0.006	6.767e-141	1.563e-136
7	Blood vascular endothelial cells	*MANF*	0.3006	0.395	0.135	3.848e-32	8.885e-28
7	Blood vascular endothelial cells	*LOC100343299*	0.3003	0.223	0.128	3.879e-07	8.958e-03
7	Blood vascular endothelial cells	*SMDT1*	0.3003	0.470	0.179	7.426e-30	1.715e-25
7	Blood vascular endothelial cells	*RALA*	0.3000	0.476	0.179	9.641e-30	2.226e-25
7	Blood vascular endothelial cells	*YEATS2*	0.2997	0.283	0.067	3.440e-45	7.944e-41
7	Blood vascular endothelial cells	*PLEKHA3*	0.2997	0.413	0.133	9.266e-37	2.140e-32
7	Blood vascular endothelial cells	*UHMK1*	0.2997	0.452	0.187	2.031e-24	4.690e-20
7	Blood vascular endothelial cells	*SH3GL1*	0.2994	0.416	0.160	3.540e-26	8.174e-22
7	Blood vascular endothelial cells	*PPP2CB*	0.2991	0.497	0.222	2.680e-22	6.188e-18
7	Blood vascular endothelial cells	*MSL1*	0.2983	0.434	0.163	1.148e-28	2.651e-24
7	Blood vascular endothelial cells	*SHB*	0.2983	0.211	0.017	8.433e-110	1.947e-105
7	Blood vascular endothelial cells	*ASB9*	0.2980	0.178	0.004	1.518e-200	3.506e-196
7	Blood vascular endothelial cells	*OGFRL1*	0.2980	0.358	0.127	4.534e-28	1.047e-23
7	Blood vascular endothelial cells	*SRSF4*	0.2979	0.630	0.354	2.845e-15	6.569e-11
7	Blood vascular endothelial cells	*EML3*	0.2977	0.220	0.040	1.532e-49	3.538e-45
7	Blood vascular endothelial cells	*GALNT10*	0.2977	0.247	0.042	7.712e-61	1.781e-56
7	Blood vascular endothelial cells	*ANKRD10*	0.2974	0.482	0.206	8.595e-24	1.985e-19
7	Blood vascular endothelial cells	*LDHA*	0.2972	0.244	0.048	2.027e-50	4.681e-46
7	Blood vascular endothelial cells	*LRP10*	0.2970	0.434	0.149	1.385e-33	3.197e-29
7	Blood vascular endothelial cells	*NAMPT*	0.2970	0.380	0.118	3.144e-37	7.260e-33
7	Blood vascular endothelial cells	*ASH1L*	0.2970	0.723	0.497	1.754e-08	4.050e-04

Original cluster	Annotated cell type	Gene	avg_log2FC	pct.1	pct.2	Raw P value	Bonferroni-adjusted P value
7	Blood vascular endothelial cells	*ZNF207*	0.2966	0.554	0.258	3.692e-21	8.527e-17
7	Blood vascular endothelial cells	*SLC30A7*	0.2960	0.443	0.198	1.699e-19	3.924e-15
7	Blood vascular endothelial cells	*DENND4C*	0.2955	0.467	0.235	3.425e-15	7.908e-11
7	Blood vascular endothelial cells	*TMEM131*	0.2955	0.431	0.179	2.864e-23	6.613e-19
7	Blood vascular endothelial cells	*RGS3*	0.2955	0.512	0.202	3.482e-28	8.040e-24
7	Blood vascular endothelial cells	*CPEB2*	0.2954	0.389	0.119	2.023e-39	4.672e-35
7	Blood vascular endothelial cells	*TRA2B*	0.2953	0.750	0.486	2.330e-11	5.381e-07
7	Blood vascular endothelial cells	*USP31*	0.2953	0.196	0.046	4.124e-31	9.523e-27
7	Blood vascular endothelial cells	*ARF6*	0.2948	0.599	0.288	3.322e-22	7.672e-18
7	Blood vascular endothelial cells	*NAGA*	0.2947	0.238	0.044	3.381e-53	7.807e-49
7	Blood vascular endothelial cells	*MSRB3*	0.2940	0.530	0.240	1.286e-23	2.970e-19
7	Blood vascular endothelial cells	*LOC103345192*	0.2930	0.747	0.444	3.219e-14	7.434e-10
7	Blood vascular endothelial cells	*KLF4*	0.2930	0.913	0.769	2.366e-10	5.463e-06
7	Blood vascular endothelial cells	*NBDY*	0.2929	0.416	0.174	6.608e-22	1.526e-17
7	Blood vascular endothelial cells	*NDUFA4*	0.2929	0.783	0.507	6.952e-13	1.605e-08
7	Blood vascular endothelial cells	*HNRNPH1*	0.2926	0.843	0.591	1.131e-11	2.612e-07
7	Blood vascular endothelial cells	*NDFIP2*	0.2925	0.530	0.247	2.167e-20	5.005e-16
7	Blood vascular endothelial cells	*ING2*	0.2924	0.271	0.077	1.387e-32	3.204e-28
7	Blood vascular endothelial cells	*GK5*	0.2924	0.316	0.094	4.604e-34	1.063e-29
7	Blood vascular endothelial cells	*ST7L*	0.2922	0.358	0.129	2.286e-27	5.279e-23
7	Blood vascular endothelial cells	*BICD1*	0.2921	0.199	0.034	9.803e-49	2.264e-44
7	Blood vascular endothelial cells	*GNAI3*	0.2913	0.605	0.314	3.552e-17	8.201e-13
7	Blood vascular endothelial cells	*CASP6*	0.2902	0.268	0.055	6.487e-52	1.498e-47
7	Blood vascular endothelial cells	*HTT*	0.2901	0.343	0.107	4.525e-34	1.045e-29
7	Blood vascular endothelial cells	*RIPK1*	0.2900	0.247	0.055	3.648e-43	8.423e-39
7	Blood vascular endothelial cells	*NCOA7*	0.2900	0.473	0.263	1.295e-10	2.990e-06
7	Blood vascular endothelial cells	*BRD4*	0.2900	0.596	0.281	3.271e-21	7.553e-17
7	Blood vascular endothelial cells	*PEAR1*	0.2899	0.265	0.038	3.704e-79	8.553e-75
7	Blood vascular endothelial cells	*RAF1*	0.2899	0.434	0.189	5.419e-21	1.251e-16
7	Blood vascular endothelial cells	*NRDC*	0.2899	0.503	0.211	8.650e-26	1.997e-21
7	Blood vascular endothelial cells	*PITPNM2*	0.2892	0.229	0.018	6.906e-121	1.595e-116
7	Blood vascular endothelial cells	*MAN2A1*	0.2891	0.494	0.215	6.585e-22	1.521e-17
7	Blood vascular endothelial cells	*LDLRAD4*	0.2889	0.518	0.275	1.537e-14	3.549e-10
7	Blood vascular endothelial cells	*MANSC1*	0.2886	0.235	0.021	2.783e-113	6.428e-109
7	Blood vascular endothelial cells	*MCAM*	0.2885	0.280	0.063	1.237e-46	2.856e-42
7	Blood vascular endothelial cells	*SMIM14*	0.2883	0.343	0.097	3.729e-40	8.611e-36
7	Blood vascular endothelial cells	*NIBAN2*	0.2878	0.256	0.055	4.403e-47	1.017e-42
7	Blood vascular endothelial cells	*AOPEP*	0.2869	0.660	0.370	6.420e-16	1.483e-11
7	Blood vascular endothelial cells	*BRF1*	0.2866	0.244	0.057	7.884e-40	1.821e-35
7	Blood vascular endothelial cells	*PHC2*	0.2866	0.416	0.150	2.760e-30	6.374e-26

Original cluster	Annotated cell type	Gene	avg_log2FC	pct.1	pct.2	Raw P value	Bonferroni-adjusted P value
7	Blood vascular endothelial cells	*LOC103348460*	0.2864	0.190	0.008	3.884e-160	8.969e-156
7	Blood vascular endothelial cells	*XAF1*	0.2852	0.178	0.023	1.079e-61	2.493e-57
7	Blood vascular endothelial cells	*ALYREF*	0.2848	0.488	0.197	4.243e-26	9.798e-22
7	Blood vascular endothelial cells	*LOC100340280*	0.2846	0.178	0.004	2.765e-198	6.384e-194
7	Blood vascular endothelial cells	*LOC108178768*	0.2846	0.154	0.031	8.358e-31	1.930e-26
7	Blood vascular endothelial cells	*AKT1*	0.2845	0.443	0.157	2.171e-32	5.013e-28
7	Blood vascular endothelial cells	*LOC103348322*	0.2842	0.160	0.002	2.803e-228	6.472e-224
7	Blood vascular endothelial cells	*MARK3*	0.2842	0.413	0.174	8.969e-22	2.071e-17
7	Blood vascular endothelial cells	*LMBR1*	0.2838	0.289	0.096	6.055e-26	1.398e-21
7	Blood vascular endothelial cells	*ALDH9A1*	0.2835	0.352	0.128	5.549e-26	1.281e-21
7	Blood vascular endothelial cells	*AP3M1*	0.2834	0.319	0.084	1.918e-41	4.429e-37
7	Blood vascular endothelial cells	*SLC7A7*	0.2833	0.232	0.034	3.037e-67	7.012e-63
7	Blood vascular endothelial cells	*IFNGR1*	0.2830	0.419	0.158	9.739e-28	2.249e-23
7	Blood vascular endothelial cells	*ZDHHC14*	0.2829	0.383	0.151	1.567e-23	3.618e-19
7	Blood vascular endothelial cells	*RPL4*	0.2827	0.858	0.757	9.264e-12	2.139e-07
7	Blood vascular endothelial cells	*LOC100345591*	0.2827	0.584	0.289	1.311e-17	3.027e-13
7	Blood vascular endothelial cells	*JAK3*	0.2826	0.169	0.013	2.125e-92	4.908e-88
7	Blood vascular endothelial cells	*DGKH*	0.2821	0.530	0.235	2.115e-24	4.884e-20
7	Blood vascular endothelial cells	*SOX4*	0.2818	0.723	0.457	6.722e-13	1.552e-08
7	Blood vascular endothelial cells	*CFLAR*	0.2815	0.328	0.109	1.719e-28	3.971e-24
7	Blood vascular endothelial cells	*SOCS5*	0.2808	0.416	0.165	1.166e-24	2.692e-20
7	Blood vascular endothelial cells	*ANXA3*	0.2806	0.449	0.185	3.825e-23	8.833e-19
7	Blood vascular endothelial cells	*RNF44*	0.2804	0.295	0.072	8.790e-44	2.030e-39
7	Blood vascular endothelial cells	*KCTD20*	0.2799	0.352	0.108	3.289e-35	7.594e-31
7	Blood vascular endothelial cells	*SUPT6H*	0.2794	0.452	0.203	2.447e-20	5.650e-16
7	Blood vascular endothelial cells	*SMAGP*	0.2792	0.187	0.008	6.795e-161	1.569e-156
7	Blood vascular endothelial cells	*RHEB*	0.2791	0.717	0.464	1.177e-11	2.718e-07
7	Blood vascular endothelial cells	*MVB12B*	0.2791	0.286	0.056	2.158e-58	4.984e-54
7	Blood vascular endothelial cells	*ST3GAL1*	0.2789	0.202	0.025	4.464e-70	1.031e-65
7	Blood vascular endothelial cells	*NDUFS8*	0.2787	0.217	0.032	3.420e-63	7.898e-59
7	Blood vascular endothelial cells	*BZW2*	0.2787	0.370	0.149	5.156e-22	1.191e-17
7	Blood vascular endothelial cells	*PTPN1*	0.2786	0.292	0.071	4.905e-44	1.133e-39
7	Blood vascular endothelial cells	*HNRNPAB*	0.2786	0.699	0.412	3.342e-14	7.718e-10
7	Blood vascular endothelial cells	*KDELR2*	0.2784	0.572	0.292	1.956e-16	4.516e-12
7	Blood vascular endothelial cells	*OSMR*	0.2781	0.265	0.093	7.215e-22	1.666e-17
7	Blood vascular endothelial cells	*TTBK2*	0.2781	0.380	0.147	4.141e-24	9.562e-20
7	Blood vascular endothelial cells	*R3HDM1*	0.2780	0.416	0.166	2.412e-24	5.571e-20
7	Blood vascular endothelial cells	*GOLGA4*	0.2779	0.476	0.240	4.014e-15	9.270e-11
7	Blood vascular endothelial cells	*UBE2R2*	0.2776	0.672	0.393	3.991e-13	9.216e-09
7	Blood vascular endothelial cells	*ATXN7*	0.2773	0.473	0.217	2.598e-20	5.999e-16

Original cluster	Annotated cell type	Gene	avg_log2FC	pct.1	pct.2	Raw P value	Bonferroni-adjusted P value
7	Blood vascular endothelial cells	*HSD17B12*	0.2770	0.404	0.179	7.789e-19	1.799e-14
7	Blood vascular endothelial cells	*FRAT2*	0.2766	0.181	0.030	1.476e-46	3.407e-42
7	Blood vascular endothelial cells	*KAT6A*	0.2762	0.428	0.190	2.801e-20	6.468e-16
7	Blood vascular endothelial cells	*NRROS*	0.2761	0.172	0.005	7.158e-181	1.653e-176
7	Blood vascular endothelial cells	*EXOC3L1*	0.2760	0.157	0.002	5.921e-238	1.367e-233
7	Blood vascular endothelial cells	*MINK1*	0.2760	0.226	0.048	1.325e-42	3.059e-38
7	Blood vascular endothelial cells	*TMEM205*	0.2756	0.506	0.224	3.992e-22	9.219e-18
7	Blood vascular endothelial cells	*RFX7*	0.2755	0.446	0.231	3.247e-14	7.499e-10
7	Blood vascular endothelial cells	*TBKBP1*	0.2753	0.193	0.017	2.036e-93	4.702e-89
7	Blood vascular endothelial cells	*FAM174A*	0.2750	0.446	0.183	5.894e-24	1.361e-19
7	Blood vascular endothelial cells	*RGS7BP*	0.2749	0.160	0.021	9.801e-53	2.263e-48
7	Blood vascular endothelial cells	*SYNE3*	0.2745	0.181	0.007	9.474e-167	2.188e-162
7	Blood vascular endothelial cells	*GPBP1*	0.2744	0.807	0.539	8.786e-12	2.029e-07
7	Blood vascular endothelial cells	*RSU1*	0.2743	0.596	0.319	1.313e-14	3.032e-10
7	Blood vascular endothelial cells	*MGAT1*	0.2743	0.289	0.070	3.914e-43	9.039e-39
7	Blood vascular endothelial cells	*PPP2R5B*	0.2743	0.244	0.044	4.548e-56	1.050e-51
7	Blood vascular endothelial cells	*RAB4B*	0.2737	0.250	0.048	4.565e-53	1.054e-48
7	Blood vascular endothelial cells	*TEAD2*	0.2736	0.461	0.185	4.402e-25	1.016e-20
7	Blood vascular endothelial cells	*ZNF800*	0.2734	0.373	0.160	4.626e-19	1.068e-14
7	Blood vascular endothelial cells	*LSM14A*	0.2733	0.557	0.268	1.150e-18	2.656e-14
7	Blood vascular endothelial cells	*PLK3*	0.2733	0.223	0.025	8.020e-86	1.852e-81
7	Blood vascular endothelial cells	*RNF216*	0.2732	0.401	0.145	5.048e-29	1.166e-24
7	Blood vascular endothelial cells	*SRSF9*	0.2730	0.617	0.338	2.336e-13	5.394e-09
7	Blood vascular endothelial cells	*EDC3*	0.2729	0.380	0.133	2.607e-29	6.019e-25
7	Blood vascular endothelial cells	*CUX1*	0.2728	0.428	0.159	1.094e-28	2.527e-24
7	Blood vascular endothelial cells	*ADRM1*	0.2719	0.392	0.135	3.454e-31	7.977e-27
7	Blood vascular endothelial cells	*HERC4*	0.2716	0.503	0.252	2.833e-16	6.543e-12
7	Blood vascular endothelial cells	*TSGA10*	0.2716	0.283	0.091	2.221e-27	5.130e-23
7	Blood vascular endothelial cells	*APCDD1*	0.2715	0.648	0.375	2.614e-13	6.037e-09
7	Blood vascular endothelial cells	*BACE2*	0.2715	0.349	0.117	6.703e-30	1.548e-25
7	Blood vascular endothelial cells	*AKT3*	0.2714	0.768	0.543	2.791e-08	6.444e-04
7	Blood vascular endothelial cells	*RAB33B*	0.2711	0.277	0.060	1.340e-49	3.094e-45
7	Blood vascular endothelial cells	*MAFF*	0.2709	0.316	0.078	1.609e-45	3.716e-41
7	Blood vascular endothelial cells	*LOC127484203*	0.2709	0.163	0.041	1.740e-24	4.017e-20
7	Blood vascular endothelial cells	*BDP1*	0.2709	0.473	0.239	3.327e-15	7.683e-11
7	Blood vascular endothelial cells	*B3GNT2*	0.2703	0.286	0.069	9.716e-44	2.244e-39
7	Blood vascular endothelial cells	*FLOT1*	0.2703	0.361	0.118	2.901e-32	6.700e-28
7	Blood vascular endothelial cells	*ZBTB46*	0.2702	0.184	0.019	5.448e-78	1.258e-73
7	Blood vascular endothelial cells	*KPNA4*	0.2701	0.509	0.239	2.063e-19	4.764e-15
7	Blood vascular endothelial cells	*PPP4R3A*	0.2699	0.560	0.258	8.806e-22	2.033e-17

Original cluster	Annotated cell type	Gene	avg_log2FC	pct.1	pct.2	Raw P value	Bonferroni-adjusted P value
7	Blood vascular endothelial cells	*ZNF462*	0.2697	0.295	0.107	1.162e-22	2.683e-18
7	Blood vascular endothelial cells	*SMAD5*	0.2697	0.506	0.233	9.159e-20	2.115e-15
7	Blood vascular endothelial cells	*LOC127491404*	0.2696	0.169	0.005	7.244e-178	1.673e-173
7	Blood vascular endothelial cells	*WDR83OS*	0.2691	0.726	0.440	3.471e-13	8.015e-09
7	Blood vascular endothelial cells	*NCK1*	0.2683	0.630	0.339	1.946e-15	4.494e-11
7	Blood vascular endothelial cells	*TSPAN32*	0.2681	0.157	0.006	8.330e-147	1.924e-142
7	Blood vascular endothelial cells	*ME2*	0.2680	0.262	0.072	1.371e-32	3.167e-28
7	Blood vascular endothelial cells	*TCERG1*	0.2680	0.449	0.200	5.394e-20	1.246e-15
7	Blood vascular endothelial cells	*CDC42EP4*	0.2679	0.398	0.138	1.145e-30	2.643e-26
7	Blood vascular endothelial cells	*SCN3B*	0.2671	0.181	0.009	2.501e-138	5.776e-134
7	Blood vascular endothelial cells	*EIF3D*	0.2670	0.443	0.172	2.893e-26	6.680e-22
7	Blood vascular endothelial cells	*CHD4*	0.2670	0.530	0.263	2.446e-16	5.648e-12
7	Blood vascular endothelial cells	*LOC138847706*	0.2669	0.464	0.195	7.719e-23	1.782e-18
7	Blood vascular endothelial cells	*ARIH2*	0.2666	0.383	0.139	4.584e-28	1.059e-23
7	Blood vascular endothelial cells	*ESYT2*	0.2665	0.464	0.228	4.144e-16	9.569e-12
7	Blood vascular endothelial cells	*UBTF*	0.2665	0.395	0.173	5.698e-19	1.316e-14
7	Blood vascular endothelial cells	*ELOVL5*	0.2661	0.355	0.128	4.319e-26	9.974e-22
7	Blood vascular endothelial cells	*NECAP2*	0.2659	0.380	0.125	1.708e-32	3.945e-28
7	Blood vascular endothelial cells	*NREP*	0.2657	0.295	0.072	3.697e-44	8.537e-40
7	Blood vascular endothelial cells	*ASB3*	0.2652	0.298	0.153	6.254e-10	1.444e-05
7	Blood vascular endothelial cells	*SESTD1*	0.2651	0.395	0.167	3.427e-20	7.914e-16
7	Blood vascular endothelial cells	*MEGF11*	0.2649	0.196	0.065	1.497e-18	3.458e-14
7	Blood vascular endothelial cells	*TMEM273*	0.2648	0.184	0.013	6.708e-107	1.549e-102
7	Blood vascular endothelial cells	*E2F5*	0.2648	0.319	0.112	9.700e-26	2.240e-21
7	Blood vascular endothelial cells	*LOC138849590*	0.2647	0.551	0.283	2.671e-17	6.169e-13
7	Blood vascular endothelial cells	*EFCAB14*	0.2640	0.470	0.207	2.543e-21	5.873e-17
7	Blood vascular endothelial cells	*STRAP*	0.2640	0.494	0.245	1.854e-16	4.281e-12
7	Blood vascular endothelial cells	*CGAS*	0.2639	0.151	0.003	1.149e-190	2.653e-186
7	Blood vascular endothelial cells	*PTPN9*	0.2637	0.364	0.128	1.048e-28	2.420e-24
7	Blood vascular endothelial cells	*LOC127491593*	0.2637	0.139	0.001	1.471e-243	3.396e-239
7	Blood vascular endothelial cells	*CCT7*	0.2634	0.461	0.203	1.081e-19	2.495e-15
7	Blood vascular endothelial cells	*THOC2*	0.2632	0.563	0.286	6.065e-17	1.401e-12
7	Blood vascular endothelial cells	*TRIM21*	0.2628	0.190	0.027	3.099e-59	7.156e-55
7	Blood vascular endothelial cells	*HMOX2*	0.2625	0.319	0.087	2.909e-39	6.717e-35
7	Blood vascular endothelial cells	*FUT4*	0.2621	0.169	0.018	2.475e-68	5.715e-64
7	Blood vascular endothelial cells	*KCTD10*	0.2618	0.292	0.072	2.316e-42	5.348e-38
7	Blood vascular endothelial cells	*NET1*	0.2618	0.431	0.215	8.548e-15	1.974e-10
7	Blood vascular endothelial cells	*LOC103350201*	0.2617	0.175	0.016	3.313e-82	7.650e-78
7	Blood vascular endothelial cells	*XBP1*	0.2617	0.476	0.265	7.818e-12	1.805e-07
7	Blood vascular endothelial cells	*EXOC2*	0.2615	0.343	0.117	8.262e-29	1.908e-24

Original cluster	Annotated cell type	Gene	avg_log2FC	pct.1	pct.2	Raw P value	Bonferroni-adjusted P value
7	Blood vascular endothelial cells	*NCK2*	0.2615	0.367	0.130	5.618e-28	1.297e-23
7	Blood vascular endothelial cells	*ZC3H11A*	0.2613	0.554	0.306	1.232e-13	2.844e-09
7	Blood vascular endothelial cells	*STOX2*	0.2613	0.313	0.124	7.438e-20	1.718e-15
7	Blood vascular endothelial cells	*DDX5*	0.2610	0.955	0.894	1.840e-08	4.248e-04
7	Blood vascular endothelial cells	*KMT5B*	0.2610	0.515	0.252	2.465e-16	5.692e-12
7	Blood vascular endothelial cells	*NCBP1*	0.2609	0.208	0.045	1.097e-38	2.532e-34
7	Blood vascular endothelial cells	*STX3*	0.2599	0.175	0.010	5.201e-122	1.201e-117
7	Blood vascular endothelial cells	*AP3B1*	0.2598	0.380	0.146	5.881e-24	1.358e-19
7	Blood vascular endothelial cells	*DGKZ*	0.2595	0.199	0.023	9.686e-77	2.237e-72
7	Blood vascular endothelial cells	*RREB1*	0.2594	0.244	0.066	3.532e-32	8.157e-28
7	Blood vascular endothelial cells	*RTN3*	0.2593	0.542	0.274	2.244e-16	5.183e-12
7	Blood vascular endothelial cells	*EP400*	0.2591	0.328	0.114	6.370e-27	1.471e-22
7	Blood vascular endothelial cells	*LOC138845370*	0.2588	0.154	0.032	5.349e-30	1.235e-25
7	Blood vascular endothelial cells	*ATAD2B*	0.2586	0.581	0.331	6.596e-13	1.523e-08
7	Blood vascular endothelial cells	*ZDHHC5*	0.2585	0.280	0.072	2.947e-39	6.806e-35
7	Blood vascular endothelial cells	*LOC103352476*	0.2583	0.244	0.069	7.115e-30	1.643e-25
7	Blood vascular endothelial cells	*ATRX*	0.2581	0.801	0.582	8.282e-10	1.912e-05
7	Blood vascular endothelial cells	*CLTA*	0.2580	0.837	0.630	2.051e-09	4.737e-05
7	Blood vascular endothelial cells	*HSPA12B*	0.2574	0.280	0.068	1.534e-41	3.542e-37
7	Blood vascular endothelial cells	*RSF1*	0.2574	0.705	0.458	8.658e-10	1.999e-05
7	Blood vascular endothelial cells	*ARIH1*	0.2570	0.527	0.264	4.981e-16	1.150e-11
7	Blood vascular endothelial cells	*TLE4*	0.2568	0.608	0.369	2.126e-10	4.909e-06
7	Blood vascular endothelial cells	*BTG2*	0.2567	0.943	0.884	2.752e-13	6.354e-09
7	Blood vascular endothelial cells	*MSRA*	0.2567	0.337	0.109	1.898e-31	4.383e-27
7	Blood vascular endothelial cells	*HDLBP*	0.2567	0.497	0.217	1.269e-22	2.930e-18
7	Blood vascular endothelial cells	*COMMD6*	0.2566	0.422	0.169	3.551e-24	8.200e-20
7	Blood vascular endothelial cells	*MSI2*	0.2563	0.485	0.223	4.212e-19	9.725e-15
7	Blood vascular endothelial cells	*HPRT1*	0.2561	0.283	0.055	1.495e-57	3.453e-53
7	Blood vascular endothelial cells	*STX12*	0.2560	0.386	0.136	5.030e-29	1.161e-24
7	Blood vascular endothelial cells	*UBE2L3*	0.2559	0.675	0.398	1.449e-11	3.346e-07
7	Blood vascular endothelial cells	*COLGALT2*	0.2558	0.208	0.026	7.458e-73	1.722e-68
7	Blood vascular endothelial cells	*GPAT4*	0.2556	0.328	0.106	1.169e-30	2.699e-26
7	Blood vascular endothelial cells	*SCAF11*	0.2556	0.581	0.330	2.475e-12	5.716e-08
7	Blood vascular endothelial cells	*EIF4EBP2*	0.2555	0.756	0.540	7.692e-09	1.776e-04
7	Blood vascular endothelial cells	*RABEP1*	0.2552	0.361	0.150	2.457e-18	5.674e-14
7	Blood vascular endothelial cells	*BRD9*	0.2551	0.253	0.054	2.339e-46	5.400e-42
7	Blood vascular endothelial cells	*G6PC3*	0.2550	0.226	0.051	1.543e-38	3.564e-34
7	Blood vascular endothelial cells	*EIF4G1*	0.2550	0.313	0.082	1.929e-41	4.455e-37
7	Blood vascular endothelial cells	*GALNT1*	0.2545	0.542	0.284	9.585e-15	2.213e-10
7	Blood vascular endothelial cells	*RUBCNL*	0.2541	0.166	0.007	5.173e-137	1.195e-132

Original cluster	Annotated cell type	Gene	avg_log2FC	pct.1	pct.2	Raw P value	Bonferroni-adjusted P value
7	Blood vascular endothelial cells	*EIF4G3*	0.2539	0.524	0.295	6.707e-13	1.549e-08
7	Blood vascular endothelial cells	*TMEM163*	0.2537	0.148	0.009	7.497e-95	1.731e-90
7	Blood vascular endothelial cells	*SYNJ2BP*	0.2534	0.389	0.181	8.266e-15	1.909e-10
7	Blood vascular endothelial cells	*PIEZO2*	0.2534	0.196	0.031	1.087e-53	2.510e-49
7	Blood vascular endothelial cells	*BIRC3*	0.2533	0.133	0.022	1.828e-34	4.221e-30
7	Blood vascular endothelial cells	*SLC48A1*	0.2532	0.289	0.094	9.087e-27	2.098e-22
7	Blood vascular endothelial cells	*CCT4*	0.2531	0.578	0.318	1.315e-12	3.038e-08
7	Blood vascular endothelial cells	*CCDC102A*	0.2528	0.298	0.099	1.868e-26	4.314e-22
7	Blood vascular endothelial cells	*LOC138849963*	0.2527	0.136	0.015	9.772e-53	2.257e-48
7	Blood vascular endothelial cells	*C1QTNF5*	0.2526	0.289	0.096	5.647e-26	1.304e-21
7	Blood vascular endothelial cells	*UBE2H*	0.2522	0.557	0.307	6.875e-13	1.588e-08
7	Blood vascular endothelial cells	*SNIP1*	0.2521	0.259	0.066	1.339e-36	3.093e-32
7	Blood vascular endothelial cells	*RALBP1*	0.2514	0.554	0.261	3.290e-19	7.598e-15
7	Blood vascular endothelial cells	*LOC100343047*	0.2511	0.283	0.081	1.790e-32	4.133e-28
7	Blood vascular endothelial cells	*TMCC3*	0.2505	0.202	0.031	4.221e-56	9.748e-52
7	Blood vascular endothelial cells	*SOD2*	0.2503	0.325	0.106	2.194e-29	5.067e-25
7	Blood vascular endothelial cells	*CFAP20DC*	0.2501	0.316	0.132	7.011e-18	1.619e-13
7	Blood vascular endothelial cells	*RHPN2*	0.2501	0.148	0.009	7.111e-97	1.642e-92
8	**Myofibroblasts**	*BNC2*	2.9327	0.949	0.325	2.232e-180	5.155e-176
8	Myofibroblasts	*PCDH7*	2.8782	0.949	0.369	1.205e-163	2.782e-159
8	Myofibroblasts	*MYH11*	2.6456	0.839	0.075	0	0
8	Myofibroblasts	*CACNA1C*	2.1476	0.659	0.186	3.022e-101	6.979e-97
8	Myofibroblasts	*C1H11orf96*	2.0720	0.799	0.273	2.056e-102	4.748e-98
8	Myofibroblasts	*RXFP1*	1.9646	0.864	0.377	1.758e-98	4.060e-94
8	Myofibroblasts	*LOC138848040*	1.8733	0.945	0.899	1.291e-28	2.982e-24
8	Myofibroblasts	*TENM3*	1.8444	0.769	0.394	7.672e-66	1.772e-61
8	Myofibroblasts	*LPP*	1.8296	0.773	0.368	2.689e-77	6.209e-73
8	Myofibroblasts	*RSPO3*	1.7958	0.637	0.157	7.581e-110	1.751e-105
8	Myofibroblasts	*PRUNE2*	1.7869	0.436	0.009	0	0
8	Myofibroblasts	*SLIT2*	1.7574	0.689	0.238	9.166e-84	2.117e-79
8	Myofibroblasts	*FHL1*	1.7432	0.571	0.138	1.774e-103	4.097e-99
8	Myofibroblasts	*PIP5K1B*	1.7419	0.502	0.103	3.354e-102	7.746e-98
8	Myofibroblasts	*TPM2*	1.7356	0.828	0.471	1.192e-75	2.752e-71
8	Myofibroblasts	*MEIS1*	1.7284	0.769	0.317	5.671e-87	1.309e-82
8	Myofibroblasts	*KCNMA1*	1.7269	0.520	0.115	8.136e-101	1.879e-96
8	Myofibroblasts	*PGM5*	1.6231	0.473	0.049	1.200e-188	2.772e-184
8	Myofibroblasts	*RYR3*	1.5654	0.527	0.083	1.598e-139	3.690e-135
8	Myofibroblasts	*TPM1*	1.5493	0.810	0.382	3.422e-83	7.901e-79
8	Myofibroblasts	*PDE5A*	1.5493	0.465	0.300	3.528e-16	8.147e-12
8	Myofibroblasts	*CACNB2*	1.5418	0.747	0.341	6.178e-66	1.427e-61

Original cluster	Annotated cell type	Gene	avg_log2FC	pct.1	pct.2	Raw P value	Bonferroni-adjusted P value
8	Myofibroblasts	*MTSS1*	1.5414	0.659	0.247	2.952e-74	6.816e-70
8	Myofibroblasts	*TAGLN*	1.5191	0.711	0.130	3.178e-154	7.339e-150
8	Myofibroblasts	*IMMP2L*	1.5149	0.788	0.462	1.725e-48	3.983e-44
8	Myofibroblasts	*DMD*	1.4751	0.568	0.174	6.074e-71	1.403e-66
8	Myofibroblasts	*PRR16*	1.4743	0.729	0.421	2.958e-45	6.831e-41
8	Myofibroblasts	*ACTG2*	1.4623	0.414	0.028	3.471e-235	8.016e-231
8	Myofibroblasts	*TRPS1*	1.4575	0.652	0.298	8.158e-51	1.884e-46
8	Myofibroblasts	*PDLIM3*	1.4571	0.487	0.114	5.307e-81	1.225e-76
8	Myofibroblasts	*PDZRN3*	1.4417	0.777	0.471	7.158e-44	1.653e-39
8	Myofibroblasts	*CAP2*	1.4268	0.495	0.120	4.465e-81	1.031e-76
8	Myofibroblasts	*FAT3*	1.4237	0.520	0.141	5.531e-74	1.277e-69
8	Myofibroblasts	*KIAA0408*	1.3980	0.399	0.065	1.442e-102	3.330e-98
8	Myofibroblasts	*HDAC9*	1.3801	0.641	0.319	8.508e-43	1.965e-38
8	Myofibroblasts	*GRM8*	1.3612	0.242	0.057	1.492e-37	3.446e-33
8	Myofibroblasts	*SNX19*	1.3335	0.344	0.029	4.871e-160	1.125e-155
8	Myofibroblasts	*CDON*	1.3308	0.513	0.138	3.240e-73	7.483e-69
8	Myofibroblasts	*MAP3K20*	1.2917	0.652	0.339	2.701e-44	6.238e-40
8	Myofibroblasts	*FILIP1L*	1.2906	0.546	0.233	1.127e-43	2.603e-39
8	Myofibroblasts	*LIMS1*	1.2663	0.623	0.295	2.555e-44	5.899e-40
8	Myofibroblasts	*CELF1*	1.2518	0.615	0.348	1.844e-32	4.259e-28
8	Myofibroblasts	*DCLK1*	1.2411	0.597	0.252	6.205e-46	1.433e-41
8	Myofibroblasts	*LEPR*	1.2387	0.370	0.063	3.377e-84	7.798e-80
8	Myofibroblasts	*NELL1*	1.2378	0.297	0.116	4.362e-22	1.007e-17
8	Myofibroblasts	*FREM2*	1.2150	0.308	0.059	9.839e-63	2.272e-58
8	Myofibroblasts	*PARD3B*	1.2041	0.788	0.569	1.208e-37	2.789e-33
8	Myofibroblasts	*FRMD4B*	1.2013	0.520	0.204	3.627e-43	8.374e-39
8	Myofibroblasts	*MYLK*	1.1910	0.399	0.071	2.995e-90	6.915e-86
8	Myofibroblasts	*CNN1*	1.1814	0.319	0.020	5.320e-192	1.229e-187
8	Myofibroblasts	*RNF150*	1.1774	0.355	0.089	9.637e-52	2.225e-47
8	Myofibroblasts	*PLCB1*	1.1748	0.648	0.391	1.624e-27	3.749e-23
8	Myofibroblasts	*PRKD1*	1.1698	0.586	0.316	2.990e-28	6.905e-24
8	Myofibroblasts	*MYL9*	1.1661	0.571	0.214	5.481e-55	1.266e-50
8	Myofibroblasts	*CAV1*	1.1562	0.663	0.239	2.215e-63	5.115e-59
8	Myofibroblasts	*SLC24A3*	1.1475	0.777	0.467	3.584e-38	8.275e-34
8	Myofibroblasts	*LOC100353865*	1.1469	0.505	0.171	4.950e-54	1.143e-49
8	Myofibroblasts	*TES*	1.1422	0.385	0.083	2.770e-70	6.396e-66
8	Myofibroblasts	*ACTA2*	1.1248	0.429	0.075	6.627e-90	1.530e-85
8	Myofibroblasts	*LRRC4C*	1.1236	0.249	0.104	1.153e-15	2.662e-11
8	Myofibroblasts	*TTC28*	1.1209	0.755	0.580	2.233e-28	5.157e-24
8	Myofibroblasts	*LOC103347707*	1.1155	0.275	0.005	0	0

Original cluster	Annotated cell type	Gene	avg_log2FC	pct.1	pct.2	Raw P value	Bonferroni-adjusted P value
8	Myofibroblasts	*LOC127484872*	1.1129	0.773	0.650	3.882e-19	8.964e-15
8	Myofibroblasts	*RBPMS*	1.1105	0.755	0.478	2.198e-39	5.075e-35
8	Myofibroblasts	*FBXO32*	1.1020	0.480	0.223	1.507e-31	3.480e-27
8	Myofibroblasts	*SGCD*	1.1015	0.473	0.248	1.211e-23	2.796e-19
8	Myofibroblasts	*RALGPS2*	1.1005	0.656	0.378	2.570e-36	5.934e-32
8	Myofibroblasts	*ANK3*	1.0589	0.505	0.245	4.369e-29	1.009e-24
8	Myofibroblasts	*DGKI*	1.0515	0.278	0.027	1.914e-115	4.420e-111
8	Myofibroblasts	*LOC138848811*	1.0494	0.286	0.076	2.479e-37	5.724e-33
8	Myofibroblasts	*ARHGAP20*	1.0406	0.469	0.225	2.158e-26	4.984e-22
8	Myofibroblasts	*RHOBTB1*	1.0387	0.429	0.213	5.899e-23	1.362e-18
8	Myofibroblasts	*MTCL3*	1.0284	0.363	0.065	2.016e-79	4.655e-75
8	Myofibroblasts	*SPARCL1*	1.0225	0.868	0.782	2.554e-36	5.897e-32
8	Myofibroblasts	*DDIT4L*	1.0196	0.414	0.146	9.298e-37	2.147e-32
8	Myofibroblasts	*RBM24*	1.0150	0.363	0.083	1.499e-60	3.463e-56
8	Myofibroblasts	*LOC138844924*	1.0149	0.264	0.025	7.754e-112	1.791e-107
8	Myofibroblasts	*TRPC4*	0.9915	0.363	0.193	3.037e-15	7.014e-11
8	Myofibroblasts	*EPB41L2*	0.9770	0.590	0.320	2.782e-29	6.425e-25
8	Myofibroblasts	*ANO4*	0.9681	0.634	0.379	2.976e-21	6.871e-17
8	Myofibroblasts	*ADRA1A*	0.9672	0.264	0.055	4.436e-47	1.024e-42
8	Myofibroblasts	*CDH2*	0.9597	0.256	0.020	3.215e-125	7.425e-121
8	Myofibroblasts	*CACNA2D1*	0.9585	0.495	0.322	1.496e-15	3.454e-11
8	Myofibroblasts	*TRPC6*	0.9549	0.234	0.041	1.780e-52	4.110e-48
8	Myofibroblasts	*PBX1*	0.9535	0.934	0.811	3.450e-28	7.966e-24
8	Myofibroblasts	*CELF2*	0.9524	0.828	0.593	4.578e-29	1.057e-24
8	Myofibroblasts	*ITPR1*	0.9273	0.304	0.107	3.333e-26	7.697e-22
8	Myofibroblasts	*CEMIP*	0.9154	0.223	0.019	7.983e-100	1.843e-95
8	Myofibroblasts	*DLGAP1*	0.9105	0.278	0.108	4.583e-20	1.058e-15
8	Myofibroblasts	*JUN*	0.9065	0.835	0.856	3.386e-17	7.820e-13
8	Myofibroblasts	*MEIS2*	0.9032	0.868	0.724	1.524e-28	3.520e-24
8	Myofibroblasts	*LOC138850424*	0.8997	0.802	0.801	2.727e-17	6.298e-13
8	Myofibroblasts	*PPP3CA*	0.8948	0.634	0.429	3.740e-20	8.636e-16
8	Myofibroblasts	*NRP2*	0.8947	0.344	0.088	6.002e-48	1.386e-43
8	Myofibroblasts	*MYL6*	0.8890	0.835	0.730	3.966e-35	9.158e-31
8	Myofibroblasts	*PCP4*	0.8855	0.231	0.011	3.549e-161	8.196e-157
8	Myofibroblasts	*PBX3*	0.8806	0.370	0.217	2.152e-12	4.970e-08
8	Myofibroblasts	*PARM1*	0.8799	0.403	0.176	7.569e-24	1.748e-19
8	Myofibroblasts	*LOC103349250*	0.8776	0.377	0.194	2.297e-17	5.304e-13
8	Myofibroblasts	*NEDD4L*	0.8738	0.352	0.121	3.552e-32	8.203e-28
8	Myofibroblasts	*RUNX1T1*	0.8705	0.729	0.569	3.361e-22	7.762e-18
8	Myofibroblasts	*FGF7*	0.8690	0.249	0.041	5.873e-59	1.356e-54

Original cluster	Annotated cell type	Gene	avg_log2FC	pct.1	pct.2	Raw P value	Bonferroni-adjusted P value
8	Myofibroblasts	*LOC138845366*	0.8662	0.337	0.172	2.728e-14	6.299e-10
8	Myofibroblasts	*TMEM200A*	0.8655	0.227	0.078	5.603e-20	1.294e-15
8	Myofibroblasts	*DIP2C*	0.8620	0.527	0.396	8.265e-11	1.908e-06
8	Myofibroblasts	*AOPEP*	0.8539	0.553	0.375	1.653e-14	3.817e-10
8	Myofibroblasts	*ARID5B*	0.8523	0.681	0.471	9.480e-20	2.189e-15
8	Myofibroblasts	*MBOAT1*	0.8519	0.256	0.065	2.006e-35	4.632e-31
8	Myofibroblasts	*DENND2B*	0.8508	0.370	0.171	4.744e-20	1.096e-15
8	Myofibroblasts	*ACTN1*	0.8490	0.421	0.230	1.395e-16	3.222e-12
8	Myofibroblasts	*NAV2*	0.8484	0.300	0.084	4.310e-37	9.952e-33
8	Myofibroblasts	*PALM2AKAP2*	0.8440	0.377	0.131	3.288e-32	7.593e-28
8	Myofibroblasts	*OGT*	0.8431	0.535	0.355	2.729e-16	6.301e-12
8	Myofibroblasts	*SMYD3*	0.8412	0.575	0.421	3.486e-12	8.049e-08
8	Myofibroblasts	*FBXL7*	0.8404	0.593	0.479	1.511e-10	3.490e-06
8	Myofibroblasts	*CPED1*	0.8353	0.564	0.308	1.960e-23	4.526e-19
8	Myofibroblasts	*EDIL3*	0.8347	0.223	0.013	3.245e-140	7.493e-136
8	Myofibroblasts	*TMEM200B*	0.8319	0.344	0.076	9.524e-57	2.199e-52
8	Myofibroblasts	*ZFPM2*	0.8315	0.414	0.194	3.602e-20	8.317e-16
8	Myofibroblasts	*SORBS1*	0.8276	0.264	0.069	1.951e-35	4.505e-31
8	Myofibroblasts	*LHFPL6*	0.8273	0.674	0.500	2.052e-15	4.739e-11
8	Myofibroblasts	*HIBADH*	0.8249	0.538	0.381	8.317e-14	1.921e-09
8	Myofibroblasts	*ESR1*	0.8203	0.861	0.781	5.560e-15	1.284e-10
8	Myofibroblasts	*MAP3K13*	0.8200	0.381	0.240	1.040e-10	2.402e-06
8	Myofibroblasts	*RGS2*	0.8177	0.355	0.220	9.509e-10	2.196e-05
8	Myofibroblasts	*TGFB1I1*	0.8154	0.458	0.222	7.134e-25	1.647e-20
8	Myofibroblasts	*MSRB3*	0.8110	0.407	0.246	3.269e-13	7.548e-09
8	Myofibroblasts	*RRAD*	0.8091	0.374	0.128	2.379e-31	5.493e-27
8	Myofibroblasts	*SSBP2*	0.8062	0.637	0.447	5.162e-19	1.192e-14
8	Myofibroblasts	*PALLD*	0.7968	0.520	0.369	1.839e-12	4.247e-08
8	Myofibroblasts	*TMEM135*	0.7946	0.520	0.392	1.512e-10	3.492e-06
8	Myofibroblasts	*MPP7*	0.7936	0.264	0.074	8.587e-32	1.983e-27
8	Myofibroblasts	*MYOCD*	0.7909	0.304	0.051	1.967e-67	4.542e-63
8	Myofibroblasts	*WFDC1*	0.7857	0.223	0.031	6.085e-64	1.405e-59
8	Myofibroblasts	*CSRP1*	0.7839	0.575	0.338	1.546e-24	3.571e-20
8	Myofibroblasts	*RBFOX2*	0.7806	0.700	0.518	6.446e-19	1.489e-14
8	Myofibroblasts	*SLC40A1*	0.7784	0.524	0.400	6.434e-11	1.486e-06
8	Myofibroblasts	*DSTN*	0.7760	0.714	0.563	2.302e-20	5.315e-16
8	Myofibroblasts	*VPS13B*	0.7755	0.689	0.579	2.984e-13	6.890e-09
8	Myofibroblasts	*IGFBP5*	0.7744	0.930	0.622	1.403e-50	3.241e-46
8	Myofibroblasts	*ANTXR2*	0.7706	0.674	0.466	6.515e-21	1.504e-16
8	Myofibroblasts	*SMTN*	0.7598	0.311	0.108	1.376e-27	3.179e-23

Original cluster	Annotated cell type	Gene	avg_log2FC	pct.1	pct.2	Raw P value	Bonferroni-adjusted P value
8	Myofibroblasts	*SLC8A1*	0.7524	0.505	0.400	9.094e-07	2.100e-02
8	Myofibroblasts	*CAMKMT*	0.7515	0.473	0.355	1.189e-07	2.746e-03
8	Myofibroblasts	*PTGFR*	0.7510	0.242	0.048	2.007e-45	4.634e-41
8	Myofibroblasts	*CEP120*	0.7500	0.271	0.131	2.181e-13	5.037e-09
8	Myofibroblasts	*GAB1*	0.7440	0.410	0.252	1.314e-11	3.033e-07
8	Myofibroblasts	*ARID1B*	0.7438	0.601	0.507	1.257e-08	2.902e-04
8	Myofibroblasts	*FGFR2*	0.7430	0.300	0.172	4.522e-09	1.044e-04
8	Myofibroblasts	*SCMH1*	0.7408	0.520	0.369	2.967e-11	6.850e-07
8	Myofibroblasts	*CKB*	0.7395	0.308	0.129	7.706e-20	1.779e-15
8	Myofibroblasts	*AMMECR1*	0.7378	0.363	0.217	1.649e-11	3.809e-07
8	Myofibroblasts	*LOC138847632*	0.7365	0.165	0.004	2.444e-176	5.643e-172
8	Myofibroblasts	*COL17A1*	0.7348	0.212	0.025	3.799e-69	8.773e-65
8	Myofibroblasts	*RAD51B*	0.7333	0.311	0.205	4.898e-07	1.131e-02
8	Myofibroblasts	*GRIA3*	0.7319	0.165	0.030	8.034e-34	1.855e-29
8	Myofibroblasts	*FILIP1*	0.7305	0.256	0.074	2.814e-29	6.497e-25
8	Myofibroblasts	*KANK1*	0.7274	0.256	0.074	1.068e-29	2.467e-25
8	Myofibroblasts	*IRAG1*	0.7252	0.271	0.074	1.080e-33	2.494e-29
8	Myofibroblasts	*PHF21A*	0.7220	0.469	0.358	6.799e-08	1.570e-03
8	Myofibroblasts	*BLTP1*	0.7220	0.626	0.495	3.671e-12	8.476e-08
8	Myofibroblasts	*SH3BGRL*	0.7160	0.476	0.348	5.190e-11	1.198e-06
8	Myofibroblasts	*IYD*	0.7156	0.256	0.039	2.749e-65	6.347e-61
8	Myofibroblasts	*REV3L*	0.7145	0.663	0.522	6.328e-14	1.461e-09
8	Myofibroblasts	*TGFBR3*	0.7144	0.718	0.562	1.566e-16	3.615e-12
8	Myofibroblasts	*CERS6*	0.7133	0.293	0.131	1.132e-16	2.613e-12
8	Myofibroblasts	*AUTS2*	0.7109	0.681	0.565	3.181e-12	7.345e-08
8	Myofibroblasts	*XPO1*	0.7104	0.568	0.449	3.048e-10	7.038e-06
8	Myofibroblasts	*CALD1*	0.7078	0.817	0.668	3.231e-27	7.460e-23
8	Myofibroblasts	*CHD3*	0.7073	0.311	0.115	6.305e-25	1.456e-20
8	Myofibroblasts	*MITF*	0.7062	0.740	0.622	2.606e-11	6.017e-07
8	Myofibroblasts	*SLC35F1*	0.7031	0.571	0.416	9.019e-12	2.083e-07
8	Myofibroblasts	*COTL1*	0.7019	0.359	0.130	2.417e-30	5.582e-26
8	Myofibroblasts	*LOC108177718*	0.7001	0.300	0.167	2.073e-10	4.787e-06
8	Myofibroblasts	*PPP1R12B*	0.6993	0.341	0.165	3.250e-16	7.505e-12
8	Myofibroblasts	*AR*	0.6985	0.399	0.243	4.152e-11	9.588e-07
8	Myofibroblasts	*PTBP3*	0.6965	0.363	0.213	1.810e-12	4.179e-08
8	Myofibroblasts	*ASPN*	0.6960	0.234	0.052	3.260e-37	7.529e-33
8	Myofibroblasts	*RHOB*	0.6914	0.740	0.707	2.634e-07	6.084e-03
8	Myofibroblasts	*TMEM158*	0.6874	0.253	0.096	9.952e-19	2.298e-14
8	Myofibroblasts	*ZNF827*	0.6825	0.300	0.148	1.682e-13	3.884e-09
8	Myofibroblasts	*VCL*	0.6808	0.476	0.277	4.173e-17	9.636e-13

Original cluster	Annotated cell type	Gene	avg_log2FC	pct.1	pct.2	Raw P value	Bonferroni-adjusted P value
8	Myofibroblasts	*SIPA1L1*	0.6799	0.407	0.251	1.458e-11	3.366e-07
8	Myofibroblasts	*MAP4K3*	0.6737	0.451	0.293	2.475e-12	5.716e-08
8	Myofibroblasts	*UBE2H*	0.6692	0.476	0.312	2.004e-12	4.627e-08
8	Myofibroblasts	*NPNT*	0.6637	0.326	0.177	1.678e-12	3.875e-08
8	Myofibroblasts	*GAS1*	0.6632	0.733	0.537	2.579e-16	5.956e-12
8	Myofibroblasts	*FBN2*	0.6621	0.249	0.113	5.444e-13	1.257e-08
8	Myofibroblasts	*EMCN*	0.6601	0.337	0.095	6.845e-38	1.581e-33
8	Myofibroblasts	*FAT4*	0.6588	0.414	0.244	7.922e-13	1.829e-08
8	Myofibroblasts	*PDGFC*	0.6580	0.209	0.047	6.485e-33	1.498e-28
8	Myofibroblasts	*ZNF532*	0.6573	0.344	0.181	3.622e-14	8.364e-10
8	Myofibroblasts	*ATF7IP*	0.6549	0.586	0.446	1.499e-10	3.462e-06
8	Myofibroblasts	*CALN1*	0.6546	0.128	0.013	4.615e-49	1.066e-44
8	Myofibroblasts	*DPP6*	0.6529	0.198	0.035	4.012e-42	9.265e-38
8	Myofibroblasts	*MOXD1*	0.6525	0.762	0.632	1.620e-13	3.741e-09
8	Myofibroblasts	*GALNT18*	0.6497	0.242	0.071	1.909e-26	4.408e-22
8	Myofibroblasts	*ITGA1*	0.6469	0.359	0.164	9.961e-18	2.300e-13
8	Myofibroblasts	*RBMS3*	0.6463	0.744	0.651	2.212e-11	5.107e-07
8	Myofibroblasts	*LOC138843476*	0.6455	0.429	0.309	7.419e-07	1.713e-02
8	Myofibroblasts	*HSPA2*	0.6438	0.520	0.388	2.260e-11	5.218e-07
8	Myofibroblasts	*RBMS1*	0.6426	0.670	0.541	4.112e-13	9.496e-09
8	Myofibroblasts	*MAML2*	0.6423	0.601	0.471	7.676e-09	1.772e-04
8	Myofibroblasts	*C4H3orf70*	0.6407	0.238	0.108	1.250e-12	2.887e-08
8	Myofibroblasts	*PTGER3*	0.6402	0.253	0.120	1.376e-11	3.178e-07
8	Myofibroblasts	*PPP1R12A*	0.6375	0.788	0.649	4.518e-19	1.043e-14
8	Myofibroblasts	*KMT2C*	0.6354	0.601	0.474	1.871e-10	4.321e-06
8	Myofibroblasts	*PTBP2*	0.6331	0.542	0.426	1.868e-09	4.314e-05
8	Myofibroblasts	*ZKSCAN8*	0.6319	0.388	0.218	2.656e-13	6.133e-09
8	Myofibroblasts	*SPEN*	0.6316	0.436	0.258	2.564e-14	5.921e-10
8	Myofibroblasts	*PTPRK*	0.6316	0.513	0.358	1.746e-09	4.031e-05
8	Myofibroblasts	*RERE*	0.6312	0.568	0.450	3.801e-10	8.778e-06
8	Myofibroblasts	*EYA1*	0.6264	0.136	0.042	5.210e-14	1.203e-09
8	Myofibroblasts	*NEXN*	0.6264	0.293	0.092	4.113e-28	9.497e-24
8	Myofibroblasts	*LYPD6*	0.6264	0.256	0.126	5.185e-11	1.197e-06
8	Myofibroblasts	*LOC138846563*	0.6221	0.234	0.140	1.054e-06	2.433e-02
8	Myofibroblasts	*PLXDC2*	0.6220	0.689	0.566	9.134e-10	2.109e-05
8	Myofibroblasts	*MACF1*	0.6193	0.637	0.480	5.072e-13	1.171e-08
8	Myofibroblasts	*BAZ2B*	0.6184	0.703	0.587	1.614e-12	3.727e-08
8	Myofibroblasts	*GPCPD1*	0.6176	0.388	0.274	8.529e-07	1.969e-02
8	Myofibroblasts	*CHCHD10*	0.6137	0.410	0.234	7.384e-14	1.705e-09
8	Myofibroblasts	*MPPED2*	0.6133	0.150	0.043	3.741e-17	8.640e-13

Original cluster	Annotated cell type	Gene	avg_log2FC	pct.1	pct.2	Raw P value	Bonferroni-adjusted P value
8	Myofibroblasts	*MCU*	0.6106	0.341	0.199	4.303e-11	9.936e-07
8	Myofibroblasts	*MYOM1*	0.6087	0.187	0.053	4.962e-22	1.146e-17
8	Myofibroblasts	*CACNA1D*	0.6086	0.201	0.066	1.400e-18	3.233e-14
8	Myofibroblasts	*GNG12*	0.6056	0.242	0.106	6.680e-14	1.543e-09
8	Myofibroblasts	*NFIA*	0.6040	0.802	0.630	4.670e-16	1.078e-11
8	Myofibroblasts	*ANKS1B*	0.6031	0.377	0.242	2.858e-08	6.601e-04
8	Myofibroblasts	*NCOR1*	0.6028	0.645	0.526	2.999e-13	6.925e-09
8	Myofibroblasts	*TET2*	0.6027	0.359	0.240	8.616e-08	1.990e-03
8	Myofibroblasts	*CHN2*	0.6006	0.238	0.116	4.043e-11	9.335e-07
8	Myofibroblasts	*PRKN*	0.6003	0.524	0.371	9.614e-10	2.220e-05
8	Myofibroblasts	*SH3GL3*	0.5988	0.223	0.077	1.773e-19	4.094e-15
8	Myofibroblasts	*BTG2*	0.5964	0.821	0.888	1.322e-06	3.053e-02
8	Myofibroblasts	*RFX3*	0.5952	0.652	0.493	1.774e-13	4.098e-09
8	Myofibroblasts	*NCOA3*	0.5945	0.245	0.121	2.546e-11	5.878e-07
8	Myofibroblasts	*NIPAL2*	0.5941	0.278	0.164	1.606e-08	3.709e-04
8	Myofibroblasts	*GPAT3*	0.5905	0.223	0.114	1.350e-08	3.117e-04
8	Myofibroblasts	*PDLIM7*	0.5900	0.242	0.098	2.664e-16	6.152e-12
8	Myofibroblasts	*GATA2*	0.5890	0.553	0.441	7.432e-10	1.716e-05
8	Myofibroblasts	*RBM26*	0.5877	0.509	0.413	8.754e-07	2.021e-02
8	Myofibroblasts	*TRIO*	0.5876	0.374	0.265	9.978e-07	2.304e-02
8	Myofibroblasts	*ETFDH*	0.5861	0.216	0.103	1.556e-10	3.593e-06
8	Myofibroblasts	*PARD3*	0.5858	0.326	0.219	1.715e-06	3.960e-02
8	Myofibroblasts	*RELCH*	0.5826	0.297	0.198	1.876e-06	4.332e-02
8	Myofibroblasts	*KDM6A*	0.5825	0.505	0.383	1.424e-08	3.289e-04
8	Myofibroblasts	*SLMAP*	0.5823	0.363	0.233	2.460e-09	5.681e-05
8	Myofibroblasts	*SENP6*	0.5818	0.601	0.518	2.082e-09	4.808e-05
8	Myofibroblasts	*DDX17*	0.5799	0.498	0.399	4.077e-07	9.415e-03
8	Myofibroblasts	*LOC103349472*	0.5797	0.165	0.057	6.511e-14	1.504e-09
8	Myofibroblasts	*TLN2*	0.5795	0.238	0.118	4.296e-10	9.919e-06
8	Myofibroblasts	*ID4*	0.5789	0.396	0.196	3.918e-17	9.047e-13
8	Myofibroblasts	*SSX2IP*	0.5785	0.245	0.087	6.296e-20	1.454e-15
8	Myofibroblasts	*MSRA*	0.5774	0.227	0.114	1.070e-09	2.470e-05
8	Myofibroblasts	*KLF12*	0.5756	0.788	0.682	3.447e-12	7.959e-08
8	Myofibroblasts	*LOC103348141*	0.5753	0.451	0.348	3.957e-07	9.139e-03
8	Myofibroblasts	*ABL2*	0.5749	0.209	0.091	4.523e-12	1.045e-07
8	Myofibroblasts	*GNAI1*	0.5747	0.322	0.194	8.453e-09	1.952e-04
8	Myofibroblasts	*LOC138848505*	0.5746	0.190	0.079	5.173e-12	1.194e-07
8	Myofibroblasts	*EHBP1*	0.5734	0.700	0.579	6.728e-11	1.554e-06
8	Myofibroblasts	*CHD6*	0.5669	0.502	0.396	1.955e-07	4.514e-03
8	Myofibroblasts	*RSU1*	0.5665	0.443	0.325	9.434e-09	2.179e-04

Original cluster	Annotated cell type	Gene	avg_log2FC	pct.1	pct.2	Raw P value	Bonferroni-adjusted P value
8	Myofibroblasts	*PAN3*	0.5664	0.473	0.364	7.856e-07	1.814e-02
8	Myofibroblasts	*LUC7L3*	0.5629	0.593	0.485	8.691e-09	2.007e-04
8	Myofibroblasts	*SCHIP1*	0.5625	0.220	0.083	2.716e-16	6.272e-12
8	Myofibroblasts	*NARS2*	0.5618	0.249	0.128	4.582e-10	1.058e-05
8	Myofibroblasts	*SLC4A7*	0.5595	0.293	0.167	3.609e-09	8.334e-05
8	Myofibroblasts	*TNRC6B*	0.5555	0.597	0.495	5.973e-08	1.379e-03
8	Myofibroblasts	*LOC138844049*	0.5555	0.136	0.036	1.901e-17	4.389e-13
8	Myofibroblasts	*LOC138849966*	0.5554	0.205	0.106	6.459e-08	1.492e-03
8	Myofibroblasts	*HOXD4*	0.5545	0.333	0.198	1.540e-09	3.555e-05
8	Myofibroblasts	*LARS2*	0.5541	0.198	0.062	1.389e-19	3.208e-15
8	Myofibroblasts	*SYNPO2*	0.5540	0.487	0.379	1.138e-06	2.628e-02
8	Myofibroblasts	*GNAO1*	0.5535	0.183	0.027	8.116e-48	1.874e-43
8	Myofibroblasts	*WWTR1*	0.5521	0.465	0.335	1.284e-08	2.965e-04
8	Myofibroblasts	*MLLT10*	0.5501	0.586	0.474	2.308e-08	5.330e-04
8	Myofibroblasts	*CUX1*	0.5492	0.278	0.166	1.919e-08	4.431e-04
8	Myofibroblasts	*DTNA*	0.5485	0.132	0.020	5.113e-33	1.181e-28
8	Myofibroblasts	*BTRC*	0.5482	0.429	0.303	5.121e-08	1.182e-03
8	Myofibroblasts	*FAM135A*	0.5472	0.374	0.270	2.063e-06	4.765e-02
8	Myofibroblasts	*RPS6KA6*	0.5466	0.198	0.090	3.027e-10	6.991e-06
8	Myofibroblasts	*FNIP2*	0.5440	0.161	0.079	2.369e-07	5.471e-03
8	Myofibroblasts	*HECTD2*	0.5440	0.476	0.343	9.056e-09	2.091e-04
8	Myofibroblasts	*SEMA3C*	0.5438	0.253	0.072	8.901e-28	2.056e-23
8	Myofibroblasts	*DMXL1*	0.5433	0.352	0.245	1.865e-06	4.306e-02
8	Myofibroblasts	*ATP8A1*	0.5432	0.198	0.096	8.254e-09	1.906e-04
8	Myofibroblasts	*ECPAS*	0.5401	0.352	0.224	1.648e-08	3.805e-04
8	Myofibroblasts	*SIMC1*	0.5352	0.220	0.117	3.824e-08	8.831e-04
8	Myofibroblasts	*PTPRR*	0.5351	0.158	0.030	3.462e-30	7.995e-26
8	Myofibroblasts	*HSD17B4*	0.5344	0.363	0.255	2.205e-07	5.092e-03
8	Myofibroblasts	*PRRX1*	0.5325	0.637	0.536	1.092e-09	2.521e-05
8	Myofibroblasts	*ARHGAP32*	0.5306	0.414	0.308	2.028e-06	4.684e-02
8	Myofibroblasts	*ITGA9*	0.5240	0.179	0.084	7.215e-09	1.666e-04
8	Myofibroblasts	*COL6A3*	0.5206	0.242	0.124	1.201e-09	2.774e-05
8	Myofibroblasts	*FNBP1*	0.5199	0.275	0.153	2.692e-09	6.216e-05
8	Myofibroblasts	*NCAM1*	0.5185	0.326	0.192	1.664e-08	3.844e-04
8	Myofibroblasts	*NBL1*	0.5153	0.443	0.295	5.073e-10	1.172e-05
8	Myofibroblasts	*SVIL*	0.5147	0.502	0.377	1.074e-07	2.480e-03
8	Myofibroblasts	*PPP2R5A*	0.5140	0.337	0.226	5.840e-07	1.348e-02
8	Myofibroblasts	*DTNB*	0.5106	0.315	0.207	2.629e-07	6.072e-03
8	Myofibroblasts	*LRIG1*	0.5062	0.201	0.064	8.413e-20	1.943e-15
8	Myofibroblasts	*SYT9*	0.5055	0.128	0.021	1.021e-30	2.359e-26

Original cluster	Annotated cell type	Gene	avg_log2FC	pct.1	pct.2	Raw P value	Bonferroni-adjusted P value
8	Myofibroblasts	*RANBP9*	0.5052	0.396	0.274	8.082e-08	1.866e-03
8	Myofibroblasts	*GLRB*	0.5051	0.154	0.037	9.335e-23	2.156e-18
8	Myofibroblasts	*RNF180*	0.5051	0.158	0.063	9.936e-11	2.294e-06
8	Myofibroblasts	*NFAT5*	0.5019	0.579	0.483	3.554e-07	8.206e-03
8	Myofibroblasts	*GLI3*	0.4966	0.286	0.142	9.032e-12	2.086e-07
8	Myofibroblasts	*LOC138847375*	0.4931	0.212	0.106	3.529e-09	8.150e-05
8	Myofibroblasts	*AGPS*	0.4913	0.190	0.094	1.477e-08	3.411e-04
8	Myofibroblasts	*ITGA8*	0.4910	0.161	0.077	1.469e-07	3.393e-03
8	Myofibroblasts	*ZNF462*	0.4887	0.201	0.111	3.805e-07	8.786e-03
8	Myofibroblasts	*HSPB8*	0.4885	0.147	0.035	6.273e-22	1.449e-17
8	Myofibroblasts	*CHRDL1*	0.4875	0.139	0.037	7.254e-18	1.675e-13
8	Myofibroblasts	*AAK1*	0.4861	0.245	0.146	3.848e-07	8.885e-03
8	Myofibroblasts	*BMPR1A*	0.4857	0.418	0.280	6.020e-09	1.390e-04
8	Myofibroblasts	*RGS7BP*	0.4851	0.136	0.023	4.499e-31	1.039e-26
8	Myofibroblasts	*TMEM26*	0.4823	0.154	0.071	1.028e-07	2.375e-03
8	Myofibroblasts	*KCNMB1*	0.4780	0.172	0.018	6.482e-64	1.497e-59
8	Myofibroblasts	*LRRC17*	0.4772	0.337	0.216	3.271e-07	7.554e-03
8	Myofibroblasts	*LOC103349764*	0.4758	0.187	0.069	2.829e-14	6.533e-10
8	Myofibroblasts	*CLMP*	0.4726	0.282	0.173	7.330e-07	1.693e-02
8	Myofibroblasts	*SRGAP1*	0.4707	0.172	0.063	1.873e-13	4.325e-09
8	Myofibroblasts	*MEF2A*	0.4696	0.454	0.337	1.059e-07	2.446e-03
8	Myofibroblasts	*PDE7B*	0.4692	0.168	0.064	4.669e-12	1.078e-07
8	Myofibroblasts	*RNF125*	0.4633	0.282	0.152	6.567e-10	1.516e-05
8	Myofibroblasts	*LOC108175891*	0.4628	0.245	0.140	1.736e-07	4.008e-03
8	Myofibroblasts	*NAALADL2*	0.4628	0.751	0.609	3.475e-11	8.026e-07
8	Myofibroblasts	*LOC100346860*	0.4624	0.132	0.029	1.351e-21	3.119e-17
8	Myofibroblasts	*PDZRN4*	0.4621	0.128	0.036	2.047e-15	4.728e-11
8	Myofibroblasts	*ABI3BP*	0.4609	0.355	0.225	4.036e-07	9.321e-03
8	Myofibroblasts	*RAB3IP*	0.4593	0.205	0.082	2.210e-13	5.103e-09
8	Myofibroblasts	*CDC42EP3*	0.4565	0.161	0.041	3.535e-22	8.163e-18
8	Myofibroblasts	*LMOD1*	0.4539	0.176	0.045	2.945e-23	6.801e-19
8	Myofibroblasts	*KMT2A*	0.4534	0.542	0.463	1.346e-06	3.107e-02
8	Myofibroblasts	*FERMT2*	0.4512	0.458	0.334	1.424e-07	3.287e-03
8	Myofibroblasts	*CCDC3*	0.4509	0.231	0.106	9.029e-12	2.085e-07
8	Myofibroblasts	*RSF1*	0.4500	0.546	0.464	1.803e-06	4.164e-02
8	Myofibroblasts	*KAT2B*	0.4481	0.293	0.185	1.904e-06	4.396e-02
8	Myofibroblasts	*JMJD1C*	0.4481	0.766	0.703	6.279e-08	1.450e-03
8	Myofibroblasts	*RALGAPA1*	0.4441	0.549	0.457	1.203e-06	2.779e-02
8	Myofibroblasts	*KCNH1*	0.4439	0.106	0.036	1.143e-09	2.639e-05
8	Myofibroblasts	*ANGPT1*	0.4379	0.267	0.141	3.826e-09	8.834e-05

Original cluster	Annotated cell type	Gene	avg_log2FC	pct.1	pct.2	Raw P value	Bonferroni-adjusted P value
8	Myofibroblasts	*BCL11A*	0.4371	0.158	0.049	9.273e-16	2.141e-11
8	Myofibroblasts	*IRS1*	0.4364	0.249	0.136	2.006e-08	4.633e-04
8	Myofibroblasts	*TXNIP*	0.4286	0.839	0.842	4.874e-09	1.126e-04
8	Myofibroblasts	*DOCK9*	0.4286	0.348	0.200	7.449e-10	1.720e-05
8	Myofibroblasts	*MBNL1*	0.4250	0.707	0.665	6.462e-07	1.492e-02
8	Myofibroblasts	*PPP1R9A*	0.4205	0.304	0.193	9.572e-07	2.210e-02
8	Myofibroblasts	*LOC138845125*	0.4193	0.194	0.102	2.316e-07	5.349e-03
8	Myofibroblasts	*BICD1*	0.4175	0.132	0.037	1.902e-15	4.392e-11
8	Myofibroblasts	*ASB2*	0.4146	0.128	0.006	7.300e-94	1.686e-89
8	Myofibroblasts	*TMEM178A*	0.4116	0.158	0.075	2.686e-07	6.203e-03
8	Myofibroblasts	*TGFB2*	0.4016	0.179	0.089	7.381e-08	1.704e-03
8	Myofibroblasts	*ARHGAP12*	0.3987	0.278	0.163	1.114e-07	2.571e-03
8	Myofibroblasts	*CST3*	0.3981	0.516	0.374	5.321e-09	1.229e-04
8	Myofibroblasts	*MED13L*	0.3959	0.729	0.657	6.402e-07	1.478e-02
8	Myofibroblasts	*LOC103349468*	0.3921	0.106	0.039	2.109e-08	4.869e-04
8	Myofibroblasts	*LOC103350898*	0.3914	0.158	0.075	2.230e-07	5.150e-03
8	Myofibroblasts	*CORO1C*	0.3911	0.158	0.078	4.599e-07	1.062e-02
8	Myofibroblasts	*LOC100356313*	0.3898	0.106	0.028	8.560e-14	1.977e-09
8	Myofibroblasts	*DUSP26*	0.3885	0.337	0.223	1.050e-06	2.424e-02
8	Myofibroblasts	*MFAP4*	0.3877	0.267	0.153	1.738e-07	4.013e-03
8	Myofibroblasts	*PFN1*	0.3846	0.612	0.560	2.022e-06	4.669e-02
8	Myofibroblasts	*COL8A1*	0.3805	0.136	0.047	1.942e-11	4.485e-07
8	Myofibroblasts	*CD109*	0.3773	0.154	0.058	3.067e-11	7.082e-07
8	Myofibroblasts	*ITM2C*	0.3745	0.377	0.240	1.248e-08	2.882e-04
8	Myofibroblasts	*LOC138848991*	0.3732	0.150	0.063	4.060e-09	9.375e-05
8	Myofibroblasts	*TSPAN2*	0.3728	0.150	0.059	1.462e-10	3.375e-06
8	Myofibroblasts	*GPR20*	0.3708	0.168	0.062	6.139e-13	1.418e-08
8	Myofibroblasts	*CDK1*	0.3694	0.132	0.035	1.071e-16	2.473e-12
8	Myofibroblasts	*SORCS1*	0.3689	0.106	0.025	5.470e-16	1.263e-11
8	Myofibroblasts	*STON1*	0.3648	0.179	0.081	5.664e-09	1.308e-04
8	Myofibroblasts	*HFM1*	0.3611	0.201	0.103	8.027e-08	1.854e-03
8	Myofibroblasts	*ACTB*	0.3596	0.930	0.973	4.448e-09	1.027e-04
8	Myofibroblasts	*ZFHX3*	0.3591	0.319	0.195	1.603e-07	3.702e-03
8	Myofibroblasts	*KCNMB4*	0.3532	0.158	0.072	5.148e-08	1.189e-03
8	Myofibroblasts	*LOC103348321*	0.3526	0.114	0.040	1.536e-09	3.546e-05
8	Myofibroblasts	*KEF51-p04*	0.3514	0.989	0.990	3.404e-12	7.860e-08
8	Myofibroblasts	*RETREG1*	0.3511	0.183	0.095	5.086e-07	1.175e-02
8	Myofibroblasts	*KCTD1*	0.3510	0.125	0.049	2.643e-08	6.103e-04
8	Myofibroblasts	*IL6R*	0.3508	0.110	0.047	1.125e-06	2.599e-02
8	Myofibroblasts	*EEFSEC*	0.3479	0.128	0.058	1.087e-06	2.511e-02

Original cluster	Annotated cell type	Gene	avg_log2FC	pct.1	pct.2	Raw P value	Bonferroni-adjusted P value
8	Myofibroblasts	*LOC127491762*	0.3463	0.161	0.079	5.546e-07	1.281e-02
8	Myofibroblasts	*LOC100353768*	0.3406	0.103	0.021	6.967e-19	1.609e-14
8	Myofibroblasts	*AQP1*	0.3242	0.165	0.067	4.635e-10	1.070e-05
8	Myofibroblasts	*FLNA*	0.3207	0.128	0.043	1.162e-11	2.683e-07
8	Myofibroblasts	*MMP11*	0.3158	0.106	0.031	4.700e-12	1.085e-07
8	Myofibroblasts	*KEF51-p08*	0.3074	0.996	0.994	1.074e-15	2.479e-11
8	Myofibroblasts	*AKAP1*	0.3027	0.132	0.056	4.109e-08	9.489e-04
8	Myofibroblasts	*KEF51-p01*	0.3023	0.993	0.990	2.698e-08	6.230e-04
8	Myofibroblasts	*TEC*	0.3014	0.253	0.150	1.346e-06	3.108e-02
8	Myofibroblasts	*PLEKHO1*	0.2970	0.147	0.059	1.700e-09	3.926e-05
8	Myofibroblasts	*KEF51-p13*	0.2902	0.982	0.986	1.261e-07	2.912e-03
8	Myofibroblasts	*COL4A2*	0.2860	0.190	0.101	1.734e-06	4.005e-02
8	Myofibroblasts	*SATB1*	0.2807	0.154	0.075	8.492e-07	1.961e-02
8	Myofibroblasts	*KEF51-p07*	0.2657	0.993	0.994	5.544e-11	1.280e-06
8	Myofibroblasts	*VPS37D*	0.2639	0.114	0.048	4.731e-07	1.093e-02
8	Myofibroblasts	*KEF51-p10*	0.2571	0.996	0.994	4.038e-10	9.326e-06
8	Myofibroblasts	*EML2*	0.2568	0.154	0.076	1.172e-06	2.707e-02
10	**Epithelial cells**	*SLC26A4*	4.2782	0.928	0.005	0	0
10	Epithelial cells	*MECOM*	4.2595	0.986	0.041	0	0
10	Epithelial cells	*LOC103350677*	4.2227	0.913	0.012	0	0
10	Epithelial cells	*PKHD1*	4.1899	0.928	0.007	0	0
10	Epithelial cells	*ERBB4*	3.8069	0.891	0.013	0	0
10	Epithelial cells	*LGR5*	3.6615	0.891	0.013	0	0
10	Epithelial cells	*MMP7*	3.5811	0.659	0.002	0	0
10	Epithelial cells	*PTPRR*	3.3963	0.855	0.022	0	0
10	Epithelial cells	*CLDN3*	3.2825	0.942	0.003	0	0
10	Epithelial cells	*DEFB1*	3.1996	0.775	0.010	0	0
10	Epithelial cells	*PDE4D*	3.1116	0.949	0.171	2.356e-157	5.441e-153
10	Epithelial cells	*LOC127482640*	2.9640	0.761	0.004	0	0
10	Epithelial cells	*BMPR1B*	2.8546	0.899	0.018	0	0
10	Epithelial cells	*CCSER1*	2.7302	0.877	0.128	9.966e-161	2.301e-156
10	Epithelial cells	*NAALADL2*	2.7198	1.000	0.608	2.389e-73	5.517e-69
10	Epithelial cells	*TPD52L1*	2.6919	0.964	0.275	1.686e-112	3.893e-108
10	Epithelial cells	*FMN1*	2.6467	0.848	0.009	0	0
10	Epithelial cells	*KCNQ5*	2.6164	0.826	0.045	0	0
10	Epithelial cells	*RNF144B*	2.6155	0.841	0.022	0	0
10	Epithelial cells	*CFTR*	2.5921	0.761	0.007	0	0
10	Epithelial cells	*MUC1*	2.5622	0.891	0.002	0	0
10	Epithelial cells	*ANK2*	2.4889	0.725	0.331	2.939e-35	6.787e-31
10	Epithelial cells	*PDE7A*	2.4872	0.884	0.076	2.030e-261	4.687e-257

Original cluster	Annotated cell type	Gene	avg_log2FC	pct.1	pct.2	Raw P value	Bonferroni-adjusted P value
10	Epithelial cells	*PDGFC*	2.4864	0.833	0.040	0	0
10	Epithelial cells	*MSX1*	2.4560	0.891	0.023	0	0
10	Epithelial cells	*EPCAM*	2.4484	0.899	0.002	0	0
10	Epithelial cells	*FHIP1A*	2.4476	0.855	0.035	0	0
10	Epithelial cells	*CYTL1*	2.4326	0.790	0.007	0	0
10	Epithelial cells	*HMGA2*	2.4308	0.783	0.016	0	0
10	Epithelial cells	*NTN4*	2.3947	0.870	0.064	9.253e-292	2.137e-287
10	Epithelial cells	*SORBS2*	2.3844	0.870	0.080	4.533e-236	1.047e-231
10	Epithelial cells	*SIPA1L1*	2.3789	0.920	0.245	9.097e-108	2.101e-103
10	Epithelial cells	*SYNE2*	2.3521	0.942	0.206	1.241e-131	2.865e-127
10	Epithelial cells	*GPC3*	2.3469	0.841	0.051	0	0
10	Epithelial cells	*ARAP2*	2.3398	0.812	0.023	0	0
10	Epithelial cells	*LOC127491701*	2.2766	0.681	0.005	0	0
10	Epithelial cells	*ADGRL3*	2.2449	0.906	0.326	6.851e-73	1.582e-68
10	Epithelial cells	*CLDN8*	2.2355	0.826	0.001	0	0
10	Epithelial cells	*PATJ*	2.2260	0.877	0.059	0	0
10	Epithelial cells	*STC1*	2.2118	0.804	0.026	0	0
10	Epithelial cells	*PLEKHS1*	2.1643	0.696	0.003	0	0
10	Epithelial cells	*BMP6*	2.1411	0.659	0.004	0	0
10	Epithelial cells	*GADD45A*	2.1339	0.877	0.266	1.684e-78	3.888e-74
10	Epithelial cells	*STAT4*	2.1203	0.761	0.016	0	0
10	Epithelial cells	*CDK8*	2.1039	0.739	0.083	7.990e-161	1.845e-156
10	Epithelial cells	*ANKRD44*	2.0980	0.833	0.195	5.668e-94	1.309e-89
10	Epithelial cells	*TNFRSF19*	2.0916	0.891	0.229	1.681e-99	3.881e-95
10	Epithelial cells	*ATP13A3*	2.0686	0.855	0.091	2.573e-206	5.941e-202
10	Epithelial cells	*LRP1B*	2.0440	0.645	0.111	1.615e-85	3.730e-81
10	Epithelial cells	*LGALS3*	2.0278	0.775	0.069	8.599e-209	1.986e-204
10	Epithelial cells	*LOC138842959*	2.0216	0.725	0.002	0	0
10	Epithelial cells	*SGCD*	2.0091	0.681	0.248	2.322e-41	5.361e-37
10	Epithelial cells	*GPHN*	2.0073	0.957	0.487	5.830e-59	1.346e-54
10	Epithelial cells	*SLC15A2*	1.9824	0.804	0.008	0	0
10	Epithelial cells	*CLDN10*	1.9396	0.833	0.031	0	0
10	Epithelial cells	*LOC138844677*	1.9244	0.594	0.004	0	0
10	Epithelial cells	*MUC15*	1.9185	0.674	0.003	0	0
10	Epithelial cells	*LOC138848811*	1.9077	0.725	0.072	9.916e-173	2.290e-168
10	Epithelial cells	*GRO-A*	1.8967	0.370	0.037	3.438e-83	7.940e-79
10	Epithelial cells	*AUTS2*	1.8961	0.957	0.563	1.025e-59	2.366e-55
10	Epithelial cells	*LOC100344368*	1.8959	0.717	0.061	1.249e-202	2.883e-198
10	Epithelial cells	*KRT18*	1.8913	0.739	0.002	0	0
10	Epithelial cells	*DNM3*	1.8882	0.761	0.116	4.244e-117	9.800e-113

Original cluster	Annotated cell type	Gene	avg_log2FC	pct.1	pct.2	Raw P value	Bonferroni-adjusted P value
10	Epithelial cells	*KHDRBS2*	1.8878	0.312	0.020	1.057e-107	2.441e-103
10	Epithelial cells	*MPPED2*	1.8813	0.761	0.036	0	0
10	Epithelial cells	*LOC103349149*	1.8747	0.710	0.010	0	0
10	Epithelial cells	*TSC22D1*	1.8542	0.986	0.605	6.517e-68	1.505e-63
10	Epithelial cells	*NRG3*	1.8445	0.514	0.017	0	0
10	Epithelial cells	*DLX5*	1.8427	0.761	0.003	0	0
10	Epithelial cells	*PRXL2A*	1.8426	0.877	0.172	4.230e-119	9.768e-115
10	Epithelial cells	*SORCS1*	1.8424	0.652	0.018	0	0
10	Epithelial cells	*TMEM123*	1.8413	0.891	0.212	5.699e-101	1.316e-96
10	Epithelial cells	*GRAMD1B*	1.8366	0.790	0.037	0	0
10	Epithelial cells	*KRT8*	1.8301	0.812	0.007	0	0
10	Epithelial cells	*AOX1*	1.8114	0.790	0.107	8.637e-144	1.994e-139
10	Epithelial cells	*PTPRK*	1.8083	0.891	0.355	6.461e-63	1.492e-58
10	Epithelial cells	*LOC127491661*	1.8081	0.565	0.002	0	0
10	Epithelial cells	*KCTD1*	1.7905	0.819	0.040	0	0
10	Epithelial cells	*FGF13*	1.7865	0.457	0.072	1.507e-65	3.481e-61
10	Epithelial cells	*PLCB1*	1.7859	0.928	0.391	6.530e-60	1.508e-55
10	Epithelial cells	*KRT19*	1.7833	0.841	0.091	3.290e-184	7.598e-180
10	Epithelial cells	*C7H1orf226*	1.7821	0.754	0.049	7.922e-266	1.829e-261
10	Epithelial cells	*DCDC2*	1.7689	0.681	0.002	0	0
10	Epithelial cells	*MLLT3*	1.7586	0.884	0.367	1.048e-54	2.420e-50
10	Epithelial cells	*ATF3*	1.7494	0.848	0.506	6.576e-27	1.518e-22
10	Epithelial cells	*ID2*	1.7485	0.906	0.341	5.233e-58	1.208e-53
10	Epithelial cells	*CDON*	1.7469	0.862	0.138	1.144e-131	2.642e-127
10	Epithelial cells	*ARHGEF28*	1.7397	0.754	0.013	0	0
10	Epithelial cells	*GRIP1*	1.7321	0.826	0.194	3.187e-79	7.360e-75
10	Epithelial cells	*IGF2BP2*	1.7217	0.775	0.104	4.173e-142	9.635e-138
10	Epithelial cells	*LOC108175891*	1.7186	0.775	0.133	2.082e-106	4.807e-102
10	Epithelial cells	*DUS4L*	1.7084	0.674	0.060	5.773e-178	1.333e-173
10	Epithelial cells	*CADM1*	1.7058	0.877	0.201	8.082e-95	1.866e-90
10	Epithelial cells	*BEND7*	1.6944	0.797	0.059	3.984e-255	9.200e-251
10	Epithelial cells	*LOC138844494*	1.6842	0.529	0.037	1.626e-173	3.755e-169
10	Epithelial cells	*TMEM72*	1.6752	0.754	0.001	0	0
10	Epithelial cells	*ITPRID2*	1.6699	0.739	0.145	2.399e-90	5.540e-86
10	Epithelial cells	*SH3RF1*	1.6643	0.804	0.186	2.633e-78	6.081e-74
10	Epithelial cells	*TBC1D4*	1.6590	0.725	0.127	1.270e-97	2.933e-93
10	Epithelial cells	*PIP4K2A*	1.6471	0.913	0.305	1.025e-73	2.367e-69
10	Epithelial cells	*TENM4*	1.6413	0.870	0.291	6.279e-58	1.450e-53
10	Epithelial cells	*LOC138844929*	1.6395	0.217	0.002	2.704e-286	6.245e-282
10	Epithelial cells	*PLCB4*	1.6247	0.717	0.080	1.338e-147	3.089e-143

Original cluster	Annotated cell type	Gene	avg_log2FC	pct.1	pct.2	Raw P value	Bonferroni-adjusted P value
10	Epithelial cells	*DOCK9*	1.6171	0.833	0.195	4.699e-87	1.085e-82
10	Epithelial cells	*PTPRZ1*	1.5972	0.594	0.008	0	0
10	Epithelial cells	*PERP*	1.5928	0.797	0.031	0	0
10	Epithelial cells	*RERG*	1.5813	0.841	0.188	5.002e-83	1.155e-78
10	Epithelial cells	*COG5*	1.5711	0.819	0.258	3.257e-59	7.521e-55
10	Epithelial cells	*WFDC2*	1.5635	0.761	0.000	0	0
10	Epithelial cells	*GABRB1*	1.5618	0.652	0.065	1.290e-148	2.978e-144
10	Epithelial cells	*STXBP6*	1.5461	0.797	0.110	6.180e-136	1.427e-131
10	Epithelial cells	*MAGI1*	1.5294	0.826	0.124	2.148e-127	4.960e-123
10	Epithelial cells	*SKAP1*	1.5289	0.725	0.014	0	0
10	Epithelial cells	*RBM47*	1.5244	0.732	0.016	0	0
10	Epithelial cells	*DACH1*	1.5223	0.819	0.263	2.238e-49	5.169e-45
10	Epithelial cells	*TACSTD2*	1.5095	0.667	0.002	0	0
10	Epithelial cells	*LOC100357899*	1.5034	0.768	0.114	7.194e-122	1.661e-117
10	Epithelial cells	*LRBA*	1.4976	0.891	0.351	1.723e-55	3.979e-51
10	Epithelial cells	*PTPRM*	1.4895	0.826	0.258	4.180e-58	9.653e-54
10	Epithelial cells	*SESN3*	1.4884	0.906	0.463	9.570e-48	2.210e-43
10	Epithelial cells	*FLNB*	1.4858	0.790	0.126	4.626e-116	1.068e-111
10	Epithelial cells	*YAP1*	1.4852	0.920	0.405	9.096e-55	2.101e-50
10	Epithelial cells	*DPYD*	1.4722	0.645	0.127	1.572e-71	3.629e-67
10	Epithelial cells	*LOC100349085*	1.4678	0.877	0.294	2.584e-59	5.968e-55
10	Epithelial cells	*MDGA2*	1.4329	0.493	0.028	5.924e-192	1.368e-187
10	Epithelial cells	*DTHD1*	1.4288	0.514	0.003	0	0
10	Epithelial cells	*LOC138850460*	1.4260	0.225	0.006	1.636e-154	3.777e-150
10	Epithelial cells	*CLDN7*	1.4236	0.659	0.001	0	0
10	Epithelial cells	*SCGB1A1*	1.4088	0.304	0.001	0	0
10	Epithelial cells	*LOC138848040*	1.4028	1.000	0.898	3.898e-08	9.002e-04
10	Epithelial cells	*C18H19orf33*	1.4024	0.587	0.001	0	0
10	Epithelial cells	*HOXB3*	1.3945	0.725	0.042	1.937e-279	4.472e-275
10	Epithelial cells	*EHF*	1.3939	0.652	0.001	0	0
10	Epithelial cells	*MAP7*	1.3896	0.681	0.005	0	0
10	Epithelial cells	*TRIM2*	1.3888	0.826	0.162	6.684e-98	1.544e-93
10	Epithelial cells	*NPDC1*	1.3829	0.826	0.118	7.104e-134	1.640e-129
10	Epithelial cells	*SPINT2*	1.3820	0.913	0.438	3.622e-53	8.364e-49
10	Epithelial cells	*LOC138844224*	1.3784	0.507	0.017	1.683e-299	3.885e-295
10	Epithelial cells	*WDR72*	1.3711	0.558	0.009	0	0
10	Epithelial cells	*GDF15*	1.3660	0.290	0.014	1.738e-129	4.014e-125
10	Epithelial cells	*CDH6*	1.3611	0.601	0.044	4.667e-186	1.078e-181
10	Epithelial cells	*ARHGEF3*	1.3595	0.688	0.065	3.765e-170	8.694e-166
10	Epithelial cells	*SRPK2*	1.3503	0.913	0.415	5.554e-46	1.282e-41

Original cluster	Annotated cell type	Gene	avg_log2FC	pct.1	pct.2	Raw P value	Bonferroni-adjusted P value
10	Epithelial cells	*RIOK3*	1.3432	0.630	0.208	8.865e-34	2.047e-29
10	Epithelial cells	*NFAT5*	1.3354	0.913	0.479	2.629e-40	6.071e-36
10	Epithelial cells	*LOC127487635*	1.3237	0.384	0.002	0	0
10	Epithelial cells	*CASD1*	1.3232	0.746	0.229	1.887e-49	4.357e-45
10	Epithelial cells	*LOC100328967*	1.3225	0.877	0.271	2.675e-60	6.178e-56
10	Epithelial cells	*NR3C2*	1.3205	0.790	0.320	1.419e-38	3.277e-34
10	Epithelial cells	*PCDH17*	1.3190	0.580	0.023	4.045e-303	9.340e-299
10	Epithelial cells	*CACHD1*	1.3182	0.732	0.164	1.850e-72	4.272e-68
10	Epithelial cells	*SDC4*	1.3115	0.681	0.061	1.833e-170	4.232e-166
10	Epithelial cells	*SLC9A7*	1.3093	0.703	0.068	1.771e-168	4.090e-164
10	Epithelial cells	*FGD4*	1.3076	0.696	0.080	1.414e-142	3.266e-138
10	Epithelial cells	*RBBP8*	1.3066	0.652	0.030	2.635e-307	6.085e-303
10	Epithelial cells	*DLGAP1*	1.3043	0.587	0.106	1.283e-69	2.963e-65
10	Epithelial cells	*MACROD2*	1.2995	0.855	0.347	1.475e-40	3.406e-36
10	Epithelial cells	*FHIT*	1.2977	0.812	0.381	1.139e-31	2.629e-27
10	Epithelial cells	*SLC15A1*	1.2966	0.638	0.001	0	0
10	Epithelial cells	*LUC7L2*	1.2933	0.884	0.322	2.120e-55	4.895e-51
10	Epithelial cells	*KDR*	1.2904	0.616	0.031	4.500e-266	1.039e-261
10	Epithelial cells	*ANK3*	1.2881	0.819	0.245	1.181e-59	2.728e-55
10	Epithelial cells	*TSPAN1*	1.2878	0.645	0.001	0	0
10	Epithelial cells	*PAK1*	1.2874	0.609	0.019	0	0
10	Epithelial cells	*CDH1*	1.2873	0.630	0.001	0	0
10	Epithelial cells	*CHL1*	1.2744	0.457	0.006	0	0
10	Epithelial cells	*PAX8*	1.2719	0.551	0.001	0	0
10	Epithelial cells	*SFTA2*	1.2625	0.623	0.001	0	0
10	Epithelial cells	*IL15*	1.2571	0.696	0.125	3.498e-84	8.078e-80
10	Epithelial cells	*MRTFB*	1.2518	0.819	0.216	4.203e-65	9.705e-61
10	Epithelial cells	*GCNT2*	1.2505	0.609	0.043	4.538e-192	1.048e-187
10	Epithelial cells	*SOX17*	1.2491	0.674	0.030	0	0
10	Epithelial cells	*PKP4*	1.2468	0.790	0.252	2.184e-50	5.042e-46
10	Epithelial cells	*MAP3K13*	1.2443	0.761	0.236	5.724e-45	1.322e-40
10	Epithelial cells	*KLF5*	1.2440	0.725	0.133	1.347e-86	3.110e-82
10	Epithelial cells	*ASAP1*	1.2416	0.862	0.310	3.100e-50	7.160e-46
10	Epithelial cells	*GLIS3*	1.2397	0.848	0.347	6.767e-41	1.563e-36
10	Epithelial cells	*GALNT1*	1.2388	0.833	0.285	2.141e-50	4.944e-46
10	Epithelial cells	*CD9*	1.2376	0.754	0.071	1.726e-179	3.986e-175
10	Epithelial cells	*ITGA2*	1.2367	0.652	0.064	1.751e-149	4.043e-145
10	Epithelial cells	*TENM3*	1.2361	0.804	0.399	8.727e-28	2.015e-23
10	Epithelial cells	*TRPM3*	1.2351	0.609	0.051	5.354e-163	1.236e-158
10	Epithelial cells	*THRB*	1.2325	0.826	0.279	2.469e-46	5.701e-42

Original cluster	Annotated cell type	Gene	avg_log2FC	pct.1	pct.2	Raw P value	Bonferroni-adjusted P value
10	Epithelial cells	*SGK3*	1.2317	0.667	0.124	2.717e-76	6.274e-72
10	Epithelial cells	*LOC127493011*	1.2248	0.486	0.022	3.009e-230	6.949e-226
10	Epithelial cells	*FBXO10*	1.2234	0.594	0.040	2.077e-195	4.796e-191
10	Epithelial cells	*CTTNBP2*	1.2233	0.681	0.108	2.475e-90	5.716e-86
10	Epithelial cells	*PLCE1*	1.2169	0.710	0.148	6.557e-72	1.514e-67
10	Epithelial cells	*NHSL1*	1.2167	0.616	0.062	7.749e-139	1.789e-134
10	Epithelial cells	*BST2*	1.2142	0.848	0.305	1.638e-40	3.782e-36
10	Epithelial cells	*SLIT2*	1.2096	0.804	0.243	2.894e-50	6.683e-46
10	Epithelial cells	*MAML2*	1.2091	0.920	0.468	6.633e-36	1.532e-31
10	Epithelial cells	*NWD2*	1.2040	0.239	0.024	5.775e-54	1.334e-49
10	Epithelial cells	*PTPN4*	1.2006	0.739	0.225	5.674e-49	1.310e-44
10	Epithelial cells	*MGST3*	1.1999	0.877	0.462	9.752e-30	2.252e-25
10	Epithelial cells	*LACC1*	1.1982	0.688	0.177	3.251e-54	7.507e-50
10	Epithelial cells	*RUNX1*	1.1974	0.580	0.035	1.019e-208	2.353e-204
10	Epithelial cells	*ARHGAP42*	1.1929	0.775	0.302	3.239e-36	7.479e-32
10	Epithelial cells	*SOX7*	1.1921	0.529	0.028	3.182e-215	7.349e-211
10	Epithelial cells	*SAT1*	1.1871	0.877	0.416	4.689e-39	1.083e-34
10	Epithelial cells	*KDM7A*	1.1845	0.696	0.136	8.953e-74	2.068e-69
10	Epithelial cells	*KLC3*	1.1811	0.688	0.005	0	0
10	Epithelial cells	*SHROOM3*	1.1801	0.616	0.066	9.066e-130	2.093e-125
10	Epithelial cells	*TJP1*	1.1800	0.775	0.222	2.372e-54	5.477e-50
10	Epithelial cells	*MCTP1*	1.1795	0.399	0.034	8.127e-105	1.877e-100
10	Epithelial cells	*OCLN*	1.1775	0.616	0.005	0	0
10	Epithelial cells	*LOC103348141*	1.1711	0.870	0.343	7.532e-41	1.739e-36
10	Epithelial cells	*DYNC1I1*	1.1684	0.710	0.028	0	0
10	Epithelial cells	*STOX2*	1.1662	0.710	0.122	2.388e-90	5.515e-86
10	Epithelial cells	*TMOD3*	1.1604	0.804	0.236	2.462e-55	5.685e-51
10	Epithelial cells	*DSP*	1.1590	0.638	0.004	0	0
10	Epithelial cells	*MNDA*	1.1552	0.862	0.320	3.275e-46	7.564e-42
10	Epithelial cells	*BAMBI*	1.1496	0.609	0.057	3.426e-144	7.911e-140
10	Epithelial cells	*CEP162*	1.1468	0.674	0.200	1.865e-39	4.306e-35
10	Epithelial cells	*NOTUM*	1.1445	0.580	0.038	2.158e-198	4.983e-194
10	Epithelial cells	*TEAD1*	1.1415	0.877	0.442	1.271e-39	2.935e-35
10	Epithelial cells	*CASK*	1.1406	0.790	0.299	1.311e-38	3.028e-34
10	Epithelial cells	*NOTCH2*	1.1395	0.855	0.324	7.922e-44	1.829e-39
10	Epithelial cells	*MET*	1.1377	0.601	0.027	8.676e-284	2.003e-279
10	Epithelial cells	*CFD*	1.1348	0.971	0.602	9.229e-33	2.131e-28
10	Epithelial cells	*FRYL*	1.1328	0.761	0.234	2.469e-48	5.702e-44
10	Epithelial cells	*CHD7*	1.1328	0.587	0.030	7.551e-252	1.744e-247
10	Epithelial cells	*LOC127493958*	1.1323	0.609	0.028	3.851e-276	8.893e-272

Original cluster	Annotated cell type	Gene	avg_log2FC	pct.1	pct.2	Raw P value	Bonferroni-adjusted P value
10	Epithelial cells	*PLCH1*	1.1316	0.565	0.080	2.044e-85	4.720e-81
10	Epithelial cells	*PUM1*	1.1299	0.884	0.425	4.841e-43	1.118e-38
10	Epithelial cells	*CLDN4*	1.1293	0.406	0.001	0	0
10	Epithelial cells	*RESF1*	1.1271	0.659	0.067	1.790e-144	4.135e-140
10	Epithelial cells	*MAN2A1*	1.1266	0.790	0.217	2.432e-58	5.617e-54
10	Epithelial cells	*EYA4*	1.1239	0.536	0.078	2.447e-79	5.652e-75
10	Epithelial cells	*MAP3K1*	1.1212	0.754	0.152	2.625e-80	6.063e-76
10	Epithelial cells	*DBI*	1.1204	0.877	0.291	8.768e-54	2.025e-49
10	Epithelial cells	*SINHCAF*	1.1102	0.659	0.041	3.815e-233	8.810e-229
10	Epithelial cells	*E2F5*	1.0988	0.725	0.110	2.339e-103	5.401e-99
10	Epithelial cells	*WLS*	1.0976	0.746	0.181	2.400e-60	5.543e-56
10	Epithelial cells	*KRT7*	1.0930	0.543	0.003	0	0
10	Epithelial cells	*CXADR*	1.0917	0.594	0.020	0	0
10	Epithelial cells	*SESTD1*	1.0887	0.696	0.167	4.630e-57	1.069e-52
10	Epithelial cells	*BCAM*	1.0828	0.812	0.119	3.681e-110	8.500e-106
10	Epithelial cells	*RASSF6*	1.0827	0.464	0.001	0	0
10	Epithelial cells	*GUCY1A1*	1.0722	0.638	0.056	9.596e-160	2.216e-155
10	Epithelial cells	*KEF51-p10*	1.0705	0.993	0.994	9.414e-63	2.174e-58
10	Epithelial cells	*GPM6A*	1.0702	0.536	0.113	4.912e-50	1.134e-45
10	Epithelial cells	*NET1*	1.0692	0.804	0.214	8.577e-59	1.981e-54
10	Epithelial cells	*DSTN*	1.0661	0.942	0.562	1.874e-37	4.326e-33
10	Epithelial cells	*MMEL1*	1.0630	0.514	0.001	0	0
10	Epithelial cells	*STON2*	1.0577	0.558	0.033	1.983e-204	4.579e-200
10	Epithelial cells	*FAM13A*	1.0548	0.717	0.189	1.026e-52	2.370e-48
10	Epithelial cells	*RALYL*	1.0520	0.493	0.116	2.376e-38	5.486e-34
10	Epithelial cells	*PTPRF*	1.0511	0.754	0.105	3.644e-116	8.414e-112
10	Epithelial cells	*FBXL17*	1.0494	0.775	0.324	8.439e-34	1.949e-29
10	Epithelial cells	*TM4SF1*	1.0482	0.522	0.064	1.023e-92	2.362e-88
10	Epithelial cells	*STXBP5*	1.0468	0.739	0.274	8.242e-37	1.903e-32
10	Epithelial cells	*KEF51-p01*	1.0395	0.993	0.990	8.771e-56	2.025e-51
10	Epithelial cells	*MAGI3*	1.0394	0.717	0.264	3.756e-33	8.674e-29
10	Epithelial cells	*NAV3*	1.0391	0.493	0.074	7.395e-71	1.708e-66
10	Epithelial cells	*FAM3C*	1.0382	0.783	0.238	5.755e-50	1.329e-45
10	Epithelial cells	*KLHL2*	1.0311	0.638	0.139	1.118e-55	2.582e-51
10	Epithelial cells	*EPB41L5*	1.0292	0.681	0.141	3.008e-66	6.946e-62
10	Epithelial cells	*RUNDC3B*	1.0270	0.420	0.036	1.401e-110	3.234e-106
10	Epithelial cells	*ITM2C*	1.0256	0.826	0.235	3.705e-54	8.555e-50
10	Epithelial cells	*ARID5B*	1.0181	0.899	0.471	3.989e-29	9.211e-25
10	Epithelial cells	*USP53*	1.0164	0.616	0.081	3.068e-102	7.085e-98
10	Epithelial cells	*SLC25A13*	1.0160	0.667	0.052	1.173e-192	2.709e-188

Original cluster	Annotated cell type	Gene	avg_log2FC	pct.1	pct.2	Raw P value	Bonferroni-adjusted P value
10	Epithelial cells	*SSH2*	1.0138	0.688	0.190	1.875e-46	4.329e-42
10	Epithelial cells	*ATOX1*	1.0125	0.797	0.213	2.855e-58	6.593e-54
10	Epithelial cells	*CDH2*	1.0125	0.420	0.021	7.979e-178	1.843e-173
10	Epithelial cells	*GRHL2*	1.0116	0.529	0.003	0	0
10	Epithelial cells	*SPIN1*	1.0105	0.891	0.398	1.863e-39	4.301e-35
10	Epithelial cells	*RIF1*	1.0091	0.710	0.171	2.189e-53	5.056e-49
10	Epithelial cells	*LIMA1*	1.0082	0.696	0.081	5.747e-131	1.327e-126
10	Epithelial cells	*PCGF6*	1.0080	0.587	0.092	1.907e-79	4.403e-75
10	Epithelial cells	*TNFSF10*	1.0076	0.623	0.060	2.781e-141	6.421e-137
10	Epithelial cells	*USP34*	1.0045	0.819	0.436	7.422e-30	1.714e-25
10	Epithelial cells	*TIPARP*	1.0009	0.638	0.335	3.172e-16	7.326e-12
10	Epithelial cells	*GPRIN3*	0.9987	0.543	0.058	1.823e-110	4.209e-106
10	Epithelial cells	*DLG1*	0.9964	0.812	0.431	1.956e-29	4.516e-25
10	Epithelial cells	*FAF1*	0.9946	0.855	0.409	2.036e-31	4.701e-27
10	Epithelial cells	*IGSF5*	0.9946	0.638	0.007	0	0
10	Epithelial cells	*IVNS1ABP*	0.9914	0.746	0.242	1.423e-39	3.286e-35
10	Epithelial cells	*EZR*	0.9884	0.543	0.019	2.190e-306	5.056e-302
10	Epithelial cells	*SOX5*	0.9873	0.877	0.388	3.656e-35	8.443e-31
10	Epithelial cells	*PTBP3*	0.9770	0.746	0.209	1.351e-48	3.120e-44
10	Epithelial cells	*KDM6A*	0.9729	0.812	0.380	8.502e-31	1.963e-26
10	Epithelial cells	*RASSF3*	0.9703	0.674	0.099	3.999e-97	9.234e-93
10	Epithelial cells	*PTPRB*	0.9696	0.449	0.037	2.337e-119	5.397e-115
10	Epithelial cells	*NCAM1*	0.9678	0.616	0.190	2.425e-34	5.599e-30
10	Epithelial cells	*LOC138845366*	0.9668	0.725	0.168	1.611e-59	3.719e-55
10	Epithelial cells	*CTBP2*	0.9662	0.732	0.192	2.886e-53	6.664e-49
10	Epithelial cells	*PPP2R5A*	0.9641	0.746	0.221	3.267e-45	7.544e-41
10	Epithelial cells	*FBXO34*	0.9630	0.623	0.079	1.046e-105	2.415e-101
10	Epithelial cells	*MTF2*	0.9599	0.855	0.343	9.488e-38	2.191e-33
10	Epithelial cells	*NEO1*	0.9510	0.688	0.147	1.305e-61	3.013e-57
10	Epithelial cells	*CTNND2*	0.9500	0.312	0.010	1.797e-186	4.150e-182
10	Epithelial cells	*WWOX*	0.9486	0.804	0.365	2.758e-28	6.370e-24
10	Epithelial cells	*MPZL2*	0.9436	0.601	0.049	1.742e-164	4.023e-160
10	Epithelial cells	*JARID2*	0.9412	0.703	0.175	6.440e-53	1.487e-48
10	Epithelial cells	*CADPS2*	0.9396	0.645	0.120	5.946e-69	1.373e-64
10	Epithelial cells	*YTHDC1*	0.9380	0.870	0.495	9.617e-29	2.221e-24
10	Epithelial cells	*SVBP*	0.9374	0.768	0.323	2.151e-30	4.968e-26
10	Epithelial cells	*LOC103347986*	0.9359	0.609	0.101	5.637e-76	1.302e-71
10	Epithelial cells	*ZNRF3*	0.9306	0.746	0.181	3.592e-59	8.294e-55
10	Epithelial cells	*SIPA1L3*	0.9303	0.638	0.097	8.072e-88	1.864e-83
10	Epithelial cells	*SAMHD1*	0.9294	0.449	0.042	1.147e-106	2.650e-102

Original cluster	Annotated cell type	Gene	avg_log2FC	pct.1	pct.2	Raw P value	Bonferroni-adjusted P value
10	Epithelial cells	*LOC138846446*	0.9259	0.486	0.019	1.488e-253	3.436e-249
10	Epithelial cells	*ZNF704*	0.9254	0.746	0.326	1.491e-25	3.444e-21
10	Epithelial cells	*PKP2*	0.9245	0.464	0.009	0	0
10	Epithelial cells	*CCN1*	0.9235	0.558	0.188	1.144e-28	2.642e-24
10	Epithelial cells	*SAMD12*	0.9231	0.543	0.096	1.135e-61	2.620e-57
10	Epithelial cells	*GABRA4*	0.9227	0.543	0.021	5.544e-286	1.280e-281
10	Epithelial cells	*RERE*	0.9224	0.870	0.447	3.649e-27	8.427e-23
10	Epithelial cells	*PPP1R1A*	0.9220	0.406	0.005	0	0
10	Epithelial cells	*LOC138843459*	0.9218	0.457	0.057	1.916e-80	4.425e-76
10	Epithelial cells	*ENTPD3*	0.9218	0.442	0.014	3.098e-275	7.153e-271
10	Epithelial cells	*FZD1*	0.9201	0.623	0.094	6.151e-86	1.420e-81
10	Epithelial cells	*RASAL2*	0.9195	0.681	0.136	4.421e-67	1.021e-62
10	Epithelial cells	*SH3D19*	0.9156	0.717	0.279	8.394e-30	1.938e-25
10	Epithelial cells	*HOOK3*	0.9144	0.862	0.433	9.085e-27	2.098e-22
10	Epithelial cells	*WEE1*	0.9105	0.616	0.096	2.473e-82	5.711e-78
10	Epithelial cells	*MFSD6*	0.9102	0.565	0.025	1.359e-267	3.138e-263
10	Epithelial cells	*HOXB2*	0.9100	0.710	0.126	8.815e-81	2.036e-76
10	Epithelial cells	*MACF1*	0.9090	0.877	0.479	3.439e-27	7.942e-23
10	Epithelial cells	*MEIS1*	0.9061	0.783	0.324	1.100e-29	2.540e-25
10	Epithelial cells	*DLX6*	0.8989	0.529	0.000	0	0
10	Epithelial cells	*ARRDC4*	0.8974	0.514	0.050	3.471e-118	8.014e-114
10	Epithelial cells	*NIPBL*	0.8957	0.862	0.458	6.954e-27	1.606e-22
10	Epithelial cells	*SGMS1*	0.8860	0.812	0.306	3.263e-35	7.535e-31
10	Epithelial cells	*CXCL1*	0.8854	0.319	0.007	2.059e-257	4.754e-253
10	Epithelial cells	*RETREG1*	0.8841	0.594	0.090	4.660e-81	1.076e-76
10	Epithelial cells	*CD2AP*	0.8791	0.862	0.344	1.037e-34	2.396e-30
10	Epithelial cells	*NEDD4L*	0.8774	0.674	0.120	1.394e-74	3.219e-70
10	Epithelial cells	*CD55*	0.8752	0.790	0.218	4.060e-52	9.376e-48
10	Epithelial cells	*MMD*	0.8744	0.543	0.088	2.516e-70	5.810e-66
10	Epithelial cells	*ARHGAP24*	0.8703	0.514	0.050	1.789e-114	4.132e-110
10	Epithelial cells	*MSX2*	0.8694	0.514	0.002	0	0
10	Epithelial cells	*SLTM*	0.8693	0.848	0.382	5.354e-29	1.236e-24
10	Epithelial cells	*CAMK1D*	0.8690	0.623	0.063	1.072e-134	2.475e-130
10	Epithelial cells	*SBF2*	0.8689	0.870	0.419	4.345e-29	1.003e-24
10	Epithelial cells	*ATP5MK*	0.8676	0.870	0.465	1.945e-25	4.491e-21
10	Epithelial cells	*NEDD9*	0.8640	0.667	0.110	3.312e-82	7.648e-78
10	Epithelial cells	*LOC138846563*	0.8634	0.616	0.135	1.653e-52	3.817e-48
10	Epithelial cells	*ACSS3*	0.8631	0.529	0.122	6.985e-43	1.613e-38
10	Epithelial cells	*LMO7*	0.8619	0.507	0.053	1.663e-106	3.839e-102
10	Epithelial cells	*HOMER2*	0.8618	0.558	0.035	6.704e-192	1.548e-187

Original cluster	Annotated cell type	Gene	avg_log2FC	pct.1	pct.2	Raw P value	Bonferroni-adjusted P value
10	Epithelial cells	*AP1S3*	0.8612	0.486	0.003	0	0
10	Epithelial cells	*GAREM1*	0.8601	0.565	0.075	3.856e-90	8.905e-86
10	Epithelial cells	*GCNT1*	0.8594	0.493	0.009	0	0
10	Epithelial cells	*TMEM131L*	0.8593	0.522	0.115	1.745e-45	4.029e-41
10	Epithelial cells	*FXYD3*	0.8577	0.471	0.001	0	0
10	Epithelial cells	*KIAA0319L*	0.8546	0.493	0.051	9.132e-105	2.109e-100
10	Epithelial cells	*LOC127484232*	0.8544	0.254	0.000	0	0
10	Epithelial cells	*ZFHX3*	0.8541	0.725	0.191	1.108e-47	2.560e-43
10	Epithelial cells	*GRB7*	0.8534	0.543	0.002	0	0
10	Epithelial cells	*EIF4E3*	0.8529	0.775	0.275	2.442e-35	5.639e-31
10	Epithelial cells	*NPAS3*	0.8502	0.725	0.344	8.278e-20	1.912e-15
10	Epithelial cells	*LOC138848390*	0.8495	0.246	0.002	0	0
10	Epithelial cells	*LOC127488638*	0.8441	0.399	0.002	0	0
10	Epithelial cells	*PIK3CB*	0.8431	0.594	0.083	1.219e-89	2.816e-85
10	Epithelial cells	*RP1*	0.8412	0.319	0.003	0	0
10	Epithelial cells	*LOC100354804*	0.8331	0.312	0.022	6.298e-99	1.454e-94
10	Epithelial cells	*IYD*	0.8321	0.435	0.040	1.168e-103	2.697e-99
10	Epithelial cells	*IER3*	0.8303	0.804	0.527	1.710e-12	3.949e-08
10	Epithelial cells	*HOXB6*	0.8292	0.587	0.044	2.131e-170	4.921e-166
10	Epithelial cells	*ARHGEF12*	0.8288	0.761	0.298	1.722e-29	3.977e-25
10	Epithelial cells	*CDKN1C*	0.8254	0.688	0.377	8.687e-14	2.006e-09
10	Epithelial cells	*LOC100354169*	0.8242	0.623	0.155	3.395e-45	7.840e-41
10	Epithelial cells	*ZNF148*	0.8213	0.841	0.404	9.548e-26	2.205e-21
10	Epithelial cells	*CDKL1*	0.8206	0.471	0.024	6.380e-196	1.473e-191
10	Epithelial cells	*KEF51-p07*	0.8183	1.000	0.994	1.729e-47	3.992e-43
10	Epithelial cells	*ABHD17B*	0.8152	0.674	0.151	2.124e-55	4.905e-51
10	Epithelial cells	*MFAP5*	0.8136	0.551	0.048	7.539e-137	1.741e-132
10	Epithelial cells	*MAP4K5*	0.8125	0.667	0.190	2.415e-39	5.576e-35
10	Epithelial cells	*ZCCHC7*	0.8121	0.797	0.402	2.343e-21	5.410e-17
10	Epithelial cells	*LOC108175596*	0.8120	0.543	0.064	7.453e-101	1.721e-96
10	Epithelial cells	*TANC1*	0.8098	0.681	0.166	6.424e-50	1.483e-45
10	Epithelial cells	*BIRC3*	0.8076	0.341	0.021	1.358e-119	3.137e-115
10	Epithelial cells	*CYTIP*	0.8075	0.471	0.030	4.545e-161	1.049e-156
10	Epithelial cells	*HSPB8*	0.8073	0.471	0.031	8.715e-154	2.012e-149
10	Epithelial cells	*EXOC4*	0.8067	0.819	0.425	4.817e-24	1.112e-19
10	Epithelial cells	*RICTOR*	0.8065	0.783	0.342	8.486e-27	1.959e-22
10	Epithelial cells	*IMMP2L*	0.8040	0.841	0.466	5.262e-20	1.215e-15
10	Epithelial cells	*TMEM238*	0.8035	0.551	0.021	6.421e-286	1.483e-281
10	Epithelial cells	*KIF26B*	0.8035	0.652	0.205	5.906e-31	1.364e-26
10	Epithelial cells	*LOC138849793*	0.8029	0.283	0.001	0	0

Original cluster	Annotated cell type	Gene	avg_log2FC	pct.1	pct.2	Raw P value	Bonferroni-adjusted P value
10	Epithelial cells	*ADGRA3*	0.8026	0.594	0.132	8.855e-50	2.045e-45
10	Epithelial cells	*ETV6*	0.8022	0.717	0.179	4.246e-52	9.805e-48
10	Epithelial cells	*LGR4*	0.8016	0.529	0.064	2.810e-93	6.489e-89
10	Epithelial cells	*IGF1R*	0.8006	0.797	0.296	1.005e-30	2.322e-26
10	Epithelial cells	*ARHGAP26*	0.8006	0.558	0.065	1.853e-104	4.279e-100
10	Epithelial cells	*MNAT1*	0.8005	0.645	0.176	2.901e-39	6.698e-35
10	Epithelial cells	*CXXC5*	0.8002	0.746	0.221	6.717e-42	1.551e-37
10	Epithelial cells	*AOPEP*	0.7993	0.855	0.374	1.413e-28	3.262e-24
10	Epithelial cells	*LRIG1*	0.7993	0.558	0.060	3.084e-112	7.122e-108
10	Epithelial cells	*CRIP2*	0.7992	0.587	0.084	3.328e-84	7.684e-80
10	Epithelial cells	*USP25*	0.7978	0.659	0.253	8.875e-24	2.049e-19
10	Epithelial cells	*SUGCT*	0.7976	0.406	0.089	3.660e-35	8.451e-31
10	Epithelial cells	*NFIB*	0.7957	0.768	0.361	2.282e-20	5.271e-16
10	Epithelial cells	*PPP1R9A*	0.7946	0.638	0.190	2.900e-35	6.696e-31
10	Epithelial cells	*LOC127492971*	0.7940	0.384	0.031	2.330e-106	5.380e-102
10	Epithelial cells	*CTNNA1*	0.7934	0.833	0.343	2.812e-30	6.492e-26
10	Epithelial cells	*F11R*	0.7913	0.522	0.016	0	0
10	Epithelial cells	*SATB1*	0.7881	0.580	0.069	5.826e-104	1.345e-99
10	Epithelial cells	*ZNRF2*	0.7875	0.572	0.102	7.252e-66	1.675e-61
10	Epithelial cells	*WNK1*	0.7874	0.761	0.334	2.752e-24	6.356e-20
10	Epithelial cells	*ERBIN*	0.7871	0.862	0.385	3.034e-24	7.007e-20
10	Epithelial cells	*ADAMTSL1*	0.7870	0.623	0.324	7.583e-13	1.751e-08
10	Epithelial cells	*PRRC2C*	0.7870	0.949	0.663	2.387e-15	5.511e-11
10	Epithelial cells	*EYA2*	0.7862	0.536	0.006	0	0
10	Epithelial cells	*CCDC122*	0.7845	0.551	0.161	9.107e-31	2.103e-26
10	Epithelial cells	*ARID1B*	0.7841	0.862	0.504	4.609e-23	1.064e-18
10	Epithelial cells	*TOX3*	0.7797	0.645	0.126	3.917e-61	9.046e-57
10	Epithelial cells	*FAM117B*	0.7770	0.507	0.088	1.356e-59	3.132e-55
10	Epithelial cells	*DAAM1*	0.7758	0.609	0.157	6.951e-41	1.605e-36
10	Epithelial cells	*HIVEP1*	0.7751	0.638	0.090	3.038e-94	7.016e-90
10	Epithelial cells	*LARP4B*	0.7747	0.754	0.175	3.143e-58	7.257e-54
10	Epithelial cells	*LRRC1*	0.7731	0.522	0.032	4.174e-184	9.638e-180
10	Epithelial cells	*FBXL20*	0.7722	0.804	0.360	2.776e-25	6.410e-21
10	Epithelial cells	*PPA1*	0.7713	0.529	0.056	1.796e-109	4.148e-105
10	Epithelial cells	*LRRC8D*	0.7702	0.580	0.061	7.714e-118	1.781e-113
10	Epithelial cells	*CTNND1*	0.7702	0.659	0.160	2.001e-49	4.622e-45
10	Epithelial cells	*PRPF18*	0.7693	0.790	0.381	2.901e-22	6.699e-18
10	Epithelial cells	*ITGB8*	0.7662	0.580	0.070	8.186e-100	1.890e-95
10	Epithelial cells	*KEF51-p11*	0.7654	0.986	0.993	2.010e-43	4.643e-39
10	Epithelial cells	*AGMO*	0.7645	0.283	0.009	5.466e-176	1.262e-171

Original cluster	Annotated cell type	Gene	avg_log2FC	pct.1	pct.2	Raw P value	Bonferroni-adjusted P value
10	Epithelial cells	*CHRM3*	0.7641	0.203	0.016	1.071e-57	2.473e-53
10	Epithelial cells	*IL6ST*	0.7612	0.848	0.361	7.883e-30	1.820e-25
10	Epithelial cells	*LAMA4*	0.7602	0.630	0.201	1.424e-29	3.288e-25
10	Epithelial cells	*PIEZO2*	0.7601	0.312	0.032	7.338e-67	1.694e-62
10	Epithelial cells	*DDX17*	0.7597	0.826	0.395	2.768e-21	6.393e-17
10	Epithelial cells	*FGFR2*	0.7571	0.717	0.167	9.015e-54	2.082e-49
10	Epithelial cells	*TC2N*	0.7556	0.572	0.109	1.920e-58	4.434e-54
10	Epithelial cells	*SMIM22*	0.7545	0.478	0.000	0	0
10	Epithelial cells	*IREB2*	0.7542	0.645	0.154	6.163e-50	1.423e-45
10	Epithelial cells	*SGK1*	0.7541	0.493	0.158	5.665e-24	1.308e-19
10	Epithelial cells	*STRBP*	0.7534	0.623	0.127	4.849e-57	1.120e-52
10	Epithelial cells	*EPB41*	0.7526	0.732	0.254	7.746e-32	1.789e-27
10	Epithelial cells	*FER*	0.7521	0.717	0.283	1.957e-25	4.518e-21
10	Epithelial cells	*CA3*	0.7517	0.246	0.000	0	0
10	Epithelial cells	*PHGDH*	0.7514	0.507	0.035	1.113e-159	2.571e-155
10	Epithelial cells	*HERC4*	0.7502	0.703	0.254	5.564e-29	1.285e-24
10	Epithelial cells	*TRPS1*	0.7480	0.681	0.303	6.209e-18	1.434e-13
10	Epithelial cells	*H1-4*	0.7477	0.580	0.076	1.040e-92	2.402e-88
10	Epithelial cells	*LAMA5*	0.7471	0.522	0.045	6.412e-131	1.481e-126
10	Epithelial cells	*FMO3*	0.7468	0.283	0.000	0	0
10	Epithelial cells	*MYO10*	0.7464	0.478	0.056	1.209e-88	2.792e-84
10	Epithelial cells	*ZNF608*	0.7461	0.449	0.047	2.977e-94	6.875e-90
10	Epithelial cells	*PAX2*	0.7461	0.478	0.001	0	0
10	Epithelial cells	*ARFGEF3*	0.7452	0.355	0.003	0	0
10	Epithelial cells	*MAP3K21*	0.7444	0.449	0.050	2.881e-86	6.653e-82
10	Epithelial cells	*WWC1*	0.7436	0.457	0.003	0	0
10	Epithelial cells	*LOC100348058*	0.7432	0.754	0.267	5.410e-32	1.249e-27
10	Epithelial cells	*CRYAB*	0.7427	0.558	0.073	8.534e-89	1.971e-84
10	Epithelial cells	*LPP*	0.7424	0.797	0.374	1.495e-22	3.453e-18
10	Epithelial cells	*SPATA13*	0.7419	0.507	0.050	3.242e-111	7.487e-107
10	Epithelial cells	*ITPR3*	0.7410	0.500	0.026	2.631e-207	6.076e-203
10	Epithelial cells	*ANKHD1*	0.7406	0.833	0.353	7.357e-28	1.699e-23
10	Epithelial cells	*LOC138844609*	0.7394	0.536	0.098	4.958e-57	1.145e-52
10	Epithelial cells	*RAB3IP*	0.7380	0.558	0.079	6.111e-81	1.411e-76
10	Epithelial cells	*ANXA2*	0.7370	0.732	0.167	9.958e-57	2.300e-52
10	Epithelial cells	*RASEF*	0.7346	0.428	0.008	0	0
10	Epithelial cells	*NCOA2*	0.7337	0.833	0.509	5.516e-15	1.274e-10
10	Epithelial cells	*LOC138843606*	0.7319	0.203	0.001	1.527e-304	3.526e-300
10	Epithelial cells	*FRK*	0.7307	0.399	0.016	2.985e-202	6.893e-198
10	Epithelial cells	*KMT2C*	0.7297	0.848	0.472	5.523e-21	1.275e-16

Original cluster	Annotated cell type	Gene	avg_log2FC	pct.1	pct.2	Raw P value	Bonferroni-adjusted P value
10	Epithelial cells	*ERBB2*	0.7293	0.587	0.081	1.754e-86	4.051e-82
10	Epithelial cells	*C2CD4B*	0.7288	0.283	0.002	0	0
10	Epithelial cells	*EXPH5*	0.7276	0.449	0.014	8.789e-281	2.029e-276
10	Epithelial cells	*LOC103349722*	0.7275	0.761	0.317	7.047e-24	1.627e-19
10	Epithelial cells	*HNF4G*	0.7267	0.341	0.002	0	0
10	Epithelial cells	*RNF24*	0.7264	0.551	0.097	1.360e-61	3.141e-57
10	Epithelial cells	*RAPH1*	0.7258	0.587	0.106	3.663e-63	8.460e-59
10	Epithelial cells	*ARID2*	0.7256	0.775	0.318	4.098e-27	9.462e-23
10	Epithelial cells	*NR6A1*	0.7253	0.442	0.049	1.247e-86	2.879e-82
10	Epithelial cells	*NKAIN3*	0.7249	0.319	0.032	1.566e-68	3.617e-64
10	Epithelial cells	*DOCK7*	0.7240	0.623	0.174	4.728e-36	1.092e-31
10	Epithelial cells	*IQGAP1*	0.7239	0.725	0.211	6.229e-40	1.438e-35
10	Epithelial cells	*LOC138845290*	0.7195	0.645	0.176	7.848e-40	1.812e-35
10	Epithelial cells	*PDCD6IP*	0.7191	0.804	0.325	2.830e-29	6.535e-25
10	Epithelial cells	*SRGAP3*	0.7187	0.565	0.128	5.800e-44	1.339e-39
10	Epithelial cells	*EML5*	0.7186	0.449	0.079	1.224e-50	2.827e-46
10	Epithelial cells	*UBE2E2*	0.7175	0.551	0.157	1.198e-31	2.767e-27
10	Epithelial cells	*PTPN14*	0.7158	0.609	0.103	4.621e-71	1.067e-66
10	Epithelial cells	*TGIF1*	0.7157	0.833	0.304	2.880e-31	6.650e-27
10	Epithelial cells	*ANKRD17*	0.7153	0.783	0.410	1.616e-19	3.732e-15
10	Epithelial cells	*PUM2*	0.7139	0.833	0.477	2.348e-19	5.422e-15
10	Epithelial cells	*CDC42BPA*	0.7137	0.797	0.416	2.374e-19	5.481e-15
10	Epithelial cells	*ADGRL2*	0.7135	0.746	0.246	2.495e-35	5.761e-31
10	Epithelial cells	*IRS2*	0.7134	0.783	0.329	7.707e-24	1.780e-19
10	Epithelial cells	*TAGLN2*	0.7133	0.862	0.407	8.134e-25	1.878e-20
10	Epithelial cells	*NEK7*	0.7132	0.667	0.238	5.511e-29	1.273e-24
10	Epithelial cells	*ID4*	0.7117	0.768	0.193	1.328e-54	3.066e-50
10	Epithelial cells	*C2H4orf19*	0.7112	0.406	0.007	0	0
10	Epithelial cells	*SHTN1*	0.7101	0.486	0.036	7.410e-140	1.711e-135
10	Epithelial cells	*CCDC50*	0.7099	0.804	0.348	2.376e-24	5.486e-20
10	Epithelial cells	*KIF12*	0.7094	0.406	0.000	0	0
10	Epithelial cells	*FNDC3B*	0.7089	0.877	0.541	7.722e-21	1.783e-16
10	Epithelial cells	*BRWD1*	0.7082	0.681	0.227	5.350e-30	1.235e-25
10	Epithelial cells	*RAPGEF2*	0.7081	0.717	0.277	1.245e-25	2.875e-21
10	Epithelial cells	*KLHL8*	0.7060	0.601	0.138	4.956e-46	1.144e-41
10	Epithelial cells	*DHRS3*	0.7054	0.464	0.056	9.114e-82	2.105e-77
10	Epithelial cells	*LRCH1*	0.7028	0.587	0.186	2.781e-27	6.421e-23
10	Epithelial cells	*FREM2*	0.7018	0.406	0.061	1.393e-55	3.217e-51
10	Epithelial cells	*BABAM2*	0.7007	0.790	0.322	2.851e-25	6.584e-21
10	Epithelial cells	*EDRF1*	0.7005	0.529	0.086	8.614e-64	1.989e-59

Original cluster	Annotated cell type	Gene	avg_log2FC	pct.1	pct.2	Raw P value	Bonferroni-adjusted P value
10	Epithelial cells	*RNF151*	0.7001	0.862	0.389	2.071e-23	4.781e-19
10	Epithelial cells	*USP6NL*	0.6992	0.507	0.066	5.428e-83	1.253e-78
10	Epithelial cells	*DOCK1*	0.6988	0.601	0.140	4.040e-47	9.330e-43
10	Epithelial cells	*EXOC6B*	0.6975	0.761	0.477	1.328e-15	3.066e-11
10	Epithelial cells	*LOC127488291*	0.6948	0.370	0.004	0	0
10	Epithelial cells	*VPS13A*	0.6942	0.594	0.188	2.302e-29	5.316e-25
10	Epithelial cells	*ESRP1*	0.6933	0.391	0.000	0	0
10	Epithelial cells	*ABCC4*	0.6924	0.696	0.217	6.057e-34	1.399e-29
10	Epithelial cells	*PAN3*	0.6922	0.790	0.361	1.473e-22	3.401e-18
10	Epithelial cells	*FNIP1*	0.6917	0.725	0.343	3.893e-19	8.990e-15
10	Epithelial cells	*ATXN7L1*	0.6911	0.681	0.184	1.114e-39	2.573e-35
10	Epithelial cells	*ZFHX4*	0.6885	0.580	0.156	1.062e-35	2.452e-31
10	Epithelial cells	*EML4*	0.6884	0.725	0.247	8.028e-31	1.854e-26
10	Epithelial cells	*FRAS1*	0.6881	0.514	0.157	2.512e-26	5.801e-22
10	Epithelial cells	*DENND1B*	0.6875	0.587	0.152	6.572e-39	1.518e-34
10	Epithelial cells	*NIPAL2*	0.6874	0.674	0.160	7.917e-49	1.828e-44
10	Epithelial cells	*RABGAP1*	0.6871	0.754	0.357	2.649e-21	6.118e-17
10	Epithelial cells	*SENP6*	0.6859	0.848	0.516	3.124e-17	7.214e-13
10	Epithelial cells	*ZNF618*	0.6859	0.688	0.194	5.444e-39	1.257e-34
10	Epithelial cells	*EFCAB11*	0.6852	0.529	0.125	9.793e-40	2.261e-35
10	Epithelial cells	*TRAF3*	0.6848	0.507	0.085	1.490e-60	3.441e-56
10	Epithelial cells	*TOM1L2*	0.6845	0.493	0.100	3.967e-45	9.160e-41
10	Epithelial cells	*TRIM44*	0.6831	0.630	0.168	1.006e-39	2.322e-35
10	Epithelial cells	*STIM2*	0.6824	0.609	0.140	3.279e-47	7.573e-43
10	Epithelial cells	*EPB41L4B*	0.6823	0.486	0.013	0	0
10	Epithelial cells	*LOC138847194*	0.6822	0.239	0.002	0	0
10	Epithelial cells	*AOX3*	0.6821	0.275	0.005	3.837e-230	8.861e-226
10	Epithelial cells	*PRRG1*	0.6816	0.464	0.090	2.461e-46	5.683e-42
10	Epithelial cells	*KREMEN1*	0.6814	0.594	0.119	3.519e-55	8.127e-51
10	Epithelial cells	*SRGAP2*	0.6807	0.703	0.300	2.148e-20	4.961e-16
10	Epithelial cells	*LUC7L3*	0.6806	0.783	0.484	3.034e-11	7.006e-07
10	Epithelial cells	*RASSF9*	0.6794	0.428	0.021	3.879e-187	8.957e-183
10	Epithelial cells	*UNC13B*	0.6794	0.457	0.047	3.036e-97	7.012e-93
10	Epithelial cells	*ELL2*	0.6780	0.572	0.149	2.471e-38	5.707e-34
10	Epithelial cells	*PAWR*	0.6779	0.659	0.181	2.195e-38	5.068e-34
10	Epithelial cells	*RNF43*	0.6774	0.391	0.008	0	0
10	Epithelial cells	*NCOA3*	0.6765	0.587	0.117	3.527e-54	8.146e-50
10	Epithelial cells	*ETV5*	0.6758	0.478	0.073	1.342e-64	3.098e-60
10	Epithelial cells	*WNT7A*	0.6748	0.399	0.000	0	0
10	Epithelial cells	*BCAS3*	0.6745	0.783	0.384	4.017e-20	9.275e-16

Original cluster	Annotated cell type	Gene	avg_log2FC	pct.1	pct.2	Raw P value	Bonferroni-adjusted P value
10	Epithelial cells	*C11H12orf75*	0.6741	0.370	0.014	1.473e-197	3.401e-193
10	Epithelial cells	*KIAA1217*	0.6731	0.652	0.177	8.427e-38	1.946e-33
10	Epithelial cells	*CCND3*	0.6721	0.696	0.232	2.407e-29	5.558e-25
10	Epithelial cells	*BSG*	0.6720	0.949	0.707	3.777e-18	8.721e-14
10	Epithelial cells	*AGPAT2*	0.6714	0.464	0.020	1.285e-226	2.968e-222
10	Epithelial cells	*RPL22L1*	0.6712	0.942	0.783	9.571e-16	2.210e-11
10	Epithelial cells	*RAVER2*	0.6707	0.739	0.269	5.954e-28	1.375e-23
10	Epithelial cells	*SETD5*	0.6705	0.783	0.377	2.226e-19	5.140e-15
10	Epithelial cells	*STK38*	0.6702	0.507	0.137	3.709e-30	8.565e-26
10	Epithelial cells	*RSF1*	0.6697	0.891	0.460	5.301e-20	1.224e-15
10	Epithelial cells	*DAG1*	0.6694	0.630	0.146	7.719e-48	1.782e-43
10	Epithelial cells	*ARL4A*	0.6682	0.732	0.289	9.279e-26	2.143e-21
10	Epithelial cells	*UGT8*	0.6680	0.377	0.005	0	0
10	Epithelial cells	*KEF51-p13*	0.6675	0.993	0.986	3.779e-30	8.726e-26
10	Epithelial cells	*CHD6*	0.6672	0.775	0.394	1.995e-19	4.608e-15
10	Epithelial cells	*CEMIP2*	0.6668	0.536	0.139	2.633e-36	6.081e-32
10	Epithelial cells	*ARHGAP8*	0.6667	0.290	0.004	3.611e-285	8.338e-281
10	Epithelial cells	*PDLIM5*	0.6666	0.652	0.234	1.507e-26	3.480e-22
10	Epithelial cells	*ZMYM2*	0.6663	0.667	0.236	4.895e-26	1.130e-21
10	Epithelial cells	*ASXL2*	0.6648	0.659	0.197	7.014e-35	1.620e-30
10	Epithelial cells	*CYB5B*	0.6642	0.471	0.069	1.035e-65	2.391e-61
10	Epithelial cells	*ACSL4*	0.6633	0.428	0.078	7.627e-46	1.761e-41
10	Epithelial cells	*CARMIL1*	0.6624	0.659	0.196	7.661e-33	1.769e-28
10	Epithelial cells	*ARFGEF1*	0.6621	0.768	0.332	1.089e-23	2.514e-19
10	Epithelial cells	*MAK*	0.6620	0.384	0.003	0	0
10	Epithelial cells	*MYO1D*	0.6609	0.449	0.063	1.396e-67	3.223e-63
10	Epithelial cells	*TNPO1*	0.6588	0.710	0.303	1.476e-21	3.409e-17
10	Epithelial cells	*TMEM54*	0.6584	0.457	0.045	4.705e-99	1.087e-94
10	Epithelial cells	*FOXO1*	0.6580	0.732	0.287	1.880e-26	4.342e-22
10	Epithelial cells	*ANKRD28*	0.6574	0.601	0.188	5.954e-29	1.375e-24
10	Epithelial cells	*SLC46A2*	0.6570	0.399	0.001	0	0
10	Epithelial cells	*TXNDC5*	0.6563	0.478	0.061	1.333e-78	3.078e-74
10	Epithelial cells	*TCF12*	0.6563	0.899	0.726	5.013e-14	1.158e-09
10	Epithelial cells	*USP32*	0.6557	0.630	0.241	1.886e-21	4.356e-17
10	Epithelial cells	*RNF39*	0.6552	0.312	0.004	0	0
10	Epithelial cells	*HUWE1*	0.6543	0.775	0.332	9.920e-24	2.291e-19
10	Epithelial cells	*ARHGAP5*	0.6541	0.870	0.507	2.071e-18	4.781e-14
10	Epithelial cells	*OMD*	0.6531	0.377	0.004	0	0
10	Epithelial cells	*TMCC1*	0.6531	0.558	0.129	9.438e-43	2.179e-38
10	Epithelial cells	*MBNL2*	0.6520	0.855	0.493	1.681e-17	3.882e-13

Original cluster	Annotated cell type	Gene	avg_log2FC	pct.1	pct.2	Raw P value	Bonferroni-adjusted P value
10	Epithelial cells	*TPD52*	0.6510	0.514	0.049	5.271e-118	1.217e-113
10	Epithelial cells	*SCAF8*	0.6493	0.783	0.347	1.114e-22	2.573e-18
10	Epithelial cells	*CHD2*	0.6480	0.739	0.327	1.462e-19	3.376e-15
10	Epithelial cells	*PCNX1*	0.6467	0.717	0.245	8.405e-28	1.941e-23
10	Epithelial cells	*CACNA1A*	0.6467	0.304	0.008	8.284e-219	1.913e-214
10	Epithelial cells	*CCDC91*	0.6451	0.601	0.186	4.396e-29	1.015e-24
10	Epithelial cells	*OXR1*	0.6443	0.804	0.459	9.042e-16	2.088e-11
10	Epithelial cells	*RORA*	0.6436	0.913	0.697	5.323e-15	1.229e-10
10	Epithelial cells	*SNX6*	0.6430	0.768	0.366	7.776e-20	1.796e-15
10	Epithelial cells	*HOXB7*	0.6425	0.406	0.017	5.422e-203	1.252e-198
10	Epithelial cells	*RAB25*	0.6415	0.413	0.001	0	0
10	Epithelial cells	*SPIDR*	0.6414	0.761	0.345	5.681e-20	1.312e-15
10	Epithelial cells	*PPP2R2A*	0.6413	0.790	0.388	7.563e-19	1.746e-14
10	Epithelial cells	*AFF1*	0.6412	0.623	0.246	1.446e-19	3.340e-15
10	Epithelial cells	*PTK2*	0.6411	0.688	0.233	6.979e-27	1.612e-22
10	Epithelial cells	*RFX7*	0.6411	0.601	0.233	3.162e-21	7.302e-17
10	Epithelial cells	*AKAP9*	0.6408	0.906	0.544	8.629e-19	1.993e-14
10	Epithelial cells	*LOC108177235*	0.6406	0.920	0.693	7.691e-15	1.776e-10
10	Epithelial cells	*ARHGAP32*	0.6406	0.732	0.305	3.657e-22	8.445e-18
10	Epithelial cells	*LOC103349169*	0.6402	0.761	0.291	2.376e-27	5.488e-23
10	Epithelial cells	*NBEAL1*	0.6396	0.717	0.346	1.596e-17	3.685e-13
10	Epithelial cells	*SLC38A6*	0.6394	0.428	0.072	2.093e-51	4.834e-47
10	Epithelial cells	*TTC39C*	0.6390	0.362	0.043	1.394e-65	3.218e-61
10	Epithelial cells	*ZBTB38*	0.6378	0.616	0.160	1.028e-38	2.375e-34
10	Epithelial cells	*ITGA6*	0.6374	0.420	0.050	2.348e-76	5.423e-72
10	Epithelial cells	*NUP153*	0.6372	0.623	0.197	1.070e-28	2.471e-24
10	Epithelial cells	*BRAF*	0.6370	0.739	0.330	5.488e-21	1.267e-16
10	Epithelial cells	*RALGAPA1*	0.6368	0.841	0.454	9.739e-18	2.249e-13
10	Epithelial cells	*NFIA*	0.6341	0.906	0.631	8.585e-14	1.982e-09
10	Epithelial cells	*SCMH1*	0.6323	0.797	0.367	8.918e-21	2.059e-16
10	Epithelial cells	*DSG2*	0.6320	0.413	0.007	0	0
10	Epithelial cells	*LTBP3*	0.6298	0.609	0.202	1.583e-26	3.654e-22
10	Epithelial cells	*PALLD*	0.6294	0.681	0.369	1.504e-11	3.474e-07
10	Epithelial cells	*TNKS*	0.6291	0.717	0.361	1.942e-15	4.484e-11
10	Epithelial cells	*XPR1*	0.6283	0.551	0.189	2.482e-24	5.732e-20
10	Epithelial cells	*ZFR*	0.6280	0.739	0.290	2.291e-23	5.290e-19
10	Epithelial cells	*SLC35A3*	0.6277	0.442	0.075	2.856e-52	6.596e-48
10	Epithelial cells	*ATP2B1*	0.6275	0.754	0.400	1.489e-16	3.439e-12
10	Epithelial cells	*DDX10*	0.6268	0.732	0.277	4.112e-25	9.496e-21
10	Epithelial cells	*PKN2*	0.6267	0.768	0.399	2.272e-15	5.247e-11

Original cluster	Annotated cell type	Gene	avg_log2FC	pct.1	pct.2	Raw P value	Bonferroni-adjusted P value
10	Epithelial cells	*ETF1*	0.6264	0.587	0.161	2.634e-34	6.083e-30
10	Epithelial cells	*CUX1*	0.6264	0.609	0.162	1.079e-36	2.492e-32
10	Epithelial cells	*SPEN*	0.6262	0.761	0.256	1.462e-30	3.377e-26
10	Epithelial cells	*TBC1D5*	0.6262	0.812	0.524	7.587e-13	1.752e-08
10	Epithelial cells	*OTUD7B*	0.6255	0.565	0.113	1.665e-52	3.845e-48
10	Epithelial cells	*ESR1*	0.6244	0.971	0.780	1.173e-11	2.709e-07
10	Epithelial cells	*CNKSR3*	0.6238	0.471	0.088	1.181e-48	2.726e-44
10	Epithelial cells	*CAMTA1*	0.6233	0.783	0.306	4.947e-26	1.142e-21
10	Epithelial cells	*MERTK*	0.6231	0.442	0.052	8.689e-81	2.006e-76
10	Epithelial cells	*LOC100351413*	0.6224	0.638	0.172	1.851e-37	4.274e-33
10	Epithelial cells	*PALS1*	0.6218	0.616	0.181	1.423e-31	3.286e-27
10	Epithelial cells	*BACH2*	0.6212	0.594	0.194	2.346e-27	5.416e-23
10	Epithelial cells	*SUCO*	0.6192	0.717	0.292	1.800e-22	4.157e-18
10	Epithelial cells	*DHRS7*	0.6190	0.442	0.052	5.277e-80	1.219e-75
10	Epithelial cells	*DIS3L2*	0.6186	0.623	0.227	9.187e-22	2.121e-17
10	Epithelial cells	*SYN2*	0.6179	0.239	0.012	6.639e-101	1.533e-96
10	Epithelial cells	*GPBP1L1*	0.6176	0.725	0.268	5.671e-24	1.310e-19
10	Epithelial cells	*TRIO*	0.6172	0.696	0.262	5.958e-25	1.376e-20
10	Epithelial cells	*MICAL3*	0.6168	0.565	0.141	3.724e-38	8.598e-34
10	Epithelial cells	*RANBP17*	0.6158	0.500	0.095	3.162e-50	7.302e-46
10	Epithelial cells	*SMG1*	0.6151	0.696	0.272	1.122e-23	2.591e-19
10	Epithelial cells	*BBLN*	0.6141	0.493	0.097	6.503e-47	1.502e-42
10	Epithelial cells	*LOC108177273*	0.6136	0.333	0.001	0	0
10	Epithelial cells	*ELF1*	0.6135	0.601	0.232	2.516e-20	5.809e-16
10	Epithelial cells	*LMCD1*	0.6134	0.413	0.047	6.850e-77	1.582e-72
10	Epithelial cells	*ARHGEF5*	0.6118	0.370	0.028	1.836e-104	4.240e-100
10	Epithelial cells	*GPD2*	0.6110	0.442	0.068	7.699e-59	1.778e-54
10	Epithelial cells	*SETBP1*	0.6109	0.768	0.400	1.015e-16	2.343e-12
10	Epithelial cells	*FZD6*	0.6105	0.464	0.066	8.366e-67	1.932e-62
10	Epithelial cells	*SRSF11*	0.6101	0.949	0.634	8.787e-13	2.029e-08
10	Epithelial cells	*GOLGA4*	0.6099	0.659	0.242	1.133e-24	2.616e-20
10	Epithelial cells	*TRA2B*	0.6098	0.884	0.490	4.442e-18	1.026e-13
10	Epithelial cells	*PEX2*	0.6097	0.522	0.136	4.062e-32	9.380e-28
10	Epithelial cells	*CDK6*	0.6088	0.681	0.299	1.502e-18	3.468e-14
10	Epithelial cells	*FZD3*	0.6086	0.413	0.039	5.103e-96	1.178e-91
10	Epithelial cells	*ARHGAP12*	0.6075	0.572	0.160	5.201e-32	1.201e-27
10	Epithelial cells	*MEF2A*	0.6073	0.746	0.334	1.093e-19	2.523e-15
10	Epithelial cells	*HOXB8*	0.6066	0.384	0.003	0	0
10	Epithelial cells	*UVRAG*	0.6066	0.623	0.215	1.233e-24	2.848e-20
10	Epithelial cells	*FNBP1L*	0.6054	0.725	0.270	1.523e-25	3.517e-21

Original cluster	Annotated cell type	Gene	avg_log2FC	pct.1	pct.2	Raw P value	Bonferroni-adjusted P value
10	Epithelial cells	*MAPK8*	0.6053	0.659	0.308	1.788e-16	4.129e-12
10	Epithelial cells	*SOX9*	0.6045	0.384	0.026	5.498e-122	1.270e-117
10	Epithelial cells	*KANK1*	0.6043	0.420	0.075	4.851e-47	1.120e-42
10	Epithelial cells	*GRAMD1C*	0.6029	0.377	0.015	3.858e-188	8.910e-184
10	Epithelial cells	*REL*	0.6022	0.478	0.063	6.927e-77	1.600e-72
10	Epithelial cells	*AVL9*	0.6021	0.594	0.176	1.834e-29	4.235e-25
10	Epithelial cells	*DIAPH3*	0.6021	0.275	0.025	2.589e-68	5.979e-64
10	Epithelial cells	*ICAM1*	0.6018	0.377	0.075	2.829e-36	6.534e-32
10	Epithelial cells	*TPK1*	0.6008	0.457	0.107	1.666e-34	3.848e-30
10	Epithelial cells	*ABLIM1*	0.6001	0.688	0.261	8.895e-24	2.054e-19
10	Epithelial cells	*PARD3B*	0.5991	0.877	0.571	7.838e-16	1.810e-11
10	Epithelial cells	*ADAM28*	0.5988	0.326	0.002	0	0
10	Epithelial cells	*CMTM8*	0.5982	0.449	0.048	1.959e-91	4.524e-87
10	Epithelial cells	*TNIK*	0.5979	0.420	0.065	1.226e-55	2.832e-51
10	Epithelial cells	*LOC138843087*	0.5969	0.341	0.004	0	0
10	Epithelial cells	*TMBIM6*	0.5968	0.906	0.619	2.896e-17	6.687e-13
10	Epithelial cells	*ATP6V0A4*	0.5962	0.297	0.003	0	0
10	Epithelial cells	*ADAMTS18*	0.5962	0.268	0.004	8.734e-281	2.017e-276
10	Epithelial cells	*ANKS1A*	0.5941	0.580	0.162	4.110e-33	9.490e-29
10	Epithelial cells	*SLC38A2*	0.5936	0.601	0.196	9.664e-26	2.232e-21
10	Epithelial cells	*NF1*	0.5933	0.826	0.466	6.150e-15	1.420e-10
10	Epithelial cells	*LOC127487360*	0.5932	0.449	0.115	8.083e-30	1.867e-25
10	Epithelial cells	*MIPOL1*	0.5930	0.558	0.188	2.329e-23	5.378e-19
10	Epithelial cells	*CPD*	0.5923	0.710	0.280	1.811e-22	4.181e-18
10	Epithelial cells	*LOC103349873*	0.5922	0.471	0.081	4.291e-53	9.910e-49
10	Epithelial cells	*PDCD4*	0.5922	0.732	0.325	8.176e-19	1.888e-14
10	Epithelial cells	*ILRUN*	0.5919	0.681	0.218	2.111e-31	4.875e-27
10	Epithelial cells	*LRATD2*	0.5915	0.428	0.034	1.676e-114	3.869e-110
10	Epithelial cells	*MICAL2*	0.5914	0.391	0.016	1.109e-198	2.560e-194
10	Epithelial cells	*CGN*	0.5908	0.413	0.010	4.387e-307	1.013e-302
10	Epithelial cells	*AGRN*	0.5900	0.384	0.049	2.375e-63	5.483e-59
10	Epithelial cells	*BCLAF1*	0.5899	0.884	0.516	2.488e-15	5.745e-11
10	Epithelial cells	*PRKAG2*	0.5895	0.630	0.202	1.318e-28	3.043e-24
10	Epithelial cells	*EVI5*	0.5889	0.717	0.278	2.346e-22	5.419e-18
10	Epithelial cells	*DOK5*	0.5882	0.326	0.002	0	0
10	Epithelial cells	*LARP1B*	0.5876	0.500	0.127	4.479e-32	1.034e-27
10	Epithelial cells	*ANXA4*	0.5870	0.435	0.044	3.705e-93	8.556e-89
10	Epithelial cells	*CTNNAL1*	0.5867	0.486	0.107	3.975e-39	9.179e-35
10	Epithelial cells	*RELCH*	0.5866	0.609	0.195	4.214e-28	9.732e-24
10	Epithelial cells	*DHX9*	0.5864	0.652	0.215	1.790e-27	4.133e-23

Original cluster	Annotated cell type	Gene	avg_log2FC	pct.1	pct.2	Raw P value	Bonferroni-adjusted P value
10	Epithelial cells	*HOOK1*	0.5854	0.457	0.073	6.227e-56	1.438e-51
10	Epithelial cells	*GABPB1*	0.5848	0.645	0.284	3.628e-17	8.377e-13
10	Epithelial cells	*CEP85L*	0.5843	0.609	0.217	2.510e-23	5.796e-19
10	Epithelial cells	*FAR1*	0.5834	0.572	0.156	7.762e-33	1.792e-28
10	Epithelial cells	*POLA1*	0.5830	0.551	0.133	2.073e-38	4.786e-34
10	Epithelial cells	*RBM33*	0.5826	0.761	0.317	6.997e-21	1.616e-16
10	Epithelial cells	*SMIM19*	0.5824	0.652	0.221	1.533e-24	3.540e-20
10	Epithelial cells	*DAPK1*	0.5819	0.471	0.049	5.122e-96	1.183e-91
10	Epithelial cells	*MYO1E*	0.5810	0.703	0.299	6.535e-20	1.509e-15
10	Epithelial cells	*AKAP13*	0.5808	0.580	0.198	1.314e-24	3.035e-20
10	Epithelial cells	*NNT*	0.5807	0.580	0.212	6.040e-20	1.395e-15
10	Epithelial cells	*ITPR1*	0.5806	0.478	0.107	2.096e-38	4.839e-34
10	Epithelial cells	*CDK13*	0.5797	0.826	0.410	1.160e-17	2.679e-13
10	Epithelial cells	*SNRK*	0.5792	0.522	0.123	3.129e-38	7.225e-34
10	Epithelial cells	*ZFYVE16*	0.5789	0.601	0.169	2.712e-33	6.263e-29
10	Epithelial cells	*LSR*	0.5781	0.413	0.026	2.436e-141	5.624e-137
10	Epithelial cells	*PLEC*	0.5777	0.428	0.128	4.483e-20	1.035e-15
10	Epithelial cells	*STAT3*	0.5776	0.812	0.478	1.084e-13	2.504e-09
10	Epithelial cells	*B4GALNT3*	0.5775	0.348	0.003	0	0
10	Epithelial cells	*CNOT6L*	0.5775	0.768	0.376	7.140e-16	1.649e-11
10	Epithelial cells	*TBL1XR1*	0.5762	0.862	0.510	2.880e-16	6.651e-12
10	Epithelial cells	*DENND1A*	0.5762	0.645	0.221	1.774e-26	4.096e-22
10	Epithelial cells	*CCDC14*	0.5757	0.406	0.060	1.355e-55	3.130e-51
10	Epithelial cells	*NRG4*	0.5755	0.601	0.256	6.488e-17	1.498e-12
10	Epithelial cells	*HIVEP2*	0.5746	0.783	0.452	1.837e-14	4.243e-10
10	Epithelial cells	*MSI2*	0.5746	0.659	0.226	1.741e-26	4.021e-22
10	Epithelial cells	*ATP1A1*	0.5728	0.739	0.309	8.639e-19	1.995e-14
10	Epithelial cells	*LSM14A*	0.5726	0.710	0.272	8.866e-24	2.047e-19
10	Epithelial cells	*HTR4*	0.5719	0.159	0.005	4.642e-97	1.072e-92
10	Epithelial cells	*MAP2K4*	0.5718	0.551	0.197	5.995e-21	1.384e-16
10	Epithelial cells	*CEBPG*	0.5716	0.565	0.130	4.044e-42	9.338e-38
10	Epithelial cells	*DYRK1A*	0.5711	0.688	0.253	3.808e-25	8.793e-21
10	Epithelial cells	*HERC2*	0.5697	0.616	0.218	9.840e-23	2.272e-18
10	Epithelial cells	*ST6GAL1*	0.5696	0.399	0.065	9.832e-49	2.270e-44
10	Epithelial cells	*SAMD13*	0.5696	0.413	0.032	4.341e-115	1.002e-110
10	Epithelial cells	*SLC7A1*	0.5688	0.377	0.031	1.794e-101	4.144e-97
10	Epithelial cells	*LOC100344156*	0.5681	0.362	0.014	4.861e-188	1.122e-183
10	Epithelial cells	*S100A14*	0.5678	0.391	0.004	0	0
10	Epithelial cells	*ABCA5*	0.5666	0.754	0.371	6.140e-15	1.418e-10
10	Epithelial cells	*MCPH1*	0.5656	0.645	0.264	9.100e-19	2.101e-14

Original cluster	Annotated cell type	Gene	avg_log2FC	pct.1	pct.2	Raw P value	Bonferroni-adjusted P value
10	Epithelial cells	*KDM3B*	0.5647	0.681	0.187	7.427e-37	1.715e-32
10	Epithelial cells	*ERBB3*	0.5636	0.384	0.012	1.592e-236	3.677e-232
10	Epithelial cells	*GIPC2*	0.5616	0.341	0.012	2.984e-193	6.891e-189
10	Epithelial cells	*PDGFD*	0.5609	0.428	0.177	1.339e-12	3.093e-08
10	Epithelial cells	*POC1B*	0.5606	0.464	0.100	1.315e-38	3.036e-34
10	Epithelial cells	*CSNK1G1*	0.5589	0.551	0.205	1.891e-19	4.367e-15
10	Epithelial cells	*ENOX2*	0.5587	0.435	0.128	8.961e-24	2.069e-19
10	Epithelial cells	*ANKRD65*	0.5581	0.333	0.006	1.091e-299	2.520e-295
10	Epithelial cells	*SUPT3H*	0.5580	0.746	0.399	2.288e-13	5.284e-09
10	Epithelial cells	*GALNT7*	0.5580	0.601	0.202	6.514e-25	1.504e-20
10	Epithelial cells	*ARID1A*	0.5578	0.688	0.212	2.655e-31	6.130e-27
10	Epithelial cells	*MACIR*	0.5577	0.442	0.044	3.763e-94	8.690e-90
10	Epithelial cells	*KTN1*	0.5568	0.942	0.709	7.579e-16	1.750e-11
10	Epithelial cells	*ATG10*	0.5563	0.616	0.196	4.641e-27	1.072e-22
10	Epithelial cells	*CA2*	0.5549	0.254	0.003	5.792e-267	1.337e-262
10	Epithelial cells	*PARP8*	0.5537	0.355	0.070	1.770e-34	4.086e-30
10	Epithelial cells	*EYA1*	0.5526	0.246	0.041	2.709e-30	6.255e-26
10	Epithelial cells	*SMAD6*	0.5526	0.384	0.036	7.586e-89	1.752e-84
10	Epithelial cells	*CEP350*	0.5524	0.652	0.247	4.832e-21	1.116e-16
10	Epithelial cells	*ENDOD1*	0.5518	0.478	0.085	5.736e-51	1.325e-46
10	Epithelial cells	*PRKDC*	0.5517	0.529	0.155	3.184e-26	7.353e-22
10	Epithelial cells	*NCKAP1*	0.5517	0.703	0.274	5.277e-21	1.218e-16
10	Epithelial cells	*IRF6*	0.5515	0.370	0.009	2.696e-279	6.226e-275
10	Epithelial cells	*ARHGEF38*	0.5496	0.297	0.010	2.240e-173	5.174e-169
10	Epithelial cells	*RECQL*	0.5489	0.536	0.167	1.231e-24	2.842e-20
10	Epithelial cells	*CHN2*	0.5480	0.529	0.113	5.551e-44	1.282e-39
10	Epithelial cells	*REV3L*	0.5477	0.812	0.522	6.708e-13	1.549e-08
10	Epithelial cells	*ZMYM4*	0.5475	0.768	0.396	4.296e-16	9.920e-12
10	Epithelial cells	*RBM6*	0.5474	0.732	0.319	1.286e-18	2.969e-14
10	Epithelial cells	*MKLN1*	0.5466	0.623	0.219	7.388e-23	1.706e-18
10	Epithelial cells	*MICU2*	0.5464	0.746	0.346	3.397e-16	7.844e-12
10	Epithelial cells	*ARL15*	0.5457	0.696	0.331	7.257e-16	1.676e-11
10	Epithelial cells	*BDP1*	0.5433	0.659	0.241	3.851e-23	8.892e-19
10	Epithelial cells	*PPP2R2B*	0.5424	0.739	0.448	1.183e-09	2.732e-05
10	Epithelial cells	*LOC127493249*	0.5423	0.290	0.001	0	0
10	Epithelial cells	*GARRE1*	0.5423	0.594	0.175	1.328e-29	3.068e-25
10	Epithelial cells	*SMURF2*	0.5413	0.667	0.238	7.588e-24	1.752e-19
10	Epithelial cells	*PDZD8*	0.5408	0.551	0.149	3.867e-32	8.930e-28
10	Epithelial cells	*PLBD1*	0.5408	0.290	0.031	1.847e-59	4.266e-55
10	Epithelial cells	*KEF51-p08*	0.5408	0.993	0.994	1.963e-24	4.533e-20

Original cluster	Annotated cell type	Gene	avg_log2FC	pct.1	pct.2	Raw P value	Bonferroni-adjusted P value
10	Epithelial cells	*FRS2*	0.5408	0.659	0.248	2.865e-22	6.616e-18
10	Epithelial cells	*OSBPL3*	0.5403	0.399	0.034	7.156e-103	1.652e-98
10	Epithelial cells	*LOC138850734*	0.5402	0.370	0.037	8.100e-81	1.870e-76
10	Epithelial cells	*TNRC6B*	0.5397	0.833	0.493	8.807e-14	2.034e-09
10	Epithelial cells	*RASSF7*	0.5397	0.391	0.004	0	0
10	Epithelial cells	*RAPGEFL1*	0.5383	0.355	0.005	0	0
10	Epithelial cells	*PNN*	0.5380	0.920	0.634	4.950e-11	1.143e-06
10	Epithelial cells	*SNX13*	0.5378	0.580	0.234	6.205e-18	1.433e-13
10	Epithelial cells	*PBX1*	0.5369	0.942	0.813	3.795e-13	8.764e-09
10	Epithelial cells	*DTNB*	0.5366	0.609	0.204	4.597e-25	1.062e-20
10	Epithelial cells	*CERT1*	0.5366	0.594	0.159	5.327e-35	1.230e-30
10	Epithelial cells	*PLEKHF2*	0.5361	0.493	0.109	1.231e-39	2.844e-35
10	Epithelial cells	*STAU2*	0.5357	0.848	0.519	1.914e-11	4.421e-07
10	Epithelial cells	*DERA*	0.5356	0.638	0.182	3.185e-32	7.355e-28
10	Epithelial cells	*LOC100341366*	0.5355	0.507	0.148	8.457e-26	1.953e-21
10	Epithelial cells	*ABCC1*	0.5355	0.391	0.016	7.551e-197	1.744e-192
10	Epithelial cells	*TOP2B*	0.5344	0.739	0.325	3.744e-18	8.645e-14
10	Epithelial cells	*FAM184A*	0.5343	0.377	0.058	3.407e-50	7.868e-46
10	Epithelial cells	*CHMP3*	0.5342	0.696	0.317	2.160e-15	4.989e-11
10	Epithelial cells	*TLE4*	0.5333	0.717	0.372	1.145e-11	2.644e-07
10	Epithelial cells	*WDR26*	0.5333	0.667	0.213	1.327e-27	3.064e-23
10	Epithelial cells	*SH3YL1*	0.5331	0.442	0.058	1.411e-69	3.259e-65
10	Epithelial cells	*MLLT10*	0.5325	0.855	0.471	3.612e-14	8.342e-10
10	Epithelial cells	*SOS1*	0.5324	0.594	0.206	2.496e-23	5.764e-19
10	Epithelial cells	*SLC44A3*	0.5320	0.297	0.010	6.707e-178	1.549e-173
10	Epithelial cells	*MDFIC*	0.5318	0.572	0.263	1.309e-12	3.024e-08
10	Epithelial cells	*KLHL5*	0.5316	0.399	0.028	1.120e-124	2.587e-120
10	Epithelial cells	*AP3B1*	0.5312	0.565	0.148	2.847e-34	6.574e-30
10	Epithelial cells	*CD44*	0.5310	0.435	0.054	4.382e-73	1.012e-68
10	Epithelial cells	*CTDSPL*	0.5309	0.580	0.149	6.616e-36	1.528e-31
10	Epithelial cells	*IKZF2*	0.5309	0.594	0.189	1.476e-26	3.409e-22
10	Epithelial cells	*ZNF292*	0.5295	0.797	0.405	3.773e-13	8.712e-09
10	Epithelial cells	*ZNF644*	0.5291	0.862	0.500	5.458e-12	1.260e-07
10	Epithelial cells	*LEF1*	0.5289	0.478	0.175	3.884e-17	8.970e-13
10	Epithelial cells	*RIMKLB*	0.5275	0.377	0.005	0	0
10	Epithelial cells	*ZNF532*	0.5272	0.594	0.179	1.373e-26	3.171e-22
10	Epithelial cells	*FAM107B*	0.5267	0.674	0.194	1.139e-34	2.631e-30
10	Epithelial cells	*EPS8L2*	0.5264	0.239	0.002	0	0
10	Epithelial cells	*ZNF827*	0.5262	0.478	0.148	7.145e-23	1.650e-18
10	Epithelial cells	*VWA3A*	0.5260	0.239	0.023	3.808e-55	8.794e-51

Original cluster	Annotated cell type	Gene	avg_log2FC	pct.1	pct.2	Raw P value	Bonferroni-adjusted P value
10	Epithelial cells	*PPM1H*	0.5259	0.348	0.032	5.057e-83	1.168e-78
10	Epithelial cells	*SORL1*	0.5254	0.507	0.073	3.342e-70	7.717e-66
10	Epithelial cells	*BCOR*	0.5253	0.442	0.067	8.929e-59	2.062e-54
10	Epithelial cells	*PARD3*	0.5246	0.601	0.217	1.016e-21	2.346e-17
10	Epithelial cells	*KEF51-p04*	0.5244	1.000	0.990	8.377e-25	1.934e-20
10	Epithelial cells	*USP9X*	0.5242	0.652	0.283	4.029e-16	9.303e-12
10	Epithelial cells	*FBXW11*	0.5241	0.681	0.250	2.190e-23	5.058e-19
10	Epithelial cells	*RAI14*	0.5240	0.449	0.057	3.988e-73	9.209e-69
10	Epithelial cells	*WAPL*	0.5240	0.638	0.223	7.450e-23	1.720e-18
10	Epithelial cells	*SPRED2*	0.5239	0.514	0.097	1.318e-50	3.044e-46
10	Epithelial cells	*LOC103352339*	0.5238	0.297	0.000	0	0
10	Epithelial cells	*PIGN*	0.5238	0.478	0.131	5.070e-28	1.171e-23
10	Epithelial cells	*HES1*	0.5235	0.457	0.178	8.071e-15	1.864e-10
10	Epithelial cells	*CRIP1*	0.5232	0.768	0.204	3.418e-47	7.893e-43
10	Epithelial cells	*ACIN1*	0.5229	0.703	0.348	9.868e-12	2.279e-07
10	Epithelial cells	*ATP2C1*	0.5229	0.659	0.222	3.043e-26	7.026e-22
10	Epithelial cells	*NCOA1*	0.5223	0.790	0.454	2.817e-12	6.505e-08
10	Epithelial cells	*CBLB*	0.5220	0.768	0.403	3.283e-16	7.581e-12
10	Epithelial cells	*PRPF39*	0.5214	0.594	0.213	2.256e-21	5.210e-17
10	Epithelial cells	*BIRC6*	0.5214	0.790	0.392	2.071e-15	4.782e-11
10	Epithelial cells	*FARP1*	0.5210	0.638	0.243	6.476e-20	1.495e-15
10	Epithelial cells	*SRSF3*	0.5209	0.884	0.688	1.009e-08	2.330e-04
10	Epithelial cells	*TWF1*	0.5209	0.471	0.121	2.271e-30	5.244e-26
10	Epithelial cells	*ITPR2*	0.5209	0.696	0.261	1.328e-21	3.066e-17
10	Epithelial cells	*DYNC1H1*	0.5202	0.493	0.169	3.420e-18	7.898e-14
10	Epithelial cells	*WDR1*	0.5198	0.551	0.126	1.161e-40	2.680e-36
10	Epithelial cells	*VPS13D*	0.5198	0.565	0.163	2.688e-30	6.208e-26
10	Epithelial cells	*NSD1*	0.5197	0.775	0.340	7.729e-19	1.785e-14
10	Epithelial cells	*ARB2A*	0.5193	0.862	0.550	7.164e-12	1.654e-07
10	Epithelial cells	*RAF1*	0.5191	0.558	0.192	8.710e-22	2.011e-17
10	Epithelial cells	*CCNT2*	0.5183	0.674	0.285	5.835e-18	1.347e-13
10	Epithelial cells	*GOLM1*	0.5180	0.514	0.074	1.259e-70	2.907e-66
10	Epithelial cells	*SPATS2*	0.5179	0.486	0.113	4.041e-36	9.331e-32
10	Epithelial cells	*NBEA*	0.5177	0.841	0.570	8.351e-12	1.929e-07
10	Epithelial cells	*LOC127482730*	0.5177	0.688	0.328	9.372e-15	2.164e-10
10	Epithelial cells	*KANSL1*	0.5170	0.848	0.411	4.341e-16	1.003e-11
10	Epithelial cells	*ATAD2B*	0.5168	0.696	0.335	1.955e-14	4.516e-10
10	Epithelial cells	*JADE3*	0.5167	0.362	0.064	7.699e-41	1.778e-36
10	Epithelial cells	*KEF51-p03*	0.5159	0.913	0.670	5.416e-09	1.251e-04
10	Epithelial cells	*TANC2*	0.5157	0.478	0.184	2.814e-15	6.499e-11

Original cluster	Annotated cell type	Gene	avg_log2FC	pct.1	pct.2	Raw P value	Bonferroni-adjusted P value
10	Epithelial cells	*NFKB1*	0.5156	0.638	0.209	1.717e-26	3.965e-22
10	Epithelial cells	*NRP2*	0.5138	0.442	0.090	1.604e-39	3.704e-35
10	Epithelial cells	*TFCP2L1*	0.5138	0.312	0.000	0	0
10	Epithelial cells	*SIK3*	0.5136	0.659	0.294	8.711e-16	2.011e-11
10	Epithelial cells	*FBLN5*	0.5135	0.623	0.211	5.200e-24	1.201e-19
10	Epithelial cells	*ZBTB44*	0.5130	0.725	0.332	1.388e-16	3.204e-12
10	Epithelial cells	*SRRM2*	0.5130	0.964	0.734	3.714e-08	8.575e-04
10	Epithelial cells	*IGDCC3*	0.5126	0.326	0.014	1.061e-155	2.451e-151
10	Epithelial cells	*LOC100346985*	0.5122	0.370	0.028	1.385e-106	3.198e-102
10	Epithelial cells	*NSD3*	0.5117	0.833	0.504	2.590e-10	5.980e-06
10	Epithelial cells	*GTF2I*	0.5115	0.645	0.227	3.225e-22	7.447e-18
10	Epithelial cells	*SLC20A2*	0.5115	0.399	0.051	2.667e-65	6.158e-61
10	Epithelial cells	*EXOC1*	0.5115	0.406	0.102	3.961e-26	9.146e-22
10	Epithelial cells	*MAP3K3*	0.5110	0.601	0.216	2.216e-21	5.118e-17
10	Epithelial cells	*SLC44A1*	0.5109	0.681	0.243	2.354e-23	5.435e-19
10	Epithelial cells	*FGGY*	0.5100	0.478	0.121	4.150e-31	9.583e-27
10	Epithelial cells	*RSRC1*	0.5097	0.855	0.527	2.314e-11	5.345e-07
10	Epithelial cells	*BIK*	0.5097	0.348	0.002	0	0
10	Epithelial cells	*LOC100358417*	0.5092	0.384	0.078	3.007e-35	6.945e-31
10	Epithelial cells	*CSNK1A1*	0.5092	0.862	0.415	4.781e-16	1.104e-11
10	Epithelial cells	*ITCH*	0.5091	0.681	0.239	1.131e-24	2.611e-20
10	Epithelial cells	*ZZZ3*	0.5089	0.674	0.290	1.927e-16	4.451e-12
10	Epithelial cells	*COP1*	0.5087	0.717	0.332	3.538e-15	8.171e-11
10	Epithelial cells	*SNRNP70*	0.5086	0.688	0.371	2.306e-08	5.325e-04
10	Epithelial cells	*FAM53B*	0.5084	0.355	0.034	2.188e-81	5.052e-77
10	Epithelial cells	*NPNT*	0.5081	0.543	0.176	6.337e-23	1.463e-18
10	Epithelial cells	*UMAD1*	0.5078	0.623	0.281	7.679e-15	1.773e-10
10	Epithelial cells	*CLASP1*	0.5074	0.630	0.241	2.672e-19	6.171e-15
10	Epithelial cells	*HIF1A*	0.5070	0.645	0.187	1.131e-31	2.611e-27
10	Epithelial cells	*SPINK8*	0.5069	0.348	0.007	4.903e-292	1.132e-287
10	Epithelial cells	*GCLC*	0.5065	0.370	0.042	2.506e-69	5.788e-65
10	Epithelial cells	*KAT6A*	0.5058	0.580	0.192	4.080e-22	9.421e-18
10	Epithelial cells	*MAN1A2*	0.5056	0.543	0.177	3.162e-22	7.303e-18
10	Epithelial cells	*DMXL2*	0.5051	0.449	0.052	2.222e-80	5.131e-76
10	Epithelial cells	*TADA2A*	0.5049	0.377	0.054	2.320e-54	5.357e-50
10	Epithelial cells	*GPR176*	0.5046	0.232	0.038	1.138e-28	2.627e-24
10	Epithelial cells	*SLC5A3*	0.5040	0.580	0.156	1.052e-33	2.428e-29
10	Epithelial cells	*VPS13C*	0.5036	0.768	0.369	2.800e-16	6.465e-12
10	Epithelial cells	*AMOTL2*	0.5034	0.435	0.083	8.615e-43	1.989e-38
10	Epithelial cells	*MARCHF7*	0.5033	0.710	0.330	7.757e-16	1.791e-11

Original cluster	Annotated cell type	Gene	avg_log2FC	pct.1	pct.2	Raw P value	Bonferroni-adjusted P value
10	Epithelial cells	*CNOT1*	0.5029	0.609	0.201	1.047e-23	2.417e-19
10	Epithelial cells	*CDYL*	0.5024	0.413	0.089	9.972e-35	2.303e-30
10	Epithelial cells	*PHACTR2*	0.5021	0.667	0.266	1.375e-19	3.176e-15
10	Epithelial cells	*MAP4K3*	0.5015	0.696	0.291	1.120e-18	2.587e-14
10	Epithelial cells	*CENPX*	0.5015	0.572	0.178	7.410e-25	1.711e-20
10	Epithelial cells	*RAD51B*	0.5013	0.609	0.202	1.831e-24	4.229e-20
10	Epithelial cells	*OSBPL9*	0.5012	0.775	0.405	5.447e-15	1.258e-10
10	Epithelial cells	*PIAS2*	0.5009	0.580	0.211	4.626e-20	1.068e-15
10	Epithelial cells	*LOC127490727*	0.5004	0.246	0.029	7.679e-46	1.773e-41
10	Epithelial cells	*RNF170*	0.5002	0.391	0.068	1.795e-43	4.145e-39
10	Epithelial cells	*LOC103344945*	0.5001	0.543	0.161	6.857e-26	1.583e-21
10	Epithelial cells	*N4BP2*	0.5000	0.659	0.270	3.275e-18	7.563e-14
10	Epithelial cells	*PLS3*	0.4999	0.514	0.142	1.354e-28	3.126e-24
10	Epithelial cells	*TCF20*	0.4994	0.536	0.147	2.604e-30	6.013e-26
10	Epithelial cells	*PRPF38B*	0.4990	0.696	0.279	2.916e-19	6.734e-15
10	Epithelial cells	*SOX6*	0.4987	0.804	0.424	6.946e-14	1.604e-09
10	Epithelial cells	*DYNLRB2*	0.4983	0.674	0.222	1.415e-26	3.268e-22
10	Epithelial cells	*KMT5A*	0.4982	0.536	0.157	2.559e-27	5.909e-23
10	Epithelial cells	*NRXN3*	0.4978	0.623	0.313	9.618e-12	2.221e-07
10	Epithelial cells	*UBTF*	0.4965	0.558	0.175	6.331e-25	1.462e-20
10	Epithelial cells	*KYAT3*	0.4964	0.514	0.105	2.899e-45	6.694e-41
10	Epithelial cells	*OCIAD2*	0.4955	0.630	0.267	1.969e-16	4.548e-12
10	Epithelial cells	*TRA2A*	0.4955	0.884	0.561	4.647e-12	1.073e-07
10	Epithelial cells	*SIRT5*	0.4955	0.812	0.372	5.601e-16	1.293e-11
10	Epithelial cells	*TLK1*	0.4952	0.674	0.279	4.162e-18	9.610e-14
10	Epithelial cells	*UBR5*	0.4933	0.717	0.334	1.141e-15	2.635e-11
10	Epithelial cells	*RALGPS1*	0.4924	0.362	0.032	2.938e-88	6.784e-84
10	Epithelial cells	*MBTD1*	0.4918	0.580	0.183	1.410e-24	3.255e-20
10	Epithelial cells	*BIRC2*	0.4916	0.659	0.234	6.415e-22	1.481e-17
10	Epithelial cells	*BLOC1S5*	0.4916	0.486	0.121	1.168e-31	2.697e-27
10	Epithelial cells	*HOXB4*	0.4916	0.348	0.027	5.683e-98	1.312e-93
10	Epithelial cells	*R3HCC1L*	0.4912	0.449	0.116	8.297e-29	1.916e-24
10	Epithelial cells	*ATP11B*	0.4910	0.580	0.184	1.478e-25	3.412e-21
10	Epithelial cells	*C1H9orf85*	0.4910	0.449	0.119	3.228e-27	7.454e-23
10	Epithelial cells	*PDZK1IP1*	0.4905	0.290	0.000	0	0
10	Epithelial cells	*TDRD3*	0.4900	0.638	0.260	2.528e-17	5.837e-13
10	Epithelial cells	*TRDMT1*	0.4898	0.442	0.091	4.063e-39	9.382e-35
10	Epithelial cells	*CADPS*	0.4898	0.167	0.029	5.038e-20	1.163e-15
10	Epithelial cells	*SMAD2*	0.4892	0.558	0.206	2.848e-19	6.576e-15
10	Epithelial cells	*H2AJ*	0.4892	0.775	0.350	4.498e-17	1.039e-12

Original cluster	Annotated cell type	Gene	avg_log2FC	pct.1	pct.2	Raw P value	Bonferroni-adjusted P value
10	Epithelial cells	*B4GALT6*	0.4889	0.333	0.009	1.743e-225	4.024e-221
10	Epithelial cells	*AFDN*	0.4880	0.399	0.034	8.227e-102	1.900e-97
10	Epithelial cells	*DIPK1A*	0.4879	0.355	0.045	3.883e-59	8.967e-55
10	Epithelial cells	*TJP2*	0.4878	0.348	0.042	1.580e-60	3.648e-56
10	Epithelial cells	*PDLIM1*	0.4878	0.420	0.050	2.890e-74	6.673e-70
10	Epithelial cells	*PCMTD1*	0.4876	0.804	0.406	3.243e-14	7.488e-10
10	Epithelial cells	*LUZP1*	0.4876	0.304	0.069	1.602e-24	3.700e-20
10	Epithelial cells	*VWA8*	0.4873	0.522	0.204	6.468e-16	1.494e-11
10	Epithelial cells	*AQP9*	0.4872	0.304	0.002	0	0
10	Epithelial cells	*CHKA*	0.4867	0.348	0.038	6.870e-68	1.586e-63
10	Epithelial cells	*ATP1B1*	0.4863	0.442	0.116	3.037e-26	7.013e-22
10	Epithelial cells	*CHMP4C*	0.4849	0.304	0.001	0	0
10	Epithelial cells	*SEMA6D*	0.4847	0.819	0.527	1.518e-07	3.506e-03
10	Epithelial cells	*PPP2CB*	0.4842	0.630	0.226	5.554e-21	1.282e-16
10	Epithelial cells	*SEC24A*	0.4842	0.420	0.102	4.451e-29	1.028e-24
10	Epithelial cells	*CUL5*	0.4829	0.638	0.246	7.380e-19	1.704e-14
10	Epithelial cells	*ATP8A1*	0.4825	0.413	0.094	2.550e-31	5.890e-27
10	Epithelial cells	*MTUS1*	0.4808	0.580	0.143	1.303e-37	3.008e-33
10	Epithelial cells	*FBXO32*	0.4806	0.543	0.226	1.987e-12	4.588e-08
10	Epithelial cells	*SPPL2A*	0.4802	0.601	0.202	1.580e-23	3.648e-19
10	Epithelial cells	*HEY2*	0.4802	0.348	0.024	3.004e-110	6.936e-106
10	Epithelial cells	*RORC*	0.4801	0.370	0.002	0	0
10	Epithelial cells	*SPTLC3*	0.4801	0.442	0.090	5.289e-39	1.221e-34
10	Epithelial cells	*EFHC2*	0.4796	0.348	0.034	2.526e-77	5.834e-73
10	Epithelial cells	*ANKRD6*	0.4794	0.283	0.012	1.926e-140	4.447e-136
10	Epithelial cells	*PPP1R14B*	0.4791	0.674	0.243	1.591e-21	3.674e-17
10	Epithelial cells	*ST3GAL4*	0.4788	0.428	0.073	1.175e-48	2.713e-44
10	Epithelial cells	*ZNF609*	0.4788	0.667	0.332	3.251e-13	7.506e-09
10	Epithelial cells	*TBC1D8*	0.4784	0.442	0.066	3.283e-60	7.580e-56
10	Epithelial cells	*TRMT9B*	0.4783	0.348	0.029	1.680e-89	3.879e-85
10	Epithelial cells	*UBE2H*	0.4771	0.732	0.310	1.464e-18	3.380e-14
10	Epithelial cells	*ADSS2*	0.4770	0.565	0.172	3.411e-26	7.877e-22
10	Epithelial cells	*NT5C2*	0.4767	0.522	0.136	2.766e-32	6.386e-28
10	Epithelial cells	*SGCG*	0.4764	0.275	0.009	4.081e-170	9.425e-166
10	Epithelial cells	*CACUL1*	0.4759	0.464	0.123	6.946e-28	1.604e-23
10	Epithelial cells	*CREBBP*	0.4756	0.681	0.288	3.198e-17	7.385e-13
10	Epithelial cells	*MTFR1*	0.4752	0.457	0.084	1.475e-46	3.406e-42
10	Epithelial cells	*LCOR*	0.4752	0.580	0.229	2.796e-16	6.456e-12
10	Epithelial cells	*GRAMD2B*	0.4747	0.362	0.049	4.237e-56	9.784e-52
10	Epithelial cells	*LYPD6B*	0.4744	0.254	0.002	7.526e-306	1.738e-301

Original cluster	Annotated cell type	Gene	avg_log2FC	pct.1	pct.2	Raw P value	Bonferroni-adjusted P value
10	Epithelial cells	*TUT4*	0.4742	0.790	0.459	9.998e-10	2.309e-05
10	Epithelial cells	*CD82*	0.4736	0.543	0.134	4.886e-35	1.128e-30
10	Epithelial cells	*MAPKAP1*	0.4736	0.609	0.219	2.384e-21	5.505e-17
10	Epithelial cells	*SVOPL*	0.4730	0.326	0.001	0	0
10	Epithelial cells	*GUCY1B1*	0.4725	0.413	0.052	3.184e-67	7.351e-63
10	Epithelial cells	*LOC100357083*	0.4723	0.725	0.289	2.293e-19	5.295e-15
10	Epithelial cells	*C13H1orf74*	0.4722	0.428	0.094	2.049e-34	4.731e-30
10	Epithelial cells	*SAMTOR*	0.4717	0.529	0.194	1.570e-17	3.625e-13
10	Epithelial cells	*ITSN2*	0.4709	0.638	0.234	1.147e-20	2.649e-16
10	Epithelial cells	*HIBADH*	0.4699	0.688	0.381	3.233e-11	7.467e-07
10	Epithelial cells	*CSNK2A1*	0.4696	0.551	0.154	4.588e-29	1.059e-24
10	Epithelial cells	*NIPAL1*	0.4695	0.254	0.001	0	0
10	Epithelial cells	*GPX1*	0.4692	0.870	0.507	3.593e-12	8.298e-08
10	Epithelial cells	*ARNT2*	0.4686	0.348	0.036	6.260e-72	1.446e-67
10	Epithelial cells	*VPS8*	0.4685	0.493	0.150	1.974e-23	4.559e-19
10	Epithelial cells	*CRELD1*	0.4684	0.246	0.033	1.965e-38	4.538e-34
10	Epithelial cells	*PRICKLE2*	0.4679	0.500	0.143	8.872e-26	2.049e-21
10	Epithelial cells	*C18H16orf87*	0.4672	0.536	0.168	2.068e-23	4.776e-19
10	Epithelial cells	*SRSF6*	0.4672	0.667	0.240	4.116e-20	9.505e-16
10	Epithelial cells	*GRM8*	0.4671	0.261	0.059	5.316e-21	1.227e-16
10	Epithelial cells	*FOXO3*	0.4667	0.703	0.326	3.831e-15	8.846e-11
10	Epithelial cells	*FOXN3*	0.4663	0.645	0.276	2.585e-15	5.970e-11
10	Epithelial cells	*MEMO1*	0.4657	0.449	0.107	1.035e-32	2.390e-28
10	Epithelial cells	*USP24*	0.4655	0.587	0.193	2.322e-24	5.361e-20
10	Epithelial cells	*CEP68*	0.4651	0.551	0.148	2.654e-31	6.129e-27
10	Epithelial cells	*GSR*	0.4649	0.420	0.064	1.157e-55	2.673e-51
10	Epithelial cells	*R3HDM2*	0.4648	0.804	0.458	1.203e-11	2.777e-07
10	Epithelial cells	*ACACA*	0.4646	0.399	0.095	1.797e-28	4.150e-24
10	Epithelial cells	*XRN2*	0.4641	0.688	0.264	1.496e-20	3.455e-16
10	Epithelial cells	*PPM1B*	0.4640	0.703	0.268	1.671e-20	3.859e-16
10	Epithelial cells	*SLC22A23*	0.4639	0.232	0.005	5.867e-176	1.355e-171
10	Epithelial cells	*GATAD2B*	0.4632	0.580	0.148	4.661e-35	1.076e-30
10	Epithelial cells	*RALA*	0.4631	0.587	0.184	1.269e-25	2.931e-21
10	Epithelial cells	*SCAF4*	0.4625	0.442	0.126	2.092e-23	4.831e-19
10	Epithelial cells	*EP300*	0.4608	0.790	0.441	1.649e-12	3.809e-08
10	Epithelial cells	*LAMA3*	0.4605	0.377	0.031	4.356e-100	1.006e-95
10	Epithelial cells	*MSMO1*	0.4599	0.348	0.063	4.893e-37	1.130e-32
10	Epithelial cells	*VGLL4*	0.4597	0.783	0.362	4.848e-16	1.120e-11
10	Epithelial cells	*FOXP1*	0.4597	0.841	0.548	6.799e-12	1.570e-07
10	Epithelial cells	*BAIAP2L1*	0.4589	0.355	0.005	0	0

Original cluster	Annotated cell type	Gene	avg_log2FC	pct.1	pct.2	Raw P value	Bonferroni-adjusted P value
10	Epithelial cells	*TIA1*	0.4585	0.667	0.243	2.230e-21	5.149e-17
10	Epithelial cells	*INSR*	0.4582	0.739	0.344	7.795e-16	1.800e-11
10	Epithelial cells	*PFKFB2*	0.4581	0.319	0.035	4.390e-63	1.014e-58
10	Epithelial cells	*DMXL1*	0.4578	0.681	0.242	1.799e-22	4.154e-18
10	Epithelial cells	*KIT*	0.4570	0.370	0.042	1.712e-67	3.952e-63
10	Epithelial cells	*EPB41L4A*	0.4568	0.268	0.004	4.836e-247	1.117e-242
10	Epithelial cells	*PHF3*	0.4567	0.826	0.503	1.377e-09	3.180e-05
10	Epithelial cells	*LOC138848813*	0.4563	0.754	0.480	8.749e-09	2.020e-04
10	Epithelial cells	*TAOK1*	0.4563	0.703	0.349	2.484e-12	5.736e-08
10	Epithelial cells	*MYO5B*	0.4561	0.355	0.009	3.564e-248	8.230e-244
10	Epithelial cells	*MED13*	0.4559	0.761	0.397	2.043e-13	4.718e-09
10	Epithelial cells	*PPP6R3*	0.4558	0.587	0.219	3.759e-19	8.680e-15
10	Epithelial cells	*LOC127490713*	0.4556	0.341	0.004	0	0
10	Epithelial cells	*LOC138849703*	0.4550	0.312	0.044	1.947e-46	4.496e-42
10	Epithelial cells	*SYPL1*	0.4518	0.652	0.219	7.078e-24	1.635e-19
10	Epithelial cells	*CAND1*	0.4516	0.580	0.219	7.949e-18	1.836e-13
10	Epithelial cells	*OSBPL10*	0.4508	0.283	0.073	5.901e-18	1.363e-13
10	Epithelial cells	*MTHFD1L*	0.4506	0.406	0.096	2.160e-29	4.989e-25
10	Epithelial cells	*RPS6KA5*	0.4504	0.601	0.259	1.602e-14	3.699e-10
10	Epithelial cells	*TLE3*	0.4504	0.399	0.040	4.954e-85	1.144e-80
10	Epithelial cells	*LOC100358013*	0.4502	0.449	0.100	1.246e-34	2.877e-30
10	Epithelial cells	*HEG1*	0.4500	0.319	0.068	1.468e-27	3.391e-23
10	Epithelial cells	*CAST*	0.4495	0.688	0.258	2.040e-20	4.711e-16
10	Epithelial cells	*BRD8*	0.4492	0.659	0.277	9.957e-15	2.299e-10
10	Epithelial cells	*PLPP1*	0.4488	0.536	0.193	6.695e-19	1.546e-14
10	Epithelial cells	*F3*	0.4487	0.239	0.040	2.001e-29	4.622e-25
10	Epithelial cells	*USP8*	0.4487	0.717	0.363	1.798e-12	4.153e-08
10	Epithelial cells	*RASA1*	0.4485	0.681	0.290	3.723e-17	8.598e-13
10	Epithelial cells	*ZNF711*	0.4481	0.667	0.243	4.286e-21	9.897e-17
10	Epithelial cells	*NDFIP2*	0.4472	0.623	0.252	7.070e-17	1.632e-12
10	Epithelial cells	*DBF4*	0.4468	0.500	0.197	3.588e-14	8.285e-10
10	Epithelial cells	*SSBP2*	0.4459	0.797	0.447	4.332e-12	1.000e-07
10	Epithelial cells	*RRAS2*	0.4457	0.413	0.101	7.637e-29	1.764e-24
10	Epithelial cells	*WRN*	0.4455	0.551	0.217	3.403e-16	7.858e-12
10	Epithelial cells	*USP42*	0.4454	0.384	0.092	2.104e-27	4.858e-23
10	Epithelial cells	*WWC2*	0.4454	0.601	0.285	9.292e-13	2.146e-08
10	Epithelial cells	*GPX2*	0.4450	0.232	0.001	0	0
10	Epithelial cells	*IDH2*	0.4450	0.536	0.156	1.704e-26	3.934e-22
10	Epithelial cells	*CLIP1*	0.4447	0.514	0.135	7.809e-31	1.803e-26
10	Epithelial cells	*SLC9B2*	0.4443	0.406	0.092	1.121e-31	2.588e-27

Original cluster	Annotated cell type	Gene	avg_log2FC	pct.1	pct.2	Raw P value	Bonferroni-adjusted P value
10	Epithelial cells	*ALPK1*	0.4440	0.261	0.038	1.660e-37	3.834e-33
10	Epithelial cells	*BPTF*	0.4439	0.833	0.456	6.196e-13	1.431e-08
10	Epithelial cells	*GPAT4*	0.4439	0.428	0.109	1.062e-26	2.453e-22
10	Epithelial cells	*GOLGB1*	0.4438	0.754	0.411	5.198e-13	1.200e-08
10	Epithelial cells	*LOC103345230*	0.4438	0.514	0.186	6.133e-18	1.416e-13
10	Epithelial cells	*RIMS2*	0.4433	0.196	0.007	2.357e-104	5.443e-100
10	Epithelial cells	*CYB561*	0.4423	0.362	0.035	4.196e-81	9.690e-77
10	Epithelial cells	*REPS1*	0.4420	0.514	0.167	1.758e-21	4.060e-17
10	Epithelial cells	*PTBP1*	0.4419	0.551	0.182	6.013e-21	1.388e-16
10	Epithelial cells	*PBX3*	0.4418	0.522	0.217	1.168e-13	2.698e-09
10	Epithelial cells	*KDM3A*	0.4418	0.442	0.110	1.378e-28	3.183e-24
10	Epithelial cells	*ABLIM3*	0.4417	0.275	0.034	3.081e-48	7.114e-44
10	Epithelial cells	*ARIH1*	0.4398	0.630	0.268	1.576e-16	3.639e-12
10	Epithelial cells	*MARCHF3*	0.4396	0.326	0.073	3.790e-27	8.753e-23
10	Epithelial cells	*SKIL*	0.4396	0.536	0.160	1.528e-26	3.529e-22
10	Epithelial cells	*SLC43A1*	0.4395	0.428	0.081	4.071e-41	9.400e-37
10	Epithelial cells	*ST14*	0.4387	0.275	0.002	0	0
10	Epithelial cells	*SKAP2*	0.4384	0.362	0.041	6.347e-69	1.466e-64
10	Epithelial cells	*DIAPH2*	0.4383	0.761	0.415	2.531e-12	5.845e-08
10	Epithelial cells	*SCAPER*	0.4381	0.645	0.238	7.006e-20	1.618e-15
10	Epithelial cells	*UBA6*	0.4380	0.500	0.187	1.517e-15	3.503e-11
10	Epithelial cells	*CDC42EP3*	0.4378	0.384	0.039	9.650e-81	2.228e-76
10	Epithelial cells	*CDCA7L*	0.4362	0.362	0.048	3.588e-57	8.285e-53
10	Epithelial cells	*NHS*	0.4361	0.362	0.104	1.073e-19	2.477e-15
10	Epithelial cells	*SLCO2A1*	0.4358	0.268	0.005	1.272e-219	2.937e-215
10	Epithelial cells	*PKP3*	0.4358	0.246	0.001	0	0
10	Epithelial cells	*FBXO7*	0.4354	0.370	0.062	1.296e-43	2.993e-39
10	Epithelial cells	*HOOK2*	0.4349	0.370	0.064	8.305e-41	1.918e-36
10	Epithelial cells	*DDR1*	0.4348	0.377	0.043	7.212e-70	1.665e-65
10	Epithelial cells	*TSTD1*	0.4344	0.355	0.028	3.543e-97	8.181e-93
10	Epithelial cells	*ZC3H6*	0.4339	0.594	0.223	2.129e-18	4.917e-14
10	Epithelial cells	*ESYT2*	0.4339	0.616	0.231	2.787e-20	6.436e-16
10	Epithelial cells	*TMEM205*	0.4337	0.638	0.228	5.042e-20	1.164e-15
10	Epithelial cells	*CRAMP1*	0.4332	0.457	0.124	3.179e-26	7.340e-22
10	Epithelial cells	*PEAK1*	0.4332	0.514	0.193	5.094e-17	1.176e-12
10	Epithelial cells	*STT3B*	0.4330	0.587	0.227	4.948e-17	1.142e-12
10	Epithelial cells	*EIF3A*	0.4330	0.688	0.310	1.631e-15	3.766e-11
10	Epithelial cells	*BRWD3*	0.4316	0.399	0.120	1.156e-19	2.669e-15
10	Epithelial cells	*LOC100355470*	0.4315	0.688	0.261	3.933e-20	9.083e-16
10	Epithelial cells	*CCDC171*	0.4313	0.312	0.061	6.051e-30	1.397e-25

Original cluster	Annotated cell type	Gene	avg_log2FC	pct.1	pct.2	Raw P value	Bonferroni-adjusted P value
10	Epithelial cells	*RNF152*	0.4309	0.326	0.028	6.625e-84	1.530e-79
10	Epithelial cells	*LOC100356842*	0.4307	0.551	0.219	3.453e-16	7.975e-12
10	Epithelial cells	*TTC17*	0.4304	0.638	0.260	2.536e-16	5.856e-12
10	Epithelial cells	*PTPN3*	0.4304	0.341	0.019	8.063e-130	1.862e-125
10	Epithelial cells	*NUCB2*	0.4302	0.681	0.268	2.991e-18	6.907e-14
10	Epithelial cells	*ATXN1*	0.4300	0.841	0.516	5.227e-11	1.207e-06
10	Epithelial cells	*CTSO*	0.4299	0.420	0.095	1.049e-32	2.423e-28
10	Epithelial cells	*PHLPP1*	0.4296	0.493	0.143	4.642e-25	1.072e-20
10	Epithelial cells	*ATP2A2*	0.4288	0.536	0.183	2.923e-19	6.749e-15
10	Epithelial cells	*GABRB3*	0.4285	0.167	0.007	2.463e-80	5.687e-76
10	Epithelial cells	*PLXNB1*	0.4281	0.297	0.019	3.189e-101	7.364e-97
10	Epithelial cells	*VEZF1*	0.4275	0.732	0.316	8.909e-16	2.057e-11
10	Epithelial cells	*ASCC3*	0.4272	0.587	0.247	3.430e-15	7.921e-11
10	Epithelial cells	*NXNL2*	0.4272	0.326	0.008	2.593e-234	5.988e-230
10	Epithelial cells	*KCMF1*	0.4271	0.594	0.217	1.749e-19	4.039e-15
10	Epithelial cells	*SLC44A4*	0.4263	0.312	0.005	3.659e-299	8.449e-295
10	Epithelial cells	*LOC100345355*	0.4263	0.804	0.416	1.997e-11	4.611e-07
10	Epithelial cells	*KIF13A*	0.4262	0.667	0.261	4.219e-18	9.743e-14
10	Epithelial cells	*CASP10*	0.4245	0.254	0.030	1.096e-46	2.530e-42
10	Epithelial cells	*MOB3B*	0.4239	0.507	0.132	4.959e-30	1.145e-25
10	Epithelial cells	*POLR2D*	0.4237	0.507	0.148	2.048e-25	4.729e-21
10	Epithelial cells	*KMT2E*	0.4235	0.949	0.650	1.989e-11	4.594e-07
10	Epithelial cells	*EWSR1*	0.4226	0.761	0.478	2.576e-08	5.949e-04
10	Epithelial cells	*GBE1*	0.4223	0.659	0.329	4.292e-11	9.911e-07
10	Epithelial cells	*NIM1K*	0.4221	0.210	0.027	7.365e-35	1.701e-30
10	Epithelial cells	*AIG1*	0.4218	0.812	0.514	6.143e-09	1.419e-04
10	Epithelial cells	*FCHO2*	0.4216	0.601	0.241	9.432e-15	2.178e-10
10	Epithelial cells	*CD46*	0.4214	0.283	0.028	1.662e-61	3.837e-57
10	Epithelial cells	*MAST2*	0.4204	0.370	0.066	5.189e-40	1.198e-35
10	Epithelial cells	*HNRNPL*	0.4203	0.696	0.299	7.214e-15	1.666e-10
10	Epithelial cells	*ATP7B*	0.4201	0.341	0.019	9.021e-131	2.083e-126
10	Epithelial cells	*HNF1B*	0.4201	0.290	0.000	0	0
10	Epithelial cells	*LMO2*	0.4197	0.420	0.050	6.827e-73	1.576e-68
10	Epithelial cells	*QRICH1*	0.4197	0.449	0.092	1.057e-38	2.440e-34
10	Epithelial cells	*CLINT1*	0.4197	0.674	0.255	7.514e-20	1.735e-15
10	Epithelial cells	*MBD5*	0.4195	0.783	0.502	4.728e-08	1.092e-03
10	Epithelial cells	*WWTR1*	0.4194	0.630	0.335	3.102e-09	7.164e-05
10	Epithelial cells	*CMBL*	0.4190	0.304	0.019	7.113e-106	1.643e-101
10	Epithelial cells	*SF3B1*	0.4190	0.884	0.546	1.402e-06	3.237e-02
10	Epithelial cells	*PLP2*	0.4189	0.514	0.126	1.058e-33	2.443e-29

Original cluster	Annotated cell type	Gene	avg_log2FC	pct.1	pct.2	Raw P value	Bonferroni-adjusted P value
10	Epithelial cells	*LOC138845125*	0.4187	0.406	0.100	2.190e-27	5.057e-23
10	Epithelial cells	*FGF12*	0.4186	0.188	0.026	5.704e-30	1.317e-25
10	Epithelial cells	*CLTRN*	0.4178	0.312	0.039	2.403e-52	5.549e-48
10	Epithelial cells	*MYO9A*	0.4174	0.659	0.301	2.879e-14	6.648e-10
10	Epithelial cells	*TASOR*	0.4173	0.645	0.253	5.809e-18	1.342e-13
10	Epithelial cells	*SETD2*	0.4171	0.616	0.254	9.855e-16	2.276e-11
10	Epithelial cells	*UBE2E3*	0.4167	0.725	0.327	1.495e-14	3.453e-10
10	Epithelial cells	*SLMAP*	0.4156	0.609	0.231	9.149e-19	2.113e-14
10	Epithelial cells	*EML2*	0.4152	0.391	0.073	1.197e-38	2.765e-34
10	Epithelial cells	*MBD2*	0.4148	0.587	0.179	3.021e-26	6.977e-22
10	Epithelial cells	*ZFAND4*	0.4142	0.304	0.004	5.231e-299	1.208e-294
10	Epithelial cells	*RAPGEF6*	0.4142	0.659	0.273	7.552e-17	1.744e-12
10	Epithelial cells	*IL1R1*	0.4140	0.304	0.021	6.079e-94	1.404e-89
10	Epithelial cells	*DCAF8*	0.4139	0.804	0.423	4.677e-11	1.080e-06
10	Epithelial cells	*RALGAPA2*	0.4134	0.529	0.191	9.890e-18	2.284e-13
10	Epithelial cells	*ABI1*	0.4131	0.645	0.278	4.003e-15	9.243e-11
10	Epithelial cells	*ZNF277*	0.4131	0.855	0.556	1.432e-09	3.306e-05
10	Epithelial cells	*LRP6*	0.4131	0.768	0.328	3.765e-17	8.694e-13
10	Epithelial cells	*PODXL*	0.4130	0.377	0.031	2.161e-97	4.989e-93
10	Epithelial cells	*KIF21A*	0.4129	0.362	0.063	3.097e-40	7.152e-36
10	Epithelial cells	*SP100*	0.4127	0.326	0.023	1.449e-100	3.346e-96
10	Epithelial cells	*MGA*	0.4127	0.616	0.282	7.452e-11	1.721e-06
10	Epithelial cells	*LOC100340719*	0.4126	0.826	0.431	1.174e-13	2.710e-09
10	Epithelial cells	*SMS*	0.4124	0.638	0.210	7.211e-24	1.665e-19
10	Epithelial cells	*MAL*	0.4123	0.290	0.010	4.638e-167	1.071e-162
10	Epithelial cells	*PDS5A*	0.4121	0.609	0.211	2.189e-21	5.056e-17
10	Epithelial cells	*XRCC4*	0.4117	0.529	0.177	9.682e-21	2.236e-16
10	Epithelial cells	*MYCBP2*	0.4117	0.746	0.453	4.842e-07	1.118e-02
10	Epithelial cells	*NINJ1*	0.4115	0.384	0.062	6.498e-47	1.501e-42
10	Epithelial cells	*LOC138850492*	0.4110	0.558	0.214	2.449e-16	5.656e-12
10	Epithelial cells	*USP40*	0.4108	0.493	0.144	2.168e-24	5.007e-20
10	Epithelial cells	*NPEPPS*	0.4107	0.681	0.282	4.910e-15	1.134e-10
10	Epithelial cells	*MGAT5*	0.4106	0.551	0.182	1.374e-21	3.172e-17
10	Epithelial cells	*IHH*	0.4106	0.138	0.000	2.356e-275	5.440e-271
10	Epithelial cells	*GPRC5C*	0.4103	0.420	0.083	3.605e-39	8.325e-35
10	Epithelial cells	*COBLL1*	0.4098	0.623	0.266	2.535e-14	5.854e-10
10	Epithelial cells	*MAML3*	0.4089	0.565	0.237	1.859e-14	4.293e-10
10	Epithelial cells	*TMEM45B*	0.4088	0.225	0.012	2.001e-87	4.620e-83
10	Epithelial cells	*DCP1A*	0.4087	0.543	0.163	2.765e-25	6.384e-21
10	Epithelial cells	*ALDH1A3*	0.4085	0.406	0.134	1.523e-16	3.518e-12

Original cluster	Annotated cell type	Gene	avg_log2FC	pct.1	pct.2	Raw P value	Bonferroni-adjusted P value
10	Epithelial cells	*TNKS2*	0.4082	0.587	0.211	8.170e-19	1.887e-14
10	Epithelial cells	*TMEM265*	0.4080	0.283	0.008	7.421e-195	1.714e-190
10	Epithelial cells	*MARVELD3*	0.4076	0.232	0.002	1.607e-269	3.712e-265
10	Epithelial cells	*HDGFL3*	0.4076	0.594	0.305	5.257e-09	1.214e-04
10	Epithelial cells	*PIK3C2A*	0.4075	0.551	0.211	7.565e-16	1.747e-11
10	Epithelial cells	*PARN*	0.4074	0.449	0.119	1.969e-26	4.548e-22
10	Epithelial cells	*COBL*	0.4071	0.326	0.005	0	0
10	Epithelial cells	*CDCP1*	0.4071	0.297	0.002	0	0
10	Epithelial cells	*OFD1*	0.4064	0.420	0.177	8.996e-10	2.077e-05
10	Epithelial cells	*EEF1AKMT2*	0.4061	0.623	0.213	4.861e-22	1.122e-17
10	Epithelial cells	*PRIM2*	0.4059	0.486	0.136	1.842e-25	4.252e-21
10	Epithelial cells	*PSD3*	0.4058	0.623	0.274	9.155e-14	2.114e-09
10	Epithelial cells	*LOC127490345*	0.4048	0.304	0.001	0	0
10	Epithelial cells	*TASP1*	0.4048	0.659	0.305	1.536e-13	3.547e-09
10	Epithelial cells	*SWAP70*	0.4045	0.464	0.133	3.636e-24	8.395e-20
10	Epithelial cells	*HACD2*	0.4045	0.558	0.182	2.274e-22	5.251e-18
10	Epithelial cells	*ZFC3H1*	0.4043	0.609	0.227	1.779e-17	4.107e-13
10	Epithelial cells	*MGST1*	0.4041	0.319	0.026	3.790e-85	8.752e-81
10	Epithelial cells	*CCNDBP1*	0.4041	0.500	0.117	3.327e-35	7.683e-31
10	Epithelial cells	*EHD4*	0.4040	0.348	0.064	4.255e-37	9.825e-33
10	Epithelial cells	*LOC138843958*	0.4040	0.471	0.109	5.562e-34	1.284e-29
10	Epithelial cells	*FBXL4*	0.4038	0.543	0.192	1.189e-19	2.745e-15
10	Epithelial cells	*ZNF654*	0.4038	0.674	0.332	8.485e-11	1.959e-06
10	Epithelial cells	*CNOT2*	0.4037	0.696	0.354	1.201e-11	2.774e-07
10	Epithelial cells	*CCDC186*	0.4037	0.587	0.195	3.405e-22	7.864e-18
10	Epithelial cells	*KRT10*	0.4036	0.797	0.449	1.424e-08	3.288e-04
10	Epithelial cells	*PSME1*	0.4035	0.630	0.230	2.364e-19	5.459e-15
10	Epithelial cells	*ZNF385B*	0.4029	0.246	0.024	3.070e-56	7.090e-52
10	Epithelial cells	*HS2ST1*	0.4028	0.601	0.252	1.642e-14	3.792e-10
10	Epithelial cells	*IL10RB*	0.4027	0.290	0.036	5.275e-50	1.218e-45
10	Epithelial cells	*LOC127492624*	0.4026	0.304	0.000	0	0
10	Epithelial cells	*STRN3*	0.4023	0.710	0.319	4.788e-15	1.106e-10
10	Epithelial cells	*TTBK2*	0.4022	0.507	0.150	1.645e-24	3.799e-20
10	Epithelial cells	*AFTPH*	0.4021	0.486	0.144	3.876e-23	8.951e-19
10	Epithelial cells	*USP47*	0.4021	0.768	0.434	1.214e-10	2.804e-06
10	Epithelial cells	*NRF1*	0.4018	0.486	0.144	2.522e-23	5.823e-19
10	Epithelial cells	*SUCLG2*	0.4013	0.609	0.196	5.430e-24	1.254e-19
10	Epithelial cells	*LOC127487832*	0.4013	0.138	0.028	9.034e-14	2.086e-09
10	Epithelial cells	*FBXW7*	0.4011	0.630	0.223	8.393e-23	1.938e-18
10	Epithelial cells	*MAL2*	0.4011	0.312	0.004	0	0

Original cluster	Annotated cell type	Gene	avg_log2FC	pct.1	pct.2	Raw P value	Bonferroni-adjusted P value
10	Epithelial cells	*CABLES1*	0.4007	0.413	0.102	3.676e-27	8.489e-23
10	Epithelial cells	*KEF51-p12*	0.4002	0.971	0.945	3.596e-12	8.304e-08
10	Epithelial cells	*PLEKHA7*	0.4000	0.275	0.006	2.754e-218	6.359e-214
10	Epithelial cells	*ARMH3*	0.3996	0.420	0.121	5.184e-22	1.197e-17
10	Epithelial cells	*ARHGAP21*	0.3992	0.406	0.116	6.926e-22	1.599e-17
10	Epithelial cells	*ARGLU1*	0.3988	0.884	0.572	5.899e-07	1.362e-02
10	Epithelial cells	*CNOT6*	0.3983	0.486	0.120	4.032e-31	9.310e-27
10	Epithelial cells	*APRT*	0.3978	0.623	0.184	4.738e-28	1.094e-23
10	Epithelial cells	*MLLT6*	0.3978	0.413	0.084	3.639e-36	8.402e-32
10	Epithelial cells	*BCL11A*	0.3971	0.355	0.048	2.712e-54	6.263e-50
10	Epithelial cells	*FAM168B*	0.3968	0.587	0.182	2.036e-24	4.701e-20
10	Epithelial cells	*STX3*	0.3965	0.217	0.013	3.253e-80	7.512e-76
10	Epithelial cells	*METTL15*	0.3962	0.616	0.236	4.811e-18	1.111e-13
10	Epithelial cells	*RNLS*	0.3962	0.326	0.083	7.911e-22	1.827e-17
10	Epithelial cells	*NUDT14*	0.3961	0.391	0.070	6.563e-42	1.515e-37
10	Epithelial cells	*CRY1*	0.3960	0.464	0.134	6.603e-24	1.525e-19
10	Epithelial cells	*RAD50*	0.3954	0.587	0.199	2.796e-20	6.456e-16
10	Epithelial cells	*FAM204A*	0.3954	0.522	0.171	2.920e-20	6.742e-16
10	Epithelial cells	*IL6R*	0.3952	0.341	0.044	3.739e-54	8.635e-50
10	Epithelial cells	*POU2F1*	0.3948	0.609	0.268	2.991e-13	6.906e-09
10	Epithelial cells	*EHMT1*	0.3948	0.551	0.190	1.808e-19	4.174e-15
10	Epithelial cells	*LY6E*	0.3942	0.601	0.233	6.678e-17	1.542e-12
10	Epithelial cells	*PKIB*	0.3941	0.297	0.017	8.357e-113	1.930e-108
10	Epithelial cells	*CCDC66*	0.3938	0.616	0.256	6.145e-14	1.419e-09
10	Epithelial cells	*ELF2*	0.3937	0.725	0.396	1.163e-09	2.686e-05
10	Epithelial cells	*HELZ*	0.3934	0.645	0.298	3.006e-12	6.942e-08
10	Epithelial cells	*HBP1*	0.3934	0.717	0.364	9.948e-12	2.297e-07
10	Epithelial cells	*HNRNPC*	0.3931	0.804	0.540	2.232e-08	5.154e-04
10	Epithelial cells	*NECTIN2*	0.3929	0.428	0.070	7.869e-50	1.817e-45
10	Epithelial cells	*CABCOCO1*	0.3928	0.268	0.038	1.496e-38	3.454e-34
10	Epithelial cells	*RIN2*	0.3928	0.609	0.239	1.684e-15	3.888e-11
10	Epithelial cells	*KDM5A*	0.3925	0.710	0.321	5.561e-15	1.284e-10
10	Epithelial cells	*SLC25A10*	0.3924	0.442	0.111	1.454e-27	3.358e-23
10	Epithelial cells	*SLC4A7*	0.3921	0.428	0.167	4.713e-13	1.088e-08
10	Epithelial cells	*RAPGEF3*	0.3915	0.254	0.021	2.361e-68	5.452e-64
10	Epithelial cells	*LOC138844537*	0.3914	0.254	0.011	1.544e-118	3.566e-114
10	Epithelial cells	*LOC138844036*	0.3913	0.275	0.011	1.169e-135	2.700e-131
10	Epithelial cells	*ATXN7*	0.3912	0.623	0.220	3.128e-21	7.223e-17
10	Epithelial cells	*PCGF5*	0.3912	0.609	0.239	1.240e-17	2.863e-13
10	Epithelial cells	*ACAP2*	0.3909	0.681	0.333	1.118e-11	2.581e-07

Original cluster	Annotated cell type	Gene	avg_log2FC	pct.1	pct.2	Raw P value	Bonferroni-adjusted P value
10	Epithelial cells	*DENND4C*	0.3904	0.638	0.238	2.940e-19	6.788e-15
10	Epithelial cells	*ARIH2*	0.3902	0.428	0.143	3.026e-16	6.987e-12
10	Epithelial cells	*LOC100345591*	0.3901	0.681	0.294	1.861e-14	4.298e-10
10	Epithelial cells	*PIBF1*	0.3899	0.616	0.305	4.279e-10	9.880e-06
10	Epithelial cells	*LOC127483435*	0.3898	0.283	0.014	1.079e-120	2.492e-116
10	Epithelial cells	*GLRB*	0.3898	0.261	0.037	1.991e-38	4.597e-34
10	Epithelial cells	*OGT*	0.3889	0.696	0.355	2.693e-11	6.220e-07
10	Epithelial cells	*ZFAND3*	0.3886	0.638	0.295	1.163e-12	2.686e-08
10	Epithelial cells	*CEP104*	0.3884	0.275	0.064	2.041e-20	4.712e-16
10	Epithelial cells	*LEMD3*	0.3883	0.565	0.221	5.570e-16	1.286e-11
10	Epithelial cells	*EDN2*	0.3882	0.203	0.000	0	0
10	Epithelial cells	*PAG1*	0.3882	0.312	0.022	1.952e-93	4.508e-89
10	Epithelial cells	*CA13*	0.3874	0.246	0.015	4.054e-86	9.361e-82
10	Epithelial cells	*BCL2L1*	0.3871	0.268	0.064	1.257e-19	2.903e-15
10	Epithelial cells	*SLC30A1*	0.3870	0.312	0.055	3.999e-35	9.234e-31
10	Epithelial cells	*NT5DC1*	0.3869	0.442	0.111	1.558e-28	3.597e-24
10	Epithelial cells	*LOC127485720*	0.3868	0.290	0.017	1.540e-108	3.556e-104
10	Epithelial cells	*ACTN4*	0.3866	0.565	0.196	2.256e-18	5.210e-14
10	Epithelial cells	*TPR*	0.3863	0.739	0.387	1.984e-11	4.581e-07
10	Epithelial cells	*NFYC*	0.3863	0.551	0.192	2.336e-19	5.394e-15
10	Epithelial cells	*GALNT10*	0.3857	0.304	0.046	3.993e-41	9.220e-37
10	Epithelial cells	*PPP4R1*	0.3850	0.565	0.202	1.537e-18	3.549e-14
10	Epithelial cells	*LOC100338469*	0.3849	0.688	0.303	8.109e-15	1.872e-10
10	Epithelial cells	*MPLKIP*	0.3843	0.565	0.203	8.667e-18	2.001e-13
10	Epithelial cells	*BAZ2B*	0.3842	0.848	0.587	9.578e-08	2.212e-03
10	Epithelial cells	*TOMM20*	0.3833	0.812	0.404	3.296e-12	7.610e-08
10	Epithelial cells	*GTF3C2*	0.3833	0.471	0.097	6.299e-40	1.454e-35
10	Epithelial cells	*TULP4*	0.3832	0.420	0.128	1.257e-19	2.903e-15
10	Epithelial cells	*SMARCC1*	0.3827	0.681	0.268	6.961e-18	1.608e-13
10	Epithelial cells	*PELI2*	0.3825	0.572	0.227	1.416e-15	3.271e-11
10	Epithelial cells	*MAP3K5*	0.3824	0.348	0.061	6.774e-39	1.564e-34
10	Epithelial cells	*RAB3GAP2*	0.3822	0.594	0.214	8.618e-20	1.990e-15
10	Epithelial cells	*RANBP9*	0.3817	0.623	0.272	8.031e-14	1.855e-09
10	Epithelial cells	*WFDC1*	0.3816	0.290	0.033	2.337e-54	5.397e-50
10	Epithelial cells	*CDKAL1*	0.3812	0.746	0.444	2.357e-08	5.443e-04
10	Epithelial cells	*CHD1*	0.3811	0.601	0.280	8.574e-12	1.980e-07
10	Epithelial cells	*TAX1BP1*	0.3810	0.891	0.587	1.056e-09	2.438e-05
10	Epithelial cells	*RND3*	0.3809	0.652	0.342	3.923e-10	9.059e-06
10	Epithelial cells	*DAPL1*	0.3808	0.188	0.001	2.131e-254	4.921e-250
10	Epithelial cells	*CMSS1*	0.3802	0.413	0.161	9.717e-13	2.244e-08

Original cluster	Annotated cell type	Gene	avg_log2FC	pct.1	pct.2	Raw P value	Bonferroni-adjusted P value
10	Epithelial cells	*TPP2*	0.3801	0.565	0.169	6.040e-25	1.395e-20
10	Epithelial cells	*TMEM258*	0.3800	0.804	0.423	3.487e-11	8.053e-07
10	Epithelial cells	*TMC4*	0.3798	0.261	0.016	8.798e-93	2.032e-88
10	Epithelial cells	*SSR3*	0.3796	0.884	0.580	2.941e-08	6.790e-04
10	Epithelial cells	*LOC138849586*	0.3790	0.290	0.068	9.285e-22	2.144e-17
10	Epithelial cells	*WWP1*	0.3788	0.659	0.317	4.848e-12	1.119e-07
10	Epithelial cells	*HIPK2*	0.3787	0.464	0.123	8.069e-26	1.863e-21
10	Epithelial cells	*CDK14*	0.3787	0.594	0.272	8.462e-12	1.954e-07
10	Epithelial cells	*SNX25*	0.3782	0.413	0.061	2.889e-55	6.671e-51
10	Epithelial cells	*RMND5A*	0.3780	0.609	0.249	2.277e-15	5.258e-11
10	Epithelial cells	*TMEM164*	0.3778	0.391	0.065	8.397e-46	1.939e-41
10	Epithelial cells	*ZNF638*	0.3775	0.826	0.500	1.057e-08	2.441e-04
10	Epithelial cells	*WDFY1*	0.3775	0.435	0.098	8.997e-33	2.078e-28
10	Epithelial cells	*NME7*	0.3773	0.551	0.235	8.740e-13	2.018e-08
10	Epithelial cells	*A2ML1*	0.3773	0.246	0.003	4.990e-288	1.152e-283
10	Epithelial cells	*PAK2*	0.3773	0.703	0.372	3.795e-11	8.762e-07
10	Epithelial cells	*USP12*	0.3770	0.543	0.175	9.133e-22	2.109e-17
10	Epithelial cells	*ZNF24*	0.3770	0.580	0.225	2.553e-15	5.895e-11
10	Epithelial cells	*NR2C2*	0.3770	0.507	0.127	7.873e-32	1.818e-27
10	Epithelial cells	*SERTAD4*	0.3765	0.283	0.015	4.643e-111	1.072e-106
10	Epithelial cells	*NDRG1*	0.3759	0.551	0.199	5.583e-18	1.289e-13
10	Epithelial cells	*CSRP1*	0.3756	0.703	0.339	1.084e-11	2.502e-07
10	Epithelial cells	*BRD4*	0.3755	0.667	0.286	2.712e-14	6.263e-10
10	Epithelial cells	*ANKRD10*	0.3751	0.616	0.210	3.874e-21	8.946e-17
10	Epithelial cells	*PRRC2B*	0.3750	0.630	0.281	3.731e-12	8.617e-08
10	Epithelial cells	*SPAAR*	0.3748	0.275	0.026	1.684e-64	3.890e-60
10	Epithelial cells	*CYB5R1*	0.3747	0.572	0.163	5.539e-27	1.279e-22
10	Epithelial cells	*MDN1*	0.3747	0.471	0.143	7.661e-22	1.769e-17
10	Epithelial cells	*EPB41L3*	0.3746	0.493	0.179	1.310e-16	3.024e-12
10	Epithelial cells	*PPP1R13B*	0.3732	0.486	0.148	9.196e-22	2.124e-17
10	Epithelial cells	*LIMCH1*	0.3726	0.362	0.117	4.475e-16	1.033e-11
10	Epithelial cells	*TTC27*	0.3724	0.514	0.142	7.457e-27	1.722e-22
10	Epithelial cells	*KPNA4*	0.3721	0.609	0.243	4.242e-16	9.797e-12
10	Epithelial cells	*PCBD1*	0.3711	0.500	0.118	9.513e-34	2.197e-29
10	Epithelial cells	*NLK*	0.3710	0.362	0.132	9.904e-13	2.287e-08
10	Epithelial cells	*RNF169*	0.3708	0.601	0.239	6.231e-16	1.439e-11
10	Epithelial cells	*ZCWPW1*	0.3708	0.326	0.053	1.172e-39	2.706e-35
10	Epithelial cells	*RPA1*	0.3707	0.428	0.091	6.938e-35	1.602e-30
10	Epithelial cells	*SMAP1*	0.3707	0.623	0.264	3.447e-14	7.960e-10
10	Epithelial cells	*SLC45A4*	0.3702	0.297	0.013	1.280e-141	2.957e-137

Original cluster	Annotated cell type	Gene	avg_log2FC	pct.1	pct.2	Raw P value	Bonferroni-adjusted P value
10	Epithelial cells	*CLASP2*	0.3701	0.667	0.335	9.062e-11	2.093e-06
10	Epithelial cells	*PSME4*	0.3700	0.601	0.185	1.028e-25	2.375e-21
10	Epithelial cells	*LOC103345076*	0.3699	0.290	0.045	5.348e-38	1.235e-33
10	Epithelial cells	*HECA*	0.3698	0.435	0.131	5.587e-20	1.290e-15
10	Epithelial cells	*TRIP10*	0.3696	0.529	0.183	1.728e-18	3.990e-14
10	Epithelial cells	*TMEM141*	0.3691	0.435	0.123	6.362e-23	1.469e-18
10	Epithelial cells	*LOC108177130*	0.3691	0.391	0.103	9.183e-24	2.121e-19
10	Epithelial cells	*RAP1GDS1*	0.3690	0.399	0.146	5.845e-14	1.350e-09
10	Epithelial cells	*PAXBP1*	0.3689	0.536	0.203	2.545e-16	5.876e-12
10	Epithelial cells	*KLF6*	0.3689	0.804	0.499	5.239e-08	1.210e-03
10	Epithelial cells	*ASAP2*	0.3687	0.420	0.107	1.015e-25	2.345e-21
10	Epithelial cells	*RABGAP1L*	0.3684	0.703	0.364	3.019e-09	6.972e-05
10	Epithelial cells	*INO80*	0.3681	0.543	0.223	4.034e-13	9.315e-09
10	Epithelial cells	*PHF12*	0.3680	0.406	0.118	2.268e-20	5.238e-16
10	Epithelial cells	*RREB1*	0.3674	0.384	0.067	8.530e-42	1.970e-37
10	Epithelial cells	*NEK3*	0.3669	0.348	0.049	3.937e-50	9.091e-46
10	Epithelial cells	*SRSF10*	0.3669	0.761	0.447	1.010e-08	2.333e-04
10	Epithelial cells	*HMBOX1*	0.3667	0.623	0.301	9.194e-12	2.123e-07
10	Epithelial cells	*PIGM*	0.3659	0.297	0.021	1.518e-91	3.506e-87
10	Epithelial cells	*MYO5C*	0.3659	0.268	0.002	0	0
10	Epithelial cells	*LOC100328910*	0.3658	0.217	0.027	6.078e-38	1.404e-33
10	Epithelial cells	*ANGPT2*	0.3658	0.370	0.094	4.926e-24	1.137e-19
10	Epithelial cells	*ORMDL2*	0.3654	0.406	0.050	3.859e-67	8.910e-63
10	Epithelial cells	*HLCS*	0.3645	0.326	0.078	7.013e-24	1.619e-19
10	Epithelial cells	*NHLRC2*	0.3645	0.514	0.197	6.334e-15	1.463e-10
10	Epithelial cells	*LCN2*	0.3640	0.123	0.009	1.301e-36	3.004e-32
10	Epithelial cells	*RLA-A3*	0.3639	0.819	0.449	4.248e-11	9.810e-07
10	Epithelial cells	*ZBTB37*	0.3639	0.543	0.169	6.938e-23	1.602e-18
10	Epithelial cells	*LOC138843887*	0.3638	0.130	0.001	7.308e-160	1.688e-155
10	Epithelial cells	*GABRP*	0.3637	0.174	0.000	0	0
10	Epithelial cells	*RRAGB*	0.3636	0.435	0.133	2.917e-20	6.735e-16
10	Epithelial cells	*RALGAPB*	0.3634	0.384	0.067	1.042e-42	2.407e-38
10	Epithelial cells	*PPP3CC*	0.3631	0.522	0.168	1.198e-21	2.766e-17
10	Epithelial cells	*TNFRSF11A*	0.3628	0.326	0.026	3.267e-88	7.544e-84
10	Epithelial cells	*LOC103345948*	0.3628	0.413	0.083	1.250e-36	2.887e-32
10	Epithelial cells	*PLAG1*	0.3627	0.428	0.103	4.342e-28	1.003e-23
10	Epithelial cells	*EFNA1*	0.3627	0.384	0.055	5.322e-54	1.229e-49
10	Epithelial cells	*UBR4*	0.3619	0.507	0.185	7.904e-17	1.825e-12
10	Epithelial cells	*AMMECR1L*	0.3619	0.391	0.059	2.384e-51	5.505e-47
10	Epithelial cells	*CYRIB*	0.3615	0.449	0.134	1.245e-21	2.874e-17

Original cluster	Annotated cell type	Gene	avg_log2FC	pct.1	pct.2	Raw P value	Bonferroni-adjusted P value
10	Epithelial cells	*RYBP*	0.3615	0.623	0.320	8.310e-11	1.919e-06
10	Epithelial cells	*KIAA1958*	0.3614	0.384	0.082	5.005e-32	1.156e-27
10	Epithelial cells	*CAMSAP2*	0.3610	0.630	0.281	6.833e-13	1.578e-08
10	Epithelial cells	*DUSP6*	0.3609	0.283	0.024	9.051e-72	2.090e-67
10	Epithelial cells	*HIPK1*	0.3608	0.536	0.208	5.754e-16	1.329e-11
10	Epithelial cells	*FNBP4*	0.3605	0.703	0.361	2.245e-08	5.184e-04
10	Epithelial cells	*EP400*	0.3602	0.428	0.117	1.358e-23	3.135e-19
10	Epithelial cells	*FAM133B*	0.3597	0.580	0.223	1.072e-15	2.476e-11
10	Epithelial cells	*PRR14L*	0.3595	0.413	0.115	2.707e-22	6.251e-18
10	Epithelial cells	*SNX14*	0.3593	0.522	0.205	6.185e-15	1.428e-10
10	Epithelial cells	*ERN1*	0.3592	0.326	0.061	2.337e-33	5.396e-29
10	Epithelial cells	*MSANTD2*	0.3591	0.536	0.226	6.048e-14	1.397e-09
10	Epithelial cells	*PRLR*	0.3590	0.210	0.015	4.090e-64	9.446e-60
10	Epithelial cells	*MXI1*	0.3589	0.667	0.300	9.055e-13	2.091e-08
10	Epithelial cells	*OBSL1*	0.3584	0.522	0.192	2.877e-16	6.644e-12
10	Epithelial cells	*DPH6*	0.3583	0.623	0.321	9.864e-09	2.278e-04
10	Epithelial cells	*ATP6V1C2*	0.3583	0.239	0.001	0	0
10	Epithelial cells	*IL17RD*	0.3580	0.384	0.054	1.133e-54	2.616e-50
10	Epithelial cells	*SPINT1*	0.3580	0.268	0.010	2.458e-140	5.675e-136
10	Epithelial cells	*ITGAV*	0.3579	0.609	0.283	1.937e-11	4.474e-07
10	Epithelial cells	*ALDH9A1*	0.3578	0.493	0.131	5.528e-28	1.277e-23
10	Epithelial cells	*AP2B1*	0.3578	0.609	0.225	9.859e-18	2.277e-13
10	Epithelial cells	*KIAA1671*	0.3577	0.254	0.034	1.315e-40	3.036e-36
10	Epithelial cells	*YTHDC2*	0.3575	0.457	0.148	1.418e-18	3.273e-14
10	Epithelial cells	*LOC103349680*	0.3574	0.217	0.009	1.726e-107	3.986e-103
10	Epithelial cells	*NBN*	0.3574	0.522	0.175	1.224e-19	2.826e-15
10	Epithelial cells	*PDGFA*	0.3571	0.399	0.063	3.310e-49	7.644e-45
10	Epithelial cells	*MPDZ*	0.3568	0.609	0.239	7.436e-16	1.717e-11
10	Epithelial cells	*PTPRU*	0.3567	0.297	0.030	9.245e-64	2.135e-59
10	Epithelial cells	*MIA2*	0.3562	0.391	0.090	1.239e-29	2.862e-25
10	Epithelial cells	*LOC103345638*	0.3561	0.341	0.094	3.292e-19	7.602e-15
10	Epithelial cells	*KIF13B*	0.3553	0.391	0.082	5.068e-33	1.170e-28
10	Epithelial cells	*SLC7A6*	0.3553	0.326	0.023	1.457e-98	3.365e-94
10	Epithelial cells	*UIMC1*	0.3549	0.428	0.143	5.439e-17	1.256e-12
10	Epithelial cells	*BLTP1*	0.3549	0.783	0.494	1.099e-07	2.537e-03
10	Epithelial cells	*ZNF398*	0.3546	0.290	0.063	4.329e-24	9.996e-20
10	Epithelial cells	*PHIP*	0.3542	0.833	0.643	1.228e-06	2.837e-02
10	Epithelial cells	*WIPF1*	0.3541	0.551	0.187	6.575e-20	1.518e-15
10	Epithelial cells	*LARGE1*	0.3540	0.333	0.082	3.529e-23	8.149e-19
10	Epithelial cells	*PRKCZ*	0.3539	0.210	0.009	1.281e-103	2.959e-99

Original cluster	Annotated cell type	Gene	avg_log2FC	pct.1	pct.2	Raw P value	Bonferroni-adjusted P value
10	Epithelial cells	*SMG7*	0.3536	0.399	0.092	5.169e-29	1.194e-24
10	Epithelial cells	*TSPAN8*	0.3536	0.210	0.001	1.539e-293	3.554e-289
10	Epithelial cells	*CBFB*	0.3530	0.428	0.112	1.504e-25	3.472e-21
10	Epithelial cells	*MED13L*	0.3525	0.877	0.656	9.060e-08	2.092e-03
10	Epithelial cells	*KLF10*	0.3524	0.580	0.245	9.705e-14	2.241e-09
10	Epithelial cells	*TRIM8*	0.3522	0.399	0.070	3.608e-43	8.332e-39
10	Epithelial cells	*PRKCI*	0.3522	0.355	0.076	1.783e-29	4.118e-25
10	Epithelial cells	*RB1CC1*	0.3521	0.775	0.421	6.777e-10	1.565e-05
10	Epithelial cells	*GPC1*	0.3519	0.355	0.053	3.993e-47	9.221e-43
10	Epithelial cells	*ABL2*	0.3518	0.370	0.091	3.687e-25	8.514e-21
10	Epithelial cells	*THBS3*	0.3518	0.268	0.036	4.360e-41	1.007e-36
10	Epithelial cells	*ERC1*	0.3517	0.616	0.300	1.465e-10	3.383e-06
10	Epithelial cells	*REXO2*	0.3517	0.529	0.153	1.961e-25	4.528e-21
10	Epithelial cells	*SFMBT1*	0.3516	0.312	0.051	5.479e-38	1.265e-33
10	Epithelial cells	*GPRC5B*	0.3516	0.268	0.023	6.076e-69	1.403e-64
10	Epithelial cells	*TASOR2*	0.3515	0.659	0.328	1.652e-09	3.816e-05
10	Epithelial cells	*PRP4K*	0.3513	0.732	0.342	1.490e-11	3.441e-07
10	Epithelial cells	*VPS41*	0.3510	0.551	0.258	1.334e-09	3.082e-05
10	Epithelial cells	*NHSL3*	0.3506	0.312	0.025	5.258e-85	1.214e-80
10	Epithelial cells	*C8H4orf51*	0.3505	0.391	0.085	2.203e-31	5.086e-27
10	Epithelial cells	*VTI1A*	0.3503	0.565	0.270	8.500e-11	1.963e-06
10	Epithelial cells	*ZBTB10*	0.3500	0.500	0.174	1.019e-17	2.353e-13
10	Epithelial cells	*CUL1*	0.3494	0.536	0.202	5.022e-16	1.160e-11
10	Epithelial cells	*TMEM161B*	0.3493	0.391	0.104	1.236e-22	2.855e-18
10	Epithelial cells	*ATF7IP2*	0.3493	0.246	0.029	1.142e-45	2.637e-41
10	Epithelial cells	*DNAI7*	0.3482	0.370	0.087	1.014e-26	2.341e-22
10	Epithelial cells	*CHCHD3*	0.3482	0.529	0.284	1.060e-06	2.448e-02
10	Epithelial cells	*LOC108175886*	0.3480	0.210	0.018	1.836e-54	4.240e-50
10	Epithelial cells	*ARID4B*	0.3479	0.862	0.619	1.996e-07	4.609e-03
10	Epithelial cells	*APLF*	0.3474	0.630	0.273	1.957e-12	4.520e-08
10	Epithelial cells	*PPTC7*	0.3472	0.283	0.052	2.680e-29	6.188e-25
10	Epithelial cells	*CDK19*	0.3472	0.543	0.220	5.306e-15	1.225e-10
10	Epithelial cells	*BHLHE40*	0.3471	0.457	0.167	4.082e-15	9.426e-11
10	Epithelial cells	*DEGS1*	0.3469	0.500	0.172	5.135e-18	1.186e-13
10	Epithelial cells	*KIF5B*	0.3468	0.667	0.302	6.750e-12	1.559e-07
10	Epithelial cells	*PDIA6*	0.3468	0.739	0.365	7.309e-11	1.688e-06
10	Epithelial cells	*EPPK1*	0.3468	0.239	0.001	0	0
10	Epithelial cells	*HNRNPM*	0.3467	0.594	0.224	2.799e-16	6.464e-12
10	Epithelial cells	*STYXL2*	0.3467	0.181	0.000	0	0
10	Epithelial cells	*PCCA*	0.3467	0.471	0.150	1.886e-20	4.356e-16

Original cluster	Annotated cell type	Gene	avg_log2FC	pct.1	pct.2	Raw P value	Bonferroni-adjusted P value
10	Epithelial cells	*LOC108177422*	0.3464	0.551	0.235	7.645e-13	1.765e-08
10	Epithelial cells	*ATM*	0.3463	0.464	0.187	1.227e-12	2.833e-08
10	Epithelial cells	*BRD7*	0.3463	0.500	0.173	8.884e-18	2.051e-13
10	Epithelial cells	*ZDHHC2*	0.3462	0.217	0.025	1.380e-41	3.187e-37
10	Epithelial cells	*MTMR12*	0.3461	0.442	0.101	1.662e-32	3.838e-28
10	Epithelial cells	*PNP*	0.3459	0.500	0.146	1.297e-24	2.994e-20
10	Epithelial cells	*LOC127492305*	0.3456	0.159	0.004	1.307e-118	3.018e-114
10	Epithelial cells	*UHRF2*	0.3456	0.623	0.302	4.416e-10	1.020e-05
10	Epithelial cells	*ZC3H13*	0.3455	0.746	0.379	9.162e-11	2.116e-06
10	Epithelial cells	*INVS*	0.3455	0.493	0.153	1.486e-21	3.430e-17
10	Epithelial cells	*TAPBP*	0.3445	0.297	0.056	1.904e-30	4.396e-26
10	Epithelial cells	*NDUFA11*	0.3442	0.659	0.297	1.259e-11	2.906e-07
10	Epithelial cells	*GPSM2*	0.3440	0.355	0.057	5.209e-43	1.203e-38
10	Epithelial cells	*RAB2A*	0.3436	0.783	0.486	1.542e-08	3.561e-04
10	Epithelial cells	*QSOX1*	0.3432	0.370	0.089	1.171e-25	2.703e-21
10	Epithelial cells	*SRSF4*	0.3425	0.696	0.359	6.886e-10	1.590e-05
10	Epithelial cells	*HEPH*	0.3425	0.138	0.002	5.304e-156	1.225e-151
10	Epithelial cells	*CHD4*	0.3421	0.594	0.268	2.859e-11	6.603e-07
10	Epithelial cells	*MSL1*	0.3420	0.522	0.167	4.832e-21	1.116e-16
10	Epithelial cells	*LOC138844427*	0.3419	0.196	0.001	1.678e-282	3.876e-278
10	Epithelial cells	*MANSC1*	0.3417	0.268	0.025	8.353e-63	1.929e-58
10	Epithelial cells	*GNG5*	0.3414	0.775	0.480	6.272e-08	1.448e-03
10	Epithelial cells	*BROX*	0.3414	0.507	0.149	4.540e-24	1.048e-19
10	Epithelial cells	*TSPAN2*	0.3412	0.370	0.057	6.824e-48	1.576e-43
10	Epithelial cells	*ATG2B*	0.3405	0.377	0.101	1.036e-21	2.392e-17
10	Epithelial cells	*NAA15*	0.3405	0.536	0.182	2.259e-19	5.216e-15
10	Epithelial cells	*THOC2*	0.3403	0.674	0.290	3.096e-14	7.148e-10
10	Epithelial cells	*HKDC1*	0.3402	0.210	0.003	3.418e-197	7.893e-193
10	Epithelial cells	*ZNF664*	0.3402	0.283	0.035	6.866e-49	1.585e-44
10	Epithelial cells	*EPC2*	0.3402	0.710	0.341	9.794e-12	2.262e-07
10	Epithelial cells	*TNFRSF12A*	0.3394	0.290	0.032	2.603e-55	6.010e-51
10	Epithelial cells	*RBM27*	0.3393	0.500	0.192	5.491e-15	1.268e-10
10	Epithelial cells	*FBXW8*	0.3392	0.529	0.185	3.332e-17	7.694e-13
10	Epithelial cells	*ZMYM6*	0.3389	0.283	0.087	2.692e-13	6.216e-09
10	Epithelial cells	*AP3S2*	0.3384	0.464	0.151	1.180e-18	2.725e-14
10	Epithelial cells	*MEX3D*	0.3383	0.268	0.033	7.433e-47	1.716e-42
10	Epithelial cells	*MTAP*	0.3381	0.449	0.114	1.640e-27	3.787e-23
10	Epithelial cells	*RC3H1*	0.3377	0.580	0.213	7.259e-18	1.676e-13
10	Epithelial cells	*CCAR1*	0.3376	0.717	0.377	6.817e-09	1.574e-04
10	Epithelial cells	*EPS8L1*	0.3372	0.196	0.002	2.976e-220	6.873e-216

Original cluster	Annotated cell type	Gene	avg_log2FC	pct.1	pct.2	Raw P value	Bonferroni-adjusted P value
10	Epithelial cells	*TAF4*	0.3370	0.239	0.060	5.923e-16	1.368e-11
10	Epithelial cells	*WIPF2*	0.3368	0.377	0.097	8.498e-24	1.962e-19
10	Epithelial cells	*EXOC2*	0.3366	0.449	0.120	1.285e-25	2.967e-21
10	Epithelial cells	*RNF138*	0.3365	0.587	0.240	3.127e-14	7.222e-10
10	Epithelial cells	*MCL1*	0.3363	0.804	0.426	4.306e-11	9.944e-07
10	Epithelial cells	*MYSM1*	0.3362	0.594	0.257	1.669e-13	3.854e-09
10	Epithelial cells	*GPRC5A*	0.3361	0.232	0.045	4.158e-23	9.603e-19
10	Epithelial cells	*CNTNAP4*	0.3360	0.174	0.008	7.744e-79	1.788e-74
10	Epithelial cells	*GPBP1*	0.3357	0.826	0.544	2.308e-07	5.331e-03
10	Epithelial cells	*RNF180*	0.3356	0.304	0.062	5.788e-28	1.336e-23
10	Epithelial cells	*SMC4*	0.3353	0.406	0.138	1.537e-15	3.548e-11
10	Epithelial cells	*DUSP14*	0.3352	0.341	0.039	6.228e-63	1.438e-58
10	Epithelial cells	*GALNT5*	0.3351	0.188	0.011	1.439e-71	3.324e-67
10	Epithelial cells	*FAM13B*	0.3350	0.638	0.279	4.927e-13	1.138e-08
10	Epithelial cells	*HTT*	0.3344	0.442	0.111	4.476e-28	1.034e-23
10	Epithelial cells	*PLEKHJ1*	0.3344	0.500	0.177	4.982e-17	1.150e-12
10	Epithelial cells	*GABRG1*	0.3343	0.225	0.033	7.483e-32	1.728e-27
10	Epithelial cells	*NCOR1*	0.3340	0.848	0.525	4.249e-09	9.812e-05
10	Epithelial cells	*ADK*	0.3337	0.580	0.277	3.877e-11	8.952e-07
10	Epithelial cells	*PCM1*	0.3336	0.826	0.542	1.193e-07	2.756e-03
10	Epithelial cells	*GRB2*	0.3335	0.529	0.202	1.166e-15	2.693e-11
10	Epithelial cells	*DAP*	0.3333	0.551	0.220	1.774e-14	4.096e-10
10	Epithelial cells	*PARP4*	0.3333	0.464	0.121	3.560e-27	8.222e-23
10	Epithelial cells	*TGFA*	0.3332	0.203	0.005	2.670e-151	6.167e-147
10	Epithelial cells	*FRRS1*	0.3330	0.232	0.002	0	0
10	Epithelial cells	*DOCK11*	0.3326	0.283	0.059	1.277e-24	2.949e-20
10	Epithelial cells	*HDLBP*	0.3322	0.543	0.222	2.760e-13	6.373e-09
10	Epithelial cells	*XRCC5*	0.3319	0.652	0.313	6.138e-11	1.417e-06
10	Epithelial cells	*TMEM170B*	0.3315	0.290	0.073	2.856e-19	6.596e-15
10	Epithelial cells	*DNAJC10*	0.3315	0.572	0.201	1.034e-20	2.388e-16
10	Epithelial cells	*PPP4R3A*	0.3312	0.638	0.263	1.569e-14	3.622e-10
10	Epithelial cells	*ZMYM5*	0.3311	0.413	0.116	3.110e-22	7.182e-18
10	Epithelial cells	*PLEKHM3*	0.3310	0.406	0.106	7.409e-25	1.711e-20
10	Epithelial cells	*LOC127493550*	0.3310	0.442	0.111	5.705e-28	1.317e-23
10	Epithelial cells	*TOM1L1*	0.3309	0.370	0.059	2.903e-45	6.703e-41
10	Epithelial cells	*RAP1B*	0.3306	0.565	0.187	1.072e-20	2.476e-16
10	Epithelial cells	*LOC103352476*	0.3304	0.275	0.072	5.739e-18	1.325e-13
10	Epithelial cells	*YAF2*	0.3302	0.587	0.233	4.800e-15	1.108e-10
10	Epithelial cells	*VPS53*	0.3296	0.428	0.105	2.669e-28	6.162e-24
10	Epithelial cells	*UCK2*	0.3292	0.261	0.047	2.412e-28	5.570e-24

Original cluster	Annotated cell type	Gene	avg_log2FC	pct.1	pct.2	Raw P value	Bonferroni-adjusted P value
10	Epithelial cells	*NCK2*	0.3292	0.435	0.134	4.402e-19	1.017e-14
10	Epithelial cells	*HID1*	0.3289	0.196	0.013	1.097e-65	2.534e-61
10	Epithelial cells	*IL18BP*	0.3287	0.261	0.018	1.316e-81	3.039e-77
10	Epithelial cells	*PARVA*	0.3285	0.551	0.202	1.132e-16	2.615e-12
10	Epithelial cells	*DIP2C*	0.3285	0.681	0.395	7.964e-08	1.839e-03
10	Epithelial cells	*MORN1*	0.3283	0.246	0.017	1.859e-76	4.293e-72
10	Epithelial cells	*OPA1*	0.3283	0.594	0.214	3.130e-18	7.228e-14
10	Epithelial cells	*SRSF9*	0.3283	0.710	0.343	2.192e-10	5.063e-06
10	Epithelial cells	*GTF2H2C*	0.3277	0.514	0.137	3.172e-28	7.324e-24
10	Epithelial cells	*YES1*	0.3271	0.565	0.227	1.152e-13	2.661e-09
10	Epithelial cells	*ANKH*	0.3270	0.326	0.106	7.503e-14	1.732e-09
10	Epithelial cells	*FAM120C*	0.3270	0.283	0.059	1.066e-24	2.462e-20
10	Epithelial cells	*DDX46*	0.3269	0.696	0.294	2.433e-14	5.619e-10
10	Epithelial cells	*CEP128*	0.3268	0.399	0.195	4.052e-07	9.356e-03
10	Epithelial cells	*MSH2*	0.3264	0.362	0.107	1.321e-17	3.051e-13
10	Epithelial cells	*EIF5*	0.3263	0.841	0.561	7.611e-08	1.758e-03
10	Epithelial cells	*CPEB1*	0.3262	0.181	0.015	1.780e-47	4.110e-43
10	Epithelial cells	*LRCH3*	0.3259	0.667	0.354	2.671e-09	6.169e-05
10	Epithelial cells	*FAM185A*	0.3258	0.283	0.044	8.753e-37	2.021e-32
10	Epithelial cells	*PAIP2*	0.3256	0.862	0.501	1.207e-08	2.787e-04
10	Epithelial cells	*MAB21L3*	0.3252	0.254	0.010	2.140e-127	4.941e-123
10	Epithelial cells	*LOC100340453*	0.3251	0.333	0.079	2.360e-24	5.449e-20
10	Epithelial cells	*LOC138847706*	0.3245	0.514	0.200	5.214e-14	1.204e-09
10	Epithelial cells	*LOC138844593*	0.3245	0.572	0.250	2.198e-12	5.075e-08
10	Epithelial cells	*EPB41L1*	0.3240	0.362	0.066	1.859e-36	4.292e-32
10	Epithelial cells	*MAGIX*	0.3237	0.246	0.010	1.655e-128	3.821e-124
10	Epithelial cells	*NHERF1*	0.3236	0.297	0.026	6.045e-75	1.396e-70
10	Epithelial cells	*TRIP12*	0.3236	0.529	0.208	1.693e-14	3.909e-10
10	Epithelial cells	*RSBN1*	0.3233	0.536	0.207	3.814e-14	8.806e-10
10	Epithelial cells	*NSMCE2*	0.3233	0.565	0.260	3.789e-11	8.749e-07
10	Epithelial cells	*PCSK5*	0.3232	0.391	0.141	1.046e-13	2.415e-09
10	Epithelial cells	*ACE*	0.3230	0.225	0.022	8.300e-51	1.917e-46
10	Epithelial cells	*PGM2L1*	0.3230	0.355	0.115	4.192e-15	9.679e-11
10	Epithelial cells	*LOC138843573*	0.3226	0.239	0.006	4.406e-167	1.018e-162
10	Epithelial cells	*CAPRIN1*	0.3225	0.572	0.279	3.730e-08	8.614e-04
10	Epithelial cells	*NAA35*	0.3220	0.435	0.132	2.983e-20	6.889e-16
10	Epithelial cells	*SCAF11*	0.3218	0.725	0.333	1.196e-12	2.762e-08
10	Epithelial cells	*ATL2*	0.3216	0.652	0.273	2.384e-15	5.505e-11
10	Epithelial cells	*TUFT1*	0.3215	0.428	0.112	1.850e-25	4.271e-21
10	Epithelial cells	*PAPOLA*	0.3214	0.775	0.420	3.447e-09	7.960e-05

Original cluster	Annotated cell type	Gene	avg_log2FC	pct.1	pct.2	Raw P value	Bonferroni-adjusted P value
10	Epithelial cells	*PLLP*	0.3214	0.225	0.003	1.144e-222	2.641e-218
10	Epithelial cells	*TMEM87B*	0.3212	0.471	0.140	3.364e-22	7.767e-18
10	Epithelial cells	*TMEM14C*	0.3211	0.732	0.388	9.230e-09	2.131e-04
10	Epithelial cells	*KLHL3*	0.3209	0.319	0.065	7.312e-29	1.688e-24
10	Epithelial cells	*HINT2*	0.3207	0.457	0.104	1.181e-32	2.727e-28
10	Epithelial cells	*CDKN2B*	0.3205	0.225	0.002	5.152e-268	1.190e-263
10	Epithelial cells	*MIB1*	0.3204	0.638	0.302	1.957e-11	4.520e-07
10	Epithelial cells	*LPAR1*	0.3203	0.442	0.197	2.192e-09	5.063e-05
10	Epithelial cells	*AMBRA1*	0.3203	0.478	0.139	3.779e-23	8.727e-19
10	Epithelial cells	*LOC138849988*	0.3199	0.225	0.032	6.655e-33	1.537e-28
10	Epithelial cells	*GIT2*	0.3194	0.297	0.121	2.619e-08	6.047e-04
10	Epithelial cells	*RYR3*	0.3192	0.254	0.094	3.400e-09	7.852e-05
10	Epithelial cells	*SEC24B*	0.3192	0.493	0.188	3.354e-14	7.745e-10
10	Epithelial cells	*UBR3*	0.3191	0.652	0.327	6.894e-10	1.592e-05
10	Epithelial cells	*ANXA3*	0.3189	0.529	0.190	4.699e-17	1.085e-12
10	Epithelial cells	*LOC138849562*	0.3182	0.413	0.079	4.278e-39	9.879e-35
10	Epithelial cells	*ASH1L*	0.3177	0.797	0.501	3.396e-08	7.841e-04
10	Epithelial cells	*TAF5*	0.3177	0.268	0.045	1.284e-31	2.965e-27
10	Epithelial cells	*CLOCK*	0.3176	0.457	0.126	1.969e-24	4.547e-20
10	Epithelial cells	*POLD3*	0.3175	0.413	0.111	2.813e-23	6.496e-19
10	Epithelial cells	*TES*	0.3174	0.399	0.087	6.821e-32	1.575e-27
10	Epithelial cells	*TAB2*	0.3173	0.674	0.252	7.679e-18	1.773e-13
10	Epithelial cells	*LOC127486866*	0.3172	0.188	0.003	1.538e-166	3.551e-162
10	Epithelial cells	*UBXN2B*	0.3171	0.399	0.112	3.927e-21	9.068e-17
10	Epithelial cells	*SLC2A13*	0.3169	0.420	0.131	1.796e-18	4.148e-14
10	Epithelial cells	*RAB11FIP1*	0.3168	0.435	0.144	4.454e-17	1.029e-12
10	Epithelial cells	*LOC103346519*	0.3168	0.130	0.037	4.541e-08	1.049e-03
10	Epithelial cells	*MKNK2*	0.3159	0.486	0.184	1.872e-14	4.323e-10
10	Epithelial cells	*RFX3*	0.3158	0.790	0.494	1.948e-07	4.498e-03
10	Epithelial cells	*FOXJ3*	0.3156	0.587	0.278	1.211e-09	2.797e-05
10	Epithelial cells	*VIPR2*	0.3154	0.246	0.046	3.093e-25	7.143e-21
10	Epithelial cells	*RIPK4*	0.3152	0.225	0.001	0	0
10	Epithelial cells	*BRD1*	0.3148	0.384	0.089	8.432e-28	1.947e-23
10	Epithelial cells	*ADAM10*	0.3145	0.652	0.292	2.380e-12	5.496e-08
10	Epithelial cells	*HSBP1*	0.3143	0.703	0.348	8.133e-11	1.878e-06
10	Epithelial cells	*LOC138849834*	0.3142	0.130	0.000	6.465e-247	1.493e-242
10	Epithelial cells	*BCR*	0.3141	0.355	0.064	3.603e-37	8.320e-33
10	Epithelial cells	*BICRAL*	0.3138	0.609	0.268	1.045e-11	2.414e-07
10	Epithelial cells	*TCERG1*	0.3137	0.536	0.204	7.446e-16	1.719e-11
10	Epithelial cells	*HS6ST1*	0.3135	0.355	0.100	1.259e-19	2.908e-15

Original cluster	Annotated cell type	Gene	avg_log2FC	pct.1	pct.2	Raw P value	Bonferroni-adjusted P value
10	Epithelial cells	*STAMBPL1*	0.3134	0.246	0.023	1.786e-57	4.123e-53
10	Epithelial cells	*LOC100351569*	0.3133	0.558	0.239	1.352e-12	3.122e-08
10	Epithelial cells	*MED14*	0.3128	0.413	0.114	4.719e-23	1.090e-18
10	Epithelial cells	*DHCR7*	0.3128	0.319	0.044	7.840e-47	1.810e-42
10	Epithelial cells	*SHOC2*	0.3125	0.696	0.378	8.906e-09	2.057e-04
10	Epithelial cells	*ZFP91*	0.3125	0.659	0.296	9.318e-13	2.152e-08
10	Epithelial cells	*MICOS13*	0.3123	0.616	0.275	1.560e-10	3.602e-06
10	Epithelial cells	*CXCL16*	0.3121	0.239	0.010	9.604e-122	2.218e-117
10	Epithelial cells	*MMP15*	0.3121	0.239	0.008	6.337e-135	1.463e-130
10	Epithelial cells	*NEU1*	0.3121	0.261	0.025	2.412e-60	5.569e-56
10	Epithelial cells	*WDR33*	0.3121	0.739	0.312	5.457e-14	1.260e-09
10	Epithelial cells	*VPS37D*	0.3118	0.275	0.046	1.661e-32	3.835e-28
10	Epithelial cells	*PDE3A*	0.3112	0.725	0.293	8.901e-21	2.055e-16
10	Epithelial cells	*UBE3C*	0.3112	0.493	0.171	4.094e-17	9.453e-13
10	Epithelial cells	*BACE2*	0.3111	0.457	0.120	4.665e-26	1.077e-21
10	Epithelial cells	*NBR1*	0.3110	0.587	0.263	1.494e-11	3.450e-07
10	Epithelial cells	*RIC1*	0.3106	0.500	0.226	3.804e-10	8.784e-06
10	Epithelial cells	*CFAP43*	0.3106	0.304	0.078	4.302e-20	9.935e-16
10	Epithelial cells	*FKBP15*	0.3103	0.232	0.049	7.824e-21	1.807e-16
10	Epithelial cells	*R3HDM1*	0.3102	0.486	0.170	8.879e-17	2.050e-12
10	Epithelial cells	*DCAF10*	0.3100	0.239	0.073	4.155e-12	9.595e-08
10	Epithelial cells	*DPP4*	0.3097	0.225	0.015	1.588e-72	3.666e-68
10	Epithelial cells	*F2RL1*	0.3096	0.167	0.005	1.832e-99	4.231e-95
10	Epithelial cells	*TOMM5*	0.3092	0.471	0.150	3.340e-19	7.712e-15
10	Epithelial cells	*SH3RF3*	0.3090	0.290	0.060	1.391e-25	3.212e-21
10	Epithelial cells	*LOC100346112*	0.3090	0.877	0.584	1.301e-06	3.004e-02
10	Epithelial cells	*RLF*	0.3089	0.572	0.228	1.190e-14	2.748e-10
10	Epithelial cells	*UBALD2*	0.3087	0.442	0.155	9.105e-16	2.102e-11
10	Epithelial cells	*CDC25B*	0.3083	0.268	0.025	2.698e-63	6.229e-59
10	Epithelial cells	*CLDN11*	0.3083	0.261	0.106	5.812e-08	1.342e-03
10	Epithelial cells	*LARP4*	0.3082	0.500	0.190	5.058e-14	1.168e-09
10	Epithelial cells	*ILDR1*	0.3081	0.246	0.005	3.699e-196	8.541e-192
10	Epithelial cells	*RPRD1A*	0.3073	0.486	0.173	8.364e-16	1.932e-11
10	Epithelial cells	*UBE3A*	0.3073	0.696	0.370	1.029e-08	2.376e-04
10	Epithelial cells	*GULP1*	0.3071	0.768	0.445	1.215e-07	2.807e-03
10	Epithelial cells	*SPAG9*	0.3068	0.616	0.297	1.745e-10	4.029e-06
10	Epithelial cells	*LOC138848526*	0.3067	0.123	0.004	6.958e-70	1.607e-65
10	Epithelial cells	*RBM26*	0.3065	0.696	0.411	8.671e-08	2.002e-03
10	Epithelial cells	*PNPLA2*	0.3060	0.290	0.077	1.610e-17	3.717e-13
10	Epithelial cells	*FRAT2*	0.3059	0.232	0.033	5.502e-35	1.271e-30

Original cluster	Annotated cell type	Gene	avg_log2FC	pct.1	pct.2	Raw P value	Bonferroni-adjusted P value
10	Epithelial cells	*DTWD2*	0.3058	0.290	0.072	5.733e-20	1.324e-15
10	Epithelial cells	*HEATR5A*	0.3058	0.333	0.118	1.599e-12	3.693e-08
10	Epithelial cells	*CWC27*	0.3056	0.652	0.346	3.610e-08	8.336e-04
10	Epithelial cells	*TLK2*	0.3053	0.601	0.255	2.205e-13	5.092e-09
10	Epithelial cells	*CTDSP2*	0.3053	0.645	0.307	1.899e-10	4.384e-06
10	Epithelial cells	*PRPSAP2*	0.3052	0.413	0.135	1.385e-16	3.198e-12
10	Epithelial cells	*ABCB7*	0.3047	0.355	0.109	9.182e-17	2.120e-12
10	Epithelial cells	*NUFIP2*	0.3046	0.790	0.430	1.842e-10	4.252e-06
10	Epithelial cells	*MAP3K9*	0.3043	0.239	0.021	5.125e-60	1.183e-55
10	Epithelial cells	*STARD10*	0.3042	0.225	0.040	5.105e-25	1.179e-20
10	Epithelial cells	*FBXL5*	0.3041	0.507	0.179	9.228e-17	2.131e-12
10	Epithelial cells	*LOC127490116*	0.3038	0.304	0.071	2.261e-22	5.220e-18
10	Epithelial cells	*NUBPL*	0.3038	0.580	0.265	5.876e-11	1.357e-06
10	Epithelial cells	*TP53BP1*	0.3037	0.457	0.174	8.713e-13	2.012e-08
10	Epithelial cells	*ATP6V1F*	0.3036	0.486	0.145	6.030e-22	1.392e-17
10	Epithelial cells	*SMAD9*	0.3036	0.275	0.040	5.130e-39	1.185e-34
10	Epithelial cells	*LOC100338442*	0.3035	0.290	0.055	5.559e-29	1.284e-24
10	Epithelial cells	*SLC14A1*	0.3034	0.145	0.015	4.881e-32	1.127e-27
10	Epithelial cells	*CALR*	0.3030	0.522	0.224	1.164e-10	2.688e-06
10	Epithelial cells	*SLC39A8*	0.3028	0.261	0.032	8.506e-46	1.964e-41
10	Epithelial cells	*VAMP8*	0.3026	0.478	0.156	1.326e-18	3.062e-14
10	Epithelial cells	*TLCD4*	0.3025	0.333	0.063	1.419e-32	3.277e-28
10	Epithelial cells	*KRAS*	0.3025	0.478	0.157	9.655e-19	2.230e-14
10	Epithelial cells	*LRRC59*	0.3025	0.341	0.066	2.662e-32	6.147e-28
10	Epithelial cells	*TRAPPC8*	0.3023	0.514	0.196	4.172e-15	9.634e-11
10	Epithelial cells	*TMED4*	0.3019	0.507	0.140	7.605e-26	1.756e-21
10	Epithelial cells	*UBE3D*	0.3019	0.406	0.143	9.682e-15	2.236e-10
10	Epithelial cells	*GLG1*	0.3018	0.630	0.266	7.137e-13	1.648e-08
10	Epithelial cells	*PDK2*	0.3017	0.355	0.095	2.657e-20	6.134e-16
10	Epithelial cells	*BCL7A*	0.3017	0.355	0.073	2.926e-31	6.758e-27
10	Epithelial cells	*PAFAH1B1*	0.3014	0.739	0.409	3.756e-08	8.674e-04
10	Epithelial cells	*APPBP2*	0.3014	0.558	0.252	5.062e-11	1.169e-06
10	Epithelial cells	*RHBDD1*	0.3014	0.471	0.207	1.705e-10	3.938e-06
10	Epithelial cells	*EIF5A2*	0.3013	0.572	0.208	1.187e-16	2.742e-12
10	Epithelial cells	*OTULINL*	0.3012	0.362	0.108	4.403e-17	1.017e-12
10	Epithelial cells	*SLC10A7*	0.3008	0.377	0.104	7.089e-21	1.637e-16
10	Epithelial cells	*PPP2CA*	0.3007	0.580	0.206	1.090e-17	2.518e-13
10	Epithelial cells	*JMJD1C*	0.3006	0.891	0.703	1.419e-07	3.277e-03
10	Epithelial cells	*ATXN2*	0.3004	0.529	0.195	8.954e-16	2.068e-11
10	Epithelial cells	*LOC100349682*	0.3003	0.130	0.001	1.890e-186	4.364e-182

Original cluster	Annotated cell type	Gene	avg_log2FC	pct.1	pct.2	Raw P value	Bonferroni-adjusted P value
10	Epithelial cells	*UBE2G1*	0.3002	0.536	0.218	3.085e-13	7.125e-09
10	Epithelial cells	*SLC16A11*	0.3002	0.217	0.003	5.255e-237	1.214e-232
10	Epithelial cells	*SPOPL*	0.3001	0.457	0.158	1.510e-16	3.486e-12
10	Epithelial cells	*THRAP3*	0.2999	0.819	0.426	1.056e-09	2.438e-05
10	Epithelial cells	*SCP2*	0.2998	0.522	0.154	6.204e-24	1.433e-19
10	Epithelial cells	*DNAJB14*	0.2998	0.428	0.121	3.863e-22	8.921e-18
10	Epithelial cells	*NKAPD1*	0.2996	0.478	0.179	1.566e-13	3.616e-09
10	Epithelial cells	*CNOT4*	0.2992	0.652	0.346	1.486e-08	3.431e-04
10	Epithelial cells	*PWWP2A*	0.2992	0.514	0.188	3.518e-15	8.125e-11
10	Epithelial cells	*RASA2*	0.2988	0.616	0.293	1.559e-11	3.601e-07
10	Epithelial cells	*TENT2*	0.2987	0.580	0.208	1.032e-17	2.382e-13
10	Epithelial cells	*MACC1*	0.2986	0.210	0.001	0	0
10	Epithelial cells	*LOC108177469*	0.2985	0.123	0.006	4.878e-55	1.126e-50
10	Epithelial cells	*LOC100354611*	0.2985	0.428	0.108	1.694e-26	3.911e-22
10	Epithelial cells	*LOC138849704*	0.2985	0.138	0.003	2.966e-105	6.849e-101
10	Epithelial cells	*CDIN1*	0.2984	0.384	0.158	1.363e-10	3.148e-06
10	Epithelial cells	*FMO4*	0.2984	0.225	0.045	1.765e-21	4.076e-17
10	Epithelial cells	*MTUS2*	0.2982	0.145	0.010	7.886e-48	1.821e-43
10	Epithelial cells	*FAM168A*	0.2980	0.391	0.121	5.005e-18	1.156e-13
10	Epithelial cells	*YPEL5*	0.2977	0.630	0.232	1.390e-16	3.210e-12
10	Epithelial cells	*CSPP1*	0.2970	0.645	0.352	1.655e-07	3.822e-03
10	Epithelial cells	*N4BP1*	0.2970	0.457	0.174	1.453e-13	3.355e-09
10	Epithelial cells	*PLXNB2*	0.2969	0.290	0.069	1.322e-20	3.052e-16
10	Epithelial cells	*GSK3B*	0.2969	0.717	0.400	2.277e-08	5.259e-04
10	Epithelial cells	*PTP4A2*	0.2968	0.645	0.327	1.620e-09	3.742e-05
10	Epithelial cells	*SPPL3*	0.2966	0.493	0.207	8.740e-12	2.018e-07
10	Epithelial cells	*ADAMTSL3*	0.2965	0.188	0.040	8.398e-17	1.939e-12
10	Epithelial cells	*EFL1*	0.2963	0.457	0.142	1.270e-19	2.932e-15
10	Epithelial cells	*FDFT1*	0.2962	0.399	0.148	2.277e-12	5.257e-08
10	Epithelial cells	*ALDH3A2*	0.2962	0.326	0.078	5.916e-23	1.366e-18
10	Epithelial cells	*TAF15*	0.2962	0.616	0.280	8.351e-11	1.928e-06
10	Epithelial cells	*RPS6KB1*	0.2960	0.500	0.225	5.097e-10	1.177e-05
10	Epithelial cells	*SMAD7*	0.2957	0.362	0.064	1.203e-38	2.778e-34
10	Epithelial cells	*ZBTB41*	0.2955	0.413	0.155	8.008e-13	1.849e-08
10	Epithelial cells	*RAB15*	0.2955	0.232	0.012	1.431e-94	3.305e-90
10	Epithelial cells	*LOC100346971*	0.2953	0.145	0.001	1.811e-230	4.182e-226
10	Epithelial cells	*MPP7*	0.2950	0.399	0.075	7.003e-39	1.617e-34
10	Epithelial cells	*ZC3H12C*	0.2947	0.312	0.110	3.563e-12	8.227e-08
10	Epithelial cells	*LOC103349457*	0.2945	0.384	0.127	1.025e-15	2.367e-11
10	Epithelial cells	*POT1*	0.2944	0.478	0.174	2.601e-15	6.006e-11

Original cluster	Annotated cell type	Gene	avg_log2FC	pct.1	pct.2	Raw P value	Bonferroni-adjusted P value
10	Epithelial cells	*OTUD4*	0.2942	0.355	0.082	3.867e-26	8.929e-22
10	Epithelial cells	*MIPEP*	0.2939	0.428	0.103	1.515e-28	3.497e-24
10	Epithelial cells	*SYBU*	0.2935	0.232	0.016	5.286e-73	1.221e-68
10	Epithelial cells	*LOC103348026*	0.2933	0.225	0.033	1.551e-31	3.582e-27
10	Epithelial cells	*NCK1*	0.2930	0.688	0.345	1.087e-09	2.510e-05
10	Epithelial cells	*MAPK13*	0.2930	0.203	0.008	1.281e-104	2.958e-100
10	Epithelial cells	*PLEKHA1*	0.2927	0.413	0.100	1.143e-27	2.640e-23
10	Epithelial cells	*SMAD4*	0.2925	0.587	0.252	1.192e-12	2.754e-08
10	Epithelial cells	*ATP8B1*	0.2922	0.391	0.066	7.837e-44	1.810e-39
10	Epithelial cells	*CCDC88C*	0.2921	0.196	0.017	6.344e-51	1.465e-46
10	Epithelial cells	*PROSER2*	0.2920	0.239	0.006	2.189e-165	5.056e-161
10	Epithelial cells	*MARK1*	0.2919	0.435	0.165	1.905e-13	4.399e-09
10	Epithelial cells	*STIM1*	0.2917	0.457	0.142	3.025e-20	6.985e-16
10	Epithelial cells	*JAK1*	0.2916	0.841	0.501	3.308e-07	7.639e-03
10	Epithelial cells	*TET2*	0.2910	0.623	0.238	2.276e-16	5.256e-12
10	Epithelial cells	*HOXB5*	0.2909	0.210	0.010	4.114e-94	9.501e-90
10	Epithelial cells	*FILIP1L*	0.2909	0.558	0.237	3.870e-12	8.938e-08
10	Epithelial cells	*STK38L*	0.2906	0.319	0.077	6.319e-23	1.459e-18
10	Epithelial cells	*OVOL2*	0.2905	0.167	0.000	9.683e-294	2.236e-289
10	Epithelial cells	*ASXL1*	0.2905	0.688	0.335	2.467e-10	5.698e-06
10	Epithelial cells	*HSD17B12*	0.2904	0.464	0.183	3.532e-13	8.155e-09
10	Epithelial cells	*RNGTT*	0.2903	0.435	0.173	1.817e-12	4.195e-08
10	Epithelial cells	*SCYL2*	0.2901	0.471	0.154	2.810e-18	6.489e-14
10	Epithelial cells	*NDUFV3*	0.2899	0.522	0.166	7.199e-21	1.662e-16
10	Epithelial cells	*CISH*	0.2898	0.326	0.072	2.204e-26	5.090e-22
10	Epithelial cells	*EXOC6*	0.2898	0.348	0.102	2.930e-18	6.765e-14
10	Epithelial cells	*REST*	0.2897	0.399	0.092	4.327e-29	9.993e-25
10	Epithelial cells	*LOC100358069*	0.2896	0.891	0.660	1.603e-06	3.701e-02
10	Epithelial cells	*LOC100354896*	0.2896	0.232	0.005	5.590e-185	1.291e-180
10	Epithelial cells	*QKI*	0.2896	0.754	0.414	7.769e-08	1.794e-03
10	Epithelial cells	*USP3*	0.2893	0.500	0.172	3.308e-17	7.640e-13
10	Epithelial cells	*FAM131C*	0.2893	0.225	0.001	0	0
10	Epithelial cells	*PEX10*	0.2891	0.362	0.110	3.328e-16	7.686e-12
10	Epithelial cells	*GIGYF2*	0.2890	0.587	0.279	2.152e-09	4.971e-05
10	Epithelial cells	*CLCN3*	0.2890	0.413	0.115	2.330e-22	5.380e-18
10	Epithelial cells	*TMEM170A*	0.2890	0.471	0.138	2.490e-22	5.750e-18
10	Epithelial cells	*APC*	0.2889	0.630	0.352	1.248e-06	2.881e-02
10	Epithelial cells	*HELQ*	0.2888	0.254	0.078	1.297e-12	2.996e-08
10	Epithelial cells	*ETNK1*	0.2887	0.703	0.389	7.041e-08	1.626e-03
10	Epithelial cells	*RPRD2*	0.2884	0.558	0.251	1.592e-11	3.676e-07

Original cluster	Annotated cell type	Gene	avg_log2FC	pct.1	pct.2	Raw P value	Bonferroni-adjusted P value
10	Epithelial cells	*CCNYL1*	0.2883	0.348	0.088	8.319e-23	1.921e-18
10	Epithelial cells	*HMGA1*	0.2881	0.210	0.005	1.270e-147	2.933e-143
10	Epithelial cells	*ATG13*	0.2880	0.370	0.096	5.345e-22	1.234e-17
10	Epithelial cells	*PPP1R13L*	0.2880	0.217	0.012	1.922e-83	4.438e-79
10	Epithelial cells	*AMFR*	0.2880	0.428	0.146	1.971e-15	4.552e-11
10	Epithelial cells	*SPICE1*	0.2877	0.493	0.174	1.297e-15	2.995e-11
10	Epithelial cells	*CLU*	0.2873	0.565	0.247	4.042e-11	9.335e-07
10	Epithelial cells	*LOC108177085*	0.2870	0.210	0.013	1.916e-72	4.424e-68
10	Epithelial cells	*SAMD11*	0.2870	0.225	0.018	2.989e-62	6.903e-58
10	Epithelial cells	*TRMT13*	0.2869	0.384	0.123	1.071e-16	2.474e-12
10	Epithelial cells	*TRIM24*	0.2868	0.391	0.111	4.817e-20	1.112e-15
10	Epithelial cells	*IAH1*	0.2866	0.304	0.044	7.601e-43	1.755e-38
10	Epithelial cells	*WHAMM*	0.2863	0.326	0.061	9.017e-33	2.082e-28
10	Epithelial cells	*MAFG*	0.2863	0.326	0.069	1.057e-27	2.440e-23
10	Epithelial cells	*CDK17*	0.2862	0.449	0.167	1.967e-14	4.543e-10
10	Epithelial cells	*TMED5*	0.2861	0.543	0.210	6.430e-15	1.485e-10
10	Epithelial cells	*UBR1*	0.2860	0.609	0.274	2.121e-11	4.898e-07
10	Epithelial cells	*HS3ST5*	0.2857	0.203	0.001	0	0
10	Epithelial cells	*TNC*	0.2854	0.225	0.005	1.874e-173	4.326e-169
10	Epithelial cells	*GMPS*	0.2854	0.522	0.187	1.762e-16	4.069e-12
10	Epithelial cells	*MTMR3*	0.2853	0.486	0.198	1.339e-12	3.092e-08
10	Epithelial cells	*UBALD1*	0.2853	0.268	0.040	2.309e-36	5.331e-32
10	Epithelial cells	*B2M*	0.2851	0.949	0.877	1.309e-07	3.022e-03
10	Epithelial cells	*GPATCH2*	0.2847	0.377	0.153	1.358e-10	3.137e-06
10	Epithelial cells	*ZNF106*	0.2845	0.551	0.235	2.902e-12	6.700e-08
10	Epithelial cells	*FLOT2*	0.2844	0.391	0.098	1.586e-24	3.661e-20
10	Epithelial cells	*DPF2*	0.2843	0.471	0.158	3.487e-17	8.052e-13
10	Epithelial cells	*ITIH5*	0.2843	0.326	0.098	3.557e-16	8.213e-12
10	Epithelial cells	*GORASP2*	0.2841	0.326	0.092	7.851e-18	1.813e-13
10	Epithelial cells	*FBXO38*	0.2839	0.420	0.126	1.278e-19	2.950e-15
10	Epithelial cells	*PHC3*	0.2836	0.558	0.216	1.288e-14	2.973e-10
10	Epithelial cells	*CFAP20DC*	0.2835	0.355	0.136	3.076e-11	7.104e-07
10	Epithelial cells	*TTLL5*	0.2835	0.275	0.075	1.594e-16	3.680e-12
10	Epithelial cells	*SLK*	0.2833	0.638	0.284	1.384e-11	3.195e-07
10	Epithelial cells	*LOC103351327*	0.2832	0.341	0.083	5.308e-23	1.226e-18
10	Epithelial cells	*HSPA4*	0.2832	0.572	0.205	3.737e-17	8.630e-13
10	Epithelial cells	*IDUA*	0.2832	0.420	0.126	1.576e-19	3.639e-15
10	Epithelial cells	*ZNF207*	0.2828	0.580	0.264	1.055e-10	2.436e-06
10	Epithelial cells	*TRAPPC10*	0.2828	0.261	0.045	5.204e-30	1.202e-25
10	Epithelial cells	*STX18*	0.2826	0.399	0.129	2.244e-16	5.182e-12

Original cluster	Annotated cell type	Gene	avg_log2FC	pct.1	pct.2	Raw P value	Bonferroni-adjusted P value
10	Epithelial cells	*PSAT1*	0.2824	0.377	0.080	9.347e-31	2.158e-26
10	Epithelial cells	*THAP12*	0.2824	0.377	0.099	1.881e-22	4.343e-18
10	Epithelial cells	*STRADB*	0.2822	0.304	0.073	3.188e-21	7.362e-17
10	Epithelial cells	*POR*	0.2822	0.377	0.085	5.434e-28	1.255e-23
10	Epithelial cells	*HECTD1*	0.2822	0.565	0.208	9.443e-16	2.181e-11
10	Epithelial cells	*UBXN8*	0.2821	0.297	0.038	4.691e-48	1.083e-43
10	Epithelial cells	*RALBP1*	0.2820	0.601	0.267	5.252e-12	1.213e-07
10	Epithelial cells	*PHF23*	0.2818	0.333	0.090	3.362e-19	7.763e-15
10	Epithelial cells	*PSMD1*	0.2817	0.500	0.176	1.902e-16	4.393e-12
10	Epithelial cells	*SREK1*	0.2817	0.746	0.407	3.063e-09	7.073e-05
10	Epithelial cells	*AP1G1*	0.2810	0.565	0.192	2.443e-19	5.642e-15
10	Epithelial cells	*ZNF800*	0.2809	0.478	0.163	8.115e-17	1.874e-12
10	Epithelial cells	*CHMP2A*	0.2808	0.630	0.299	6.771e-10	1.564e-05
10	Epithelial cells	*POMK*	0.2807	0.283	0.044	1.881e-36	4.345e-32
10	Epithelial cells	*PAIP1*	0.2801	0.659	0.353	2.653e-08	6.125e-04
10	Epithelial cells	*AGK*	0.2798	0.275	0.080	1.037e-14	2.396e-10
10	Epithelial cells	*ZFYVE9*	0.2797	0.536	0.222	1.612e-12	3.722e-08
10	Epithelial cells	*RFC3*	0.2795	0.435	0.123	1.114e-21	2.572e-17
10	Epithelial cells	*GGNBP2*	0.2794	0.609	0.279	5.830e-11	1.346e-06
10	Epithelial cells	*LOC100343764*	0.2793	0.130	0.000	6.017e-261	1.389e-256
10	Epithelial cells	*AMMECR1*	0.2792	0.449	0.218	1.156e-07	2.670e-03
10	Epithelial cells	*CUX2*	0.2791	0.188	0.011	2.583e-68	5.965e-64
10	Epithelial cells	*MTMR1*	0.2790	0.312	0.068	7.537e-26	1.740e-21
10	Epithelial cells	*LOC138843211*	0.2788	0.239	0.015	1.340e-84	3.093e-80
10	Epithelial cells	*PJA2*	0.2786	0.623	0.279	1.945e-11	4.492e-07
10	Epithelial cells	*HIRA*	0.2785	0.333	0.078	4.057e-24	9.367e-20
10	Epithelial cells	*KLHL18*	0.2784	0.377	0.106	5.757e-20	1.329e-15
10	Epithelial cells	*SRI*	0.2782	0.522	0.236	2.781e-10	6.422e-06
10	Epithelial cells	*MYRIP*	0.2782	0.159	0.039	2.866e-12	6.619e-08
10	Epithelial cells	*MBOAT2*	0.2777	0.384	0.104	1.658e-21	3.828e-17
10	Epithelial cells	*LOC127489220*	0.2777	0.217	0.018	1.362e-58	3.145e-54
10	Epithelial cells	*LOC100348238*	0.2775	0.246	0.048	1.963e-24	4.532e-20
10	Epithelial cells	*ZRANB1*	0.2775	0.442	0.143	7.776e-18	1.796e-13
10	Epithelial cells	*THOC1*	0.2774	0.478	0.192	1.342e-12	3.100e-08
10	Epithelial cells	*RAI2*	0.2768	0.362	0.066	9.660e-37	2.231e-32
10	Epithelial cells	*NR2F6*	0.2767	0.399	0.105	3.775e-23	8.717e-19
10	Epithelial cells	*KDM2A*	0.2765	0.638	0.335	3.370e-08	7.782e-04
10	Epithelial cells	*CDC73*	0.2764	0.594	0.258	5.955e-12	1.375e-07
10	Epithelial cells	*PHF11*	0.2763	0.326	0.090	1.770e-18	4.086e-14
10	Epithelial cells	*ACVR1C*	0.2762	0.145	0.021	6.352e-22	1.467e-17

Original cluster	Annotated cell type	Gene	avg_log2FC	pct.1	pct.2	Raw P value	Bonferroni-adjusted P value
10	Epithelial cells	*VMP1*	0.2761	0.558	0.177	8.062e-22	1.862e-17
10	Epithelial cells	*MDM4*	0.2759	0.623	0.327	4.714e-08	1.089e-03
10	Epithelial cells	*LLGL2*	0.2759	0.225	0.002	1.317e-262	3.040e-258
10	Epithelial cells	*ZDHHC5*	0.2758	0.348	0.075	1.405e-28	3.243e-24
10	Epithelial cells	*SERPINE2*	0.2758	0.312	0.070	2.884e-24	6.660e-20
10	Epithelial cells	*SMYD2*	0.2758	0.370	0.071	9.058e-35	2.092e-30
10	Epithelial cells	*HSBP1L1*	0.2757	0.239	0.014	1.211e-89	2.796e-85
10	Epithelial cells	*FGD3*	0.2756	0.138	0.015	5.456e-28	1.260e-23
10	Epithelial cells	*NUP98*	0.2755	0.652	0.321	3.476e-10	8.026e-06
10	Epithelial cells	*BTBD1*	0.2755	0.507	0.198	1.101e-13	2.543e-09
10	Epithelial cells	*TENT5A*	0.2755	0.406	0.124	1.909e-18	4.407e-14
10	Epithelial cells	*TCIM*	0.2754	0.188	0.050	2.887e-12	6.667e-08
10	Epithelial cells	*TTC1*	0.2753	0.355	0.104	1.096e-17	2.530e-13
10	Epithelial cells	*PSPH*	0.2752	0.268	0.052	4.951e-26	1.143e-21
10	Epithelial cells	*VTA1*	0.2752	0.529	0.248	6.310e-10	1.457e-05
10	Epithelial cells	*TTC23L*	0.2751	0.167	0.037	2.619e-14	6.047e-10
10	Epithelial cells	*SCARB2*	0.2751	0.500	0.151	1.858e-21	4.291e-17
10	Epithelial cells	*ABCA2*	0.2750	0.225	0.013	2.005e-84	4.629e-80
10	Epithelial cells	*LENG8*	0.2749	0.283	0.087	1.292e-13	2.983e-09
10	Epithelial cells	*ALDH7A1*	0.2748	0.594	0.234	3.316e-14	7.657e-10
10	Epithelial cells	*PTMA*	0.2745	0.978	0.982	5.042e-09	1.164e-04
10	Epithelial cells	*ATG5*	0.2743	0.464	0.192	1.986e-11	4.587e-07
10	Epithelial cells	*CHD3*	0.2743	0.406	0.116	4.020e-20	9.284e-16
10	Epithelial cells	*MYO1B*	0.2741	0.551	0.208	3.709e-16	8.565e-12
10	Epithelial cells	*SOD2*	0.2740	0.406	0.110	5.876e-23	1.357e-18
10	Epithelial cells	*MCEE*	0.2739	0.391	0.128	4.700e-16	1.085e-11
10	Epithelial cells	*PRR15L*	0.2739	0.174	0.001	4.580e-286	1.058e-281
10	Epithelial cells	*YTHDF2*	0.2738	0.536	0.226	1.393e-11	3.216e-07
10	Epithelial cells	*CST6*	0.2738	0.196	0.001	2.160e-260	4.988e-256
10	Epithelial cells	*NUP93*	0.2732	0.341	0.086	8.299e-22	1.916e-17
10	Epithelial cells	*SLAIN1*	0.2730	0.239	0.017	1.438e-74	3.321e-70
10	Epithelial cells	*MYCBP*	0.2730	0.159	0.024	5.554e-23	1.283e-18
10	Epithelial cells	*TLN2*	0.2729	0.399	0.117	1.535e-19	3.545e-15
10	Epithelial cells	*LOC138847720*	0.2728	0.174	0.033	3.021e-18	6.977e-14
10	Epithelial cells	*METAP1*	0.2726	0.333	0.065	1.127e-31	2.603e-27
10	Epithelial cells	*MED23*	0.2725	0.420	0.154	9.632e-14	2.224e-09
10	Epithelial cells	*GMDS*	0.2725	0.406	0.150	2.779e-13	6.418e-09
10	Epithelial cells	*KCNK1*	0.2722	0.196	0.003	8.054e-180	1.860e-175
10	Epithelial cells	*GFOD1*	0.2721	0.225	0.029	2.656e-37	6.132e-33
10	Epithelial cells	*UBE4A*	0.2719	0.333	0.122	1.950e-11	4.503e-07

Original cluster	Annotated cell type	Gene	avg_log2FC	pct.1	pct.2	Raw P value	Bonferroni-adjusted P value
10	Epithelial cells	*SMG6*	0.2715	0.623	0.299	9.244e-11	2.135e-06
10	Epithelial cells	*SET*	0.2714	0.681	0.326	6.826e-10	1.576e-05
10	Epithelial cells	*UBAP2*	0.2713	0.580	0.216	4.222e-16	9.749e-12
10	Epithelial cells	*EMC6*	0.2712	0.623	0.264	1.492e-12	3.446e-08
10	Epithelial cells	*SPATA6L*	0.2712	0.333	0.082	1.182e-22	2.730e-18
10	Epithelial cells	*CMTM4*	0.2711	0.225	0.022	8.380e-51	1.935e-46
10	Epithelial cells	*LOC138849600*	0.2710	0.312	0.059	1.075e-30	2.482e-26
10	Epithelial cells	*UBASH3B*	0.2708	0.181	0.025	4.192e-28	9.679e-24
10	Epithelial cells	*TJP3*	0.2706	0.203	0.001	0	0
10	Epithelial cells	*LOC138849360*	0.2705	0.370	0.115	1.079e-16	2.492e-12
10	Epithelial cells	*SERINC5*	0.2705	0.333	0.065	1.700e-31	3.926e-27
10	Epithelial cells	*ERICH1*	0.2704	0.304	0.070	1.063e-22	2.454e-18
10	Epithelial cells	*RGL2*	0.2701	0.348	0.113	2.052e-14	4.738e-10
10	Epithelial cells	*B4GALT5*	0.2700	0.304	0.038	2.927e-51	6.758e-47
10	Epithelial cells	*VILL*	0.2700	0.145	0.012	1.974e-38	4.558e-34
10	Epithelial cells	*RASSF4*	0.2697	0.210	0.012	4.155e-77	9.596e-73
10	Epithelial cells	*LOC103351785*	0.2696	0.254	0.041	2.480e-31	5.726e-27
10	Epithelial cells	*TSEN54*	0.2690	0.304	0.050	1.102e-36	2.545e-32
10	Epithelial cells	*CEPT1*	0.2690	0.514	0.212	1.330e-12	3.072e-08
10	Epithelial cells	*RAB11A*	0.2689	0.435	0.124	9.779e-22	2.258e-17
10	Epithelial cells	*HAPSTR1*	0.2689	0.478	0.203	3.461e-11	7.992e-07
10	Epithelial cells	*AP3D1*	0.2688	0.333	0.107	3.368e-14	7.778e-10
10	Epithelial cells	*B3GALNT2*	0.2687	0.232	0.046	2.667e-22	6.158e-18
10	Epithelial cells	*TAF1*	0.2686	0.587	0.292	4.442e-08	1.026e-03
10	Epithelial cells	*UAP1*	0.2686	0.428	0.126	1.266e-20	2.923e-16
10	Epithelial cells	*C18H16orf74*	0.2685	0.239	0.012	1.156e-103	2.669e-99
10	Epithelial cells	*KDM1A*	0.2685	0.543	0.186	8.446e-18	1.950e-13
10	Epithelial cells	*TRIM33*	0.2685	0.522	0.219	5.714e-12	1.320e-07
10	Epithelial cells	*LOC108177222*	0.2685	0.145	0.021	2.061e-21	4.759e-17
10	Epithelial cells	*MAFF*	0.2682	0.333	0.083	3.756e-22	8.674e-18
10	Epithelial cells	*ATRN*	0.2678	0.428	0.143	1.778e-16	4.106e-12
10	Epithelial cells	*LOC138850595*	0.2675	0.348	0.063	1.268e-35	2.929e-31
10	Epithelial cells	*HS3ST1*	0.2675	0.297	0.038	1.708e-48	3.945e-44
10	Epithelial cells	*ABHD2*	0.2675	0.341	0.088	1.324e-21	3.057e-17
10	Epithelial cells	*BRD3*	0.2674	0.420	0.157	5.013e-13	1.158e-08
10	Epithelial cells	*SUPT20H*	0.2674	0.594	0.268	5.192e-11	1.199e-06
10	Epithelial cells	*ZNF407*	0.2674	0.478	0.221	2.204e-09	5.089e-05
10	Epithelial cells	*PTPN11*	0.2669	0.348	0.091	1.446e-21	3.339e-17
10	Epithelial cells	*QNG1*	0.2669	0.449	0.133	1.781e-20	4.113e-16
10	Epithelial cells	*KDM5B*	0.2668	0.645	0.275	1.004e-12	2.318e-08

Original cluster	Annotated cell type	Gene	avg_log2FC	pct.1	pct.2	Raw P value	Bonferroni-adjusted P value
10	Epithelial cells	*CBS*	0.2668	0.326	0.082	6.958e-21	1.607e-16
10	Epithelial cells	*CHM*	0.2666	0.435	0.168	3.698e-12	8.540e-08
10	Epithelial cells	*FNDC3A*	0.2665	0.768	0.484	1.500e-06	3.464e-02
10	Epithelial cells	*TBC1D23*	0.2658	0.442	0.188	1.191e-10	2.750e-06
10	Epithelial cells	*LCLAT1*	0.2656	0.399	0.160	1.038e-10	2.396e-06
10	Epithelial cells	*UST*	0.2653	0.471	0.171	3.969e-15	9.165e-11
10	Epithelial cells	*SLC6A1*	0.2651	0.109	0.003	4.641e-81	1.072e-76
10	Epithelial cells	*FOCAD*	0.2649	0.312	0.133	6.405e-08	1.479e-03
10	Epithelial cells	*HOXB9*	0.2648	0.116	0.005	1.509e-57	3.485e-53
10	Epithelial cells	*DCAF5*	0.2648	0.377	0.120	2.742e-16	6.332e-12
10	Epithelial cells	*XIAP*	0.2648	0.370	0.125	4.328e-14	9.994e-10
10	Epithelial cells	*SMARCAD1*	0.2646	0.370	0.127	6.837e-14	1.579e-09
10	Epithelial cells	*PELI1*	0.2640	0.522	0.248	5.824e-10	1.345e-05
10	Epithelial cells	*SEMA6A*	0.2640	0.355	0.089	9.515e-23	2.197e-18
10	Epithelial cells	*PANK2*	0.2637	0.442	0.154	2.797e-15	6.458e-11
10	Epithelial cells	*CD200*	0.2635	0.449	0.174	1.326e-12	3.061e-08
10	Epithelial cells	*ATP11C*	0.2635	0.507	0.254	6.580e-08	1.519e-03
10	Epithelial cells	*LAP3*	0.2635	0.616	0.248	1.051e-13	2.426e-09
10	Epithelial cells	*LOC138844383*	0.2634	0.138	0.001	2.085e-166	4.816e-162
10	Epithelial cells	*YPEL2*	0.2633	0.341	0.074	1.590e-27	3.672e-23
10	Epithelial cells	*TMEM131*	0.2633	0.507	0.183	7.581e-16	1.751e-11
10	Epithelial cells	*NUAK2*	0.2633	0.188	0.014	8.904e-57	2.056e-52
10	Epithelial cells	*UGT1A7*	0.2632	0.217	0.025	1.362e-41	3.144e-37
10	Epithelial cells	*DCAF11*	0.2631	0.188	0.044	3.915e-15	9.040e-11
10	Epithelial cells	*NMT1*	0.2630	0.442	0.149	2.289e-16	5.285e-12
10	Epithelial cells	*PPP4R2*	0.2630	0.717	0.348	3.653e-10	8.435e-06
10	Epithelial cells	*ELK4*	0.2629	0.507	0.172	2.174e-17	5.021e-13
10	Epithelial cells	*SPCS3*	0.2628	0.580	0.267	1.547e-09	3.573e-05
10	Epithelial cells	*FRY*	0.2624	0.493	0.148	8.690e-24	2.007e-19
10	Epithelial cells	*LRP5*	0.2622	0.326	0.068	3.935e-28	9.087e-24
10	Epithelial cells	*GMCL1*	0.2621	0.457	0.165	7.267e-15	1.678e-10
10	Epithelial cells	*CDC42SE2*	0.2620	0.326	0.089	5.394e-19	1.246e-14
10	Epithelial cells	*RSU1*	0.2619	0.652	0.324	2.672e-09	6.170e-05
10	Epithelial cells	*HIPK3*	0.2618	0.674	0.342	4.524e-10	1.045e-05
10	Epithelial cells	*FIG4*	0.2618	0.348	0.090	1.680e-21	3.878e-17
10	Epithelial cells	*FRMD5*	0.2616	0.181	0.026	1.336e-26	3.084e-22
10	Epithelial cells	*LOC100348995*	0.2615	0.210	0.005	2.491e-151	5.752e-147
10	Epithelial cells	*LOXL1*	0.2609	0.290	0.048	2.950e-34	6.813e-30
10	Epithelial cells	*NPAS2*	0.2608	0.174	0.007	2.998e-85	6.923e-81
10	Epithelial cells	*BARD1*	0.2607	0.406	0.146	2.979e-13	6.880e-09

Original cluster	Annotated cell type	Gene	avg_log2FC	pct.1	pct.2	Raw P value	Bonferroni-adjusted P value
10	Epithelial cells	*FBP1*	0.2604	0.188	0.003	4.167e-191	9.623e-187
10	Epithelial cells	*ALMS1*	0.2604	0.514	0.203	2.141e-13	4.945e-09
10	Epithelial cells	*NEXMIF*	0.2597	0.159	0.009	3.868e-64	8.932e-60
10	Epithelial cells	*CHCHD6*	0.2597	0.435	0.147	5.661e-16	1.307e-11
10	Epithelial cells	*TET3*	0.2596	0.283	0.045	4.632e-35	1.070e-30
10	Epithelial cells	*MISP3*	0.2596	0.217	0.028	2.475e-35	5.715e-31
10	Epithelial cells	*ELOVL6*	0.2592	0.290	0.044	1.916e-38	4.424e-34
10	Epithelial cells	*PIK3R4*	0.2592	0.333	0.102	1.617e-15	3.734e-11
10	Epithelial cells	*GALNT3*	0.2590	0.210	0.012	4.298e-77	9.925e-73
10	Epithelial cells	*NFATC1*	0.2590	0.232	0.040	4.253e-27	9.822e-23
10	Epithelial cells	*THADA*	0.2582	0.413	0.154	2.012e-13	4.646e-09
10	Epithelial cells	*TRAPPC6A*	0.2580	0.341	0.072	1.780e-28	4.111e-24
10	Epithelial cells	*IARS2*	0.2578	0.362	0.099	3.218e-20	7.432e-16
10	Epithelial cells	*PPFIBP1*	0.2578	0.493	0.169	7.776e-17	1.796e-12
10	Epithelial cells	*AUH*	0.2578	0.674	0.394	1.600e-06	3.695e-02
10	Epithelial cells	*BLNK*	0.2578	0.225	0.007	8.406e-137	1.941e-132
10	Epithelial cells	*SRF*	0.2578	0.319	0.101	2.291e-14	5.291e-10
10	Epithelial cells	*PPP6R1*	0.2576	0.254	0.069	2.257e-15	5.212e-11
10	Epithelial cells	*PHF13*	0.2574	0.232	0.042	1.630e-24	3.763e-20
10	Epithelial cells	*CSNK1G3*	0.2573	0.471	0.206	4.641e-10	1.072e-05
10	Epithelial cells	*ELOA*	0.2571	0.225	0.061	1.357e-13	3.133e-09
10	Epithelial cells	*AFG2A*	0.2570	0.319	0.100	1.248e-14	2.883e-10
10	Epithelial cells	*ARFIP1*	0.2570	0.522	0.174	1.267e-18	2.925e-14
10	Epithelial cells	*RPGR*	0.2567	0.370	0.138	2.949e-12	6.809e-08
10	Epithelial cells	*SLC25A30*	0.2567	0.384	0.122	5.993e-16	1.384e-11
10	Epithelial cells	*TNPO3*	0.2566	0.406	0.139	8.913e-15	2.058e-10
10	Epithelial cells	*MAP4*	0.2566	0.572	0.274	4.147e-09	9.576e-05
10	Epithelial cells	*ATXN10*	0.2565	0.399	0.102	2.905e-24	6.709e-20
10	Epithelial cells	*ISCA2*	0.2565	0.428	0.195	4.431e-08	1.023e-03
10	Epithelial cells	*AGPS*	0.2563	0.341	0.093	3.107e-19	7.175e-15
10	Epithelial cells	*PARP12*	0.2562	0.232	0.046	1.153e-22	2.661e-18
10	Epithelial cells	*LOC100352865*	0.2559	0.217	0.037	1.090e-25	2.518e-21
10	Epithelial cells	*RPE*	0.2556	0.239	0.053	8.436e-20	1.948e-15
10	Epithelial cells	*FOXP4*	0.2550	0.203	0.010	3.650e-89	8.429e-85
10	Epithelial cells	*TARS2*	0.2547	0.275	0.081	5.355e-14	1.237e-09
10	Epithelial cells	*PATZ1*	0.2539	0.348	0.076	1.248e-27	2.882e-23
10	Epithelial cells	*SPCS1*	0.2538	0.659	0.323	2.711e-08	6.259e-04
10	Epithelial cells	*AKAP8L*	0.2538	0.486	0.189	4.470e-12	1.032e-07
10	Epithelial cells	*KDF1*	0.2536	0.116	0.000	3.462e-218	7.995e-214
10	Epithelial cells	*NR3C1*	0.2534	0.754	0.421	8.046e-07	1.858e-02

Original cluster	Annotated cell type	Gene	avg_log2FC	pct.1	pct.2	Raw P value	Bonferroni-adjusted P value
10	Epithelial cells	*CSNK1D*	0.2532	0.435	0.142	9.084e-17	2.098e-12
10	Epithelial cells	*PDIA5*	0.2532	0.268	0.038	4.651e-39	1.074e-34
10	Epithelial cells	*TBCK*	0.2532	0.377	0.173	5.419e-08	1.251e-03
10	Epithelial cells	*KRTCAP3*	0.2531	0.167	0.005	6.793e-101	1.569e-96
10	Epithelial cells	*NOP14*	0.2529	0.159	0.041	4.644e-11	1.072e-06
10	Epithelial cells	*TAP2*	0.2525	0.246	0.050	6.958e-23	1.607e-18
10	Epithelial cells	*ADAMTS6*	0.2525	0.174	0.018	6.910e-38	1.596e-33
10	Epithelial cells	*MYH14*	0.2524	0.196	0.004	2.601e-173	6.006e-169
10	Epithelial cells	*CARF*	0.2523	0.333	0.072	4.892e-27	1.130e-22
10	Epithelial cells	*TCIRG1*	0.2522	0.174	0.062	2.975e-07	6.869e-03
10	Epithelial cells	*ITGA3*	0.2521	0.246	0.023	1.039e-57	2.399e-53
10	Epithelial cells	*WDR70*	0.2521	0.435	0.172	7.478e-12	1.727e-07
10	Epithelial cells	*MARK3*	0.2516	0.464	0.178	3.497e-13	8.076e-09
10	Epithelial cells	*MAFA*	0.2516	0.217	0.023	1.312e-44	3.031e-40
10	Epithelial cells	*ARHGEF26*	0.2515	0.217	0.051	9.399e-17	2.170e-12
10	Epithelial cells	*METTL9*	0.2514	0.493	0.157	2.184e-19	5.044e-15
10	Epithelial cells	*KIAA0825*	0.2514	0.413	0.143	2.638e-15	6.092e-11
10	Epithelial cells	*AEBP2*	0.2513	0.587	0.242	1.344e-12	3.104e-08
10	Epithelial cells	*WASL*	0.2510	0.652	0.335	1.181e-08	2.728e-04
10	Epithelial cells	*DPP6*	0.2509	0.152	0.038	3.981e-11	9.193e-07
10	Epithelial cells	*CWF19L2*	0.2509	0.529	0.220	2.832e-12	6.541e-08
10	Epithelial cells	*NXF1*	0.2506	0.188	0.055	2.906e-10	6.710e-06
10	Epithelial cells	*PDCD10*	0.2504	0.565	0.225	2.080e-13	4.804e-09
10	Epithelial cells	*CPNE1*	0.2503	0.638	0.299	6.288e-10	1.452e-05
10	Epithelial cells	*NAA30*	0.2500	0.362	0.094	3.497e-22	8.074e-18
9	**Neutrophils**	*S100A9*	8.6872	0.552	0.020	0	0
9	Neutrophils	*LOC100008703*	7.6925	0.455	0.007	0	0
9	Neutrophils	*S100A8*	7.6729	0.552	0.010	0	0
9	Neutrophils	*LCN2*	5.5472	0.299	0.007	8.411e-229	1.942e-224
9	Neutrophils	*PGLYRP1*	5.4019	0.478	0.002	0	0
9	Neutrophils	*SAMSN1*	4.9546	0.776	0.033	0	0
9	Neutrophils	*IL1B*	4.8510	0.627	0.004	0	0
9	Neutrophils	*TYROBP*	4.7900	0.813	0.003	0	0
9	Neutrophils	*RLA-DRB1*	4.7196	0.463	0.004	0	0
9	Neutrophils	*ANXA1*	4.4236	0.821	0.474	4.907e-42	1.133e-37
9	Neutrophils	*LOC127482571*	4.4225	0.418	0.004	0	0
9	Neutrophils	*CXCL8*	4.2523	0.634	0.007	0	0
9	Neutrophils	*LOC100351163*	4.1995	0.619	0.061	3.089e-156	7.133e-152
9	Neutrophils	*LOC100009166*	4.1121	0.425	0.030	1.268e-139	2.929e-135
9	Neutrophils	*CD74*	4.0928	0.485	0.004	0	0

Original cluster	Annotated cell type	Gene	avg_log2FC	pct.1	pct.2	Raw P value	Bonferroni-adjusted P value
9	Neutrophils	*ARHGAP15*	4.0264	0.761	0.026	0	0
9	Neutrophils	*CCL2*	3.9842	0.194	0.006	2.119e-123	4.892e-119
9	Neutrophils	*RLA-DR-ALPHA*	3.9560	0.455	0.003	0	0
9	Neutrophils	*CAMP*	3.8416	0.313	0.002	0	0
9	Neutrophils	*C7H1orf162*	3.7660	0.634	0.001	0	0
9	Neutrophils	*SRGN*	3.7313	0.799	0.051	2.190e-294	5.056e-290
9	Neutrophils	*LCP1*	3.7222	0.903	0.025	0	0
9	Neutrophils	*BCL2A1*	3.4582	0.746	0.007	0	0
9	Neutrophils	*PLAC8*	3.4087	0.552	0.002	0	0
9	Neutrophils	*MXD1*	3.3673	0.694	0.147	5.976e-81	1.380e-76
9	Neutrophils	*PDE4D*	3.2583	0.619	0.176	8.049e-46	1.859e-41
9	Neutrophils	*LOC127493681*	3.2468	0.463	0.020	7.444e-227	1.719e-222
9	Neutrophils	*MEGF9*	3.1633	0.590	0.048	1.950e-172	4.503e-168
9	Neutrophils	*FCER1G*	3.1574	0.701	0.012	0	0
9	Neutrophils	*LOC100348776*	3.1426	0.201	0.002	8.455e-263	1.952e-258
9	Neutrophils	*CYTIP*	3.1107	0.649	0.028	0	0
9	Neutrophils	*PTPRC*	3.0867	0.881	0.016	0	0
9	Neutrophils	*LOC138847548*	3.0762	0.485	0.005	0	0
9	Neutrophils	*IPCEF1*	3.0504	0.545	0.039	1.653e-176	3.816e-172
9	Neutrophils	*DOCK8*	3.0499	0.799	0.037	0	0
9	Neutrophils	*ELMO1*	3.0221	0.813	0.105	1.759e-160	4.063e-156
9	Neutrophils	*HP*	2.9865	0.366	0.005	0	0
9	Neutrophils	*IDO1*	2.9841	0.261	0.001	0	0
9	Neutrophils	*NR4A3*	2.9509	0.537	0.140	6.472e-45	1.495e-40
9	Neutrophils	*LOC100351888*	2.9450	0.731	0.002	0	0
9	Neutrophils	*S100A11*	2.9430	0.806	0.752	5.912e-18	1.365e-13
9	Neutrophils	*CD37*	2.9281	0.806	0.045	0	0
9	Neutrophils	*LYN*	2.8901	0.791	0.025	0	0
9	Neutrophils	*LOC103347892*	2.8845	0.701	0.042	2.971e-270	6.860e-266
9	Neutrophils	*RGS2*	2.8614	0.791	0.215	1.071e-78	2.474e-74
9	Neutrophils	*PFKFB3*	2.8474	0.470	0.009	0	0
9	Neutrophils	*CORO1A*	2.8127	0.701	0.013	0	0
9	Neutrophils	*ATP8B4*	2.7859	0.694	0.012	0	0
9	Neutrophils	*LOC100350534*	2.7856	0.582	0.029	5.198e-258	1.200e-253
9	Neutrophils	*PFN1*	2.7708	0.896	0.557	2.050e-50	4.734e-46
9	Neutrophils	*SKAP2*	2.7567	0.754	0.035	0	0
9	Neutrophils	*AIF1*	2.7563	0.381	0.001	0	0
9	Neutrophils	*DOCK2*	2.7509	0.761	0.016	0	0
9	Neutrophils	*CD48*	2.7463	0.709	0.017	0	0
9	Neutrophils	*STK17B*	2.6999	0.701	0.032	0	0

Original cluster	Annotated cell type	Gene	avg_log2FC	pct.1	pct.2	Raw P value	Bonferroni-adjusted P value
9	Neutrophils	*LOC100328967*	2.6952	0.858	0.271	4.896e-77	1.131e-72
9	Neutrophils	*SSH2*	2.6869	0.746	0.190	3.050e-68	7.044e-64
9	Neutrophils	*PICALM*	2.6191	0.828	0.281	2.148e-60	4.960e-56
9	Neutrophils	*MS4A6A*	2.6131	0.552	0.001	0	0
9	Neutrophils	*CD83*	2.5686	0.336	0.055	6.063e-46	1.400e-41
9	Neutrophils	*ALOX5AP*	2.5621	0.634	0.005	0	0
9	Neutrophils	*LYST*	2.5514	0.776	0.289	1.488e-50	3.437e-46
9	Neutrophils	*NEDD9*	2.5468	0.709	0.109	6.774e-113	1.564e-108
9	Neutrophils	*ARPC1B*	2.5427	0.754	0.120	1.001e-124	2.311e-120
9	Neutrophils	*RUNX1*	2.5422	0.709	0.034	0	0
9	Neutrophils	*TSPO*	2.5417	0.694	0.207	3.377e-56	7.797e-52
9	Neutrophils	*MALT1*	2.5411	0.612	0.087	7.580e-105	1.750e-100
9	Neutrophils	*ARHGAP24*	2.5297	0.403	0.052	1.069e-70	2.469e-66
9	Neutrophils	*BRAF*	2.5154	0.791	0.329	1.282e-41	2.959e-37
9	Neutrophils	*LAPTM5*	2.4831	0.761	0.013	0	0
9	Neutrophils	*CSGALNACT2*	2.4712	0.769	0.154	1.402e-96	3.238e-92
9	Neutrophils	*CYRIB*	2.4658	0.843	0.128	7.133e-144	1.647e-139
9	Neutrophils	*PYGL*	2.4596	0.500	0.016	0	0
9	Neutrophils	*MCTP1*	2.4587	0.485	0.033	9.109e-160	2.103e-155
9	Neutrophils	*RNF24*	2.4416	0.425	0.099	1.251e-39	2.890e-35
9	Neutrophils	*PLEK*	2.4408	0.694	0.135	4.565e-86	1.054e-81
9	Neutrophils	*ACTR3*	2.4247	0.888	0.263	4.412e-86	1.019e-81
9	Neutrophils	*B2M*	2.4183	0.948	0.877	1.257e-43	2.904e-39
9	Neutrophils	*LOC100350165*	2.4163	0.366	0.000	0	0
9	Neutrophils	*TMSB4X*	2.4109	0.978	0.975	1.323e-53	3.055e-49
9	Neutrophils	*DUSP1*	2.4038	0.866	0.450	5.354e-48	1.236e-43
9	Neutrophils	*TUT7*	2.3902	0.575	0.116	2.292e-64	5.294e-60
9	Neutrophils	*ANKRD44*	2.3867	0.813	0.196	1.569e-84	3.624e-80
9	Neutrophils	*ARHGDIB*	2.3733	0.799	0.089	4.974e-183	1.148e-178
9	Neutrophils	*RAB8B*	2.3621	0.828	0.300	5.992e-57	1.384e-52
9	Neutrophils	*PECAM1*	2.3401	0.687	0.040	3.723e-261	8.597e-257
9	Neutrophils	*VAV3*	2.3199	0.746	0.111	7.248e-122	1.674e-117
9	Neutrophils	*EMB*	2.3120	0.575	0.003	0	0
9	Neutrophils	*ADK*	2.3046	0.761	0.275	8.673e-45	2.003e-40
9	Neutrophils	*ETV6*	2.3016	0.672	0.179	8.064e-54	1.862e-49
9	Neutrophils	*GCA*	2.2953	0.575	0.049	1.811e-157	4.183e-153
9	Neutrophils	*UCP2*	2.2933	0.769	0.030	0	0
9	Neutrophils	*SYNDIG1*	2.2725	0.590	0.001	0	0
9	Neutrophils	*CD44*	2.2722	0.701	0.050	2.509e-228	5.793e-224
9	Neutrophils	*GMFG*	2.2715	0.746	0.036	0	0

Original cluster	Annotated cell type	Gene	avg_log2FC	pct.1	pct.2	Raw P value	Bonferroni-adjusted P value
9	Neutrophils	*BAZ1A*	2.2645	0.784	0.053	2.163e-271	4.995e-267
9	Neutrophils	*BASP1*	2.2615	0.493	0.044	3.282e-129	7.578e-125
9	Neutrophils	*ITGAM*	2.2568	0.560	0.003	0	0
9	Neutrophils	*ARPC2*	2.2519	0.851	0.400	1.757e-56	4.058e-52
9	Neutrophils	*CCL4*	2.2487	0.209	0.013	8.074e-72	1.864e-67
9	Neutrophils	*RIPOR2*	2.2432	0.619	0.112	2.309e-81	5.333e-77
9	Neutrophils	*CTSK*	2.2276	0.567	0.271	2.174e-22	5.021e-18
9	Neutrophils	*SMAP2*	2.2209	0.746	0.055	7.319e-237	1.690e-232
9	Neutrophils	*CTSS*	2.2201	0.478	0.035	1.773e-152	4.095e-148
9	Neutrophils	*TMEM154*	2.2147	0.463	0.012	0	0
9	Neutrophils	*TMSB10*	2.2134	0.866	0.962	1.553e-33	3.587e-29
9	Neutrophils	*FYB1*	2.2102	0.709	0.015	0	0
9	Neutrophils	*CXCR4*	2.1988	0.560	0.040	1.798e-178	4.153e-174
9	Neutrophils	*ANXA2*	2.1860	0.552	0.169	3.093e-40	7.142e-36
9	Neutrophils	*PPM1H*	2.1737	0.575	0.029	5.534e-252	1.278e-247
9	Neutrophils	*GRO-A*	2.1660	0.179	0.040	2.822e-16	6.517e-12
9	Neutrophils	*SELPLG*	2.1494	0.634	0.008	0	0
9	Neutrophils	*PLAUR*	2.1439	0.642	0.025	0	0
9	Neutrophils	*CCL14*	2.1409	0.478	0.001	0	0
9	Neutrophils	*PPP3CA*	2.1358	0.806	0.429	5.106e-32	1.179e-27
9	Neutrophils	*SORL1*	2.1218	0.694	0.070	1.518e-162	3.505e-158
9	Neutrophils	*ATP11B*	2.1183	0.627	0.184	2.346e-44	5.418e-40
9	Neutrophils	*SH3GLB1*	2.1129	0.828	0.313	2.148e-51	4.960e-47
9	Neutrophils	*LYZ*	2.1092	0.418	0.001	0	0
9	Neutrophils	*SAT1*	2.0895	0.903	0.415	4.835e-54	1.117e-49
9	Neutrophils	*LSP1*	2.0794	0.642	0.171	6.030e-59	1.392e-54
9	Neutrophils	*DOCK11*	2.0781	0.701	0.053	4.235e-214	9.780e-210
9	Neutrophils	*HCST*	2.0680	0.440	0.014	1.046e-258	2.416e-254
9	Neutrophils	*CD53*	2.0556	0.672	0.014	0	0
9	Neutrophils	*RCSD1*	2.0406	0.694	0.090	1.257e-126	2.903e-122
9	Neutrophils	*WNK1*	2.0292	0.731	0.334	1.674e-30	3.866e-26
9	Neutrophils	*C3*	2.0267	0.403	0.008	0	0
9	Neutrophils	*RAB27A*	2.0246	0.575	0.052	1.665e-151	3.846e-147
9	Neutrophils	*MSRA*	2.0183	0.493	0.111	2.094e-47	4.836e-43
9	Neutrophils	*VSIR*	1.9996	0.627	0.058	1.374e-161	3.172e-157
9	Neutrophils	*CAP1*	1.9913	0.776	0.206	1.141e-70	2.635e-66
9	Neutrophils	*NIN*	1.9637	0.627	0.074	1.294e-125	2.987e-121
9	Neutrophils	*ARL15*	1.9627	0.791	0.330	5.332e-36	1.231e-31
9	Neutrophils	*STOM*	1.9598	0.463	0.202	3.501e-14	8.083e-10
9	Neutrophils	*VASP*	1.9524	0.664	0.100	9.589e-106	2.214e-101

Original cluster	Annotated cell type	Gene	avg_log2FC	pct.1	pct.2	Raw P value	Bonferroni-adjusted P value
9	Neutrophils	*GNG2*	1.9418	0.784	0.183	3.190e-80	7.367e-76
9	Neutrophils	*DENND4A*	1.9400	0.724	0.250	3.320e-43	7.667e-39
9	Neutrophils	*F13A1*	1.9351	0.321	0.014	3.240e-149	7.482e-145
9	Neutrophils	*ACTB*	1.9335	0.970	0.972	1.620e-43	3.741e-39
9	Neutrophils	*RFLNB*	1.9294	0.440	0.057	4.857e-78	1.122e-73
9	Neutrophils	*IRAK3*	1.9279	0.507	0.096	1.015e-60	2.343e-56
9	Neutrophils	*REL*	1.9278	0.478	0.063	8.536e-83	1.971e-78
9	Neutrophils	*C1QC*	1.9267	0.104	0.001	1.101e-145	2.542e-141
9	Neutrophils	*APH1B*	1.9229	0.604	0.080	1.963e-108	4.534e-104
9	Neutrophils	*PRKCB*	1.9218	0.657	0.012	0	0
9	Neutrophils	*UBE2H*	1.9212	0.716	0.311	2.571e-29	5.937e-25
9	Neutrophils	*ASAP1*	1.9176	0.746	0.312	1.368e-34	3.160e-30
9	Neutrophils	*ARG1*	1.9174	0.179	0.001	5.305e-285	1.225e-280
9	Neutrophils	*LTF*	1.9150	0.254	0.000	0	0
9	Neutrophils	*LTA4H*	1.9064	0.470	0.127	2.166e-33	5.002e-29
9	Neutrophils	*IGSF6*	1.9053	0.560	0.002	0	0
9	Neutrophils	*AOAH*	1.9015	0.537	0.010	0	0
9	Neutrophils	*IFI30*	1.9001	0.358	0.020	1.352e-138	3.123e-134
9	Neutrophils	*ADAM19*	1.8949	0.440	0.035	3.174e-125	7.329e-121
9	Neutrophils	*FRY*	1.8931	0.597	0.146	1.210e-51	2.793e-47
9	Neutrophils	*TLR4*	1.8917	0.590	0.025	8.891e-293	2.053e-288
9	Neutrophils	*IFNGR1*	1.8910	0.687	0.160	2.713e-70	6.266e-66
9	Neutrophils	*SOCS6*	1.8850	0.537	0.093	1.472e-68	3.400e-64
9	Neutrophils	*TFDP2*	1.8843	0.612	0.379	2.788e-15	6.437e-11
9	Neutrophils	*RAC2*	1.8841	0.672	0.012	0	0
9	Neutrophils	*APOBEC1*	1.8819	0.448	0.000	0	0
9	Neutrophils	*LBR*	1.8806	0.739	0.140	3.473e-96	8.019e-92
9	Neutrophils	*IRAG2*	1.8804	0.515	0.013	0	0
9	Neutrophils	*TRIB1*	1.8793	0.612	0.028	2.507e-290	5.789e-286
9	Neutrophils	*CTSB*	1.8753	0.493	0.299	4.287e-10	9.899e-06
9	Neutrophils	*OSM*	1.8732	0.343	0.002	0	0
9	Neutrophils	*RLA-DMA*	1.8643	0.425	0.012	3.041e-287	7.023e-283
9	Neutrophils	*CHIT1*	1.8577	0.515	0.001	0	0
9	Neutrophils	*FGD4*	1.8532	0.627	0.081	1.077e-115	2.488e-111
9	Neutrophils	*KLF5*	1.8522	0.358	0.139	1.767e-15	4.080e-11
9	Neutrophils	*SPI1*	1.8482	0.642	0.001	0	0
9	Neutrophils	*HMGB2*	1.8382	0.821	0.739	8.721e-07	2.014e-02
9	Neutrophils	*SH3BGRL3*	1.8362	0.739	0.206	1.595e-63	3.684e-59
9	Neutrophils	*BBLN*	1.8323	0.649	0.095	6.814e-104	1.573e-99
9	Neutrophils	*SELL*	1.8296	0.463	0.003	0	0

Original cluster	Annotated cell type	Gene	avg_log2FC	pct.1	pct.2	Raw P value	Bonferroni-adjusted P value
9	Neutrophils	*COTL1*	1.8283	0.701	0.128	2.958e-95	6.831e-91
9	Neutrophils	*DIAPH2*	1.8278	0.821	0.414	1.581e-36	3.650e-32
9	Neutrophils	*ATP8A1*	1.8183	0.590	0.092	3.212e-84	7.418e-80
9	Neutrophils	*FAM107B*	1.8116	0.701	0.193	2.306e-57	5.326e-53
9	Neutrophils	*MPEG1*	1.8110	0.336	0.001	0	0
9	Neutrophils	*ST8SIA4*	1.8085	0.463	0.073	6.589e-64	1.521e-59
9	Neutrophils	*CYBA*	1.7985	0.657	0.177	1.103e-56	2.546e-52
9	Neutrophils	*DOCK5*	1.7946	0.515	0.039	9.210e-152	2.127e-147
9	Neutrophils	*TMEM164*	1.7926	0.410	0.065	6.377e-58	1.472e-53
9	Neutrophils	*SYNE1*	1.7912	0.724	0.445	1.005e-16	2.322e-12
9	Neutrophils	*ARHGAP26*	1.7843	0.582	0.064	1.562e-122	3.608e-118
9	Neutrophils	*IL32*	1.7736	0.216	0.015	2.515e-70	5.808e-66
9	Neutrophils	*PIP4K2A*	1.7686	0.843	0.306	9.211e-51	2.127e-46
9	Neutrophils	*PKIB*	1.7642	0.396	0.015	7.758e-210	1.791e-205
9	Neutrophils	*RASA2*	1.7540	0.649	0.293	1.415e-23	3.269e-19
9	Neutrophils	*LOC100349085*	1.7521	0.634	0.298	1.840e-21	4.249e-17
9	Neutrophils	*PLCL2*	1.7481	0.500	0.077	1.667e-71	3.850e-67
9	Neutrophils	*PDLIM5*	1.7473	0.672	0.234	4.136e-37	9.550e-33
9	Neutrophils	*TRPS1*	1.7468	0.634	0.303	5.299e-22	1.224e-17
9	Neutrophils	*SH3KBP1*	1.7362	0.761	0.301	1.618e-39	3.736e-35
9	Neutrophils	*MCU*	1.7351	0.537	0.198	3.234e-26	7.468e-22
9	Neutrophils	*LOC138849805*	1.7318	0.448	0.000	0	0
9	Neutrophils	*HERC1*	1.7295	0.716	0.283	1.006e-35	2.323e-31
9	Neutrophils	*CCRL2*	1.7290	0.328	0.003	0	0
9	Neutrophils	*FNBP1*	1.7277	0.649	0.149	9.227e-61	2.131e-56
9	Neutrophils	*SLC36A4*	1.7252	0.433	0.162	3.468e-18	8.009e-14
9	Neutrophils	*TCN1*	1.7220	0.373	0.000	0	0
9	Neutrophils	*MCL1*	1.7159	0.828	0.426	1.160e-36	2.678e-32
9	Neutrophils	*STK4*	1.7104	0.687	0.128	7.765e-85	1.793e-80
9	Neutrophils	*CD14*	1.7069	0.500	0.013	0	0
9	Neutrophils	*LST1*	1.7059	0.515	0.002	0	0
9	Neutrophils	*PLTP*	1.7017	0.216	0.072	2.943e-11	6.796e-07
9	Neutrophils	*ITGB2*	1.6999	0.604	0.011	0	0
9	Neutrophils	*GPCPD1*	1.6992	0.619	0.273	1.868e-22	4.314e-18
9	Neutrophils	*ARL5B*	1.6969	0.552	0.182	5.992e-32	1.384e-27
9	Neutrophils	*LOC100344565*	1.6949	0.493	0.001	0	0
9	Neutrophils	*LOC100352503*	1.6945	0.299	0.002	0	0
9	Neutrophils	*CLEC7A*	1.6870	0.381	0.000	0	0
9	Neutrophils	*IL1RAP*	1.6849	0.552	0.109	2.909e-62	6.718e-58
9	Neutrophils	*MARCHF3*	1.6762	0.366	0.072	5.544e-38	1.280e-33

Original cluster	Annotated cell type	Gene	avg_log2FC	pct.1	pct.2	Raw P value	Bonferroni-adjusted P value
9	Neutrophils	*ARFGEF1*	1.6720	0.701	0.333	7.330e-21	1.693e-16
9	Neutrophils	*LOC138847486*	1.6704	0.313	0.000	0	0
9	Neutrophils	*NUMB*	1.6685	0.687	0.313	5.356e-24	1.237e-19
9	Neutrophils	*BMX*	1.6671	0.328	0.002	0	0
9	Neutrophils	*RGS1*	1.6665	0.194	0.011	2.727e-72	6.298e-68
9	Neutrophils	*APBB1IP*	1.6657	0.590	0.018	0	0
9	Neutrophils	*NCF2*	1.6648	0.575	0.051	3.584e-149	8.275e-145
9	Neutrophils	*SLCO4C1*	1.6635	0.269	0.002	0	0
9	Neutrophils	*CPNE3*	1.6610	0.634	0.260	4.247e-28	9.806e-24
9	Neutrophils	*LOC100354804*	1.6463	0.149	0.024	5.325e-20	1.230e-15
9	Neutrophils	*ATP6V0C*	1.6428	0.843	0.495	5.348e-40	1.235e-35
9	Neutrophils	*DACH1*	1.6387	0.463	0.268	6.169e-10	1.424e-05
9	Neutrophils	*GLIPR1*	1.6379	0.619	0.020	0	0
9	Neutrophils	*DSTN*	1.6376	0.828	0.564	5.073e-17	1.171e-12
9	Neutrophils	*FBXL17*	1.6328	0.649	0.326	1.930e-20	4.458e-16
9	Neutrophils	*PGK1*	1.6304	0.724	0.243	1.158e-47	2.674e-43
9	Neutrophils	*MGST1*	1.6304	0.507	0.023	2.164e-235	4.997e-231
9	Neutrophils	*NFKBID*	1.6195	0.373	0.023	6.390e-133	1.476e-128
9	Neutrophils	*RAPGEF6*	1.6147	0.769	0.272	8.715e-45	2.012e-40
9	Neutrophils	*HCLS1*	1.6142	0.612	0.028	1.374e-281	3.172e-277
9	Neutrophils	*SGMS1*	1.6094	0.672	0.308	2.424e-25	5.598e-21
9	Neutrophils	*PTGS2*	1.6090	0.306	0.153	2.299e-08	5.308e-04
9	Neutrophils	*MSN*	1.6059	0.769	0.201	8.292e-69	1.915e-64
9	Neutrophils	*ZFAND3*	1.6047	0.724	0.294	1.458e-34	3.368e-30
9	Neutrophils	*MBNL1*	1.6046	0.881	0.663	1.230e-25	2.840e-21
9	Neutrophils	*RGS19*	1.5984	0.612	0.030	5.281e-268	1.219e-263
9	Neutrophils	*SERPINB1*	1.5981	0.619	0.204	9.457e-39	2.184e-34
9	Neutrophils	*LOC100359306*	1.5968	0.448	0.003	0	0
9	Neutrophils	*STXBP5*	1.5956	0.507	0.278	1.637e-10	3.781e-06
9	Neutrophils	*DGAT2*	1.5927	0.291	0.018	1.191e-101	2.751e-97
9	Neutrophils	*LOC100125981*	1.5915	0.672	0.238	9.440e-39	2.180e-34
9	Neutrophils	*NCKAP1L*	1.5877	0.530	0.012	0	0
9	Neutrophils	*LOC100351485*	1.5866	0.582	0.064	5.029e-126	1.161e-121
9	Neutrophils	*CHI3L1*	1.5847	0.396	0.008	0	0
9	Neutrophils	*CLINT1*	1.5812	0.627	0.256	1.036e-24	2.392e-20
9	Neutrophils	*DYSF*	1.5810	0.187	0.022	1.899e-35	4.385e-31
9	Neutrophils	*MAPK14*	1.5768	0.672	0.196	2.718e-48	6.277e-44
9	Neutrophils	*CAT*	1.5702	0.649	0.143	1.697e-64	3.919e-60
9	Neutrophils	*ACTR2*	1.5691	0.769	0.293	1.312e-48	3.029e-44
9	Neutrophils	*XYLT1*	1.5638	0.552	0.128	1.014e-48	2.341e-44

Original cluster	Annotated cell type	Gene	avg_log2FC	pct.1	pct.2	Raw P value	Bonferroni-adjusted P value
9	Neutrophils	*CD80*	1.5600	0.321	0.008	2.147e-239	4.958e-235
9	Neutrophils	*TCP11L2*	1.5585	0.500	0.173	1.026e-25	2.369e-21
9	Neutrophils	*ZSWIM6*	1.5572	0.761	0.370	1.896e-29	4.378e-25
9	Neutrophils	*EFHD2*	1.5541	0.664	0.107	5.828e-94	1.346e-89
9	Neutrophils	*FLI1*	1.5528	0.582	0.046	9.728e-164	2.246e-159
9	Neutrophils	*ACOD1*	1.5501	0.157	0.000	2.175e-262	5.022e-258
9	Neutrophils	*H1-2*	1.5432	0.254	0.027	3.569e-53	8.241e-49
9	Neutrophils	*WWP1*	1.5425	0.724	0.316	1.728e-32	3.991e-28
9	Neutrophils	*CTSE*	1.5416	0.425	0.000	0	0
9	Neutrophils	*CREB5*	1.5397	0.522	0.172	1.423e-28	3.287e-24
9	Neutrophils	*ELL2*	1.5392	0.590	0.149	5.347e-47	1.235e-42
9	Neutrophils	*PTEN*	1.5391	0.709	0.449	6.035e-16	1.394e-11
9	Neutrophils	*LCP2*	1.5386	0.470	0.011	0	0
9	Neutrophils	*PARP8*	1.5335	0.433	0.069	2.100e-59	4.849e-55
9	Neutrophils	*LITAF*	1.5331	0.597	0.075	1.748e-108	4.036e-104
9	Neutrophils	*PDE7A*	1.5240	0.507	0.082	1.182e-66	2.731e-62
9	Neutrophils	*LOC100350325*	1.5228	0.396	0.096	2.570e-32	5.934e-28
9	Neutrophils	*LOC103346238*	1.5218	0.373	0.001	0	0
9	Neutrophils	*DNAJC13*	1.5215	0.590	0.218	3.484e-24	8.045e-20
9	Neutrophils	*RASGEF1B*	1.5205	0.403	0.133	3.319e-21	7.665e-17
9	Neutrophils	*RAB11FIP1*	1.5193	0.418	0.145	9.742e-21	2.250e-16
9	Neutrophils	*MAN2B1*	1.5193	0.478	0.022	4.208e-218	9.717e-214
9	Neutrophils	*LRRC8D*	1.5181	0.507	0.063	2.109e-93	4.871e-89
9	Neutrophils	*MCP2*	1.5143	0.201	0.002	1.662e-238	3.838e-234
9	Neutrophils	*SLC9A9*	1.5129	0.246	0.057	4.510e-21	1.042e-16
9	Neutrophils	*CYTH1*	1.5086	0.642	0.164	1.554e-52	3.587e-48
9	Neutrophils	*TKT*	1.5072	0.530	0.081	1.118e-80	2.582e-76
9	Neutrophils	*LOC100340954*	1.5046	0.679	0.451	2.416e-13	5.580e-09
9	Neutrophils	*PSMB8*	1.5018	0.575	0.033	8.281e-220	1.912e-215
9	Neutrophils	*CSF3R*	1.4998	0.299	0.000	0	0
9	Neutrophils	*JAML*	1.4972	0.515	0.008	0	0
9	Neutrophils	*GIMAP4*	1.4971	0.418	0.010	0	0
9	Neutrophils	*FXYD5*	1.4893	0.522	0.041	1.658e-150	3.828e-146
9	Neutrophils	*WDFY2*	1.4849	0.604	0.174	4.666e-40	1.077e-35
9	Neutrophils	*E2F3*	1.4847	0.396	0.011	1.947e-260	4.495e-256
9	Neutrophils	*FAR2*	1.4835	0.201	0.024	1.591e-37	3.674e-33
9	Neutrophils	*IL1R2*	1.4790	0.299	0.000	0	0
9	Neutrophils	*PLIN2*	1.4758	0.679	0.310	1.005e-26	2.321e-22
9	Neutrophils	*PTPRJ*	1.4737	0.530	0.044	6.640e-143	1.533e-138
9	Neutrophils	*TNNI2*	1.4711	0.388	0.017	1.722e-185	3.976e-181

Original cluster	Annotated cell type	Gene	avg_log2FC	pct.1	pct.2	Raw P value	Bonferroni-adjusted P value
9	Neutrophils	*TMEM170B*	1.4656	0.545	0.069	5.116e-99	1.181e-94
9	Neutrophils	*NR4A2*	1.4650	0.366	0.145	2.468e-14	5.699e-10
9	Neutrophils	*MYO1F*	1.4648	0.537	0.011	0	0
9	Neutrophils	*PAK2*	1.4632	0.843	0.370	2.442e-43	5.640e-39
9	Neutrophils	*IL1RN*	1.4594	0.284	0.000	0	0
9	Neutrophils	*RETN*	1.4528	0.224	0.009	3.808e-118	8.794e-114
9	Neutrophils	*RAP1B*	1.4527	0.657	0.186	1.838e-48	4.245e-44
9	Neutrophils	*FCHO2*	1.4499	0.701	0.240	1.231e-43	2.842e-39
9	Neutrophils	*DEFB1*	1.4467	0.321	0.017	1.113e-130	2.571e-126
9	Neutrophils	*CRABP1*	1.4445	0.313	0.016	5.519e-131	1.275e-126
9	Neutrophils	*PIK3CG*	1.4424	0.403	0.008	0	0
9	Neutrophils	*ARPC5*	1.4421	0.784	0.296	3.253e-48	7.512e-44
9	Neutrophils	*LOC127482730*	1.4348	0.664	0.329	5.855e-19	1.352e-14
9	Neutrophils	*TNFAIP8*	1.4333	0.724	0.204	1.799e-52	4.153e-48
9	Neutrophils	*RPGRIP1*	1.4316	0.284	0.008	2.557e-188	5.904e-184
9	Neutrophils	*JAK2*	1.4297	0.567	0.174	2.556e-34	5.902e-30
9	Neutrophils	*PTPRE*	1.4270	0.515	0.039	2.142e-151	4.945e-147
9	Neutrophils	*CXCR2*	1.4238	0.336	0.000	0	0
9	Neutrophils	*STEAP4*	1.4224	0.388	0.007	0	0
9	Neutrophils	*TMCC1*	1.4210	0.440	0.131	1.689e-26	3.901e-22
9	Neutrophils	*VCL*	1.4199	0.642	0.278	3.543e-24	8.182e-20
9	Neutrophils	*BST1*	1.4111	0.343	0.043	1.232e-61	2.844e-57
9	Neutrophils	*PLXNC1*	1.4071	0.418	0.016	4.109e-219	9.488e-215
9	Neutrophils	*CEBPE*	1.4042	0.239	0.000	0	0
9	Neutrophils	*CMIP*	1.4040	0.634	0.097	1.531e-92	3.536e-88
9	Neutrophils	*EVI2B*	1.4035	0.493	0.007	0	0
9	Neutrophils	*C16H19orf38*	1.4020	0.463	0.024	6.781e-190	1.566e-185
9	Neutrophils	*NFKB1*	1.3995	0.455	0.212	8.240e-13	1.903e-08
9	Neutrophils	*FERMT3*	1.3898	0.552	0.009	0	0
9	Neutrophils	*TIAM1*	1.3883	0.485	0.151	2.916e-29	6.734e-25
9	Neutrophils	*CYBB*	1.3869	0.321	0.002	0	0
9	Neutrophils	*PDE4B*	1.3798	0.358	0.033	4.430e-88	1.023e-83
9	Neutrophils	*SAMD9L*	1.3792	0.410	0.095	9.216e-36	2.128e-31
9	Neutrophils	*LOC103351679*	1.3746	0.560	0.083	1.612e-77	3.723e-73
9	Neutrophils	*KLF6*	1.3729	0.739	0.500	3.707e-18	8.560e-14
9	Neutrophils	*IL13RA1*	1.3710	0.455	0.069	1.037e-66	2.395e-62
9	Neutrophils	*ANKS1A*	1.3708	0.552	0.162	9.960e-35	2.300e-30
9	Neutrophils	*LPCAT2*	1.3679	0.507	0.094	5.133e-57	1.185e-52
9	Neutrophils	*KCTD12*	1.3672	0.731	0.285	4.479e-38	1.034e-33
9	Neutrophils	*VPS37B*	1.3660	0.381	0.065	1.882e-48	4.345e-44

Original cluster	Annotated cell type	Gene	avg_log2FC	pct.1	pct.2	Raw P value	Bonferroni-adjusted P value
9	Neutrophils	*ANTXR2*	1.3619	0.694	0.469	4.955e-10	1.144e-05
9	Neutrophils	*LIMD2*	1.3603	0.530	0.054	1.800e-118	4.157e-114
9	Neutrophils	*LOC100357777*	1.3565	0.284	0.000	0	0
9	Neutrophils	*FMNL2*	1.3541	0.478	0.197	1.498e-16	3.458e-12
9	Neutrophils	*CKAP4*	1.3533	0.463	0.201	4.804e-17	1.109e-12
9	Neutrophils	*NT5C2*	1.3503	0.470	0.137	1.419e-28	3.278e-24
9	Neutrophils	*IRAK2*	1.3464	0.522	0.110	2.364e-51	5.459e-47
9	Neutrophils	*RHOA*	1.3445	0.888	0.610	3.749e-30	8.658e-26
9	Neutrophils	*USP38*	1.3406	0.418	0.091	1.422e-38	3.284e-34
9	Neutrophils	*CTSH*	1.3396	0.381	0.088	4.540e-33	1.048e-28
9	Neutrophils	*TEC*	1.3361	0.433	0.149	4.276e-21	9.874e-17
9	Neutrophils	*GALNT10*	1.3332	0.403	0.045	9.831e-81	2.270e-76
9	Neutrophils	*SYK*	1.3330	0.425	0.001	0	0
9	Neutrophils	*LTB*	1.3328	0.328	0.016	1.832e-140	4.231e-136
9	Neutrophils	*ADD3*	1.3308	0.634	0.455	6.753e-10	1.559e-05
9	Neutrophils	*GPR65*	1.3306	0.463	0.008	0	0
9	Neutrophils	*CFL1*	1.3305	0.828	0.511	6.785e-36	1.567e-31
9	Neutrophils	*EIF4G3*	1.3287	0.709	0.298	1.811e-29	4.181e-25
9	Neutrophils	*CD55*	1.3268	0.642	0.221	3.789e-35	8.750e-31
9	Neutrophils	*CLTC*	1.3208	0.724	0.256	4.724e-41	1.091e-36
9	Neutrophils	*LOC100350907*	1.3169	0.321	0.000	0	0
9	Neutrophils	*FBXW7*	1.3131	0.575	0.224	1.298e-21	2.998e-17
9	Neutrophils	*TPD52*	1.3104	0.545	0.048	8.756e-141	2.022e-136
9	Neutrophils	*RGS10*	1.3099	0.381	0.073	4.391e-42	1.014e-37
9	Neutrophils	*NABP1*	1.3095	0.575	0.209	4.843e-29	1.118e-24
9	Neutrophils	*CAPZA1*	1.3079	0.746	0.288	8.964e-40	2.070e-35
9	Neutrophils	*CSF1R*	1.3062	0.306	0.004	0	0
9	Neutrophils	*DENND5A*	1.3045	0.634	0.253	2.406e-27	5.555e-23
9	Neutrophils	*ST6GAL1*	1.3043	0.470	0.064	3.150e-74	7.274e-70
9	Neutrophils	*CSF2RB*	1.3016	0.403	0.001	0	0
9	Neutrophils	*CXCR1*	1.3008	0.343	0.000	0	0
9	Neutrophils	*LOC100352927*	1.2997	0.179	0.000	6.051e-306	1.397e-301
9	Neutrophils	*MSR1*	1.2948	0.149	0.001	1.367e-190	3.158e-186
9	Neutrophils	*MED13L*	1.2924	0.873	0.656	1.414e-16	3.266e-12
9	Neutrophils	*RAP1GAP2*	1.2911	0.313	0.005	3.152e-283	7.278e-279
9	Neutrophils	*UBALD2*	1.2903	0.649	0.152	6.953e-58	1.606e-53
9	Neutrophils	*RLA-A3*	1.2902	0.754	0.451	1.915e-23	4.421e-19
9	Neutrophils	*HCK*	1.2901	0.425	0.000	0	0
9	Neutrophils	*CSTA*	1.2895	0.231	0.013	1.414e-91	3.266e-87
9	Neutrophils	*RAB1A*	1.2887	0.776	0.431	3.134e-28	7.238e-24

Original cluster	Annotated cell type	Gene	avg_log2FC	pct.1	pct.2	Raw P value	Bonferroni-adjusted P value
9	Neutrophils	*MCTP2*	1.2884	0.291	0.048	1.399e-38	3.230e-34
9	Neutrophils	*IQGAP1*	1.2880	0.672	0.212	1.364e-42	3.150e-38
9	Neutrophils	*IL6R*	1.2879	0.425	0.043	2.535e-93	5.854e-89
9	Neutrophils	*STK38L*	1.2857	0.358	0.076	6.536e-34	1.509e-29
9	Neutrophils	*TAOK3*	1.2806	0.679	0.203	2.493e-45	5.757e-41
9	Neutrophils	*WAS*	1.2779	0.493	0.021	6.916e-236	1.597e-231
9	Neutrophils	*FGD3*	1.2772	0.403	0.011	3.433e-267	7.928e-263
9	Neutrophils	*RAB7A*	1.2767	0.716	0.327	1.213e-25	2.802e-21
9	Neutrophils	*BATF*	1.2747	0.216	0.006	4.430e-149	1.023e-144
9	Neutrophils	*C5AR1*	1.2684	0.470	0.000	0	0
9	Neutrophils	*TREM2*	1.2679	0.313	0.000	0	0
9	Neutrophils	*FBXW2*	1.2624	0.485	0.186	1.919e-21	4.431e-17
9	Neutrophils	*CLEC5A*	1.2578	0.440	0.000	0	0
9	Neutrophils	*EGR3*	1.2556	0.276	0.031	1.907e-55	4.404e-51
9	Neutrophils	*ARL4C*	1.2521	0.493	0.028	2.186e-186	5.047e-182
9	Neutrophils	*KMT2C*	1.2503	0.776	0.473	3.202e-15	7.395e-11
9	Neutrophils	*SGPP2*	1.2502	0.224	0.006	2.316e-145	5.349e-141
9	Neutrophils	*IL17RA*	1.2498	0.396	0.015	4.317e-212	9.969e-208
9	Neutrophils	*BIRC3*	1.2481	0.224	0.023	1.792e-47	4.139e-43
9	Neutrophils	*MYH9*	1.2468	0.582	0.214	8.839e-26	2.041e-21
9	Neutrophils	*OSBPL9*	1.2434	0.806	0.405	6.553e-23	1.513e-18
9	Neutrophils	*ELF1*	1.2427	0.664	0.231	3.940e-35	9.097e-31
9	Neutrophils	*LOC138849806*	1.2423	0.336	0.000	0	0
9	Neutrophils	*KDM4C*	1.2401	0.485	0.133	2.315e-32	5.345e-28
9	Neutrophils	*CHD7*	1.2393	0.321	0.034	4.341e-68	1.003e-63
9	Neutrophils	*DNAI7*	1.2378	0.328	0.088	9.792e-23	2.261e-18
9	Neutrophils	*MRC1*	1.2365	0.261	0.005	2.276e-231	5.256e-227
9	Neutrophils	*RIOK3*	1.2328	0.634	0.208	2.526e-34	5.833e-30
9	Neutrophils	*PSTPIP2*	1.2323	0.351	0.015	3.663e-171	8.459e-167
9	Neutrophils	*AKAP13*	1.2298	0.642	0.197	8.469e-40	1.956e-35
9	Neutrophils	*ETF1*	1.2289	0.575	0.162	7.692e-40	1.776e-35
9	Neutrophils	*VAMP8*	1.2212	0.627	0.154	1.083e-53	2.500e-49
9	Neutrophils	*PLBD1*	1.2191	0.276	0.031	1.973e-54	4.555e-50
9	Neutrophils	*RBM47*	1.2184	0.403	0.021	1.002e-162	2.313e-158
9	Neutrophils	*LOC100346112*	1.2164	0.851	0.585	5.525e-24	1.276e-19
9	Neutrophils	*WDR26*	1.2126	0.560	0.214	1.324e-23	3.058e-19
9	Neutrophils	*EXOC4*	1.2104	0.813	0.425	7.487e-21	1.729e-16
9	Neutrophils	*PSTPIP1*	1.2099	0.381	0.008	6.469e-304	1.494e-299
9	Neutrophils	*NHSL2*	1.2095	0.366	0.151	1.538e-12	3.552e-08
9	Neutrophils	*LRG1*	1.2090	0.448	0.084	7.282e-50	1.682e-45

Original cluster	Annotated cell type	Gene	avg_log2FC	pct.1	pct.2	Raw P value	Bonferroni-adjusted P value
9	Neutrophils	*LOC100339318*	1.2086	0.440	0.073	1.013e-57	2.340e-53
9	Neutrophils	*PLEKHJ1*	1.2083	0.627	0.175	2.281e-43	5.267e-39
9	Neutrophils	*TPM3*	1.2079	0.649	0.184	4.683e-49	1.081e-44
9	Neutrophils	*PTBP3*	1.2077	0.642	0.211	8.610e-36	1.988e-31
9	Neutrophils	*ZFC3H1*	1.2077	0.545	0.228	2.831e-20	6.538e-16
9	Neutrophils	*HIPK1*	1.2072	0.642	0.206	2.343e-34	5.411e-30
9	Neutrophils	*GPR155*	1.2068	0.403	0.023	1.795e-149	4.144e-145
9	Neutrophils	*RAP1A*	1.2050	0.724	0.344	7.266e-20	1.678e-15
9	Neutrophils	*SELENOP*	1.1988	0.313	0.812	1.388e-14	3.205e-10
9	Neutrophils	*BTAF1*	1.1962	0.396	0.176	6.852e-11	1.582e-06
9	Neutrophils	*IRF5*	1.1930	0.425	0.001	0	0
9	Neutrophils	*EIF4E3*	1.1880	0.545	0.279	2.811e-12	6.491e-08
9	Neutrophils	*UBP1*	1.1856	0.440	0.103	6.064e-38	1.400e-33
9	Neutrophils	*FPR2*	1.1829	0.321	0.000	0	0
9	Neutrophils	*PHACTR2*	1.1828	0.493	0.268	7.133e-09	1.647e-04
9	Neutrophils	*NOTCH2*	1.1782	0.612	0.328	4.715e-14	1.089e-09
9	Neutrophils	*CDC42SE2*	1.1772	0.440	0.087	3.035e-44	7.008e-40
9	Neutrophils	*STK17A*	1.1726	0.604	0.106	5.191e-74	1.199e-69
9	Neutrophils	*WDFY4*	1.1723	0.396	0.003	0	0
9	Neutrophils	*PELI1*	1.1716	0.522	0.248	6.146e-14	1.419e-09
9	Neutrophils	*MTMR3*	1.1703	0.590	0.196	2.493e-30	5.757e-26
9	Neutrophils	*TENT5C*	1.1666	0.425	0.212	2.956e-09	6.826e-05
9	Neutrophils	*PIAS1*	1.1658	0.769	0.378	6.071e-22	1.402e-17
9	Neutrophils	*BMP2K*	1.1645	0.537	0.187	1.019e-23	2.354e-19
9	Neutrophils	*CCND3*	1.1641	0.530	0.235	4.414e-17	1.019e-12
9	Neutrophils	*FOXO3*	1.1633	0.634	0.327	2.360e-17	5.449e-13
9	Neutrophils	*MFSD14B*	1.1630	0.425	0.127	1.022e-25	2.361e-21
9	Neutrophils	*EXOC6*	1.1627	0.403	0.102	3.036e-29	7.010e-25
9	Neutrophils	*SIK3*	1.1621	0.649	0.294	1.020e-21	2.354e-17
9	Neutrophils	*DISC1*	1.1585	0.276	0.029	7.369e-59	1.702e-54
9	Neutrophils	*LOC127488978*	1.1580	0.410	0.000	0	0
9	Neutrophils	*ADAM17*	1.1573	0.425	0.054	5.871e-74	1.356e-69
9	Neutrophils	*MAN1A1*	1.1572	0.403	0.158	3.396e-16	7.842e-12
9	Neutrophils	*VSTM1*	1.1569	0.284	0.000	0	0
9	Neutrophils	*MTHFS*	1.1555	0.343	0.035	3.498e-75	8.078e-71
9	Neutrophils	*CMTM6*	1.1519	0.567	0.156	1.518e-38	3.505e-34
9	Neutrophils	*CBLB*	1.1500	0.627	0.405	7.407e-08	1.710e-03
9	Neutrophils	*RAB32*	1.1500	0.493	0.077	1.198e-68	2.766e-64
9	Neutrophils	*ATP2A2*	1.1489	0.552	0.183	1.290e-26	2.980e-22
9	Neutrophils	*WDFY3*	1.1381	0.724	0.438	2.066e-14	4.771e-10

Original cluster	Annotated cell type	Gene	avg_log2FC	pct.1	pct.2	Raw P value	Bonferroni-adjusted P value
9	Neutrophils	*TFEC*	1.1376	0.246	0.001	0	0
9	Neutrophils	*GNAI3*	1.1354	0.642	0.320	1.068e-17	2.465e-13
9	Neutrophils	*AFTPH*	1.1344	0.493	0.144	3.751e-31	8.662e-27
9	Neutrophils	*MILR1*	1.1343	0.284	0.000	0	0
9	Neutrophils	*PIK3CB*	1.1340	0.478	0.085	8.793e-56	2.030e-51
9	Neutrophils	*PSMB9*	1.1324	0.463	0.029	1.132e-160	2.615e-156
9	Neutrophils	*ATP13A3*	1.1309	0.410	0.098	1.486e-32	3.431e-28
9	Neutrophils	*STX11*	1.1248	0.254	0.005	5.840e-215	1.349e-210
9	Neutrophils	*SLC25A51*	1.1236	0.440	0.076	5.434e-54	1.255e-49
9	Neutrophils	*LDHA*	1.1235	0.440	0.049	9.501e-90	2.194e-85
9	Neutrophils	*MAP3K2*	1.1209	0.687	0.360	3.360e-19	7.760e-15
9	Neutrophils	*ZSWIM5*	1.1196	0.284	0.057	1.095e-27	2.529e-23
9	Neutrophils	*LOC100338575*	1.1177	0.119	0.015	1.706e-21	3.940e-17
9	Neutrophils	*RLA-DMB*	1.1170	0.358	0.003	0	0
9	Neutrophils	*MKLN1*	1.1170	0.545	0.221	9.214e-19	2.128e-14
9	Neutrophils	*STAP1*	1.1162	0.134	0.006	1.488e-60	3.435e-56
9	Neutrophils	*LMO2*	1.1148	0.500	0.049	2.296e-110	5.302e-106
9	Neutrophils	*KDM7A*	1.1140	0.575	0.138	3.067e-48	7.083e-44
9	Neutrophils	*HMGCR*	1.1127	0.493	0.151	4.691e-25	1.083e-20
9	Neutrophils	*TALDO1*	1.1109	0.746	0.606	3.967e-10	9.160e-06
9	Neutrophils	*CDKN2D*	1.1098	0.440	0.125	1.867e-28	4.311e-24
9	Neutrophils	*DMXL2*	1.1082	0.366	0.054	7.361e-54	1.700e-49
9	Neutrophils	*RAF1*	1.1081	0.522	0.193	3.705e-22	8.556e-18
9	Neutrophils	*CDKN1A*	1.1075	0.343	0.114	1.826e-17	4.218e-13
9	Neutrophils	*NCK1*	1.1073	0.634	0.346	5.007e-14	1.156e-09
9	Neutrophils	*OLR1*	1.1066	0.172	0.001	7.075e-245	1.634e-240
9	Neutrophils	*SIPA1L1*	1.1005	0.455	0.252	4.860e-09	1.122e-04
9	Neutrophils	*TESK2*	1.0990	0.201	0.060	1.765e-12	4.077e-08
9	Neutrophils	*F5*	1.0989	0.328	0.001	0	0
9	Neutrophils	*H1-4*	1.0983	0.440	0.078	8.548e-52	1.974e-47
9	Neutrophils	*IL1A*	1.0954	0.119	0.000	9.899e-201	2.286e-196
9	Neutrophils	*PSME4*	1.0938	0.500	0.187	4.109e-21	9.487e-17
9	Neutrophils	*LOC100348267*	1.0919	0.500	0.134	3.266e-35	7.542e-31
9	Neutrophils	*PIK3R5*	1.0911	0.418	0.003	0	0
9	Neutrophils	*ARHGEF6*	1.0907	0.410	0.064	1.688e-56	3.897e-52
9	Neutrophils	*TANK*	1.0896	0.560	0.168	1.165e-31	2.689e-27
9	Neutrophils	*MKNK1*	1.0890	0.448	0.084	1.484e-48	3.426e-44
9	Neutrophils	*IFRD1*	1.0883	0.448	0.215	9.109e-12	2.103e-07
9	Neutrophils	*EBI3*	1.0872	0.381	0.001	0	0
9	Neutrophils	*PPP1R3B*	1.0870	0.261	0.047	5.286e-31	1.221e-26

Original cluster	Annotated cell type	Gene	avg_log2FC	pct.1	pct.2	Raw P value	Bonferroni-adjusted P value
9	Neutrophils	*PAK1*	1.0864	0.231	0.025	2.575e-47	5.945e-43
9	Neutrophils	*ZDHHC20*	1.0859	0.537	0.166	3.442e-31	7.948e-27
9	Neutrophils	*EGR2*	1.0858	0.231	0.024	3.143e-50	7.257e-46
9	Neutrophils	*MMP9*	1.0853	0.201	0.000	0	0
9	Neutrophils	*VSIG4*	1.0824	0.187	0.001	7.307e-257	1.687e-252
9	Neutrophils	*CDC14A*	1.0804	0.455	0.073	1.708e-59	3.944e-55
9	Neutrophils	*KLF7*	1.0804	0.410	0.231	5.720e-09	1.321e-04
9	Neutrophils	*MEMO1*	1.0789	0.485	0.106	2.980e-42	6.881e-38
9	Neutrophils	*PADI4*	1.0787	0.239	0.000	0	0
9	Neutrophils	*SLMAP*	1.0783	0.530	0.233	4.149e-16	9.580e-12
9	Neutrophils	*TNFSF10*	1.0773	0.440	0.063	5.694e-65	1.315e-60
9	Neutrophils	*RNF130*	1.0767	0.761	0.420	7.340e-22	1.695e-17
9	Neutrophils	*LRRK2*	1.0763	0.351	0.135	5.152e-15	1.190e-10
9	Neutrophils	*NDEL1*	1.0758	0.478	0.150	4.890e-26	1.129e-21
9	Neutrophils	*ASXL2*	1.0743	0.537	0.199	4.156e-21	9.597e-17
9	Neutrophils	*DUSP6*	1.0703	0.328	0.024	2.053e-100	4.742e-96
9	Neutrophils	*CD244*	1.0702	0.134	0.003	6.009e-100	1.388e-95
9	Neutrophils	*OSTF1*	1.0669	0.687	0.328	7.008e-21	1.618e-16
9	Neutrophils	*CYFIP2*	1.0649	0.306	0.073	6.603e-24	1.525e-19
9	Neutrophils	*ACAP2*	1.0626	0.604	0.335	1.291e-12	2.980e-08
9	Neutrophils	*LY96*	1.0626	0.403	0.040	5.130e-89	1.185e-84
9	Neutrophils	*LOC103350930*	1.0623	0.276	0.017	3.510e-97	8.106e-93
9	Neutrophils	*PAFAH1B1*	1.0620	0.739	0.409	3.237e-18	7.475e-14
9	Neutrophils	*ARPC3*	1.0596	0.694	0.346	3.772e-26	8.710e-22
9	Neutrophils	*SPAG9*	1.0585	0.604	0.298	4.173e-17	9.635e-13
9	Neutrophils	*WIPF1*	1.0553	0.567	0.187	6.945e-30	1.604e-25
9	Neutrophils	*OSBPL6*	1.0545	0.313	0.093	1.037e-18	2.394e-14
9	Neutrophils	*LOC100352110*	1.0534	0.328	0.005	0	0
9	Neutrophils	*BCAS3*	1.0528	0.612	0.387	4.938e-09	1.140e-04
9	Neutrophils	*SLAMF6*	1.0527	0.224	0.003	1.830e-219	4.226e-215
9	Neutrophils	*KDM6B*	1.0524	0.560	0.279	4.327e-16	9.993e-12
9	Neutrophils	*GNA13*	1.0522	0.522	0.161	2.488e-30	5.745e-26
9	Neutrophils	*TNRC6B*	1.0483	0.761	0.495	1.641e-10	3.790e-06
9	Neutrophils	*WWP2*	1.0479	0.343	0.066	3.017e-35	6.967e-31
9	Neutrophils	*RAB2A*	1.0470	0.821	0.485	1.340e-20	3.095e-16
9	Neutrophils	*TNFRSF1B*	1.0448	0.433	0.029	5.556e-144	1.283e-139
9	Neutrophils	*TLE4*	1.0435	0.657	0.373	2.116e-14	4.886e-10
9	Neutrophils	*LAIR1*	1.0428	0.410	0.003	0	0
9	Neutrophils	*RNASE6*	1.0388	0.351	0.000	0	0
9	Neutrophils	*LOC100347254*	1.0371	0.366	0.088	2.606e-28	6.018e-24

Original cluster	Annotated cell type	Gene	avg_log2FC	pct.1	pct.2	Raw P value	Bonferroni-adjusted P value
9	Neutrophils	*ABI1*	1.0367	0.634	0.278	1.206e-19	2.784e-15
9	Neutrophils	*HYCC1*	1.0361	0.485	0.142	3.030e-29	6.998e-25
9	Neutrophils	*UBE2D3*	1.0336	0.776	0.494	4.476e-15	1.034e-10
9	Neutrophils	*SUCO*	1.0331	0.522	0.295	3.965e-09	9.155e-05
9	Neutrophils	*SAMHD1*	1.0324	0.284	0.044	1.427e-38	3.295e-34
9	Neutrophils	*MIER1*	1.0310	0.769	0.457	1.197e-16	2.764e-12
9	Neutrophils	*ADAM9*	1.0308	0.575	0.257	5.987e-19	1.382e-14
9	Neutrophils	*LOC103345057*	1.0266	0.269	0.003	0	0
9	Neutrophils	*BCL2L15*	1.0257	0.299	0.000	0	0
9	Neutrophils	*FBXO38*	1.0255	0.388	0.126	2.030e-20	4.688e-16
9	Neutrophils	*RPL36AL*	1.0252	0.239	0.054	8.761e-22	2.023e-17
9	Neutrophils	*GRN*	1.0234	0.530	0.168	2.978e-30	6.878e-26
9	Neutrophils	*RUBCNL*	1.0212	0.194	0.010	3.159e-78	7.296e-74
9	Neutrophils	*MARK2*	1.0192	0.410	0.081	1.457e-41	3.364e-37
9	Neutrophils	*SP100*	1.0190	0.366	0.023	4.752e-129	1.097e-124
9	Neutrophils	*LOC100354169*	1.0180	0.418	0.158	2.321e-16	5.361e-12
9	Neutrophils	*TULP4*	1.0148	0.403	0.128	2.051e-20	4.735e-16
9	Neutrophils	*CDC42SE1*	1.0143	0.604	0.155	8.660e-45	2.000e-40
9	Neutrophils	*EPB41L3*	1.0141	0.410	0.180	2.922e-12	6.747e-08
9	Neutrophils	*CPD*	1.0139	0.478	0.283	4.016e-08	9.274e-04
9	Neutrophils	*JAK1*	1.0135	0.836	0.502	6.369e-15	1.471e-10
9	Neutrophils	*EHD4*	1.0110	0.433	0.062	1.006e-63	2.322e-59
9	Neutrophils	*ATP6V0A1*	1.0102	0.470	0.103	7.142e-43	1.649e-38
9	Neutrophils	*MSL1*	1.0094	0.530	0.167	9.812e-28	2.266e-23
9	Neutrophils	*ITGAL*	1.0091	0.306	0.006	1.380e-250	3.188e-246
9	Neutrophils	*LOC100358478*	1.0078	0.216	0.001	0	0
9	Neutrophils	*SENP7*	1.0059	0.590	0.277	3.682e-16	8.503e-12
9	Neutrophils	*CREM*	1.0055	0.448	0.150	5.571e-22	1.286e-17
9	Neutrophils	*MOB1A*	1.0039	0.507	0.112	1.092e-46	2.521e-42
9	Neutrophils	*TMEM131L*	1.0037	0.433	0.117	1.405e-29	3.244e-25
9	Neutrophils	*METTL9*	1.0008	0.433	0.158	1.155e-17	2.667e-13
9	Neutrophils	*ARHGAP30*	0.9982	0.425	0.007	0	0
9	Neutrophils	*GAB2*	0.9968	0.530	0.298	2.354e-10	5.436e-06
9	Neutrophils	*GLIPR2*	0.9942	0.403	0.042	7.946e-84	1.835e-79
9	Neutrophils	*CLEC12A*	0.9931	0.306	0.000	0	0
9	Neutrophils	*BIN2*	0.9930	0.410	0.008	0	0
9	Neutrophils	*PPP4R1*	0.9929	0.463	0.204	8.952e-14	2.067e-09
9	Neutrophils	*BACH1*	0.9914	0.604	0.203	1.238e-29	2.859e-25
9	Neutrophils	*LPCAT3*	0.9900	0.366	0.043	5.235e-67	1.209e-62
9	Neutrophils	*NLRP3*	0.9891	0.381	0.000	0	0

Original cluster	Annotated cell type	Gene	avg_log2FC	pct.1	pct.2	Raw P value	Bonferroni-adjusted P value
9	Neutrophils	*WIPI1*	0.9877	0.351	0.048	1.742e-55	4.022e-51
9	Neutrophils	*NCKAP5*	0.9867	0.343	0.108	1.283e-18	2.964e-14
9	Neutrophils	*CEMIP2*	0.9855	0.351	0.141	2.094e-12	4.835e-08
9	Neutrophils	*NUP98*	0.9846	0.649	0.321	1.274e-16	2.941e-12
9	Neutrophils	*AP3B1*	0.9844	0.530	0.149	1.094e-32	2.527e-28
9	Neutrophils	*ANXA3*	0.9838	0.418	0.192	2.194e-13	5.066e-09
9	Neutrophils	*FCHSD2*	0.9818	0.545	0.240	7.945e-18	1.835e-13
9	Neutrophils	*ARPC4*	0.9802	0.552	0.161	1.030e-34	2.378e-30
9	Neutrophils	*LASP1*	0.9774	0.627	0.238	1.567e-27	3.618e-23
9	Neutrophils	*ATF6*	0.9767	0.507	0.163	1.648e-24	3.806e-20
9	Neutrophils	*LOC100346553*	0.9758	0.201	0.011	3.947e-79	9.113e-75
9	Neutrophils	*SNX13*	0.9746	0.545	0.235	7.769e-16	1.794e-11
9	Neutrophils	*CYLD*	0.9745	0.440	0.126	1.845e-27	4.262e-23
9	Neutrophils	*CSF2RA*	0.9742	0.351	0.000	0	0
9	Neutrophils	*GRB2*	0.9706	0.590	0.201	1.086e-30	2.509e-26
9	Neutrophils	*SEL1L3*	0.9702	0.313	0.010	4.994e-196	1.153e-191
9	Neutrophils	*DAPK1*	0.9701	0.433	0.050	6.000e-82	1.386e-77
9	Neutrophils	*LPIN2*	0.9687	0.485	0.151	7.285e-25	1.682e-20
9	Neutrophils	*RASSF3*	0.9669	0.463	0.103	4.136e-41	9.551e-37
9	Neutrophils	*EFCAB14*	0.9661	0.448	0.213	8.365e-11	1.932e-06
9	Neutrophils	*PTK2B*	0.9659	0.388	0.013	2.261e-232	5.220e-228
9	Neutrophils	*SNX10*	0.9652	0.388	0.024	2.172e-135	5.016e-131
9	Neutrophils	*ATAD2B*	0.9651	0.642	0.336	6.276e-13	1.449e-08
9	Neutrophils	*LIMS1*	0.9642	0.560	0.301	2.128e-11	4.913e-07
9	Neutrophils	*GRAMD1B*	0.9630	0.209	0.046	3.433e-18	7.927e-14
9	Neutrophils	*VPS26A*	0.9626	0.493	0.211	8.465e-15	1.955e-10
9	Neutrophils	*INPP5D*	0.9624	0.418	0.018	5.701e-201	1.317e-196
9	Neutrophils	*ACSL1*	0.9622	0.396	0.106	1.305e-27	3.014e-23
9	Neutrophils	*SYNGR2*	0.9598	0.485	0.101	2.411e-47	5.568e-43
9	Neutrophils	*CDK19*	0.9595	0.478	0.221	2.129e-13	4.917e-09
9	Neutrophils	*ABRACL*	0.9578	0.396	0.038	3.445e-91	7.955e-87
9	Neutrophils	*CDC42*	0.9575	0.843	0.630	6.311e-17	1.457e-12
9	Neutrophils	*LOC103346857*	0.9556	0.351	0.000	0	0
9	Neutrophils	*CPEB3*	0.9555	0.403	0.181	2.428e-11	5.607e-07
9	Neutrophils	*YWHAZ*	0.9554	0.873	0.643	1.828e-15	4.222e-11
9	Neutrophils	*CHPT1*	0.9541	0.530	0.155	1.355e-31	3.129e-27
9	Neutrophils	*PAN3*	0.9530	0.649	0.363	2.790e-13	6.443e-09
9	Neutrophils	*ENO1*	0.9528	0.731	0.558	6.563e-13	1.516e-08
9	Neutrophils	*CROT*	0.9521	0.493	0.116	1.839e-37	4.246e-33
9	Neutrophils	*GCNT1*	0.9516	0.194	0.014	2.275e-61	5.254e-57

Original cluster	Annotated cell type	Gene	avg_log2FC	pct.1	pct.2	Raw P value	Bonferroni-adjusted P value
9	Neutrophils	*SRP14*	0.9508	0.694	0.355	6.205e-21	1.433e-16
9	Neutrophils	*CREB1*	0.9482	0.642	0.354	3.353e-12	7.743e-08
9	Neutrophils	*WAPL*	0.9482	0.545	0.224	3.266e-19	7.541e-15
9	Neutrophils	*HNRNPLL*	0.9473	0.336	0.126	1.318e-12	3.042e-08
9	Neutrophils	*SLC12A6*	0.9458	0.537	0.211	1.619e-19	3.739e-15
9	Neutrophils	*MTHFD2*	0.9457	0.381	0.026	6.277e-124	1.450e-119
9	Neutrophils	*LOC100337909*	0.9450	0.321	0.001	0	0
9	Neutrophils	*USP3*	0.9436	0.403	0.174	4.850e-12	1.120e-07
9	Neutrophils	*CAB39*	0.9436	0.537	0.184	7.671e-25	1.771e-20
9	Neutrophils	*MPZL3*	0.9435	0.239	0.018	4.213e-70	9.729e-66
9	Neutrophils	*LOC138843205*	0.9434	0.134	0.001	2.037e-159	4.704e-155
9	Neutrophils	*TRAK2*	0.9429	0.306	0.088	9.196e-18	2.123e-13
9	Neutrophils	*FGR*	0.9426	0.306	0.008	6.099e-215	1.408e-210
9	Neutrophils	*FCGR1A*	0.9418	0.216	0.002	1.768e-275	4.084e-271
9	Neutrophils	*ANXA11*	0.9402	0.537	0.163	1.782e-31	4.115e-27
9	Neutrophils	*RC3H2*	0.9391	0.560	0.225	6.960e-19	1.607e-14
9	Neutrophils	*LIPA*	0.9373	0.299	0.073	8.666e-23	2.001e-18
9	Neutrophils	*IPMK*	0.9368	0.373	0.071	2.235e-39	5.162e-35
9	Neutrophils	*AGTPBP1*	0.9337	0.336	0.098	9.739e-20	2.249e-15
9	Neutrophils	*USP32*	0.9335	0.493	0.243	1.825e-11	4.213e-07
9	Neutrophils	*NHERF1*	0.9314	0.336	0.025	6.248e-99	1.443e-94
9	Neutrophils	*CD79A*	0.9276	0.134	0.006	8.572e-60	1.979e-55
9	Neutrophils	*TNFRSF21*	0.9268	0.433	0.076	1.064e-49	2.457e-45
9	Neutrophils	*NFAM1*	0.9250	0.351	0.001	0	0
9	Neutrophils	*RHOG*	0.9234	0.470	0.056	1.997e-85	4.612e-81
9	Neutrophils	*ATP6V0D1*	0.9231	0.493	0.110	2.950e-44	6.813e-40
9	Neutrophils	*LOC103350037*	0.9229	0.299	0.009	1.504e-191	3.473e-187
9	Neutrophils	*CCR1*	0.9228	0.269	0.000	0	0
9	Neutrophils	*FNIP1*	0.9218	0.627	0.344	5.855e-14	1.352e-09
9	Neutrophils	*COG5*	0.9207	0.560	0.262	7.791e-14	1.799e-09
9	Neutrophils	*TAGAP*	0.9199	0.306	0.008	3.862e-211	8.917e-207
9	Neutrophils	*ATP6V1G1*	0.9196	0.754	0.482	2.713e-20	6.266e-16
9	Neutrophils	*HOMER1*	0.9196	0.522	0.237	1.676e-14	3.871e-10
9	Neutrophils	*LACC1*	0.9189	0.403	0.181	4.990e-11	1.152e-06
9	Neutrophils	*SLC66A3*	0.9187	0.396	0.104	4.303e-28	9.935e-24
9	Neutrophils	*SUCLA2*	0.9178	0.448	0.167	3.380e-18	7.805e-14
9	Neutrophils	*IL10RB*	0.9159	0.403	0.034	1.564e-106	3.611e-102
9	Neutrophils	*ZNF407*	0.9151	0.448	0.222	1.527e-09	3.525e-05
9	Neutrophils	*LY86*	0.9151	0.291	0.000	0	0
9	Neutrophils	*SNRK*	0.9135	0.403	0.125	3.922e-21	9.057e-17

Original cluster	Annotated cell type	Gene	avg_log2FC	pct.1	pct.2	Raw P value	Bonferroni-adjusted P value
9	Neutrophils	*SRI*	0.9128	0.604	0.235	2.445e-25	5.647e-21
9	Neutrophils	*YWHAB*	0.9120	0.716	0.406	4.211e-20	9.725e-16
9	Neutrophils	*CSK*	0.9094	0.500	0.159	1.353e-27	3.124e-23
9	Neutrophils	*TNFAIP3*	0.9080	0.269	0.071	8.591e-18	1.984e-13
9	Neutrophils	*CD68*	0.9077	0.336	0.023	2.613e-106	6.034e-102
9	Neutrophils	*LOC100353676*	0.9063	0.313	0.020	5.952e-106	1.374e-101
9	Neutrophils	*PPP1R18*	0.9054	0.418	0.058	1.935e-64	4.469e-60
9	Neutrophils	*MEF2C*	0.8995	0.396	0.130	1.672e-18	3.862e-14
9	Neutrophils	*HMOX2*	0.8989	0.336	0.092	2.572e-21	5.940e-17
9	Neutrophils	*MAPK13*	0.8988	0.231	0.008	1.768e-136	4.082e-132
9	Neutrophils	*HOOK3*	0.8986	0.701	0.436	2.378e-11	5.492e-07
9	Neutrophils	*CRLF3*	0.8979	0.403	0.074	6.608e-45	1.526e-40
9	Neutrophils	*CCNL1*	0.8964	0.843	0.666	2.236e-09	5.163e-05
9	Neutrophils	*CENPF*	0.8933	0.127	0.007	3.952e-51	9.126e-47
9	Neutrophils	*TRAPPC8*	0.8916	0.448	0.197	1.327e-11	3.064e-07
9	Neutrophils	*CTSZ*	0.8891	0.440	0.112	6.385e-33	1.474e-28
9	Neutrophils	*VTI1A*	0.8889	0.493	0.272	3.181e-08	7.346e-04
9	Neutrophils	*SYNJ1*	0.8885	0.425	0.105	1.694e-31	3.912e-27
9	Neutrophils	*ATP6V1B2*	0.8854	0.440	0.104	1.133e-34	2.615e-30
9	Neutrophils	*CYRIA*	0.8844	0.366	0.059	1.571e-47	3.627e-43
9	Neutrophils	*HAPSTR1*	0.8829	0.522	0.203	1.245e-19	2.875e-15
9	Neutrophils	*PLA2G4A*	0.8821	0.254	0.100	1.997e-09	4.611e-05
9	Neutrophils	*FBXL5*	0.8821	0.440	0.180	1.774e-15	4.098e-11
9	Neutrophils	*ITSN2*	0.8800	0.560	0.236	1.133e-17	2.616e-13
9	Neutrophils	*LOC100341917*	0.8788	0.142	0.000	1.240e-283	2.864e-279
9	Neutrophils	*IL10RA*	0.8787	0.358	0.001	0	0
9	Neutrophils	*ANKRD22*	0.8765	0.216	0.002	1.444e-282	3.335e-278
9	Neutrophils	*SUSD6*	0.8763	0.351	0.087	1.431e-25	3.304e-21
9	Neutrophils	*FAM13B*	0.8755	0.493	0.281	8.832e-07	2.039e-02
9	Neutrophils	*AHR*	0.8755	0.261	0.032	1.066e-45	2.462e-41
9	Neutrophils	*PLD3*	0.8740	0.433	0.150	3.083e-20	7.120e-16
9	Neutrophils	*PTPN6*	0.8736	0.396	0.023	1.005e-145	2.321e-141
9	Neutrophils	*AP1G1*	0.8734	0.560	0.192	1.282e-23	2.960e-19
9	Neutrophils	*RNF19B*	0.8731	0.418	0.192	1.082e-11	2.498e-07
9	Neutrophils	*SLC16A3*	0.8721	0.261	0.000	0	0
9	Neutrophils	*RESF1*	0.8713	0.291	0.073	1.497e-20	3.456e-16
9	Neutrophils	*HNMT*	0.8708	0.299	0.041	1.225e-47	2.830e-43
9	Neutrophils	*PANK3*	0.8706	0.478	0.136	1.117e-28	2.579e-24
9	Neutrophils	*ARHGAP45*	0.8706	0.343	0.008	2.880e-253	6.650e-249
9	Neutrophils	*USP15*	0.8700	0.597	0.347	4.690e-10	1.083e-05

Original cluster	Annotated cell type	Gene	avg_log2FC	pct.1	pct.2	Raw P value	Bonferroni-adjusted P value
9	Neutrophils	*PTAFR*	0.8690	0.321	0.003	0	0
9	Neutrophils	*ABCA6*	0.8685	0.187	0.047	2.814e-14	6.499e-10
9	Neutrophils	*OSBPL3*	0.8679	0.172	0.037	5.511e-16	1.273e-11
9	Neutrophils	*LOC103351099*	0.8677	0.276	0.000	0	0
9	Neutrophils	*RASSF2*	0.8670	0.388	0.033	3.336e-101	7.704e-97
9	Neutrophils	*SRRM2*	0.8666	0.821	0.737	4.920e-16	1.136e-11
9	Neutrophils	*TEX2*	0.8663	0.313	0.080	2.728e-22	6.299e-18
9	Neutrophils	*PGD*	0.8662	0.343	0.067	1.155e-35	2.668e-31
9	Neutrophils	*SYAP1*	0.8655	0.410	0.143	6.301e-18	1.455e-13
9	Neutrophils	*JPT1*	0.8640	0.448	0.133	6.937e-26	1.602e-21
9	Neutrophils	*TREML4*	0.8630	0.224	0.001	0	0
9	Neutrophils	*LOC100351416*	0.8630	0.231	0.001	0	0
9	Neutrophils	*RUNX2*	0.8623	0.254	0.092	1.926e-10	4.447e-06
9	Neutrophils	*COQ10B*	0.8621	0.604	0.255	2.685e-19	6.200e-15
9	Neutrophils	*LOC127491011*	0.8613	0.418	0.057	7.713e-65	1.781e-60
9	Neutrophils	*DYRK1A*	0.8609	0.515	0.256	6.214e-12	1.435e-07
9	Neutrophils	*LACTB*	0.8591	0.470	0.127	9.516e-31	2.197e-26
9	Neutrophils	*LOC100350957*	0.8574	0.187	0.007	1.868e-103	4.314e-99
9	Neutrophils	*UGT1A7*	0.8561	0.201	0.025	3.245e-35	7.493e-31
9	Neutrophils	*SMIM14*	0.8561	0.463	0.100	5.352e-42	1.236e-37
9	Neutrophils	*LMNB1*	0.8554	0.291	0.053	1.328e-33	3.067e-29
9	Neutrophils	*STIM1*	0.8548	0.425	0.142	4.094e-20	9.453e-16
9	Neutrophils	*PDE3B*	0.8545	0.336	0.134	2.933e-11	6.774e-07
9	Neutrophils	*FUT8*	0.8534	0.336	0.164	1.672e-08	3.862e-04
9	Neutrophils	*ACSL4*	0.8524	0.239	0.081	5.957e-11	1.376e-06
9	Neutrophils	*VAV1*	0.8518	0.336	0.005	0	0
9	Neutrophils	*TMEM123*	0.8508	0.455	0.219	1.003e-11	2.316e-07
9	Neutrophils	*LOC100351300*	0.8497	0.321	0.000	0	0
9	Neutrophils	*NEK7*	0.8492	0.455	0.241	1.316e-08	3.038e-04
9	Neutrophils	*FMNL1*	0.8491	0.381	0.007	0	0
9	Neutrophils	*ACTN1*	0.8488	0.560	0.231	1.043e-18	2.408e-14
9	Neutrophils	*LOC103345076*	0.8472	0.388	0.044	1.306e-75	3.016e-71
9	Neutrophils	*SNX18*	0.8458	0.515	0.151	2.053e-29	4.740e-25
9	Neutrophils	*PREX1*	0.8457	0.336	0.018	2.378e-130	5.491e-126
9	Neutrophils	*NCF1*	0.8457	0.261	0.010	1.906e-138	4.401e-134
9	Neutrophils	*SLC49A4*	0.8440	0.410	0.140	3.065e-18	7.078e-14
9	Neutrophils	*MS4A3*	0.8429	0.179	0.000	0	0
9	Neutrophils	*LGALS8*	0.8425	0.433	0.142	1.384e-20	3.196e-16
9	Neutrophils	*SPSB2*	0.8419	0.440	0.141	2.835e-23	6.547e-19
9	Neutrophils	*SOS2*	0.8386	0.560	0.239	2.560e-17	5.912e-13

Original cluster	Annotated cell type	Gene	avg_log2FC	pct.1	pct.2	Raw P value	Bonferroni-adjusted P value
9	Neutrophils	*CASP1*	0.8385	0.269	0.000	0	0
9	Neutrophils	*UVRAG*	0.8382	0.493	0.218	3.933e-13	9.083e-09
9	Neutrophils	*SIRT7*	0.8382	0.433	0.056	3.123e-72	7.212e-68
9	Neutrophils	*PITPNC1*	0.8379	0.351	0.140	9.428e-12	2.177e-07
9	Neutrophils	*SDCBP*	0.8376	0.791	0.639	6.246e-09	1.442e-04
9	Neutrophils	*MARK3*	0.8371	0.500	0.178	1.964e-21	4.535e-17
9	Neutrophils	*ARF4*	0.8370	0.724	0.521	2.390e-07	5.520e-03
9	Neutrophils	*PKM*	0.8352	0.642	0.399	3.171e-12	7.321e-08
9	Neutrophils	*UBAC2*	0.8350	0.418	0.143	1.814e-17	4.190e-13
9	Neutrophils	*KPNA2*	0.8341	0.358	0.043	5.217e-65	1.205e-60
9	Neutrophils	*WDR7*	0.8321	0.448	0.140	6.954e-23	1.606e-18
9	Neutrophils	*IKZF1*	0.8315	0.373	0.007	0	0
9	Neutrophils	*KIAA0513*	0.8310	0.313	0.052	2.467e-39	5.697e-35
9	Neutrophils	*TRIP12*	0.8268	0.507	0.209	5.882e-16	1.358e-11
9	Neutrophils	*SMG1*	0.8259	0.522	0.275	6.015e-09	1.389e-04
9	Neutrophils	*DIS3L2*	0.8249	0.448	0.230	4.930e-09	1.138e-04
9	Neutrophils	*ABTB1*	0.8247	0.328	0.051	1.738e-44	4.012e-40
9	Neutrophils	*PTPN12*	0.8244	0.425	0.227	2.956e-08	6.826e-04
9	Neutrophils	*JAZF1*	0.8242	0.567	0.339	9.948e-08	2.297e-03
9	Neutrophils	*TUBA4A*	0.8239	0.321	0.037	1.604e-60	3.705e-56
9	Neutrophils	*NAAA*	0.8239	0.463	0.223	7.708e-11	1.780e-06
9	Neutrophils	*MAP3K5*	0.8227	0.343	0.061	4.875e-38	1.126e-33
9	Neutrophils	*ETS2*	0.8217	0.522	0.171	9.804e-25	2.264e-20
9	Neutrophils	*MOB3A*	0.8216	0.463	0.159	3.491e-20	8.062e-16
9	Neutrophils	*SNX9*	0.8214	0.403	0.185	3.393e-12	7.836e-08
9	Neutrophils	*KCNN3*	0.8208	0.172	0.026	9.262e-25	2.139e-20
9	Neutrophils	*RIN3*	0.8207	0.328	0.019	2.639e-123	6.094e-119
9	Neutrophils	*DENND1B*	0.8201	0.410	0.154	3.090e-15	7.136e-11
9	Neutrophils	*NUCB2*	0.8185	0.582	0.269	2.241e-16	5.175e-12
9	Neutrophils	*HSD17B12*	0.8165	0.396	0.184	3.987e-09	9.206e-05
9	Neutrophils	*LOC138849334*	0.8140	0.164	0.001	2.363e-266	5.457e-262
9	Neutrophils	*RALGPS1*	0.8138	0.299	0.033	9.327e-59	2.154e-54
9	Neutrophils	*RAB31*	0.8136	0.381	0.114	1.089e-20	2.515e-16
9	Neutrophils	*DGKD*	0.8129	0.269	0.061	2.235e-23	5.160e-19
9	Neutrophils	*REPS2*	0.8105	0.239	0.043	1.788e-26	4.128e-22
9	Neutrophils	*NFIL3*	0.8088	0.403	0.148	1.028e-16	2.375e-12
9	Neutrophils	*RAB37*	0.8082	0.216	0.002	1.722e-252	3.976e-248
9	Neutrophils	*LOC138844296*	0.8070	0.410	0.079	2.259e-41	5.216e-37
9	Neutrophils	*MFNG*	0.8064	0.410	0.043	8.294e-84	1.915e-79
9	Neutrophils	*LAT2*	0.8057	0.313	0.005	2.110e-278	4.873e-274

Original cluster	Annotated cell type	Gene	avg_log2FC	pct.1	pct.2	Raw P value	Bonferroni-adjusted P value
9	Neutrophils	*DRAM1*	0.8056	0.306	0.039	1.358e-51	3.136e-47
9	Neutrophils	*PDZD8*	0.8053	0.403	0.152	8.654e-15	1.998e-10
9	Neutrophils	*HPRT1*	0.8029	0.388	0.059	2.962e-54	6.839e-50
9	Neutrophils	*GSAP*	0.8021	0.284	0.031	6.886e-57	1.590e-52
9	Neutrophils	*TGFA*	0.8010	0.157	0.006	1.315e-87	3.037e-83
9	Neutrophils	*PILRA*	0.7973	0.261	0.004	2.884e-263	6.659e-259
9	Neutrophils	*ORM1*	0.7961	0.187	0.001	1.037e-289	2.394e-285
9	Neutrophils	*ADAM8*	0.7954	0.194	0.014	7.855e-59	1.814e-54
9	Neutrophils	*MBP*	0.7948	0.328	0.021	1.163e-112	2.686e-108
9	Neutrophils	*CFP*	0.7932	0.358	0.009	4.429e-259	1.023e-254
9	Neutrophils	*RLF*	0.7931	0.455	0.229	3.047e-09	7.036e-05
9	Neutrophils	*HIF1A*	0.7928	0.478	0.190	1.967e-15	4.541e-11
9	Neutrophils	*SEC14L1*	0.7897	0.366	0.086	1.354e-29	3.128e-25
9	Neutrophils	*BTG1*	0.7889	0.761	0.663	1.459e-08	3.368e-04
9	Neutrophils	*MGAT5*	0.7874	0.410	0.185	8.870e-12	2.048e-07
9	Neutrophils	*ICAM3*	0.7872	0.269	0.012	5.579e-123	1.288e-118
9	Neutrophils	*LOC138846993*	0.7840	0.209	0.001	1.860e-282	4.296e-278
9	Neutrophils	*AP1S2*	0.7834	0.567	0.303	7.137e-13	1.648e-08
9	Neutrophils	*AKNA*	0.7819	0.336	0.022	1.374e-112	3.173e-108
9	Neutrophils	*SLC16A6*	0.7779	0.216	0.007	2.456e-127	5.672e-123
9	Neutrophils	*MARCHF1*	0.7772	0.291	0.009	3.443e-177	7.951e-173
9	Neutrophils	*STK10*	0.7771	0.336	0.031	5.152e-81	1.190e-76
9	Neutrophils	*PPP1CA*	0.7769	0.597	0.232	3.098e-22	7.155e-18
9	Neutrophils	*HCAR2*	0.7763	0.164	0.016	1.998e-38	4.614e-34
9	Neutrophils	*CTSA*	0.7742	0.485	0.226	6.277e-14	1.450e-09
9	Neutrophils	*RHOH*	0.7720	0.209	0.027	3.015e-34	6.963e-30
9	Neutrophils	*PTP4A1*	0.7714	0.545	0.274	3.711e-11	8.569e-07
9	Neutrophils	*PSEN1*	0.7714	0.343	0.130	1.790e-12	4.133e-08
9	Neutrophils	*TLN1*	0.7709	0.619	0.331	1.348e-13	3.114e-09
9	Neutrophils	*LNPEP*	0.7702	0.425	0.114	4.490e-28	1.037e-23
9	Neutrophils	*KCNJ2*	0.7700	0.194	0.016	1.730e-53	3.994e-49
9	Neutrophils	*ROCK1*	0.7690	0.731	0.423	1.794e-12	4.142e-08
9	Neutrophils	*LIPN*	0.7685	0.119	0.000	4.135e-239	9.548e-235
9	Neutrophils	*ZCCHC2*	0.7684	0.328	0.102	2.554e-18	5.898e-14
9	Neutrophils	*RARA*	0.7651	0.313	0.059	3.804e-34	8.783e-30
9	Neutrophils	*TGFBI*	0.7634	0.358	0.105	8.473e-22	1.957e-17
9	Neutrophils	*LOC100341358*	0.7627	0.172	0.001	1.724e-261	3.982e-257
9	Neutrophils	*NPEPPS*	0.7623	0.567	0.284	2.696e-12	6.225e-08
9	Neutrophils	*CDK17*	0.7616	0.373	0.168	3.165e-09	7.309e-05
9	Neutrophils	*USP12*	0.7615	0.433	0.177	1.507e-14	3.479e-10

Original cluster	Annotated cell type	Gene	avg_log2FC	pct.1	pct.2	Raw P value	Bonferroni-adjusted P value
9	Neutrophils	*TBL1XR1*	0.7615	0.716	0.513	6.440e-08	1.487e-03
9	Neutrophils	*IQSEC1*	0.7606	0.343	0.092	1.288e-22	2.975e-18
9	Neutrophils	*ARHGAP25*	0.7590	0.313	0.020	2.636e-107	6.086e-103
9	Neutrophils	*MAP4K4*	0.7568	0.612	0.400	3.742e-07	8.640e-03
9	Neutrophils	*UBE2Q2*	0.7561	0.500	0.202	1.035e-15	2.390e-11
9	Neutrophils	*SP3*	0.7557	0.709	0.400	1.320e-11	3.048e-07
9	Neutrophils	*STX3*	0.7544	0.164	0.014	5.089e-44	1.175e-39
9	Neutrophils	*SPTLC2*	0.7523	0.299	0.089	4.879e-17	1.127e-12
9	Neutrophils	*GRK2*	0.7517	0.403	0.086	2.539e-36	5.863e-32
9	Neutrophils	*LOC138850522*	0.7505	0.276	0.008	3.152e-175	7.279e-171
9	Neutrophils	*LOC100341088*	0.7498	0.164	0.001	2.553e-247	5.896e-243
9	Neutrophils	*LOC103347294*	0.7494	0.254	0.000	0	0
9	Neutrophils	*FPR1*	0.7480	0.157	0.000	0	0
9	Neutrophils	*MAP2K3*	0.7476	0.373	0.064	7.940e-44	1.834e-39
9	Neutrophils	*PXN*	0.7454	0.388	0.103	6.093e-26	1.407e-21
9	Neutrophils	*MTMR6*	0.7446	0.381	0.109	6.741e-22	1.557e-17
9	Neutrophils	*LOC138843868*	0.7444	0.209	0.002	5.552e-251	1.282e-246
9	Neutrophils	*PACS1*	0.7434	0.396	0.131	5.119e-19	1.182e-14
9	Neutrophils	*ALOX5*	0.7433	0.246	0.015	1.425e-88	3.291e-84
9	Neutrophils	*INAFM2*	0.7431	0.276	0.041	2.477e-39	5.719e-35
9	Neutrophils	*OAZ1*	0.7420	0.828	0.805	1.784e-09	4.120e-05
9	Neutrophils	*BID*	0.7411	0.358	0.045	1.532e-60	3.539e-56
9	Neutrophils	*MCEMP1*	0.7399	0.209	0.000	0	0
9	Neutrophils	*EHBP1L1*	0.7398	0.336	0.022	1.165e-107	2.690e-103
9	Neutrophils	*EMP3*	0.7382	0.687	0.465	1.407e-08	3.250e-04
9	Neutrophils	*ALDOA*	0.7381	0.657	0.464	1.499e-07	3.463e-03
9	Neutrophils	*GPBP1L1*	0.7370	0.604	0.269	1.128e-16	2.604e-12
9	Neutrophils	*EPS15*	0.7354	0.597	0.285	8.218e-15	1.898e-10
9	Neutrophils	*RANBP10*	0.7352	0.254	0.066	1.983e-17	4.580e-13
9	Neutrophils	*SIPA1*	0.7307	0.388	0.036	2.673e-92	6.173e-88
9	Neutrophils	*OGFRL1*	0.7303	0.440	0.131	1.250e-24	2.886e-20
9	Neutrophils	*LOC100342498*	0.7297	0.261	0.003	7.926e-285	1.830e-280
9	Neutrophils	*PIK3AP1*	0.7297	0.351	0.047	2.248e-55	5.192e-51
9	Neutrophils	*HERC4*	0.7295	0.500	0.257	7.091e-09	1.637e-04
9	Neutrophils	*SMURF2*	0.7277	0.515	0.241	8.606e-12	1.987e-07
9	Neutrophils	*JARID2*	0.7273	0.373	0.180	1.323e-08	3.056e-04
9	Neutrophils	*RAB4B*	0.7261	0.396	0.050	4.669e-67	1.078e-62
9	Neutrophils	*SPECC1*	0.7257	0.276	0.067	1.315e-20	3.036e-16
9	Neutrophils	*HK2*	0.7249	0.194	0.011	4.314e-75	9.962e-71
9	Neutrophils	*GNG5*	0.7235	0.709	0.481	2.759e-12	6.370e-08

Original cluster	Annotated cell type	Gene	avg_log2FC	pct.1	pct.2	Raw P value	Bonferroni-adjusted P value
9	Neutrophils	*LOC100342250*	0.7230	0.194	0.005	3.292e-135	7.603e-131
9	Neutrophils	*CENPP*	0.7221	0.112	0.029	3.107e-08	7.174e-04
9	Neutrophils	*FAM111A*	0.7220	0.336	0.058	1.191e-39	2.750e-35
9	Neutrophils	*SWAP70*	0.7220	0.351	0.135	1.053e-12	2.432e-08
9	Neutrophils	*RAB11A*	0.7209	0.448	0.124	6.260e-28	1.446e-23
9	Neutrophils	*PLCG2*	0.7204	0.254	0.004	3.642e-227	8.411e-223
9	Neutrophils	*LTB4R*	0.7177	0.269	0.003	1.208e-296	2.790e-292
9	Neutrophils	*RBKS*	0.7176	0.299	0.061	2.393e-28	5.525e-24
9	Neutrophils	*RBM6*	0.7159	0.560	0.322	6.672e-08	1.541e-03
9	Neutrophils	*EPB41*	0.7157	0.500	0.258	3.415e-09	7.886e-05
9	Neutrophils	*PHF20L1*	0.7151	0.575	0.278	1.475e-13	3.407e-09
9	Neutrophils	*MGAT4A*	0.7151	0.313	0.078	2.085e-22	4.814e-18
9	Neutrophils	*SH2D1B*	0.7150	0.187	0.002	2.944e-199	6.799e-195
9	Neutrophils	*TIPARP*	0.7146	0.530	0.336	8.185e-07	1.890e-02
9	Neutrophils	*RAB5B*	0.7139	0.157	0.051	7.471e-08	1.725e-03
9	Neutrophils	*DHRS1*	0.7136	0.507	0.239	2.816e-12	6.503e-08
9	Neutrophils	*PCNX1*	0.7116	0.530	0.248	1.589e-12	3.668e-08
9	Neutrophils	*CNTRL*	0.7111	0.493	0.226	2.511e-12	5.797e-08
9	Neutrophils	*NIBAN1*	0.7088	0.179	0.015	1.707e-46	3.943e-42
9	Neutrophils	*SLC4A7*	0.7071	0.328	0.169	4.177e-07	9.646e-03
9	Neutrophils	*OSBPL11*	0.7071	0.328	0.120	2.857e-12	6.596e-08
9	Neutrophils	*BRD10*	0.7052	0.537	0.304	8.573e-08	1.980e-03
9	Neutrophils	*CSNK1G1*	0.7031	0.388	0.208	3.043e-07	7.026e-03
9	Neutrophils	*LOC100345591*	0.7024	0.634	0.295	3.925e-17	9.063e-13
9	Neutrophils	*PLEKHA2*	0.7018	0.396	0.098	1.826e-28	4.217e-24
9	Neutrophils	*PLCB2*	0.6986	0.269	0.002	0	0
9	Neutrophils	*COMMD6*	0.6966	0.507	0.173	1.020e-22	2.356e-18
9	Neutrophils	*HK3*	0.6962	0.216	0.001	0	0
9	Neutrophils	*PLEKHB2*	0.6958	0.224	0.000	0	0
9	Neutrophils	*NFE2*	0.6955	0.284	0.000	0	0
9	Neutrophils	*N4BP2L1*	0.6927	0.328	0.160	7.639e-08	1.764e-03
9	Neutrophils	*NR2C2*	0.6919	0.306	0.130	2.698e-09	6.230e-05
9	Neutrophils	*LARP4B*	0.6916	0.485	0.179	3.222e-18	7.441e-14
9	Neutrophils	*SNX20*	0.6895	0.299	0.008	3.281e-205	7.577e-201
9	Neutrophils	*HHEX*	0.6892	0.224	0.020	2.024e-56	4.673e-52
9	Neutrophils	*BANK1*	0.6884	0.149	0.040	2.466e-10	5.694e-06
9	Neutrophils	*EVI2A*	0.6881	0.291	0.005	4.915e-250	1.135e-245
9	Neutrophils	*TMED8*	0.6873	0.246	0.033	5.412e-39	1.250e-34
9	Neutrophils	*STK38*	0.6852	0.381	0.139	1.064e-15	2.456e-11
9	Neutrophils	*SEC24B*	0.6848	0.440	0.189	5.050e-12	1.166e-07

Original cluster	Annotated cell type	Gene	avg_log2FC	pct.1	pct.2	Raw P value	Bonferroni-adjusted P value
9	Neutrophils	*ARHGAP18*	0.6846	0.321	0.091	5.777e-19	1.334e-14
9	Neutrophils	*ATP1B3*	0.6841	0.418	0.175	5.242e-12	1.210e-07
9	Neutrophils	*KCMF1*	0.6840	0.463	0.219	1.287e-09	2.972e-05
9	Neutrophils	*MAP1LC3B*	0.6833	0.507	0.215	2.234e-14	5.159e-10
9	Neutrophils	*SUSD3*	0.6831	0.351	0.066	6.359e-36	1.468e-31
9	Neutrophils	*MGAM*	0.6821	0.142	0.000	1.240e-283	2.864e-279
9	Neutrophils	*TNFAIP8L2*	0.6807	0.313	0.003	0	0
9	Neutrophils	*CSNK1A1*	0.6775	0.634	0.419	1.289e-07	2.977e-03
9	Neutrophils	*CLEC4E*	0.6755	0.231	0.002	1.804e-295	4.166e-291
9	Neutrophils	*LOC138847422*	0.6752	0.194	0.006	1.311e-117	3.027e-113
9	Neutrophils	*GNAI2*	0.6749	0.806	0.538	1.108e-14	2.559e-10
9	Neutrophils	*C5AR2*	0.6748	0.187	0.004	7.817e-141	1.805e-136
9	Neutrophils	*DBI*	0.6743	0.522	0.297	4.166e-09	9.620e-05
9	Neutrophils	*UBQLN1*	0.6743	0.522	0.196	1.373e-18	3.170e-14
9	Neutrophils	*MAP3K3*	0.6735	0.582	0.216	5.937e-21	1.371e-16
9	Neutrophils	*QKI*	0.6732	0.582	0.417	9.916e-07	2.290e-02
9	Neutrophils	*LOC103349679*	0.6720	0.284	0.049	8.053e-33	1.860e-28
9	Neutrophils	*TAB3*	0.6720	0.269	0.103	9.472e-10	2.187e-05
9	Neutrophils	*PROK2*	0.6709	0.142	0.001	1.777e-223	4.104e-219
9	Neutrophils	*NFATC3*	0.6703	0.403	0.193	8.714e-09	2.012e-04
9	Neutrophils	*DAZAP2*	0.6699	0.522	0.144	7.713e-31	1.781e-26
9	Neutrophils	*FAM241A*	0.6691	0.306	0.076	1.700e-21	3.927e-17
9	Neutrophils	*RALB*	0.6685	0.381	0.121	1.488e-17	3.437e-13
9	Neutrophils	*TBXAS1*	0.6682	0.209	0.001	0	0
9	Neutrophils	*IRF2*	0.6681	0.515	0.282	9.950e-08	2.298e-03
9	Neutrophils	*CSNK1G3*	0.6676	0.455	0.207	1.135e-11	2.621e-07
9	Neutrophils	*NT5C3A*	0.6666	0.463	0.199	1.987e-14	4.588e-10
9	Neutrophils	*KRAS*	0.6662	0.410	0.158	1.938e-14	4.474e-10
9	Neutrophils	*MTMR8*	0.6658	0.231	0.037	6.179e-31	1.427e-26
9	Neutrophils	*CBL*	0.6646	0.358	0.076	6.495e-32	1.500e-27
9	Neutrophils	*CORO1B*	0.6630	0.410	0.161	1.706e-14	3.939e-10
9	Neutrophils	*FKBP5*	0.6619	0.239	0.035	3.986e-34	9.205e-30
9	Neutrophils	*EP300*	0.6618	0.701	0.442	1.356e-06	3.132e-02
9	Neutrophils	*LOC127494001*	0.6614	0.187	0.056	2.121e-10	4.898e-06
9	Neutrophils	*PGAM1*	0.6614	0.358	0.092	2.907e-25	6.712e-21
9	Neutrophils	*ATP6AP2*	0.6602	0.515	0.202	5.699e-16	1.316e-11
9	Neutrophils	*GIT2*	0.6594	0.381	0.120	6.131e-19	1.416e-14
9	Neutrophils	*FRYL*	0.6594	0.470	0.238	1.207e-08	2.788e-04
9	Neutrophils	*SPPL2A*	0.6585	0.463	0.204	1.837e-12	4.242e-08
9	Neutrophils	*PIK3R6*	0.6581	0.179	0.003	6.817e-172	1.574e-167

Original cluster	Annotated cell type	Gene	avg_log2FC	pct.1	pct.2	Raw P value	Bonferroni-adjusted P value
9	Neutrophils	*RB1*	0.6576	0.485	0.271	7.508e-08	1.734e-03
9	Neutrophils	*LOC138846723*	0.6571	0.433	0.118	3.267e-27	7.544e-23
9	Neutrophils	*KPNA4*	0.6564	0.582	0.244	6.472e-17	1.494e-12
9	Neutrophils	*AAK1*	0.6558	0.381	0.145	9.606e-13	2.218e-08
9	Neutrophils	*NUS1*	0.6555	0.358	0.131	3.080e-13	7.112e-09
9	Neutrophils	*CFLAR*	0.6555	0.299	0.114	1.514e-10	3.496e-06
9	Neutrophils	*ITGA4*	0.6550	0.313	0.046	2.722e-44	6.285e-40
9	Neutrophils	*DAPP1*	0.6540	0.224	0.003	3.870e-215	8.937e-211
9	Neutrophils	*STAU1*	0.6534	0.358	0.158	2.539e-09	5.863e-05
9	Neutrophils	*LAMTOR5*	0.6523	0.560	0.270	2.904e-13	6.706e-09
9	Neutrophils	*BBC3*	0.6510	0.284	0.047	1.883e-35	4.349e-31
9	Neutrophils	*ETHE1*	0.6505	0.396	0.092	6.145e-31	1.419e-26
9	Neutrophils	*ETNK1*	0.6502	0.590	0.391	3.387e-08	7.821e-04
9	Neutrophils	*CEACAM1*	0.6498	0.157	0.001	2.278e-197	5.260e-193
9	Neutrophils	*CALM1*	0.6477	0.821	0.746	5.649e-11	1.304e-06
9	Neutrophils	*FBXO9*	0.6471	0.388	0.175	7.831e-10	1.808e-05
9	Neutrophils	*MLANA*	0.6452	0.172	0.012	1.165e-55	2.690e-51
9	Neutrophils	*POLB*	0.6436	0.418	0.098	5.722e-31	1.321e-26
9	Neutrophils	*IL2RG*	0.6428	0.201	0.008	3.053e-98	7.051e-94
9	Neutrophils	*GRK3*	0.6410	0.246	0.011	1.090e-117	2.517e-113
9	Neutrophils	*LOC100350168*	0.6399	0.187	0.000	0	0
9	Neutrophils	*WDR1*	0.6399	0.448	0.128	8.144e-27	1.881e-22
9	Neutrophils	*TBC1D15*	0.6377	0.403	0.205	3.438e-08	7.940e-04
9	Neutrophils	*LOC138846623*	0.6376	0.231	0.000	0	0
9	Neutrophils	*SEPTIN5*	0.6363	0.172	0.028	3.850e-22	8.890e-18
9	Neutrophils	*WASF2*	0.6344	0.500	0.248	4.184e-10	9.662e-06
9	Neutrophils	*GAS7*	0.6330	0.343	0.076	4.934e-29	1.139e-24
9	Neutrophils	*SIAH2*	0.6328	0.328	0.099	1.505e-17	3.476e-13
9	Neutrophils	*ADAM15*	0.6328	0.231	0.045	1.675e-23	3.868e-19
9	Neutrophils	*ADSS2*	0.6312	0.418	0.174	4.571e-12	1.056e-07
9	Neutrophils	*UNC93B1*	0.6310	0.343	0.046	5.949e-55	1.374e-50
9	Neutrophils	*LOC138846022*	0.6304	0.201	0.000	0	0
9	Neutrophils	*RP2*	0.6299	0.299	0.039	7.978e-48	1.842e-43
9	Neutrophils	*GPI*	0.6288	0.493	0.265	5.056e-10	1.167e-05
9	Neutrophils	*LOC100347239*	0.6248	0.187	0.008	6.577e-94	1.519e-89
9	Neutrophils	*SLA*	0.6247	0.187	0.001	6.971e-264	1.610e-259
9	Neutrophils	*LAMTOR3*	0.6233	0.410	0.132	7.584e-19	1.751e-14
9	Neutrophils	*ULK1*	0.6229	0.269	0.063	4.136e-21	9.551e-17
9	Neutrophils	*IFNGR2*	0.6223	0.261	0.018	9.298e-82	2.147e-77
9	Neutrophils	*CHFR*	0.6222	0.254	0.057	2.160e-21	4.988e-17

Original cluster	Annotated cell type	Gene	avg_log2FC	pct.1	pct.2	Raw P value	Bonferroni-adjusted P value
9	Neutrophils	*TMEM120A*	0.6222	0.254	0.034	4.482e-40	1.035e-35
9	Neutrophils	*PRKCH*	0.6211	0.291	0.064	4.951e-24	1.143e-19
9	Neutrophils	*CARD9*	0.6196	0.313	0.033	2.936e-63	6.779e-59
9	Neutrophils	*KLF13*	0.6184	0.433	0.172	2.766e-14	6.386e-10
9	Neutrophils	*INPP4A*	0.6183	0.276	0.067	1.025e-20	2.368e-16
9	Neutrophils	*TMX1*	0.6174	0.478	0.185	2.760e-16	6.374e-12
9	Neutrophils	*ZCWPW1*	0.6166	0.164	0.056	9.061e-08	2.092e-03
9	Neutrophils	*PKN1*	0.6140	0.358	0.081	6.791e-29	1.568e-24
9	Neutrophils	*FES*	0.6137	0.321	0.048	3.792e-44	8.756e-40
9	Neutrophils	*ZNRF2*	0.6135	0.448	0.104	1.118e-34	2.582e-30
9	Neutrophils	*EPSTI1*	0.6130	0.254	0.053	8.086e-24	1.867e-19
9	Neutrophils	*POLN*	0.6126	0.149	0.012	5.105e-43	1.179e-38
9	Neutrophils	*PIK3C2A*	0.6124	0.448	0.213	3.562e-10	8.226e-06
9	Neutrophils	*PNP*	0.6104	0.343	0.149	4.167e-09	9.622e-05
9	Neutrophils	*STK24*	0.6097	0.493	0.179	1.169e-18	2.699e-14
9	Neutrophils	*CDK2AP2*	0.6091	0.403	0.100	6.984e-29	1.613e-24
9	Neutrophils	*CD274*	0.6078	0.194	0.049	5.638e-14	1.302e-09
9	Neutrophils	*CASP10*	0.6078	0.224	0.031	4.699e-35	1.085e-30
9	Neutrophils	*MRTFA*	0.6077	0.358	0.154	6.593e-10	1.522e-05
9	Neutrophils	*SPP1*	0.6065	0.134	0.006	1.996e-66	4.610e-62
9	Neutrophils	*CLIP1*	0.6051	0.396	0.137	6.491e-17	1.499e-12
9	Neutrophils	*CPEB4*	0.6043	0.396	0.125	1.139e-18	2.630e-14
9	Neutrophils	*VPS4B*	0.6038	0.507	0.170	2.139e-21	4.940e-17
9	Neutrophils	*LOC138843103*	0.6036	0.157	0.001	2.685e-225	6.200e-221
9	Neutrophils	*DENND1A*	0.6030	0.403	0.225	1.123e-06	2.593e-02
9	Neutrophils	*RBM7*	0.6028	0.448	0.214	1.910e-10	4.410e-06
9	Neutrophils	*PATL1*	0.6017	0.284	0.118	1.459e-08	3.368e-04
9	Neutrophils	*ICAM1*	0.6012	0.269	0.077	4.209e-16	9.720e-12
9	Neutrophils	*ISOC1*	0.6006	0.254	0.043	1.987e-30	4.588e-26
9	Neutrophils	*FGGY*	0.6003	0.276	0.124	1.190e-07	2.747e-03
9	Neutrophils	*LRRFIP2*	0.5995	0.470	0.225	3.327e-10	7.684e-06
9	Neutrophils	*SNX29*	0.5974	0.291	0.091	9.634e-15	2.225e-10
9	Neutrophils	*PEAK3*	0.5971	0.194	0.000	0	0
9	Neutrophils	*EML4*	0.5951	0.507	0.250	2.382e-10	5.500e-06
9	Neutrophils	*PIK3CD*	0.5948	0.246	0.006	2.074e-171	4.790e-167
9	Neutrophils	*GALNT1*	0.5941	0.537	0.290	3.115e-09	7.193e-05
9	Neutrophils	*EPS15L1*	0.5933	0.239	0.041	4.578e-28	1.057e-23
9	Neutrophils	*ARF6*	0.5920	0.567	0.296	5.995e-13	1.384e-08
9	Neutrophils	*CAPZA2*	0.5916	0.724	0.560	1.468e-07	3.389e-03
9	Neutrophils	*STAT4*	0.5913	0.112	0.026	1.096e-09	2.531e-05

Original cluster	Annotated cell type	Gene	avg_log2FC	pct.1	pct.2	Raw P value	Bonferroni-adjusted P value
9	Neutrophils	*PSME1*	0.5908	0.463	0.233	1.825e-10	4.214e-06
9	Neutrophils	*VPS36*	0.5884	0.410	0.150	1.132e-15	2.613e-11
9	Neutrophils	*IRAK4*	0.5862	0.336	0.087	8.601e-23	1.986e-18
9	Neutrophils	*GRK6*	0.5860	0.209	0.050	5.795e-16	1.338e-11
9	Neutrophils	*G6PD*	0.5856	0.261	0.015	2.643e-96	6.102e-92
9	Neutrophils	*PPP4C*	0.5856	0.552	0.236	6.964e-15	1.608e-10
9	Neutrophils	*GPR34*	0.5837	0.142	0.003	1.298e-116	2.998e-112
9	Neutrophils	*SNX2*	0.5833	0.634	0.423	1.668e-08	3.851e-04
9	Neutrophils	*SH3BP1*	0.5826	0.224	0.019	2.413e-56	5.571e-52
9	Neutrophils	*MYO9B*	0.5805	0.261	0.039	1.034e-36	2.387e-32
9	Neutrophils	*SLC6A6*	0.5798	0.231	0.027	1.792e-42	4.139e-38
9	Neutrophils	*SPOPL*	0.5795	0.403	0.159	5.732e-13	1.324e-08
9	Neutrophils	*CEBPG*	0.5792	0.425	0.132	1.697e-20	3.918e-16
9	Neutrophils	*CCNJL*	0.5786	0.142	0.004	5.884e-91	1.359e-86
9	Neutrophils	*MTPN*	0.5764	0.515	0.246	1.042e-10	2.406e-06
9	Neutrophils	*MTF1*	0.5762	0.313	0.069	7.350e-27	1.697e-22
9	Neutrophils	*C19H16orf54*	0.5761	0.209	0.008	5.319e-114	1.228e-109
9	Neutrophils	*ENTPD1*	0.5755	0.358	0.068	5.402e-36	1.247e-31
9	Neutrophils	*SERPINE2*	0.5738	0.209	0.072	9.777e-10	2.258e-05
9	Neutrophils	*FRAT2*	0.5737	0.306	0.032	3.290e-64	7.597e-60
9	Neutrophils	*COP1*	0.5724	0.545	0.334	1.039e-06	2.400e-02
9	Neutrophils	*BAZ2A*	0.5723	0.246	0.102	2.768e-07	6.391e-03
9	Neutrophils	*LOC138846406*	0.5717	0.216	0.000	0	0
9	Neutrophils	*DOK3*	0.5706	0.187	0.002	3.981e-230	9.193e-226
9	Neutrophils	*RIOK1*	0.5704	0.231	0.074	1.736e-11	4.008e-07
9	Neutrophils	*LOC100349893*	0.5701	0.313	0.033	2.545e-63	5.876e-59
9	Neutrophils	*EDEM3*	0.5698	0.187	0.060	9.786e-10	2.260e-05
9	Neutrophils	*KLF2*	0.5694	0.687	0.486	1.881e-06	4.343e-02
9	Neutrophils	*TGFB1*	0.5684	0.351	0.068	4.357e-35	1.006e-30
9	Neutrophils	*LOC108178205*	0.5668	0.104	0.000	3.126e-195	7.219e-191
9	Neutrophils	*C9orf72*	0.5661	0.313	0.077	2.228e-23	5.145e-19
9	Neutrophils	*ATP6V1A*	0.5657	0.328	0.105	1.175e-16	2.714e-12
9	Neutrophils	*TLCD4*	0.5647	0.231	0.064	1.185e-14	2.736e-10
9	Neutrophils	*SETD2*	0.5642	0.485	0.256	5.376e-08	1.242e-03
9	Neutrophils	*TAP1*	0.5638	0.261	0.017	7.113e-86	1.643e-81
9	Neutrophils	*DIAPH3*	0.5623	0.119	0.027	1.649e-10	3.808e-06
9	Neutrophils	*JDP2*	0.5619	0.410	0.190	1.122e-09	2.590e-05
9	Neutrophils	*AKIRIN1*	0.5609	0.328	0.088	5.025e-21	1.160e-16
9	Neutrophils	*SKIL*	0.5602	0.358	0.163	6.846e-09	1.581e-04
9	Neutrophils	*KDM5A*	0.5594	0.560	0.323	9.968e-07	2.302e-02

Original cluster	Annotated cell type	Gene	avg_log2FC	pct.1	pct.2	Raw P value	Bonferroni-adjusted P value
9	Neutrophils	*STX8*	0.5594	0.306	0.125	5.784e-10	1.336e-05
9	Neutrophils	*RSAD2*	0.5593	0.127	0.010	4.437e-37	1.025e-32
9	Neutrophils	*LAMTOR4*	0.5574	0.627	0.375	9.582e-08	2.213e-03
9	Neutrophils	*GNA15*	0.5574	0.284	0.004	3.506e-301	8.095e-297
9	Neutrophils	*RGS18*	0.5572	0.142	0.002	8.301e-132	1.917e-127
9	Neutrophils	*PDLIM7*	0.5568	0.284	0.099	3.493e-12	8.066e-08
9	Neutrophils	*ARMH3*	0.5559	0.351	0.122	5.566e-14	1.285e-09
9	Neutrophils	*LOC100350480*	0.5554	0.216	0.000	0	0
9	Neutrophils	*RAB22A*	0.5552	0.336	0.110	4.375e-15	1.010e-10
9	Neutrophils	*IFNAR1*	0.5538	0.343	0.122	1.663e-13	3.840e-09
9	Neutrophils	*KLHL28*	0.5536	0.343	0.148	2.429e-09	5.610e-05
9	Neutrophils	*BTK*	0.5517	0.224	0.002	7.721e-270	1.783e-265
9	Neutrophils	*MAPK1*	0.5515	0.418	0.198	4.101e-09	9.470e-05
9	Neutrophils	*LOC138843849*	0.5513	0.164	0.043	9.731e-12	2.247e-07
9	Neutrophils	*LOC138846613*	0.5504	0.216	0.010	1.050e-99	2.425e-95
9	Neutrophils	*FOXN2*	0.5503	0.299	0.121	1.806e-09	4.171e-05
9	Neutrophils	*RAB6A*	0.5501	0.403	0.167	1.288e-11	2.975e-07
9	Neutrophils	*UBE2A*	0.5492	0.321	0.107	4.978e-14	1.149e-09
9	Neutrophils	*SDF2L1*	0.5481	0.246	0.019	1.274e-69	2.942e-65
9	Neutrophils	*ITPR1*	0.5472	0.321	0.110	7.213e-14	1.666e-09
9	Neutrophils	*LIN54*	0.5464	0.291	0.079	3.499e-18	8.080e-14
9	Neutrophils	*B4GALT1*	0.5455	0.187	0.038	6.524e-18	1.506e-13
9	Neutrophils	*RAP2C*	0.5448	0.224	0.033	9.438e-33	2.179e-28
9	Neutrophils	*ANKRD11*	0.5443	0.396	0.194	1.320e-07	3.049e-03
9	Neutrophils	*RAB5C*	0.5443	0.373	0.178	8.138e-08	1.879e-03
9	Neutrophils	*GGNBP2*	0.5438	0.522	0.281	1.041e-07	2.403e-03
9	Neutrophils	*TBC1D8*	0.5425	0.239	0.069	8.574e-14	1.980e-09
9	Neutrophils	*ADIPOR2*	0.5413	0.306	0.125	1.293e-09	2.985e-05
9	Neutrophils	*PRNP*	0.5412	0.284	0.138	6.230e-07	1.439e-02
9	Neutrophils	*HEXA*	0.5407	0.440	0.149	1.800e-18	4.157e-14
9	Neutrophils	*NEDD4L*	0.5406	0.299	0.125	5.123e-09	1.183e-04
9	Neutrophils	*PPP2R5A*	0.5406	0.455	0.226	7.569e-09	1.748e-04
9	Neutrophils	*SNAP23*	0.5398	0.351	0.120	4.714e-15	1.089e-10
9	Neutrophils	*TSPAN14*	0.5384	0.209	0.048	1.897e-17	4.381e-13
9	Neutrophils	*DENND11*	0.5379	0.127	0.022	5.632e-16	1.301e-11
9	Neutrophils	*PRAM1*	0.5365	0.194	0.002	1.164e-236	2.687e-232
9	Neutrophils	*UBAP1*	0.5364	0.373	0.134	2.998e-14	6.923e-10
9	Neutrophils	*ABHD5*	0.5362	0.276	0.064	9.823e-22	2.268e-17
9	Neutrophils	*MCMBP*	0.5361	0.276	0.078	3.221e-16	7.438e-12
9	Neutrophils	*NADK*	0.5352	0.231	0.029	3.223e-39	7.442e-35

Original cluster	Annotated cell type	Gene	avg_log2FC	pct.1	pct.2	Raw P value	Bonferroni-adjusted P value
9	Neutrophils	*KCNAB2*	0.5348	0.194	0.026	2.294e-30	5.298e-26
9	Neutrophils	*CCDC125*	0.5344	0.157	0.027	6.658e-19	1.537e-14
9	Neutrophils	*CD36*	0.5340	0.104	0.007	1.265e-33	2.921e-29
9	Neutrophils	*FOXN3*	0.5340	0.552	0.278	1.171e-10	2.705e-06
9	Neutrophils	*HPS3*	0.5335	0.358	0.049	6.679e-54	1.542e-49
9	Neutrophils	*MYBPC1*	0.5331	0.179	0.017	1.172e-42	2.707e-38
9	Neutrophils	*LOC138847411*	0.5319	0.313	0.094	1.011e-16	2.334e-12
9	Neutrophils	*MED14*	0.5318	0.284	0.116	9.126e-09	2.107e-04
9	Neutrophils	*WDR37*	0.5317	0.313	0.116	1.812e-11	4.184e-07
9	Neutrophils	*ANKRD17*	0.5311	0.619	0.412	1.172e-06	2.707e-02
9	Neutrophils	*CCDC88B*	0.5294	0.134	0.006	3.476e-66	8.027e-62
9	Neutrophils	*PTPN2*	0.5292	0.440	0.145	1.714e-18	3.959e-14
9	Neutrophils	*CNOT1*	0.5291	0.463	0.203	3.556e-12	8.212e-08
9	Neutrophils	*LPXN*	0.5287	0.149	0.017	1.835e-28	4.238e-24
9	Neutrophils	*ELK4*	0.5285	0.410	0.174	4.034e-10	9.315e-06
9	Neutrophils	*CAMK1D*	0.5282	0.261	0.068	2.014e-17	4.650e-13
9	Neutrophils	*SLC28A3*	0.5282	0.112	0.001	7.948e-145	1.835e-140
9	Neutrophils	*USP4*	0.5271	0.254	0.090	3.173e-10	7.328e-06
9	Neutrophils	*ATP7A*	0.5269	0.321	0.138	6.432e-09	1.485e-04
9	Neutrophils	*BCL10*	0.5260	0.328	0.096	5.229e-18	1.207e-13
9	Neutrophils	*RREB1*	0.5253	0.231	0.070	2.994e-12	6.915e-08
9	Neutrophils	*RPS6KA1*	0.5243	0.216	0.003	4.566e-224	1.054e-219
9	Neutrophils	*CERT1*	0.5226	0.358	0.162	1.044e-08	2.412e-04
9	Neutrophils	*IRF8*	0.5209	0.239	0.008	8.523e-133	1.968e-128
9	Neutrophils	*CD101*	0.5190	0.157	0.007	1.026e-73	2.370e-69
9	Neutrophils	*LOC138846622*	0.5188	0.209	0.000	0	0
9	Neutrophils	*LOC138850055*	0.5178	0.164	0.001	4.790e-210	1.106e-205
9	Neutrophils	*TRDMT1*	0.5170	0.269	0.093	2.278e-11	5.259e-07
9	Neutrophils	*CNN2*	0.5159	0.306	0.089	9.547e-18	2.205e-13
9	Neutrophils	*PIK3C2B*	0.5150	0.201	0.037	1.437e-22	3.317e-18
9	Neutrophils	*RNF115*	0.5147	0.396	0.202	3.881e-07	8.962e-03
9	Neutrophils	*PRCP*	0.5139	0.127	0.032	1.604e-09	3.703e-05
9	Neutrophils	*UGP2*	0.5135	0.478	0.208	1.389e-11	3.208e-07
9	Neutrophils	*TOP1*	0.5106	0.731	0.568	9.204e-08	2.125e-03
9	Neutrophils	*CX3CR1*	0.5099	0.134	0.001	3.325e-209	7.678e-205
9	Neutrophils	*PHF10*	0.5094	0.299	0.081	1.903e-18	4.395e-14
9	Neutrophils	*TRAPPC9*	0.5092	0.239	0.087	3.381e-09	7.809e-05
9	Neutrophils	*DUSP10*	0.5069	0.134	0.021	2.811e-18	6.492e-14
9	Neutrophils	*C12H15orf48*	0.5068	0.119	0.020	1.530e-15	3.533e-11
9	Neutrophils	*FEM1C*	0.5063	0.276	0.118	7.950e-08	1.836e-03

Original cluster	Annotated cell type	Gene	avg_log2FC	pct.1	pct.2	Raw P value	Bonferroni-adjusted P value
9	Neutrophils	*MPP7*	0.5052	0.224	0.078	5.240e-10	1.210e-05
9	Neutrophils	*LOC127482769*	0.5051	0.104	0.000	2.956e-195	6.827e-191
9	Neutrophils	*LOC100357927*	0.5048	0.239	0.037	4.224e-32	9.754e-28
9	Neutrophils	*GNB2*	0.5032	0.672	0.392	1.892e-08	4.370e-04
9	Neutrophils	*TM6SF1*	0.5025	0.179	0.025	4.133e-27	9.544e-23
9	Neutrophils	*CENPK*	0.4993	0.164	0.024	1.620e-24	3.742e-20
9	Neutrophils	*UBASH3B*	0.4977	0.209	0.025	6.758e-38	1.561e-33
9	Neutrophils	*CCDC61*	0.4975	0.224	0.033	1.735e-32	4.006e-28
9	Neutrophils	*SLC39A11*	0.4956	0.194	0.033	1.290e-22	2.979e-18
9	Neutrophils	*PTPN18*	0.4934	0.239	0.005	3.676e-200	8.489e-196
9	Neutrophils	*CHMP4B*	0.4927	0.321	0.134	1.760e-09	4.065e-05
9	Neutrophils	*GDE1*	0.4927	0.157	0.028	5.216e-18	1.204e-13
9	Neutrophils	*AMZ2*	0.4925	0.291	0.125	7.852e-08	1.813e-03
9	Neutrophils	*KLHL2*	0.4924	0.373	0.143	3.425e-12	7.910e-08
9	Neutrophils	*DDHD1*	0.4915	0.254	0.061	3.276e-19	7.564e-15
9	Neutrophils	*HACD4*	0.4912	0.299	0.042	1.626e-44	3.756e-40
9	Neutrophils	*CASP8*	0.4911	0.239	0.009	1.845e-131	4.259e-127
9	Neutrophils	*SMDT1*	0.4900	0.470	0.186	4.864e-14	1.123e-09
9	Neutrophils	*POU2F2*	0.4891	0.224	0.009	1.568e-114	3.620e-110
9	Neutrophils	*RAB20*	0.4890	0.246	0.004	2.599e-226	6.001e-222
9	Neutrophils	*CCNT1*	0.4876	0.336	0.125	6.881e-12	1.589e-07
9	Neutrophils	*MMP25*	0.4874	0.172	0.000	0	0
9	Neutrophils	*ACP5*	0.4869	0.224	0.007	4.101e-133	9.469e-129
9	Neutrophils	*CDR2*	0.4867	0.187	0.023	1.362e-33	3.145e-29
9	Neutrophils	*CRLF2*	0.4860	0.134	0.000	8.651e-269	1.998e-264
9	Neutrophils	*LOC100344919*	0.4853	0.134	0.017	9.693e-24	2.238e-19
9	Neutrophils	*CDS2*	0.4850	0.269	0.116	8.161e-08	1.885e-03
9	Neutrophils	*GSDMD*	0.4848	0.246	0.006	3.311e-170	7.646e-166
9	Neutrophils	*LOC138846410*	0.4847	0.164	0.000	0	0
9	Neutrophils	*SMIM5*	0.4825	0.127	0.001	4.233e-158	9.776e-154
9	Neutrophils	*DIPK1A*	0.4824	0.201	0.048	8.188e-16	1.891e-11
9	Neutrophils	*SEMA4D*	0.4815	0.269	0.107	8.120e-09	1.875e-04
9	Neutrophils	*B3GNT2*	0.4807	0.276	0.074	1.420e-17	3.280e-13
9	Neutrophils	*ARAP2*	0.4787	0.269	0.031	4.052e-48	9.357e-44
9	Neutrophils	*DNASE1L1*	0.4779	0.239	0.027	7.351e-45	1.697e-40
9	Neutrophils	*ARHGEF18*	0.4778	0.239	0.046	5.731e-25	1.323e-20
9	Neutrophils	*NLRP12*	0.4774	0.164	0.000	0	0
9	Neutrophils	*EAF1*	0.4771	0.187	0.027	5.772e-28	1.333e-23
9	Neutrophils	*AKAP10*	0.4770	0.291	0.117	1.247e-09	2.880e-05
9	Neutrophils	*NCEH1*	0.4765	0.201	0.070	1.127e-08	2.602e-04

Original cluster	Annotated cell type	Gene	avg_log2FC	pct.1	pct.2	Raw P value	Bonferroni-adjusted P value
9	Neutrophils	*DEF6*	0.4763	0.172	0.014	8.672e-48	2.002e-43
9	Neutrophils	*CD86*	0.4759	0.149	0.004	1.128e-93	2.604e-89
9	Neutrophils	*RASGRP4*	0.4752	0.149	0.000	4.783e-284	1.105e-279
9	Neutrophils	*PHTF2*	0.4750	0.269	0.113	1.583e-07	3.656e-03
9	Neutrophils	*CXCL16*	0.4743	0.239	0.010	5.767e-119	1.332e-114
9	Neutrophils	*BIN3*	0.4740	0.261	0.099	8.497e-10	1.962e-05
9	Neutrophils	*WASHC3*	0.4739	0.604	0.367	1.972e-06	4.554e-02
9	Neutrophils	*PDGFC*	0.4733	0.149	0.050	2.430e-07	5.612e-03
9	Neutrophils	*ZYX*	0.4731	0.373	0.166	8.643e-10	1.996e-05
9	Neutrophils	*MAP2K2*	0.4729	0.284	0.090	7.852e-14	1.813e-09
9	Neutrophils	*STK40*	0.4723	0.284	0.083	7.550e-16	1.743e-11
9	Neutrophils	*RNF144A*	0.4697	0.254	0.095	2.117e-09	4.889e-05
9	Neutrophils	*BNIP2*	0.4681	0.440	0.156	3.296e-16	7.610e-12
9	Neutrophils	*ARIH1*	0.4673	0.522	0.270	4.509e-09	1.041e-04
9	Neutrophils	*CHST15*	0.4662	0.104	0.024	2.902e-09	6.701e-05
9	Neutrophils	*NSF*	0.4659	0.299	0.088	1.401e-16	3.236e-12
9	Neutrophils	*AOX1*	0.4657	0.313	0.114	1.495e-11	3.453e-07
9	Neutrophils	*LOC138843262*	0.4656	0.127	0.000	5.998e-254	1.385e-249
9	Neutrophils	*TREML2*	0.4654	0.149	0.000	1.768e-298	4.083e-294
9	Neutrophils	*ACOX1*	0.4650	0.254	0.109	3.633e-07	8.389e-03
9	Neutrophils	*ABHD2*	0.4649	0.269	0.089	2.879e-12	6.648e-08
9	Neutrophils	*LOC138844391*	0.4647	0.172	0.001	1.149e-229	2.653e-225
9	Neutrophils	*GGCT*	0.4647	0.179	0.043	6.632e-14	1.531e-09
9	Neutrophils	*NECAP2*	0.4624	0.328	0.132	6.969e-11	1.609e-06
9	Neutrophils	*LRRC8B*	0.4622	0.172	0.024	8.971e-27	2.072e-22
9	Neutrophils	*GMIP*	0.4620	0.134	0.009	2.668e-42	6.161e-38
9	Neutrophils	*LOC100355470*	0.4613	0.515	0.263	1.568e-07	3.620e-03
9	Neutrophils	*RRAGC*	0.4612	0.284	0.069	3.993e-20	9.220e-16
9	Neutrophils	*TCIRG1*	0.4611	0.231	0.061	2.477e-15	5.721e-11
9	Neutrophils	*TSGA10*	0.4606	0.336	0.094	3.082e-19	7.116e-15
9	Neutrophils	*LOC138845226*	0.4601	0.112	0.001	9.622e-139	2.222e-134
9	Neutrophils	*ARRB2*	0.4599	0.269	0.048	4.768e-30	1.101e-25
9	Neutrophils	*PDCD6*	0.4597	0.552	0.267	1.202e-10	2.775e-06
9	Neutrophils	*AP3S1*	0.4583	0.381	0.185	2.996e-07	6.918e-03
9	Neutrophils	*PLEKHM3*	0.4582	0.261	0.108	5.089e-08	1.175e-03
9	Neutrophils	*ATP6V0B*	0.4576	0.575	0.321	1.910e-08	4.411e-04
9	Neutrophils	*GLCE*	0.4575	0.291	0.123	2.460e-08	5.681e-04
9	Neutrophils	*LOC138850718*	0.4567	0.112	0.016	3.416e-17	7.888e-13
9	Neutrophils	*CD300LF*	0.4566	0.112	0.001	5.143e-159	1.188e-154
9	Neutrophils	*UQCC5*	0.4556	0.127	0.026	1.751e-12	4.044e-08

Original cluster	Annotated cell type	Gene	avg_log2FC	pct.1	pct.2	Raw P value	Bonferroni-adjusted P value
9	Neutrophils	*TOX4*	0.4551	0.366	0.154	1.856e-10	4.286e-06
9	Neutrophils	*ABCC1*	0.4544	0.209	0.019	4.264e-51	9.845e-47
9	Neutrophils	*PLEKHF2*	0.4541	0.351	0.111	6.013e-16	1.389e-11
9	Neutrophils	*PARP4*	0.4541	0.299	0.124	3.789e-09	8.750e-05
9	Neutrophils	*CCDC93*	0.4537	0.321	0.134	1.274e-09	2.941e-05
9	Neutrophils	*DHRS7*	0.4534	0.216	0.056	9.721e-15	2.245e-10
9	Neutrophils	*TBC1D10C*	0.4534	0.164	0.011	8.297e-54	1.916e-49
9	Neutrophils	*LRRC25*	0.4526	0.216	0.006	1.252e-135	2.891e-131
9	Neutrophils	*LOC138844609*	0.4523	0.261	0.103	8.092e-09	1.869e-04
9	Neutrophils	*NDUFB1*	0.4512	0.478	0.210	9.265e-12	2.140e-07
9	Neutrophils	*LRCH4*	0.4505	0.224	0.034	2.961e-30	6.837e-26
9	Neutrophils	*TFEB*	0.4500	0.134	0.038	1.420e-08	3.280e-04
9	Neutrophils	*HIVEP1*	0.4498	0.291	0.095	5.393e-13	1.245e-08
9	Neutrophils	*SMG7*	0.4490	0.291	0.094	3.664e-14	8.461e-10
9	Neutrophils	*LMBRD2*	0.4487	0.284	0.118	1.864e-08	4.304e-04
9	Neutrophils	*NBEAL2*	0.4476	0.187	0.016	7.069e-48	1.632e-43
9	Neutrophils	*RNF10*	0.4468	0.507	0.305	1.724e-06	3.981e-02
9	Neutrophils	*LPAR6*	0.4462	0.127	0.043	2.024e-06	4.673e-02
9	Neutrophils	*VPS39*	0.4461	0.291	0.092	1.042e-13	2.406e-09
9	Neutrophils	*PHF8*	0.4450	0.321	0.147	6.392e-08	1.476e-03
9	Neutrophils	*WSCD1*	0.4444	0.194	0.006	3.336e-119	7.704e-115
9	Neutrophils	*IGF2BP3*	0.4439	0.157	0.007	8.278e-71	1.912e-66
9	Neutrophils	*R3HCC1L*	0.4438	0.351	0.118	6.262e-15	1.446e-10
9	Neutrophils	*ARHGEF2*	0.4426	0.299	0.129	1.028e-07	2.375e-03
9	Neutrophils	*RNF216*	0.4421	0.336	0.151	6.187e-08	1.429e-03
9	Neutrophils	*NRP1*	0.4418	0.336	0.138	1.520e-09	3.510e-05
9	Neutrophils	*TAF10*	0.4416	0.433	0.202	2.194e-09	5.066e-05
9	Neutrophils	*LRRFIP1*	0.4413	0.448	0.202	1.724e-10	3.982e-06
9	Neutrophils	*BLTP2*	0.4403	0.336	0.112	1.207e-14	2.787e-10
9	Neutrophils	*TMED7*	0.4401	0.493	0.217	2.869e-11	6.624e-07
9	Neutrophils	*SGMS2*	0.4384	0.209	0.045	7.233e-19	1.670e-14
9	Neutrophils	*LOC138850498*	0.4383	0.209	0.005	3.972e-161	9.172e-157
9	Neutrophils	*RFX1*	0.4380	0.224	0.092	5.027e-07	1.161e-02
9	Neutrophils	*MYO1G*	0.4377	0.194	0.007	3.006e-107	6.941e-103
9	Neutrophils	*IKBKE*	0.4368	0.157	0.019	8.107e-28	1.872e-23
9	Neutrophils	*PRKD3*	0.4368	0.493	0.245	3.030e-09	6.996e-05
9	Neutrophils	*CAPG*	0.4367	0.209	0.033	5.371e-27	1.240e-22
9	Neutrophils	*LOC103346273*	0.4357	0.172	0.000	1.432e-302	3.307e-298
9	Neutrophils	*RAB21*	0.4354	0.358	0.177	7.666e-07	1.770e-02
9	Neutrophils	*FOXK2*	0.4350	0.216	0.044	3.307e-20	7.635e-16

Original cluster	Annotated cell type	Gene	avg_log2FC	pct.1	pct.2	Raw P value	Bonferroni-adjusted P value
9	Neutrophils	*GSDME*	0.4343	0.119	0.022	9.785e-14	2.260e-09
9	Neutrophils	*DNASE2*	0.4342	0.246	0.090	3.629e-09	8.381e-05
9	Neutrophils	*PTPN22*	0.4342	0.164	0.013	2.252e-45	5.199e-41
9	Neutrophils	*GALNT3*	0.4340	0.142	0.013	2.868e-33	6.622e-29
9	Neutrophils	*R3HDM4*	0.4337	0.299	0.074	8.452e-21	1.952e-16
9	Neutrophils	*CDC42EP3*	0.4336	0.179	0.042	1.524e-14	3.520e-10
9	Neutrophils	*TES*	0.4334	0.246	0.090	8.118e-10	1.875e-05
9	Neutrophils	*ETV3*	0.4332	0.276	0.127	1.878e-06	4.336e-02
9	Neutrophils	*ATG7*	0.4331	0.231	0.053	3.133e-18	7.235e-14
9	Neutrophils	*TMEM131*	0.4324	0.388	0.185	8.920e-08	2.060e-03
9	Neutrophils	*THEMIS2*	0.4300	0.224	0.008	8.224e-122	1.899e-117
9	Neutrophils	*GAPVD1*	0.4300	0.313	0.142	1.800e-07	4.156e-03
9	Neutrophils	*TET3*	0.4299	0.201	0.047	4.324e-16	9.986e-12
9	Neutrophils	*SPG11*	0.4295	0.231	0.079	6.578e-10	1.519e-05
9	Neutrophils	*XIAP*	0.4292	0.299	0.126	1.866e-08	4.308e-04
9	Neutrophils	*LOC100347048*	0.4291	0.299	0.086	7.090e-17	1.637e-12
9	Neutrophils	*ZNF710*	0.4289	0.269	0.055	1.100e-24	2.540e-20
9	Neutrophils	*SQOR*	0.4288	0.313	0.132	6.808e-09	1.572e-04
9	Neutrophils	*LOC100340393*	0.4284	0.276	0.047	9.580e-32	2.212e-27
9	Neutrophils	*ZBTB7B*	0.4277	0.164	0.019	1.071e-30	2.474e-26
9	Neutrophils	*SLK*	0.4271	0.515	0.286	3.845e-07	8.878e-03
9	Neutrophils	*MYCBP*	0.4264	0.239	0.022	4.320e-55	9.975e-51
9	Neutrophils	*TRAPPC1*	0.4255	0.366	0.134	2.909e-13	6.718e-09
9	Neutrophils	*GRIPAP1*	0.4249	0.291	0.078	4.330e-18	9.999e-14
9	Neutrophils	*TACC3*	0.4247	0.179	0.011	1.558e-65	3.597e-61
9	Neutrophils	*PTPN11*	0.4246	0.246	0.092	6.084e-09	1.405e-04
9	Neutrophils	*ABTB2*	0.4240	0.142	0.030	3.242e-13	7.487e-09
9	Neutrophils	*ZNFX1*	0.4232	0.231	0.085	1.571e-08	3.629e-04
9	Neutrophils	*CHMP3*	0.4231	0.619	0.318	1.255e-09	2.898e-05
9	Neutrophils	*PCMT1*	0.4230	0.463	0.236	2.068e-08	4.775e-04
9	Neutrophils	*BAX*	0.4230	0.351	0.114	3.045e-15	7.032e-11
9	Neutrophils	*RPS6KA2*	0.4225	0.209	0.054	2.106e-14	4.863e-10
9	Neutrophils	*DCK*	0.4224	0.254	0.053	7.695e-23	1.777e-18
9	Neutrophils	*SPN*	0.4223	0.164	0.010	1.580e-58	3.648e-54
9	Neutrophils	*DAGLB*	0.4221	0.157	0.026	1.051e-19	2.428e-15
9	Neutrophils	*STX7*	0.4221	0.448	0.170	1.184e-14	2.734e-10
9	Neutrophils	*ANKRD13A*	0.4221	0.261	0.047	2.310e-28	5.334e-24
9	Neutrophils	*SEC23B*	0.4215	0.313	0.079	4.363e-21	1.007e-16
9	Neutrophils	*PDLIM2*	0.4213	0.291	0.078	2.629e-18	6.070e-14
9	Neutrophils	*TGFBR1*	0.4213	0.448	0.191	2.154e-11	4.974e-07

Original cluster	Annotated cell type	Gene	avg_log2FC	pct.1	pct.2	Raw P value	Bonferroni-adjusted P value
9	Neutrophils	*LOC100343767*	0.4207	0.179	0.008	1.443e-82	3.331e-78
9	Neutrophils	*HIP1*	0.4192	0.343	0.111	3.555e-15	8.208e-11
9	Neutrophils	*BLTP3B*	0.4188	0.358	0.174	3.033e-07	7.004e-03
9	Neutrophils	*SNX3*	0.4185	0.246	0.073	1.902e-13	4.393e-09
9	Neutrophils	*UPP1*	0.4184	0.142	0.016	3.542e-27	8.180e-23
9	Neutrophils	*GAS2L3*	0.4174	0.224	0.049	5.193e-19	1.199e-14
9	Neutrophils	*KIF21B*	0.4170	0.119	0.009	1.934e-34	4.467e-30
9	Neutrophils	*LOC138849409*	0.4164	0.209	0.084	4.802e-07	1.109e-02
9	Neutrophils	*TAX1BP3*	0.4159	0.299	0.073	3.699e-21	8.542e-17
9	Neutrophils	*PLP2*	0.4158	0.313	0.129	8.419e-10	1.944e-05
9	Neutrophils	*RAB5A*	0.4157	0.366	0.174	3.252e-07	7.510e-03
9	Neutrophils	*LOC138849238*	0.4153	0.187	0.009	4.110e-82	9.492e-78
9	Neutrophils	*NCOA3*	0.4149	0.291	0.122	2.451e-08	5.659e-04
9	Neutrophils	*STK26*	0.4144	0.187	0.061	4.592e-09	1.060e-04
9	Neutrophils	*GPR132*	0.4144	0.134	0.001	6.818e-154	1.574e-149
9	Neutrophils	*PSMB10*	0.4141	0.328	0.094	7.268e-18	1.678e-13
9	Neutrophils	*IDH1*	0.4134	0.254	0.082	3.824e-12	8.830e-08
9	Neutrophils	*NHP2*	0.4134	0.299	0.087	5.150e-16	1.189e-11
9	Neutrophils	*RNASEK*	0.4134	0.575	0.349	1.209e-06	2.791e-02
9	Neutrophils	*CTCF*	0.4126	0.403	0.215	5.037e-07	1.163e-02
9	Neutrophils	*KLHL5*	0.4119	0.164	0.031	1.834e-17	4.235e-13
9	Neutrophils	*PRKCD*	0.4111	0.216	0.024	3.193e-41	7.373e-37
9	Neutrophils	*PPP2R5C*	0.4095	0.366	0.164	2.655e-08	6.130e-04
9	Neutrophils	*ARHGDIA*	0.4085	0.590	0.371	1.262e-06	2.914e-02
9	Neutrophils	*DGKZ*	0.4078	0.224	0.026	1.462e-41	3.376e-37
9	Neutrophils	*UBE2K*	0.4073	0.366	0.165	8.021e-09	1.852e-04
9	Neutrophils	*RASSF4*	0.4070	0.224	0.012	3.856e-86	8.905e-82
9	Neutrophils	*TESC*	0.4051	0.149	0.026	2.772e-18	6.400e-14
9	Neutrophils	*NCF4*	0.4038	0.179	0.002	9.324e-212	2.153e-207
9	Neutrophils	*SNAP29*	0.4036	0.284	0.105	5.697e-10	1.316e-05
9	Neutrophils	*LOC100355380*	0.4033	0.172	0.011	2.534e-59	5.852e-55
9	Neutrophils	*RBM38*	0.4029	0.157	0.015	6.358e-36	1.468e-31
9	Neutrophils	*DOP1B*	0.4026	0.149	0.026	1.632e-17	3.768e-13
9	Neutrophils	*ZDHHC5*	0.4022	0.254	0.076	1.591e-13	3.673e-09
9	Neutrophils	*TP53INP2*	0.4019	0.194	0.024	2.937e-34	6.782e-30
9	Neutrophils	*ABR*	0.4018	0.299	0.076	2.431e-20	5.613e-16
9	Neutrophils	*TWF2*	0.4014	0.336	0.108	2.505e-15	5.785e-11
9	Neutrophils	*KIF13B*	0.4013	0.209	0.085	8.096e-07	1.869e-02
9	Neutrophils	*HSPBAP1*	0.4013	0.209	0.056	2.753e-13	6.358e-09
9	Neutrophils	*SLC15A3*	0.4012	0.299	0.061	7.904e-27	1.825e-22

Original cluster	Annotated cell type	Gene	avg_log2FC	pct.1	pct.2	Raw P value	Bonferroni-adjusted P value
9	Neutrophils	*COX17*	0.4011	0.478	0.247	6.345e-08	1.465e-03
9	Neutrophils	*SESN2*	0.4007	0.104	0.016	2.806e-15	6.480e-11
9	Neutrophils	*FEZ2*	0.4004	0.321	0.128	5.989e-10	1.383e-05
9	Neutrophils	*GSR*	0.3997	0.224	0.067	1.886e-12	4.356e-08
9	Neutrophils	*LOC108175363*	0.3984	0.187	0.012	8.048e-63	1.858e-58
9	Neutrophils	*AP2B1*	0.3978	0.478	0.227	6.511e-10	1.503e-05
9	Neutrophils	*CCR5*	0.3977	0.187	0.007	4.346e-96	1.003e-91
9	Neutrophils	*DPP8*	0.3973	0.231	0.092	7.441e-08	1.718e-03
9	Neutrophils	*GGTA1*	0.3972	0.418	0.183	6.031e-10	1.393e-05
9	Neutrophils	*GPD2*	0.3963	0.216	0.071	3.989e-10	9.211e-06
9	Neutrophils	*MERTK*	0.3958	0.201	0.055	1.054e-12	2.434e-08
9	Neutrophils	*NAPSA*	0.3954	0.201	0.005	5.748e-143	1.327e-138
9	Neutrophils	*PLEKHO2*	0.3947	0.179	0.048	6.723e-12	1.552e-07
9	Neutrophils	*CD163*	0.3937	0.149	0.000	1.768e-298	4.083e-294
9	Neutrophils	*SLC25A13*	0.3930	0.261	0.058	1.065e-21	2.460e-17
9	Neutrophils	*GPR107*	0.3919	0.149	0.023	6.438e-20	1.487e-15
9	Neutrophils	*EYA1*	0.3906	0.142	0.043	5.063e-08	1.169e-03
9	Neutrophils	*ST6GALNAC4*	0.3905	0.209	0.053	7.347e-15	1.696e-10
9	Neutrophils	*ATP6V1F*	0.3902	0.366	0.147	1.036e-10	2.392e-06
9	Neutrophils	*BZW2*	0.3887	0.328	0.154	2.165e-07	4.999e-03
9	Neutrophils	*PITPNA*	0.3877	0.336	0.143	9.936e-09	2.294e-04
9	Neutrophils	*GFOD1*	0.3875	0.172	0.030	2.469e-20	5.700e-16
9	Neutrophils	*GNL2*	0.3874	0.284	0.101	1.579e-10	3.645e-06
9	Neutrophils	*LOC138843758*	0.3861	0.119	0.001	1.950e-151	4.503e-147
9	Neutrophils	*CDYL*	0.3860	0.284	0.091	3.510e-13	8.105e-09
9	Neutrophils	*IFNAR2*	0.3859	0.313	0.097	2.158e-15	4.983e-11
9	Neutrophils	*TMEM9B*	0.3849	0.276	0.075	1.737e-16	4.012e-12
9	Neutrophils	*KLF3*	0.3841	0.351	0.172	1.108e-06	2.558e-02
9	Neutrophils	*E2F2*	0.3841	0.127	0.001	1.776e-186	4.102e-182
9	Neutrophils	*DNAJB14*	0.3831	0.306	0.123	1.494e-09	3.451e-05
9	Neutrophils	*PAG1*	0.3829	0.134	0.025	1.021e-14	2.358e-10
9	Neutrophils	*SYTL1*	0.3822	0.134	0.008	2.780e-50	6.418e-46
9	Neutrophils	*RAPGEF1*	0.3818	0.194	0.052	1.672e-12	3.860e-08
9	Neutrophils	*ATG16L2*	0.3815	0.179	0.035	5.500e-18	1.270e-13
9	Neutrophils	*PDCD10*	0.3814	0.448	0.227	2.612e-07	6.031e-03
9	Neutrophils	*GGH*	0.3813	0.164	0.008	3.467e-68	8.005e-64
9	Neutrophils	*MEF2A*	0.3794	0.567	0.337	7.746e-07	1.789e-02
9	Neutrophils	*SLC35A5*	0.3792	0.343	0.120	3.385e-13	7.816e-09
9	Neutrophils	*LOC100352672*	0.3780	0.194	0.006	3.998e-123	9.233e-119
9	Neutrophils	*CCDC12*	0.3776	0.336	0.112	4.168e-14	9.624e-10

Original cluster	Annotated cell type	Gene	avg_log2FC	pct.1	pct.2	Raw P value	Bonferroni-adjusted P value
9	Neutrophils	*TPP1*	0.3770	0.328	0.096	1.816e-17	4.193e-13
9	Neutrophils	*SFT2D1*	0.3769	0.358	0.166	2.501e-08	5.775e-04
9	Neutrophils	*FKBP15*	0.3769	0.216	0.049	3.183e-17	7.350e-13
9	Neutrophils	*TIFAB*	0.3767	0.164	0.000	0	0
9	Neutrophils	*MBOAT7*	0.3757	0.254	0.096	4.137e-09	9.553e-05
9	Neutrophils	*PARP14*	0.3757	0.246	0.074	2.330e-13	5.379e-09
9	Neutrophils	*DENND3*	0.3741	0.142	0.007	7.589e-61	1.752e-56
9	Neutrophils	*IDH2*	0.3739	0.328	0.159	7.904e-07	1.825e-02
9	Neutrophils	*RABGEF1*	0.3739	0.306	0.120	7.041e-10	1.626e-05
9	Neutrophils	*DBNL*	0.3735	0.336	0.140	1.493e-09	3.448e-05
9	Neutrophils	*FGL2*	0.3730	0.410	0.174	4.317e-11	9.968e-07
9	Neutrophils	*QPCT*	0.3727	0.164	0.024	1.889e-24	4.362e-20
9	Neutrophils	*TENT2*	0.3723	0.463	0.210	2.375e-10	5.485e-06
9	Neutrophils	*HIGD1A*	0.3723	0.209	0.046	3.086e-18	7.126e-14
9	Neutrophils	*MORN1*	0.3717	0.209	0.018	6.124e-53	1.414e-48
9	Neutrophils	*MAX*	0.3693	0.425	0.207	1.157e-07	2.671e-03
9	Neutrophils	*PARN*	0.3686	0.306	0.121	1.108e-09	2.558e-05
9	Neutrophils	*AP1B1*	0.3684	0.224	0.036	6.352e-28	1.467e-23
9	Neutrophils	*LOC108176496*	0.3672	0.187	0.070	7.696e-07	1.777e-02
9	Neutrophils	*TRAF3IP3*	0.3664	0.172	0.028	3.450e-22	7.966e-18
9	Neutrophils	*INPP5B*	0.3658	0.216	0.060	4.168e-13	9.624e-09
9	Neutrophils	*IPPK*	0.3652	0.157	0.033	2.380e-14	5.495e-10
9	Neutrophils	*CFH*	0.3637	0.142	0.001	4.140e-166	9.561e-162
9	Neutrophils	*RHOQ*	0.3633	0.306	0.129	1.173e-08	2.710e-04
9	Neutrophils	*CORO1C*	0.3632	0.291	0.077	2.306e-18	5.325e-14
9	Neutrophils	*SSR1*	0.3631	0.299	0.094	3.445e-14	7.955e-10
9	Neutrophils	*PTPN9*	0.3619	0.306	0.134	1.091e-07	2.520e-03
9	Neutrophils	*TMED3*	0.3608	0.448	0.237	1.653e-06	3.816e-02
9	Neutrophils	*LOC100357099*	0.3605	0.149	0.046	2.835e-08	6.547e-04
9	Neutrophils	*FAM78A*	0.3598	0.209	0.015	2.732e-64	6.309e-60
9	Neutrophils	*RNF145*	0.3584	0.216	0.088	6.581e-07	1.520e-02
9	Neutrophils	*BATF3*	0.3577	0.157	0.002	1.739e-185	4.015e-181
9	Neutrophils	*LOC138844733*	0.3573	0.104	0.020	1.182e-11	2.730e-07
9	Neutrophils	*ZHX2*	0.3558	0.246	0.104	5.435e-07	1.255e-02
9	Neutrophils	*SGK3*	0.3550	0.313	0.130	8.859e-09	2.046e-04
9	Neutrophils	*SLC35A3*	0.3550	0.239	0.078	8.124e-11	1.876e-06
9	Neutrophils	*CMBL*	0.3544	0.134	0.021	6.025e-18	1.391e-13
9	Neutrophils	*SHKBP1*	0.3540	0.187	0.020	3.773e-38	8.714e-34
9	Neutrophils	*LOC138849562*	0.3539	0.216	0.082	8.292e-08	1.915e-03
9	Neutrophils	*MICAL2*	0.3530	0.157	0.019	1.340e-27	3.095e-23

Original cluster	Annotated cell type	Gene	avg_log2FC	pct.1	pct.2	Raw P value	Bonferroni-adjusted P value
9	Neutrophils	*EEPD1*	0.3522	0.149	0.021	6.445e-23	1.488e-18
9	Neutrophils	*PRODH*	0.3514	0.164	0.021	5.558e-28	1.283e-23
9	Neutrophils	*ADGRE5*	0.3511	0.209	0.027	4.731e-34	1.093e-29
9	Neutrophils	*RNPEPL1*	0.3510	0.179	0.048	1.710e-11	3.948e-07
9	Neutrophils	*CORO7*	0.3498	0.104	0.009	3.487e-28	8.051e-24
9	Neutrophils	*TLE3*	0.3491	0.172	0.043	2.755e-12	6.362e-08
9	Neutrophils	*BCL3*	0.3490	0.164	0.040	2.077e-12	4.797e-08
9	Neutrophils	*SH2B3*	0.3489	0.254	0.033	1.708e-40	3.944e-36
9	Neutrophils	*PTPRCAP*	0.3488	0.104	0.027	6.924e-08	1.599e-03
9	Neutrophils	*ARHGAP9*	0.3477	0.142	0.026	1.609e-15	3.716e-11
9	Neutrophils	*ARID3A*	0.3475	0.179	0.068	9.312e-07	2.150e-02
9	Neutrophils	*RNF44*	0.3472	0.224	0.078	1.380e-09	3.188e-05
9	Neutrophils	*ZNF217*	0.3471	0.261	0.115	6.902e-07	1.594e-02
9	Neutrophils	*NATD1*	0.3470	0.194	0.046	4.607e-15	1.064e-10
9	Neutrophils	*EDEM1*	0.3468	0.254	0.044	6.301e-29	1.455e-24
9	Neutrophils	*PSMD13*	0.3465	0.261	0.117	1.948e-06	4.497e-02
9	Neutrophils	*IL16*	0.3451	0.201	0.020	4.610e-43	1.065e-38
9	Neutrophils	*JOSD1*	0.3449	0.276	0.066	2.813e-20	6.496e-16
9	Neutrophils	*SLC26A2*	0.3446	0.194	0.053	3.005e-12	6.940e-08
9	Neutrophils	*PARVG*	0.3446	0.209	0.006	1.343e-130	3.101e-126
9	Neutrophils	*SLC45A4*	0.3446	0.127	0.015	4.777e-23	1.103e-18
9	Neutrophils	*SASH3*	0.3427	0.194	0.009	3.599e-90	8.312e-86
9	Neutrophils	*TXNDC9*	0.3424	0.284	0.073	5.132e-18	1.185e-13
9	Neutrophils	*TTYH3*	0.3424	0.269	0.073	3.101e-16	7.160e-12
9	Neutrophils	*MOB3C*	0.3423	0.201	0.028	2.501e-30	5.776e-26
9	Neutrophils	*ARSB*	0.3423	0.187	0.039	6.207e-17	1.433e-12
9	Neutrophils	*FBH1*	0.3416	0.142	0.038	1.438e-09	3.321e-05
9	Neutrophils	*SLC43A2*	0.3414	0.172	0.027	3.417e-23	7.890e-19
9	Neutrophils	*IRF4*	0.3410	0.127	0.003	8.555e-100	1.976e-95
9	Neutrophils	*KYNU*	0.3404	0.149	0.003	2.750e-123	6.350e-119
9	Neutrophils	*CACUL1*	0.3401	0.291	0.126	8.407e-08	1.941e-03
9	Neutrophils	*PHACTR1*	0.3398	0.313	0.092	3.460e-16	7.990e-12
9	Neutrophils	*PRR14L*	0.3397	0.284	0.117	4.390e-08	1.014e-03
9	Neutrophils	*MAP3K1*	0.3397	0.351	0.158	7.433e-08	1.716e-03
9	Neutrophils	*LOC103349875*	0.3397	0.142	0.006	1.049e-66	2.422e-62
9	Neutrophils	*LYL1*	0.3395	0.194	0.043	1.948e-16	4.498e-12
9	Neutrophils	*NUP210*	0.3388	0.216	0.024	1.692e-41	3.908e-37
9	Neutrophils	*LOC100344156*	0.3382	0.134	0.018	2.445e-22	5.646e-18
9	Neutrophils	*SLC37A2*	0.3373	0.142	0.002	3.817e-135	8.814e-131
9	Neutrophils	*PHYKPL*	0.3364	0.269	0.110	6.379e-08	1.473e-03

Original cluster	Annotated cell type	Gene	avg_log2FC	pct.1	pct.2	Raw P value	Bonferroni-adjusted P value
9	Neutrophils	*GAA*	0.3355	0.142	0.047	7.414e-07	1.712e-02
9	Neutrophils	*ARIH2*	0.3349	0.328	0.145	1.007e-07	2.324e-03
9	Neutrophils	*PSD4*	0.3339	0.134	0.026	7.954e-14	1.837e-09
9	Neutrophils	*TRAPPC14*	0.3336	0.142	0.002	1.643e-128	3.795e-124
9	Neutrophils	*LOC103345202*	0.3331	0.149	0.027	2.345e-16	5.414e-12
9	Neutrophils	*MARCHF2*	0.3328	0.157	0.024	2.653e-21	6.126e-17
9	Neutrophils	*PRDM10*	0.3316	0.164	0.056	1.434e-07	3.311e-03
9	Neutrophils	*IGF2R*	0.3314	0.149	0.032	1.573e-13	3.632e-09
9	Neutrophils	*DTX2*	0.3308	0.179	0.033	3.390e-19	7.828e-15
9	Neutrophils	*PPM1G*	0.3307	0.351	0.145	1.328e-09	3.067e-05
9	Neutrophils	*FAM118B*	0.3305	0.179	0.027	1.407e-24	3.248e-20
9	Neutrophils	*MAFG*	0.3297	0.254	0.070	7.027e-15	1.623e-10
9	Neutrophils	*PRKAA1*	0.3288	0.231	0.084	4.141e-09	9.562e-05
9	Neutrophils	*GSK3A*	0.3280	0.284	0.125	2.703e-07	6.242e-03
9	Neutrophils	*LOC138848553*	0.3277	0.216	0.044	1.810e-20	4.180e-16
9	Neutrophils	*BCL2L11*	0.3266	0.209	0.080	1.396e-07	3.225e-03
9	Neutrophils	*LOC138844436*	0.3262	0.149	0.016	9.549e-32	2.205e-27
9	Neutrophils	*LOC127493011*	0.3260	0.172	0.026	4.675e-23	1.080e-18
9	Neutrophils	*AP2A1*	0.3255	0.276	0.078	1.998e-15	4.614e-11
9	Neutrophils	*ARID1A*	0.3253	0.448	0.216	2.509e-08	5.794e-04
9	Neutrophils	*BRWD1*	0.3248	0.448	0.230	1.948e-06	4.498e-02
9	Neutrophils	*FLOT1*	0.3224	0.276	0.124	5.968e-07	1.378e-02
9	Neutrophils	*ST3GAL2*	0.3223	0.276	0.103	3.166e-10	7.311e-06
9	Neutrophils	*ZC3H12A*	0.3204	0.142	0.017	1.493e-26	3.447e-22
9	Neutrophils	*RALGAPB*	0.3196	0.209	0.069	9.517e-10	2.198e-05
9	Neutrophils	*PGP*	0.3194	0.127	0.025	9.396e-13	2.170e-08
9	Neutrophils	*USP36*	0.3177	0.209	0.045	3.672e-18	8.479e-14
9	Neutrophils	*RIPK1*	0.3167	0.209	0.059	3.853e-12	8.898e-08
9	Neutrophils	*DNAJC5*	0.3158	0.179	0.036	1.606e-17	3.708e-13
9	Neutrophils	*MANBA*	0.3156	0.149	0.023	6.531e-21	1.508e-16
9	Neutrophils	*PARD6A*	0.3156	0.127	0.034	8.164e-09	1.885e-04
9	Neutrophils	*LOC138844757*	0.3156	0.157	0.039	1.133e-11	2.617e-07
9	Neutrophils	*EMILIN2*	0.3154	0.216	0.090	1.596e-06	3.685e-02
9	Neutrophils	*ZMAT2*	0.3153	0.336	0.127	6.718e-11	1.551e-06
9	Neutrophils	*USP6NL*	0.3153	0.209	0.070	3.303e-09	7.627e-05
9	Neutrophils	*TRABD2A*	0.3151	0.209	0.028	5.823e-33	1.345e-28
9	Neutrophils	*MRPL9*	0.3151	0.313	0.136	5.692e-08	1.314e-03
9	Neutrophils	*SMIM27*	0.3149	0.239	0.078	9.275e-11	2.142e-06
9	Neutrophils	*LOC100349247*	0.3146	0.172	0.012	7.800e-54	1.801e-49
9	Neutrophils	*CCDC124*	0.3141	0.261	0.099	3.514e-09	8.115e-05

Original cluster	Annotated cell type	Gene	avg_log2FC	pct.1	pct.2	Raw P value	Bonferroni-adjusted P value
9	Neutrophils	*ACAP1*	0.3141	0.119	0.010	1.556e-30	3.594e-26
9	Neutrophils	*TMEM273*	0.3123	0.157	0.017	3.346e-31	7.728e-27
9	Neutrophils	*PLAGL2*	0.3120	0.216	0.065	1.759e-11	4.061e-07
9	Neutrophils	*SPCS3*	0.3104	0.507	0.269	8.011e-07	1.850e-02
9	Neutrophils	*STARD4*	0.3088	0.164	0.004	2.632e-127	6.077e-123
9	Neutrophils	*MASTL*	0.3081	0.142	0.045	1.742e-07	4.024e-03
9	Neutrophils	*NXT1*	0.3069	0.231	0.072	1.924e-11	4.443e-07
9	Neutrophils	*GMEB1*	0.3069	0.187	0.064	3.407e-08	7.868e-04
9	Neutrophils	*RNF144B*	0.3054	0.246	0.031	1.074e-41	2.480e-37
9	Neutrophils	*CBFB*	0.3054	0.276	0.115	1.593e-07	3.680e-03
9	Neutrophils	*LOC138850176*	0.3050	0.187	0.025	5.217e-29	1.205e-24
9	Neutrophils	*EPG5*	0.3047	0.284	0.123	3.675e-07	8.487e-03
9	Neutrophils	*TICAM2*	0.3044	0.134	0.003	6.929e-115	1.600e-110
9	Neutrophils	*ATG101*	0.3039	0.396	0.159	5.098e-11	1.177e-06
9	Neutrophils	*SVIP*	0.3035	0.172	0.041	4.581e-13	1.058e-08
9	Neutrophils	*BLVRA*	0.3028	0.201	0.027	7.996e-32	1.846e-27
9	Neutrophils	*WIPF2*	0.3024	0.254	0.099	4.156e-08	9.597e-04
9	Neutrophils	*SHISA5*	0.3019	0.366	0.174	3.031e-07	7.000e-03
9	Neutrophils	*LOC100341005*	0.3019	0.164	0.025	2.058e-22	4.752e-18
9	Neutrophils	*RASAL3*	0.3015	0.134	0.011	8.809e-37	2.034e-32
9	Neutrophils	*ARRB1*	0.3003	0.254	0.065	1.357e-16	3.133e-12
9	Neutrophils	*MON1B*	0.3000	0.216	0.052	4.157e-16	9.600e-12
9	Neutrophils	*ACLY*	0.2980	0.209	0.081	3.091e-07	7.137e-03
9	Neutrophils	*CRYBG1*	0.2968	0.246	0.084	3.561e-10	8.224e-06
9	Neutrophils	*HRH2*	0.2965	0.104	0.001	4.517e-132	1.043e-127
9	Neutrophils	*HSD17B11*	0.2960	0.201	0.029	6.888e-29	1.591e-24
9	Neutrophils	*TLR6*	0.2958	0.142	0.034	4.093e-11	9.452e-07
9	Neutrophils	*ZZEF1*	0.2957	0.239	0.101	5.095e-07	1.177e-02
9	Neutrophils	*KCNE3*	0.2957	0.134	0.002	9.422e-125	2.176e-120
9	Neutrophils	*NDUFS8*	0.2956	0.209	0.036	3.057e-24	7.059e-20
9	Neutrophils	*UBTD1*	0.2949	0.134	0.023	3.028e-16	6.993e-12
9	Neutrophils	*ERAP1*	0.2939	0.187	0.033	3.418e-21	7.893e-17
9	Neutrophils	*DCAF11*	0.2937	0.164	0.044	1.158e-10	2.674e-06
9	Neutrophils	*MORC3*	0.2936	0.403	0.188	2.055e-08	4.746e-04
9	Neutrophils	*STAMBPL1*	0.2923	0.149	0.025	6.776e-19	1.565e-14
9	Neutrophils	*UNC13B*	0.2915	0.149	0.052	1.374e-06	3.173e-02
9	Neutrophils	*TRIM5*	0.2911	0.127	0.035	2.247e-08	5.190e-04
9	Neutrophils	*CYP4V2*	0.2897	0.179	0.036	1.884e-17	4.351e-13
9	Neutrophils	*P2RY12*	0.2896	0.104	0.002	8.753e-103	2.021e-98
9	Neutrophils	*SCYL3*	0.2895	0.164	0.040	2.733e-12	6.312e-08

Original cluster	Annotated cell type	Gene	avg_log2FC	pct.1	pct.2	Raw P value	Bonferroni-adjusted P value
9	Neutrophils	*ST3GAL5*	0.2894	0.127	0.022	6.332e-15	1.462e-10
9	Neutrophils	*MAPKAPK2*	0.2890	0.269	0.081	1.395e-13	3.221e-09
9	Neutrophils	*SLC36A1*	0.2889	0.104	0.008	6.886e-29	1.590e-24
9	Neutrophils	*CLN5*	0.2882	0.299	0.110	2.088e-10	4.821e-06
9	Neutrophils	*NLRP2*	0.2881	0.142	0.003	1.582e-113	3.653e-109
9	Neutrophils	*B4GALT5*	0.2865	0.142	0.040	8.972e-09	2.072e-04
9	Neutrophils	*IQGAP2*	0.2862	0.179	0.020	2.361e-35	5.452e-31
9	Neutrophils	*LPGAT1*	0.2861	0.269	0.115	2.810e-07	6.489e-03
9	Neutrophils	*NUP50*	0.2859	0.291	0.101	1.358e-11	3.135e-07
9	Neutrophils	*LOC100350928*	0.2849	0.216	0.087	9.624e-07	2.222e-02
9	Neutrophils	*ARAP1*	0.2834	0.224	0.046	2.213e-20	5.111e-16
9	Neutrophils	*UBE2F*	0.2834	0.313	0.136	9.606e-08	2.218e-03
9	Neutrophils	*PTGER4*	0.2829	0.172	0.046	4.091e-11	9.448e-07
9	Neutrophils	*RAB39A*	0.2828	0.157	0.002	4.285e-161	9.895e-157
9	Neutrophils	*PPP1R3D*	0.2824	0.127	0.014	8.631e-26	1.993e-21
9	Neutrophils	*PLEKHM2*	0.2809	0.179	0.065	4.421e-07	1.021e-02
9	Neutrophils	*SUDS3*	0.2800	0.373	0.164	1.221e-08	2.819e-04
9	Neutrophils	*RGS14*	0.2796	0.142	0.023	3.498e-18	8.077e-14
9	Neutrophils	*TTLL5*	0.2789	0.201	0.076	5.148e-07	1.189e-02
9	Neutrophils	*NT5C*	0.2772	0.336	0.136	2.712e-09	6.262e-05
9	Neutrophils	*RHOF*	0.2760	0.112	0.012	1.337e-22	3.087e-18
9	Neutrophils	*RELL1*	0.2759	0.239	0.084	1.400e-09	3.234e-05
9	Neutrophils	*CCM2*	0.2756	0.209	0.066	3.939e-10	9.095e-06
9	Neutrophils	*YWHAG*	0.2744	0.418	0.215	1.285e-06	2.968e-02
9	Neutrophils	*RAP2B*	0.2743	0.313	0.145	9.848e-07	2.274e-02
9	Neutrophils	*DHRS11*	0.2738	0.209	0.064	6.092e-11	1.407e-06
9	Neutrophils	*UBE2J1*	0.2735	0.299	0.119	6.718e-09	1.551e-04
9	Neutrophils	*MXD4*	0.2734	0.231	0.063	4.700e-14	1.085e-09
9	Neutrophils	*LSM1*	0.2728	0.343	0.114	8.767e-14	2.025e-09
9	Neutrophils	*KATNIP*	0.2722	0.149	0.042	4.457e-09	1.029e-04
9	Neutrophils	*BLNK*	0.2719	0.134	0.009	4.357e-46	1.006e-41
9	Neutrophils	*ZNF787*	0.2718	0.209	0.071	6.790e-09	1.568e-04
9	Neutrophils	*RNF167*	0.2713	0.463	0.235	2.690e-07	6.212e-03
9	Neutrophils	*ADCY7*	0.2711	0.164	0.006	7.743e-84	1.788e-79
9	Neutrophils	*MAP2K1*	0.2701	0.104	0.022	4.242e-10	9.795e-06
9	Neutrophils	*WDR77*	0.2699	0.209	0.059	2.093e-12	4.833e-08
9	Neutrophils	*CNOT10*	0.2698	0.239	0.088	5.808e-09	1.341e-04
9	Neutrophils	*DERL1*	0.2692	0.336	0.165	1.486e-06	3.432e-02
9	Neutrophils	*NRROS*	0.2661	0.187	0.008	2.503e-86	5.781e-82
9	Neutrophils	*STAT1*	0.2659	0.321	0.142	8.440e-08	1.949e-03

Original cluster	Annotated cell type	Gene	avg_log2FC	pct.1	pct.2	Raw P value	Bonferroni-adjusted P value
9	Neutrophils	*OTULINL*	0.2656	0.291	0.110	2.255e-09	5.208e-05
9	Neutrophils	*SNX30*	0.2648	0.239	0.071	1.619e-12	3.740e-08
9	Neutrophils	*UNC13D*	0.2642	0.127	0.006	6.614e-57	1.527e-52
9	Neutrophils	*MAP4K2*	0.2638	0.149	0.019	5.880e-25	1.358e-20
9	Neutrophils	*APBA1*	0.2637	0.142	0.049	2.020e-06	4.664e-02
9	Neutrophils	*EXOC8*	0.2631	0.194	0.066	2.361e-08	5.452e-04
9	Neutrophils	*ARSG*	0.2626	0.134	0.031	2.924e-11	6.753e-07
9	Neutrophils	*RNF123*	0.2607	0.112	0.024	1.644e-10	3.795e-06
9	Neutrophils	*ZBTB2*	0.2602	0.164	0.060	1.521e-06	3.513e-02
9	Neutrophils	*ZBTB7A*	0.2596	0.269	0.120	1.024e-06	2.364e-02
9	Neutrophils	*ADRB2*	0.2590	0.119	0.015	1.342e-21	3.098e-17
9	Neutrophils	*TRIM8*	0.2589	0.239	0.072	4.099e-12	9.465e-08
9	Neutrophils	*LOC103349171*	0.2580	0.172	0.045	1.606e-11	3.710e-07
9	Neutrophils	*ATOSB*	0.2574	0.149	0.045	2.592e-08	5.986e-04
9	Neutrophils	*BCL7B*	0.2569	0.224	0.047	1.301e-19	3.004e-15
9	Neutrophils	*PARP12*	0.2567	0.149	0.047	9.685e-08	2.237e-03
9	Neutrophils	*ZFYVE26*	0.2563	0.209	0.041	2.818e-20	6.507e-16
9	Neutrophils	*CIAO2A*	0.2560	0.313	0.114	4.788e-11	1.106e-06
9	Neutrophils	*RENBP*	0.2558	0.201	0.039	6.347e-20	1.466e-15
9	Neutrophils	*FMO5*	0.2556	0.149	0.042	2.934e-09	6.775e-05
9	Neutrophils	*SF3B5*	0.2552	0.321	0.109	1.295e-12	2.990e-08
9	Neutrophils	*SLC11A2*	0.2547	0.157	0.028	1.246e-17	2.877e-13
9	Neutrophils	*ZNF518B*	0.2539	0.276	0.111	3.798e-08	8.771e-04
9	Neutrophils	*ZDHHC14*	0.2537	0.328	0.157	1.256e-06	2.901e-02
9	Neutrophils	*TMEM127*	0.2533	0.157	0.034	1.320e-13	3.049e-09
9	Neutrophils	*KMT2B*	0.2511	0.187	0.071	1.320e-06	3.049e-02
9	Neutrophils	*LONRF3*	0.2511	0.224	0.089	3.633e-07	8.390e-03
9	Neutrophils	*PPP6C*	0.2510	0.269	0.101	2.612e-09	6.032e-05
9	Neutrophils	*ITPRIP*	0.2508	0.187	0.058	1.212e-09	2.799e-05
9	Neutrophils	*UBE2L6*	0.2505	0.112	0.029	2.875e-08	6.639e-04
11	**CD8+ T cells**	*LOC100349255*	5.3604	0.848	0.004	0	0
11	CD8+ T cells	*CCL5*	5.0230	0.470	0.004	0	0
11	CD8+ T cells	*CCL4*	4.3057	0.455	0.010	0	0
11	CD8+ T cells	*PTPRC*	4.2934	1.000	0.014	0	0
11	CD8+ T cells	*KCNQ5*	4.1800	0.947	0.043	0	0
11	CD8+ T cells	*LOC127493681*	3.7403	0.955	0.013	0	0
11	CD8+ T cells	*RGCC*	3.6432	0.780	0.044	0	0
11	CD8+ T cells	*LOC103345244*	3.6318	0.917	0.002	0	0
11	CD8+ T cells	*LOC138849090*	3.6305	0.833	0.001	0	0
11	CD8+ T cells	*HCST*	3.5037	0.917	0.008	0	0

Original cluster	Annotated cell type	Gene	avg_log2FC	pct.1	pct.2	Raw P value	Bonferroni-adjusted P value
11	CD8+ T cells	*ARHGAP15*	3.4143	0.947	0.024	0	0
11	CD8+ T cells	*LOC103351170*	3.3270	0.985	0.059	0	0
11	CD8+ T cells	*CTSW*	3.2646	0.742	0.019	0	0
11	CD8+ T cells	*IL32*	3.2185	0.917	0.004	0	0
11	CD8+ T cells	*ST8SIA4*	3.1809	0.848	0.068	1.286e-261	2.970e-257
11	CD8+ T cells	*LOC100353676*	3.1777	0.795	0.013	0	0
11	CD8+ T cells	*STK17B*	3.1667	0.947	0.028	0	0
11	CD8+ T cells	*ARPC1B*	3.1428	0.955	0.117	8.258e-221	1.907e-216
11	CD8+ T cells	*LTB*	3.0910	0.955	0.007	0	0
11	CD8+ T cells	*PTPRCAP*	3.0519	0.947	0.015	0	0
11	CD8+ T cells	*NCR3*	2.9880	0.765	0.002	0	0
11	CD8+ T cells	*TARP*	2.9802	0.576	0.001	0	0
11	CD8+ T cells	*LOC103349040*	2.9724	0.492	0.000	0	0
11	CD8+ T cells	*STK17A*	2.9556	0.939	0.101	4.157e-236	9.599e-232
11	CD8+ T cells	*LOC103351679*	2.9431	0.985	0.077	5.389e-291	1.244e-286
11	CD8+ T cells	*PDE3B*	2.9416	0.841	0.127	1.163e-147	2.685e-143
11	CD8+ T cells	*ID2*	2.9367	0.992	0.340	7.941e-106	1.834e-101
11	CD8+ T cells	*LOC100353245*	2.8278	0.939	0.307	4.081e-101	9.424e-97
11	CD8+ T cells	*RIPOR2*	2.8213	0.894	0.108	5.518e-197	1.274e-192
11	CD8+ T cells	*FBXW7*	2.8163	0.811	0.220	1.406e-76	3.246e-72
11	CD8+ T cells	*TOX*	2.7692	0.848	0.068	1.131e-258	2.612e-254
11	CD8+ T cells	*LOC103348990*	2.7478	0.667	0.001	0	0
11	CD8+ T cells	*FCER1G*	2.7426	0.583	0.014	0	0
11	CD8+ T cells	*CD48*	2.7308	0.894	0.015	0	0
11	CD8+ T cells	*PRKCH*	2.7289	0.894	0.055	0	0
11	CD8+ T cells	*TMSB10*	2.7186	1.000	0.960	5.744e-80	1.326e-75
11	CD8+ T cells	*CD3D*	2.6957	0.758	0.000	0	0
11	CD8+ T cells	*CBLB*	2.6849	0.932	0.400	5.998e-67	1.385e-62
11	CD8+ T cells	*SAMSN1*	2.6764	0.924	0.031	0	0
11	CD8+ T cells	*CRIP1*	2.6686	1.000	0.201	7.277e-141	1.680e-136
11	CD8+ T cells	*RGS1*	2.6582	0.636	0.005	0	0
11	CD8+ T cells	*INPP5D*	2.6053	0.758	0.013	0	0
11	CD8+ T cells	*SKAP1*	2.5851	0.667	0.015	0	0
11	CD8+ T cells	*STAT4*	2.5779	0.750	0.017	0	0
11	CD8+ T cells	*LOC103344972*	2.5765	0.758	0.105	1.384e-136	3.197e-132
11	CD8+ T cells	*NR4A2*	2.5745	0.909	0.137	1.721e-153	3.975e-149
11	CD8+ T cells	*REL*	2.5617	0.833	0.058	2.089e-282	4.824e-278
11	CD8+ T cells	*LOC100348315*	2.5383	0.538	0.005	0	0
11	CD8+ T cells	*SH3BGRL3*	2.5357	0.955	0.203	5.974e-140	1.380e-135
11	CD8+ T cells	*PFN1*	2.5045	0.992	0.555	4.010e-84	9.260e-80

Original cluster	Annotated cell type	Gene	avg_log2FC	pct.1	pct.2	Raw P value	Bonferroni-adjusted P value
11	CD8+ T cells	*FXYD5*	2.5045	0.871	0.036	0	0
11	CD8+ T cells	*LOC100328967*	2.5028	0.985	0.270	4.671e-116	1.079e-111
11	CD8+ T cells	*ATP10A*	2.5001	0.652	0.032	5.206e-287	1.202e-282
11	CD8+ T cells	*ETS1*	2.4893	0.902	0.112	2.381e-193	5.498e-189
11	CD8+ T cells	*TM4SF5*	2.4786	0.712	0.001	0	0
11	CD8+ T cells	*B2M*	2.4598	1.000	0.876	6.166e-81	1.424e-76
11	CD8+ T cells	*LOC127492672*	2.4338	0.727	0.001	0	0
11	CD8+ T cells	*CD37*	2.4329	0.894	0.043	0	0
11	CD8+ T cells	*SATB1*	2.4288	0.712	0.068	2.803e-181	6.473e-177
11	CD8+ T cells	*LOC100349085*	2.4248	0.992	0.293	5.549e-111	1.281e-106
11	CD8+ T cells	*FGL2*	2.4221	0.886	0.168	3.354e-124	7.745e-120
11	CD8+ T cells	*INPP4B*	2.4190	0.871	0.231	2.746e-77	6.340e-73
11	CD8+ T cells	*LOC103348991*	2.4026	0.553	0.001	0	0
11	CD8+ T cells	*DOCK8*	2.3937	0.856	0.036	0	0
11	CD8+ T cells	*LOC100339529*	2.3720	0.682	0.001	0	0
11	CD8+ T cells	*ARL4C*	2.3319	0.750	0.025	0	0
11	CD8+ T cells	*FYB1*	2.3210	0.811	0.014	0	0
11	CD8+ T cells	*DOCK2*	2.2981	0.833	0.015	0	0
11	CD8+ T cells	*RAC2*	2.2968	0.811	0.011	0	0
11	CD8+ T cells	*GMFG*	2.2588	0.886	0.034	0	0
11	CD8+ T cells	*HECTD2*	2.2490	0.894	0.339	5.823e-63	1.345e-58
11	CD8+ T cells	*FUT8*	2.2474	0.848	0.157	1.700e-121	3.925e-117
11	CD8+ T cells	*IL12RB2*	2.2435	0.583	0.011	0	0
11	CD8+ T cells	*LCK*	2.2378	0.841	0.010	0	0
11	CD8+ T cells	*SSH2*	2.2289	0.811	0.189	4.539e-89	1.048e-84
11	CD8+ T cells	*PITPNC1*	2.2188	0.742	0.134	1.813e-103	4.188e-99
11	CD8+ T cells	*ELMO1*	2.2081	0.848	0.105	5.631e-167	1.300e-162
11	CD8+ T cells	*DOCK10*	2.1952	0.909	0.264	1.379e-89	3.185e-85
11	CD8+ T cells	*SLC35D1*	2.1930	0.780	0.081	6.563e-185	1.516e-180
11	CD8+ T cells	*CXCR4*	2.1789	0.500	0.041	3.268e-140	7.547e-136
11	CD8+ T cells	*LOC100339772*	2.1719	0.735	0.001	0	0
11	CD8+ T cells	*CSK*	2.1699	0.955	0.152	3.258e-163	7.524e-159
11	CD8+ T cells	*CORO1A*	2.1640	0.848	0.011	0	0
11	CD8+ T cells	*CNOT6L*	2.1615	0.894	0.374	5.063e-62	1.169e-57
11	CD8+ T cells	*IKZF3*	2.1473	0.697	0.003	0	0
11	CD8+ T cells	*LOC100346726*	2.1437	0.712	0.006	0	0
11	CD8+ T cells	*LCP1*	2.1244	0.818	0.026	0	0
11	CD8+ T cells	*CD44*	2.1173	0.788	0.049	2.449e-288	5.656e-284
11	CD8+ T cells	*HOPX*	2.1131	0.826	0.032	0	0
11	CD8+ T cells	*MSN*	2.1101	0.909	0.199	2.034e-115	4.698e-111

Original cluster	Annotated cell type	Gene	avg_log2FC	pct.1	pct.2	Raw P value	Bonferroni-adjusted P value
11	CD8+ T cells	*CD2*	2.0927	0.780	0.000	0	0
11	CD8+ T cells	*RHOH*	2.0852	0.742	0.019	0	0
11	CD8+ T cells	*CD53*	2.0643	0.894	0.011	0	0
11	CD8+ T cells	*SNTB1*	2.0587	0.727	0.066	3.281e-189	7.577e-185
11	CD8+ T cells	*ACSL4*	2.0494	0.712	0.074	5.499e-165	1.270e-160
11	CD8+ T cells	*LAPTM5*	2.0235	0.811	0.012	0	0
11	CD8+ T cells	*LOC100348108*	2.0211	0.811	0.004	0	0
11	CD8+ T cells	*EML4*	2.0203	0.811	0.246	7.429e-69	1.715e-64
11	CD8+ T cells	*ARAP2*	1.9950	0.758	0.024	0	0
11	CD8+ T cells	*AFF3*	1.9883	0.750	0.334	4.147e-38	9.575e-34
11	CD8+ T cells	*APBB1IP*	1.9767	0.727	0.016	0	0
11	CD8+ T cells	*PDE4B*	1.9672	0.598	0.029	3.047e-262	7.035e-258
11	CD8+ T cells	*DUSP1*	1.9582	0.879	0.450	9.358e-39	2.161e-34
11	CD8+ T cells	*CD96*	1.9578	0.735	0.002	0	0
11	CD8+ T cells	*ATP8B4*	1.9563	0.545	0.014	0	0
11	CD8+ T cells	*LCP2*	1.9491	0.705	0.007	0	0
11	CD8+ T cells	*LOC138843111*	1.9477	0.742	0.009	0	0
11	CD8+ T cells	*DENND1B*	1.9417	0.788	0.149	3.651e-102	8.431e-98
11	CD8+ T cells	*UBAC2*	1.9337	0.773	0.138	4.811e-111	1.111e-106
11	CD8+ T cells	*ZFP36L2*	1.9008	0.992	0.631	1.365e-65	3.151e-61
11	CD8+ T cells	*ARPC2*	1.8955	0.955	0.399	2.349e-74	5.424e-70
11	CD8+ T cells	*STK4*	1.8947	0.803	0.126	1.432e-127	3.307e-123
11	CD8+ T cells	*NEDD9*	1.8767	0.758	0.109	5.211e-124	1.203e-119
11	CD8+ T cells	*ARHGDIB*	1.8741	0.833	0.089	6.168e-186	1.424e-181
11	CD8+ T cells	*CST7*	1.8731	0.667	0.001	0	0
11	CD8+ T cells	*RLA-A3*	1.8643	1.000	0.447	4.561e-76	1.053e-71
11	CD8+ T cells	*IKZF2*	1.8552	0.598	0.189	4.086e-37	9.435e-33
11	CD8+ T cells	*ITK*	1.8549	0.727	0.002	0	0
11	CD8+ T cells	*PPP1CB*	1.8537	0.909	0.439	1.117e-59	2.579e-55
11	CD8+ T cells	*RUNX3*	1.8531	0.682	0.002	0	0
11	CD8+ T cells	*TNFAIP8*	1.8524	0.841	0.203	9.405e-87	2.172e-82
11	CD8+ T cells	*FOXO1*	1.8365	0.803	0.286	5.461e-51	1.261e-46
11	CD8+ T cells	*ANKRD44*	1.8353	0.818	0.196	2.512e-85	5.801e-81
11	CD8+ T cells	*PSMB8*	1.8246	0.750	0.031	0	0
11	CD8+ T cells	*VAV3*	1.8238	0.674	0.112	1.751e-93	4.044e-89
11	CD8+ T cells	*PLAAT3*	1.8220	0.848	0.153	9.726e-118	2.246e-113
11	CD8+ T cells	*RUNX2*	1.8217	0.606	0.087	4.633e-100	1.070e-95
11	CD8+ T cells	*LOC100337728*	1.8209	0.402	0.000	0	0
11	CD8+ T cells	*LOC103345076*	1.8007	0.720	0.039	3.573e-294	8.250e-290
11	CD8+ T cells	*CXCR6*	1.7992	0.545	0.001	0	0

Original cluster	Annotated cell type	Gene	avg_log2FC	pct.1	pct.2	Raw P value	Bonferroni-adjusted P value
11	CD8+ T cells	*TUBA4A*	1.7868	0.667	0.032	3.758e-298	8.678e-294
11	CD8+ T cells	*SRRM2*	1.7820	0.985	0.734	1.440e-69	3.325e-65
11	CD8+ T cells	*TAGLN2*	1.7741	0.955	0.406	2.247e-61	5.188e-57
11	CD8+ T cells	*PSMB9*	1.7682	0.750	0.025	0	0
11	CD8+ T cells	*SLC9A9*	1.7612	0.636	0.051	7.684e-180	1.774e-175
11	CD8+ T cells	*CAB39*	1.7584	0.795	0.180	4.851e-87	1.120e-82
11	CD8+ T cells	*SORL1*	1.7545	0.758	0.069	7.217e-197	1.667e-192
11	CD8+ T cells	*RPS6KA5*	1.7361	0.727	0.258	2.793e-47	6.450e-43
11	CD8+ T cells	*CYTIP*	1.7333	0.674	0.027	0	0
11	CD8+ T cells	*TMSB4X*	1.7316	1.000	0.974	4.340e-74	1.002e-69
11	CD8+ T cells	*CD247*	1.7297	0.674	0.002	0	0
11	CD8+ T cells	*LOC100339318*	1.7240	0.788	0.068	2.244e-214	5.182e-210
11	CD8+ T cells	*KLF6*	1.7179	0.864	0.498	4.192e-28	9.681e-24
11	CD8+ T cells	*IL7R*	1.7143	0.273	0.002	0	0
11	CD8+ T cells	*TNFAIP3*	1.7036	0.652	0.066	2.118e-149	4.892e-145
11	CD8+ T cells	*BCL2L11*	1.7012	0.636	0.073	7.357e-130	1.699e-125
11	CD8+ T cells	*RASA2*	1.7000	0.871	0.290	1.362e-63	3.144e-59
11	CD8+ T cells	*ELF1*	1.6935	0.818	0.229	2.456e-70	5.671e-66
11	CD8+ T cells	*SPN*	1.6900	0.652	0.003	0	0
11	CD8+ T cells	*ST6GAL1*	1.6857	0.629	0.062	4.734e-146	1.093e-141
11	CD8+ T cells	*CD226*	1.6800	0.636	0.007	0	0
11	CD8+ T cells	*C12H15orf48*	1.6767	0.553	0.013	0	0
11	CD8+ T cells	*GIMAP4*	1.6716	0.644	0.006	0	0
11	CD8+ T cells	*ARF6*	1.6696	0.917	0.291	4.509e-78	1.041e-73
11	CD8+ T cells	*MBNL1*	1.6690	0.970	0.662	8.671e-44	2.002e-39
11	CD8+ T cells	*RNF19A*	1.6684	0.765	0.329	3.233e-36	7.465e-32
11	CD8+ T cells	*SLC9B2*	1.6655	0.530	0.090	1.522e-71	3.514e-67
11	CD8+ T cells	*BCL11B*	1.6628	0.583	0.001	0	0
11	CD8+ T cells	*SRGN*	1.6624	0.720	0.052	2.310e-216	5.334e-212
11	CD8+ T cells	*RABGAP1L*	1.6577	0.811	0.363	1.802e-41	4.162e-37
11	CD8+ T cells	*NKG7*	1.6570	0.470	0.001	0	0
11	CD8+ T cells	*SYTL3*	1.6562	0.621	0.010	0	0
11	CD8+ T cells	*PTGER4*	1.6509	0.545	0.040	1.911e-165	4.413e-161
11	CD8+ T cells	*ITGA4*	1.6229	0.553	0.042	1.701e-162	3.927e-158
11	CD8+ T cells	*TNFRSF18*	1.6194	0.576	0.006	0	0
11	CD8+ T cells	*ITGB2*	1.6011	0.636	0.011	0	0
11	CD8+ T cells	*PRF1*	1.6002	0.515	0.001	0	0
11	CD8+ T cells	*SLC4A7*	1.5878	0.697	0.163	4.609e-68	1.064e-63
11	CD8+ T cells	*TXK*	1.5826	0.439	0.007	0	0
11	CD8+ T cells	*SGPL1*	1.5802	0.621	0.030	1.277e-270	2.950e-266

Original cluster	Annotated cell type	Gene	avg_log2FC	pct.1	pct.2	Raw P value	Bonferroni-adjusted P value
11	CD8+ T cells	*MAN1A1*	1.5789	0.606	0.155	1.774e-49	4.097e-45
11	CD8+ T cells	*HIF1A*	1.5754	0.735	0.186	2.665e-62	6.155e-58
11	CD8+ T cells	*PPP2R5C*	1.5748	0.705	0.159	9.667e-75	2.232e-70
11	CD8+ T cells	*HCLS1*	1.5652	0.667	0.028	0	0
11	CD8+ T cells	*ABTB3*	1.5588	0.606	0.054	4.578e-154	1.057e-149
11	CD8+ T cells	*GLCCI1*	1.5583	0.545	0.145	1.239e-40	2.861e-36
11	CD8+ T cells	*GATA3*	1.5561	0.500	0.006	0	0
11	CD8+ T cells	*NR3C1*	1.5515	0.886	0.419	3.061e-46	7.067e-42
11	CD8+ T cells	*NCALD*	1.5514	0.538	0.044	1.655e-149	3.822e-145
11	CD8+ T cells	*UST*	1.5490	0.659	0.168	2.540e-52	5.866e-48
11	CD8+ T cells	*GLIPR2*	1.5417	0.674	0.038	5.860e-259	1.353e-254
11	CD8+ T cells	*DPP4*	1.5370	0.470	0.012	0	0
11	CD8+ T cells	*LSP1*	1.5154	0.848	0.168	1.199e-98	2.768e-94
11	CD8+ T cells	*FRYL*	1.5103	0.750	0.234	1.767e-49	4.081e-45
11	CD8+ T cells	*SH2D1A*	1.5090	0.432	0.001	0	0
11	CD8+ T cells	*MFNG*	1.5082	0.689	0.039	2.069e-264	4.778e-260
11	CD8+ T cells	*RGS2*	1.5054	0.523	0.219	2.761e-21	6.376e-17
11	CD8+ T cells	*LOC100343521*	1.4980	0.629	0.000	0	0
11	CD8+ T cells	*CCR5*	1.4909	0.500	0.003	0	0
11	CD8+ T cells	*ICAM3*	1.4906	0.652	0.007	0	0
11	CD8+ T cells	*CD3G*	1.4901	0.553	0.000	0	0
11	CD8+ T cells	*LOC103351160*	1.4891	0.288	0.000	0	0
11	CD8+ T cells	*GPR171*	1.4869	0.614	0.001	0	0
11	CD8+ T cells	*AKAP13*	1.4863	0.742	0.196	5.215e-64	1.204e-59
11	CD8+ T cells	*PIP4K2A*	1.4784	0.818	0.307	9.151e-50	2.113e-45
11	CD8+ T cells	*ZAP70*	1.4725	0.606	0.000	0	0
11	CD8+ T cells	*LOC100352865*	1.4680	0.485	0.033	7.411e-158	1.711e-153
11	CD8+ T cells	*RALGAPA2*	1.4664	0.697	0.189	2.137e-54	4.935e-50
11	CD8+ T cells	*TPM3*	1.4640	0.742	0.182	8.498e-72	1.962e-67
11	CD8+ T cells	*FYN*	1.4574	0.795	0.253	2.376e-56	5.486e-52
11	CD8+ T cells	*B3GNT5*	1.4555	0.583	0.041	1.002e-184	2.313e-180
11	CD8+ T cells	*CRTAM*	1.4490	0.273	0.001	0	0
11	CD8+ T cells	*LOC100355340*	1.4407	0.530	0.000	0	0
11	CD8+ T cells	*CEMIP2*	1.4378	0.508	0.139	5.379e-37	1.242e-32
11	CD8+ T cells	*PIK3R1*	1.4373	0.841	0.478	4.432e-34	1.024e-29
11	CD8+ T cells	*CDC42SE2*	1.4268	0.598	0.085	2.840e-96	6.558e-92
11	CD8+ T cells	*DENND4A*	1.4223	0.644	0.251	5.447e-31	1.258e-26
11	CD8+ T cells	*LOC100354169*	1.4171	0.705	0.154	3.218e-74	7.431e-70
11	CD8+ T cells	*CD8A*	1.4142	0.470	0.000	0	0
11	CD8+ T cells	*PLCL2*	1.4131	0.576	0.076	2.427e-98	5.606e-94

Original cluster	Annotated cell type	Gene	avg_log2FC	pct.1	pct.2	Raw P value	Bonferroni-adjusted P value
11	CD8+ T cells	*TAGAP*	1.4092	0.492	0.005	0	0
11	CD8+ T cells	*PRKCQ*	1.4018	0.667	0.100	1.001e-98	2.311e-94
11	CD8+ T cells	*TNFRSF1B*	1.3999	0.652	0.026	0	0
11	CD8+ T cells	*TNFRSF25*	1.3931	0.553	0.004	0	0
11	CD8+ T cells	*SIK1*	1.3888	0.674	0.100	6.714e-103	1.550e-98
11	CD8+ T cells	*FAM241A*	1.3870	0.576	0.073	5.732e-103	1.324e-98
11	CD8+ T cells	*GNG2*	1.3830	0.758	0.184	3.336e-66	7.703e-62
11	CD8+ T cells	*NFKB1*	1.3763	0.674	0.209	1.500e-41	3.463e-37
11	CD8+ T cells	*RASAL3*	1.3758	0.636	0.004	0	0
11	CD8+ T cells	*JAK1*	1.3726	0.947	0.500	6.131e-46	1.416e-41
11	CD8+ T cells	*LOC100343767*	1.3695	0.523	0.003	0	0
11	CD8+ T cells	*DOK2*	1.3601	0.561	0.009	0	0
11	CD8+ T cells	*PTPN22*	1.3542	0.508	0.008	0	0
11	CD8+ T cells	*RAP1A*	1.3537	0.833	0.342	5.113e-45	1.181e-40
11	CD8+ T cells	*CRLF3*	1.3536	0.689	0.070	6.426e-154	1.484e-149
11	CD8+ T cells	*ELL2*	1.3516	0.644	0.148	8.618e-58	1.990e-53
11	CD8+ T cells	*ITM2C*	1.3503	0.598	0.239	2.200e-26	5.080e-22
11	CD8+ T cells	*TNIK*	1.3499	0.576	0.063	1.453e-119	3.356e-115
11	CD8+ T cells	*ITGB1*	1.3496	0.886	0.452	3.486e-39	8.050e-35
11	CD8+ T cells	*LOC100347754*	1.3480	0.583	0.052	5.397e-149	1.246e-144
11	CD8+ T cells	*CFL1*	1.3432	0.939	0.509	2.174e-47	5.020e-43
11	CD8+ T cells	*UCP2*	1.3392	0.576	0.033	6.867e-214	1.586e-209
11	CD8+ T cells	*CREM*	1.3350	0.682	0.146	2.999e-65	6.926e-61
11	CD8+ T cells	*RRAS2*	1.3268	0.621	0.098	4.798e-84	1.108e-79
11	CD8+ T cells	*CYFIP2*	1.3236	0.561	0.069	1.627e-102	3.757e-98
11	CD8+ T cells	*LOC138848967*	1.3233	0.348	0.014	2.805e-171	6.477e-167
11	CD8+ T cells	*TTC39C*	1.3206	0.447	0.042	1.521e-105	3.512e-101
11	CD8+ T cells	*BACH2*	1.3110	0.515	0.196	4.299e-23	9.928e-19
11	CD8+ T cells	*TESC*	1.2969	0.508	0.021	7.137e-256	1.648e-251
11	CD8+ T cells	*FAM78A*	1.2914	0.500	0.011	0	0
11	CD8+ T cells	*GLIPR1*	1.2900	0.538	0.021	5.801e-278	1.340e-273
11	CD8+ T cells	*ZSWIM6*	1.2894	0.727	0.371	6.379e-22	1.473e-17
11	CD8+ T cells	*FLI1*	1.2843	0.629	0.046	1.721e-186	3.974e-182
11	CD8+ T cells	*MIER1*	1.2815	0.864	0.456	1.225e-33	2.830e-29
11	CD8+ T cells	*HECA*	1.2812	0.614	0.129	2.802e-63	6.472e-59
11	CD8+ T cells	*SIK3*	1.2797	0.652	0.294	7.570e-25	1.748e-20
11	CD8+ T cells	*ARPC3*	1.2750	0.864	0.343	1.152e-50	2.661e-46
11	CD8+ T cells	*IQGAP1*	1.2573	0.750	0.211	8.487e-55	1.960e-50
11	CD8+ T cells	*BAZ1A*	1.2542	0.561	0.057	3.720e-123	8.591e-119
11	CD8+ T cells	*CSGALNACT2*	1.2512	0.644	0.156	1.463e-54	3.377e-50

Original cluster	Annotated cell type	Gene	avg_log2FC	pct.1	pct.2	Raw P value	Bonferroni-adjusted P value
11	CD8+ T cells	*DOCK11*	1.2508	0.576	0.055	6.880e-132	1.589e-127
11	CD8+ T cells	*LOC138850611*	1.2505	0.492	0.004	0	0
11	CD8+ T cells	*BIN2*	1.2501	0.523	0.006	0	0
11	CD8+ T cells	*ARPC5*	1.2492	0.848	0.295	2.833e-53	6.543e-49
11	CD8+ T cells	*SMCO4*	1.2463	0.606	0.071	3.240e-116	7.481e-112
11	CD8+ T cells	*PRMT7*	1.2426	0.561	0.093	5.128e-74	1.184e-69
11	CD8+ T cells	*DNAJC1*	1.2406	0.629	0.289	4.608e-21	1.064e-16
11	CD8+ T cells	*TRAT1*	1.2397	0.485	0.001	0	0
11	CD8+ T cells	*CD28*	1.2390	0.295	0.001	0	0
11	CD8+ T cells	*IPO7*	1.2322	0.659	0.190	4.331e-46	1.000e-41
11	CD8+ T cells	*BCL2A1*	1.2310	0.417	0.012	1.068e-262	2.467e-258
11	CD8+ T cells	*SEPTIN1*	1.2304	0.545	0.020	6.028e-300	1.392e-295
11	CD8+ T cells	*EOMES*	1.2288	0.341	0.000	0	0
11	CD8+ T cells	*NR4A3*	1.2279	0.545	0.140	1.463e-39	3.378e-35
11	CD8+ T cells	*SEPTIN6*	1.2242	0.591	0.081	2.195e-96	5.069e-92
11	CD8+ T cells	*WAS*	1.2238	0.583	0.020	0	0
11	CD8+ T cells	*BANK1*	1.2198	0.356	0.037	2.937e-76	6.781e-72
11	CD8+ T cells	*ABCB1*	1.2187	0.364	0.049	4.521e-57	1.044e-52
11	CD8+ T cells	*SEMA4D*	1.2170	0.659	0.101	8.573e-93	1.980e-88
11	CD8+ T cells	*RASGRP1*	1.2083	0.455	0.020	2.188e-212	5.052e-208
11	CD8+ T cells	*MYH9*	1.2068	0.758	0.211	5.768e-57	1.332e-52
11	CD8+ T cells	*CYRIB*	1.2065	0.621	0.131	4.364e-63	1.008e-58
11	CD8+ T cells	*STK10*	1.2013	0.545	0.028	5.901e-229	1.363e-224
11	CD8+ T cells	*CCND3*	1.1963	0.705	0.233	5.222e-43	1.206e-38
11	CD8+ T cells	*DYNLT1*	1.1919	0.833	0.390	6.579e-36	1.519e-31
11	CD8+ T cells	*BBLN*	1.1893	0.644	0.095	2.396e-96	5.533e-92
11	CD8+ T cells	*UBASH3B*	1.1866	0.417	0.022	1.033e-168	2.386e-164
11	CD8+ T cells	*GPX1*	1.1849	0.902	0.507	3.010e-34	6.950e-30
11	CD8+ T cells	*CD27*	1.1848	0.492	0.002	0	0
11	CD8+ T cells	*ZNRF1*	1.1827	0.530	0.150	7.213e-35	1.666e-30
11	CD8+ T cells	*PPP1R18*	1.1806	0.545	0.056	1.042e-118	2.406e-114
11	CD8+ T cells	*OSTF1*	1.1805	0.833	0.326	1.109e-44	2.560e-40
11	CD8+ T cells	*ACTR3*	1.1801	0.788	0.265	2.204e-50	5.089e-46
11	CD8+ T cells	*CD8B*	1.1798	0.356	0.002	0	0
11	CD8+ T cells	*FNBP1*	1.1790	0.606	0.150	5.839e-52	1.348e-47
11	CD8+ T cells	*SASH3*	1.1786	0.568	0.003	0	0
11	CD8+ T cells	*TRAF3IP3*	1.1784	0.538	0.022	2.094e-262	4.836e-258
11	CD8+ T cells	*IL2RG*	1.1760	0.553	0.003	0	0
11	CD8+ T cells	*ARHGAP25*	1.1711	0.455	0.018	4.131e-235	9.540e-231
11	CD8+ T cells	*PTPRE*	1.1638	0.545	0.039	8.566e-169	1.978e-164

Original cluster	Annotated cell type	Gene	avg_log2FC	pct.1	pct.2	Raw P value	Bonferroni-adjusted P value
11	CD8+ T cells	*PTK2B*	1.1616	0.492	0.011	0	0
11	CD8+ T cells	*LAT*	1.1603	0.447	0.000	0	0
11	CD8+ T cells	*ARHGAP26*	1.1598	0.515	0.066	1.927e-90	4.449e-86
11	CD8+ T cells	*LOC100351163*	1.1585	0.727	0.059	1.741e-200	4.020e-196
11	CD8+ T cells	*COTL1*	1.1575	0.598	0.130	5.433e-58	1.255e-53
11	CD8+ T cells	*CD200R1*	1.1570	0.318	0.002	0	0
11	CD8+ T cells	*APRT*	1.1509	0.735	0.183	3.120e-61	7.204e-57
11	CD8+ T cells	*TSPAN5*	1.1469	0.545	0.092	4.557e-67	1.052e-62
11	CD8+ T cells	*RCSD1*	1.1448	0.598	0.092	1.799e-85	4.154e-81
11	CD8+ T cells	*LIMD2*	1.1418	0.500	0.055	9.245e-103	2.135e-98
11	CD8+ T cells	*CDK2AP2*	1.1417	0.636	0.097	7.506e-91	1.733e-86
11	CD8+ T cells	*HYCC1*	1.1399	0.515	0.141	1.835e-35	4.237e-31
11	CD8+ T cells	*B3GNT2*	1.1387	0.530	0.070	1.082e-88	2.498e-84
11	CD8+ T cells	*COQ10B*	1.1377	0.765	0.253	7.834e-45	1.809e-40
11	CD8+ T cells	*PSME1*	1.1346	0.765	0.228	3.962e-50	9.149e-46
11	CD8+ T cells	*LOC100350928*	1.1341	0.545	0.083	4.436e-79	1.024e-74
11	CD8+ T cells	*ARHGEF1*	1.1305	0.614	0.075	1.450e-111	3.349e-107
11	CD8+ T cells	*YWHAB*	1.1303	0.833	0.405	1.231e-31	2.843e-27
11	CD8+ T cells	*TENT5C*	1.1208	0.553	0.211	1.147e-24	2.649e-20
11	CD8+ T cells	*MAML2*	1.1188	0.705	0.471	4.356e-12	1.006e-07
11	CD8+ T cells	*MOB3A*	1.1170	0.674	0.156	2.430e-59	5.611e-55
11	CD8+ T cells	*BZW2*	1.1155	0.591	0.150	2.676e-46	6.179e-42
11	CD8+ T cells	*SEPTIN11*	1.1153	0.591	0.156	3.454e-44	7.977e-40
11	CD8+ T cells	*TGFB1*	1.1136	0.553	0.065	2.945e-104	6.801e-100
11	CD8+ T cells	*LOC108177063*	1.1119	0.333	0.000	0	0
11	CD8+ T cells	*NHP2*	1.1116	0.538	0.084	1.152e-75	2.661e-71
11	CD8+ T cells	*KIF2A*	1.1079	0.780	0.340	3.413e-33	7.881e-29
11	CD8+ T cells	*PABPC1*	1.1069	0.985	0.874	3.972e-42	9.173e-38
11	CD8+ T cells	*ERO1A*	1.1050	0.598	0.101	8.309e-75	1.919e-70
11	CD8+ T cells	*FCHSD2*	1.1039	0.667	0.238	4.875e-31	1.126e-26
11	CD8+ T cells	*S100A10*	1.1024	0.811	0.345	1.279e-33	2.954e-29
11	CD8+ T cells	*SP4*	1.1011	0.545	0.171	6.925e-32	1.599e-27
11	CD8+ T cells	*FAM107B*	1.0962	0.659	0.194	4.429e-40	1.023e-35
11	CD8+ T cells	*MARK2*	1.0945	0.553	0.079	3.228e-85	7.454e-81
11	CD8+ T cells	*WARS1*	1.0916	0.447	0.069	4.728e-62	1.092e-57
11	CD8+ T cells	*RNF125*	1.0888	0.583	0.150	1.045e-44	2.414e-40
11	CD8+ T cells	*LOC138850174*	1.0869	0.455	0.049	1.233e-92	2.847e-88
11	CD8+ T cells	*LOC100350425*	1.0845	0.318	0.039	3.775e-57	8.718e-53
11	CD8+ T cells	*PSTPIP1*	1.0836	0.477	0.007	0	0
11	CD8+ T cells	*IKZF1*	1.0832	0.432	0.006	0	0

Original cluster	Annotated cell type	Gene	avg_log2FC	pct.1	pct.2	Raw P value	Bonferroni-adjusted P value
11	CD8+ T cells	*PRDM2*	1.0825	0.455	0.123	1.764e-32	4.074e-28
11	CD8+ T cells	*LOC127483256*	1.0810	0.348	0.032	1.327e-83	3.065e-79
11	CD8+ T cells	*SDF2L1*	1.0801	0.447	0.016	1.475e-244	3.406e-240
11	CD8+ T cells	*LOC100358069*	1.0786	0.955	0.660	1.623e-37	3.747e-33
11	CD8+ T cells	*LRBA*	1.0777	0.803	0.352	4.928e-32	1.138e-27
11	CD8+ T cells	*LBR*	1.0722	0.606	0.142	1.347e-52	3.110e-48
11	CD8+ T cells	*LPAR6*	1.0702	0.432	0.039	3.543e-107	8.182e-103
11	CD8+ T cells	*CYTH1*	1.0694	0.644	0.164	1.716e-50	3.963e-46
11	CD8+ T cells	*SKIL*	1.0660	0.576	0.160	2.731e-39	6.305e-35
11	CD8+ T cells	*LOC100346112*	1.0645	0.917	0.584	9.556e-36	2.207e-31
11	CD8+ T cells	*FOXN3*	1.0631	0.629	0.276	4.247e-24	9.808e-20
11	CD8+ T cells	*ARHGEF3*	1.0582	0.500	0.068	1.169e-78	2.700e-74
11	CD8+ T cells	*MGAT4A*	1.0540	0.402	0.077	1.897e-43	4.381e-39
11	CD8+ T cells	*VPS37B*	1.0539	0.500	0.063	3.896e-87	8.998e-83
11	CD8+ T cells	*SP100*	1.0537	0.417	0.022	8.358e-169	1.930e-164
11	CD8+ T cells	*RPS21*	1.0519	0.985	0.981	2.682e-55	6.194e-51
11	CD8+ T cells	*ACTR2*	1.0517	0.788	0.293	4.866e-40	1.124e-35
11	CD8+ T cells	*PARP4*	1.0507	0.553	0.120	6.913e-51	1.596e-46
11	CD8+ T cells	*CHST11*	1.0491	0.508	0.081	1.300e-68	3.002e-64
11	CD8+ T cells	*RILPL2*	1.0486	0.864	0.382	1.679e-36	3.878e-32
11	CD8+ T cells	*MYCBP2*	1.0475	0.833	0.452	6.330e-26	1.462e-21
11	CD8+ T cells	*ATF6*	1.0444	0.644	0.161	9.515e-50	2.197e-45
11	CD8+ T cells	*HINT1*	1.0424	0.795	0.396	7.148e-29	1.651e-24
11	CD8+ T cells	*PREX1*	1.0350	0.432	0.017	2.775e-219	6.408e-215
11	CD8+ T cells	*ARHGAP45*	1.0310	0.477	0.006	0	0
11	CD8+ T cells	*IL18R1*	1.0303	0.235	0.001	0	0
11	CD8+ T cells	*WIPF1*	1.0219	0.636	0.186	6.658e-41	1.537e-36
11	CD8+ T cells	*SMURF2*	1.0137	0.621	0.239	2.291e-24	5.291e-20
11	CD8+ T cells	*TPT1*	1.0104	0.985	0.987	6.045e-57	1.396e-52
11	CD8+ T cells	*LOC138847635*	1.0103	0.295	0.000	0	0
11	CD8+ T cells	*CALM1*	1.0096	0.970	0.744	9.259e-33	2.138e-28
11	CD8+ T cells	*EPB41*	1.0095	0.689	0.255	3.317e-33	7.660e-29
11	CD8+ T cells	*ERN1*	1.0080	0.439	0.060	1.820e-70	4.204e-66
11	CD8+ T cells	*MAP3K2*	1.0077	0.705	0.359	5.624e-21	1.299e-16
11	CD8+ T cells	*LOC127489568*	1.0052	0.705	0.320	2.774e-27	6.406e-23
11	CD8+ T cells	*SEPTIN9*	1.0016	0.568	0.121	1.617e-54	3.735e-50
11	CD8+ T cells	*STAP1*	1.0008	0.250	0.005	2.780e-214	6.421e-210
11	CD8+ T cells	*UBA52*	1.0001	0.985	0.980	2.486e-54	5.740e-50
11	CD8+ T cells	*BICDL1*	0.9988	0.311	0.004	0	0
11	CD8+ T cells	*ATP8A1*	0.9971	0.477	0.094	1.225e-47	2.830e-43

Original cluster	Annotated cell type	Gene	avg_log2FC	pct.1	pct.2	Raw P value	Bonferroni-adjusted P value
11	CD8+ T cells	*PAK2*	0.9935	0.826	0.370	2.192e-28	5.062e-24
11	CD8+ T cells	*CAPZA1*	0.9930	0.750	0.288	9.449e-35	2.182e-30
11	CD8+ T cells	*SELPLG*	0.9920	0.455	0.011	0	0
11	CD8+ T cells	*DHRS7*	0.9896	0.492	0.052	9.297e-104	2.147e-99
11	CD8+ T cells	*VASP*	0.9872	0.598	0.101	8.667e-74	2.001e-69
11	CD8+ T cells	*LFNG*	0.9872	0.402	0.018	8.460e-186	1.954e-181
11	CD8+ T cells	*SYTL2*	0.9866	0.447	0.027	4.248e-155	9.810e-151
11	CD8+ T cells	*TAOK3*	0.9843	0.591	0.204	3.954e-30	9.130e-26
11	CD8+ T cells	*ZC3HAV1*	0.9803	0.477	0.091	1.157e-51	2.671e-47
11	CD8+ T cells	*CMTM6*	0.9788	0.576	0.156	4.788e-39	1.106e-34
11	CD8+ T cells	*ITSN2*	0.9771	0.674	0.234	4.293e-35	9.914e-31
11	CD8+ T cells	*DDX3X*	0.9769	0.917	0.592	5.546e-25	1.281e-20
11	CD8+ T cells	*TANK*	0.9759	0.508	0.169	1.224e-23	2.827e-19
11	CD8+ T cells	*CHD2*	0.9745	0.682	0.328	3.517e-19	8.122e-15
11	CD8+ T cells	*LOC138849471*	0.9744	0.356	0.000	0	0
11	CD8+ T cells	*CLIC1*	0.9731	0.886	0.534	7.883e-26	1.820e-21
11	CD8+ T cells	*GSAP*	0.9722	0.386	0.029	1.290e-111	2.979e-107
11	CD8+ T cells	*HIVEP2*	0.9674	0.780	0.452	9.609e-17	2.219e-12
11	CD8+ T cells	*PPP1R14B*	0.9667	0.667	0.243	7.138e-32	1.648e-27
11	CD8+ T cells	*KLRK1*	0.9650	0.371	0.000	0	0
11	CD8+ T cells	*GRK3*	0.9623	0.477	0.007	0	0
11	CD8+ T cells	*BTBD9*	0.9617	0.629	0.253	1.660e-23	3.833e-19
11	CD8+ T cells	*RB1*	0.9606	0.689	0.268	2.027e-28	4.682e-24
11	CD8+ T cells	*JARID2*	0.9605	0.515	0.178	4.414e-25	1.019e-20
11	CD8+ T cells	*RPS11*	0.9601	1.000	0.981	6.049e-40	1.397e-35
11	CD8+ T cells	*RNF122*	0.9593	0.492	0.103	3.119e-44	7.202e-40
11	CD8+ T cells	*INPP4A*	0.9586	0.364	0.066	3.059e-41	7.063e-37
11	CD8+ T cells	*FERMT3*	0.9583	0.424	0.011	1.925e-287	4.445e-283
11	CD8+ T cells	*SLC25A51*	0.9577	0.492	0.075	2.237e-68	5.166e-64
11	CD8+ T cells	*SIPA1*	0.9546	0.462	0.035	2.869e-136	6.624e-132
11	CD8+ T cells	*SKAP2*	0.9542	0.341	0.041	3.555e-61	8.208e-57
11	CD8+ T cells	*MACF1*	0.9535	0.864	0.479	2.349e-27	5.425e-23
11	CD8+ T cells	*ABRACL*	0.9528	0.447	0.037	1.721e-116	3.974e-112
11	CD8+ T cells	*DOCK5*	0.9523	0.409	0.041	2.541e-89	5.867e-85
11	CD8+ T cells	*ST3GAL5*	0.9515	0.402	0.019	3.505e-179	8.095e-175
11	CD8+ T cells	*ARHGAP30*	0.9503	0.394	0.008	0	0
11	CD8+ T cells	*KLF13*	0.9488	0.614	0.169	2.359e-41	5.448e-37
11	CD8+ T cells	*FASLG*	0.9479	0.250	0.000	0	0
11	CD8+ T cells	*FRS2*	0.9447	0.561	0.250	4.531e-18	1.046e-13
11	CD8+ T cells	*CTPS2*	0.9447	0.523	0.172	2.057e-27	4.751e-23

Original cluster	Annotated cell type	Gene	avg_log2FC	pct.1	pct.2	Raw P value	Bonferroni-adjusted P value
11	CD8+ T cells	*FMNL1*	0.9427	0.447	0.006	0	0
11	CD8+ T cells	*CDKN1B*	0.9424	0.591	0.200	1.636e-29	3.777e-25
11	CD8+ T cells	*EIF2AK3*	0.9411	0.432	0.075	7.891e-52	1.822e-47
11	CD8+ T cells	*ELK4*	0.9388	0.561	0.172	6.610e-32	1.526e-27
11	CD8+ T cells	*RPLP2*	0.9377	0.985	0.919	2.791e-40	6.446e-36
11	CD8+ T cells	*UBALD2*	0.9362	0.598	0.153	1.323e-43	3.056e-39
11	CD8+ T cells	*SLAMF1*	0.9328	0.258	0.005	1.173e-219	2.708e-215
11	CD8+ T cells	*DHRS3*	0.9322	0.447	0.057	6.378e-77	1.473e-72
11	CD8+ T cells	*JAML*	0.9320	0.364	0.010	1.916e-235	4.425e-231
11	CD8+ T cells	*EEIG1*	0.9310	0.477	0.046	3.413e-108	7.882e-104
11	CD8+ T cells	*STK26*	0.9296	0.485	0.057	1.134e-89	2.618e-85
11	CD8+ T cells	*PHF20L1*	0.9288	0.682	0.277	1.041e-28	2.404e-24
11	CD8+ T cells	*LOC108176263*	0.9287	0.288	0.001	0	0
11	CD8+ T cells	*EPRS1*	0.9259	0.492	0.277	3.943e-08	9.106e-04
11	CD8+ T cells	*DOCK9*	0.9241	0.545	0.200	3.736e-23	8.628e-19
11	CD8+ T cells	*CASP10*	0.9241	0.364	0.029	2.409e-102	5.563e-98
11	CD8+ T cells	*GPR18*	0.9240	0.371	0.001	0	0
11	CD8+ T cells	*CXCR3*	0.9221	0.371	0.000	0	0
11	CD8+ T cells	*STK39*	0.9145	0.689	0.219	3.194e-36	7.376e-32
11	CD8+ T cells	*TCF7*	0.9142	0.455	0.070	2.391e-62	5.521e-58
11	CD8+ T cells	*SMARCA5*	0.9090	0.712	0.339	9.172e-22	2.118e-17
11	CD8+ T cells	*CTLA4*	0.9080	0.136	0.000	2.604e-258	6.013e-254
11	CD8+ T cells	*BTAF1*	0.9064	0.424	0.176	5.286e-14	1.221e-09
11	CD8+ T cells	*SEPTIN7*	0.9047	0.970	0.690	1.503e-29	3.472e-25
11	CD8+ T cells	*NSMAF*	0.9042	0.447	0.068	8.822e-61	2.037e-56
11	CD8+ T cells	*MGAT5*	0.9010	0.561	0.183	1.150e-29	2.655e-25
11	CD8+ T cells	*UBE2G1*	0.9007	0.621	0.217	3.110e-30	7.182e-26
11	CD8+ T cells	*PPP1CC*	0.9005	0.773	0.388	1.288e-22	2.974e-18
11	CD8+ T cells	*CDR2*	0.8943	0.409	0.019	1.996e-178	4.610e-174
11	CD8+ T cells	*NUP210*	0.8935	0.379	0.022	5.818e-140	1.344e-135
11	CD8+ T cells	*PLEKHJ1*	0.8932	0.644	0.175	1.594e-42	3.681e-38
11	CD8+ T cells	*SLC25A5*	0.8913	0.803	0.390	3.656e-26	8.442e-22
11	CD8+ T cells	*ST8SIA1*	0.8899	0.364	0.006	0	0
11	CD8+ T cells	*PTPN9*	0.8887	0.538	0.131	1.413e-41	3.262e-37
11	CD8+ T cells	*GJA3*	0.8878	0.333	0.000	0	0
11	CD8+ T cells	*ZFAND3*	0.8852	0.689	0.295	6.281e-25	1.450e-20
11	CD8+ T cells	*LIN54*	0.8849	0.348	0.078	2.286e-30	5.279e-26
11	CD8+ T cells	*CD55*	0.8849	0.629	0.221	4.985e-29	1.151e-24
11	CD8+ T cells	*BFAR*	0.8837	0.432	0.058	1.138e-68	2.629e-64
11	CD8+ T cells	*CHSY1*	0.8820	0.432	0.100	9.704e-37	2.241e-32

Original cluster	Annotated cell type	Gene	avg_log2FC	pct.1	pct.2	Raw P value	Bonferroni-adjusted P value
11	CD8+ T cells	*NDFIP2*	0.8818	0.629	0.252	3.568e-26	8.238e-22
11	CD8+ T cells	*LPIN2*	0.8809	0.545	0.150	2.352e-35	5.431e-31
11	CD8+ T cells	*CMC1*	0.8805	0.508	0.190	3.262e-20	7.533e-16
11	CD8+ T cells	*CCDC69*	0.8803	0.402	0.011	9.063e-274	2.093e-269
11	CD8+ T cells	*ARL5B*	0.8737	0.439	0.183	8.957e-15	2.068e-10
11	CD8+ T cells	*RESF1*	0.8721	0.439	0.071	3.465e-57	8.000e-53
11	CD8+ T cells	*GPR65*	0.8689	0.303	0.011	7.941e-168	1.834e-163
11	CD8+ T cells	*GRAMD1B*	0.8658	0.409	0.043	4.767e-84	1.101e-79
11	CD8+ T cells	*MYO5A*	0.8632	0.447	0.097	5.512e-40	1.273e-35
11	CD8+ T cells	*TOMM20*	0.8612	0.758	0.405	3.559e-21	8.218e-17
11	CD8+ T cells	*ABI1*	0.8612	0.674	0.278	6.149e-26	1.420e-21
11	CD8+ T cells	*ANKRD17*	0.8596	0.697	0.411	1.419e-12	3.276e-08
11	CD8+ T cells	*SGMS1*	0.8594	0.636	0.309	2.817e-17	6.505e-13
11	CD8+ T cells	*LRRC8C*	0.8583	0.379	0.058	3.321e-51	7.669e-47
11	CD8+ T cells	*ARHGEF6*	0.8567	0.447	0.064	6.558e-66	1.514e-61
11	CD8+ T cells	*SUSD3*	0.8560	0.477	0.064	1.846e-73	4.264e-69
11	CD8+ T cells	*GALNT3*	0.8535	0.280	0.012	3.077e-138	7.105e-134
11	CD8+ T cells	*SH3KBP1*	0.8530	0.553	0.304	3.038e-13	7.015e-09
11	CD8+ T cells	*PARN*	0.8520	0.477	0.119	1.223e-35	2.825e-31
11	CD8+ T cells	*CCSER2*	0.8499	0.553	0.275	3.434e-15	7.930e-11
11	CD8+ T cells	*MAP3K8*	0.8498	0.273	0.029	2.434e-57	5.620e-53
11	CD8+ T cells	*ICOS*	0.8494	0.212	0.000	0	0
11	CD8+ T cells	*CCDC138*	0.8486	0.379	0.079	7.572e-36	1.748e-31
11	CD8+ T cells	*TSC22D3*	0.8471	0.705	0.379	1.277e-16	2.948e-12
11	CD8+ T cells	*CCR2*	0.8465	0.265	0.001	0	0
11	CD8+ T cells	*SOCS1*	0.8463	0.652	0.274	3.239e-21	7.479e-17
11	CD8+ T cells	*UBE2H*	0.8438	0.689	0.311	1.776e-21	4.102e-17
11	CD8+ T cells	*IFNAR2*	0.8425	0.364	0.096	4.006e-25	9.251e-21
11	CD8+ T cells	*LOC127482786*	0.8425	0.386	0.027	3.648e-119	8.425e-115
11	CD8+ T cells	*CYLD*	0.8419	0.492	0.126	2.096e-34	4.840e-30
11	CD8+ T cells	*NT5C3A*	0.8375	0.576	0.197	1.567e-27	3.619e-23
11	CD8+ T cells	*PLP2*	0.8375	0.500	0.127	3.689e-36	8.520e-32
11	CD8+ T cells	*THEMIS*	0.8373	0.182	0.001	4.498e-240	1.039e-235
11	CD8+ T cells	*IL21R*	0.8372	0.288	0.003	0	0
11	CD8+ T cells	*CAP1*	0.8365	0.652	0.208	5.143e-33	1.188e-28
11	CD8+ T cells	*OXCT1*	0.8359	0.394	0.098	1.427e-29	3.295e-25
11	CD8+ T cells	*SYAP1*	0.8357	0.515	0.141	2.736e-33	6.317e-29
11	CD8+ T cells	*TEC*	0.8356	0.417	0.149	2.065e-17	4.767e-13
11	CD8+ T cells	*PDE7A*	0.8343	0.341	0.085	1.156e-24	2.670e-20
11	CD8+ T cells	*NEK10*	0.8337	0.250	0.011	2.988e-114	6.901e-110

Original cluster	Annotated cell type	Gene	avg_log2FC	pct.1	pct.2	Raw P value	Bonferroni-adjusted P value
11	CD8+ T cells	*PHF20*	0.8325	0.523	0.172	1.923e-27	4.440e-23
11	CD8+ T cells	*NDFIP1*	0.8320	0.955	0.651	1.889e-25	4.361e-21
11	CD8+ T cells	*TNFSF10*	0.8315	0.447	0.063	2.443e-65	5.640e-61
11	CD8+ T cells	*GPR174*	0.8295	0.189	0.001	3.576e-294	8.257e-290
11	CD8+ T cells	*NIBAN1*	0.8291	0.364	0.013	1.925e-203	4.444e-199
11	CD8+ T cells	*B3GNT7*	0.8281	0.326	0.005	0	0
11	CD8+ T cells	*ETV6*	0.8276	0.492	0.182	3.747e-20	8.653e-16
11	CD8+ T cells	*DSE*	0.8276	0.508	0.134	5.306e-34	1.225e-29
11	CD8+ T cells	*HUWE1*	0.8254	0.727	0.333	8.877e-21	2.050e-16
11	CD8+ T cells	*CMTM7*	0.8251	0.614	0.205	8.011e-29	1.850e-24
11	CD8+ T cells	*ITGAE*	0.8238	0.288	0.044	6.021e-40	1.390e-35
11	CD8+ T cells	*DNAJB6*	0.8236	0.553	0.210	1.871e-22	4.321e-18
11	CD8+ T cells	*ARHGAP9*	0.8216	0.417	0.022	4.381e-161	1.012e-156
11	CD8+ T cells	*RICTOR*	0.8204	0.598	0.345	1.029e-13	2.375e-09
11	CD8+ T cells	*CCR4*	0.8190	0.197	0.000	0	0
11	CD8+ T cells	*ATP6V1G1*	0.8189	0.864	0.480	7.268e-24	1.678e-19
11	CD8+ T cells	*BRAF*	0.8180	0.561	0.332	2.797e-09	6.458e-05
11	CD8+ T cells	*ACTB*	0.8176	1.000	0.971	5.392e-30	1.245e-25
11	CD8+ T cells	*ARHGDIA*	0.8171	0.750	0.368	2.273e-23	5.250e-19
11	CD8+ T cells	*LOC138843527*	0.8168	0.955	0.658	7.604e-26	1.756e-21
11	CD8+ T cells	*LOC138844609*	0.8160	0.386	0.101	1.582e-26	3.654e-22
11	CD8+ T cells	*ENO3*	0.8159	0.409	0.051	2.178e-70	5.030e-66
11	CD8+ T cells	*LOC100342250*	0.8135	0.341	0.003	0	0
11	CD8+ T cells	*LOC127490692*	0.8129	0.250	0.000	0	0
11	CD8+ T cells	*FAM111A*	0.8100	0.379	0.058	1.893e-51	4.372e-47
11	CD8+ T cells	*S100A11*	0.8099	0.826	0.751	4.269e-13	9.859e-09
11	CD8+ T cells	*DBI*	0.8063	0.758	0.293	8.967e-30	2.071e-25
11	CD8+ T cells	*TRABD2A*	0.8059	0.333	0.026	3.074e-93	7.098e-89
11	CD8+ T cells	*KCNA3*	0.8057	0.273	0.005	6.525e-233	1.507e-228
11	CD8+ T cells	*TLN1*	0.8054	0.727	0.330	7.074e-23	1.633e-18
11	CD8+ T cells	*PAG1*	0.8039	0.364	0.022	3.397e-128	7.843e-124
11	CD8+ T cells	*NCOA2*	0.8012	0.795	0.510	4.345e-13	1.003e-08
11	CD8+ T cells	*ACP5*	0.8007	0.311	0.006	4.266e-259	9.852e-255
11	CD8+ T cells	*MAP2K3*	0.8006	0.409	0.063	9.254e-55	2.137e-50
11	CD8+ T cells	*DBN1*	0.7996	0.386	0.043	2.834e-75	6.545e-71
11	CD8+ T cells	*LOC138849940*	0.7993	0.970	0.932	3.469e-34	8.012e-30
11	CD8+ T cells	*VAMP8*	0.7987	0.553	0.155	9.043e-34	2.088e-29
11	CD8+ T cells	*STXBP1*	0.7986	0.417	0.040	4.077e-94	9.415e-90
11	CD8+ T cells	*CASP8*	0.7967	0.318	0.007	2.895e-236	6.685e-232
11	CD8+ T cells	*LOC103348133*	0.7940	0.311	0.019	5.356e-107	1.237e-102

Original cluster	Annotated cell type	Gene	avg_log2FC	pct.1	pct.2	Raw P value	Bonferroni-adjusted P value
11	CD8+ T cells	*IL18RAP*	0.7939	0.295	0.002	0	0
11	CD8+ T cells	*C19H16orf54*	0.7939	0.333	0.006	3.568e-295	8.238e-291
11	CD8+ T cells	*LOC100346467*	0.7936	0.386	0.045	7.945e-71	1.835e-66
11	CD8+ T cells	*RHOA*	0.7933	0.932	0.609	2.614e-26	6.036e-22
11	CD8+ T cells	*HHEX*	0.7924	0.235	0.019	1.823e-62	4.210e-58
11	CD8+ T cells	*HTATSF1*	0.7903	0.561	0.269	2.147e-15	4.959e-11
11	CD8+ T cells	*GDAP2*	0.7897	0.341	0.089	3.177e-23	7.337e-19
11	CD8+ T cells	*LNPEP*	0.7884	0.455	0.114	8.703e-33	2.010e-28
11	CD8+ T cells	*GRB2*	0.7866	0.576	0.202	2.106e-26	4.862e-22
11	CD8+ T cells	*LOC138847422*	0.7846	0.333	0.004	0	0
11	CD8+ T cells	*GPC3*	0.7845	0.311	0.059	1.098e-31	2.536e-27
11	CD8+ T cells	*CD274*	0.7832	0.326	0.047	6.871e-48	1.587e-43
11	CD8+ T cells	*MAPRE2*	0.7825	0.614	0.270	8.558e-17	1.976e-12
11	CD8+ T cells	*ESYT2*	0.7815	0.508	0.233	5.379e-14	1.242e-09
11	CD8+ T cells	*SMC4*	0.7811	0.455	0.137	3.903e-25	9.013e-21
11	CD8+ T cells	*SIPA1L1*	0.7803	0.644	0.250	8.668e-24	2.002e-19
11	CD8+ T cells	*CDC42*	0.7753	0.939	0.629	1.846e-21	4.262e-17
11	CD8+ T cells	*EIF4G3*	0.7745	0.621	0.299	1.193e-16	2.754e-12
11	CD8+ T cells	*JMJD4*	0.7729	0.235	0.028	4.132e-42	9.541e-38
11	CD8+ T cells	*SH2D2A*	0.7721	0.303	0.000	0	0
11	CD8+ T cells	*TBC1D10C*	0.7712	0.364	0.008	6.661e-276	1.538e-271
11	CD8+ T cells	*KDM4C*	0.7706	0.462	0.133	6.537e-28	1.510e-23
11	CD8+ T cells	*ABR*	0.7697	0.394	0.075	3.255e-42	7.517e-38
11	CD8+ T cells	*RCAN3*	0.7684	0.295	0.015	3.267e-124	7.544e-120
11	CD8+ T cells	*RNF138*	0.7679	0.523	0.241	2.607e-13	6.020e-09
11	CD8+ T cells	*FOSL2*	0.7679	0.659	0.411	4.965e-12	1.147e-07
11	CD8+ T cells	*CMIP*	0.7650	0.455	0.100	6.914e-40	1.597e-35
11	CD8+ T cells	*CORO1C*	0.7637	0.409	0.075	2.663e-45	6.150e-41
11	CD8+ T cells	*TRPM7*	0.7625	0.591	0.312	7.089e-13	1.637e-08
11	CD8+ T cells	*PPP4R3A*	0.7623	0.705	0.263	3.243e-28	7.489e-24
11	CD8+ T cells	*DGKD*	0.7622	0.348	0.060	2.453e-41	5.663e-37
11	CD8+ T cells	*SUSD6*	0.7620	0.447	0.085	8.250e-46	1.905e-41
11	CD8+ T cells	*ANXA2*	0.7614	0.538	0.170	4.482e-27	1.035e-22
11	CD8+ T cells	*TNRC6B*	0.7611	0.803	0.494	4.887e-16	1.128e-11
11	CD8+ T cells	*ASAP1*	0.7596	0.614	0.314	2.938e-15	6.784e-11
11	CD8+ T cells	*EDARADD*	0.7578	0.273	0.038	5.909e-41	1.364e-36
11	CD8+ T cells	*LOC138850498*	0.7570	0.303	0.003	0	0
11	CD8+ T cells	*PPP6R3*	0.7564	0.515	0.220	2.028e-16	4.684e-12
11	CD8+ T cells	*FGFR1OP2*	0.7557	0.644	0.261	2.712e-22	6.261e-18
11	CD8+ T cells	*ASXL2*	0.7542	0.545	0.199	2.032e-22	4.692e-18

Original cluster	Annotated cell type	Gene	avg_log2FC	pct.1	pct.2	Raw P value	Bonferroni-adjusted P value
11	CD8+ T cells	*CAPN2*	0.7539	0.530	0.167	3.424e-28	7.907e-24
11	CD8+ T cells	*CERT1*	0.7529	0.500	0.160	6.173e-25	1.425e-20
11	CD8+ T cells	*BPTF*	0.7520	0.780	0.457	7.085e-17	1.636e-12
11	CD8+ T cells	*TP53I11*	0.7513	0.341	0.017	1.186e-145	2.738e-141
11	CD8+ T cells	*LOC100340954*	0.7510	0.848	0.449	4.082e-22	9.425e-18
11	CD8+ T cells	*CREB1*	0.7506	0.667	0.354	8.614e-14	1.989e-09
11	CD8+ T cells	*TRAM1*	0.7505	0.727	0.369	3.008e-18	6.947e-14
11	CD8+ T cells	*CERS6*	0.7503	0.424	0.131	1.479e-21	3.415e-17
11	CD8+ T cells	*ROCK2*	0.7501	0.583	0.344	9.957e-10	2.299e-05
11	CD8+ T cells	*VAV1*	0.7484	0.326	0.005	1.503e-297	3.472e-293
11	CD8+ T cells	*TBL1XR1*	0.7453	0.803	0.512	1.252e-13	2.890e-09
11	CD8+ T cells	*BHLHE40*	0.7446	0.447	0.168	1.284e-16	2.965e-12
11	CD8+ T cells	*RAP1GDS1*	0.7446	0.447	0.145	1.279e-21	2.955e-17
11	CD8+ T cells	*MTHFD2*	0.7430	0.326	0.027	2.503e-87	5.780e-83
11	CD8+ T cells	*PHLDA1*	0.7412	0.326	0.079	7.894e-25	1.823e-20
11	CD8+ T cells	*FAR2*	0.7411	0.235	0.024	9.477e-51	2.189e-46
11	CD8+ T cells	*CAMK4*	0.7398	0.311	0.002	0	0
11	CD8+ T cells	*COX4I1*	0.7392	0.970	0.917	1.329e-29	3.070e-25
11	CD8+ T cells	*TNFRSF4*	0.7385	0.265	0.003	5.829e-285	1.346e-280
11	CD8+ T cells	*B4GALT5*	0.7382	0.333	0.038	2.921e-64	6.746e-60
11	CD8+ T cells	*PGK1*	0.7379	0.568	0.245	4.953e-18	1.144e-13
11	CD8+ T cells	*LOC103350037*	0.7376	0.273	0.009	6.080e-157	1.404e-152
11	CD8+ T cells	*ZNF831*	0.7364	0.288	0.008	1.189e-192	2.745e-188
11	CD8+ T cells	*ATXN7*	0.7343	0.508	0.222	5.357e-16	1.237e-11
11	CD8+ T cells	*PRKCB*	0.7339	0.326	0.017	4.419e-132	1.020e-127
11	CD8+ T cells	*LOC138850734*	0.7321	0.303	0.038	1.924e-52	4.443e-48
11	CD8+ T cells	*LPIN1*	0.7321	0.303	0.048	4.333e-40	1.001e-35
11	CD8+ T cells	*OTULINL*	0.7316	0.470	0.107	1.122e-37	2.591e-33
11	CD8+ T cells	*MBP*	0.7296	0.364	0.020	1.107e-136	2.557e-132
11	CD8+ T cells	*STIM1*	0.7295	0.485	0.142	2.467e-27	5.696e-23
11	CD8+ T cells	*LOC100346285*	0.7293	0.106	0.000	1.592e-174	3.677e-170
11	CD8+ T cells	*NFKBID*	0.7289	0.265	0.025	2.400e-61	5.543e-57
11	CD8+ T cells	*NCOA1*	0.7270	0.727	0.455	1.971e-13	4.553e-09
11	CD8+ T cells	*TES*	0.7268	0.364	0.088	5.809e-27	1.341e-22
11	CD8+ T cells	*CD6*	0.7267	0.227	0.000	0	0
11	CD8+ T cells	*RAB27A*	0.7266	0.371	0.055	1.326e-52	3.063e-48
11	CD8+ T cells	*MAPRE1*	0.7265	0.652	0.282	1.025e-20	2.368e-16
11	CD8+ T cells	*LOC127489744*	0.7264	0.591	0.188	3.599e-29	8.312e-25
11	CD8+ T cells	*IRAG2*	0.7263	0.341	0.015	4.301e-153	9.931e-149
11	CD8+ T cells	*RETREG1*	0.7260	0.409	0.093	1.906e-33	4.400e-29

Original cluster	Annotated cell type	Gene	avg_log2FC	pct.1	pct.2	Raw P value	Bonferroni-adjusted P value
11	CD8+ T cells	*RPS19*	0.7255	0.985	0.985	1.020e-30	2.356e-26
11	CD8+ T cells	*AHI1*	0.7254	0.606	0.310	2.432e-14	5.616e-10
11	CD8+ T cells	*HNRNPAB*	0.7249	0.780	0.417	9.413e-19	2.174e-14
11	CD8+ T cells	*RPS23*	0.7249	0.977	0.985	1.071e-37	2.473e-33
11	CD8+ T cells	*IGFLR1*	0.7231	0.295	0.011	5.646e-160	1.304e-155
11	CD8+ T cells	*SUB1*	0.7217	0.902	0.561	2.596e-20	5.996e-16
11	CD8+ T cells	*SERP1*	0.7214	0.788	0.426	3.845e-17	8.878e-13
11	CD8+ T cells	*LOC103348182*	0.7195	0.326	0.037	2.054e-61	4.743e-57
11	CD8+ T cells	*LOC100350325*	0.7194	0.386	0.096	4.896e-28	1.131e-23
11	CD8+ T cells	*LOC100357801*	0.7191	0.227	0.035	1.263e-30	2.917e-26
11	CD8+ T cells	*ASCC3*	0.7175	0.553	0.248	5.989e-16	1.383e-11
11	CD8+ T cells	*MYBL1*	0.7171	0.197	0.012	1.081e-69	2.497e-65
11	CD8+ T cells	*LOC100349257*	0.7159	0.295	0.022	9.801e-85	2.263e-80
11	CD8+ T cells	*GPBP1L1*	0.7154	0.629	0.269	7.174e-20	1.657e-15
11	CD8+ T cells	*CBFB*	0.7149	0.424	0.112	3.847e-28	8.884e-24
11	CD8+ T cells	*LOC100351569*	0.7148	0.561	0.239	1.386e-17	3.200e-13
11	CD8+ T cells	*STT3B*	0.7135	0.530	0.228	5.428e-16	1.253e-11
11	CD8+ T cells	*COX6A1*	0.7110	0.871	0.568	2.947e-17	6.806e-13
11	CD8+ T cells	*PRKAR1A*	0.7092	0.848	0.507	1.639e-17	3.784e-13
11	CD8+ T cells	*BZW1*	0.7085	0.621	0.282	7.563e-19	1.747e-14
11	CD8+ T cells	*RUNX1*	0.7071	0.295	0.040	2.079e-46	4.801e-42
11	CD8+ T cells	*LOC138847548*	0.7070	0.341	0.008	8.270e-255	1.910e-250
11	CD8+ T cells	*LOC138849409*	0.7067	0.265	0.084	6.059e-14	1.399e-09
11	CD8+ T cells	*CSNK1G3*	0.7056	0.538	0.206	2.834e-20	6.543e-16
11	CD8+ T cells	*CCDC50*	0.7056	0.682	0.350	1.890e-15	4.364e-11
11	CD8+ T cells	*IPCEF1*	0.7052	0.311	0.043	5.758e-48	1.330e-43
11	CD8+ T cells	*VPS13D*	0.7040	0.455	0.165	7.954e-19	1.837e-14
11	CD8+ T cells	*AARS1*	0.7038	0.386	0.103	1.418e-25	3.275e-21
11	CD8+ T cells	*ANKRD13C*	0.7035	0.500	0.199	7.954e-17	1.837e-12
11	CD8+ T cells	*MOB1A*	0.7033	0.508	0.112	1.954e-42	4.512e-38
11	CD8+ T cells	*AKR1B1*	0.7032	0.326	0.050	1.627e-43	3.757e-39
11	CD8+ T cells	*FGD3*	0.7028	0.326	0.013	4.930e-168	1.138e-163
11	CD8+ T cells	*UBE2J1*	0.6984	0.447	0.117	2.204e-29	5.089e-25
11	CD8+ T cells	*ZNF683*	0.6978	0.265	0.000	0	0
11	CD8+ T cells	*SIT1*	0.6965	0.295	0.000	0	0
11	CD8+ T cells	*KPNA2*	0.6958	0.280	0.044	1.868e-36	4.313e-32
11	CD8+ T cells	*ST3GAL6*	0.6935	0.409	0.103	1.073e-28	2.478e-24
11	CD8+ T cells	*MNDA*	0.6928	0.682	0.323	2.488e-17	5.745e-13
11	CD8+ T cells	*EMP3*	0.6925	0.765	0.464	1.933e-13	4.464e-09
11	CD8+ T cells	*KCNAB2*	0.6921	0.348	0.024	4.912e-107	1.134e-102

Original cluster	Annotated cell type	Gene	avg_log2FC	pct.1	pct.2	Raw P value	Bonferroni-adjusted P value
11	CD8+ T cells	*RCOR1*	0.6917	0.591	0.273	8.968e-16	2.071e-11
11	CD8+ T cells	*CNTRL*	0.6913	0.523	0.225	9.852e-16	2.275e-11
11	CD8+ T cells	*CNN2*	0.6895	0.402	0.087	8.996e-34	2.077e-29
11	CD8+ T cells	*EGR3*	0.6881	0.242	0.031	6.409e-40	1.480e-35
11	CD8+ T cells	*SIK2*	0.6877	0.614	0.359	1.718e-10	3.966e-06
11	CD8+ T cells	*RAP1B*	0.6867	0.545	0.187	1.603e-24	3.702e-20
11	CD8+ T cells	*RIPOR1*	0.6866	0.333	0.054	2.631e-42	6.074e-38
11	CD8+ T cells	*NEK7*	0.6859	0.621	0.239	1.590e-21	3.671e-17
11	CD8+ T cells	*SLC9A7*	0.6852	0.356	0.073	6.745e-33	1.558e-28
11	CD8+ T cells	*AP3M2*	0.6851	0.432	0.127	2.066e-24	4.770e-20
11	CD8+ T cells	*HSPA4*	0.6828	0.591	0.205	3.088e-25	7.131e-21
11	CD8+ T cells	*ATXN1*	0.6811	0.765	0.517	3.523e-10	8.136e-06
11	CD8+ T cells	*SRI*	0.6805	0.576	0.235	1.192e-18	2.752e-14
11	CD8+ T cells	*ARPC4*	0.6804	0.515	0.162	9.806e-26	2.264e-21
11	CD8+ T cells	*USP3*	0.6803	0.485	0.173	5.387e-20	1.244e-15
11	CD8+ T cells	*BATF*	0.6795	0.212	0.006	7.817e-141	1.805e-136
11	CD8+ T cells	*PTPN2*	0.6791	0.439	0.146	8.821e-21	2.037e-16
11	CD8+ T cells	*ROCK1*	0.6783	0.795	0.423	7.146e-18	1.650e-13
11	CD8+ T cells	*KMT2C*	0.6774	0.765	0.473	2.780e-14	6.419e-10
11	CD8+ T cells	*VPS13A*	0.6771	0.515	0.189	5.493e-20	1.268e-15
11	CD8+ T cells	*RPS29*	0.6769	0.977	0.977	6.232e-34	1.439e-29
11	CD8+ T cells	*FAM174A*	0.6765	0.515	0.188	1.278e-21	2.952e-17
11	CD8+ T cells	*MCUB*	0.6763	0.583	0.258	1.805e-16	4.167e-12
11	CD8+ T cells	*GRK6*	0.6760	0.341	0.048	1.281e-50	2.957e-46
11	CD8+ T cells	*LRRFIP1*	0.6752	0.538	0.200	8.566e-21	1.978e-16
11	CD8+ T cells	*CHST3*	0.6741	0.311	0.025	6.030e-83	1.392e-78
11	CD8+ T cells	*CD40LG*	0.6702	0.129	0.000	8.066e-258	1.863e-253
11	CD8+ T cells	*ZBTB38*	0.6697	0.394	0.164	2.513e-12	5.803e-08
11	CD8+ T cells	*LRRC8D*	0.6696	0.311	0.066	1.740e-28	4.019e-24
11	CD8+ T cells	*GLCE*	0.6691	0.439	0.121	8.217e-27	1.898e-22
11	CD8+ T cells	*LOC100341181*	0.6678	0.545	0.159	2.824e-29	6.522e-25
11	CD8+ T cells	*MYO1F*	0.6675	0.318	0.014	2.476e-148	5.717e-144
11	CD8+ T cells	*COMMD6*	0.6672	0.508	0.173	1.614e-22	3.727e-18
11	CD8+ T cells	*SIRT7*	0.6662	0.348	0.057	1.085e-43	2.506e-39
11	CD8+ T cells	*AGFG1*	0.6656	0.545	0.231	7.085e-17	1.636e-12
11	CD8+ T cells	*COG5*	0.6646	0.545	0.262	5.977e-13	1.380e-08
11	CD8+ T cells	*GMDS*	0.6633	0.424	0.150	4.426e-18	1.022e-13
11	CD8+ T cells	*SMAP2*	0.6632	0.409	0.060	2.049e-57	4.733e-53
11	CD8+ T cells	*ZNRF2*	0.6631	0.417	0.104	3.060e-29	7.067e-25
11	CD8+ T cells	*NLRC3*	0.6621	0.295	0.002	0	0

Original cluster	Annotated cell type	Gene	avg_log2FC	pct.1	pct.2	Raw P value	Bonferroni-adjusted P value
11	CD8+ T cells	*GPD2*	0.6620	0.341	0.070	7.879e-33	1.819e-28
11	CD8+ T cells	*PPP2R5E*	0.6616	0.636	0.296	8.126e-16	1.876e-11
11	CD8+ T cells	*PLXNC1*	0.6613	0.273	0.018	6.681e-87	1.543e-82
11	CD8+ T cells	*MRPL1*	0.6611	0.462	0.187	1.149e-15	2.654e-11
11	CD8+ T cells	*MYO1G*	0.6610	0.265	0.006	1.403e-202	3.240e-198
11	CD8+ T cells	*TMC6*	0.6605	0.295	0.016	8.966e-118	2.070e-113
11	CD8+ T cells	*AVPI1*	0.6593	0.439	0.124	6.685e-26	1.544e-21
11	CD8+ T cells	*JUND*	0.6584	0.992	0.957	2.193e-17	5.063e-13
11	CD8+ T cells	*USP25*	0.6578	0.515	0.255	3.552e-11	8.201e-07
11	CD8+ T cells	*CAPG*	0.6568	0.333	0.032	3.541e-75	8.177e-71
11	CD8+ T cells	*CAMK2G*	0.6551	0.386	0.122	5.812e-20	1.342e-15
11	CD8+ T cells	*CAMK1D*	0.6549	0.303	0.068	2.540e-26	5.864e-22
11	CD8+ T cells	*KIF21B*	0.6546	0.318	0.006	4.075e-259	9.409e-255
11	CD8+ T cells	*NHERF1*	0.6545	0.333	0.025	4.036e-95	9.320e-91
11	CD8+ T cells	*SELL*	0.6543	0.174	0.007	1.185e-85	2.736e-81
11	CD8+ T cells	*LOC138850522*	0.6533	0.197	0.009	3.677e-86	8.491e-82
11	CD8+ T cells	*LOC108177586*	0.6528	0.250	0.001	0	0
11	CD8+ T cells	*MCTP2*	0.6524	0.333	0.047	9.469e-49	2.187e-44
11	CD8+ T cells	*CASS4*	0.6508	0.242	0.002	1.089e-299	2.514e-295
11	CD8+ T cells	*ARRDC4*	0.6493	0.258	0.054	3.990e-24	9.213e-20
11	CD8+ T cells	*DDX5*	0.6491	1.000	0.895	5.929e-21	1.369e-16
11	CD8+ T cells	*SLA2*	0.6478	0.311	0.009	3.205e-199	7.400e-195
11	CD8+ T cells	*LOC100349347*	0.6473	0.394	0.084	6.420e-35	1.482e-30
11	CD8+ T cells	*HELZ*	0.6462	0.576	0.300	4.188e-12	9.671e-08
11	CD8+ T cells	*CDC42SE1*	0.6462	0.492	0.157	3.907e-24	9.022e-20
11	CD8+ T cells	*AKIRIN1*	0.6461	0.424	0.087	2.286e-39	5.279e-35
11	CD8+ T cells	*DHRS11*	0.6454	0.364	0.062	4.273e-43	9.867e-39
11	CD8+ T cells	*LOC100345060*	0.6451	0.311	0.039	3.385e-53	7.817e-49
11	CD8+ T cells	*UBXN7*	0.6450	0.477	0.207	5.981e-13	1.381e-08
11	CD8+ T cells	*IRF7*	0.6444	0.189	0.068	4.956e-08	1.144e-03
11	CD8+ T cells	*PDLIM2*	0.6438	0.417	0.076	4.191e-45	9.677e-41
11	CD8+ T cells	*GRK2*	0.6434	0.402	0.086	2.125e-35	4.906e-31
11	CD8+ T cells	*ZZEF1*	0.6433	0.348	0.099	3.258e-21	7.524e-17
11	CD8+ T cells	*LOC100352672*	0.6432	0.242	0.005	4.544e-193	1.049e-188
11	CD8+ T cells	*LOC138844224*	0.6418	0.174	0.022	3.223e-29	7.444e-25
11	CD8+ T cells	*IRF2*	0.6412	0.652	0.280	6.737e-19	1.556e-14
11	CD8+ T cells	*NABP1*	0.6406	0.500	0.210	1.690e-15	3.903e-11
11	CD8+ T cells	*YWHAZ*	0.6402	0.932	0.642	2.387e-15	5.512e-11
11	CD8+ T cells	*HIVEP1*	0.6389	0.356	0.094	4.930e-24	1.139e-19
11	CD8+ T cells	*DEGS1*	0.6388	0.530	0.172	9.960e-25	2.300e-20

Original cluster	Annotated cell type	Gene	avg_log2FC	pct.1	pct.2	Raw P value	Bonferroni-adjusted P value
11	CD8+ T cells	*HOMER1*	0.6376	0.477	0.238	3.038e-09	7.016e-05
11	CD8+ T cells	*SGK1*	0.6371	0.379	0.160	2.357e-11	5.443e-07
11	CD8+ T cells	*NUP50*	0.6370	0.432	0.099	6.859e-34	1.584e-29
11	CD8+ T cells	*PIK3CD*	0.6355	0.280	0.006	2.212e-220	5.109e-216
11	CD8+ T cells	*GNA15*	0.6342	0.235	0.004	6.268e-203	1.447e-198
11	CD8+ T cells	*LOC138845055*	0.6342	0.235	0.000	0	0
11	CD8+ T cells	*CFLAR*	0.6342	0.402	0.113	9.627e-25	2.223e-20
11	CD8+ T cells	*WNK1*	0.6334	0.636	0.336	5.783e-12	1.335e-07
11	CD8+ T cells	*TAB2*	0.6316	0.561	0.254	1.951e-14	4.505e-10
11	CD8+ T cells	*PAFAH1B1*	0.6315	0.750	0.409	2.719e-15	6.279e-11
11	CD8+ T cells	*RECQL*	0.6313	0.455	0.168	1.383e-17	3.195e-13
11	CD8+ T cells	*BOLA2B*	0.6292	0.644	0.299	2.720e-16	6.281e-12
11	CD8+ T cells	*TBC1D2B*	0.6284	0.326	0.074	2.040e-26	4.711e-22
11	CD8+ T cells	*STIM2*	0.6275	0.439	0.143	3.771e-20	8.709e-16
11	CD8+ T cells	*LOC100349247*	0.6268	0.318	0.010	4.099e-194	9.467e-190
11	CD8+ T cells	*HDAC8*	0.6254	0.492	0.192	1.840e-16	4.249e-12
11	CD8+ T cells	*CAST*	0.6247	0.583	0.260	1.991e-15	4.598e-11
11	CD8+ T cells	*SPTY2D1*	0.6241	0.515	0.199	2.795e-18	6.454e-14
11	CD8+ T cells	*FBXL17*	0.6222	0.606	0.326	2.948e-11	6.808e-07
11	CD8+ T cells	*PHTF2*	0.6217	0.326	0.112	3.260e-14	7.527e-10
11	CD8+ T cells	*PHACTR2*	0.6216	0.500	0.268	3.620e-09	8.360e-05
11	CD8+ T cells	*CDK6*	0.6208	0.561	0.301	1.765e-10	4.075e-06
11	CD8+ T cells	*ZCCHC2*	0.6192	0.356	0.101	3.141e-21	7.253e-17
11	CD8+ T cells	*SYTL1*	0.6190	0.318	0.005	9.075e-293	2.096e-288
11	CD8+ T cells	*KIAA0040*	0.6189	0.303	0.034	8.357e-58	1.930e-53
11	CD8+ T cells	*NLRC5*	0.6168	0.273	0.007	8.917e-181	2.059e-176
11	CD8+ T cells	*RRM2B*	0.6168	0.333	0.060	3.647e-37	8.422e-33
11	CD8+ T cells	*WDR77*	0.6164	0.311	0.057	3.414e-33	7.883e-29
11	CD8+ T cells	*ARHGAP18*	0.6153	0.364	0.091	1.010e-25	2.333e-21
11	CD8+ T cells	*EIF3A*	0.6149	0.614	0.311	1.607e-13	3.710e-09
11	CD8+ T cells	*CLINT1*	0.6146	0.583	0.257	2.015e-15	4.653e-11
11	CD8+ T cells	*THRAP3*	0.6134	0.758	0.427	3.789e-15	8.748e-11
11	CD8+ T cells	*TSPYL1*	0.6133	0.417	0.191	3.620e-11	8.359e-07
11	CD8+ T cells	*MBD2*	0.6128	0.485	0.181	9.861e-18	2.277e-13
11	CD8+ T cells	*NSD3*	0.6120	0.758	0.506	4.223e-11	9.751e-07
11	CD8+ T cells	*SFMBT1*	0.6113	0.318	0.051	1.794e-40	4.144e-36
11	CD8+ T cells	*PSEN2*	0.6108	0.515	0.177	2.320e-21	5.357e-17
11	CD8+ T cells	*TPST2*	0.6104	0.379	0.105	2.304e-23	5.320e-19
11	CD8+ T cells	*EGLN3*	0.6102	0.242	0.035	1.358e-35	3.136e-31
11	CD8+ T cells	*LOC100355171*	0.6095	0.250	0.030	2.750e-44	6.350e-40

Original cluster	Annotated cell type	Gene	avg_log2FC	pct.1	pct.2	Raw P value	Bonferroni-adjusted P value
11	CD8+ T cells	*LOC103350898*	0.6093	0.356	0.074	1.027e-31	2.372e-27
11	CD8+ T cells	*GPR162*	0.6091	0.288	0.031	2.920e-57	6.742e-53
11	CD8+ T cells	*RGS14*	0.6088	0.235	0.022	1.564e-54	3.612e-50
11	CD8+ T cells	*RPL36*	0.6074	0.977	0.971	1.022e-24	2.359e-20
11	CD8+ T cells	*CHD1*	0.6070	0.598	0.280	6.941e-14	1.603e-09
11	CD8+ T cells	*LOC108175891*	0.6058	0.409	0.139	2.051e-18	4.737e-14
11	CD8+ T cells	*HES6*	0.6057	0.250	0.014	1.264e-92	2.919e-88
11	CD8+ T cells	*PPP1CA*	0.6055	0.598	0.232	3.031e-20	6.998e-16
11	CD8+ T cells	*CRYBG1*	0.6046	0.356	0.082	2.042e-27	4.714e-23
11	CD8+ T cells	*ZNF654*	0.6045	0.636	0.332	3.993e-12	9.220e-08
11	CD8+ T cells	*SNX14*	0.6025	0.447	0.206	1.258e-11	2.905e-07
11	CD8+ T cells	*EHBP1L1*	0.6021	0.273	0.023	9.049e-69	2.090e-64
11	CD8+ T cells	*USP51*	0.6021	0.280	0.104	8.515e-11	1.966e-06
11	CD8+ T cells	*PKN1*	0.6017	0.341	0.082	5.187e-26	1.198e-21
11	CD8+ T cells	*EIF5A*	0.6006	0.864	0.577	4.497e-14	1.038e-09
11	CD8+ T cells	*TSGA10*	0.5993	0.402	0.093	5.174e-31	1.195e-26
11	CD8+ T cells	*PIK3CG*	0.5985	0.273	0.010	1.841e-141	4.251e-137
11	CD8+ T cells	*UQCR11*	0.5971	0.606	0.294	1.386e-13	3.201e-09
11	CD8+ T cells	*CD79A*	0.5961	0.242	0.005	1.089e-197	2.514e-193
11	CD8+ T cells	*ATP6V1F*	0.5951	0.455	0.146	1.090e-21	2.516e-17
11	CD8+ T cells	*RAB2A*	0.5949	0.780	0.486	2.349e-11	5.425e-07
11	CD8+ T cells	*RAB8B*	0.5945	0.621	0.303	3.086e-14	7.127e-10
11	CD8+ T cells	*NSF*	0.5941	0.356	0.087	6.017e-26	1.389e-21
11	CD8+ T cells	*IPMK*	0.5940	0.258	0.072	8.236e-16	1.902e-11
11	CD8+ T cells	*RAB11FIP1*	0.5931	0.424	0.145	2.319e-18	5.355e-14
11	CD8+ T cells	*EPSTI1*	0.5930	0.265	0.053	2.129e-25	4.917e-21
11	CD8+ T cells	*PPP4C*	0.5922	0.553	0.236	5.259e-16	1.214e-11
11	CD8+ T cells	*RASGEF1B*	0.5920	0.311	0.134	5.722e-09	1.321e-04
11	CD8+ T cells	*ZNF644*	0.5919	0.682	0.502	1.693e-06	3.910e-02
11	CD8+ T cells	*CD4*	0.5895	0.159	0.002	2.605e-152	6.014e-148
11	CD8+ T cells	*DCAF10*	0.5887	0.295	0.072	4.216e-22	9.735e-18
11	CD8+ T cells	*FKBP11*	0.5885	0.402	0.206	5.828e-08	1.346e-03
11	CD8+ T cells	*NCOA3*	0.5883	0.394	0.120	5.338e-21	1.233e-16
11	CD8+ T cells	*TLK1*	0.5883	0.530	0.281	2.993e-11	6.912e-07
11	CD8+ T cells	*LOC100358539*	0.5875	0.235	0.015	1.144e-80	2.642e-76
11	CD8+ T cells	*TBX21*	0.5860	0.212	0.001	0	0
11	CD8+ T cells	*MKNK2*	0.5851	0.447	0.185	1.789e-13	4.130e-09
11	CD8+ T cells	*TGOLN2*	0.5851	0.621	0.297	8.128e-15	1.877e-10
11	CD8+ T cells	*MAT2B*	0.5845	0.598	0.269	1.873e-15	4.326e-11
11	CD8+ T cells	*RHOF*	0.5842	0.295	0.010	2.344e-170	5.413e-166

Original cluster	Annotated cell type	Gene	avg_log2FC	pct.1	pct.2	Raw P value	Bonferroni-adjusted P value
11	CD8+ T cells	*ACAP1*	0.5839	0.242	0.009	3.089e-132	7.133e-128
11	CD8+ T cells	*SLAIN1*	0.5833	0.265	0.016	7.970e-91	1.841e-86
11	CD8+ T cells	*USP15*	0.5822	0.644	0.347	1.766e-11	4.078e-07
11	CD8+ T cells	*FYCO1*	0.5820	0.295	0.107	3.064e-12	7.076e-08
11	CD8+ T cells	*EXOC6*	0.5814	0.333	0.103	1.032e-17	2.382e-13
11	CD8+ T cells	*SC5D*	0.5807	0.273	0.083	5.862e-15	1.354e-10
11	CD8+ T cells	*TRAPPC1*	0.5804	0.409	0.134	5.144e-19	1.188e-14
11	CD8+ T cells	*PITPNA*	0.5793	0.424	0.142	4.323e-19	9.982e-15
11	CD8+ T cells	*JAK3*	0.5788	0.265	0.015	5.055e-98	1.167e-93
11	CD8+ T cells	*TMSB15A*	0.5788	0.811	0.487	3.992e-13	9.218e-09
11	CD8+ T cells	*UQCRFS1*	0.5786	0.598	0.275	2.099e-15	4.846e-11
11	CD8+ T cells	*RPS26*	0.5782	0.985	0.985	7.339e-25	1.695e-20
11	CD8+ T cells	*TRAF5*	0.5774	0.318	0.046	5.171e-45	1.194e-40
11	CD8+ T cells	*XRN2*	0.5773	0.598	0.265	3.316e-16	7.656e-12
11	CD8+ T cells	*TAF15*	0.5764	0.568	0.281	4.009e-12	9.258e-08
11	CD8+ T cells	*RASA1*	0.5761	0.508	0.293	1.650e-08	3.809e-04
11	CD8+ T cells	*TWF2*	0.5753	0.379	0.107	2.739e-22	6.326e-18
11	CD8+ T cells	*LOC103352098*	0.5747	0.242	0.001	0	0
11	CD8+ T cells	*TRERF1*	0.5734	0.364	0.140	6.532e-13	1.508e-08
11	CD8+ T cells	*LOC138848553*	0.5732	0.295	0.043	5.946e-42	1.373e-37
11	CD8+ T cells	*UBR4*	0.5731	0.417	0.187	2.892e-11	6.679e-07
11	CD8+ T cells	*SINHCAF*	0.5719	0.318	0.047	1.064e-44	2.456e-40
11	CD8+ T cells	*ATP2B1*	0.5718	0.583	0.403	5.671e-07	1.309e-02
11	CD8+ T cells	*RNF115*	0.5702	0.500	0.200	1.311e-16	3.027e-12
11	CD8+ T cells	*SEL1L3*	0.5699	0.258	0.010	4.051e-128	9.355e-124
11	CD8+ T cells	*SPCS1*	0.5698	0.644	0.323	3.313e-13	7.649e-09
11	CD8+ T cells	*RAPGEF6*	0.5683	0.576	0.275	5.484e-14	1.266e-09
11	CD8+ T cells	*ITGAL*	0.5682	0.220	0.007	8.749e-125	2.020e-120
11	CD8+ T cells	*CMPK1*	0.5678	0.424	0.145	5.291e-18	1.222e-13
11	CD8+ T cells	*TMCO4*	0.5664	0.250	0.023	1.997e-58	4.610e-54
11	CD8+ T cells	*XPA*	0.5663	0.462	0.144	1.584e-22	3.658e-18
11	CD8+ T cells	*PSMB10*	0.5658	0.364	0.094	3.613e-24	8.344e-20
11	CD8+ T cells	*LOC138844967*	0.5654	0.242	0.044	1.794e-26	4.142e-22
11	CD8+ T cells	*TRIP12*	0.5649	0.500	0.209	4.559e-14	1.053e-09
11	CD8+ T cells	*PELI1*	0.5646	0.485	0.248	1.618e-09	3.736e-05
11	CD8+ T cells	*LDLRAD4*	0.5643	0.500	0.280	1.528e-08	3.529e-04
11	CD8+ T cells	*PTPN6*	0.5641	0.265	0.025	1.383e-60	3.194e-56
11	CD8+ T cells	*IRAK2*	0.5626	0.402	0.112	2.380e-23	5.497e-19
11	CD8+ T cells	*ENPP4*	0.5623	0.303	0.047	3.027e-39	6.990e-35
11	CD8+ T cells	*UNC13D*	0.5620	0.265	0.004	1.885e-255	4.353e-251

Original cluster	Annotated cell type	Gene	avg_log2FC	pct.1	pct.2	Raw P value	Bonferroni-adjusted P value
11	CD8+ T cells	*NLRP2*	0.5601	0.189	0.002	1.479e-202	3.415e-198
11	CD8+ T cells	*DERL1*	0.5586	0.439	0.163	2.025e-16	4.675e-12
11	CD8+ T cells	*MIA2*	0.5581	0.379	0.090	5.515e-28	1.273e-23
11	CD8+ T cells	*LOC100358013*	0.5575	0.356	0.102	1.255e-20	2.897e-16
11	CD8+ T cells	*MARCHF8*	0.5572	0.409	0.144	1.034e-16	2.389e-12
11	CD8+ T cells	*TNFRSF9*	0.5570	0.144	0.016	3.894e-29	8.993e-25
11	CD8+ T cells	*TMC8*	0.5567	0.242	0.007	4.126e-162	9.528e-158
11	CD8+ T cells	*EBP*	0.5549	0.447	0.126	1.021e-24	2.357e-20
11	CD8+ T cells	*USP34*	0.5547	0.742	0.437	7.384e-12	1.705e-07
11	CD8+ T cells	*LYSET*	0.5537	0.341	0.083	1.320e-24	3.047e-20
11	CD8+ T cells	*RPLP1*	0.5536	0.992	0.991	7.382e-22	1.705e-17
11	CD8+ T cells	*PTPN1*	0.5535	0.348	0.075	1.524e-30	3.519e-26
11	CD8+ T cells	*SAMD9L*	0.5534	0.356	0.096	3.578e-22	8.262e-18
11	CD8+ T cells	*CTSS*	0.5533	0.311	0.038	1.386e-54	3.201e-50
11	CD8+ T cells	*SPPL2A*	0.5530	0.485	0.204	6.535e-14	1.509e-09
11	CD8+ T cells	*BTG1*	0.5526	0.917	0.661	9.504e-12	2.195e-07
11	CD8+ T cells	*AK5*	0.5525	0.136	0.005	6.193e-72	1.430e-67
11	CD8+ T cells	*FBXO33*	0.5525	0.470	0.211	1.661e-12	3.836e-08
11	CD8+ T cells	*ARB2A*	0.5524	0.750	0.552	7.833e-07	1.809e-02
11	CD8+ T cells	*DPF3*	0.5517	0.235	0.027	8.624e-44	1.991e-39
11	CD8+ T cells	*PTPN18*	0.5499	0.273	0.004	1.029e-259	2.376e-255
11	CD8+ T cells	*MCL1*	0.5498	0.727	0.428	4.628e-11	1.069e-06
11	CD8+ T cells	*EFR3A*	0.5498	0.492	0.240	9.485e-12	2.190e-07
11	CD8+ T cells	*NAPSA*	0.5497	0.227	0.005	3.101e-181	7.162e-177
11	CD8+ T cells	*ACOXL*	0.5495	0.182	0.006	8.289e-100	1.914e-95
11	CD8+ T cells	*DEF6*	0.5488	0.235	0.013	3.608e-91	8.331e-87
11	CD8+ T cells	*FKBP5*	0.5473	0.205	0.036	9.559e-24	2.207e-19
11	CD8+ T cells	*LPXN*	0.5472	0.258	0.016	4.069e-89	9.397e-85
11	CD8+ T cells	*RPL36A*	0.5463	0.985	0.946	1.427e-18	3.295e-14
11	CD8+ T cells	*TMED5*	0.5463	0.424	0.212	4.588e-09	1.059e-04
11	CD8+ T cells	*HPRT1*	0.5460	0.311	0.060	1.258e-31	2.905e-27
11	CD8+ T cells	*CD80*	0.5460	0.212	0.009	5.875e-99	1.357e-94
11	CD8+ T cells	*REEP5*	0.5459	0.583	0.302	1.067e-11	2.463e-07
11	CD8+ T cells	*SMDT1*	0.5446	0.485	0.186	2.932e-17	6.770e-13
11	CD8+ T cells	*PARVG*	0.5445	0.227	0.006	4.605e-154	1.063e-149
11	CD8+ T cells	*YY1*	0.5443	0.530	0.282	5.179e-10	1.196e-05
11	CD8+ T cells	*PPIA*	0.5438	0.773	0.475	3.363e-12	7.765e-08
11	CD8+ T cells	*SLC7A6*	0.5434	0.265	0.024	4.229e-62	9.766e-58
11	CD8+ T cells	*VRK1*	0.5429	0.303	0.058	2.190e-30	5.057e-26
11	CD8+ T cells	*IKBKE*	0.5427	0.242	0.018	1.248e-70	2.883e-66

Original cluster	Annotated cell type	Gene	avg_log2FC	pct.1	pct.2	Raw P value	Bonferroni-adjusted P value
11	CD8+ T cells	*PDCD10*	0.5422	0.508	0.226	5.876e-13	1.357e-08
11	CD8+ T cells	*FOXO3*	0.5418	0.561	0.328	3.568e-07	8.240e-03
11	CD8+ T cells	*LPP*	0.5417	0.636	0.376	8.388e-10	1.937e-05
11	CD8+ T cells	*CCNI*	0.5412	0.568	0.266	1.362e-13	3.146e-09
11	CD8+ T cells	*LEPROTL1*	0.5402	0.364	0.126	2.433e-15	5.618e-11
11	CD8+ T cells	*RIN3*	0.5401	0.258	0.020	2.087e-72	4.818e-68
11	CD8+ T cells	*TBC1D1*	0.5401	0.333	0.084	2.964e-23	6.844e-19
11	CD8+ T cells	*MAN1A2*	0.5399	0.424	0.179	9.330e-13	2.155e-08
11	CD8+ T cells	*TRAPPC8*	0.5365	0.432	0.197	1.148e-10	2.650e-06
11	CD8+ T cells	*VCPKMT*	0.5362	0.303	0.050	6.498e-37	1.501e-32
11	CD8+ T cells	*PRELID1*	0.5355	0.485	0.209	8.172e-14	1.887e-09
11	CD8+ T cells	*CPD*	0.5350	0.545	0.282	4.320e-10	9.977e-06
11	CD8+ T cells	*LOC108177422*	0.5348	0.530	0.236	2.002e-14	4.624e-10
11	CD8+ T cells	*ATP5PD*	0.5338	0.902	0.653	8.654e-14	1.998e-09
11	CD8+ T cells	*LOC127491011*	0.5335	0.250	0.059	5.729e-19	1.323e-14
11	CD8+ T cells	*IL27RA*	0.5314	0.265	0.009	3.736e-151	8.626e-147
11	CD8+ T cells	*RBPJ*	0.5307	0.659	0.458	4.944e-07	1.142e-02
11	CD8+ T cells	*RPL35*	0.5300	0.985	0.988	1.136e-23	2.623e-19
11	CD8+ T cells	*RFTN1*	0.5293	0.386	0.143	2.618e-14	6.044e-10
11	CD8+ T cells	*PTP4A2*	0.5282	0.629	0.327	1.477e-12	3.412e-08
11	CD8+ T cells	*GIT2*	0.5281	0.379	0.120	1.350e-18	3.116e-14
11	CD8+ T cells	*C18H16orf74*	0.5272	0.242	0.012	5.921e-104	1.367e-99
11	CD8+ T cells	*TNFSF14*	0.5270	0.167	0.001	2.654e-270	6.129e-266
11	CD8+ T cells	*WDR26*	0.5270	0.523	0.215	8.084e-16	1.867e-11
11	CD8+ T cells	*PAN3*	0.5263	0.674	0.363	2.646e-12	6.109e-08
11	CD8+ T cells	*APAF1*	0.5259	0.561	0.248	2.506e-14	5.787e-10
11	CD8+ T cells	*ATP6V0D1*	0.5254	0.379	0.112	1.394e-20	3.219e-16
11	CD8+ T cells	*EVI2B*	0.5252	0.242	0.011	5.923e-113	1.368e-108
11	CD8+ T cells	*LOC138846506*	0.5233	0.280	0.053	2.671e-29	6.168e-25
11	CD8+ T cells	*LCOR*	0.5227	0.462	0.231	4.655e-10	1.075e-05
11	CD8+ T cells	*DNAJC3*	0.5226	0.583	0.294	8.499e-12	1.963e-07
11	CD8+ T cells	*VSIR*	0.5222	0.318	0.063	4.279e-31	9.882e-27
11	CD8+ T cells	*GRAP*	0.5222	0.258	0.008	2.685e-163	6.201e-159
11	CD8+ T cells	*ADCY7*	0.5203	0.227	0.006	2.369e-162	5.470e-158
11	CD8+ T cells	*LOC138845873*	0.5201	0.258	0.049	8.641e-26	1.995e-21
11	CD8+ T cells	*KPNA4*	0.5197	0.455	0.246	8.172e-08	1.887e-03
11	CD8+ T cells	*SOCS4*	0.5194	0.311	0.049	1.244e-39	2.873e-35
11	CD8+ T cells	*IMP3*	0.5186	0.326	0.105	4.695e-16	1.084e-11
11	CD8+ T cells	*GNG5*	0.5166	0.788	0.480	1.414e-10	3.265e-06
11	CD8+ T cells	*EEIG2*	0.5161	0.303	0.108	2.511e-12	5.798e-08

Original cluster	Annotated cell type	Gene	avg_log2FC	pct.1	pct.2	Raw P value	Bonferroni-adjusted P value
11	CD8+ T cells	*STAMBPL1*	0.5157	0.242	0.023	8.993e-55	2.077e-50
11	CD8+ T cells	*TNFSF8*	0.5153	0.167	0.001	8.620e-235	1.990e-230
11	CD8+ T cells	*ABI3*	0.5152	0.227	0.009	7.078e-119	1.635e-114
11	CD8+ T cells	*LASP1*	0.5147	0.508	0.240	1.802e-11	4.161e-07
11	CD8+ T cells	*EXOC4*	0.5141	0.667	0.428	2.893e-09	6.680e-05
11	CD8+ T cells	*FAM89B*	0.5138	0.598	0.310	3.258e-11	7.523e-07
11	CD8+ T cells	*PAICS*	0.5137	0.295	0.078	8.901e-19	2.055e-14
11	CD8+ T cells	*AP3B1*	0.5137	0.402	0.151	6.671e-15	1.540e-10
11	CD8+ T cells	*RALGAPB*	0.5132	0.273	0.068	1.879e-19	4.338e-15
11	CD8+ T cells	*CCDC12*	0.5131	0.394	0.112	2.817e-22	6.505e-18
11	CD8+ T cells	*LOC127487570*	0.5124	0.220	0.001	0	0
11	CD8+ T cells	*TMEM163*	0.5111	0.152	0.012	5.996e-41	1.385e-36
11	CD8+ T cells	*TMEM41B*	0.5099	0.394	0.125	5.942e-19	1.372e-14
11	CD8+ T cells	*PTP4A1*	0.5095	0.530	0.274	4.251e-09	9.817e-05
11	CD8+ T cells	*OSBPL6*	0.5095	0.303	0.093	2.335e-15	5.392e-11
11	CD8+ T cells	*EVI2A*	0.5092	0.197	0.007	1.659e-110	3.832e-106
11	CD8+ T cells	*RPL13A*	0.5091	0.992	0.987	6.355e-24	1.467e-19
11	CD8+ T cells	*YEATS2*	0.5088	0.303	0.071	1.861e-23	4.299e-19
11	CD8+ T cells	*TAP2*	0.5087	0.311	0.049	1.225e-39	2.828e-35
11	CD8+ T cells	*SRSF9*	0.5080	0.659	0.344	2.818e-13	6.508e-09
11	CD8+ T cells	*PRKAG2*	0.5075	0.515	0.204	6.796e-16	1.569e-11
11	CD8+ T cells	*AHCYL1*	0.5073	0.280	0.114	2.786e-09	6.434e-05
11	CD8+ T cells	*ARRB2*	0.5066	0.311	0.047	8.935e-41	2.063e-36
11	CD8+ T cells	*LOC138843760*	0.5059	0.136	0.000	2.604e-258	6.013e-254
11	CD8+ T cells	*OTUD3*	0.5057	0.258	0.032	1.918e-44	4.430e-40
11	CD8+ T cells	*RAB43*	0.5057	0.280	0.063	3.101e-23	7.162e-19
11	CD8+ T cells	*ELK3*	0.5053	0.356	0.117	4.242e-16	9.796e-12
11	CD8+ T cells	*GTF2F2*	0.5034	0.288	0.106	4.964e-11	1.146e-06
11	CD8+ T cells	*SNX25*	0.5031	0.280	0.063	6.953e-23	1.606e-18
11	CD8+ T cells	*MEMO1*	0.5028	0.326	0.109	1.338e-14	3.090e-10
11	CD8+ T cells	*ARHGEF18*	0.5028	0.273	0.045	5.431e-33	1.254e-28
11	CD8+ T cells	*RAB10*	0.5027	0.727	0.420	9.434e-10	2.178e-05
11	CD8+ T cells	*UXT*	0.5023	0.386	0.139	6.335e-15	1.463e-10
11	CD8+ T cells	*RBM38*	0.5018	0.242	0.014	4.777e-90	1.103e-85
11	CD8+ T cells	*LOC100340393*	0.5006	0.303	0.047	7.935e-40	1.832e-35
11	CD8+ T cells	*PARP14*	0.5006	0.220	0.074	3.353e-10	7.742e-06
11	CD8+ T cells	*ZNF326*	0.5005	0.750	0.503	1.166e-08	2.693e-04
11	CD8+ T cells	*CDK17*	0.4999	0.386	0.168	3.316e-10	7.657e-06
11	CD8+ T cells	*TTYH3*	0.4997	0.311	0.072	9.735e-24	2.248e-19
11	CD8+ T cells	*ZNF407*	0.4994	0.439	0.222	1.123e-08	2.594e-04

Original cluster	Annotated cell type	Gene	avg_log2FC	pct.1	pct.2	Raw P value	Bonferroni-adjusted P value
11	CD8+ T cells	*LOC127491977*	0.4990	0.197	0.000	0	0
11	CD8+ T cells	*CYRIA*	0.4988	0.280	0.061	3.236e-24	7.472e-20
11	CD8+ T cells	*FAS*	0.4982	0.205	0.053	5.740e-14	1.326e-09
11	CD8+ T cells	*DDX46*	0.4980	0.561	0.297	5.006e-10	1.156e-05
11	CD8+ T cells	*ZNF292*	0.4977	0.712	0.406	2.751e-10	6.352e-06
11	CD8+ T cells	*NFATC1*	0.4972	0.242	0.039	1.521e-30	3.511e-26
11	CD8+ T cells	*FLT3LG*	0.4969	0.280	0.032	2.888e-52	6.670e-48
11	CD8+ T cells	*MLLT6*	0.4958	0.311	0.086	4.986e-19	1.151e-14
11	CD8+ T cells	*C16H19orf25*	0.4958	0.402	0.128	1.109e-18	2.561e-14
11	CD8+ T cells	*ORMDL2*	0.4955	0.295	0.052	3.517e-33	8.122e-29
11	CD8+ T cells	*EIF4EBP2*	0.4954	0.788	0.544	2.133e-09	4.925e-05
11	CD8+ T cells	*G2E3*	0.4947	0.295	0.127	1.150e-08	2.655e-04
11	CD8+ T cells	*ANTXR2*	0.4946	0.705	0.469	2.002e-07	4.623e-03
11	CD8+ T cells	*TRIR*	0.4943	0.833	0.546	2.936e-11	6.779e-07
11	CD8+ T cells	*M6PR*	0.4937	0.530	0.277	3.532e-10	8.157e-06
11	CD8+ T cells	*ISCU*	0.4934	0.780	0.514	5.869e-10	1.355e-05
11	CD8+ T cells	*UBQLN1*	0.4930	0.439	0.197	3.306e-11	7.635e-07
11	CD8+ T cells	*MICAL1*	0.4928	0.235	0.017	8.773e-69	2.026e-64
11	CD8+ T cells	*USP12*	0.4927	0.409	0.178	5.089e-11	1.175e-06
11	CD8+ T cells	*UCHL5*	0.4927	0.386	0.137	2.652e-15	6.124e-11
11	CD8+ T cells	*TMEM258*	0.4923	0.705	0.424	3.002e-10	6.932e-06
11	CD8+ T cells	*COX5A*	0.4913	0.727	0.399	3.500e-11	8.082e-07
11	CD8+ T cells	*CEP350*	0.4905	0.500	0.249	1.708e-10	3.944e-06
11	CD8+ T cells	*HSD17B4*	0.4905	0.462	0.255	3.573e-07	8.251e-03
11	CD8+ T cells	*ANKRD11*	0.4896	0.402	0.194	1.814e-09	4.189e-05
11	CD8+ T cells	*RPL13*	0.4891	0.977	0.988	2.930e-22	6.766e-18
11	CD8+ T cells	*RPTOR*	0.4891	0.250	0.062	5.663e-18	1.308e-13
11	CD8+ T cells	*AMZ2*	0.4885	0.333	0.124	1.957e-12	4.518e-08
11	CD8+ T cells	*SLC20A1*	0.4882	0.303	0.136	3.402e-08	7.855e-04
11	CD8+ T cells	*TMEM123*	0.4881	0.492	0.218	1.826e-12	4.217e-08
11	CD8+ T cells	*IARS2*	0.4876	0.295	0.100	4.883e-13	1.128e-08
11	CD8+ T cells	*TDG*	0.4876	0.379	0.120	2.492e-18	5.754e-14
11	CD8+ T cells	*PI4K2B*	0.4876	0.212	0.027	8.176e-36	1.888e-31
11	CD8+ T cells	*TAP1*	0.4874	0.205	0.018	2.847e-50	6.575e-46
11	CD8+ T cells	*ARID1A*	0.4872	0.470	0.216	1.370e-11	3.163e-07
11	CD8+ T cells	*UIMC1*	0.4863	0.394	0.143	6.232e-15	1.439e-10
11	CD8+ T cells	*MORN1*	0.4859	0.197	0.018	1.657e-46	3.826e-42
11	CD8+ T cells	*ZFAND6*	0.4857	0.508	0.234	1.296e-11	2.992e-07
11	CD8+ T cells	*ABHD3*	0.4856	0.220	0.025	3.096e-41	7.149e-37
11	CD8+ T cells	*SLC36A4*	0.4852	0.432	0.162	1.442e-14	3.330e-10

Original cluster	Annotated cell type	Gene	avg_log2FC	pct.1	pct.2	Raw P value	Bonferroni-adjusted P value
11	CD8+ T cells	*OST4*	0.4847	0.856	0.624	1.807e-09	4.173e-05
11	CD8+ T cells	*TAPBPL*	0.4845	0.250	0.038	4.520e-34	1.044e-29
11	CD8+ T cells	*BTLA*	0.4840	0.152	0.003	1.905e-134	4.400e-130
11	CD8+ T cells	*LOC100345591*	0.4832	0.561	0.296	1.314e-09	3.033e-05
11	CD8+ T cells	*MKLN1*	0.4828	0.530	0.221	3.150e-15	7.274e-11
11	CD8+ T cells	*SNX10*	0.4825	0.220	0.027	9.203e-39	2.125e-34
11	CD8+ T cells	*PTPN12*	0.4822	0.402	0.228	1.020e-06	2.356e-02
11	CD8+ T cells	*MFSD6*	0.4821	0.227	0.030	1.171e-36	2.703e-32
11	CD8+ T cells	*SNURF*	0.4814	0.432	0.180	1.316e-12	3.040e-08
11	CD8+ T cells	*RNF44*	0.4814	0.273	0.078	4.967e-16	1.147e-11
11	CD8+ T cells	*TBC1D5*	0.4813	0.780	0.525	3.082e-08	7.117e-04
11	CD8+ T cells	*ACTN4*	0.4799	0.447	0.197	3.374e-11	7.790e-07
11	CD8+ T cells	*C16H19orf53*	0.4795	0.697	0.445	9.977e-10	2.304e-05
11	CD8+ T cells	*THEM6*	0.4795	0.227	0.024	4.484e-47	1.035e-42
11	CD8+ T cells	*STARD3NL*	0.4794	0.409	0.177	2.020e-11	4.664e-07
11	CD8+ T cells	*SLAMF6*	0.4788	0.197	0.004	1.543e-165	3.563e-161
11	CD8+ T cells	*LOC127490718*	0.4786	0.212	0.008	3.121e-114	7.207e-110
11	CD8+ T cells	*RPL4*	0.4783	0.917	0.758	1.588e-10	3.667e-06
11	CD8+ T cells	*DNTTIP2*	0.4783	0.379	0.165	2.752e-10	6.354e-06
11	CD8+ T cells	*THADA*	0.4773	0.379	0.155	2.544e-11	5.875e-07
11	CD8+ T cells	*CD38*	0.4772	0.197	0.003	2.376e-178	5.486e-174
11	CD8+ T cells	*LOC103350079*	0.4771	0.212	0.000	0	0
11	CD8+ T cells	*TMEM131*	0.4770	0.424	0.185	2.550e-11	5.888e-07
11	CD8+ T cells	*LOC100340280*	0.4764	0.167	0.008	2.214e-69	5.112e-65
11	CD8+ T cells	*LOC100345774*	0.4763	0.848	0.589	1.418e-09	3.274e-05
11	CD8+ T cells	*CCM2*	0.4759	0.295	0.065	1.923e-24	4.442e-20
11	CD8+ T cells	*TGFBR2*	0.4758	0.485	0.235	3.501e-10	8.086e-06
11	CD8+ T cells	*CKLF*	0.4756	0.348	0.088	1.077e-22	2.487e-18
11	CD8+ T cells	*RNF167*	0.4748	0.477	0.235	1.733e-09	4.002e-05
11	CD8+ T cells	*PTBP3*	0.4748	0.538	0.212	1.264e-16	2.918e-12
11	CD8+ T cells	*SMAP1*	0.4743	0.515	0.266	2.545e-09	5.876e-05
11	CD8+ T cells	*FSAF1*	0.4740	0.318	0.094	2.215e-17	5.114e-13
11	CD8+ T cells	*SYNGR2*	0.4738	0.356	0.103	1.837e-19	4.241e-15
11	CD8+ T cells	*TACC1*	0.4737	0.576	0.227	2.049e-17	4.732e-13
11	CD8+ T cells	*FGR*	0.4717	0.182	0.010	8.638e-72	1.995e-67
11	CD8+ T cells	*PAPOLA*	0.4705	0.735	0.421	6.893e-11	1.592e-06
11	CD8+ T cells	*PRPF38B*	0.4705	0.598	0.281	1.086e-11	2.507e-07
11	CD8+ T cells	*PPP1R3D*	0.4705	0.205	0.013	3.039e-70	7.018e-66
11	CD8+ T cells	*BAX*	0.4703	0.311	0.114	6.139e-12	1.418e-07
11	CD8+ T cells	*ELF4*	0.4700	0.273	0.023	3.507e-70	8.099e-66

Original cluster	Annotated cell type	Gene	avg_log2FC	pct.1	pct.2	Raw P value	Bonferroni-adjusted P value
11	CD8+ T cells	*GPR158*	0.4697	0.144	0.004	6.716e-89	1.551e-84
11	CD8+ T cells	*DNAJA2*	0.4691	0.538	0.271	1.776e-10	4.101e-06
11	CD8+ T cells	*FAM13B*	0.4688	0.508	0.281	2.340e-08	5.404e-04
11	CD8+ T cells	*ITGA11*	0.4683	0.189	0.017	1.363e-44	3.147e-40
11	CD8+ T cells	*ZFP82*	0.4682	0.258	0.033	3.595e-42	8.302e-38
11	CD8+ T cells	*RNF144A*	0.4676	0.318	0.094	5.564e-17	1.285e-12
11	CD8+ T cells	*EZR*	0.4674	0.235	0.024	2.870e-49	6.628e-45
11	CD8+ T cells	*LOC100351548*	0.4673	0.144	0.000	5.838e-288	1.348e-283
11	CD8+ T cells	*NDUFB1*	0.4667	0.432	0.211	1.693e-09	3.909e-05
11	CD8+ T cells	*CD3E*	0.4655	0.227	0.000	0	0
11	CD8+ T cells	*AKNA*	0.4653	0.235	0.023	3.901e-51	9.009e-47
11	CD8+ T cells	*RGS18*	0.4652	0.159	0.002	5.162e-164	1.192e-159
11	CD8+ T cells	*SET*	0.4650	0.614	0.327	3.089e-10	7.134e-06
11	CD8+ T cells	*LRRC8B*	0.4650	0.189	0.023	6.672e-33	1.541e-28
11	CD8+ T cells	*ABHD5*	0.4646	0.212	0.065	1.244e-11	2.873e-07
11	CD8+ T cells	*COX7C*	0.4636	0.856	0.652	9.506e-08	2.195e-03
11	CD8+ T cells	*NUP153*	0.4630	0.477	0.200	2.783e-13	6.426e-09
11	CD8+ T cells	*CRACR2A*	0.4622	0.303	0.087	3.702e-17	8.550e-13
11	CD8+ T cells	*PPP1R16B*	0.4621	0.182	0.012	1.232e-59	2.845e-55
11	CD8+ T cells	*CD244*	0.4585	0.197	0.002	1.028e-213	2.373e-209
11	CD8+ T cells	*LOC138846933*	0.4581	0.205	0.002	7.947e-242	1.835e-237
11	CD8+ T cells	*TCF25*	0.4574	0.750	0.457	3.153e-08	7.282e-04
11	CD8+ T cells	*PIK3R5*	0.4574	0.174	0.007	3.670e-88	8.474e-84
11	CD8+ T cells	*LOC103345396*	0.4563	0.136	0.000	6.882e-273	1.589e-268
11	CD8+ T cells	*CDYL2*	0.4562	0.182	0.009	8.697e-74	2.008e-69
11	CD8+ T cells	*CXXC5*	0.4546	0.432	0.226	4.487e-08	1.036e-03
11	CD8+ T cells	*UBE2N*	0.4537	0.492	0.214	1.183e-12	2.731e-08
11	CD8+ T cells	*DYRK1A*	0.4528	0.470	0.257	7.981e-09	1.843e-04
11	CD8+ T cells	*UBE2A*	0.4528	0.379	0.107	2.819e-21	6.509e-17
11	CD8+ T cells	*SOS1*	0.4528	0.394	0.209	1.091e-06	2.519e-02
11	CD8+ T cells	*COX8A*	0.4525	0.841	0.560	3.265e-09	7.540e-05
11	CD8+ T cells	*BCL6*	0.4524	0.371	0.160	8.422e-10	1.945e-05
11	CD8+ T cells	*RAPGEF1*	0.4521	0.235	0.051	3.289e-20	7.594e-16
11	CD8+ T cells	*LOC138843475*	0.4517	0.924	0.799	4.373e-08	1.010e-03
11	CD8+ T cells	*SRP9*	0.4513	0.477	0.196	1.426e-13	3.292e-09
11	CD8+ T cells	*RNGTT*	0.4512	0.386	0.174	4.874e-09	1.125e-04
11	CD8+ T cells	*GCLM*	0.4507	0.250	0.049	2.526e-24	5.833e-20
11	CD8+ T cells	*PARP8*	0.4507	0.220	0.072	1.716e-10	3.963e-06
11	CD8+ T cells	*USP36*	0.4505	0.242	0.045	4.608e-26	1.064e-21
11	CD8+ T cells	*ST6GALNAC4*	0.4501	0.235	0.052	1.943e-19	4.488e-15

Original cluster	Annotated cell type	Gene	avg_log2FC	pct.1	pct.2	Raw P value	Bonferroni-adjusted P value
11	CD8+ T cells	*LAMTOR5*	0.4494	0.568	0.270	2.668e-11	6.161e-07
11	CD8+ T cells	*P2RX5*	0.4487	0.144	0.001	4.485e-201	1.036e-196
11	CD8+ T cells	*LAIR1*	0.4487	0.167	0.007	1.749e-84	4.039e-80
11	CD8+ T cells	*SERF2*	0.4487	0.977	0.952	2.245e-13	5.184e-09
11	CD8+ T cells	*ATG101*	0.4478	0.424	0.159	1.394e-14	3.218e-10
11	CD8+ T cells	*PPP6R1*	0.4478	0.311	0.068	1.320e-25	3.049e-21
11	CD8+ T cells	*RNASEH2B*	0.4477	0.364	0.144	6.693e-12	1.546e-07
11	CD8+ T cells	*GRAMD1A*	0.4471	0.273	0.048	1.853e-30	4.279e-26
11	CD8+ T cells	*DDA1*	0.4470	0.364	0.119	2.598e-16	5.998e-12
11	CD8+ T cells	*PDLIM5*	0.4469	0.545	0.236	2.187e-13	5.050e-09
11	CD8+ T cells	*CDS2*	0.4469	0.371	0.115	7.125e-18	1.645e-13
11	CD8+ T cells	*MAP3K5*	0.4468	0.258	0.062	4.638e-19	1.071e-14
11	CD8+ T cells	*PHF6*	0.4464	0.356	0.150	2.870e-10	6.627e-06
11	CD8+ T cells	*MPHOSPH10*	0.4464	0.402	0.165	1.440e-11	3.324e-07
11	CD8+ T cells	*ELOVL5*	0.4460	0.303	0.134	2.518e-08	5.814e-04
11	CD8+ T cells	*TPR*	0.4458	0.621	0.389	1.066e-07	2.463e-03
11	CD8+ T cells	*CCDC88C*	0.4452	0.220	0.017	5.006e-64	1.156e-59
11	CD8+ T cells	*MAN2A1*	0.4449	0.447	0.222	2.060e-08	4.757e-04
11	CD8+ T cells	*CUL3*	0.4447	0.636	0.409	2.156e-06	4.979e-02
11	CD8+ T cells	*CSNK1G1*	0.4446	0.417	0.208	4.163e-08	9.614e-04
11	CD8+ T cells	*EIF5B*	0.4444	0.530	0.272	5.761e-10	1.330e-05
11	CD8+ T cells	*STAT1*	0.4442	0.379	0.141	2.570e-13	5.935e-09
11	CD8+ T cells	*ARF1*	0.4439	0.629	0.350	5.243e-09	1.211e-04
11	CD8+ T cells	*ARPP21*	0.4435	0.114	0.001	7.539e-154	1.741e-149
11	CD8+ T cells	*CEBPG*	0.4428	0.364	0.133	1.100e-13	2.539e-09
11	CD8+ T cells	*RSBN1L*	0.4427	0.697	0.371	9.666e-11	2.232e-06
11	CD8+ T cells	*DCUN1D1*	0.4415	0.356	0.130	1.594e-13	3.681e-09
11	CD8+ T cells	*NME2*	0.4415	0.879	0.695	2.672e-08	6.171e-04
11	CD8+ T cells	*RPS13*	0.4411	0.977	0.984	3.845e-21	8.878e-17
11	CD8+ T cells	*GADD45B*	0.4409	0.492	0.257	4.288e-08	9.901e-04
11	CD8+ T cells	*UBE2D3*	0.4406	0.735	0.495	1.423e-07	3.286e-03
11	CD8+ T cells	*SND1*	0.4406	0.455	0.193	1.262e-12	2.913e-08
11	CD8+ T cells	*NDUFS7*	0.4405	0.333	0.129	2.196e-11	5.072e-07
11	CD8+ T cells	*POLH*	0.4404	0.220	0.061	8.046e-14	1.858e-09
11	CD8+ T cells	*GRAP2*	0.4404	0.174	0.001	2.372e-265	5.476e-261
11	CD8+ T cells	*NBEAL2*	0.4402	0.197	0.016	7.839e-53	1.810e-48
11	CD8+ T cells	*LOC108176811*	0.4397	0.273	0.108	1.039e-08	2.400e-04
11	CD8+ T cells	*LRRC2*	0.4395	0.174	0.008	1.617e-75	3.733e-71
11	CD8+ T cells	*KDM6A*	0.4392	0.652	0.383	5.746e-09	1.327e-04
11	CD8+ T cells	*LOC138844356*	0.4388	0.174	0.003	1.160e-144	2.679e-140

Original cluster	Annotated cell type	Gene	avg_log2FC	pct.1	pct.2	Raw P value	Bonferroni-adjusted P value
11	CD8+ T cells	*SIGIRR*	0.4387	0.182	0.016	6.698e-46	1.547e-41
11	CD8+ T cells	*LOC100347048*	0.4384	0.273	0.087	2.870e-13	6.627e-09
11	CD8+ T cells	*SLC25A3*	0.4378	0.712	0.482	5.716e-07	1.320e-02
11	CD8+ T cells	*C6H1orf35*	0.4376	0.667	0.364	1.634e-09	3.773e-05
11	CD8+ T cells	*STK24*	0.4374	0.477	0.179	1.133e-15	2.616e-11
11	CD8+ T cells	*ZC3H12A*	0.4371	0.189	0.016	3.412e-49	7.879e-45
11	CD8+ T cells	*HERC4*	0.4368	0.530	0.257	1.053e-10	2.432e-06
11	CD8+ T cells	*TBCA*	0.4367	0.871	0.661	6.961e-09	1.607e-04
11	CD8+ T cells	*ARID5A*	0.4362	0.295	0.055	2.550e-30	5.889e-26
11	CD8+ T cells	*CHD7*	0.4361	0.250	0.035	1.767e-37	4.079e-33
11	CD8+ T cells	*TMOD3*	0.4360	0.591	0.240	1.481e-16	3.419e-12
11	CD8+ T cells	*VAMP5*	0.4357	0.295	0.059	1.233e-27	2.848e-23
11	CD8+ T cells	*NME1*	0.4356	0.250	0.047	7.472e-26	1.726e-21
11	CD8+ T cells	*VAMP2*	0.4355	0.470	0.217	1.509e-10	3.484e-06
11	CD8+ T cells	*JAK2*	0.4352	0.402	0.176	1.842e-10	4.255e-06
11	CD8+ T cells	*RAB11A*	0.4351	0.348	0.125	1.339e-13	3.092e-09
11	CD8+ T cells	*COPE*	0.4349	0.492	0.267	1.536e-07	3.547e-03
11	CD8+ T cells	*DAZAP1*	0.4343	0.386	0.181	2.015e-08	4.654e-04
11	CD8+ T cells	*GARS1*	0.4342	0.303	0.099	5.605e-14	1.294e-09
11	CD8+ T cells	*RABEP1*	0.4342	0.379	0.155	1.291e-11	2.981e-07
11	CD8+ T cells	*MRPL41*	0.4337	0.379	0.191	1.356e-07	3.132e-03
11	CD8+ T cells	*GOLPH3*	0.4337	0.379	0.166	6.574e-10	1.518e-05
11	CD8+ T cells	*ARHGAP4*	0.4336	0.220	0.011	3.398e-91	7.848e-87
11	CD8+ T cells	*MCF2L2*	0.4328	0.159	0.016	4.762e-34	1.100e-29
11	CD8+ T cells	*TXNDC9*	0.4327	0.303	0.073	6.155e-22	1.421e-17
11	CD8+ T cells	*NAP1L1*	0.4320	0.909	0.742	3.013e-07	6.957e-03
11	CD8+ T cells	*GNA13*	0.4319	0.386	0.163	4.911e-11	1.134e-06
11	CD8+ T cells	*BUD31*	0.4318	0.652	0.342	7.803e-11	1.802e-06
11	CD8+ T cells	*AFTPH*	0.4316	0.341	0.147	1.115e-09	2.576e-05
11	CD8+ T cells	*ABCF1*	0.4314	0.235	0.069	2.718e-13	6.277e-09
11	CD8+ T cells	*SLCO3A1*	0.4309	0.159	0.011	4.813e-50	1.111e-45
11	CD8+ T cells	*FAM120A*	0.4309	0.568	0.276	2.139e-11	4.940e-07
11	CD8+ T cells	*IPO5*	0.4303	0.348	0.154	2.571e-09	5.936e-05
11	CD8+ T cells	*CDCA7L*	0.4302	0.250	0.050	8.139e-24	1.879e-19
11	CD8+ T cells	*C1GALT1*	0.4301	0.295	0.077	1.130e-18	2.609e-14
11	CD8+ T cells	*TNFSF9*	0.4301	0.152	0.009	5.462e-55	1.261e-50
11	CD8+ T cells	*TRIM33*	0.4298	0.439	0.221	2.357e-08	5.442e-04
11	CD8+ T cells	*CTCF*	0.4294	0.485	0.213	1.047e-11	2.418e-07
11	CD8+ T cells	*EAF2*	0.4284	0.220	0.044	7.570e-21	1.748e-16
11	CD8+ T cells	*STX11*	0.4282	0.167	0.006	3.803e-89	8.783e-85

Original cluster	Annotated cell type	Gene	avg_log2FC	pct.1	pct.2	Raw P value	Bonferroni-adjusted P value
11	CD8+ T cells	*NRDC*	0.4282	0.485	0.218	5.541e-11	1.279e-06
11	CD8+ T cells	*CYP2J2*	0.4278	0.364	0.160	1.245e-09	2.875e-05
11	CD8+ T cells	*CBL*	0.4277	0.288	0.077	2.486e-18	5.741e-14
11	CD8+ T cells	*EDEM1*	0.4276	0.227	0.044	1.452e-22	3.353e-18
11	CD8+ T cells	*GSPT1*	0.4275	0.545	0.247	3.038e-12	7.015e-08
11	CD8+ T cells	*LSM1*	0.4270	0.295	0.115	2.028e-10	4.682e-06
11	CD8+ T cells	*SPECC1*	0.4265	0.212	0.068	1.125e-10	2.597e-06
11	CD8+ T cells	*TMEM154*	0.4265	0.205	0.016	5.426e-56	1.253e-51
11	CD8+ T cells	*AKT1*	0.4265	0.386	0.164	7.324e-11	1.691e-06
11	CD8+ T cells	*LOC138846176*	0.4261	0.197	0.003	1.222e-188	2.823e-184
11	CD8+ T cells	*HNRNPK*	0.4252	0.833	0.616	1.400e-06	3.233e-02
11	CD8+ T cells	*LOC100009451*	0.4251	0.205	0.008	4.752e-100	1.097e-95
11	CD8+ T cells	*TTN*	0.4248	0.136	0.004	1.710e-82	3.950e-78
11	CD8+ T cells	*LOC100341994*	0.4244	0.197	0.001	2.651e-299	6.122e-295
11	CD8+ T cells	*MGST1*	0.4244	0.220	0.028	4.176e-37	9.644e-33
11	CD8+ T cells	*ANAPC11*	0.4233	0.591	0.287	5.056e-11	1.168e-06
11	CD8+ T cells	*CCNT1*	0.4225	0.356	0.125	9.363e-14	2.162e-09
11	CD8+ T cells	*PPP2CA*	0.4225	0.439	0.208	1.339e-09	3.093e-05
11	CD8+ T cells	*TMCC1*	0.4216	0.348	0.132	6.172e-12	1.425e-07
11	CD8+ T cells	*TOP1*	0.4212	0.818	0.567	9.486e-08	2.191e-03
11	CD8+ T cells	*CYBA*	0.4211	0.455	0.181	1.759e-13	4.062e-09
11	CD8+ T cells	*CHMP3*	0.4209	0.553	0.319	7.896e-07	1.823e-02
11	CD8+ T cells	*IL16*	0.4201	0.205	0.020	2.564e-44	5.922e-40
11	CD8+ T cells	*HBS1L*	0.4199	0.523	0.283	2.596e-08	5.994e-04
11	CD8+ T cells	*AGK*	0.4197	0.273	0.080	7.133e-15	1.647e-10
11	CD8+ T cells	*TMEM273*	0.4195	0.197	0.017	1.475e-50	3.407e-46
11	CD8+ T cells	*MCMBP*	0.4195	0.273	0.078	1.821e-15	4.206e-11
11	CD8+ T cells	*DAPK2*	0.4194	0.174	0.015	1.383e-44	3.194e-40
11	CD8+ T cells	*ERBIN*	0.4193	0.598	0.389	7.694e-07	1.777e-02
11	CD8+ T cells	*UBN1*	0.4188	0.326	0.099	1.588e-16	3.666e-12
11	CD8+ T cells	*LSM14A*	0.4184	0.523	0.275	4.173e-09	9.636e-05
11	CD8+ T cells	*MNAT1*	0.4183	0.394	0.180	3.316e-09	7.658e-05
11	CD8+ T cells	*EGR2*	0.4180	0.159	0.025	3.839e-21	8.864e-17
11	CD8+ T cells	*PICALM*	0.4180	0.568	0.285	6.979e-11	1.612e-06
11	CD8+ T cells	*XRCC6*	0.4175	0.311	0.115	9.356e-12	2.160e-07
11	CD8+ T cells	*COPS9*	0.4161	0.576	0.299	1.933e-09	4.465e-05
11	CD8+ T cells	*KATNBL1*	0.4160	0.356	0.160	1.378e-08	3.181e-04
11	CD8+ T cells	*DNAJC25*	0.4160	0.273	0.084	1.430e-13	3.303e-09
11	CD8+ T cells	*LOC100357058*	0.4157	0.402	0.150	1.076e-13	2.485e-09
11	CD8+ T cells	*KAT6A*	0.4148	0.371	0.196	7.495e-07	1.731e-02

Original cluster	Annotated cell type	Gene	avg_log2FC	pct.1	pct.2	Raw P value	Bonferroni-adjusted P value
11	CD8+ T cells	*MARCHF7*	0.4144	0.576	0.332	3.056e-07	7.058e-03
11	CD8+ T cells	*MRPL28*	0.4142	0.295	0.104	3.497e-12	8.076e-08
11	CD8+ T cells	*CNOT1*	0.4139	0.470	0.203	8.972e-12	2.072e-07
11	CD8+ T cells	*ZMAT2*	0.4132	0.311	0.127	1.331e-09	3.073e-05
11	CD8+ T cells	*CLTC*	0.4130	0.515	0.260	4.243e-10	9.799e-06
11	CD8+ T cells	*SRA1*	0.4125	0.220	0.053	1.245e-16	2.875e-12
11	CD8+ T cells	*PLGRKT*	0.4125	0.402	0.180	2.217e-09	5.120e-05
11	CD8+ T cells	*HSD17B11*	0.4125	0.189	0.029	1.469e-25	3.391e-21
11	CD8+ T cells	*NCKAP1L*	0.4125	0.205	0.017	5.129e-53	1.184e-48
11	CD8+ T cells	*TESPA1*	0.4121	0.152	0.001	1.246e-231	2.877e-227
11	CD8+ T cells	*LAX1*	0.4116	0.174	0.002	4.627e-192	1.068e-187
11	CD8+ T cells	*SEC23B*	0.4114	0.288	0.079	2.299e-17	5.308e-13
11	CD8+ T cells	*USP4*	0.4113	0.303	0.089	2.540e-16	5.865e-12
11	CD8+ T cells	*LAT2*	0.4110	0.189	0.007	2.999e-97	6.925e-93
11	CD8+ T cells	*TSTD1*	0.4108	0.205	0.030	1.867e-28	4.310e-24
11	CD8+ T cells	*PRP4K*	0.4106	0.591	0.344	6.080e-08	1.404e-03
11	CD8+ T cells	*TNFAIP8L2*	0.4104	0.167	0.005	1.094e-106	2.526e-102
11	CD8+ T cells	*GCSAML*	0.4101	0.159	0.002	2.155e-168	4.976e-164
11	CD8+ T cells	*PTAR1*	0.4099	0.348	0.155	8.681e-09	2.005e-04
11	CD8+ T cells	*DAZAP2*	0.4092	0.364	0.147	6.132e-11	1.416e-06
11	CD8+ T cells	*CD84*	0.4092	0.136	0.002	7.403e-138	1.710e-133
11	CD8+ T cells	*RAB21*	0.4092	0.394	0.176	5.972e-10	1.379e-05
11	CD8+ T cells	*YBX1*	0.4090	0.894	0.693	1.718e-07	3.967e-03
11	CD8+ T cells	*HSD17B12*	0.4090	0.394	0.184	9.184e-09	2.121e-04
11	CD8+ T cells	*FBXL5*	0.4085	0.424	0.180	6.988e-11	1.614e-06
11	CD8+ T cells	*ITPA*	0.4076	0.386	0.143	3.681e-13	8.501e-09
11	CD8+ T cells	*P2RY8*	0.4072	0.167	0.002	1.197e-180	2.765e-176
11	CD8+ T cells	*TNKS2*	0.4072	0.424	0.214	3.616e-08	8.349e-04
11	CD8+ T cells	*PSD4*	0.4070	0.212	0.025	1.031e-37	2.380e-33
11	CD8+ T cells	*CARD11*	0.4066	0.159	0.003	3.445e-145	7.955e-141
11	CD8+ T cells	*CASC3*	0.4063	0.303	0.118	7.828e-10	1.808e-05
11	CD8+ T cells	*SNX20*	0.4058	0.220	0.009	7.392e-107	1.707e-102
11	CD8+ T cells	*DAG1*	0.4043	0.371	0.150	2.839e-11	6.556e-07
11	CD8+ T cells	*CSNK1G2*	0.4040	0.462	0.219	1.136e-09	2.622e-05
11	CD8+ T cells	*KPNA1*	0.4040	0.318	0.092	2.387e-17	5.512e-13
11	CD8+ T cells	*UBP1*	0.4039	0.303	0.105	7.113e-13	1.643e-08
11	CD8+ T cells	*TMEM141*	0.4030	0.371	0.124	1.145e-15	2.645e-11
11	CD8+ T cells	*RPS17*	0.4028	0.992	0.983	7.328e-15	1.692e-10
11	CD8+ T cells	*PIK3CB*	0.4027	0.258	0.088	6.881e-11	1.589e-06
11	CD8+ T cells	*ST3GAL2*	0.4023	0.326	0.102	3.173e-15	7.326e-11

Original cluster	Annotated cell type	Gene	avg_log2FC	pct.1	pct.2	Raw P value	Bonferroni-adjusted P value
11	CD8+ T cells	*LOC138843213*	0.4014	0.659	0.399	3.960e-08	9.144e-04
11	CD8+ T cells	*LOC100342498*	0.4014	0.205	0.004	2.685e-170	6.201e-166
11	CD8+ T cells	*RAN*	0.4013	0.394	0.175	3.054e-09	7.052e-05
11	CD8+ T cells	*SMAD2*	0.4012	0.417	0.208	3.414e-08	7.884e-04
11	CD8+ T cells	*SOD2*	0.4010	0.295	0.112	9.256e-11	2.137e-06
11	CD8+ T cells	*GNAI3*	0.4009	0.576	0.321	4.494e-08	1.038e-03
11	CD8+ T cells	*EIF4G2*	0.4009	0.795	0.591	2.436e-07	5.625e-03
11	CD8+ T cells	*NFATC3*	0.4005	0.447	0.193	2.389e-11	5.517e-07
11	CD8+ T cells	*ATP11B*	0.3991	0.394	0.187	5.146e-09	1.188e-04
11	CD8+ T cells	*SH3BP1*	0.3986	0.197	0.020	5.967e-42	1.378e-37
11	CD8+ T cells	*PRPF40A*	0.3984	0.674	0.393	1.398e-07	3.228e-03
11	CD8+ T cells	*MARK3*	0.3983	0.417	0.179	4.852e-11	1.120e-06
11	CD8+ T cells	*NDUFB5*	0.3974	0.606	0.325	8.187e-10	1.891e-05
11	CD8+ T cells	*DNAJC14*	0.3972	0.258	0.075	3.208e-14	7.408e-10
11	CD8+ T cells	*LOC138844857*	0.3961	0.144	0.017	1.016e-25	2.346e-21
11	CD8+ T cells	*TRAF3*	0.3961	0.258	0.089	7.329e-11	1.692e-06
11	CD8+ T cells	*SETD3*	0.3954	0.152	0.042	5.281e-10	1.220e-05
11	CD8+ T cells	*TMEM160*	0.3953	0.295	0.088	1.274e-15	2.941e-11
11	CD8+ T cells	*PDCD6*	0.3950	0.500	0.268	3.589e-08	8.288e-04
11	CD8+ T cells	*CCDC198*	0.3949	0.167	0.001	6.487e-227	1.498e-222
11	CD8+ T cells	*EWSR1*	0.3947	0.712	0.478	3.247e-07	7.497e-03
11	CD8+ T cells	*PDS5A*	0.3946	0.424	0.214	6.464e-08	1.493e-03
11	CD8+ T cells	*MTMR10*	0.3937	0.235	0.050	1.162e-20	2.684e-16
11	CD8+ T cells	*PDE4D*	0.3936	0.402	0.179	5.277e-11	1.219e-06
11	CD8+ T cells	*DSN1*	0.3931	0.144	0.018	1.332e-24	3.075e-20
11	CD8+ T cells	*CDYL*	0.3924	0.258	0.091	1.499e-10	3.461e-06
11	CD8+ T cells	*TLE5*	0.3917	0.856	0.662	4.065e-07	9.387e-03
11	CD8+ T cells	*SMARCC1*	0.3915	0.508	0.271	6.577e-08	1.519e-03
11	CD8+ T cells	*CSE1L*	0.3910	0.273	0.070	3.844e-18	8.876e-14
11	CD8+ T cells	*WWP2*	0.3901	0.265	0.067	2.493e-18	5.756e-14
11	CD8+ T cells	*ERCC6*	0.3897	0.189	0.036	4.080e-20	9.421e-16
11	CD8+ T cells	*RARA*	0.3887	0.227	0.061	1.403e-14	3.240e-10
11	CD8+ T cells	*CHD1L*	0.3887	0.212	0.049	7.187e-17	1.660e-12
11	CD8+ T cells	*VPS4B*	0.3885	0.402	0.172	6.754e-11	1.560e-06
11	CD8+ T cells	*ZBTB1*	0.3875	0.288	0.096	7.556e-13	1.745e-08
11	CD8+ T cells	*TP53INP2*	0.3872	0.182	0.024	1.770e-29	4.087e-25
11	CD8+ T cells	*ST3GAL1*	0.3866	0.212	0.029	1.829e-32	4.224e-28
11	CD8+ T cells	*CUL2*	0.3865	0.280	0.109	7.642e-10	1.765e-05
11	CD8+ T cells	*ME2*	0.3863	0.273	0.076	4.869e-16	1.124e-11
11	CD8+ T cells	*TIPARP*	0.3863	0.606	0.335	9.908e-10	2.288e-05

Original cluster	Annotated cell type	Gene	avg_log2FC	pct.1	pct.2	Raw P value	Bonferroni-adjusted P value
11	CD8+ T cells	*TIMM13*	0.3859	0.515	0.263	1.913e-08	4.418e-04
11	CD8+ T cells	*ATP5F1A*	0.3855	0.606	0.372	4.368e-07	1.009e-02
11	CD8+ T cells	*INPP5B*	0.3848	0.220	0.060	6.793e-14	1.569e-09
11	CD8+ T cells	*TRIM38*	0.3845	0.220	0.051	1.958e-17	4.522e-13
11	CD8+ T cells	*TSPAN32*	0.3836	0.189	0.008	2.971e-87	6.862e-83
11	CD8+ T cells	*PDCD4*	0.3832	0.561	0.328	9.535e-07	2.202e-02
11	CD8+ T cells	*ATP13A3*	0.3823	0.258	0.100	3.939e-09	9.096e-05
11	CD8+ T cells	*SLC35B3*	0.3822	0.212	0.091	1.732e-06	4.000e-02
11	CD8+ T cells	*CSRNP1*	0.3815	0.545	0.215	1.319e-15	3.045e-11
11	CD8+ T cells	*SMIM3*	0.3815	0.159	0.018	3.803e-30	8.782e-26
11	CD8+ T cells	*RBMS1*	0.3808	0.758	0.542	2.023e-06	4.672e-02
11	CD8+ T cells	*FAM174C*	0.3806	0.250	0.070	7.283e-15	1.682e-10
11	CD8+ T cells	*SVIP*	0.3804	0.182	0.041	4.671e-15	1.079e-10
11	CD8+ T cells	*ATF7IP2*	0.3804	0.189	0.030	7.443e-25	1.719e-20
11	CD8+ T cells	*SRPK1*	0.3793	0.318	0.132	1.834e-09	4.235e-05
11	CD8+ T cells	*UBE2K*	0.3793	0.356	0.165	1.613e-08	3.724e-04
11	CD8+ T cells	*NDUFA11*	0.3792	0.545	0.299	1.026e-07	2.370e-03
11	CD8+ T cells	*SCP2*	0.3792	0.364	0.156	7.394e-10	1.707e-05
11	CD8+ T cells	*GLRX2*	0.3790	0.280	0.105	6.093e-10	1.407e-05
11	CD8+ T cells	*SIAH2*	0.3788	0.333	0.099	5.446e-17	1.258e-12
11	CD8+ T cells	*ABHD17B*	0.3787	0.318	0.156	1.243e-06	2.870e-02
11	CD8+ T cells	*UBE2D2*	0.3784	0.583	0.301	6.264e-09	1.446e-04
11	CD8+ T cells	*CNOT6*	0.3783	0.348	0.122	1.961e-13	4.528e-09
11	CD8+ T cells	*GPCPD1*	0.3782	0.492	0.275	1.070e-07	2.472e-03
11	CD8+ T cells	*ETV3*	0.3772	0.303	0.127	4.962e-09	1.146e-04
11	CD8+ T cells	*RGS19*	0.3769	0.197	0.037	4.481e-21	1.035e-16
11	CD8+ T cells	*SLC31A2*	0.3766	0.189	0.038	4.652e-18	1.074e-13
11	CD8+ T cells	*RAB37*	0.3765	0.129	0.004	3.808e-86	8.793e-82
11	CD8+ T cells	*KDM7A*	0.3763	0.333	0.142	2.081e-09	4.805e-05
11	CD8+ T cells	*SFMBT2*	0.3756	0.356	0.175	2.751e-07	6.353e-03
11	CD8+ T cells	*LOC138850176*	0.3749	0.220	0.025	4.611e-41	1.065e-36
11	CD8+ T cells	*SEC14L1*	0.3745	0.303	0.087	1.058e-16	2.444e-12
11	CD8+ T cells	*BMAL1*	0.3745	0.174	0.013	1.407e-49	3.248e-45
11	CD8+ T cells	*NRF1*	0.3742	0.303	0.147	9.697e-07	2.239e-02
11	CD8+ T cells	*RPS16*	0.3738	0.977	0.980	2.298e-14	5.307e-10
11	CD8+ T cells	*NCK1*	0.3737	0.621	0.346	5.848e-09	1.350e-04
11	CD8+ T cells	*IRF8*	0.3734	0.159	0.010	2.945e-56	6.802e-52
11	CD8+ T cells	*LOC100349893*	0.3726	0.197	0.035	4.116e-22	9.505e-18
11	CD8+ T cells	*MAP3K1*	0.3716	0.318	0.158	1.315e-06	3.037e-02
11	CD8+ T cells	*MRPL14*	0.3711	0.455	0.211	1.008e-09	2.327e-05

Original cluster	Annotated cell type	Gene	avg_log2FC	pct.1	pct.2	Raw P value	Bonferroni-adjusted P value
11	CD8+ T cells	*PSPC1*	0.3707	0.591	0.336	4.942e-08	1.141e-03
11	CD8+ T cells	*NDEL1*	0.3704	0.394	0.151	1.171e-12	2.704e-08
11	CD8+ T cells	*DAPP1*	0.3697	0.129	0.005	1.517e-68	3.503e-64
11	CD8+ T cells	*CD101*	0.3693	0.129	0.007	4.674e-48	1.079e-43
11	CD8+ T cells	*PSMA2*	0.3693	0.538	0.294	3.700e-08	8.544e-04
11	CD8+ T cells	*ABHD12*	0.3691	0.182	0.039	4.010e-16	9.261e-12
11	CD8+ T cells	*KMT2E*	0.3690	0.841	0.652	7.585e-07	1.751e-02
11	CD8+ T cells	*YAF2*	0.3686	0.470	0.235	2.319e-08	5.355e-04
11	CD8+ T cells	*DCTPP1*	0.3684	0.341	0.142	3.056e-09	7.056e-05
11	CD8+ T cells	*LOC103347986*	0.3681	0.273	0.106	2.677e-09	6.182e-05
11	CD8+ T cells	*ARHGAP27*	0.3672	0.144	0.014	5.104e-33	1.179e-28
11	CD8+ T cells	*AMFR*	0.3669	0.364	0.147	1.601e-10	3.697e-06
11	CD8+ T cells	*LOC100348267*	0.3668	0.288	0.138	1.520e-06	3.511e-02
11	CD8+ T cells	*CHCHD7*	0.3665	0.258	0.113	4.049e-07	9.349e-03
11	CD8+ T cells	*RNPEPL1*	0.3653	0.197	0.048	8.964e-15	2.070e-10
11	CD8+ T cells	*TACC3*	0.3650	0.159	0.011	1.662e-50	3.837e-46
11	CD8+ T cells	*SAMD3*	0.3648	0.144	0.001	8.864e-227	2.047e-222
11	CD8+ T cells	*SLAIN2*	0.3647	0.462	0.244	2.907e-07	6.713e-03
11	CD8+ T cells	*STAU1*	0.3644	0.356	0.158	3.655e-09	8.441e-05
11	CD8+ T cells	*EMC6*	0.3638	0.538	0.266	1.741e-08	4.021e-04
11	CD8+ T cells	*TMEM71*	0.3637	0.129	0.002	3.150e-139	7.273e-135
11	CD8+ T cells	*MANF*	0.3636	0.356	0.142	8.603e-11	1.987e-06
11	CD8+ T cells	*UBE2F*	0.3631	0.341	0.136	9.813e-11	2.266e-06
11	CD8+ T cells	*ZFC3H1*	0.3630	0.462	0.230	6.749e-09	1.559e-04
11	CD8+ T cells	*RBKS*	0.3629	0.197	0.063	6.421e-10	1.483e-05
11	CD8+ T cells	*MLXIP*	0.3628	0.295	0.075	1.103e-19	2.546e-15
11	CD8+ T cells	*MEGF9*	0.3622	0.174	0.054	1.688e-09	3.899e-05
11	CD8+ T cells	*SMIM14*	0.3619	0.288	0.103	3.361e-11	7.762e-07
11	CD8+ T cells	*LOC138844132*	0.3619	0.152	0.003	1.087e-137	2.509e-133
11	CD8+ T cells	*FAR1*	0.3617	0.341	0.160	1.956e-07	4.516e-03
11	CD8+ T cells	*RHEB*	0.3615	0.735	0.469	1.930e-06	4.456e-02
11	CD8+ T cells	*LOC100355886*	0.3614	0.591	0.304	1.853e-09	4.278e-05
11	CD8+ T cells	*TOPBP1*	0.3612	0.250	0.105	1.144e-07	2.643e-03
11	CD8+ T cells	*LOC127490299*	0.3607	0.167	0.013	6.696e-45	1.546e-40
11	CD8+ T cells	*FEM1C*	0.3604	0.288	0.118	1.378e-08	3.181e-04
11	CD8+ T cells	*CD5*	0.3602	0.159	0.000	6.253e-290	1.444e-285
11	CD8+ T cells	*DENND1C*	0.3598	0.152	0.002	7.192e-171	1.661e-166
11	CD8+ T cells	*ZNF827*	0.3597	0.348	0.150	3.524e-09	8.139e-05
11	CD8+ T cells	*USP24*	0.3594	0.417	0.195	6.793e-09	1.569e-04
11	CD8+ T cells	*LOC138846723*	0.3594	0.311	0.119	1.936e-10	4.470e-06

Original cluster	Annotated cell type	Gene	avg_log2FC	pct.1	pct.2	Raw P value	Bonferroni-adjusted P value
11	CD8+ T cells	*LOC127485353*	0.3593	0.182	0.037	2.466e-17	5.695e-13
11	CD8+ T cells	*WDR33*	0.3592	0.568	0.314	3.345e-08	7.725e-04
11	CD8+ T cells	*NAA38*	0.3583	0.250	0.069	5.278e-15	1.219e-10
11	CD8+ T cells	*ARCN1*	0.3582	0.394	0.204	7.259e-07	1.676e-02
11	CD8+ T cells	*MRPL9*	0.3579	0.318	0.136	6.453e-09	1.490e-04
11	CD8+ T cells	*AAGAB*	0.3572	0.182	0.034	3.001e-19	6.929e-15
11	CD8+ T cells	*UBE2L3*	0.3566	0.659	0.404	3.687e-07	8.514e-03
11	CD8+ T cells	*ARLN*	0.3560	0.356	0.167	6.278e-08	1.450e-03
11	CD8+ T cells	*LOC138846210*	0.3551	0.159	0.030	9.962e-17	2.300e-12
11	CD8+ T cells	*GLUD1*	0.3547	0.538	0.316	9.549e-07	2.205e-02
11	CD8+ T cells	*ANKMY2*	0.3543	0.333	0.148	1.084e-08	2.502e-04
11	CD8+ T cells	*HMGCR*	0.3540	0.333	0.153	5.461e-08	1.261e-03
11	CD8+ T cells	*RPS15*	0.3539	0.992	0.989	1.488e-12	3.437e-08
11	CD8+ T cells	*CHIC2*	0.3538	0.394	0.170	9.465e-10	2.186e-05
11	CD8+ T cells	*EMC7*	0.3535	0.333	0.151	5.160e-08	1.192e-03
11	CD8+ T cells	*TP53*	0.3535	0.273	0.100	2.788e-10	6.439e-06
11	CD8+ T cells	*TLE3*	0.3525	0.197	0.043	3.953e-17	9.129e-13
11	CD8+ T cells	*SPSB2*	0.3525	0.311	0.143	2.560e-07	5.911e-03
11	CD8+ T cells	*ADORA2A*	0.3521	0.136	0.005	9.874e-70	2.280e-65
11	CD8+ T cells	*CHCHD5*	0.3516	0.182	0.050	1.820e-11	4.203e-07
11	CD8+ T cells	*ARPP19*	0.3497	0.515	0.240	1.531e-09	3.536e-05
11	CD8+ T cells	*RPL36AL*	0.3494	0.227	0.054	3.288e-17	7.592e-13
11	CD8+ T cells	*LOC127493173*	0.3489	0.144	0.007	5.841e-64	1.349e-59
11	CD8+ T cells	*LOC100355380*	0.3488	0.136	0.011	3.793e-36	8.758e-32
11	CD8+ T cells	*CDC14A*	0.3485	0.265	0.076	7.164e-15	1.654e-10
11	CD8+ T cells	*PLEKHM1*	0.3474	0.197	0.038	5.801e-20	1.340e-15
11	CD8+ T cells	*RNF216*	0.3466	0.333	0.152	3.466e-08	8.004e-04
11	CD8+ T cells	*ATP5MC2*	0.3466	0.894	0.755	6.995e-07	1.615e-02
11	CD8+ T cells	*EBAG9*	0.3461	0.273	0.091	3.080e-12	7.113e-08
11	CD8+ T cells	*HSPA9*	0.3461	0.462	0.225	8.092e-09	1.868e-04
11	CD8+ T cells	*EIF3D*	0.3452	0.371	0.179	1.042e-07	2.407e-03
11	CD8+ T cells	*STK40*	0.3448	0.235	0.084	1.859e-09	4.294e-05
11	CD8+ T cells	*MTHFD1L*	0.3436	0.280	0.098	4.284e-11	9.892e-07
11	CD8+ T cells	*HSBP1L1*	0.3434	0.182	0.015	9.986e-49	2.306e-44
11	CD8+ T cells	*TRAK2*	0.3432	0.235	0.089	9.678e-09	2.235e-04
11	CD8+ T cells	*EPS15*	0.3432	0.508	0.287	8.677e-07	2.004e-02
11	CD8+ T cells	*PSMB3*	0.3431	0.477	0.209	1.703e-10	3.933e-06
11	CD8+ T cells	*SRP19*	0.3427	0.462	0.252	9.379e-07	2.166e-02
11	CD8+ T cells	*CGGBP1*	0.3426	0.598	0.328	6.909e-08	1.595e-03
11	CD8+ T cells	*REST*	0.3417	0.265	0.094	1.420e-10	3.279e-06

Original cluster	Annotated cell type	Gene	avg_log2FC	pct.1	pct.2	Raw P value	Bonferroni-adjusted P value
11	CD8+ T cells	*DDX21*	0.3417	0.364	0.156	1.461e-09	3.373e-05
11	CD8+ T cells	*DPM1*	0.3412	0.356	0.181	1.256e-06	2.900e-02
11	CD8+ T cells	*SERBP1*	0.3406	0.856	0.621	1.469e-06	3.393e-02
11	CD8+ T cells	*MGST3*	0.3405	0.727	0.465	7.588e-07	1.752e-02
11	CD8+ T cells	*GALNT11*	0.3404	0.258	0.105	5.761e-08	1.330e-03
11	CD8+ T cells	*DENND2D*	0.3401	0.144	0.003	3.332e-106	7.694e-102
11	CD8+ T cells	*BCAS4*	0.3400	0.227	0.043	1.148e-22	2.650e-18
11	CD8+ T cells	*CCNT2*	0.3399	0.530	0.287	8.231e-08	1.901e-03
11	CD8+ T cells	*PARG*	0.3398	0.235	0.074	1.141e-11	2.634e-07
11	CD8+ T cells	*ITGA2*	0.3396	0.220	0.071	1.661e-10	3.836e-06
11	CD8+ T cells	*ICAM2*	0.3392	0.189	0.039	7.703e-18	1.779e-13
11	CD8+ T cells	*ATP2A3*	0.3391	0.159	0.007	1.335e-75	3.082e-71
11	CD8+ T cells	*DERA*	0.3391	0.371	0.186	2.438e-07	5.631e-03
11	CD8+ T cells	*UPP1*	0.3390	0.167	0.016	2.133e-37	4.927e-33
11	CD8+ T cells	*PPM1D*	0.3386	0.273	0.106	3.354e-09	7.746e-05
11	CD8+ T cells	*SPATA13*	0.3381	0.242	0.055	2.070e-19	4.779e-15
11	CD8+ T cells	*PTPN11*	0.3377	0.295	0.092	2.129e-14	4.915e-10
11	CD8+ T cells	*MAPK13*	0.3366	0.152	0.009	4.271e-55	9.862e-51
11	CD8+ T cells	*CDC25B*	0.3364	0.129	0.027	2.088e-12	4.822e-08
11	CD8+ T cells	*NR2C2*	0.3364	0.273	0.130	1.992e-06	4.599e-02
11	CD8+ T cells	*LOC138844436*	0.3364	0.174	0.015	8.850e-44	2.044e-39
11	CD8+ T cells	*DYM*	0.3351	0.417	0.220	4.609e-07	1.064e-02
11	CD8+ T cells	*HTT*	0.3344	0.318	0.113	3.653e-12	8.436e-08
11	CD8+ T cells	*BCAS3*	0.3339	0.629	0.387	1.433e-06	3.308e-02
11	CD8+ T cells	*UBL5*	0.3337	0.924	0.844	1.806e-07	4.171e-03
11	CD8+ T cells	*CDC42EP3*	0.3336	0.197	0.042	1.910e-17	4.410e-13
11	CD8+ T cells	*MYCBP*	0.3334	0.174	0.023	1.953e-27	4.510e-23
11	CD8+ T cells	*LOC103347227*	0.3332	0.402	0.208	7.411e-07	1.711e-02
11	CD8+ T cells	*EIF1AX*	0.3324	0.553	0.312	9.493e-07	2.192e-02
11	CD8+ T cells	*MAP4K1*	0.3318	0.152	0.002	5.263e-166	1.215e-161
11	CD8+ T cells	*COMMD8*	0.3311	0.265	0.112	9.014e-08	2.082e-03
11	CD8+ T cells	*OFD1*	0.3311	0.356	0.178	4.486e-07	1.036e-02
11	CD8+ T cells	*MRPL57*	0.3311	0.386	0.191	2.012e-07	4.646e-03
11	CD8+ T cells	*LOC100352968*	0.3310	0.970	0.921	7.135e-07	1.648e-02
11	CD8+ T cells	*GALNT1*	0.3307	0.515	0.290	4.683e-07	1.081e-02
11	CD8+ T cells	*AKAP5*	0.3298	0.114	0.025	2.825e-10	6.524e-06
11	CD8+ T cells	*RFC3*	0.3296	0.280	0.126	3.290e-07	7.596e-03
11	CD8+ T cells	*HACD4*	0.3295	0.189	0.043	1.852e-15	4.277e-11
11	CD8+ T cells	*TMX3*	0.3291	0.258	0.112	2.419e-07	5.587e-03
11	CD8+ T cells	*SPOCK2*	0.3290	0.152	0.016	5.164e-32	1.193e-27

Original cluster	Annotated cell type	Gene	avg_log2FC	pct.1	pct.2	Raw P value	Bonferroni-adjusted P value
11	CD8+ T cells	*TRAPPC9*	0.3285	0.265	0.087	7.243e-12	1.673e-07
11	CD8+ T cells	*SRSF2*	0.3283	0.924	0.692	3.565e-07	8.232e-03
11	CD8+ T cells	*TAF13*	0.3279	0.318	0.117	2.447e-11	5.650e-07
11	CD8+ T cells	*PLK3*	0.3273	0.144	0.031	2.263e-13	5.225e-09
11	CD8+ T cells	*LOC100345985*	0.3273	0.485	0.255	1.624e-07	3.751e-03
11	CD8+ T cells	*STK38*	0.3271	0.303	0.140	4.761e-07	1.099e-02
11	CD8+ T cells	*PACS1*	0.3262	0.326	0.132	1.314e-09	3.033e-05
11	CD8+ T cells	*ZNFX1*	0.3257	0.227	0.085	1.332e-08	3.076e-04
11	CD8+ T cells	*LOC127484098*	0.3255	0.174	0.054	3.846e-09	8.881e-05
11	CD8+ T cells	*SSH3*	0.3253	0.182	0.050	1.216e-11	2.808e-07
11	CD8+ T cells	*SESN2*	0.3253	0.159	0.015	8.221e-37	1.898e-32
11	CD8+ T cells	*RAB35*	0.3252	0.197	0.044	1.421e-16	3.282e-12
11	CD8+ T cells	*FBXO34*	0.3247	0.235	0.085	2.235e-09	5.161e-05
11	CD8+ T cells	*CHCHD2*	0.3243	0.856	0.611	3.362e-08	7.763e-04
11	CD8+ T cells	*ADGRE5*	0.3238	0.167	0.028	1.453e-20	3.356e-16
11	CD8+ T cells	*ZC3H12D*	0.3236	0.106	0.003	1.235e-66	2.851e-62
11	CD8+ T cells	*ITGB7*	0.3232	0.197	0.029	3.305e-27	7.631e-23
11	CD8+ T cells	*AAK1*	0.3224	0.341	0.146	5.689e-09	1.314e-04
11	CD8+ T cells	*PXN*	0.3218	0.273	0.105	1.564e-09	3.612e-05
11	CD8+ T cells	*LOC138850166*	0.3213	0.212	0.057	1.879e-13	4.339e-09
11	CD8+ T cells	*ATP5MC1*	0.3209	0.432	0.224	2.647e-07	6.112e-03
11	CD8+ T cells	*SUMO2*	0.3208	0.333	0.157	2.269e-07	5.240e-03
11	CD8+ T cells	*ERCC4*	0.3204	0.129	0.033	1.944e-09	4.490e-05
11	CD8+ T cells	*LOC127486212*	0.3200	0.152	0.020	7.911e-24	1.827e-19
11	CD8+ T cells	*OGFOD3*	0.3199	0.197	0.036	4.498e-21	1.039e-16
11	CD8+ T cells	*RPS18*	0.3198	0.977	0.990	5.702e-16	1.317e-11
11	CD8+ T cells	*ITGAM*	0.3196	0.174	0.009	3.676e-70	8.490e-66
11	CD8+ T cells	*TEX2*	0.3192	0.235	0.081	6.247e-10	1.443e-05
11	CD8+ T cells	*PRORP*	0.3187	0.280	0.118	6.286e-08	1.452e-03
11	CD8+ T cells	*LOC138844589*	0.3182	0.485	0.243	3.926e-08	9.065e-04
11	CD8+ T cells	*RCC2*	0.3180	0.197	0.048	9.892e-15	2.284e-10
11	CD8+ T cells	*ZNF398*	0.3177	0.197	0.064	2.889e-09	6.671e-05
11	CD8+ T cells	*UQCRC1*	0.3175	0.295	0.125	2.746e-08	6.342e-04
11	CD8+ T cells	*CCDC88B*	0.3174	0.144	0.005	6.524e-75	1.506e-70
11	CD8+ T cells	*AP3S1*	0.3168	0.386	0.185	6.714e-08	1.550e-03
11	CD8+ T cells	*EML2*	0.3162	0.212	0.076	1.624e-08	3.750e-04
11	CD8+ T cells	*MAP3K3*	0.3160	0.417	0.219	6.930e-07	1.600e-02
11	CD8+ T cells	*LOC138842984*	0.3157	0.159	0.003	3.424e-138	7.906e-134
11	CD8+ T cells	*SNX29*	0.3151	0.250	0.091	3.264e-09	7.537e-05
11	CD8+ T cells	*AHR*	0.3144	0.121	0.034	8.415e-08	1.943e-03

Original cluster	Annotated cell type	Gene	avg_log2FC	pct.1	pct.2	Raw P value	Bonferroni-adjusted P value
11	CD8+ T cells	*FBXO42*	0.3140	0.273	0.122	5.020e-07	1.159e-02
11	CD8+ T cells	*CAAP1*	0.3140	0.220	0.079	8.088e-09	1.868e-04
11	CD8+ T cells	*OVGP1*	0.3139	0.114	0.001	8.230e-170	1.901e-165
11	CD8+ T cells	*PPTC7*	0.3139	0.227	0.053	3.048e-17	7.038e-13
11	CD8+ T cells	*PSMD14*	0.3139	0.235	0.080	9.975e-10	2.303e-05
11	CD8+ T cells	*NAGPA*	0.3137	0.258	0.094	9.364e-10	2.162e-05
11	CD8+ T cells	*BAZ2A*	0.3133	0.265	0.102	3.532e-09	8.156e-05
11	CD8+ T cells	*RAB11FIP4*	0.3133	0.136	0.005	9.901e-72	2.286e-67
11	CD8+ T cells	*DCLK2*	0.3132	0.144	0.029	2.567e-14	5.928e-10
11	CD8+ T cells	*GALNT10*	0.3129	0.174	0.048	2.879e-11	6.647e-07
11	CD8+ T cells	*TRIM5*	0.3128	0.182	0.034	3.169e-19	7.318e-15
11	CD8+ T cells	*RNASEH2C*	0.3127	0.227	0.085	2.089e-08	4.825e-04
11	CD8+ T cells	*CPOX*	0.3123	0.189	0.063	1.333e-08	3.077e-04
11	CD8+ T cells	*MTMR1*	0.3123	0.212	0.069	4.891e-10	1.129e-05
11	CD8+ T cells	*NMT2*	0.3123	0.364	0.147	3.535e-10	8.163e-06
11	CD8+ T cells	*ZBTB11*	0.3117	0.303	0.124	5.527e-09	1.276e-04
11	CD8+ T cells	*EHD1*	0.3116	0.311	0.134	3.370e-08	7.781e-04
11	CD8+ T cells	*ADRM1*	0.3114	0.311	0.142	2.495e-07	5.760e-03
11	CD8+ T cells	*RFFL*	0.3111	0.242	0.093	2.509e-08	5.793e-04
11	CD8+ T cells	*ATAD2B*	0.3110	0.583	0.337	4.770e-07	1.101e-02
11	CD8+ T cells	*SSU72*	0.3109	0.712	0.436	4.229e-07	9.765e-03
11	CD8+ T cells	*MCU*	0.3109	0.394	0.200	7.072e-07	1.633e-02
11	CD8+ T cells	*AOAH*	0.3109	0.114	0.016	1.109e-17	2.561e-13
11	CD8+ T cells	*TFRC*	0.3106	0.212	0.071	1.536e-09	3.547e-05
11	CD8+ T cells	*ADA*	0.3105	0.152	0.025	7.372e-19	1.702e-14
11	CD8+ T cells	*RPS7*	0.3103	0.985	0.988	1.024e-11	2.364e-07
11	CD8+ T cells	*ITGA6*	0.3102	0.197	0.053	1.292e-12	2.984e-08
11	CD8+ T cells	*LOC100340154*	0.3102	0.144	0.043	1.426e-08	3.293e-04
11	CD8+ T cells	*AKIRIN2*	0.3098	0.318	0.153	1.376e-06	3.178e-02
11	CD8+ T cells	*TSC22D4*	0.3098	0.280	0.107	2.191e-09	5.060e-05
11	CD8+ T cells	*LOC127492663*	0.3091	0.167	0.022	8.648e-27	1.997e-22
11	CD8+ T cells	*PCCA*	0.3091	0.326	0.152	3.835e-07	8.855e-03
11	CD8+ T cells	*MEF2D*	0.3091	0.205	0.080	2.760e-07	6.374e-03
11	CD8+ T cells	*WAPL*	0.3080	0.447	0.226	5.742e-08	1.326e-03
11	CD8+ T cells	*TMEM131L*	0.3080	0.280	0.119	8.906e-08	2.057e-03
11	CD8+ T cells	*IQGAP2*	0.3078	0.167	0.020	4.583e-30	1.058e-25
11	CD8+ T cells	*ARIH1*	0.3073	0.492	0.271	1.084e-06	2.504e-02
11	CD8+ T cells	*KLRG1*	0.3066	0.114	0.000	4.453e-189	1.028e-184
11	CD8+ T cells	*DNAJB11*	0.3065	0.303	0.122	5.025e-09	1.160e-04
11	CD8+ T cells	*SELENOI*	0.3063	0.152	0.018	6.491e-28	1.499e-23

Original cluster	Annotated cell type	Gene	avg_log2FC	pct.1	pct.2	Raw P value	Bonferroni-adjusted P value
11	CD8+ T cells	*TOPORS*	0.3059	0.447	0.237	3.759e-07	8.680e-03
11	CD8+ T cells	*SLC16A10*	0.3052	0.144	0.024	1.331e-17	3.075e-13
11	CD8+ T cells	*LMNB1*	0.3048	0.205	0.054	2.462e-13	5.686e-09
11	CD8+ T cells	*MAPK6*	0.3044	0.303	0.125	9.396e-09	2.170e-04
11	CD8+ T cells	*SPAG1*	0.3041	0.167	0.049	1.071e-09	2.474e-05
11	CD8+ T cells	*CISD3*	0.3039	0.250	0.100	6.129e-08	1.415e-03
11	CD8+ T cells	*UPB1*	0.3038	0.144	0.003	5.421e-127	1.252e-122
11	CD8+ T cells	*TMEM156*	0.3035	0.144	0.002	3.317e-158	7.660e-154
11	CD8+ T cells	*HIPK1*	0.3033	0.402	0.210	1.009e-06	2.329e-02
11	CD8+ T cells	*MMS22L*	0.3032	0.129	0.019	3.443e-18	7.950e-14
11	CD8+ T cells	*RETN*	0.3028	0.152	0.010	2.080e-51	4.802e-47
11	CD8+ T cells	*NSMCE4A*	0.3026	0.280	0.099	5.816e-11	1.343e-06
11	CD8+ T cells	*CORO7*	0.3025	0.129	0.008	4.337e-43	1.002e-38
11	CD8+ T cells	*TET3*	0.3020	0.197	0.047	3.939e-15	9.095e-11
11	CD8+ T cells	*RNF14*	0.3018	0.242	0.093	1.584e-08	3.657e-04
11	CD8+ T cells	*MAST3*	0.3018	0.167	0.025	1.490e-22	3.441e-18
11	CD8+ T cells	*R3HDM4*	0.3017	0.212	0.075	1.357e-08	3.134e-04
11	CD8+ T cells	*ARFIP1*	0.3015	0.356	0.176	6.742e-07	1.557e-02
11	CD8+ T cells	*MIEN1*	0.3012	0.212	0.056	9.745e-14	2.250e-09
11	CD8+ T cells	*STK11*	0.3012	0.364	0.174	3.318e-07	7.662e-03
11	CD8+ T cells	*TRAF1*	0.3002	0.121	0.028	2.655e-10	6.130e-06
11	CD8+ T cells	*SCAMP2*	0.3001	0.341	0.132	1.477e-10	3.411e-06
11	CD8+ T cells	*RASSF2*	0.2997	0.182	0.036	5.571e-18	1.286e-13
11	CD8+ T cells	*BCL2L1*	0.2996	0.205	0.065	4.131e-10	9.540e-06
11	CD8+ T cells	*GFOD1*	0.2996	0.159	0.030	7.600e-17	1.755e-12
11	CD8+ T cells	*MAN1B1*	0.2994	0.212	0.088	1.527e-06	3.527e-02
11	CD8+ T cells	*ABHD17A*	0.2992	0.371	0.186	8.265e-07	1.909e-02
11	CD8+ T cells	*TICAM1*	0.2989	0.152	0.032	2.585e-14	5.969e-10
11	CD8+ T cells	*PMM2*	0.2989	0.197	0.065	3.396e-09	7.843e-05
11	CD8+ T cells	*ACTR1A*	0.2988	0.159	0.044	1.990e-10	4.594e-06
11	CD8+ T cells	*TSNAX*	0.2981	0.500	0.272	4.675e-07	1.080e-02
11	CD8+ T cells	*TRPC4AP*	0.2980	0.356	0.172	2.491e-07	5.753e-03
11	CD8+ T cells	*TRRAP*	0.2978	0.258	0.109	1.871e-07	4.319e-03
11	CD8+ T cells	*NOTCH2*	0.2971	0.576	0.329	1.700e-06	3.925e-02
11	CD8+ T cells	*MTCL1*	0.2968	0.106	0.013	1.083e-18	2.500e-14
11	CD8+ T cells	*KIAA1958*	0.2965	0.205	0.084	1.149e-06	2.653e-02
11	CD8+ T cells	*HIGD1A*	0.2963	0.197	0.046	2.226e-15	5.141e-11
11	CD8+ T cells	*TIAM2*	0.2958	0.197	0.075	3.172e-07	7.324e-03
11	CD8+ T cells	*RPN1*	0.2949	0.462	0.216	7.967e-09	1.840e-04
11	CD8+ T cells	*WDR3*	0.2946	0.197	0.078	8.787e-07	2.029e-02

Original cluster	Annotated cell type	Gene	avg_log2FC	pct.1	pct.2	Raw P value	Bonferroni-adjusted P value
11	CD8+ T cells	*LRCH4*	0.2946	0.144	0.036	5.557e-11	1.283e-06
11	CD8+ T cells	*TTC39B*	0.2945	0.273	0.090	7.301e-12	1.686e-07
11	CD8+ T cells	*GPR68*	0.2944	0.106	0.001	7.560e-148	1.746e-143
11	CD8+ T cells	*DCLRE1C*	0.2940	0.197	0.050	1.025e-13	2.367e-09
11	CD8+ T cells	*MAP2K4*	0.2931	0.424	0.199	2.924e-08	6.752e-04
11	CD8+ T cells	*ERCC8*	0.2924	0.227	0.091	1.653e-07	3.817e-03
11	CD8+ T cells	*NECAP2*	0.2919	0.333	0.131	6.270e-10	1.448e-05
11	CD8+ T cells	*PLEKHA8*	0.2919	0.205	0.066	6.014e-10	1.389e-05
11	CD8+ T cells	*LOC127493203*	0.2913	0.106	0.020	4.437e-12	1.025e-07
11	CD8+ T cells	*TTC7A*	0.2911	0.129	0.021	1.792e-16	4.139e-12
11	CD8+ T cells	*QSOX1*	0.2903	0.242	0.091	1.968e-08	4.544e-04
11	CD8+ T cells	*LOC100354197*	0.2898	0.121	0.008	1.623e-39	3.748e-35
11	CD8+ T cells	*KDM5C*	0.2894	0.197	0.074	2.122e-07	4.900e-03
11	CD8+ T cells	*LOC138845633*	0.2883	0.258	0.111	4.523e-07	1.044e-02
11	CD8+ T cells	*LOC100351389*	0.2883	0.227	0.073	1.450e-10	3.348e-06
11	CD8+ T cells	*PIK3C2B*	0.2882	0.152	0.037	1.753e-11	4.048e-07
11	CD8+ T cells	*CCT3*	0.2881	0.402	0.201	7.640e-07	1.764e-02
11	CD8+ T cells	*ENTPD3*	0.2877	0.129	0.019	4.189e-19	9.672e-15
11	CD8+ T cells	*OPTN*	0.2874	0.205	0.067	1.360e-09	3.140e-05
11	CD8+ T cells	*GAMT*	0.2873	0.318	0.134	2.908e-08	6.715e-04
11	CD8+ T cells	*DGKA*	0.2871	0.189	0.048	4.899e-13	1.131e-08
11	CD8+ T cells	*SLC1A5*	0.2863	0.189	0.059	1.553e-09	3.586e-05
11	CD8+ T cells	*HERC1*	0.2860	0.523	0.286	6.766e-07	1.562e-02
11	CD8+ T cells	*SLC7A1*	0.2859	0.136	0.034	3.821e-10	8.824e-06
11	CD8+ T cells	*RPLP0*	0.2856	0.977	0.982	7.569e-07	1.748e-02
11	CD8+ T cells	*HECTD1*	0.2855	0.432	0.210	8.192e-08	1.892e-03
11	CD8+ T cells	*RFLNB*	0.2854	0.227	0.061	2.663e-14	6.150e-10
11	CD8+ T cells	*SLC41A2*	0.2853	0.197	0.081	2.162e-06	4.992e-02
11	CD8+ T cells	*ENTREP2*	0.2851	0.106	0.012	4.212e-20	9.726e-16
11	CD8+ T cells	*CYBC1*	0.2845	0.189	0.052	1.509e-11	3.484e-07
11	CD8+ T cells	*AGPAT2*	0.2841	0.129	0.025	7.867e-14	1.817e-09
11	CD8+ T cells	*TRADD*	0.2838	0.205	0.066	1.248e-09	2.883e-05
11	CD8+ T cells	*MUS81*	0.2832	0.167	0.052	7.571e-09	1.748e-04
11	CD8+ T cells	*AURKA*	0.2825	0.106	0.005	1.631e-47	3.765e-43
11	CD8+ T cells	*LOC127493228*	0.2825	0.114	0.000	1.089e-227	2.515e-223
11	CD8+ T cells	*PLEKHA1*	0.2824	0.235	0.103	1.859e-06	4.293e-02
11	CD8+ T cells	*TMEM9B*	0.2824	0.212	0.076	1.418e-08	3.274e-04
11	CD8+ T cells	*NCBP1*	0.2822	0.189	0.049	4.323e-13	9.983e-09
11	CD8+ T cells	*BLOC1S2*	0.2822	0.235	0.096	3.256e-07	7.520e-03
11	CD8+ T cells	*CYB5B*	0.2822	0.212	0.073	3.884e-09	8.969e-05

Original cluster	Annotated cell type	Gene	avg_log2FC	pct.1	pct.2	Raw P value	Bonferroni-adjusted P value
11	CD8+ T cells	*UQCC4*	0.2821	0.348	0.164	4.057e-07	9.369e-03
11	CD8+ T cells	*VPS54*	0.2814	0.333	0.163	1.262e-06	2.915e-02
11	CD8+ T cells	*RELA*	0.2812	0.356	0.170	4.988e-07	1.152e-02
11	CD8+ T cells	*SCYL3*	0.2811	0.129	0.040	3.953e-07	9.127e-03
11	CD8+ T cells	*SELENOS*	0.2808	0.295	0.127	9.030e-08	2.085e-03
11	CD8+ T cells	*TUBB4A*	0.2804	0.136	0.012	8.401e-34	1.940e-29
11	CD8+ T cells	*TRIM14*	0.2799	0.114	0.008	2.859e-36	6.601e-32
11	CD8+ T cells	*GBF1*	0.2798	0.303	0.107	1.527e-11	3.526e-07
11	CD8+ T cells	*SLC23A2*	0.2794	0.189	0.074	1.136e-06	2.623e-02
11	CD8+ T cells	*NCF4*	0.2793	0.106	0.003	4.368e-72	1.009e-67
11	CD8+ T cells	*ADGRB1*	0.2789	0.114	0.003	3.090e-87	7.136e-83
11	CD8+ T cells	*GSK3A*	0.2785	0.303	0.125	2.277e-08	5.259e-04
11	CD8+ T cells	*GTF2H5*	0.2785	0.394	0.199	8.723e-07	2.014e-02
11	CD8+ T cells	*DCP2*	0.2783	0.235	0.096	2.265e-07	5.229e-03
11	CD8+ T cells	*DIABLO*	0.2782	0.348	0.172	1.626e-06	3.754e-02
11	CD8+ T cells	*TTI2*	0.2775	0.182	0.056	1.391e-09	3.212e-05
11	CD8+ T cells	*RPL12*	0.2761	0.985	0.987	2.617e-07	6.043e-03
11	CD8+ T cells	*MLH3*	0.2759	0.220	0.076	7.860e-09	1.815e-04
11	CD8+ T cells	*RALB*	0.2755	0.303	0.122	6.156e-09	1.421e-04
11	CD8+ T cells	*ZMYND19*	0.2742	0.189	0.044	9.967e-15	2.302e-10
11	CD8+ T cells	*DR1*	0.2740	0.250	0.112	1.441e-06	3.327e-02
11	CD8+ T cells	*ACOT7*	0.2733	0.129	0.012	1.604e-31	3.703e-27
11	CD8+ T cells	*RAP2B*	0.2730	0.326	0.144	1.110e-07	2.563e-03
11	CD8+ T cells	*SH2B3*	0.2729	0.159	0.035	3.960e-14	9.144e-10
11	CD8+ T cells	*UBTF*	0.2728	0.371	0.178	5.270e-07	1.217e-02
11	CD8+ T cells	*C1H11orf16*	0.2726	0.121	0.003	1.928e-83	4.451e-79
11	CD8+ T cells	*CISH*	0.2716	0.189	0.074	8.395e-07	1.939e-02
11	CD8+ T cells	*ZFYVE27*	0.2714	0.167	0.032	7.545e-17	1.742e-12
11	CD8+ T cells	*NUBP2*	0.2712	0.174	0.044	2.042e-12	4.716e-08
11	CD8+ T cells	*STK38L*	0.2710	0.197	0.079	7.436e-07	1.717e-02
11	CD8+ T cells	*EIF3B*	0.2709	0.258	0.114	9.603e-07	2.218e-02
11	CD8+ T cells	*PLCG1*	0.2697	0.220	0.076	2.999e-09	6.926e-05
11	CD8+ T cells	*LOC103350039*	0.2696	0.114	0.004	6.267e-67	1.447e-62
11	CD8+ T cells	*DENND11*	0.2695	0.144	0.021	2.660e-20	6.142e-16
11	CD8+ T cells	*THOC3*	0.2692	0.152	0.046	1.872e-08	4.324e-04
11	CD8+ T cells	*BID*	0.2688	0.197	0.047	1.469e-14	3.393e-10
11	CD8+ T cells	*SGK3*	0.2680	0.288	0.130	5.515e-07	1.274e-02
11	CD8+ T cells	*ERICH1*	0.2679	0.189	0.072	6.386e-07	1.475e-02
11	CD8+ T cells	*SEMA7A*	0.2675	0.114	0.007	5.426e-38	1.253e-33
11	CD8+ T cells	*LOC103347630*	0.2675	0.106	0.000	2.994e-198	6.913e-194

Original cluster	Annotated cell type	Gene	avg_log2FC	pct.1	pct.2	Raw P value	Bonferroni-adjusted P value
11	CD8+ T cells	*IFIH1*	0.2672	0.212	0.063	4.725e-11	1.091e-06
11	CD8+ T cells	*SDE2*	0.2670	0.326	0.142	6.944e-08	1.603e-03
11	CD8+ T cells	*AIP*	0.2662	0.212	0.082	2.411e-07	5.567e-03
11	CD8+ T cells	*LOC138849008*	0.2658	0.106	0.013	1.712e-18	3.953e-14
11	CD8+ T cells	*TSSC4*	0.2652	0.167	0.035	2.257e-15	5.212e-11
11	CD8+ T cells	*LDHA*	0.2650	0.220	0.052	4.067e-16	9.393e-12
11	CD8+ T cells	*EMC8*	0.2646	0.258	0.095	2.946e-09	6.803e-05
11	CD8+ T cells	*CDC34*	0.2645	0.356	0.171	6.874e-07	1.587e-02
11	CD8+ T cells	*IRF3*	0.2644	0.326	0.155	1.193e-06	2.754e-02
11	CD8+ T cells	*WDR1*	0.2644	0.295	0.130	1.260e-07	2.909e-03
11	CD8+ T cells	*DESI1*	0.2644	0.197	0.070	5.740e-08	1.326e-03
11	CD8+ T cells	*LOC103345057*	0.2642	0.121	0.005	3.538e-61	8.169e-57
11	CD8+ T cells	*THEMIS2*	0.2639	0.106	0.010	2.135e-25	4.930e-21
11	CD8+ T cells	*CCDC92*	0.2639	0.114	0.006	2.723e-44	6.289e-40
11	CD8+ T cells	*DGKE*	0.2639	0.189	0.051	4.862e-12	1.123e-07
11	CD8+ T cells	*CALHM2*	0.2637	0.167	0.060	5.399e-07	1.247e-02
11	CD8+ T cells	*A4GALT*	0.2633	0.121	0.002	2.199e-102	5.077e-98
11	CD8+ T cells	*NFKBIE*	0.2631	0.129	0.016	4.711e-22	1.088e-17
11	CD8+ T cells	*CHORDC1*	0.2629	0.212	0.075	9.194e-09	2.123e-04
11	CD8+ T cells	*ADPRH*	0.2628	0.174	0.035	9.048e-17	2.089e-12
11	CD8+ T cells	*WDR47*	0.2628	0.265	0.114	4.322e-07	9.981e-03
11	CD8+ T cells	*LOC103348363*	0.2623	0.136	0.009	1.864e-45	4.304e-41
11	CD8+ T cells	*VPS36*	0.2618	0.356	0.150	6.419e-09	1.482e-04
11	CD8+ T cells	*MRM1*	0.2615	0.144	0.029	5.015e-14	1.158e-09
11	CD8+ T cells	*TRABD*	0.2610	0.189	0.062	6.216e-09	1.435e-04
11	CD8+ T cells	*LIG3*	0.2608	0.189	0.051	4.220e-12	9.745e-08
11	CD8+ T cells	*KBTBD2*	0.2604	0.280	0.127	8.877e-07	2.050e-02
11	CD8+ T cells	*LOC138849403*	0.2603	0.114	0.010	2.740e-29	6.327e-25
11	CD8+ T cells	*TRIM24*	0.2599	0.265	0.113	2.781e-07	6.421e-03
11	CD8+ T cells	*METTL21A*	0.2595	0.159	0.044	6.046e-10	1.396e-05
11	CD8+ T cells	*LRRC47*	0.2593	0.379	0.187	1.386e-06	3.200e-02
11	CD8+ T cells	*RPS10*	0.2593	0.977	0.982	1.542e-07	3.561e-03
11	CD8+ T cells	*TRNT1*	0.2590	0.174	0.058	3.772e-08	8.711e-04
11	CD8+ T cells	*INTS13*	0.2589	0.250	0.104	3.064e-07	7.076e-03
11	CD8+ T cells	*PEDS1*	0.2585	0.121	0.029	1.052e-09	2.428e-05
11	CD8+ T cells	*LOC100328959*	0.2578	0.121	0.025	1.667e-11	3.849e-07
11	CD8+ T cells	*SUSD4*	0.2578	0.121	0.002	2.227e-102	5.143e-98
11	CD8+ T cells	*SLC45A4*	0.2572	0.136	0.015	2.285e-26	5.276e-22
11	CD8+ T cells	*MSMO1*	0.2571	0.220	0.065	1.463e-11	3.379e-07
11	CD8+ T cells	*SLC35D2*	0.2566	0.144	0.021	2.297e-20	5.305e-16

Original cluster	Annotated cell type	Gene	avg_log2FC	pct.1	pct.2	Raw P value	Bonferroni-adjusted P value
11	CD8+ T cells	*TFIP11*	0.2557	0.136	0.038	1.224e-08	2.827e-04
11	CD8+ T cells	*CCNY*	0.2556	0.227	0.092	3.239e-07	7.480e-03
11	CD8+ T cells	*CENPX*	0.2552	0.364	0.181	1.522e-06	3.515e-02
11	CD8+ T cells	*RINL*	0.2551	0.121	0.001	6.897e-184	1.593e-179
11	CD8+ T cells	*ORAI3*	0.2550	0.197	0.064	2.240e-09	5.172e-05
11	CD8+ T cells	*MANBA*	0.2548	0.136	0.023	9.259e-17	2.138e-12
11	CD8+ T cells	*LOC138843200*	0.2536	0.136	0.025	4.169e-15	9.628e-11
11	CD8+ T cells	*PFKP*	0.2532	0.212	0.083	6.361e-07	1.469e-02
11	CD8+ T cells	*PPP6C*	0.2529	0.250	0.101	1.273e-07	2.939e-03
11	CD8+ T cells	*SGSM3*	0.2529	0.265	0.103	1.626e-08	3.754e-04
11	CD8+ T cells	*ACLY*	0.2528	0.212	0.081	1.782e-07	4.116e-03
11	CD8+ T cells	*ANKRD27*	0.2523	0.189	0.059	1.661e-09	3.837e-05
11	CD8+ T cells	*EFHD2*	0.2518	0.258	0.113	1.243e-06	2.871e-02
11	CD8+ T cells	*ARIH2*	0.2516	0.311	0.145	9.065e-07	2.093e-02
11	CD8+ T cells	*CNIH1*	0.2515	0.265	0.114	7.006e-07	1.618e-02
11	CD8+ T cells	*MADD*	0.2515	0.167	0.043	1.337e-11	3.088e-07
11	CD8+ T cells	*AMPD2*	0.2510	0.136	0.022	1.953e-17	4.511e-13
11	CD8+ T cells	*MAK16*	0.2508	0.220	0.072	7.064e-10	1.631e-05
11	CD8+ T cells	*SLC25A13*	0.2502	0.167	0.059	5.038e-07	1.163e-02
13	**Lymphatic endothelial cells**	*CCL21*	7.5334	0.985	0.008	0	0
13	Lymphatic endothelial cells	*MMRN1*	5.2994	0.985	0.004	0	0
13	Lymphatic endothelial cells	*CLDN5*	4.8505	1.000	0.050	5.445e-272	1.257e-267
13	Lymphatic endothelial cells	*LYVE1*	4.5452	0.940	0.011	0	0
13	Lymphatic endothelial cells	*RAMP2*	4.4152	1.000	0.215	2.694e-83	6.222e-79
13	Lymphatic endothelial cells	*PROX1*	4.0421	0.970	0.008	0	0
13	Lymphatic endothelial cells	*GNG11*	3.7470	0.985	0.356	3.451e-56	7.969e-52
13	Lymphatic endothelial cells	*RELN*	3.5483	0.896	0.048	2.885e-220	6.663e-216
13	Lymphatic endothelial cells	*LOC100008716*	3.4643	0.910	0.001	0	0
13	Lymphatic endothelial cells	*CAVIN2*	3.4450	0.955	0.052	3.284e-233	7.584e-229
13	Lymphatic endothelial cells	*FGL2*	3.3958	0.940	0.172	8.697e-83	2.008e-78
13	Lymphatic endothelial cells	*APOD*	3.1908	0.866	0.075	2.593e-141	5.988e-137
13	Lymphatic endothelial cells	*KCTD12*	3.0965	1.000	0.286	6.772e-66	1.564e-61
13	Lymphatic endothelial cells	*THY1*	3.0315	0.985	0.296	3.239e-61	7.480e-57
13	Lymphatic endothelial cells	*FLT4*	2.9930	0.896	0.014	0	0
13	Lymphatic endothelial cells	*EGFL7*	2.8967	0.940	0.040	5.725e-267	1.322e-262
13	Lymphatic endothelial cells	*ECSCR*	2.8940	0.910	0.030	0	0
13	Lymphatic endothelial cells	*CRACR2B*	2.8802	0.896	0.038	5.111e-266	1.180e-261
13	Lymphatic endothelial cells	*STAB1*	2.7843	0.791	0.012	0	0
13	Lymphatic endothelial cells	*IGFBP7*	2.7090	1.000	0.516	3.339e-44	7.711e-40
13	Lymphatic endothelial cells	*MAGI1*	2.7042	0.836	0.130	4.527e-74	1.045e-69

Original cluster	Annotated cell type	Gene	avg_log2FC	pct.1	pct.2	Raw P value	Bonferroni-adjusted P value
13	Lymphatic endothelial cells	*PTX3*	2.6941	0.701	0.023	1.028e-252	2.375e-248
13	Lymphatic endothelial cells	*TBX1*	2.6747	0.910	0.001	0	0
13	Lymphatic endothelial cells	*PDE4D*	2.5706	0.836	0.178	8.880e-55	2.051e-50
13	Lymphatic endothelial cells	*STON2*	2.5425	0.836	0.035	7.288e-250	1.683e-245
13	Lymphatic endothelial cells	*KLHL4*	2.5146	0.776	0.014	0	0
13	Lymphatic endothelial cells	*GRO-A*	2.4839	0.552	0.038	4.804e-99	1.109e-94
13	Lymphatic endothelial cells	*VIM*	2.4604	1.000	0.934	1.181e-41	2.728e-37
13	Lymphatic endothelial cells	*RHOJ*	2.4485	0.910	0.225	5.948e-58	1.374e-53
13	Lymphatic endothelial cells	*S100A16*	2.4474	0.955	0.266	9.599e-61	2.217e-56
13	Lymphatic endothelial cells	*KLF6*	2.4212	0.940	0.500	1.616e-35	3.731e-31
13	Lymphatic endothelial cells	*FXYD5*	2.4096	0.910	0.041	3.948e-250	9.116e-246
13	Lymphatic endothelial cells	*KLF2*	2.3834	0.940	0.485	2.048e-28	4.729e-24
13	Lymphatic endothelial cells	*TMSB10*	2.3823	1.000	0.960	4.216e-40	9.735e-36
13	Lymphatic endothelial cells	*MPP7*	2.3780	0.836	0.074	2.085e-128	4.814e-124
13	Lymphatic endothelial cells	*TGFBR2*	2.3769	0.866	0.234	2.112e-52	4.877e-48
13	Lymphatic endothelial cells	*TSPAN18*	2.3579	0.851	0.029	4.547e-301	1.050e-296
13	Lymphatic endothelial cells	*NR2F1*	2.3370	0.896	0.388	1.756e-38	4.055e-34
13	Lymphatic endothelial cells	*CCN5*	2.3173	0.716	0.012	0	0
13	Lymphatic endothelial cells	*FAM174B*	2.2907	0.866	0.049	3.815e-198	8.809e-194
13	Lymphatic endothelial cells	*ELK3*	2.2874	0.821	0.115	1.002e-84	2.314e-80
13	Lymphatic endothelial cells	*DOCK9*	2.2731	0.910	0.200	3.404e-61	7.861e-57
13	Lymphatic endothelial cells	*PPFIBP1*	2.2457	0.836	0.169	2.997e-59	6.920e-55
13	Lymphatic endothelial cells	*ANXA2*	2.2455	0.925	0.170	1.760e-74	4.064e-70
13	Lymphatic endothelial cells	*CRIP2*	2.2250	0.851	0.086	1.115e-114	2.574e-110
13	Lymphatic endothelial cells	*ARHGAP29*	2.2156	0.985	0.467	4.742e-45	1.095e-40
13	Lymphatic endothelial cells	*ARL4A*	2.2127	0.970	0.290	7.869e-54	1.817e-49
13	Lymphatic endothelial cells	*TFPI*	2.2082	0.881	0.222	1.738e-54	4.014e-50
13	Lymphatic endothelial cells	*LMO2*	2.1939	0.791	0.050	3.443e-158	7.951e-154
13	Lymphatic endothelial cells	*GALNT1*	2.1918	0.925	0.289	3.436e-50	7.934e-46
13	Lymphatic endothelial cells	*AKAP12*	2.1846	0.896	0.348	6.305e-34	1.456e-29
13	Lymphatic endothelial cells	*SOX18*	2.1793	0.776	0.034	1.538e-213	3.552e-209
13	Lymphatic endothelial cells	*CPNE8*	2.1725	0.910	0.159	6.855e-74	1.583e-69
13	Lymphatic endothelial cells	*CLEC3B*	2.1522	0.821	0.254	3.994e-33	9.223e-29
13	Lymphatic endothelial cells	*MAF*	2.1320	0.866	0.246	6.523e-44	1.506e-39
13	Lymphatic endothelial cells	*SGK1*	2.1306	0.806	0.158	2.670e-52	6.166e-48
13	Lymphatic endothelial cells	*S1PR1*	2.1262	0.776	0.021	0	0
13	Lymphatic endothelial cells	*PDE2A*	2.1182	0.791	0.024	0	0
13	Lymphatic endothelial cells	*MFNG*	2.0821	0.821	0.043	1.562e-200	3.607e-196
13	Lymphatic endothelial cells	*PLVAP*	2.0810	0.627	0.028	1.037e-165	2.395e-161
13	Lymphatic endothelial cells	*PLAAT3*	2.0601	0.836	0.158	2.321e-59	5.361e-55

Original cluster	Annotated cell type	Gene	avg_log2FC	pct.1	pct.2	Raw P value	Bonferroni-adjusted P value
13	Lymphatic endothelial cells	*LOC103351679*	2.0544	0.896	0.084	4.934e-114	1.139e-109
13	Lymphatic endothelial cells	*KANK3*	2.0437	0.866	0.032	2.707e-278	6.252e-274
13	Lymphatic endothelial cells	*TSPAN13*	2.0423	0.836	0.150	9.485e-66	2.190e-61
13	Lymphatic endothelial cells	*PECAM1*	2.0324	0.821	0.044	1.174e-189	2.711e-185
13	Lymphatic endothelial cells	*WWTR1*	2.0317	0.866	0.335	9.631e-35	2.224e-30
13	Lymphatic endothelial cells	*NRP2*	2.0179	0.657	0.091	8.906e-62	2.057e-57
13	Lymphatic endothelial cells	*TSPAN5*	2.0092	0.791	0.093	1.858e-89	4.290e-85
13	Lymphatic endothelial cells	*KDR*	1.9745	0.761	0.034	9.206e-209	2.126e-204
13	Lymphatic endothelial cells	*GYPC*	1.9601	0.910	0.371	1.066e-39	2.462e-35
13	Lymphatic endothelial cells	*CD9*	1.9418	0.791	0.076	2.991e-106	6.906e-102
13	Lymphatic endothelial cells	*SCN3A*	1.9246	0.612	0.000	0	0
13	Lymphatic endothelial cells	*GPR182*	1.9193	0.687	0.013	0	0
13	Lymphatic endothelial cells	*CDH5*	1.8676	0.761	0.031	1.924e-221	4.443e-217
13	Lymphatic endothelial cells	*CYB5R3*	1.8592	0.940	0.334	2.528e-42	5.837e-38
13	Lymphatic endothelial cells	*GMDS*	1.8330	0.806	0.149	1.325e-56	3.060e-52
13	Lymphatic endothelial cells	*HS3ST1*	1.8256	0.746	0.037	4.630e-188	1.069e-183
13	Lymphatic endothelial cells	*CTNNAL1*	1.8202	0.761	0.108	6.172e-70	1.425e-65
13	Lymphatic endothelial cells	*ABI3BP*	1.8193	0.896	0.224	3.430e-46	7.920e-42
13	Lymphatic endothelial cells	*BSG*	1.8107	1.000	0.709	2.179e-36	5.031e-32
13	Lymphatic endothelial cells	*GJA1*	1.8107	0.701	0.106	3.435e-61	7.932e-57
13	Lymphatic endothelial cells	*DAB2*	1.7912	0.731	0.110	9.290e-64	2.145e-59
13	Lymphatic endothelial cells	*SEMA6A*	1.7826	0.791	0.088	3.686e-93	8.513e-89
13	Lymphatic endothelial cells	*PDPN*	1.7813	0.642	0.004	0	0
13	Lymphatic endothelial cells	*FZD6*	1.7743	0.746	0.067	1.786e-107	4.123e-103
13	Lymphatic endothelial cells	*CHST7*	1.7717	0.746	0.087	2.899e-84	6.694e-80
13	Lymphatic endothelial cells	*LOC103345647*	1.7645	0.836	0.212	9.698e-47	2.240e-42
13	Lymphatic endothelial cells	*ETS1*	1.7458	0.821	0.118	1.642e-72	3.791e-68
13	Lymphatic endothelial cells	*SMAD1*	1.7436	0.821	0.200	1.971e-43	4.551e-39
13	Lymphatic endothelial cells	*FYB2*	1.7045	0.582	0.002	0	0
13	Lymphatic endothelial cells	*DKK3*	1.7039	0.582	0.053	6.771e-82	1.564e-77
13	Lymphatic endothelial cells	*PRKCZ*	1.6975	0.597	0.008	0	0
13	Lymphatic endothelial cells	*MARCKSL1*	1.6964	0.881	0.506	2.343e-27	5.411e-23
13	Lymphatic endothelial cells	*EGLN3*	1.6688	0.657	0.033	5.770e-160	1.332e-155
13	Lymphatic endothelial cells	*SELENOW*	1.6460	0.955	0.472	1.846e-32	4.263e-28
13	Lymphatic endothelial cells	*SH3BGRL3*	1.6406	0.836	0.210	1.586e-44	3.663e-40
13	Lymphatic endothelial cells	*FNDC4*	1.6310	0.642	0.048	2.262e-110	5.223e-106
13	Lymphatic endothelial cells	*APLP2*	1.6163	0.970	0.677	2.989e-32	6.902e-28
13	Lymphatic endothelial cells	*S100A10*	1.5993	0.881	0.348	1.709e-31	3.947e-27
13	Lymphatic endothelial cells	*MYZAP*	1.5929	0.582	0.067	1.323e-64	3.055e-60
13	Lymphatic endothelial cells	*EPB41L2*	1.5796	0.896	0.324	2.021e-33	4.668e-29

Original cluster	Annotated cell type	Gene	avg_log2FC	pct.1	pct.2	Raw P value	Bonferroni-adjusted P value
13	Lymphatic endothelial cells	*GNAS*	1.5712	1.000	0.881	6.250e-33	1.443e-28
13	Lymphatic endothelial cells	*ITIH5*	1.5588	0.567	0.098	3.556e-40	8.212e-36
13	Lymphatic endothelial cells	*PCSK6*	1.5581	0.731	0.100	2.083e-69	4.810e-65
13	Lymphatic endothelial cells	*DUSP6*	1.5455	0.507	0.025	3.293e-127	7.604e-123
13	Lymphatic endothelial cells	*PARD6G*	1.5416	0.612	0.024	7.032e-187	1.624e-182
13	Lymphatic endothelial cells	*MPZL2*	1.5393	0.552	0.053	1.946e-72	4.493e-68
13	Lymphatic endothelial cells	*FOXC1*	1.5362	0.597	0.031	5.227e-143	1.207e-138
13	Lymphatic endothelial cells	*ARHGAP18*	1.5294	0.597	0.091	8.849e-49	2.044e-44
13	Lymphatic endothelial cells	*SPTBN1*	1.5206	0.896	0.660	1.117e-20	2.579e-16
13	Lymphatic endothelial cells	*CARHSP1*	1.5171	0.672	0.131	5.597e-43	1.292e-38
13	Lymphatic endothelial cells	*GNG12*	1.5135	0.687	0.106	1.714e-55	3.957e-51
13	Lymphatic endothelial cells	*LOC127482786*	1.5107	0.582	0.028	1.455e-147	3.360e-143
13	Lymphatic endothelial cells	*TLR4*	1.5084	0.672	0.029	6.014e-190	1.389e-185
13	Lymphatic endothelial cells	*PODXL*	1.5083	0.642	0.032	1.515e-155	3.499e-151
13	Lymphatic endothelial cells	*IL7*	1.5072	0.522	0.005	0	0
13	Lymphatic endothelial cells	*CALHM2*	1.5061	0.657	0.057	4.326e-95	9.990e-91
13	Lymphatic endothelial cells	*CD200*	1.5042	0.731	0.174	5.482e-38	1.266e-33
13	Lymphatic endothelial cells	*ELAPOR2*	1.4963	0.597	0.010	0	0
13	Lymphatic endothelial cells	*TF*	1.4887	0.627	0.040	4.512e-121	1.042e-116
13	Lymphatic endothelial cells	*PPP1R9A*	1.4866	0.701	0.193	1.232e-28	2.845e-24
13	Lymphatic endothelial cells	*TANC2*	1.4789	0.627	0.186	1.881e-23	4.344e-19
13	Lymphatic endothelial cells	*STXBP6*	1.4732	0.672	0.117	3.862e-46	8.918e-42
13	Lymphatic endothelial cells	*TCN2*	1.4712	0.672	0.151	2.209e-36	5.102e-32
13	Lymphatic endothelial cells	*PDE7B*	1.4688	0.537	0.064	4.394e-54	1.015e-49
13	Lymphatic endothelial cells	*LOC138848040*	1.4676	0.985	0.899	1.506e-18	3.478e-14
13	Lymphatic endothelial cells	*EPS15*	1.4629	0.761	0.286	7.430e-24	1.716e-19
13	Lymphatic endothelial cells	*GJA4*	1.4612	0.463	0.032	5.902e-82	1.363e-77
13	Lymphatic endothelial cells	*PPA1*	1.4576	0.612	0.059	1.589e-80	3.670e-76
13	Lymphatic endothelial cells	*GNGT2*	1.4557	0.582	0.008	0	0
13	Lymphatic endothelial cells	*CALM1*	1.4468	0.970	0.746	3.050e-29	7.044e-25
13	Lymphatic endothelial cells	*TPM4*	1.4438	0.806	0.346	9.759e-22	2.253e-17
13	Lymphatic endothelial cells	*ENG*	1.4438	0.701	0.090	7.002e-67	1.617e-62
13	Lymphatic endothelial cells	*SEPTIN6*	1.4401	0.657	0.084	2.158e-64	4.984e-60
13	Lymphatic endothelial cells	*EFNA1*	1.4340	0.612	0.056	2.236e-84	5.163e-80
13	Lymphatic endothelial cells	*MDFIC*	1.4331	0.791	0.264	2.561e-26	5.914e-22
13	Lymphatic endothelial cells	*DYSF*	1.4319	0.627	0.020	9.802e-228	2.264e-223
13	Lymphatic endothelial cells	*BLVRB*	1.4313	0.821	0.255	1.607e-33	3.712e-29
13	Lymphatic endothelial cells	*MSN*	1.4235	0.746	0.205	2.230e-33	5.149e-29
13	Lymphatic endothelial cells	*TMSB4X*	1.4213	1.000	0.975	2.067e-35	4.773e-31
13	Lymphatic endothelial cells	*CAV1*	1.4171	0.896	0.247	5.754e-35	1.329e-30

Original cluster	Annotated cell type	Gene	avg_log2FC	pct.1	pct.2	Raw P value	Bonferroni-adjusted P value
13	Lymphatic endothelial cells	*MTCH1*	1.4115	0.970	0.599	1.042e-26	2.407e-22
13	Lymphatic endothelial cells	*ZNF521*	1.4096	0.687	0.236	1.685e-21	3.892e-17
13	Lymphatic endothelial cells	*SNCG*	1.4093	0.567	0.048	1.755e-79	4.053e-75
13	Lymphatic endothelial cells	*LOC100354804*	1.4083	0.358	0.023	1.137e-66	2.626e-62
13	Lymphatic endothelial cells	*LGMN*	1.4069	0.821	0.343	2.276e-24	5.256e-20
13	Lymphatic endothelial cells	*FCGRT*	1.4041	0.940	0.580	1.967e-25	4.543e-21
13	Lymphatic endothelial cells	*PTPN14*	1.4021	0.716	0.106	3.071e-58	7.091e-54
13	Lymphatic endothelial cells	*RTN4RL2*	1.3957	0.597	0.007	0	0
13	Lymphatic endothelial cells	*N4BP3*	1.3941	0.567	0.021	4.494e-182	1.038e-177
13	Lymphatic endothelial cells	*CYP4B1*	1.3916	0.507	0.003	0	0
13	Lymphatic endothelial cells	*DHCR24*	1.3911	0.567	0.010	0	0
13	Lymphatic endothelial cells	*LIMCH1*	1.3824	0.657	0.117	1.065e-43	2.460e-39
13	Lymphatic endothelial cells	*CAVIN1*	1.3797	0.821	0.254	3.999e-31	9.235e-27
13	Lymphatic endothelial cells	*ADD3*	1.3768	0.821	0.455	8.009e-18	1.849e-13
13	Lymphatic endothelial cells	*JUP*	1.3684	0.672	0.128	1.574e-41	3.635e-37
13	Lymphatic endothelial cells	*ERG*	1.3670	0.522	0.040	5.965e-83	1.377e-78
13	Lymphatic endothelial cells	*S100A13*	1.3651	0.746	0.311	1.428e-19	3.298e-15
13	Lymphatic endothelial cells	*CHP2*	1.3596	0.522	0.016	7.374e-197	1.703e-192
13	Lymphatic endothelial cells	*DAPK1*	1.3566	0.582	0.052	6.286e-83	1.452e-78
13	Lymphatic endothelial cells	*RASIP1*	1.3528	0.537	0.029	5.676e-122	1.311e-117
13	Lymphatic endothelial cells	*NOTCH1*	1.3516	0.627	0.056	1.572e-87	3.631e-83
13	Lymphatic endothelial cells	*TPST2*	1.3508	0.687	0.105	1.794e-57	4.143e-53
13	Lymphatic endothelial cells	*ADGRG3*	1.3507	0.537	0.005	0	0
13	Lymphatic endothelial cells	*NFAT5*	1.3499	0.761	0.483	5.624e-13	1.299e-08
13	Lymphatic endothelial cells	*MINDY2*	1.3478	0.821	0.363	1.651e-24	3.813e-20
13	Lymphatic endothelial cells	*LOC138843527*	1.3475	0.985	0.660	1.989e-26	4.593e-22
13	Lymphatic endothelial cells	*ANKH*	1.3323	0.642	0.105	7.632e-48	1.762e-43
13	Lymphatic endothelial cells	*GPIHBP1*	1.3317	0.522	0.031	2.941e-104	6.791e-100
13	Lymphatic endothelial cells	*GPX1*	1.3256	0.881	0.510	1.274e-19	2.941e-15
13	Lymphatic endothelial cells	*SNTB2*	1.3224	0.657	0.191	3.556e-27	8.212e-23
13	Lymphatic endothelial cells	*EHD1*	1.3169	0.642	0.132	1.606e-36	3.708e-32
13	Lymphatic endothelial cells	*SKAP2*	1.3156	0.582	0.042	3.529e-100	8.148e-96
13	Lymphatic endothelial cells	*ADRB2*	1.3154	0.522	0.012	1.352e-241	3.121e-237
13	Lymphatic endothelial cells	*RASGRP3*	1.3149	0.522	0.039	2.092e-84	4.832e-80
13	Lymphatic endothelial cells	*PDE4B*	1.3128	0.612	0.033	1.969e-137	4.546e-133
13	Lymphatic endothelial cells	*SRRM2*	1.3107	0.955	0.736	1.063e-24	2.455e-20
13	Lymphatic endothelial cells	*ZFPM2*	1.3057	0.746	0.197	7.242e-30	1.672e-25
13	Lymphatic endothelial cells	*YWHAB*	1.3022	0.836	0.408	6.845e-22	1.581e-17
13	Lymphatic endothelial cells	*ADAM10*	1.2975	0.791	0.294	3.732e-25	8.617e-21
13	Lymphatic endothelial cells	*YES1*	1.2946	0.701	0.229	2.195e-24	5.069e-20

Original cluster	Annotated cell type	Gene	avg_log2FC	pct.1	pct.2	Raw P value	Bonferroni-adjusted P value
13	Lymphatic endothelial cells	*TC2N*	1.2875	0.627	0.112	9.762e-43	2.254e-38
13	Lymphatic endothelial cells	*TUBB6*	1.2852	0.642	0.128	1.100e-37	2.540e-33
13	Lymphatic endothelial cells	*GMFG*	1.2849	0.567	0.042	5.037e-93	1.163e-88
13	Lymphatic endothelial cells	*VAT1*	1.2794	0.716	0.214	1.218e-28	2.812e-24
13	Lymphatic endothelial cells	*FAM124B*	1.2753	0.597	0.014	3.705e-284	8.556e-280
13	Lymphatic endothelial cells	*ELK4*	1.2744	0.672	0.174	2.759e-30	6.371e-26
13	Lymphatic endothelial cells	*SHANK3*	1.2644	0.537	0.033	5.383e-106	1.243e-101
13	Lymphatic endothelial cells	*PNPLA2*	1.2559	0.552	0.077	7.750e-47	1.790e-42
13	Lymphatic endothelial cells	*ROBO4*	1.2532	0.552	0.024	4.990e-154	1.152e-149
13	Lymphatic endothelial cells	*COL4A2*	1.2524	0.627	0.100	1.651e-44	3.812e-40
13	Lymphatic endothelial cells	*RALB*	1.2365	0.657	0.121	4.042e-42	9.334e-38
13	Lymphatic endothelial cells	*DPP4*	1.2352	0.239	0.017	3.086e-42	7.126e-38
13	Lymphatic endothelial cells	*HIF1A*	1.2290	0.672	0.191	3.013e-26	6.958e-22
13	Lymphatic endothelial cells	*ELN*	1.2289	0.522	0.062	5.860e-54	1.353e-49
13	Lymphatic endothelial cells	*TTC9*	1.2279	0.433	0.010	5.463e-208	1.261e-203
13	Lymphatic endothelial cells	*ELMO1*	1.2162	0.642	0.112	2.215e-42	5.114e-38
13	Lymphatic endothelial cells	*ITGB1*	1.2161	0.791	0.456	1.413e-15	3.262e-11
13	Lymphatic endothelial cells	*TSPAN14*	1.2108	0.567	0.046	3.881e-86	8.963e-82
13	Lymphatic endothelial cells	*LOC138850522*	1.2010	0.388	0.009	5.018e-177	1.159e-172
13	Lymphatic endothelial cells	*FLRT2*	1.2002	0.672	0.184	6.445e-28	1.488e-23
13	Lymphatic endothelial cells	*PLEKHO2*	1.1955	0.463	0.047	2.402e-55	5.547e-51
13	Lymphatic endothelial cells	*CMTM7*	1.1926	0.746	0.206	3.343e-29	7.721e-25
13	Lymphatic endothelial cells	*LOC103350037*	1.1919	0.493	0.009	5.332e-268	1.231e-263
13	Lymphatic endothelial cells	*CTHRC1*	1.1910	0.418	0.014	6.640e-148	1.533e-143
13	Lymphatic endothelial cells	*LRP5*	1.1904	0.522	0.068	6.074e-49	1.403e-44
13	Lymphatic endothelial cells	*B4GALNT1*	1.1897	0.567	0.023	3.317e-169	7.660e-165
13	Lymphatic endothelial cells	*STOX2*	1.1884	0.612	0.127	6.910e-32	1.596e-27
13	Lymphatic endothelial cells	*NFIB*	1.1832	0.776	0.364	1.388e-15	3.205e-11
13	Lymphatic endothelial cells	*LOC100351475*	1.1822	0.537	0.012	4.192e-256	9.680e-252
13	Lymphatic endothelial cells	*TMBIM1*	1.1821	0.537	0.114	4.861e-28	1.122e-23
13	Lymphatic endothelial cells	*COL18A1*	1.1808	0.463	0.071	1.888e-35	4.361e-31
13	Lymphatic endothelial cells	*DTX1*	1.1803	0.463	0.001	0	0
13	Lymphatic endothelial cells	*MGAT4B*	1.1722	0.776	0.233	1.613e-29	3.725e-25
13	Lymphatic endothelial cells	*AHNAK*	1.1645	0.955	0.629	4.513e-21	1.042e-16
13	Lymphatic endothelial cells	*PALM*	1.1624	0.522	0.046	1.570e-72	3.625e-68
13	Lymphatic endothelial cells	*TSPAN4*	1.1583	0.627	0.172	6.049e-25	1.397e-20
13	Lymphatic endothelial cells	*APP*	1.1569	0.970	0.649	1.524e-20	3.519e-16
13	Lymphatic endothelial cells	*PKIG*	1.1532	0.552	0.120	9.958e-28	2.299e-23
13	Lymphatic endothelial cells	*PTPRB*	1.1493	0.597	0.039	3.181e-108	7.347e-104
13	Lymphatic endothelial cells	*TIMP2*	1.1410	0.925	0.600	6.587e-19	1.521e-14

Original cluster	Annotated cell type	Gene	avg_log2FC	pct.1	pct.2	Raw P value	Bonferroni-adjusted P value
13	Lymphatic endothelial cells	*MOB2*	1.1354	0.642	0.212	3.627e-19	8.376e-15
13	Lymphatic endothelial cells	*MARCKS*	1.1350	0.985	0.760	1.712e-22	3.953e-18
13	Lymphatic endothelial cells	*CDH13*	1.1312	0.478	0.053	1.131e-51	2.611e-47
13	Lymphatic endothelial cells	*FRMD3*	1.1310	0.463	0.049	1.338e-52	3.090e-48
13	Lymphatic endothelial cells	*NICOL1*	1.1257	0.866	0.541	3.000e-15	6.927e-11
13	Lymphatic endothelial cells	*TLL1*	1.1251	0.403	0.067	1.171e-27	2.704e-23
13	Lymphatic endothelial cells	*PTMS*	1.1189	0.896	0.613	2.625e-17	6.061e-13
13	Lymphatic endothelial cells	*SLC29A1*	1.1181	0.687	0.197	1.346e-24	3.108e-20
13	Lymphatic endothelial cells	*AKT1*	1.1164	0.687	0.164	4.080e-31	9.422e-27
13	Lymphatic endothelial cells	*LRRC1*	1.1147	0.433	0.036	4.485e-63	1.036e-58
13	Lymphatic endothelial cells	*UNC5B*	1.1134	0.478	0.041	2.333e-68	5.387e-64
13	Lymphatic endothelial cells	*PGM5*	1.1132	0.522	0.058	2.556e-55	5.903e-51
13	Lymphatic endothelial cells	*CD151*	1.1131	0.597	0.154	2.946e-23	6.802e-19
13	Lymphatic endothelial cells	*HES1*	1.1081	0.657	0.179	1.213e-22	2.802e-18
13	Lymphatic endothelial cells	*REEP3*	1.1067	0.701	0.271	1.126e-18	2.600e-14
13	Lymphatic endothelial cells	*LAMA4*	1.1053	0.687	0.204	1.867e-25	4.312e-21
13	Lymphatic endothelial cells	*PLEKHG1*	1.1053	0.537	0.081	1.966e-40	4.540e-36
13	Lymphatic endothelial cells	*DNASE1L1*	1.1033	0.463	0.027	1.251e-95	2.889e-91
13	Lymphatic endothelial cells	*SCN3B*	1.1022	0.418	0.012	1.631e-163	3.766e-159
13	Lymphatic endothelial cells	*MAN1C1*	1.1022	0.582	0.101	2.232e-38	5.155e-34
13	Lymphatic endothelial cells	*NUPR1*	1.1004	0.851	0.383	5.469e-19	1.263e-14
13	Lymphatic endothelial cells	*EHD2*	1.0979	0.657	0.136	3.764e-35	8.691e-31
13	Lymphatic endothelial cells	*LYL1*	1.0918	0.493	0.042	2.162e-70	4.992e-66
13	Lymphatic endothelial cells	*SASH1*	1.0894	0.701	0.285	3.063e-17	7.073e-13
13	Lymphatic endothelial cells	*GNAI2*	1.0821	0.881	0.540	4.954e-17	1.144e-12
13	Lymphatic endothelial cells	*PDCD4*	1.0815	0.672	0.328	9.679e-13	2.235e-08
13	Lymphatic endothelial cells	*HSPG2*	1.0790	0.627	0.123	8.319e-36	1.921e-31
13	Lymphatic endothelial cells	*LY6H*	1.0787	0.418	0.030	3.724e-70	8.599e-66
13	Lymphatic endothelial cells	*ARRB1*	1.0783	0.433	0.065	5.260e-34	1.215e-29
13	Lymphatic endothelial cells	*LOC103347814*	1.0777	0.448	0.013	2.882e-173	6.656e-169
13	Lymphatic endothelial cells	*VAMP8*	1.0761	0.627	0.157	1.494e-25	3.449e-21
13	Lymphatic endothelial cells	*F11R*	1.0745	0.403	0.021	2.631e-94	6.075e-90
13	Lymphatic endothelial cells	*TRIOBP*	1.0722	0.612	0.117	7.195e-36	1.661e-31
13	Lymphatic endothelial cells	*ARID5B*	1.0694	0.836	0.475	1.156e-14	2.670e-10
13	Lymphatic endothelial cells	*ARHGEF12*	1.0672	0.701	0.302	6.221e-16	1.437e-11
13	Lymphatic endothelial cells	*ENPP3*	1.0647	0.254	0.027	1.841e-29	4.252e-25
13	Lymphatic endothelial cells	*MAP3K1*	1.0629	0.612	0.157	6.199e-25	1.431e-20
13	Lymphatic endothelial cells	*LAYN*	1.0621	0.463	0.073	2.146e-34	4.957e-30
13	Lymphatic endothelial cells	*OAF*	1.0604	0.582	0.145	1.536e-23	3.548e-19
13	Lymphatic endothelial cells	*UTRN*	1.0583	0.866	0.548	2.507e-14	5.789e-10

Original cluster	Annotated cell type	Gene	avg_log2FC	pct.1	pct.2	Raw P value	Bonferroni-adjusted P value
13	Lymphatic endothelial cells	*NID2*	1.0578	0.552	0.081	8.160e-43	1.884e-38
13	Lymphatic endothelial cells	*ARHGDIA*	1.0570	0.776	0.371	1.886e-15	4.355e-11
13	Lymphatic endothelial cells	*PRKCH*	1.0567	0.552	0.064	3.368e-55	7.777e-51
13	Lymphatic endothelial cells	*RAB11FIP1*	1.0492	0.552	0.146	3.340e-22	7.713e-18
13	Lymphatic endothelial cells	*CAVIN3*	1.0438	0.896	0.553	2.151e-12	4.967e-08
13	Lymphatic endothelial cells	*DYNC1H1*	1.0396	0.582	0.171	4.333e-19	1.001e-14
13	Lymphatic endothelial cells	*HOXB5*	1.0357	0.403	0.010	1.610e-178	3.717e-174
13	Lymphatic endothelial cells	*OSMR*	1.0340	0.507	0.097	1.327e-28	3.064e-24
13	Lymphatic endothelial cells	*HMCN1*	1.0332	0.522	0.104	3.286e-28	7.588e-24
13	Lymphatic endothelial cells	*IGF1*	1.0326	0.806	0.476	6.071e-15	1.402e-10
13	Lymphatic endothelial cells	*GARRE1*	1.0326	0.701	0.177	4.942e-29	1.141e-24
13	Lymphatic endothelial cells	*NECTIN2*	1.0320	0.448	0.073	1.318e-31	3.044e-27
13	Lymphatic endothelial cells	*MYH9*	1.0317	0.672	0.216	6.944e-21	1.603e-16
13	Lymphatic endothelial cells	*OST4*	1.0296	0.910	0.626	2.140e-16	4.941e-12
13	Lymphatic endothelial cells	*SREBF2*	1.0285	0.478	0.064	1.099e-41	2.538e-37
13	Lymphatic endothelial cells	*ENDOD1*	1.0263	0.507	0.088	3.606e-33	8.326e-29
13	Lymphatic endothelial cells	*TIE1*	1.0252	0.478	0.049	3.100e-56	7.159e-52
13	Lymphatic endothelial cells	*MRC1*	1.0243	0.403	0.005	5.162e-279	1.192e-274
13	Lymphatic endothelial cells	*TNS1*	1.0199	0.701	0.358	2.437e-13	5.627e-09
13	Lymphatic endothelial cells	*ABHD17C*	1.0169	0.463	0.047	3.528e-55	8.146e-51
13	Lymphatic endothelial cells	*PIEZO2*	1.0168	0.433	0.034	2.627e-67	6.066e-63
13	Lymphatic endothelial cells	*KBTBD11*	1.0167	0.493	0.004	0	0
13	Lymphatic endothelial cells	*ADGRL4*	1.0127	0.567	0.038	3.742e-97	8.640e-93
13	Lymphatic endothelial cells	*ARAP3*	1.0118	0.478	0.030	4.867e-93	1.124e-88
13	Lymphatic endothelial cells	*FSCN1*	1.0115	0.567	0.166	1.496e-18	3.455e-14
13	Lymphatic endothelial cells	*RALBP1*	1.0115	0.672	0.269	7.975e-15	1.842e-10
13	Lymphatic endothelial cells	*DEPP1*	1.0115	0.313	0.069	3.618e-15	8.355e-11
13	Lymphatic endothelial cells	*SIPA1*	1.0105	0.388	0.038	1.200e-48	2.771e-44
13	Lymphatic endothelial cells	*LOC127493681*	1.0094	0.493	0.023	5.465e-124	1.262e-119
13	Lymphatic endothelial cells	*RAC1*	1.0085	0.776	0.494	1.830e-13	4.225e-09
13	Lymphatic endothelial cells	*ADGRF5*	1.0080	0.552	0.063	4.442e-54	1.026e-49
13	Lymphatic endothelial cells	*PEAR1*	1.0075	0.418	0.043	1.331e-48	3.073e-44
13	Lymphatic endothelial cells	*MAN1A1*	1.0056	0.642	0.158	3.973e-27	9.174e-23
13	Lymphatic endothelial cells	*PPP1CA*	0.9988	0.657	0.234	1.011e-17	2.334e-13
13	Lymphatic endothelial cells	*REEP5*	0.9975	0.716	0.303	4.550e-16	1.051e-11
13	Lymphatic endothelial cells	*UBALD2*	0.9939	0.657	0.156	1.333e-28	3.077e-24
13	Lymphatic endothelial cells	*MLF2*	0.9913	0.522	0.133	1.443e-21	3.332e-17
13	Lymphatic endothelial cells	*TBC1D4*	0.9899	0.567	0.132	3.046e-25	7.033e-21
13	Lymphatic endothelial cells	*VWF*	0.9892	0.269	0.047	1.908e-17	4.405e-13
13	Lymphatic endothelial cells	*LY96*	0.9861	0.463	0.043	6.490e-61	1.499e-56

Original cluster	Annotated cell type	Gene	avg_log2FC	pct.1	pct.2	Raw P value	Bonferroni-adjusted P value
13	Lymphatic endothelial cells	*VAV3*	0.9859	0.567	0.117	6.909e-29	1.595e-24
13	Lymphatic endothelial cells	*CAST*	0.9840	0.687	0.262	1.181e-17	2.727e-13
13	Lymphatic endothelial cells	*FOXP1*	0.9837	0.866	0.550	3.093e-14	7.143e-10
13	Lymphatic endothelial cells	*EPHB4*	0.9809	0.388	0.051	1.145e-34	2.643e-30
13	Lymphatic endothelial cells	*PPIA*	0.9793	0.851	0.477	1.148e-15	2.651e-11
13	Lymphatic endothelial cells	*HYAL2*	0.9769	0.478	0.084	1.325e-30	3.060e-26
13	Lymphatic endothelial cells	*PLEC*	0.9761	0.507	0.130	4.380e-21	1.011e-16
13	Lymphatic endothelial cells	*RNF150*	0.9733	0.463	0.094	3.791e-24	8.754e-20
13	Lymphatic endothelial cells	*GAS6*	0.9730	0.701	0.212	9.734e-23	2.248e-18
13	Lymphatic endothelial cells	*LIMS1*	0.9720	0.746	0.302	3.077e-17	7.104e-13
13	Lymphatic endothelial cells	*SRGAP2*	0.9714	0.687	0.303	1.293e-14	2.986e-10
13	Lymphatic endothelial cells	*SLC8A3*	0.9713	0.343	0.001	0	0
13	Lymphatic endothelial cells	*TJP1*	0.9712	0.701	0.227	6.448e-22	1.489e-17
13	Lymphatic endothelial cells	*TLE1*	0.9665	0.657	0.229	1.186e-17	2.738e-13
13	Lymphatic endothelial cells	*PON2*	0.9657	0.597	0.159	3.099e-24	7.157e-20
13	Lymphatic endothelial cells	*XBP1*	0.9657	0.642	0.270	4.312e-14	9.958e-10
13	Lymphatic endothelial cells	*S100A1*	0.9652	0.403	0.032	2.117e-62	4.888e-58
13	Lymphatic endothelial cells	*LTC4S*	0.9649	0.373	0.023	3.314e-74	7.653e-70
13	Lymphatic endothelial cells	*EIF3A*	0.9646	0.716	0.313	2.297e-14	5.305e-10
13	Lymphatic endothelial cells	*CHST15*	0.9640	0.448	0.022	7.426e-109	1.715e-104
13	Lymphatic endothelial cells	*C13H1orf21*	0.9600	0.478	0.087	3.427e-28	7.915e-24
13	Lymphatic endothelial cells	*TMSB15A*	0.9568	0.821	0.489	1.101e-13	2.542e-09
13	Lymphatic endothelial cells	*LOC100351485*	0.9564	0.493	0.068	9.265e-40	2.140e-35
13	Lymphatic endothelial cells	*BBLN*	0.9510	0.493	0.100	4.933e-26	1.139e-21
13	Lymphatic endothelial cells	*C1QTNF5*	0.9496	0.493	0.100	4.290e-26	9.906e-22
13	Lymphatic endothelial cells	*SYNM*	0.9495	0.448	0.047	8.110e-50	1.873e-45
13	Lymphatic endothelial cells	*HMG20B*	0.9461	0.507	0.125	5.282e-21	1.220e-16
13	Lymphatic endothelial cells	*DOCK4*	0.9442	0.463	0.102	5.621e-22	1.298e-17
13	Lymphatic endothelial cells	*SHC1*	0.9436	0.507	0.104	5.622e-27	1.298e-22
13	Lymphatic endothelial cells	*TES*	0.9433	0.463	0.089	7.289e-26	1.683e-21
13	Lymphatic endothelial cells	*PHLDB3*	0.9433	0.373	0.013	3.386e-121	7.818e-117
13	Lymphatic endothelial cells	*TMEM44*	0.9427	0.358	0.012	2.039e-126	4.708e-122
13	Lymphatic endothelial cells	*HMOX2*	0.9418	0.463	0.093	8.067e-26	1.863e-21
13	Lymphatic endothelial cells	*PALM2AKAP2*	0.9414	0.612	0.135	1.485e-27	3.430e-23
13	Lymphatic endothelial cells	*COL4A1*	0.9396	0.776	0.203	2.571e-28	5.937e-24
13	Lymphatic endothelial cells	*KIAA1671*	0.9396	0.403	0.035	8.479e-58	1.958e-53
13	Lymphatic endothelial cells	*PFN1*	0.9386	0.896	0.559	4.502e-19	1.040e-14
13	Lymphatic endothelial cells	*S100A11*	0.9381	0.970	0.751	7.886e-20	1.821e-15
13	Lymphatic endothelial cells	*CUEDC1*	0.9360	0.403	0.047	7.605e-41	1.756e-36
13	Lymphatic endothelial cells	*TACC1*	0.9333	0.597	0.229	1.660e-13	3.832e-09

Original cluster	Annotated cell type	Gene	avg_log2FC	pct.1	pct.2	Raw P value	Bonferroni-adjusted P value
13	Lymphatic endothelial cells	*EDNRB*	0.9326	0.373	0.038	4.507e-44	1.041e-39
13	Lymphatic endothelial cells	*MKNK2*	0.9302	0.597	0.186	1.489e-17	3.438e-13
13	Lymphatic endothelial cells	*SBSPON*	0.9288	0.328	0.018	1.038e-73	2.398e-69
13	Lymphatic endothelial cells	*RHOA*	0.9282	0.881	0.612	4.088e-14	9.439e-10
13	Lymphatic endothelial cells	*EPB41*	0.9262	0.597	0.259	5.127e-12	1.184e-07
13	Lymphatic endothelial cells	*TAX1BP3*	0.9242	0.463	0.074	8.537e-33	1.971e-28
13	Lymphatic endothelial cells	*C13H1orf115*	0.9187	0.478	0.035	1.892e-76	4.368e-72
13	Lymphatic endothelial cells	*MVB12A*	0.9179	0.597	0.190	4.748e-17	1.096e-12
13	Lymphatic endothelial cells	*REEP1*	0.9168	0.373	0.012	1.357e-135	3.133e-131
13	Lymphatic endothelial cells	*LRRC32*	0.9111	0.478	0.062	3.268e-42	7.548e-38
13	Lymphatic endothelial cells	*LOC127483734*	0.9107	0.418	0.021	4.463e-99	1.031e-94
13	Lymphatic endothelial cells	*TBC1D9*	0.9105	0.493	0.102	4.891e-25	1.130e-20
13	Lymphatic endothelial cells	*CD93*	0.9104	0.254	0.053	7.397e-13	1.708e-08
13	Lymphatic endothelial cells	*FHL1*	0.9101	0.597	0.147	4.113e-24	9.498e-20
13	Lymphatic endothelial cells	*MTUS1*	0.9070	0.522	0.146	1.037e-18	2.394e-14
13	Lymphatic endothelial cells	*ITSN1*	0.9067	0.612	0.180	8.971e-20	2.072e-15
13	Lymphatic endothelial cells	*TSC22D4*	0.9067	0.507	0.106	9.101e-26	2.102e-21
13	Lymphatic endothelial cells	*C6H5orf24*	0.9064	0.791	0.494	5.171e-11	1.194e-06
13	Lymphatic endothelial cells	*KALRN*	0.9046	0.507	0.149	1.031e-16	2.381e-12
13	Lymphatic endothelial cells	*SPRY1*	0.9029	0.403	0.080	2.850e-21	6.581e-17
13	Lymphatic endothelial cells	*FGD6*	0.9012	0.463	0.057	3.047e-43	7.037e-39
13	Lymphatic endothelial cells	*MAPK3*	0.8981	0.507	0.128	1.492e-20	3.446e-16
13	Lymphatic endothelial cells	*MARCHF3*	0.8976	0.463	0.074	3.043e-33	7.026e-29
13	Lymphatic endothelial cells	*FLNC*	0.8971	0.388	0.052	2.266e-33	5.232e-29
13	Lymphatic endothelial cells	*NOD1*	0.8969	0.388	0.030	1.272e-60	2.938e-56
13	Lymphatic endothelial cells	*PTPN3*	0.8935	0.358	0.021	1.056e-72	2.439e-68
13	Lymphatic endothelial cells	*SH3GL1*	0.8898	0.597	0.166	7.215e-21	1.666e-16
13	Lymphatic endothelial cells	*CTSB*	0.8858	0.657	0.299	6.502e-11	1.502e-06
13	Lymphatic endothelial cells	*CAV2*	0.8845	0.687	0.234	4.409e-17	1.018e-12
13	Lymphatic endothelial cells	*EXOC6*	0.8837	0.507	0.103	3.839e-27	8.865e-23
13	Lymphatic endothelial cells	*NUDT4*	0.8834	0.672	0.351	8.704e-11	2.010e-06
13	Lymphatic endothelial cells	*MANSC1*	0.8810	0.358	0.026	1.106e-59	2.553e-55
13	Lymphatic endothelial cells	*PAK2*	0.8802	0.731	0.374	7.083e-13	1.636e-08
13	Lymphatic endothelial cells	*CAPN2*	0.8801	0.552	0.169	2.057e-16	4.750e-12
13	Lymphatic endothelial cells	*ARPC1B*	0.8782	0.597	0.126	2.008e-29	4.637e-25
13	Lymphatic endothelial cells	*TMEM204*	0.8769	0.507	0.116	6.728e-23	1.554e-18
13	Lymphatic endothelial cells	*LYN*	0.8760	0.463	0.033	1.546e-78	3.570e-74
13	Lymphatic endothelial cells	*ASAP1*	0.8757	0.672	0.316	1.050e-12	2.425e-08
13	Lymphatic endothelial cells	*DOCK1*	0.8751	0.537	0.144	4.998e-20	1.154e-15
13	Lymphatic endothelial cells	*ITGA6*	0.8747	0.463	0.052	3.238e-48	7.478e-44

Original cluster	Annotated cell type	Gene	avg_log2FC	pct.1	pct.2	Raw P value	Bonferroni-adjusted P value
13	Lymphatic endothelial cells	*MAP4K2*	0.8702	0.388	0.019	8.000e-98	1.847e-93
13	Lymphatic endothelial cells	*TNFAIP8L1*	0.8675	0.358	0.022	1.615e-72	3.729e-68
13	Lymphatic endothelial cells	*HID1*	0.8675	0.343	0.013	2.280e-106	5.266e-102
13	Lymphatic endothelial cells	*GFOD2*	0.8668	0.433	0.061	2.275e-35	5.252e-31
13	Lymphatic endothelial cells	*VPS36*	0.8664	0.448	0.151	1.924e-11	4.443e-07
13	Lymphatic endothelial cells	*SRGAP1*	0.8657	0.418	0.064	4.903e-31	1.132e-26
13	Lymphatic endothelial cells	*LOC138849238*	0.8644	0.388	0.009	9.910e-187	2.288e-182
13	Lymphatic endothelial cells	*LOC100340954*	0.8629	0.821	0.452	1.886e-13	4.356e-09
13	Lymphatic endothelial cells	*PIR*	0.8624	0.358	0.018	5.109e-87	1.180e-82
13	Lymphatic endothelial cells	*CASKIN2*	0.8624	0.403	0.050	1.478e-37	3.413e-33
13	Lymphatic endothelial cells	*CD63*	0.8621	0.985	0.893	6.751e-16	1.559e-11
13	Lymphatic endothelial cells	*ITGA1*	0.8591	0.627	0.166	9.016e-22	2.082e-17
13	Lymphatic endothelial cells	*PGER5*	0.8552	0.731	0.290	6.633e-15	1.532e-10
13	Lymphatic endothelial cells	*MACF1*	0.8534	0.776	0.483	7.031e-11	1.624e-06
13	Lymphatic endothelial cells	*CALCRL*	0.8518	0.642	0.345	6.822e-09	1.575e-04
13	Lymphatic endothelial cells	*PLD3*	0.8502	0.537	0.152	4.325e-18	9.987e-14
13	Lymphatic endothelial cells	*GOLIM4*	0.8493	0.657	0.320	2.122e-10	4.899e-06
13	Lymphatic endothelial cells	*RTN4*	0.8492	0.806	0.537	6.725e-09	1.553e-04
13	Lymphatic endothelial cells	*AAK1*	0.8482	0.537	0.146	4.108e-19	9.486e-15
13	Lymphatic endothelial cells	*LIMD1*	0.8464	0.388	0.078	4.653e-21	1.074e-16
13	Lymphatic endothelial cells	*SERP1*	0.8464	0.776	0.428	1.722e-10	3.977e-06
13	Lymphatic endothelial cells	*MRPL41*	0.8451	0.597	0.191	1.891e-17	4.367e-13
13	Lymphatic endothelial cells	*CSTB*	0.8441	0.836	0.639	3.379e-09	7.803e-05
13	Lymphatic endothelial cells	*TXNRD2*	0.8430	0.343	0.028	5.057e-51	1.168e-46
13	Lymphatic endothelial cells	*CHMP3*	0.8413	0.731	0.319	4.417e-14	1.020e-09
13	Lymphatic endothelial cells	*PEA15*	0.8404	0.493	0.122	2.375e-20	5.485e-16
13	Lymphatic endothelial cells	*CTSZ*	0.8404	0.448	0.115	1.223e-17	2.823e-13
13	Lymphatic endothelial cells	*MAP4K4*	0.8376	0.761	0.401	1.048e-10	2.419e-06
13	Lymphatic endothelial cells	*QKI*	0.8324	0.821	0.417	1.502e-13	3.467e-09
13	Lymphatic endothelial cells	*RAB11A*	0.8314	0.537	0.126	2.723e-23	6.287e-19
13	Lymphatic endothelial cells	*LY6E*	0.8302	0.493	0.236	1.379e-07	3.185e-03
13	Lymphatic endothelial cells	*CERT1*	0.8286	0.567	0.162	1.221e-18	2.819e-14
13	Lymphatic endothelial cells	*DCBLD1*	0.8245	0.373	0.036	7.100e-47	1.639e-42
13	Lymphatic endothelial cells	*TSPO*	0.8242	0.612	0.211	8.331e-16	1.924e-11
13	Lymphatic endothelial cells	*F8*	0.8231	0.388	0.013	2.509e-131	5.793e-127
13	Lymphatic endothelial cells	*TPD52L1*	0.8199	0.672	0.283	3.763e-13	8.689e-09
13	Lymphatic endothelial cells	*P3H2*	0.8198	0.418	0.067	2.379e-28	5.493e-24
13	Lymphatic endothelial cells	*TMEM205*	0.8186	0.612	0.232	6.321e-13	1.460e-08
13	Lymphatic endothelial cells	*CD1D*	0.8183	0.388	0.026	1.998e-69	4.615e-65
13	Lymphatic endothelial cells	*ABCB10*	0.8178	0.343	0.056	1.206e-23	2.784e-19

Original cluster	Annotated cell type	Gene	avg_log2FC	pct.1	pct.2	Raw P value	Bonferroni-adjusted P value
13	Lymphatic endothelial cells	*RNF125*	0.8149	0.522	0.153	4.311e-17	9.955e-13
13	Lymphatic endothelial cells	*ATP6V0A1*	0.8148	0.463	0.105	1.698e-21	3.922e-17
13	Lymphatic endothelial cells	*LTBP3*	0.8147	0.537	0.206	4.565e-11	1.054e-06
13	Lymphatic endothelial cells	*EVA1A*	0.8120	0.403	0.028	6.339e-70	1.464e-65
13	Lymphatic endothelial cells	*PDLIM1*	0.8078	0.418	0.053	3.566e-38	8.236e-34
13	Lymphatic endothelial cells	*PLXND1*	0.8073	0.463	0.098	1.280e-21	2.955e-17
13	Lymphatic endothelial cells	*YBX1*	0.8058	0.896	0.694	4.577e-12	1.057e-07
13	Lymphatic endothelial cells	*TNFRSF11A*	0.8055	0.403	0.028	7.873e-71	1.818e-66
13	Lymphatic endothelial cells	*VAMP3*	0.8032	0.567	0.207	3.847e-13	8.884e-09
13	Lymphatic endothelial cells	*RAB3IL1*	0.8018	0.478	0.096	3.129e-24	7.225e-20
13	Lymphatic endothelial cells	*ADAMTSL3*	0.8013	0.313	0.040	7.927e-28	1.830e-23
13	Lymphatic endothelial cells	*CHMP2A*	0.8008	0.657	0.302	2.564e-11	5.921e-07
13	Lymphatic endothelial cells	*PROKR1*	0.8002	0.313	0.000	0	0
13	Lymphatic endothelial cells	*FLOT1*	0.7987	0.418	0.124	1.260e-13	2.910e-09
13	Lymphatic endothelial cells	*GLUL*	0.7986	0.851	0.417	6.955e-15	1.606e-10
13	Lymphatic endothelial cells	*DLL4*	0.7973	0.418	0.018	2.099e-112	4.848e-108
13	Lymphatic endothelial cells	*CNKSR3*	0.7973	0.433	0.091	1.336e-21	3.084e-17
13	Lymphatic endothelial cells	*OSBPL3*	0.7955	0.418	0.036	5.009e-58	1.157e-53
13	Lymphatic endothelial cells	*PARVA*	0.7950	0.567	0.205	1.494e-12	3.450e-08
13	Lymphatic endothelial cells	*DIPK2B*	0.7950	0.343	0.019	2.961e-75	6.837e-71
13	Lymphatic endothelial cells	*CFL1*	0.7949	0.821	0.513	1.578e-09	3.644e-05
13	Lymphatic endothelial cells	*FKBP1A*	0.7942	0.537	0.216	2.785e-10	6.430e-06
13	Lymphatic endothelial cells	*FTL*	0.7938	1.000	0.988	1.950e-17	4.504e-13
13	Lymphatic endothelial cells	*FRMD6*	0.7931	0.418	0.109	2.394e-15	5.528e-11
13	Lymphatic endothelial cells	*CDK17*	0.7915	0.597	0.168	4.260e-19	9.838e-15
13	Lymphatic endothelial cells	*SH2D3C*	0.7905	0.448	0.090	2.935e-23	6.777e-19
13	Lymphatic endothelial cells	*ECE1*	0.7898	0.507	0.177	2.295e-12	5.299e-08
13	Lymphatic endothelial cells	*PXDC1*	0.7895	0.478	0.084	7.540e-30	1.741e-25
13	Lymphatic endothelial cells	*TTYH3*	0.7891	0.403	0.073	9.360e-25	2.161e-20
13	Lymphatic endothelial cells	*TMEM219*	0.7869	0.522	0.162	4.893e-15	1.130e-10
13	Lymphatic endothelial cells	*NREP*	0.7861	0.313	0.078	5.416e-13	1.251e-08
13	Lymphatic endothelial cells	*CEBPD*	0.7847	0.896	0.711	9.988e-08	2.306e-03
13	Lymphatic endothelial cells	*LOC127490996*	0.7846	0.239	0.058	5.070e-10	1.171e-05
13	Lymphatic endothelial cells	*LOC100358069*	0.7845	0.896	0.662	3.698e-10	8.539e-06
13	Lymphatic endothelial cells	*DNAJB4*	0.7843	0.478	0.162	1.191e-11	2.751e-07
13	Lymphatic endothelial cells	*SEMA3D*	0.7838	0.358	0.062	7.419e-23	1.713e-18
13	Lymphatic endothelial cells	*AFDN*	0.7821	0.343	0.037	4.508e-38	1.041e-33
13	Lymphatic endothelial cells	*COLEC12*	0.7815	0.657	0.291	6.992e-11	1.615e-06
13	Lymphatic endothelial cells	*ZNRF1*	0.7801	0.478	0.153	2.630e-13	6.074e-09
13	Lymphatic endothelial cells	*FGF13*	0.7798	0.328	0.076	1.531e-14	3.535e-10

Original cluster	Annotated cell type	Gene	avg_log2FC	pct.1	pct.2	Raw P value	Bonferroni-adjusted P value
13	Lymphatic endothelial cells	*USHBP1*	0.7793	0.299	0.017	3.364e-63	7.768e-59
13	Lymphatic endothelial cells	*ACTN1*	0.7786	0.612	0.233	6.820e-13	1.575e-08
13	Lymphatic endothelial cells	*FAM43A*	0.7763	0.522	0.211	2.614e-10	6.037e-06
13	Lymphatic endothelial cells	*WASHC4*	0.7751	0.612	0.245	2.653e-12	6.127e-08
13	Lymphatic endothelial cells	*CPNE2*	0.7742	0.373	0.042	7.180e-40	1.658e-35
13	Lymphatic endothelial cells	*UBASH3B*	0.7739	0.343	0.025	1.163e-56	2.685e-52
13	Lymphatic endothelial cells	*SMAD5*	0.7732	0.612	0.240	2.306e-12	5.326e-08
13	Lymphatic endothelial cells	*PALD1*	0.7728	0.343	0.027	2.117e-53	4.888e-49
13	Lymphatic endothelial cells	*RAB5C*	0.7728	0.537	0.179	6.883e-14	1.589e-09
13	Lymphatic endothelial cells	*RABAC1*	0.7717	0.746	0.434	2.606e-10	6.017e-06
13	Lymphatic endothelial cells	*MAP4K5*	0.7692	0.537	0.194	2.119e-12	4.894e-08
13	Lymphatic endothelial cells	*MPRIP*	0.7691	0.507	0.169	6.163e-13	1.423e-08
13	Lymphatic endothelial cells	*CYB5A*	0.7667	0.821	0.460	1.573e-09	3.631e-05
13	Lymphatic endothelial cells	*LOC100328942*	0.7666	0.522	0.213	1.102e-09	2.546e-05
13	Lymphatic endothelial cells	*LOC100354142*	0.7661	0.567	0.208	3.291e-12	7.600e-08
13	Lymphatic endothelial cells	*SPTLC2*	0.7651	0.507	0.089	6.261e-31	1.446e-26
13	Lymphatic endothelial cells	*TGFB1*	0.7643	0.388	0.070	1.481e-23	3.419e-19
13	Lymphatic endothelial cells	*FLI1*	0.7641	0.418	0.051	7.978e-39	1.842e-34
13	Lymphatic endothelial cells	*ATP6V0E1*	0.7614	0.896	0.587	6.671e-10	1.541e-05
13	Lymphatic endothelial cells	*COPS9*	0.7612	0.642	0.300	7.252e-11	1.675e-06
13	Lymphatic endothelial cells	*CAPNS1*	0.7590	0.791	0.435	2.113e-09	4.879e-05
13	Lymphatic endothelial cells	*ST3GAL2*	0.7590	0.433	0.103	4.462e-18	1.030e-13
13	Lymphatic endothelial cells	*TEK*	0.7582	0.388	0.031	1.246e-58	2.878e-54
13	Lymphatic endothelial cells	*ANAPC11*	0.7579	0.642	0.289	2.827e-10	6.527e-06
13	Lymphatic endothelial cells	*USP31*	0.7575	0.418	0.049	3.084e-41	7.120e-37
13	Lymphatic endothelial cells	*ITGA9*	0.7570	0.448	0.084	6.411e-25	1.480e-20
13	Lymphatic endothelial cells	*CHD7*	0.7560	0.373	0.036	7.980e-47	1.843e-42
13	Lymphatic endothelial cells	*CNN3*	0.7555	0.851	0.689	4.747e-10	1.096e-05
13	Lymphatic endothelial cells	*SMIM12*	0.7552	0.418	0.168	1.055e-08	2.436e-04
13	Lymphatic endothelial cells	*SPAAR*	0.7519	0.328	0.027	2.311e-48	5.336e-44
13	Lymphatic endothelial cells	*TMEM109*	0.7512	0.537	0.267	1.702e-07	3.929e-03
13	Lymphatic endothelial cells	*LOC138843080*	0.7509	0.836	0.569	2.010e-06	4.641e-02
13	Lymphatic endothelial cells	*PPP1R18*	0.7496	0.358	0.061	1.373e-23	3.171e-19
13	Lymphatic endothelial cells	*APC*	0.7494	0.657	0.354	1.109e-09	2.561e-05
13	Lymphatic endothelial cells	*CPM*	0.7481	0.328	0.013	4.487e-100	1.036e-95
13	Lymphatic endothelial cells	*ELOB*	0.7478	0.806	0.471	2.605e-11	6.015e-07
13	Lymphatic endothelial cells	*CXCL8*	0.7476	0.403	0.014	8.706e-137	2.010e-132
13	Lymphatic endothelial cells	*TLN1*	0.7464	0.612	0.334	1.015e-08	2.345e-04
13	Lymphatic endothelial cells	*THSD7A*	0.7461	0.299	0.056	2.639e-17	6.093e-13
13	Lymphatic endothelial cells	*TRIM24*	0.7448	0.403	0.113	6.962e-14	1.608e-09

Original cluster	Annotated cell type	Gene	avg_log2FC	pct.1	pct.2	Raw P value	Bonferroni-adjusted P value
13	Lymphatic endothelial cells	*STMN1*	0.7442	0.896	0.604	4.125e-08	9.526e-04
13	Lymphatic endothelial cells	*ATP6V0C*	0.7422	0.821	0.498	8.426e-10	1.946e-05
13	Lymphatic endothelial cells	*TMBIM6*	0.7415	0.881	0.622	5.390e-09	1.245e-04
13	Lymphatic endothelial cells	*SOX4*	0.7413	0.761	0.464	4.603e-08	1.063e-03
13	Lymphatic endothelial cells	*HHEX*	0.7404	0.328	0.020	6.235e-65	1.440e-60
13	Lymphatic endothelial cells	*SNED1*	0.7382	0.597	0.245	3.207e-11	7.405e-07
13	Lymphatic endothelial cells	*MAFF*	0.7378	0.478	0.084	6.052e-29	1.398e-24
13	Lymphatic endothelial cells	*BST2*	0.7364	0.731	0.310	1.432e-14	3.306e-10
13	Lymphatic endothelial cells	*SCAMP2*	0.7359	0.478	0.133	7.136e-16	1.648e-11
13	Lymphatic endothelial cells	*NXN*	0.7339	0.522	0.143	1.333e-17	3.079e-13
13	Lymphatic endothelial cells	*TAL1*	0.7338	0.313	0.006	1.007e-175	2.325e-171
13	Lymphatic endothelial cells	*PXN*	0.7336	0.418	0.105	3.956e-16	9.136e-12
13	Lymphatic endothelial cells	*CTNNA1*	0.7332	0.731	0.347	4.906e-12	1.133e-07
13	Lymphatic endothelial cells	*SHE*	0.7319	0.358	0.020	2.752e-78	6.354e-74
13	Lymphatic endothelial cells	*SFT2D2*	0.7309	0.433	0.101	5.839e-19	1.348e-14
13	Lymphatic endothelial cells	*GNG5*	0.7297	0.746	0.483	2.345e-08	5.414e-04
13	Lymphatic endothelial cells	*ADAM19*	0.7292	0.433	0.038	6.445e-58	1.488e-53
13	Lymphatic endothelial cells	*BMP2K*	0.7288	0.552	0.189	1.534e-13	3.542e-09
13	Lymphatic endothelial cells	*TNFAIP8L3*	0.7287	0.313	0.015	1.610e-76	3.717e-72
13	Lymphatic endothelial cells	*PLCG1*	0.7261	0.358	0.076	1.002e-17	2.313e-13
13	Lymphatic endothelial cells	*AP2S1*	0.7260	0.627	0.233	1.421e-13	3.281e-09
13	Lymphatic endothelial cells	*XKR6*	0.7249	0.343	0.051	6.541e-26	1.510e-21
13	Lymphatic endothelial cells	*RADIL*	0.7242	0.373	0.058	1.149e-26	2.654e-22
13	Lymphatic endothelial cells	*RHPN1*	0.7232	0.284	0.017	1.047e-56	2.418e-52
13	Lymphatic endothelial cells	*RLA-A3*	0.7208	0.716	0.453	1.813e-06	4.185e-02
13	Lymphatic endothelial cells	*CTTNBP2NL*	0.7207	0.463	0.059	4.412e-42	1.019e-37
13	Lymphatic endothelial cells	*STIM2*	0.7172	0.433	0.145	9.999e-11	2.309e-06
13	Lymphatic endothelial cells	*AURKAIP1*	0.7172	0.761	0.438	8.248e-09	1.905e-04
13	Lymphatic endothelial cells	*AP2M1*	0.7154	0.687	0.348	6.375e-10	1.472e-05
13	Lymphatic endothelial cells	*SEMA3A*	0.7149	0.299	0.070	1.254e-12	2.895e-08
13	Lymphatic endothelial cells	*PTGFRN*	0.7134	0.448	0.124	9.647e-15	2.228e-10
13	Lymphatic endothelial cells	*LOC103345796*	0.7132	0.224	0.005	2.765e-110	6.385e-106
13	Lymphatic endothelial cells	*PRKCSH*	0.7127	0.343	0.128	1.450e-07	3.347e-03
13	Lymphatic endothelial cells	*PGK1*	0.7125	0.567	0.247	3.319e-09	7.665e-05
13	Lymphatic endothelial cells	*DNASE2*	0.7099	0.418	0.090	6.881e-20	1.589e-15
13	Lymphatic endothelial cells	*RIPOR1*	0.7090	0.284	0.057	1.258e-15	2.904e-11
13	Lymphatic endothelial cells	*DYNLL1*	0.7076	0.776	0.520	7.466e-07	1.724e-02
13	Lymphatic endothelial cells	*ITGB1BP1*	0.7064	0.328	0.108	6.794e-09	1.569e-04
13	Lymphatic endothelial cells	*MYL6*	0.7041	0.910	0.732	1.899e-10	4.385e-06
13	Lymphatic endothelial cells	*DEGS1*	0.7040	0.493	0.175	1.371e-11	3.165e-07

Original cluster	Annotated cell type	Gene	avg_log2FC	pct.1	pct.2	Raw P value	Bonferroni-adjusted P value
13	Lymphatic endothelial cells	*WBP2*	0.7035	0.567	0.245	2.897e-09	6.689e-05
13	Lymphatic endothelial cells	*LYPD6*	0.7034	0.478	0.128	8.368e-16	1.932e-11
13	Lymphatic endothelial cells	*SOCS2*	0.7030	0.507	0.173	4.951e-12	1.143e-07
13	Lymphatic endothelial cells	*SYNE2*	0.7020	0.522	0.215	2.427e-09	5.604e-05
13	Lymphatic endothelial cells	*SELENOP*	0.7008	0.970	0.804	5.019e-12	1.159e-07
13	Lymphatic endothelial cells	*NAGA*	0.7000	0.313	0.049	9.257e-23	2.138e-18
13	Lymphatic endothelial cells	*SPOCK2*	0.6992	0.343	0.015	2.536e-92	5.857e-88
13	Lymphatic endothelial cells	*PHACTR2*	0.6981	0.642	0.269	1.463e-10	3.379e-06
13	Lymphatic endothelial cells	*BOK*	0.6971	0.299	0.026	1.889e-41	4.361e-37
13	Lymphatic endothelial cells	*LOC103350974*	0.6954	0.209	0.004	1.196e-110	2.761e-106
13	Lymphatic endothelial cells	*THBD*	0.6944	0.388	0.053	2.401e-31	5.545e-27
13	Lymphatic endothelial cells	*ARPC4*	0.6935	0.448	0.165	3.056e-10	7.058e-06
13	Lymphatic endothelial cells	*PPP1R14B*	0.6926	0.552	0.247	1.627e-09	3.757e-05
13	Lymphatic endothelial cells	*CORO1B*	0.6911	0.537	0.162	3.334e-16	7.698e-12
13	Lymphatic endothelial cells	*SMAGP*	0.6902	0.299	0.012	2.041e-87	4.714e-83
13	Lymphatic endothelial cells	*SMDT1*	0.6901	0.522	0.187	1.161e-11	2.682e-07
13	Lymphatic endothelial cells	*HNRNPUL2*	0.6900	0.507	0.242	1.201e-07	2.774e-03
13	Lymphatic endothelial cells	*HNMT*	0.6874	0.343	0.042	9.858e-32	2.276e-27
13	Lymphatic endothelial cells	*YWHAG*	0.6874	0.522	0.215	7.286e-10	1.682e-05
13	Lymphatic endothelial cells	*TSPAN9*	0.6842	0.358	0.055	7.734e-26	1.786e-21
13	Lymphatic endothelial cells	*RNH1*	0.6824	0.463	0.151	4.769e-12	1.101e-07
13	Lymphatic endothelial cells	*EIF4EBP2*	0.6823	0.806	0.546	6.843e-09	1.580e-04
13	Lymphatic endothelial cells	*MTMR12*	0.6789	0.343	0.104	4.914e-10	1.135e-05
13	Lymphatic endothelial cells	*SNAP23*	0.6787	0.478	0.121	7.539e-18	1.741e-13
13	Lymphatic endothelial cells	*APRT*	0.6780	0.448	0.189	6.205e-08	1.433e-03
13	Lymphatic endothelial cells	*C9H12orf57*	0.6780	0.731	0.351	1.989e-08	4.593e-04
13	Lymphatic endothelial cells	*SMYD2*	0.6777	0.373	0.074	4.373e-20	1.010e-15
13	Lymphatic endothelial cells	*BPNT2*	0.6769	0.507	0.192	1.352e-10	3.123e-06
13	Lymphatic endothelial cells	*NR1H3*	0.6768	0.328	0.043	9.568e-29	2.210e-24
13	Lymphatic endothelial cells	*WASF3*	0.6752	0.388	0.168	5.025e-07	1.160e-02
13	Lymphatic endothelial cells	*CORO1C*	0.6746	0.403	0.078	3.355e-22	7.748e-18
13	Lymphatic endothelial cells	*RASSF2*	0.6743	0.284	0.036	2.834e-26	6.544e-22
13	Lymphatic endothelial cells	*GCHFR*	0.6731	0.284	0.021	2.767e-47	6.390e-43
13	Lymphatic endothelial cells	*GPR146*	0.6721	0.299	0.052	6.523e-19	1.506e-14
13	Lymphatic endothelial cells	*ETHE1*	0.6711	0.463	0.093	2.816e-23	6.502e-19
13	Lymphatic endothelial cells	*BNIP2*	0.6707	0.507	0.158	5.923e-14	1.368e-09
13	Lymphatic endothelial cells	*MYCT1*	0.6694	0.328	0.021	9.611e-63	2.219e-58
13	Lymphatic endothelial cells	*DMTN*	0.6688	0.194	0.006	1.455e-71	3.361e-67
13	Lymphatic endothelial cells	*DENND4A*	0.6671	0.507	0.255	7.201e-07	1.663e-02
13	Lymphatic endothelial cells	*RCSD1*	0.6664	0.478	0.096	1.283e-23	2.963e-19

Original cluster	Annotated cell type	Gene	avg_log2FC	pct.1	pct.2	Raw P value	Bonferroni-adjusted P value
13	Lymphatic endothelial cells	*NHSL3*	0.6657	0.254	0.028	6.660e-28	1.538e-23
13	Lymphatic endothelial cells	*LOC100345591*	0.6645	0.642	0.297	2.284e-10	5.275e-06
13	Lymphatic endothelial cells	*FAM171A1*	0.6641	0.433	0.118	5.686e-15	1.313e-10
13	Lymphatic endothelial cells	*PTPN12*	0.6628	0.493	0.228	1.699e-07	3.924e-03
13	Lymphatic endothelial cells	*ZFAND3*	0.6624	0.597	0.298	1.046e-07	2.415e-03
13	Lymphatic endothelial cells	*TXNIP*	0.6623	0.985	0.841	1.074e-09	2.479e-05
13	Lymphatic endothelial cells	*COPZ2*	0.6610	0.299	0.065	2.258e-14	5.214e-10
13	Lymphatic endothelial cells	*SCARF1*	0.6598	0.313	0.008	1.843e-139	4.256e-135
13	Lymphatic endothelial cells	*COX4I1*	0.6572	0.985	0.917	7.395e-12	1.708e-07
13	Lymphatic endothelial cells	*KIF1B*	0.6571	0.552	0.251	1.207e-08	2.786e-04
13	Lymphatic endothelial cells	*SLC66A3*	0.6569	0.328	0.106	1.450e-09	3.349e-05
13	Lymphatic endothelial cells	*SMIM29*	0.6561	0.373	0.084	3.618e-17	8.355e-13
13	Lymphatic endothelial cells	*ARHGAP31*	0.6551	0.448	0.105	3.477e-18	8.029e-14
13	Lymphatic endothelial cells	*MMP16*	0.6528	0.478	0.169	2.295e-10	5.300e-06
13	Lymphatic endothelial cells	*TMEM160*	0.6523	0.403	0.089	2.245e-18	5.184e-14
13	Lymphatic endothelial cells	*DHRS3*	0.6522	0.313	0.060	1.324e-17	3.058e-13
13	Lymphatic endothelial cells	*EIF4G2*	0.6520	0.881	0.592	3.247e-09	7.498e-05
13	Lymphatic endothelial cells	*AGRN*	0.6500	0.284	0.052	4.830e-17	1.115e-12
13	Lymphatic endothelial cells	*SPTAN1*	0.6499	0.567	0.222	1.435e-11	3.314e-07
13	Lymphatic endothelial cells	*SLC25A5*	0.6497	0.687	0.394	6.170e-08	1.425e-03
13	Lymphatic endothelial cells	*SH3TC1*	0.6493	0.284	0.008	9.720e-117	2.245e-112
13	Lymphatic endothelial cells	*SLC41A1*	0.6488	0.358	0.041	3.067e-36	7.083e-32
13	Lymphatic endothelial cells	*MAP3K11*	0.6478	0.299	0.038	6.608e-28	1.526e-23
13	Lymphatic endothelial cells	*EFCC1*	0.6477	0.328	0.028	7.682e-47	1.774e-42
13	Lymphatic endothelial cells	*CEP19*	0.6477	0.403	0.088	2.411e-18	5.567e-14
13	Lymphatic endothelial cells	*PTTG1IP*	0.6465	0.806	0.526	9.053e-09	2.091e-04
13	Lymphatic endothelial cells	*CDC34*	0.6449	0.507	0.171	2.138e-12	4.937e-08
13	Lymphatic endothelial cells	*TMEM88*	0.6448	0.358	0.030	7.428e-50	1.715e-45
13	Lymphatic endothelial cells	*IQSEC1*	0.6448	0.373	0.094	7.860e-15	1.815e-10
13	Lymphatic endothelial cells	*CTSS*	0.6447	0.343	0.039	3.141e-35	7.254e-31
13	Lymphatic endothelial cells	*RALGAPA2*	0.6445	0.493	0.194	4.545e-09	1.050e-04
13	Lymphatic endothelial cells	*WDFY1*	0.6441	0.403	0.101	2.331e-15	5.384e-11
13	Lymphatic endothelial cells	*FNDC1*	0.6439	0.254	0.007	2.307e-104	5.326e-100
13	Lymphatic endothelial cells	*GRAP*	0.6431	0.239	0.009	1.252e-71	2.891e-67
13	Lymphatic endothelial cells	*RNASEK*	0.6430	0.642	0.350	5.382e-07	1.243e-02
13	Lymphatic endothelial cells	*SGIP1*	0.6422	0.343	0.030	7.518e-47	1.736e-42
13	Lymphatic endothelial cells	*ANKRD45*	0.6422	0.254	0.032	1.661e-23	3.835e-19
13	Lymphatic endothelial cells	*EFNB2*	0.6418	0.493	0.232	4.031e-07	9.308e-03
13	Lymphatic endothelial cells	*BCAM*	0.6413	0.433	0.128	2.943e-12	6.796e-08
13	Lymphatic endothelial cells	*LRRFIP1*	0.6412	0.582	0.203	2.359e-13	5.448e-09

Original cluster	Annotated cell type	Gene	avg_log2FC	pct.1	pct.2	Raw P value	Bonferroni-adjusted P value
13	Lymphatic endothelial cells	*NHSL2*	0.6394	0.478	0.152	9.493e-13	2.192e-08
13	Lymphatic endothelial cells	*MEGF6*	0.6383	0.254	0.004	1.246e-163	2.877e-159
13	Lymphatic endothelial cells	*SPNS2*	0.6378	0.224	0.004	8.559e-123	1.976e-118
13	Lymphatic endothelial cells	*LOC108176426*	0.6371	0.299	0.023	2.976e-46	6.872e-42
13	Lymphatic endothelial cells	*POLR2I*	0.6362	0.433	0.128	5.273e-13	1.218e-08
13	Lymphatic endothelial cells	*ANKRD6*	0.6341	0.328	0.014	6.977e-94	1.611e-89
13	Lymphatic endothelial cells	*UQCC4*	0.6340	0.463	0.165	5.867e-10	1.355e-05
13	Lymphatic endothelial cells	*CCNYL1*	0.6323	0.343	0.090	1.866e-12	4.308e-08
13	Lymphatic endothelial cells	*EMC1*	0.6321	0.328	0.093	4.631e-11	1.069e-06
13	Lymphatic endothelial cells	*SDCBP*	0.6307	0.821	0.640	3.467e-07	8.006e-03
13	Lymphatic endothelial cells	*TRIM47*	0.6306	0.284	0.067	1.768e-12	4.082e-08
13	Lymphatic endothelial cells	*LOC100344594*	0.6306	0.403	0.164	1.790e-07	4.133e-03
13	Lymphatic endothelial cells	*NFATC1*	0.6304	0.313	0.040	3.691e-28	8.523e-24
13	Lymphatic endothelial cells	*JCAD*	0.6278	0.373	0.076	1.252e-18	2.891e-14
13	Lymphatic endothelial cells	*GNB1*	0.6264	0.716	0.429	6.813e-07	1.573e-02
13	Lymphatic endothelial cells	*SLK*	0.6258	0.597	0.287	3.629e-07	8.380e-03
13	Lymphatic endothelial cells	*LMCD1*	0.6248	0.313	0.051	4.180e-21	9.653e-17
13	Lymphatic endothelial cells	*RAB11FIP3*	0.6247	0.299	0.049	3.208e-20	7.408e-16
13	Lymphatic endothelial cells	*CMTM8*	0.6235	0.343	0.051	1.896e-25	4.378e-21
13	Lymphatic endothelial cells	*RNASE4*	0.6234	0.970	0.762	2.456e-09	5.670e-05
13	Lymphatic endothelial cells	*RALGDS*	0.6224	0.418	0.087	2.615e-20	6.039e-16
13	Lymphatic endothelial cells	*WSB2*	0.6217	0.299	0.044	7.668e-23	1.771e-18
13	Lymphatic endothelial cells	*HOXB7*	0.6216	0.269	0.021	1.399e-42	3.231e-38
13	Lymphatic endothelial cells	*ADIPOR1*	0.6209	0.433	0.101	4.518e-18	1.043e-13
13	Lymphatic endothelial cells	*ITGB4*	0.6199	0.179	0.012	2.410e-32	5.566e-28
13	Lymphatic endothelial cells	*PLOD1*	0.6196	0.284	0.048	9.731e-19	2.247e-14
13	Lymphatic endothelial cells	*HIP1*	0.6192	0.463	0.111	3.332e-18	7.695e-14
13	Lymphatic endothelial cells	*TIMM13*	0.6189	0.612	0.264	2.661e-09	6.145e-05
13	Lymphatic endothelial cells	*CA8*	0.6185	0.284	0.008	8.919e-109	2.060e-104
13	Lymphatic endothelial cells	*ALAD*	0.6179	0.284	0.043	6.722e-21	1.552e-16
13	Lymphatic endothelial cells	*FAM110D*	0.6174	0.343	0.090	3.432e-12	7.925e-08
13	Lymphatic endothelial cells	*RCAN1*	0.6173	0.358	0.088	3.416e-14	7.888e-10
13	Lymphatic endothelial cells	*CCDC50*	0.6170	0.612	0.353	1.432e-06	3.306e-02
13	Lymphatic endothelial cells	*ARHGEF3*	0.6170	0.358	0.072	1.536e-18	3.546e-14
13	Lymphatic endothelial cells	*CYP2J2*	0.6147	0.448	0.160	2.399e-10	5.540e-06
13	Lymphatic endothelial cells	*PON3*	0.6139	0.254	0.008	7.773e-96	1.795e-91
13	Lymphatic endothelial cells	*EMP1*	0.6130	0.328	0.047	2.309e-25	5.331e-21
13	Lymphatic endothelial cells	*TPM3*	0.6107	0.478	0.188	2.527e-09	5.835e-05
13	Lymphatic endothelial cells	*PTPRE*	0.6096	0.373	0.044	8.999e-37	2.078e-32
13	Lymphatic endothelial cells	*RAB6B*	0.6081	0.179	0.022	5.378e-17	1.242e-12

Original cluster	Annotated cell type	Gene	avg_log2FC	pct.1	pct.2	Raw P value	Bonferroni-adjusted P value
13	Lymphatic endothelial cells	*UFC1*	0.6081	0.552	0.268	1.416e-07	3.271e-03
13	Lymphatic endothelial cells	*MRPL20*	0.6076	0.537	0.215	2.691e-09	6.213e-05
13	Lymphatic endothelial cells	*TOMM20*	0.6070	0.701	0.408	1.896e-07	4.379e-03
13	Lymphatic endothelial cells	*ACER3*	0.6067	0.463	0.157	3.209e-11	7.410e-07
13	Lymphatic endothelial cells	*SIGIRR*	0.6053	0.239	0.017	4.423e-42	1.021e-37
13	Lymphatic endothelial cells	*ECH1*	0.6046	0.328	0.087	1.027e-11	2.372e-07
13	Lymphatic endothelial cells	*EMC10*	0.6042	0.463	0.201	4.121e-08	9.516e-04
13	Lymphatic endothelial cells	*RTN3*	0.6041	0.597	0.282	1.006e-07	2.323e-03
13	Lymphatic endothelial cells	*SMARCA4*	0.6035	0.463	0.141	7.429e-13	1.716e-08
13	Lymphatic endothelial cells	*GNPTG*	0.6028	0.522	0.242	7.023e-08	1.622e-03
13	Lymphatic endothelial cells	*NDUFS6*	0.6012	0.343	0.114	1.077e-08	2.487e-04
13	Lymphatic endothelial cells	*NEDD4*	0.6007	0.388	0.105	2.468e-13	5.700e-09
13	Lymphatic endothelial cells	*SNX9*	0.5999	0.463	0.186	9.344e-09	2.158e-04
13	Lymphatic endothelial cells	*LOC108177130*	0.5995	0.388	0.105	2.693e-13	6.220e-09
13	Lymphatic endothelial cells	*SGK3*	0.5989	0.433	0.130	7.976e-13	1.842e-08
13	Lymphatic endothelial cells	*AP2A2*	0.5980	0.403	0.133	2.029e-10	4.684e-06
13	Lymphatic endothelial cells	*MARVELD1*	0.5973	0.373	0.146	7.600e-07	1.755e-02
13	Lymphatic endothelial cells	*BMP1*	0.5973	0.254	0.040	9.290e-19	2.145e-14
13	Lymphatic endothelial cells	*LOC108178853*	0.5972	0.209	0.007	8.482e-71	1.959e-66
13	Lymphatic endothelial cells	*CCDC141*	0.5967	0.269	0.009	5.769e-96	1.332e-91
13	Lymphatic endothelial cells	*LOC100354197*	0.5933	0.269	0.008	3.211e-104	7.414e-100
13	Lymphatic endothelial cells	*ORMDL2*	0.5925	0.343	0.054	3.144e-24	7.261e-20
13	Lymphatic endothelial cells	*COPE*	0.5924	0.567	0.268	4.617e-08	1.066e-03
13	Lymphatic endothelial cells	*RAP1B*	0.5924	0.507	0.190	5.215e-10	1.204e-05
13	Lymphatic endothelial cells	*RAPGEF5*	0.5911	0.284	0.042	1.881e-21	4.343e-17
13	Lymphatic endothelial cells	*EMCN*	0.5910	0.597	0.098	1.546e-36	3.570e-32
13	Lymphatic endothelial cells	*CARD10*	0.5910	0.299	0.014	3.774e-75	8.715e-71
13	Lymphatic endothelial cells	*TM4SF18*	0.5908	0.209	0.019	6.227e-29	1.438e-24
13	Lymphatic endothelial cells	*LOC138848553*	0.5908	0.284	0.045	4.653e-20	1.075e-15
13	Lymphatic endothelial cells	*CMPK1*	0.5895	0.388	0.148	2.194e-08	5.066e-04
13	Lymphatic endothelial cells	*CDC42EP2*	0.5885	0.343	0.050	9.579e-27	2.212e-22
13	Lymphatic endothelial cells	*EIF4EBP1*	0.5884	0.373	0.096	1.147e-13	2.649e-09
13	Lymphatic endothelial cells	*TSPAN12*	0.5883	0.284	0.023	1.443e-41	3.332e-37
13	Lymphatic endothelial cells	*CD81*	0.5864	1.000	0.949	8.662e-10	2.000e-05
13	Lymphatic endothelial cells	*SOX7*	0.5862	0.239	0.034	2.112e-19	4.877e-15
13	Lymphatic endothelial cells	*GNA12*	0.5861	0.478	0.162	6.676e-11	1.542e-06
13	Lymphatic endothelial cells	*TMEM203*	0.5855	0.493	0.219	1.176e-07	2.717e-03
13	Lymphatic endothelial cells	*HIC1*	0.5852	0.418	0.178	5.505e-07	1.271e-02
13	Lymphatic endothelial cells	*LRRC8A*	0.5848	0.507	0.159	2.833e-13	6.542e-09
13	Lymphatic endothelial cells	*GOLGA7*	0.5848	0.463	0.150	9.212e-12	2.127e-07

Original cluster	Annotated cell type	Gene	avg_log2FC	pct.1	pct.2	Raw P value	Bonferroni-adjusted P value
13	Lymphatic endothelial cells	*PRKAG2*	0.5836	0.522	0.206	1.050e-09	2.425e-05
13	Lymphatic endothelial cells	*HRAS*	0.5827	0.299	0.046	5.917e-22	1.366e-17
13	Lymphatic endothelial cells	*NFIA*	0.5824	0.866	0.633	8.133e-08	1.878e-03
13	Lymphatic endothelial cells	*LOC138843459*	0.5813	0.313	0.061	4.115e-17	9.503e-13
13	Lymphatic endothelial cells	*RAB6A*	0.5802	0.463	0.168	5.365e-10	1.239e-05
13	Lymphatic endothelial cells	*EPOR*	0.5802	0.269	0.023	1.425e-37	3.291e-33
13	Lymphatic endothelial cells	*PSMB3*	0.5801	0.507	0.210	1.150e-08	2.656e-04
13	Lymphatic endothelial cells	*LOC138847739*	0.5789	0.582	0.269	9.438e-09	2.180e-04
13	Lymphatic endothelial cells	*SSH2*	0.5789	0.567	0.195	1.006e-12	2.322e-08
13	Lymphatic endothelial cells	*KIF26A*	0.5783	0.269	0.014	5.697e-64	1.315e-59
13	Lymphatic endothelial cells	*LFNG*	0.5779	0.239	0.022	1.094e-31	2.526e-27
13	Lymphatic endothelial cells	*MICAL2*	0.5769	0.284	0.019	4.284e-50	9.893e-46
13	Lymphatic endothelial cells	*HOOK3*	0.5765	0.776	0.437	8.486e-08	1.960e-03
13	Lymphatic endothelial cells	*TMED10*	0.5761	0.731	0.431	7.563e-07	1.746e-02
13	Lymphatic endothelial cells	*CELSR1*	0.5759	0.224	0.011	3.000e-56	6.928e-52
13	Lymphatic endothelial cells	*LOC100349085*	0.5758	0.672	0.300	1.830e-10	4.227e-06
13	Lymphatic endothelial cells	*SLC22A23*	0.5751	0.254	0.007	8.135e-104	1.878e-99
13	Lymphatic endothelial cells	*RNF152*	0.5746	0.284	0.030	9.604e-32	2.218e-27
13	Lymphatic endothelial cells	*GADD45A*	0.5743	0.672	0.272	1.728e-12	3.991e-08
13	Lymphatic endothelial cells	*CDC14A*	0.5743	0.328	0.077	1.913e-13	4.418e-09
13	Lymphatic endothelial cells	*PTBP3*	0.5742	0.537	0.215	1.070e-09	2.470e-05
13	Lymphatic endothelial cells	*PRRG2*	0.5732	0.269	0.038	3.410e-22	7.873e-18
13	Lymphatic endothelial cells	*SLC38A2*	0.5730	0.478	0.200	2.742e-08	6.331e-04
13	Lymphatic endothelial cells	*SSNA1*	0.5729	0.313	0.091	7.546e-10	1.743e-05
13	Lymphatic endothelial cells	*WDR1*	0.5711	0.433	0.130	2.248e-12	5.191e-08
13	Lymphatic endothelial cells	*AMPD3*	0.5711	0.224	0.019	1.293e-32	2.987e-28
13	Lymphatic endothelial cells	*NDUFS7*	0.5710	0.418	0.130	2.168e-11	5.007e-07
13	Lymphatic endothelial cells	*PLEKHA3*	0.5699	0.493	0.141	6.629e-15	1.531e-10
13	Lymphatic endothelial cells	*APOLD1*	0.5688	0.448	0.167	5.553e-09	1.282e-04
13	Lymphatic endothelial cells	*BBC3*	0.5683	0.343	0.048	1.504e-27	3.474e-23
13	Lymphatic endothelial cells	*ATP5MF*	0.5680	0.612	0.289	3.985e-08	9.203e-04
13	Lymphatic endothelial cells	*LOC100328910*	0.5654	0.313	0.028	1.102e-41	2.545e-37
13	Lymphatic endothelial cells	*SF3B5*	0.5653	0.328	0.110	2.136e-08	4.932e-04
13	Lymphatic endothelial cells	*RGS19*	0.5641	0.284	0.037	3.303e-25	7.628e-21
13	Lymphatic endothelial cells	*LAMB1*	0.5640	0.388	0.096	9.268e-15	2.140e-10
13	Lymphatic endothelial cells	*CXXC5*	0.5634	0.493	0.227	1.231e-06	2.844e-02
13	Lymphatic endothelial cells	*FADS3*	0.5625	0.418	0.171	2.269e-07	5.241e-03
13	Lymphatic endothelial cells	*MTSS1*	0.5624	0.552	0.257	8.208e-08	1.895e-03
13	Lymphatic endothelial cells	*ANGPT2*	0.5612	0.313	0.096	8.667e-09	2.001e-04
13	Lymphatic endothelial cells	*TRMT9B*	0.5611	0.313	0.032	3.879e-36	8.958e-32

Original cluster	Annotated cell type	Gene	avg_log2FC	pct.1	pct.2	Raw P value	Bonferroni-adjusted P value
13	Lymphatic endothelial cells	*BCL7B*	0.5598	0.313	0.048	4.356e-23	1.006e-18
13	Lymphatic endothelial cells	*ATP13A3*	0.5585	0.313	0.101	1.243e-08	2.870e-04
13	Lymphatic endothelial cells	*RAB11FIP5*	0.5583	0.269	0.048	1.487e-16	3.433e-12
13	Lymphatic endothelial cells	*ZNF277*	0.5580	0.821	0.559	7.518e-07	1.736e-02
13	Lymphatic endothelial cells	*CLTC*	0.5566	0.522	0.261	5.587e-07	1.290e-02
13	Lymphatic endothelial cells	*PCBD1*	0.5554	0.313	0.122	1.410e-06	3.257e-02
13	Lymphatic endothelial cells	*EPDR1*	0.5553	0.254	0.004	2.134e-160	4.928e-156
13	Lymphatic endothelial cells	*CETP*	0.5547	0.179	0.000	0	0
13	Lymphatic endothelial cells	*B3GNT5*	0.5546	0.343	0.046	6.253e-29	1.444e-24
13	Lymphatic endothelial cells	*TMEM141*	0.5544	0.388	0.126	3.467e-10	8.007e-06
13	Lymphatic endothelial cells	*UNC13B*	0.5541	0.358	0.051	1.770e-28	4.087e-24
13	Lymphatic endothelial cells	*STRN*	0.5536	0.537	0.221	5.722e-09	1.321e-04
13	Lymphatic endothelial cells	*UAP1L1*	0.5534	0.209	0.038	7.161e-13	1.654e-08
13	Lymphatic endothelial cells	*DHRS11*	0.5526	0.328	0.064	1.002e-17	2.314e-13
13	Lymphatic endothelial cells	*NLN*	0.5524	0.403	0.127	9.314e-11	2.151e-06
13	Lymphatic endothelial cells	*SDF4*	0.5516	0.507	0.217	3.294e-08	7.606e-04
13	Lymphatic endothelial cells	*RASSF3*	0.5516	0.373	0.106	2.816e-12	6.502e-08
13	Lymphatic endothelial cells	*RNPEPL1*	0.5503	0.254	0.049	1.439e-14	3.323e-10
13	Lymphatic endothelial cells	*MAP3K3*	0.5502	0.493	0.219	9.722e-08	2.245e-03
13	Lymphatic endothelial cells	*TMEM158*	0.5498	0.358	0.098	7.128e-12	1.646e-07
13	Lymphatic endothelial cells	*ATL1*	0.5498	0.358	0.095	1.562e-12	3.606e-08
13	Lymphatic endothelial cells	*CDK2AP2*	0.5497	0.388	0.102	7.669e-14	1.771e-09
13	Lymphatic endothelial cells	*FAM241A*	0.5496	0.299	0.078	4.659e-11	1.076e-06
13	Lymphatic endothelial cells	*DPP8*	0.5482	0.269	0.093	1.285e-06	2.967e-02
13	Lymphatic endothelial cells	*LOC100353676*	0.5481	0.269	0.022	2.076e-38	4.793e-34
13	Lymphatic endothelial cells	*CHCHD2*	0.5461	0.836	0.613	5.991e-07	1.384e-02
13	Lymphatic endothelial cells	*CLIC4*	0.5460	0.701	0.360	2.142e-08	4.946e-04
13	Lymphatic endothelial cells	*RTL10*	0.5451	0.328	0.104	3.213e-09	7.419e-05
13	Lymphatic endothelial cells	*SNRK*	0.5445	0.433	0.127	5.898e-13	1.362e-08
13	Lymphatic endothelial cells	*FGD5*	0.5432	0.328	0.057	1.688e-20	3.898e-16
13	Lymphatic endothelial cells	*WLS*	0.5429	0.463	0.187	2.853e-08	6.588e-04
13	Lymphatic endothelial cells	*GCNT1*	0.5424	0.269	0.014	2.244e-60	5.182e-56
13	Lymphatic endothelial cells	*PSMA7*	0.5409	0.851	0.554	1.935e-06	4.468e-02
13	Lymphatic endothelial cells	*PGER4*	0.5398	0.149	0.013	4.647e-22	1.073e-17
13	Lymphatic endothelial cells	*CMIP*	0.5398	0.358	0.103	2.403e-11	5.550e-07
13	Lymphatic endothelial cells	*TMEM9B*	0.5391	0.269	0.077	1.252e-08	2.891e-04
13	Lymphatic endothelial cells	*DHCR7*	0.5370	0.224	0.047	1.287e-11	2.972e-07
13	Lymphatic endothelial cells	*EIF4G1*	0.5364	0.328	0.088	2.568e-11	5.931e-07
13	Lymphatic endothelial cells	*GPRC5B*	0.5356	0.224	0.025	4.954e-24	1.144e-19
13	Lymphatic endothelial cells	*MYO18A*	0.5356	0.313	0.050	1.756e-21	4.054e-17

Original cluster	Annotated cell type	Gene	avg_log2FC	pct.1	pct.2	Raw P value	Bonferroni-adjusted P value
13	Lymphatic endothelial cells	*BAG1*	0.5348	0.403	0.118	4.379e-12	1.011e-07
13	Lymphatic endothelial cells	*C16H19orf25*	0.5345	0.388	0.130	1.327e-09	3.064e-05
13	Lymphatic endothelial cells	*PACSIN3*	0.5336	0.224	0.058	9.273e-09	2.141e-04
13	Lymphatic endothelial cells	*LOC138844642*	0.5324	0.209	0.013	8.705e-43	2.010e-38
13	Lymphatic endothelial cells	*LOC100355171*	0.5323	0.299	0.031	1.645e-33	3.798e-29
13	Lymphatic endothelial cells	*PTPN11*	0.5315	0.299	0.093	1.270e-08	2.932e-04
13	Lymphatic endothelial cells	*SCG3*	0.5312	0.134	0.002	2.104e-100	4.858e-96
13	Lymphatic endothelial cells	*CDC42*	0.5302	0.866	0.632	1.536e-06	3.546e-02
13	Lymphatic endothelial cells	*UBTD1*	0.5295	0.224	0.024	8.484e-26	1.959e-21
13	Lymphatic endothelial cells	*NHSL1*	0.5289	0.299	0.069	4.487e-13	1.036e-08
13	Lymphatic endothelial cells	*SOD2*	0.5287	0.343	0.112	6.672e-09	1.541e-04
13	Lymphatic endothelial cells	*NDFIP2*	0.5286	0.537	0.255	1.241e-06	2.866e-02
13	Lymphatic endothelial cells	*PBXIP1*	0.5275	0.507	0.209	1.807e-08	4.174e-04
13	Lymphatic endothelial cells	*SYPL1*	0.5266	0.522	0.224	6.146e-08	1.419e-03
13	Lymphatic endothelial cells	*LBR*	0.5261	0.493	0.146	2.124e-13	4.905e-09
13	Lymphatic endothelial cells	*ARHGAP27*	0.5259	0.224	0.014	4.550e-43	1.051e-38
13	Lymphatic endothelial cells	*YY1*	0.5258	0.612	0.284	5.773e-08	1.333e-03
13	Lymphatic endothelial cells	*RAB13*	0.5240	0.537	0.231	6.588e-08	1.521e-03
13	Lymphatic endothelial cells	*LOC100008921*	0.5239	0.179	0.001	2.552e-195	5.894e-191
13	Lymphatic endothelial cells	*SKP2*	0.5232	0.313	0.079	8.930e-12	2.062e-07
13	Lymphatic endothelial cells	*THSD1*	0.5210	0.269	0.023	2.054e-37	4.742e-33
13	Lymphatic endothelial cells	*SPSB2*	0.5198	0.358	0.144	8.280e-07	1.912e-02
13	Lymphatic endothelial cells	*TMOD2*	0.5193	0.299	0.053	7.345e-18	1.696e-13
13	Lymphatic endothelial cells	*GRN*	0.5193	0.418	0.171	5.141e-07	1.187e-02
13	Lymphatic endothelial cells	*SQOR*	0.5192	0.418	0.132	1.008e-10	2.327e-06
13	Lymphatic endothelial cells	*CDV3*	0.5185	0.403	0.162	3.144e-07	7.261e-03
13	Lymphatic endothelial cells	*GPR4*	0.5182	0.164	0.006	3.897e-50	8.999e-46
13	Lymphatic endothelial cells	*KDM3A*	0.5170	0.373	0.113	1.322e-10	3.052e-06
13	Lymphatic endothelial cells	*SNX20*	0.5163	0.224	0.011	4.894e-57	1.130e-52
13	Lymphatic endothelial cells	*TBXA2R*	0.5160	0.194	0.002	7.863e-157	1.816e-152
13	Lymphatic endothelial cells	*C12H15orf61*	0.5155	0.388	0.158	6.189e-07	1.429e-02
13	Lymphatic endothelial cells	*TARS3*	0.5152	0.284	0.067	4.868e-12	1.124e-07
13	Lymphatic endothelial cells	*DAG1*	0.5139	0.373	0.151	9.242e-07	2.134e-02
13	Lymphatic endothelial cells	*CSGALNACT1*	0.5127	0.239	0.044	2.989e-14	6.903e-10
13	Lymphatic endothelial cells	*UBE2M*	0.5122	0.507	0.246	1.914e-06	4.420e-02
13	Lymphatic endothelial cells	*TNPO1*	0.5118	0.597	0.307	4.726e-07	1.091e-02
13	Lymphatic endothelial cells	*UBN1*	0.5113	0.343	0.101	2.973e-10	6.865e-06
13	Lymphatic endothelial cells	*LOC127492451*	0.5101	0.224	0.000	0	0
13	Lymphatic endothelial cells	*RELA*	0.5100	0.403	0.171	7.910e-07	1.827e-02
13	Lymphatic endothelial cells	*THSD4*	0.5093	0.403	0.089	1.141e-17	2.634e-13

Original cluster	Annotated cell type	Gene	avg_log2FC	pct.1	pct.2	Raw P value	Bonferroni-adjusted P value
13	Lymphatic endothelial cells	*GALNT18*	0.5068	0.299	0.075	2.058e-11	4.753e-07
13	Lymphatic endothelial cells	*ARF3*	0.5067	0.284	0.095	4.932e-07	1.139e-02
13	Lymphatic endothelial cells	*KIF1C*	0.5065	0.284	0.034	1.207e-27	2.788e-23
13	Lymphatic endothelial cells	*ACVRL1*	0.5055	0.254	0.035	3.326e-21	7.680e-17
13	Lymphatic endothelial cells	*TNFAIP1*	0.5044	0.209	0.037	1.686e-13	3.894e-09
13	Lymphatic endothelial cells	*RAI14*	0.5039	0.284	0.062	1.263e-13	2.916e-09
13	Lymphatic endothelial cells	*MID2*	0.5036	0.358	0.092	3.212e-13	7.418e-09
13	Lymphatic endothelial cells	*TMC6*	0.5028	0.194	0.018	3.484e-25	8.046e-21
13	Lymphatic endothelial cells	*RAB7A*	0.5025	0.701	0.330	8.723e-09	2.014e-04
13	Lymphatic endothelial cells	*PLTP*	0.5017	0.284	0.073	1.216e-10	2.808e-06
13	Lymphatic endothelial cells	*LOC108175596*	0.5006	0.284	0.069	8.022e-12	1.852e-07
13	Lymphatic endothelial cells	*CLPTM1L*	0.4999	0.388	0.142	3.422e-08	7.902e-04
13	Lymphatic endothelial cells	*PEDS1*	0.4998	0.209	0.029	1.161e-17	2.681e-13
13	Lymphatic endothelial cells	*FEZ2*	0.4991	0.388	0.129	1.325e-09	3.061e-05
13	Lymphatic endothelial cells	*SLC9A9*	0.4981	0.284	0.058	2.300e-14	5.311e-10
13	Lymphatic endothelial cells	*CCDC28B*	0.4978	0.343	0.115	1.485e-08	3.428e-04
13	Lymphatic endothelial cells	*ICAM2*	0.4974	0.254	0.040	2.629e-18	6.072e-14
13	Lymphatic endothelial cells	*MOSPD1*	0.4971	0.463	0.181	6.269e-08	1.448e-03
13	Lymphatic endothelial cells	*STX10*	0.4961	0.552	0.281	1.400e-06	3.232e-02
13	Lymphatic endothelial cells	*MERTK*	0.4956	0.239	0.056	2.043e-10	4.718e-06
13	Lymphatic endothelial cells	*LIG3*	0.4944	0.194	0.052	2.113e-07	4.879e-03
13	Lymphatic endothelial cells	*ATP6V1F*	0.4942	0.373	0.149	3.685e-07	8.510e-03
13	Lymphatic endothelial cells	*STK25*	0.4929	0.343	0.119	5.961e-08	1.377e-03
13	Lymphatic endothelial cells	*SETD7*	0.4927	0.328	0.107	1.204e-08	2.781e-04
13	Lymphatic endothelial cells	*SEMA3F*	0.4927	0.179	0.036	4.782e-10	1.104e-05
13	Lymphatic endothelial cells	*KPNB1*	0.4905	0.463	0.196	3.805e-07	8.786e-03
13	Lymphatic endothelial cells	*PRODH*	0.4902	0.179	0.022	8.675e-18	2.003e-13
13	Lymphatic endothelial cells	*LOC100350928*	0.4896	0.343	0.087	7.123e-13	1.645e-08
13	Lymphatic endothelial cells	*HSPB1*	0.4893	0.970	0.868	1.415e-07	3.268e-03
13	Lymphatic endothelial cells	*LOC100357058*	0.4892	0.388	0.152	1.990e-07	4.596e-03
13	Lymphatic endothelial cells	*FUCA1*	0.4886	0.522	0.205	9.106e-09	2.103e-04
13	Lymphatic endothelial cells	*PSMD2*	0.4884	0.269	0.093	1.544e-06	3.566e-02
13	Lymphatic endothelial cells	*GGTA1*	0.4873	0.478	0.185	9.609e-09	2.219e-04
13	Lymphatic endothelial cells	*RGL2*	0.4869	0.358	0.115	2.241e-09	5.174e-05
13	Lymphatic endothelial cells	*WNT9A*	0.4859	0.254	0.055	2.545e-12	5.878e-08
13	Lymphatic endothelial cells	*PSEN2*	0.4858	0.463	0.180	2.240e-08	5.172e-04
13	Lymphatic endothelial cells	*AOX1*	0.4851	0.343	0.115	1.257e-08	2.904e-04
13	Lymphatic endothelial cells	*BCL10*	0.4843	0.313	0.098	5.901e-09	1.363e-04
13	Lymphatic endothelial cells	*SLC27A3*	0.4843	0.284	0.098	7.168e-07	1.655e-02
13	Lymphatic endothelial cells	*ANXA7*	0.4841	0.552	0.214	2.120e-09	4.895e-05

Original cluster	Annotated cell type	Gene	avg_log2FC	pct.1	pct.2	Raw P value	Bonferroni-adjusted P value
13	Lymphatic endothelial cells	*PRELID3B*	0.4835	0.463	0.192	1.613e-07	3.724e-03
13	Lymphatic endothelial cells	*BLCAP*	0.4833	0.328	0.088	2.239e-11	5.169e-07
13	Lymphatic endothelial cells	*HOXD8*	0.4822	0.358	0.113	2.399e-09	5.539e-05
13	Lymphatic endothelial cells	*PLXNA4*	0.4815	0.194	0.009	7.244e-49	1.673e-44
13	Lymphatic endothelial cells	*IGSF3*	0.4808	0.313	0.072	1.898e-13	4.383e-09
13	Lymphatic endothelial cells	*GIT1*	0.4782	0.224	0.036	3.428e-16	7.916e-12
13	Lymphatic endothelial cells	*PAPLN*	0.4751	0.194	0.011	4.197e-40	9.691e-36
13	Lymphatic endothelial cells	*SNCAIP*	0.4750	0.373	0.120	1.170e-09	2.701e-05
13	Lymphatic endothelial cells	*DAZAP2*	0.4749	0.373	0.148	4.598e-07	1.062e-02
13	Lymphatic endothelial cells	*LOC103350201*	0.4728	0.209	0.020	5.959e-26	1.376e-21
13	Lymphatic endothelial cells	*HOMER3*	0.4721	0.209	0.058	1.990e-07	4.594e-03
13	Lymphatic endothelial cells	*TEC*	0.4720	0.448	0.151	3.821e-10	8.824e-06
13	Lymphatic endothelial cells	*PEAK1*	0.4718	0.522	0.195	4.369e-10	1.009e-05
13	Lymphatic endothelial cells	*CRIP1*	0.4716	0.537	0.210	1.613e-09	3.725e-05
13	Lymphatic endothelial cells	*CPSF4*	0.4709	0.284	0.058	2.709e-14	6.256e-10
13	Lymphatic endothelial cells	*CDC42BPB*	0.4696	0.373	0.134	5.615e-08	1.297e-03
13	Lymphatic endothelial cells	*SPATS2*	0.4694	0.343	0.117	2.965e-08	6.848e-04
13	Lymphatic endothelial cells	*TFEB*	0.4689	0.209	0.038	7.698e-13	1.778e-08
13	Lymphatic endothelial cells	*KLHL3*	0.4685	0.239	0.067	4.263e-08	9.843e-04
13	Lymphatic endothelial cells	*CCM2L*	0.4679	0.224	0.008	5.108e-73	1.180e-68
13	Lymphatic endothelial cells	*CCDC85B*	0.4677	0.388	0.134	8.838e-09	2.041e-04
13	Lymphatic endothelial cells	*LOC100342240*	0.4669	0.254	0.051	1.527e-13	3.526e-09
13	Lymphatic endothelial cells	*FLOT2*	0.4664	0.388	0.100	1.119e-13	2.585e-09
13	Lymphatic endothelial cells	*BCAR1*	0.4650	0.254	0.042	5.208e-17	1.203e-12
13	Lymphatic endothelial cells	*TANC1*	0.4643	0.403	0.172	2.107e-06	4.866e-02
13	Lymphatic endothelial cells	*UBA52*	0.4641	1.000	0.980	3.262e-10	7.533e-06
13	Lymphatic endothelial cells	*GARS1*	0.4638	0.343	0.101	2.733e-10	6.312e-06
13	Lymphatic endothelial cells	*BCL6B*	0.4634	0.209	0.014	1.167e-39	2.695e-35
13	Lymphatic endothelial cells	*ARHGAP23*	0.4629	0.269	0.070	3.271e-10	7.553e-06
13	Lymphatic endothelial cells	*HERPUD2*	0.4622	0.328	0.112	6.312e-08	1.457e-03
13	Lymphatic endothelial cells	*ANXA4*	0.4621	0.239	0.049	9.790e-13	2.261e-08
13	Lymphatic endothelial cells	*TMEM243*	0.4613	0.313	0.111	3.451e-07	7.970e-03
13	Lymphatic endothelial cells	*PLCD3*	0.4613	0.224	0.017	2.775e-35	6.408e-31
13	Lymphatic endothelial cells	*TSGA10*	0.4602	0.313	0.096	2.849e-09	6.579e-05
13	Lymphatic endothelial cells	*DGCR6L*	0.4589	0.239	0.076	5.813e-07	1.342e-02
13	Lymphatic endothelial cells	*LOC127493157*	0.4578	0.134	0.006	3.499e-39	8.080e-35
13	Lymphatic endothelial cells	*ARPC2*	0.4576	0.701	0.405	6.920e-07	1.598e-02
13	Lymphatic endothelial cells	*DSP*	0.4568	0.119	0.013	2.029e-14	4.685e-10
13	Lymphatic endothelial cells	*XPA*	0.4537	0.388	0.147	9.286e-08	2.144e-03
13	Lymphatic endothelial cells	*MKRN1*	0.4522	0.313	0.102	2.881e-08	6.652e-04

Original cluster	Annotated cell type	Gene	avg_log2FC	pct.1	pct.2	Raw P value	Bonferroni-adjusted P value
13	Lymphatic endothelial cells	*LAMC1*	0.4518	0.612	0.301	4.139e-07	9.558e-03
13	Lymphatic endothelial cells	*TRIB1*	0.4514	0.269	0.034	3.228e-24	7.454e-20
13	Lymphatic endothelial cells	*ARMCX4*	0.4510	0.328	0.073	2.774e-14	6.407e-10
13	Lymphatic endothelial cells	*SYT7*	0.4507	0.224	0.007	9.751e-84	2.252e-79
13	Lymphatic endothelial cells	*RASSF7*	0.4496	0.209	0.009	9.649e-61	2.228e-56
13	Lymphatic endothelial cells	*MTMR2*	0.4485	0.343	0.133	1.080e-06	2.494e-02
13	Lymphatic endothelial cells	*GPAT4*	0.4477	0.313	0.112	5.324e-07	1.229e-02
13	Lymphatic endothelial cells	*CILP*	0.4475	0.179	0.030	4.378e-12	1.011e-07
13	Lymphatic endothelial cells	*GMFB*	0.4467	0.254	0.062	2.679e-10	6.186e-06
13	Lymphatic endothelial cells	*SMTN*	0.4466	0.388	0.112	1.046e-11	2.416e-07
13	Lymphatic endothelial cells	*MED24*	0.4460	0.164	0.037	8.899e-08	2.055e-03
13	Lymphatic endothelial cells	*PLBD2*	0.4460	0.343	0.112	1.114e-08	2.572e-04
13	Lymphatic endothelial cells	*ADAM12*	0.4457	0.194	0.003	6.093e-128	1.407e-123
13	Lymphatic endothelial cells	*PLPP2*	0.4439	0.209	0.062	1.078e-06	2.489e-02
13	Lymphatic endothelial cells	*TWF1*	0.4438	0.343	0.125	1.730e-07	3.996e-03
13	Lymphatic endothelial cells	*SRM*	0.4437	0.209	0.047	6.293e-10	1.453e-05
13	Lymphatic endothelial cells	*VAMP5*	0.4436	0.269	0.061	6.977e-12	1.611e-07
13	Lymphatic endothelial cells	*CHN1*	0.4434	0.552	0.270	2.105e-06	4.862e-02
13	Lymphatic endothelial cells	*ABCF1*	0.4432	0.224	0.070	1.232e-06	2.844e-02
13	Lymphatic endothelial cells	*SLC43A2*	0.4423	0.164	0.028	2.937e-11	6.782e-07
13	Lymphatic endothelial cells	*TRIM16*	0.4411	0.209	0.014	4.036e-39	9.319e-35
13	Lymphatic endothelial cells	*MRPS25*	0.4408	0.328	0.118	3.108e-07	7.177e-03
13	Lymphatic endothelial cells	*MYO7A*	0.4400	0.209	0.003	3.576e-138	8.257e-134
13	Lymphatic endothelial cells	*RIN3*	0.4397	0.194	0.022	1.787e-20	4.127e-16
13	Lymphatic endothelial cells	*ZNF423*	0.4382	0.358	0.094	4.609e-12	1.064e-07
13	Lymphatic endothelial cells	*MYADM*	0.4381	0.821	0.535	1.909e-06	4.409e-02
13	Lymphatic endothelial cells	*ARF1*	0.4380	0.687	0.352	7.956e-07	1.837e-02
13	Lymphatic endothelial cells	*GLRX2*	0.4379	0.299	0.106	6.444e-07	1.488e-02
13	Lymphatic endothelial cells	*ASL*	0.4361	0.269	0.063	3.534e-11	8.161e-07
13	Lymphatic endothelial cells	*WNT2B*	0.4350	0.209	0.035	5.438e-14	1.256e-09
13	Lymphatic endothelial cells	*RHOG*	0.4346	0.299	0.060	3.926e-15	9.065e-11
13	Lymphatic endothelial cells	*EPHA2*	0.4340	0.239	0.025	1.470e-27	3.396e-23
13	Lymphatic endothelial cells	*PNP*	0.4336	0.418	0.149	8.275e-09	1.911e-04
13	Lymphatic endothelial cells	*YIPF2*	0.4324	0.269	0.065	4.097e-11	9.461e-07
13	Lymphatic endothelial cells	*DDOST*	0.4322	0.567	0.270	1.880e-06	4.341e-02
13	Lymphatic endothelial cells	*CNDP2*	0.4314	0.299	0.087	2.631e-09	6.077e-05
13	Lymphatic endothelial cells	*MEF2C*	0.4313	0.507	0.131	1.105e-15	2.552e-11
13	Lymphatic endothelial cells	*ITPRIP*	0.4311	0.254	0.059	3.031e-11	6.998e-07
13	Lymphatic endothelial cells	*PLA1A*	0.4294	0.194	0.002	1.207e-161	2.787e-157
13	Lymphatic endothelial cells	*RNF141*	0.4286	0.388	0.097	4.666e-14	1.077e-09

Original cluster	Annotated cell type	Gene	avg_log2FC	pct.1	pct.2	Raw P value	Bonferroni-adjusted P value
13	Lymphatic endothelial cells	*EEIG1*	0.4286	0.254	0.051	1.881e-13	4.344e-09
13	Lymphatic endothelial cells	*RAI2*	0.4283	0.299	0.069	9.329e-13	2.154e-08
13	Lymphatic endothelial cells	*MAP1S*	0.4282	0.254	0.064	7.168e-10	1.655e-05
13	Lymphatic endothelial cells	*ARHGEF15*	0.4282	0.194	0.015	2.815e-30	6.500e-26
13	Lymphatic endothelial cells	*MRPL9*	0.4277	0.373	0.137	6.839e-08	1.579e-03
13	Lymphatic endothelial cells	*AFAP1L1*	0.4266	0.224	0.012	1.653e-51	3.817e-47
13	Lymphatic endothelial cells	*PSMG3*	0.4266	0.358	0.110	7.211e-10	1.665e-05
13	Lymphatic endothelial cells	*PSMC4*	0.4264	0.254	0.053	1.416e-12	3.271e-08
13	Lymphatic endothelial cells	*CRELD1*	0.4248	0.224	0.035	3.668e-16	8.470e-12
13	Lymphatic endothelial cells	*OPTN*	0.4233	0.284	0.067	1.333e-11	3.078e-07
13	Lymphatic endothelial cells	*LOC138844553*	0.4224	0.179	0.006	1.819e-66	4.201e-62
13	Lymphatic endothelial cells	*CDK2AP1*	0.4214	0.269	0.062	9.761e-12	2.254e-07
13	Lymphatic endothelial cells	*FAM120B*	0.4202	0.224	0.071	1.650e-06	3.810e-02
13	Lymphatic endothelial cells	*IRAK2*	0.4172	0.373	0.114	5.575e-10	1.287e-05
13	Lymphatic endothelial cells	*ENTREP2*	0.4163	0.164	0.013	2.966e-26	6.849e-22
13	Lymphatic endothelial cells	*CDC42SE2*	0.4147	0.313	0.091	1.092e-09	2.522e-05
13	Lymphatic endothelial cells	*AGAP1*	0.4145	0.388	0.138	2.459e-08	5.679e-04
13	Lymphatic endothelial cells	*DRAM1*	0.4138	0.209	0.042	2.556e-11	5.901e-07
13	Lymphatic endothelial cells	*LOC103350187*	0.4137	0.194	0.009	1.176e-48	2.716e-44
13	Lymphatic endothelial cells	*LOC138850176*	0.4136	0.164	0.027	9.263e-12	2.139e-07
13	Lymphatic endothelial cells	*LEPROT*	0.4132	0.299	0.099	1.705e-07	3.938e-03
13	Lymphatic endothelial cells	*NUDT16L1*	0.4124	0.343	0.121	1.546e-07	3.570e-03
13	Lymphatic endothelial cells	*LOC103347040*	0.4123	0.209	0.022	3.222e-24	7.441e-20
13	Lymphatic endothelial cells	*MTHFD1L*	0.4115	0.284	0.099	1.174e-06	2.711e-02
13	Lymphatic endothelial cells	*ARHGEF7*	0.4113	0.313	0.106	1.604e-07	3.704e-03
13	Lymphatic endothelial cells	*BLVRA*	0.4104	0.209	0.028	3.818e-18	8.816e-14
13	Lymphatic endothelial cells	*G6PC3*	0.4100	0.299	0.056	1.078e-16	2.489e-12
13	Lymphatic endothelial cells	*KEF51-p11*	0.4099	0.985	0.993	6.716e-07	1.551e-02
13	Lymphatic endothelial cells	*ARHGEF40*	0.4096	0.224	0.070	1.768e-06	4.082e-02
13	Lymphatic endothelial cells	*PLCB4*	0.4093	0.313	0.088	1.523e-09	3.517e-05
13	Lymphatic endothelial cells	*LOC103350039*	0.4079	0.179	0.004	2.837e-86	6.552e-82
13	Lymphatic endothelial cells	*CISD3*	0.4070	0.284	0.101	1.622e-06	3.746e-02
13	Lymphatic endothelial cells	*B3GNT7*	0.4068	0.194	0.008	4.342e-54	1.003e-49
13	Lymphatic endothelial cells	*HOXB3*	0.4064	0.269	0.050	4.133e-15	9.544e-11
13	Lymphatic endothelial cells	*LOC138849320*	0.4063	0.164	0.030	3.910e-10	9.030e-06
13	Lymphatic endothelial cells	*TWF2*	0.4060	0.328	0.110	4.856e-08	1.121e-03
13	Lymphatic endothelial cells	*LOC138845996*	0.4059	0.254	0.058	5.411e-11	1.250e-06
13	Lymphatic endothelial cells	*PUS7*	0.4051	0.194	0.040	2.783e-10	6.426e-06
13	Lymphatic endothelial cells	*HACD4*	0.4048	0.239	0.044	3.531e-14	8.154e-10
13	Lymphatic endothelial cells	*BRD9*	0.4040	0.224	0.060	2.497e-08	5.765e-04

Original cluster	Annotated cell type	Gene	avg_log2FC	pct.1	pct.2	Raw P value	Bonferroni-adjusted P value
13	Lymphatic endothelial cells	*LOC108177265*	0.4036	0.149	0.001	6.757e-187	1.560e-182
13	Lymphatic endothelial cells	*DGKZ*	0.4023	0.134	0.028	1.963e-07	4.533e-03
13	Lymphatic endothelial cells	*TMEM140*	0.4008	0.239	0.040	8.706e-16	2.010e-11
13	Lymphatic endothelial cells	*ANGPTL2*	0.3989	0.269	0.069	6.561e-10	1.515e-05
13	Lymphatic endothelial cells	*HAPLN1*	0.3989	0.134	0.001	1.508e-142	3.482e-138
13	Lymphatic endothelial cells	*LOC138846933*	0.3976	0.119	0.004	9.955e-42	2.299e-37
13	Lymphatic endothelial cells	*E2F4*	0.3972	0.239	0.064	1.117e-08	2.579e-04
13	Lymphatic endothelial cells	*SLC2A13*	0.3971	0.343	0.134	1.544e-06	3.564e-02
13	Lymphatic endothelial cells	*SLCO2B1*	0.3962	0.209	0.003	1.158e-137	2.673e-133
13	Lymphatic endothelial cells	*PCDH12*	0.3956	0.209	0.012	3.073e-45	7.096e-41
13	Lymphatic endothelial cells	*MRPL28*	0.3956	0.328	0.105	1.399e-08	3.230e-04
13	Lymphatic endothelial cells	*EIF2AK3*	0.3952	0.284	0.078	1.652e-09	3.814e-05
13	Lymphatic endothelial cells	*ITGA5*	0.3947	0.164	0.026	2.669e-12	6.162e-08
13	Lymphatic endothelial cells	*PKN1*	0.3942	0.284	0.084	1.101e-08	2.543e-04
13	Lymphatic endothelial cells	*TSPAN11*	0.3940	0.149	0.012	1.103e-22	2.548e-18
13	Lymphatic endothelial cells	*MRPL58*	0.3928	0.284	0.090	1.327e-07	3.063e-03
13	Lymphatic endothelial cells	*RPS21*	0.3891	0.970	0.982	1.580e-08	3.649e-04
13	Lymphatic endothelial cells	*TPCN1*	0.3883	0.209	0.059	4.331e-07	1.000e-02
13	Lymphatic endothelial cells	*PPP2CB*	0.3879	0.522	0.230	4.318e-07	9.971e-03
13	Lymphatic endothelial cells	*EPHB1*	0.3877	0.164	0.002	2.088e-108	4.821e-104
13	Lymphatic endothelial cells	*HYAL3*	0.3855	0.119	0.008	3.185e-22	7.355e-18
13	Lymphatic endothelial cells	*CPOX*	0.3849	0.224	0.064	1.713e-07	3.955e-03
13	Lymphatic endothelial cells	*SPRING1*	0.3848	0.269	0.082	7.051e-08	1.628e-03
13	Lymphatic endothelial cells	*NUDT14*	0.3840	0.254	0.073	2.777e-08	6.413e-04
13	Lymphatic endothelial cells	*EHD4*	0.3838	0.239	0.067	4.447e-08	1.027e-03
13	Lymphatic endothelial cells	*ELOVL1*	0.3837	0.224	0.035	5.969e-16	1.378e-11
13	Lymphatic endothelial cells	*NCK2*	0.3831	0.418	0.137	7.469e-10	1.725e-05
13	Lymphatic endothelial cells	*ABHD5*	0.3824	0.254	0.066	2.429e-09	5.610e-05
13	Lymphatic endothelial cells	*KEF51-p10*	0.3777	1.000	0.994	1.124e-07	2.596e-03
13	Lymphatic endothelial cells	*ADRM1*	0.3773	0.373	0.142	1.125e-06	2.598e-02
13	Lymphatic endothelial cells	*SLC45A3*	0.3769	0.209	0.003	2.370e-144	5.473e-140
13	Lymphatic endothelial cells	*ZSWIM8*	0.3752	0.239	0.055	1.871e-10	4.320e-06
13	Lymphatic endothelial cells	*EMID1*	0.3749	0.224	0.053	1.723e-09	3.978e-05
13	Lymphatic endothelial cells	*BCO2*	0.3737	0.164	0.008	6.245e-41	1.442e-36
13	Lymphatic endothelial cells	*CKM*	0.3731	0.104	0.012	1.940e-11	4.481e-07
13	Lymphatic endothelial cells	*MXD4*	0.3730	0.254	0.064	8.114e-10	1.874e-05
13	Lymphatic endothelial cells	*MMRN2*	0.3718	0.179	0.014	2.103e-28	4.857e-24
13	Lymphatic endothelial cells	*CNIH1*	0.3714	0.328	0.115	2.015e-07	4.654e-03
13	Lymphatic endothelial cells	*CFAP54*	0.3712	0.134	0.019	6.604e-12	1.525e-07
13	Lymphatic endothelial cells	*USP6NL*	0.3712	0.239	0.071	2.048e-07	4.730e-03

Original cluster	Annotated cell type	Gene	avg_log2FC	pct.1	pct.2	Raw P value	Bonferroni-adjusted P value
13	Lymphatic endothelial cells	*ZBTB38*	0.3683	0.418	0.165	3.008e-07	6.947e-03
13	Lymphatic endothelial cells	*FLNB*	0.3655	0.358	0.134	5.029e-07	1.161e-02
13	Lymphatic endothelial cells	*LOC100340453*	0.3652	0.254	0.081	7.131e-07	1.647e-02
13	Lymphatic endothelial cells	*HSPA12B*	0.3652	0.269	0.074	5.026e-09	1.161e-04
13	Lymphatic endothelial cells	*LOC138844436*	0.3648	0.134	0.017	1.954e-13	4.511e-09
13	Lymphatic endothelial cells	*TMEM234*	0.3633	0.194	0.055	1.080e-06	2.495e-02
13	Lymphatic endothelial cells	*IFNGR2*	0.3631	0.194	0.020	4.492e-22	1.037e-17
13	Lymphatic endothelial cells	*BTD*	0.3628	0.239	0.069	1.613e-07	3.724e-03
13	Lymphatic endothelial cells	*INKA1*	0.3626	0.224	0.047	2.767e-11	6.389e-07
13	Lymphatic endothelial cells	*LOC138846506*	0.3615	0.224	0.055	2.157e-09	4.981e-05
13	Lymphatic endothelial cells	*TBC1D19*	0.3614	0.313	0.109	4.104e-07	9.476e-03
13	Lymphatic endothelial cells	*MTOR*	0.3609	0.239	0.044	7.760e-14	1.792e-09
13	Lymphatic endothelial cells	*GAS2L1*	0.3602	0.119	0.019	4.716e-09	1.089e-04
13	Lymphatic endothelial cells	*COL4A4*	0.3602	0.179	0.035	2.726e-10	6.295e-06
13	Lymphatic endothelial cells	*TULP4*	0.3595	0.343	0.131	1.533e-06	3.541e-02
13	Lymphatic endothelial cells	*TMEM184B*	0.3582	0.194	0.049	8.283e-08	1.913e-03
13	Lymphatic endothelial cells	*NRARP*	0.3579	0.284	0.051	1.123e-16	2.592e-12
13	Lymphatic endothelial cells	*DCLK2*	0.3577	0.179	0.030	1.460e-12	3.372e-08
13	Lymphatic endothelial cells	*ANKS1A*	0.3569	0.433	0.166	1.641e-07	3.790e-03
13	Lymphatic endothelial cells	*FGD2*	0.3568	0.119	0.002	7.877e-87	1.819e-82
13	Lymphatic endothelial cells	*DENND2C*	0.3567	0.254	0.057	1.856e-11	4.286e-07
13	Lymphatic endothelial cells	*EIF2AK1*	0.3563	0.343	0.104	2.641e-09	6.098e-05
13	Lymphatic endothelial cells	*SUSD6*	0.3558	0.269	0.089	8.236e-07	1.902e-02
13	Lymphatic endothelial cells	*SLC12A7*	0.3553	0.179	0.033	4.128e-11	9.533e-07
13	Lymphatic endothelial cells	*LOC100345135*	0.3530	0.134	0.005	1.652e-45	3.814e-41
13	Lymphatic endothelial cells	*LOC138844625*	0.3528	0.209	0.029	2.753e-17	6.358e-13
13	Lymphatic endothelial cells	*DNAJC14*	0.3521	0.254	0.077	1.264e-07	2.919e-03
13	Lymphatic endothelial cells	*CREB5*	0.3520	0.448	0.175	9.031e-08	2.085e-03
13	Lymphatic endothelial cells	*PIEZO1*	0.3515	0.194	0.036	1.802e-11	4.161e-07
13	Lymphatic endothelial cells	*GATAD2B*	0.3507	0.403	0.153	2.581e-07	5.960e-03
13	Lymphatic endothelial cells	*AP2A1*	0.3499	0.254	0.080	4.225e-07	9.755e-03
13	Lymphatic endothelial cells	*SERTAD2*	0.3495	0.388	0.149	7.579e-07	1.750e-02
13	Lymphatic endothelial cells	*PDRG1*	0.3483	0.224	0.062	7.914e-08	1.827e-03
13	Lymphatic endothelial cells	*NATD1*	0.3477	0.194	0.047	3.581e-08	8.268e-04
13	Lymphatic endothelial cells	*CXCL1*	0.3474	0.164	0.010	3.298e-32	7.615e-28
13	Lymphatic endothelial cells	*LOC103348070*	0.3473	0.149	0.002	1.687e-95	3.896e-91
13	Lymphatic endothelial cells	*MBNL3*	0.3463	0.179	0.014	9.020e-28	2.083e-23
13	Lymphatic endothelial cells	*TRIL*	0.3459	0.269	0.081	1.020e-07	2.356e-03
13	Lymphatic endothelial cells	*CCDC92*	0.3459	0.149	0.007	5.765e-40	1.331e-35
13	Lymphatic endothelial cells	*TRIB2*	0.3457	0.194	0.014	3.951e-33	9.125e-29

Original cluster	Annotated cell type	Gene	avg_log2FC	pct.1	pct.2	Raw P value	Bonferroni-adjusted P value
13	Lymphatic endothelial cells	*MET*	0.3441	0.209	0.035	3.270e-14	7.551e-10
13	Lymphatic endothelial cells	*CYYR1*	0.3439	0.239	0.036	2.996e-17	6.919e-13
13	Lymphatic endothelial cells	*RASGRP2*	0.3430	0.179	0.024	7.595e-16	1.754e-11
13	Lymphatic endothelial cells	*NDUFS8*	0.3429	0.194	0.038	5.279e-11	1.219e-06
13	Lymphatic endothelial cells	*DOCK6*	0.3413	0.179	0.047	5.672e-07	1.310e-02
13	Lymphatic endothelial cells	*CASP6*	0.3407	0.239	0.061	4.503e-09	1.040e-04
13	Lymphatic endothelial cells	*FLII*	0.3401	0.134	0.015	1.900e-15	4.388e-11
13	Lymphatic endothelial cells	*JAK3*	0.3399	0.164	0.018	1.408e-18	3.251e-14
13	Lymphatic endothelial cells	*ATP6V0A2*	0.3382	0.209	0.065	2.080e-06	4.804e-02
13	Lymphatic endothelial cells	*PIGQ*	0.3380	0.164	0.034	5.980e-09	1.381e-04
13	Lymphatic endothelial cells	*PPL*	0.3368	0.149	0.028	4.469e-09	1.032e-04
13	Lymphatic endothelial cells	*NOVA2*	0.3361	0.179	0.006	4.471e-62	1.032e-57
13	Lymphatic endothelial cells	*CDKL1*	0.3358	0.179	0.030	2.065e-12	4.769e-08
13	Lymphatic endothelial cells	*GET4*	0.3354	0.179	0.047	5.677e-07	1.311e-02
13	Lymphatic endothelial cells	*ACKR2*	0.3354	0.104	0.000	7.367e-134	1.701e-129
13	Lymphatic endothelial cells	*TCIRG1*	0.3347	0.209	0.062	1.320e-06	3.047e-02
13	Lymphatic endothelial cells	*NEU1*	0.3340	0.134	0.027	1.063e-07	2.455e-03
13	Lymphatic endothelial cells	*MAB21L3*	0.3336	0.149	0.013	3.374e-21	7.790e-17
13	Lymphatic endothelial cells	*ARHGEF10*	0.3334	0.209	0.038	8.499e-13	1.963e-08
13	Lymphatic endothelial cells	*C9*	0.3319	0.149	0.004	5.094e-65	1.176e-60
13	Lymphatic endothelial cells	*NANS*	0.3313	0.284	0.081	7.621e-09	1.760e-04
13	Lymphatic endothelial cells	*TRIM21*	0.3308	0.194	0.031	1.006e-13	2.324e-09
13	Lymphatic endothelial cells	*ABLIM3*	0.3308	0.239	0.036	9.520e-18	2.198e-13
13	Lymphatic endothelial cells	*ZDHHC7*	0.3306	0.254	0.081	1.333e-06	3.079e-02
13	Lymphatic endothelial cells	*UNC93B1*	0.3306	0.224	0.049	1.040e-10	2.403e-06
13	Lymphatic endothelial cells	*MFSD4A*	0.3304	0.164	0.007	3.516e-46	8.119e-42
13	Lymphatic endothelial cells	*LOC108175735*	0.3303	0.149	0.017	1.300e-15	3.001e-11
13	Lymphatic endothelial cells	*RASGRF2*	0.3289	0.164	0.005	8.699e-61	2.009e-56
13	Lymphatic endothelial cells	*CASTOR1*	0.3265	0.254	0.082	1.013e-06	2.339e-02
13	Lymphatic endothelial cells	*LOC138845018*	0.3262	0.209	0.030	4.419e-17	1.021e-12
13	Lymphatic endothelial cells	*LNX2*	0.3257	0.149	0.036	1.043e-06	2.409e-02
13	Lymphatic endothelial cells	*UCK1*	0.3255	0.284	0.086	3.975e-08	9.179e-04
13	Lymphatic endothelial cells	*MAN2A1*	0.3237	0.537	0.223	2.098e-07	4.846e-03
13	Lymphatic endothelial cells	*DENND3*	0.3233	0.104	0.008	6.249e-17	1.443e-12
13	Lymphatic endothelial cells	*LOC138845367*	0.3221	0.104	0.008	9.978e-17	2.304e-12
13	Lymphatic endothelial cells	*ST3GAL6*	0.3220	0.343	0.105	7.262e-09	1.677e-04
13	Lymphatic endothelial cells	*PEX26*	0.3219	0.149	0.021	1.085e-12	2.505e-08
13	Lymphatic endothelial cells	*PKHD1L1*	0.3202	0.119	0.003	3.433e-48	7.928e-44
13	Lymphatic endothelial cells	*PPP6R1*	0.3197	0.239	0.071	2.227e-07	5.142e-03
13	Lymphatic endothelial cells	*UNC93A*	0.3194	0.104	0.003	1.457e-41	3.365e-37

Original cluster	Annotated cell type	Gene	avg_log2FC	pct.1	pct.2	Raw P value	Bonferroni-adjusted P value
13	Lymphatic endothelial cells	*RPAIN*	0.3193	0.164	0.028	5.827e-11	1.346e-06
13	Lymphatic endothelial cells	*SMIM14*	0.3189	0.299	0.104	7.497e-07	1.731e-02
13	Lymphatic endothelial cells	*TOR1AIP2*	0.3183	0.269	0.068	4.321e-10	9.978e-06
13	Lymphatic endothelial cells	*GRAMD4*	0.3177	0.179	0.033	1.582e-10	3.653e-06
13	Lymphatic endothelial cells	*TUSC2*	0.3169	0.269	0.075	1.020e-08	2.354e-04
13	Lymphatic endothelial cells	*RAPGEF3*	0.3157	0.164	0.023	8.585e-14	1.982e-09
13	Lymphatic endothelial cells	*FIBCD1*	0.3151	0.119	0.001	7.866e-128	1.816e-123
13	Lymphatic endothelial cells	*TMEM255B*	0.3144	0.149	0.022	6.730e-12	1.554e-07
13	Lymphatic endothelial cells	*FOXK1*	0.3135	0.299	0.097	2.209e-07	5.101e-03
13	Lymphatic endothelial cells	*ABI3*	0.3122	0.134	0.011	9.159e-21	2.115e-16
13	Lymphatic endothelial cells	*FZD8*	0.3084	0.179	0.029	1.095e-12	2.528e-08
13	Lymphatic endothelial cells	*VPS53*	0.3081	0.328	0.108	8.318e-08	1.921e-03
13	Lymphatic endothelial cells	*PTRHD1*	0.3077	0.224	0.062	1.324e-07	3.058e-03
13	Lymphatic endothelial cells	*HPSE*	0.3069	0.164	0.003	9.276e-87	2.142e-82
13	Lymphatic endothelial cells	*TRPC3*	0.3059	0.164	0.018	7.386e-18	1.706e-13
13	Lymphatic endothelial cells	*GPSM2*	0.3045	0.239	0.060	3.676e-09	8.489e-05
13	Lymphatic endothelial cells	*HECW2*	0.3020	0.269	0.050	1.007e-14	2.326e-10
13	Lymphatic endothelial cells	*ZMAT4*	0.3020	0.149	0.006	2.680e-47	6.190e-43
13	Lymphatic endothelial cells	*KCNJ3*	0.2993	0.239	0.064	2.926e-08	6.757e-04
13	Lymphatic endothelial cells	*CCDC69*	0.2985	0.134	0.015	1.478e-14	3.413e-10
13	Lymphatic endothelial cells	*BCAR3*	0.2977	0.194	0.032	2.724e-13	6.289e-09
13	Lymphatic endothelial cells	*RASSF9*	0.2927	0.149	0.026	4.272e-10	9.864e-06
13	Lymphatic endothelial cells	*DUSP5*	0.2913	0.254	0.073	4.556e-08	1.052e-03
13	Lymphatic endothelial cells	*SLC7A7*	0.2854	0.179	0.040	2.251e-08	5.199e-04
13	Lymphatic endothelial cells	*MAP7D1*	0.2850	0.269	0.090	1.150e-06	2.656e-02
13	Lymphatic endothelial cells	*CEP57L1*	0.2827	0.164	0.041	7.831e-07	1.808e-02
13	Lymphatic endothelial cells	*RIT1*	0.2827	0.224	0.067	1.112e-06	2.568e-02
13	Lymphatic endothelial cells	*DAPK2*	0.2820	0.104	0.016	2.992e-08	6.908e-04
13	Lymphatic endothelial cells	*RAPH1*	0.2801	0.328	0.112	1.779e-07	4.108e-03
13	Lymphatic endothelial cells	*CD14*	0.2800	0.104	0.019	4.962e-07	1.146e-02
13	Lymphatic endothelial cells	*CCDC102B*	0.2791	0.269	0.058	2.701e-12	6.237e-08
13	Lymphatic endothelial cells	*ARHGAP25*	0.2771	0.149	0.023	2.468e-11	5.698e-07
13	Lymphatic endothelial cells	*CCDC88C*	0.2770	0.149	0.019	9.658e-15	2.230e-10
13	Lymphatic endothelial cells	*ACLY*	0.2758	0.254	0.082	1.071e-06	2.473e-02
13	Lymphatic endothelial cells	*LOC138845376*	0.2739	0.149	0.012	5.477e-23	1.265e-18
13	Lymphatic endothelial cells	*TMEM150C*	0.2731	0.239	0.076	1.519e-06	3.508e-02
13	Lymphatic endothelial cells	*TUBB4A*	0.2704	0.119	0.013	1.305e-13	3.014e-09
13	Lymphatic endothelial cells	*ABCB1*	0.2701	0.269	0.052	4.001e-14	9.240e-10
13	Lymphatic endothelial cells	*SNRNP48*	0.2689	0.224	0.069	1.678e-06	3.874e-02
13	Lymphatic endothelial cells	*PEPD*	0.2688	0.209	0.059	4.972e-07	1.148e-02

Original cluster	Annotated cell type	Gene	avg_log2FC	pct.1	pct.2	Raw P value	Bonferroni-adjusted P value
13	Lymphatic endothelial cells	*GSR*	0.2676	0.224	0.068	1.266e-06	2.923e-02
13	Lymphatic endothelial cells	*TMEM273*	0.2654	0.164	0.018	7.842e-18	1.811e-13
13	Lymphatic endothelial cells	*ING2*	0.2653	0.269	0.082	2.142e-07	4.947e-03
13	Lymphatic endothelial cells	*LATS2*	0.2640	0.239	0.076	1.513e-06	3.493e-02
13	Lymphatic endothelial cells	*UBE2E2*	0.2633	0.403	0.161	2.116e-06	4.887e-02
13	Lymphatic endothelial cells	*SHISA4*	0.2623	0.119	0.017	5.067e-10	1.170e-05
13	Lymphatic endothelial cells	*ELOVL7*	0.2617	0.179	0.026	3.293e-14	7.605e-10
13	Lymphatic endothelial cells	*LOC100328948*	0.2616	0.149	0.000	5.782e-214	1.335e-209
13	Lymphatic endothelial cells	*SLC31A2*	0.2610	0.179	0.040	1.453e-08	3.356e-04
13	Lymphatic endothelial cells	*TNC*	0.2593	0.104	0.008	3.432e-18	7.926e-14
13	Lymphatic endothelial cells	*CPTP*	0.2566	0.134	0.031	1.428e-06	3.298e-02
13	Lymphatic endothelial cells	*BAZ1A*	0.2521	0.239	0.063	1.225e-08	2.829e-04
13	Lymphatic endothelial cells	*EPSTI1*	0.2516	0.209	0.055	1.453e-07	3.355e-03
13	Lymphatic endothelial cells	*LOC127492685*	0.2515	0.104	0.001	5.267e-98	1.216e-93
13	Lymphatic endothelial cells	*LOC127491011*	0.2509	0.209	0.061	1.476e-06	3.409e-02
13	Lymphatic endothelial cells	*FUBP3*	0.2503	0.224	0.067	8.764e-07	2.024e-02
14	**Glial cells**	*SCN7A*	6.0753	1.000	0.003	0	0
14	Glial cells	*CADM2*	5.8952	0.985	0.181	3.655e-91	8.439e-87
14	Glial cells	*ABCA8*	5.2430	1.000	0.039	0	0
14	Glial cells	*CNTNAP2*	5.2313	0.788	0.038	4.281e-212	9.886e-208
14	Glial cells	*CDH19*	5.0434	0.985	0.002	0	0
14	Glial cells	*PLP1*	4.1942	0.924	0.001	0	0
14	Glial cells	*STARD13*	3.9369	0.939	0.217	2.052e-69	4.738e-65
14	Glial cells	*GRIK2*	3.9327	0.803	0.005	0	0
14	Glial cells	*MPZ*	3.7927	0.909	0.000	0	0
14	Glial cells	*S100B*	3.5319	0.864	0.002	0	0
14	Glial cells	*PMP22*	3.4829	0.939	0.461	8.655e-42	1.999e-37
14	Glial cells	*MDGA2*	3.4574	0.667	0.030	2.240e-183	5.172e-179
14	Glial cells	*MLIP*	3.4316	0.773	0.001	0	0
14	Glial cells	*TENM1*	3.2975	0.667	0.064	2.902e-94	6.701e-90
14	Glial cells	*NCAM2*	3.2594	0.636	0.045	5.605e-117	1.294e-112
14	Glial cells	*FGL2*	3.1990	0.758	0.174	2.755e-46	6.361e-42
14	Glial cells	*MAP2*	3.1772	0.864	0.127	2.593e-87	5.987e-83
14	Glial cells	*FRMD4A*	3.1629	0.833	0.194	7.183e-56	1.659e-51
14	Glial cells	*CRYAB*	3.1628	0.758	0.075	1.652e-103	3.814e-99
14	Glial cells	*TENM4*	3.0915	0.864	0.296	3.096e-42	7.150e-38
14	Glial cells	*SLC35F1*	3.0597	0.939	0.417	2.178e-41	5.030e-37
14	Glial cells	*NRG3*	2.9443	0.424	0.022	1.202e-102	2.775e-98
14	Glial cells	*CST3*	2.9289	0.788	0.375	9.098e-27	2.101e-22
14	Glial cells	*ANK3*	2.8913	0.879	0.249	3.459e-50	7.988e-46

Original cluster	Annotated cell type	Gene	avg_log2FC	pct.1	pct.2	Raw P value	Bonferroni-adjusted P value
14	Glial cells	*FIGN*	2.8814	0.788	0.107	6.050e-81	1.397e-76
14	Glial cells	*XKR4*	2.8454	0.591	0.014	2.341e-280	5.406e-276
14	Glial cells	*CHL1*	2.8076	0.652	0.008	0	0
14	Glial cells	*NLGN1*	2.8062	0.712	0.122	4.729e-55	1.092e-50
14	Glial cells	*LOC103350276*	2.8012	0.788	0.000	0	0
14	Glial cells	*FOXD3*	2.7813	0.652	0.000	0	0
14	Glial cells	*IGSF11*	2.7674	0.697	0.005	0	0
14	Glial cells	*DMD*	2.7532	0.864	0.181	2.084e-59	4.813e-55
14	Glial cells	*SIPA1L2*	2.7503	0.788	0.133	4.946e-67	1.142e-62
14	Glial cells	*NDRG2*	2.6984	0.818	0.200	1.452e-51	3.353e-47
14	Glial cells	*IL1RAPL2*	2.6895	0.545	0.023	8.336e-155	1.925e-150
14	Glial cells	*APOD*	2.6365	0.742	0.076	2.630e-97	6.073e-93
14	Glial cells	*CD9*	2.5821	0.742	0.076	8.906e-97	2.057e-92
14	Glial cells	*NPDC1*	2.4447	0.773	0.124	6.648e-69	1.535e-64
14	Glial cells	*SPATA13*	2.4417	0.621	0.053	6.132e-96	1.416e-91
14	Glial cells	*TGFBR2*	2.4248	0.788	0.234	1.322e-39	3.053e-35
14	Glial cells	*SOX10*	2.4027	0.652	0.000	0	0
14	Glial cells	*ALDH1A1*	2.3896	0.606	0.257	7.768e-18	1.794e-13
14	Glial cells	*ZNF536*	2.3439	0.636	0.002	0	0
14	Glial cells	*LAMB1*	2.3352	0.682	0.094	3.511e-67	8.108e-63
14	Glial cells	*ZEB2*	2.3172	0.909	0.466	7.244e-30	1.673e-25
14	Glial cells	*MARCKS*	2.2886	0.970	0.760	2.755e-32	6.362e-28
14	Glial cells	*DBI*	2.2686	0.818	0.296	4.817e-36	1.112e-31
14	Glial cells	*RDH10*	2.2624	0.439	0.063	2.358e-38	5.446e-34
14	Glial cells	*BCHE*	2.2543	0.636	0.123	2.434e-43	5.621e-39
14	Glial cells	*SORCS1*	2.2541	0.530	0.024	3.125e-143	7.215e-139
14	Glial cells	*CCN1*	2.2390	0.591	0.191	4.787e-21	1.105e-16
14	Glial cells	*ALCAM*	2.2374	0.682	0.200	3.929e-31	9.072e-27
14	Glial cells	*CHD7*	2.2358	0.530	0.035	3.851e-103	8.892e-99
14	Glial cells	*ZSWIM6*	2.2160	0.848	0.372	1.523e-29	3.517e-25
14	Glial cells	*SNCA*	2.1970	0.606	0.043	1.237e-108	2.857e-104
14	Glial cells	*TTYH1*	2.1915	0.530	0.000	0	0
14	Glial cells	*DST*	2.1882	0.970	0.577	2.816e-36	6.503e-32
14	Glial cells	*FST*	2.1565	0.530	0.029	3.891e-120	8.985e-116
14	Glial cells	*IL1RAPL1*	2.1403	0.545	0.155	8.046e-23	1.858e-18
14	Glial cells	*ANKRD28*	2.1105	0.682	0.191	9.312e-33	2.150e-28
14	Glial cells	*FZD1*	2.1084	0.621	0.098	2.589e-51	5.979e-47
14	Glial cells	*PTPRZ1*	2.0951	0.409	0.014	5.234e-139	1.209e-134
14	Glial cells	*ITGA1*	2.0880	0.742	0.165	9.092e-45	2.100e-40
14	Glial cells	*SLITRK6*	2.0839	0.439	0.020	1.412e-114	3.261e-110

Original cluster	Annotated cell type	Gene	avg_log2FC	pct.1	pct.2	Raw P value	Bonferroni-adjusted P value
14	Glial cells	*CADM1*	2.0663	0.667	0.208	1.196e-27	2.763e-23
14	Glial cells	*NALF1*	2.0606	0.470	0.086	4.426e-31	1.022e-26
14	Glial cells	*NR6A1*	2.0588	0.470	0.052	1.506e-53	3.478e-49
14	Glial cells	*PTPN13*	2.0581	0.712	0.309	4.931e-22	1.139e-17
14	Glial cells	*GAS2L3*	2.0579	0.576	0.047	1.362e-90	3.146e-86
14	Glial cells	*ITGA6*	2.0569	0.576	0.051	1.570e-83	3.624e-79
14	Glial cells	*NCAM1*	2.0502	0.667	0.193	1.345e-29	3.106e-25
14	Glial cells	*ADGRL3*	2.0151	0.803	0.332	7.228e-27	1.669e-22
14	Glial cells	*PCDH17*	1.9882	0.424	0.028	7.124e-79	1.645e-74
14	Glial cells	*SPECC1*	1.9864	0.515	0.067	1.364e-50	3.149e-46
14	Glial cells	*COL4A1*	1.9790	0.652	0.204	2.210e-25	5.102e-21
14	Glial cells	*TSPAN15*	1.9769	0.636	0.210	5.052e-24	1.167e-19
14	Glial cells	*CRIP1*	1.9628	0.758	0.208	1.038e-33	2.398e-29
14	Glial cells	*FXYD1*	1.9591	0.939	0.751	2.366e-29	5.465e-25
14	Glial cells	*PDGFA*	1.9551	0.576	0.064	6.252e-66	1.444e-61
14	Glial cells	*PDE8A*	1.9531	0.394	0.041	5.583e-48	1.289e-43
14	Glial cells	*TSPAN6*	1.9423	0.727	0.335	4.925e-23	1.137e-18
14	Glial cells	*ADGRG6*	1.9308	0.485	0.028	3.420e-103	7.898e-99
14	Glial cells	*ANTXR2*	1.8984	0.848	0.469	8.377e-23	1.934e-18
14	Glial cells	*CNN3*	1.8932	0.955	0.689	1.810e-29	4.181e-25
14	Glial cells	*SCN9A*	1.8916	0.485	0.051	9.927e-59	2.292e-54
14	Glial cells	*NTNG1*	1.8839	0.561	0.209	1.260e-16	2.910e-12
14	Glial cells	*AATK*	1.8831	0.409	0.002	0	0
14	Glial cells	*DTNA*	1.8808	0.394	0.021	8.997e-90	2.077e-85
14	Glial cells	*SOX5*	1.8778	0.788	0.392	1.624e-19	3.750e-15
14	Glial cells	*CADM3*	1.8570	0.500	0.038	2.362e-83	5.453e-79
14	Glial cells	*MEGF9*	1.8534	0.545	0.052	6.189e-72	1.429e-67
14	Glial cells	*CTNNAL1*	1.8337	0.576	0.110	2.913e-39	6.727e-35
14	Glial cells	*COL18A1*	1.8212	0.576	0.070	1.247e-61	2.879e-57
14	Glial cells	*CMTM5*	1.8211	0.530	0.005	0	0
14	Glial cells	*LGI4*	1.8188	0.439	0.014	1.250e-154	2.887e-150
14	Glial cells	*ADAM10*	1.8139	0.742	0.295	2.101e-25	4.853e-21
14	Glial cells	*GAB1*	1.8004	0.652	0.254	1.952e-20	4.509e-16
14	Glial cells	*LOC103344972*	1.7944	0.424	0.112	7.134e-18	1.647e-13
14	Glial cells	*GPM6A*	1.7770	0.409	0.117	8.029e-16	1.854e-11
14	Glial cells	*TMEM196*	1.7719	0.288	0.000	0	0
14	Glial cells	*DLG2*	1.7504	0.621	0.344	6.984e-12	1.613e-07
14	Glial cells	*ERBB3*	1.7445	0.409	0.015	2.076e-134	4.794e-130
14	Glial cells	*LRP1B*	1.7393	0.379	0.117	2.189e-12	5.056e-08
14	Glial cells	*NFATC2*	1.7195	0.424	0.150	5.631e-11	1.300e-06

Original cluster	Annotated cell type	Gene	avg_log2FC	pct.1	pct.2	Raw P value	Bonferroni-adjusted P value
14	Glial cells	*RIGI*	1.7117	0.424	0.237	9.461e-07	2.185e-02
14	Glial cells	*GAS7*	1.6945	0.515	0.076	4.809e-44	1.111e-39
14	Glial cells	*ATF3*	1.6920	0.682	0.510	2.308e-07	5.329e-03
14	Glial cells	*SBSPON*	1.6886	0.424	0.017	4.606e-126	1.064e-121
14	Glial cells	*PTPRJ*	1.6808	0.424	0.048	4.798e-47	1.108e-42
14	Glial cells	*CRABP1*	1.6778	0.333	0.018	5.465e-75	1.262e-70
14	Glial cells	*ATL1*	1.6748	0.500	0.094	1.926e-32	4.448e-28
14	Glial cells	*LOC127493990*	1.6709	0.379	0.012	2.927e-140	6.760e-136
14	Glial cells	*ADGRB3*	1.6670	0.212	0.047	9.437e-11	2.179e-06
14	Glial cells	*COL21A1*	1.6325	0.470	0.157	1.025e-15	2.367e-11
14	Glial cells	*MINDY2*	1.6323	0.773	0.363	4.048e-22	9.347e-18
14	Glial cells	*TFAP2A*	1.6281	0.303	0.001	0	0
14	Glial cells	*CD59*	1.6274	0.636	0.291	3.518e-16	8.124e-12
14	Glial cells	*NR4A2*	1.6170	0.530	0.145	5.106e-21	1.179e-16
14	Glial cells	*SLIT2*	1.6151	0.455	0.250	7.418e-07	1.713e-02
14	Glial cells	*PTPRM*	1.6064	0.530	0.264	2.268e-10	5.236e-06
14	Glial cells	*DKK1*	1.5981	0.288	0.075	9.532e-12	2.201e-07
14	Glial cells	*PTEN*	1.5768	0.727	0.450	4.930e-16	1.138e-11
14	Glial cells	*VGLL3*	1.5762	0.394	0.028	3.387e-70	7.822e-66
14	Glial cells	*DPP6*	1.5750	0.242	0.039	5.153e-18	1.190e-13
14	Glial cells	*VWA1*	1.5732	0.455	0.068	1.575e-38	3.636e-34
14	Glial cells	*VMP1*	1.5611	0.470	0.180	2.033e-13	4.694e-09
14	Glial cells	*LOC138845002*	1.5531	0.333	0.010	3.159e-124	7.295e-120
14	Glial cells	*PLCE1*	1.5439	0.530	0.154	1.452e-21	3.354e-17
14	Glial cells	*ARPC1A*	1.5406	0.652	0.412	1.017e-12	2.349e-08
14	Glial cells	*LOC100350425*	1.5352	0.364	0.040	1.977e-40	4.565e-36
14	Glial cells	*LOC100349085*	1.5330	0.606	0.301	1.044e-12	2.412e-08
14	Glial cells	*CLASP2*	1.5322	0.667	0.338	1.411e-16	3.257e-12
14	Glial cells	*LOC138844537*	1.5238	0.318	0.013	1.373e-94	3.171e-90
14	Glial cells	*CTNND1*	1.5180	0.515	0.165	1.474e-19	3.404e-15
14	Glial cells	*LIPA*	1.5146	0.424	0.074	5.279e-30	1.219e-25
14	Glial cells	*CELF2*	1.5145	0.864	0.598	1.221e-17	2.819e-13
14	Glial cells	*GPX4*	1.5068	0.773	0.547	1.027e-14	2.371e-10
14	Glial cells	*PRNP*	1.4877	0.470	0.138	4.455e-18	1.029e-13
14	Glial cells	*COL28A1*	1.4843	0.348	0.002	0	0
14	Glial cells	*SNTB1*	1.4828	0.379	0.073	8.925e-23	2.061e-18
14	Glial cells	*TANC2*	1.4815	0.530	0.186	8.331e-16	1.924e-11
14	Glial cells	*TENM3*	1.4811	0.742	0.402	2.606e-14	6.018e-10
14	Glial cells	*PTAR1*	1.4703	0.394	0.156	6.533e-10	1.509e-05
14	Glial cells	*COL4A2*	1.4646	0.379	0.101	1.850e-15	4.271e-11

Original cluster	Annotated cell type	Gene	avg_log2FC	pct.1	pct.2	Raw P value	Bonferroni-adjusted P value
14	Glial cells	*NID2*	1.4618	0.379	0.082	8.176e-21	1.888e-16
14	Glial cells	*MARCHF3*	1.4501	0.318	0.075	3.375e-15	7.795e-11
14	Glial cells	*RETREG1*	1.4424	0.515	0.095	5.830e-34	1.346e-29
14	Glial cells	*ATXN7L1*	1.4375	0.500	0.190	1.256e-13	2.900e-09
14	Glial cells	*ASAP2*	1.4361	0.394	0.110	4.282e-16	9.887e-12
14	Glial cells	*SEMA3C*	1.4263	0.439	0.075	2.933e-29	6.773e-25
14	Glial cells	*LIMA1*	1.4255	0.303	0.089	4.308e-11	9.947e-07
14	Glial cells	*ARAP2*	1.4159	0.379	0.032	1.456e-53	3.361e-49
14	Glial cells	*L1CAM*	1.4132	0.409	0.001	0	0
14	Glial cells	*ANK2*	1.4042	0.561	0.335	7.312e-08	1.689e-03
14	Glial cells	*PCDH10*	1.3917	0.273	0.018	1.742e-51	4.023e-47
14	Glial cells	*MACROD2*	1.3917	0.652	0.352	8.143e-11	1.880e-06
14	Glial cells	*QKI*	1.3881	0.682	0.418	2.348e-13	5.421e-09
14	Glial cells	*SLC44A1*	1.3875	0.545	0.248	1.084e-12	2.504e-08
14	Glial cells	*OXR1*	1.3868	0.667	0.462	1.065e-09	2.460e-05
14	Glial cells	*ARHGEF26*	1.3791	0.409	0.051	2.823e-40	6.520e-36
14	Glial cells	*RND3*	1.3718	0.591	0.345	8.068e-10	1.863e-05
14	Glial cells	*POU3F1*	1.3713	0.318	0.024	2.243e-51	5.180e-47
14	Glial cells	*COL3A1*	1.3645	0.879	0.682	3.848e-12	8.885e-08
14	Glial cells	*ANKRD50*	1.3591	0.576	0.331	2.989e-09	6.902e-05
14	Glial cells	*ART3*	1.3587	0.303	0.003	1.216e-268	2.808e-264
14	Glial cells	*CREB5*	1.3524	0.379	0.176	3.927e-07	9.069e-03
14	Glial cells	*GULP1*	1.3482	0.712	0.448	2.247e-10	5.189e-06
14	Glial cells	*THSD7A*	1.3441	0.348	0.056	1.507e-25	3.480e-21
14	Glial cells	*SORBS1*	1.3427	0.379	0.072	4.140e-23	9.560e-19
14	Glial cells	*CEP112*	1.3356	0.667	0.396	3.416e-11	7.888e-07
14	Glial cells	*RIC1*	1.3353	0.485	0.228	1.307e-10	3.018e-06
14	Glial cells	*PPFIBP1*	1.3300	0.439	0.172	1.459e-11	3.370e-07
14	Glial cells	*SRGAP2*	1.3203	0.576	0.304	1.270e-09	2.933e-05
14	Glial cells	*MAML3*	1.3196	0.455	0.240	1.359e-07	3.138e-03
14	Glial cells	*SKAP2*	1.3149	0.424	0.043	1.968e-51	4.545e-47
14	Glial cells	*MATN2*	1.3126	0.727	0.384	3.523e-15	8.135e-11
14	Glial cells	*AHR*	1.3061	0.258	0.034	8.444e-24	1.950e-19
14	Glial cells	*KHDRBS3*	1.3049	0.379	0.175	2.268e-07	5.238e-03
14	Glial cells	*PLEKHA5*	1.3022	0.727	0.540	6.820e-11	1.575e-06
14	Glial cells	*RBMS1*	1.3000	0.667	0.544	5.581e-08	1.289e-03
14	Glial cells	*RAPGEF2*	1.2953	0.500	0.282	1.238e-07	2.858e-03
14	Glial cells	*LOC103348942*	1.2940	0.197	0.003	2.493e-115	5.757e-111
14	Glial cells	*NDRG1*	1.2878	0.485	0.203	2.099e-11	4.848e-07
14	Glial cells	*ADAMTSL5*	1.2820	0.273	0.029	6.939e-32	1.602e-27

Original cluster	Annotated cell type	Gene	avg_log2FC	pct.1	pct.2	Raw P value	Bonferroni-adjusted P value
14	Glial cells	*HSPG2*	1.2790	0.409	0.125	1.397e-14	3.225e-10
14	Glial cells	*CDH13*	1.2730	0.258	0.055	1.080e-13	2.494e-09
14	Glial cells	*RANBP17*	1.2624	0.379	0.099	5.739e-16	1.325e-11
14	Glial cells	*LMOD1*	1.2575	0.242	0.048	2.987e-14	6.897e-10
14	Glial cells	*MCAM*	1.2546	0.379	0.069	4.416e-24	1.020e-19
14	Glial cells	*TMEM117*	1.2527	0.348	0.106	3.233e-12	7.466e-08
14	Glial cells	*APP*	1.2526	0.773	0.651	1.971e-09	4.552e-05
14	Glial cells	*TLN2*	1.2486	0.394	0.119	2.145e-14	4.954e-10
14	Glial cells	*KCNJ3*	1.2402	0.258	0.063	1.911e-11	4.414e-07
14	Glial cells	*SH3PXD2A*	1.2388	0.424	0.127	4.760e-16	1.099e-11
14	Glial cells	*NDUFS4*	1.2371	0.667	0.386	2.132e-13	4.924e-09
14	Glial cells	*SESN3*	1.2363	0.636	0.468	4.760e-08	1.099e-03
14	Glial cells	*LOC127482892*	1.2329	0.212	0.001	2.357e-228	5.442e-224
14	Glial cells	*SYNM*	1.2257	0.303	0.048	9.289e-23	2.145e-18
14	Glial cells	*SMTN*	1.2251	0.394	0.112	1.851e-15	4.274e-11
14	Glial cells	*COL5A2*	1.2248	0.576	0.361	7.527e-09	1.738e-04
14	Glial cells	*CYP2J2*	1.2168	0.455	0.160	4.452e-14	1.028e-09
14	Glial cells	*RASGEF1C*	1.2092	0.242	0.001	0	0
14	Glial cells	*TIMP3*	1.1994	0.818	0.756	2.364e-07	5.460e-03
14	Glial cells	*CNKSR2*	1.1970	0.545	0.213	1.049e-13	2.422e-09
14	Glial cells	*POSTN*	1.1918	0.379	0.157	3.537e-08	8.167e-04
14	Glial cells	*LOC138843476*	1.1870	0.576	0.311	1.424e-09	3.287e-05
14	Glial cells	*LOC103349543*	1.1862	0.258	0.004	1.441e-149	3.326e-145
14	Glial cells	*DHRS3*	1.1856	0.318	0.060	1.449e-19	3.345e-15
14	Glial cells	*ZNF24*	1.1842	0.485	0.229	9.688e-11	2.237e-06
14	Glial cells	*LOC100346046*	1.1840	0.212	0.011	3.746e-50	8.650e-46
14	Glial cells	*KLF6*	1.1823	0.667	0.502	2.834e-07	6.545e-03
14	Glial cells	*PRSS23*	1.1810	0.455	0.186	2.379e-10	5.493e-06
14	Glial cells	*SLC25A10*	1.1803	0.409	0.114	1.346e-16	3.107e-12
14	Glial cells	*USP6NL*	1.1786	0.318	0.071	3.702e-16	8.549e-12
14	Glial cells	*CCNYL1*	1.1745	0.333	0.090	1.360e-13	3.140e-09
14	Glial cells	*MSN*	1.1682	0.530	0.207	4.677e-14	1.080e-09
14	Glial cells	*GFRA2*	1.1678	0.288	0.014	1.132e-71	2.614e-67
14	Glial cells	*CKB*	1.1674	0.379	0.132	3.827e-11	8.837e-07
14	Glial cells	*COL9A3*	1.1446	0.258	0.050	2.516e-15	5.811e-11
14	Glial cells	*SHISA4*	1.1425	0.273	0.016	1.007e-55	2.326e-51
14	Glial cells	*LOC127492930*	1.1348	0.227	0.000	0	0
14	Glial cells	*ENTPD2*	1.1319	0.242	0.004	1.168e-134	2.697e-130
14	Glial cells	*FERMT2*	1.1289	0.515	0.337	1.563e-06	3.610e-02
14	Glial cells	*PPP1R9A*	1.1277	0.545	0.194	1.421e-14	3.282e-10

Original cluster	Annotated cell type	Gene	avg_log2FC	pct.1	pct.2	Raw P value	Bonferroni-adjusted P value
14	Glial cells	*AKAP6*	1.1273	0.242	0.047	5.370e-14	1.240e-09
14	Glial cells	*KCNK5*	1.1226	0.303	0.046	6.915e-24	1.597e-19
14	Glial cells	*DEPTOR*	1.1212	0.333	0.102	4.305e-11	9.941e-07
14	Glial cells	*LOC100328967*	1.1054	0.485	0.278	1.803e-06	4.164e-02
14	Glial cells	*B2M*	1.1037	0.939	0.877	2.768e-15	6.392e-11
14	Glial cells	*GTDC1*	1.0997	0.500	0.285	2.322e-07	5.362e-03
14	Glial cells	*ITGAV*	1.0988	0.485	0.286	1.030e-06	2.378e-02
14	Glial cells	*FLRT1*	1.0957	0.273	0.022	2.388e-41	5.514e-37
14	Glial cells	*FABP7*	1.0956	0.288	0.006	3.792e-140	8.757e-136
14	Glial cells	*OPHN1*	1.0895	0.379	0.187	6.915e-07	1.597e-02
14	Glial cells	*BMPR1B*	1.0877	0.273	0.030	8.252e-30	1.906e-25
14	Glial cells	*VCL*	1.0855	0.530	0.281	2.358e-09	5.444e-05
14	Glial cells	*AMPH*	1.0850	0.152	0.030	5.191e-09	1.199e-04
14	Glial cells	*ADAMTS20*	1.0835	0.273	0.025	7.789e-36	1.799e-31
14	Glial cells	*MEGF10*	1.0833	0.197	0.006	7.834e-73	1.809e-68
14	Glial cells	*NF1*	1.0816	0.667	0.470	5.923e-08	1.368e-03
14	Glial cells	*ARHGAP42*	1.0749	0.561	0.308	1.400e-08	3.233e-04
14	Glial cells	*AFAP1L2*	1.0740	0.364	0.104	1.090e-13	2.517e-09
14	Glial cells	*LOC103345647*	1.0707	0.455	0.215	3.696e-09	8.535e-05
14	Glial cells	*BCL6*	1.0700	0.439	0.161	6.915e-12	1.597e-07
14	Glial cells	*NR3C1*	1.0682	0.621	0.424	8.974e-08	2.072e-03
14	Glial cells	*ARHGEF12*	1.0592	0.500	0.304	1.012e-06	2.337e-02
14	Glial cells	*PCDH20*	1.0536	0.197	0.002	6.504e-150	1.502e-145
14	Glial cells	*PREX2*	1.0505	0.712	0.453	5.522e-09	1.275e-04
14	Glial cells	*FOXN2*	1.0451	0.318	0.123	7.242e-08	1.672e-03
14	Glial cells	*SVIP*	1.0435	0.258	0.042	5.967e-19	1.378e-14
14	Glial cells	*ATP8A1*	1.0420	0.303	0.098	1.827e-09	4.219e-05
14	Glial cells	*ITPR3*	1.0405	0.258	0.031	3.984e-26	9.199e-22
14	Glial cells	*KCTD1*	1.0400	0.242	0.050	2.845e-13	6.569e-09
14	Glial cells	*KCNB2*	1.0386	0.167	0.035	1.017e-08	2.349e-04
14	Glial cells	*COL1A2*	1.0350	0.758	0.569	1.222e-10	2.822e-06
14	Glial cells	*CCL2*	1.0286	0.106	0.008	4.514e-18	1.042e-13
14	Glial cells	*ITPRID2*	1.0272	0.348	0.153	4.041e-07	9.331e-03
14	Glial cells	*FAM171A1*	1.0249	0.333	0.118	5.680e-09	1.312e-04
14	Glial cells	*PDLIM4*	1.0205	0.439	0.241	9.759e-07	2.254e-02
14	Glial cells	*SOX6*	1.0177	0.636	0.428	1.636e-07	3.779e-03
14	Glial cells	*PLEKHB1*	1.0174	0.288	0.081	4.765e-11	1.100e-06
14	Glial cells	*CYTH3*	1.0143	0.333	0.106	1.447e-10	3.342e-06
14	Glial cells	*MAML2*	1.0127	0.652	0.473	1.267e-06	2.926e-02
14	Glial cells	*RHBDF1*	1.0115	0.197	0.060	8.472e-07	1.956e-02

Original cluster	Annotated cell type	Gene	avg_log2FC	pct.1	pct.2	Raw P value	Bonferroni-adjusted P value
14	Glial cells	*ZFHX4*	1.0113	0.379	0.161	5.071e-08	1.171e-03
14	Glial cells	*BACH2*	1.0112	0.409	0.199	5.802e-07	1.340e-02
14	Glial cells	*TAX1BP3*	1.0101	0.273	0.075	1.004e-10	2.318e-06
14	Glial cells	*SLC27A6*	1.0059	0.273	0.087	8.438e-09	1.948e-04
14	Glial cells	*LOC138850734*	1.0039	0.303	0.040	4.088e-28	9.441e-24
14	Glial cells	*KITLG*	1.0039	0.227	0.046	6.143e-13	1.419e-08
14	Glial cells	*CDH6*	1.0023	0.182	0.051	7.583e-07	1.751e-02
14	Glial cells	*PDPN*	0.9996	0.227	0.008	5.904e-79	1.363e-74
14	Glial cells	*CSK*	0.9994	0.379	0.162	2.986e-08	6.896e-04
14	Glial cells	*RIMS1*	0.9932	0.197	0.052	3.368e-08	7.778e-04
14	Glial cells	*SH3PXD2B*	0.9914	0.288	0.119	1.483e-06	3.424e-02
14	Glial cells	*TNFRSF12A*	0.9907	0.242	0.035	4.312e-20	9.957e-16
14	Glial cells	*RAB38*	0.9855	0.182	0.008	1.162e-46	2.684e-42
14	Glial cells	*EFS*	0.9845	0.303	0.095	3.711e-10	8.570e-06
14	Glial cells	*IGFBP7*	0.9829	0.818	0.518	1.895e-12	4.376e-08
14	Glial cells	*PLP2*	0.9671	0.333	0.131	3.601e-08	8.315e-04
14	Glial cells	*MEX3D*	0.9660	0.212	0.035	2.840e-15	6.559e-11
14	Glial cells	*PPM1E*	0.9625	0.152	0.033	5.644e-08	1.303e-03
14	Glial cells	*LOC138850424*	0.9617	0.864	0.801	4.632e-09	1.070e-04
14	Glial cells	*LOC127489189*	0.9615	0.167	0.037	1.245e-08	2.875e-04
14	Glial cells	*SORBS2*	0.9576	0.303	0.090	9.105e-10	2.103e-05
14	Glial cells	*LGR4*	0.9574	0.242	0.069	8.866e-09	2.047e-04
14	Glial cells	*LOC138850522*	0.9573	0.227	0.010	4.820e-59	1.113e-54
14	Glial cells	*MPP7*	0.9559	0.258	0.078	1.046e-08	2.414e-04
14	Glial cells	*SPTBN1*	0.9558	0.818	0.661	1.642e-09	3.792e-05
14	Glial cells	*CAMK2D*	0.9554	0.742	0.621	2.739e-08	6.325e-04
14	Glial cells	*JPH1*	0.9531	0.182	0.011	1.624e-36	3.751e-32
14	Glial cells	*FAS*	0.9506	0.273	0.054	1.064e-15	2.457e-11
14	Glial cells	*TMCC3*	0.9448	0.212	0.036	8.280e-15	1.912e-10
14	Glial cells	*LOC127489220*	0.9353	0.227	0.019	4.321e-33	9.979e-29
14	Glial cells	*TBC1D9*	0.9315	0.273	0.104	5.658e-07	1.306e-02
14	Glial cells	*RARB*	0.9288	0.318	0.121	5.388e-08	1.244e-03
14	Glial cells	*SPTSSA*	0.9225	0.318	0.134	4.909e-07	1.134e-02
14	Glial cells	*SPATA6*	0.9164	0.318	0.075	6.652e-15	1.536e-10
14	Glial cells	*CNMD*	0.9141	0.167	0.016	6.871e-22	1.587e-17
14	Glial cells	*GFRA3*	0.9135	0.167	0.001	1.065e-152	2.459e-148
14	Glial cells	*NOL4*	0.9129	0.182	0.011	1.693e-35	3.909e-31
14	Glial cells	*PLEKHG1*	0.9093	0.303	0.082	1.881e-11	4.343e-07
14	Glial cells	*SLC43A3*	0.9065	0.227	0.036	2.371e-17	5.476e-13
14	Glial cells	*RBMS3*	0.9057	0.773	0.653	8.083e-08	1.867e-03

Original cluster	Annotated cell type	Gene	avg_log2FC	pct.1	pct.2	Raw P value	Bonferroni-adjusted P value
14	Glial cells	*ETS1*	0.9054	0.318	0.122	1.746e-07	4.033e-03
14	Glial cells	*SEMA3B*	0.9016	0.258	0.045	2.723e-17	6.288e-13
14	Glial cells	*LOC103350898*	0.9003	0.288	0.076	1.221e-11	2.819e-07
14	Glial cells	*MPPED2*	0.8998	0.197	0.045	1.793e-09	4.139e-05
14	Glial cells	*LOC100356004*	0.8954	0.576	0.403	6.317e-07	1.459e-02
14	Glial cells	*JUN*	0.8946	0.909	0.855	1.729e-10	3.993e-06
14	Glial cells	*OLFM1*	0.8914	0.303	0.070	3.134e-14	7.238e-10
14	Glial cells	*CRIP2*	0.8913	0.333	0.090	8.616e-13	1.990e-08
14	Glial cells	*HSPA12A*	0.8887	0.242	0.030	3.735e-24	8.624e-20
14	Glial cells	*EVI2A*	0.8882	0.167	0.008	2.519e-40	5.817e-36
14	Glial cells	*ANKRD12*	0.8835	0.697	0.513	1.720e-07	3.971e-03
14	Glial cells	*ENDOD1*	0.8823	0.273	0.090	2.822e-08	6.517e-04
14	Glial cells	*SDC4*	0.8762	0.273	0.069	3.559e-11	8.219e-07
14	Glial cells	*OLFML2B*	0.8751	0.121	0.006	5.135e-32	1.186e-27
14	Glial cells	*LGALS1*	0.8743	0.742	0.533	1.282e-07	2.960e-03
14	Glial cells	*ITGA2*	0.8644	0.288	0.071	1.129e-12	2.607e-08
14	Glial cells	*SRPX2*	0.8640	0.152	0.000	7.421e-254	1.714e-249
14	Glial cells	*AGRN*	0.8632	0.273	0.053	2.869e-16	6.624e-12
14	Glial cells	*OGN*	0.8534	0.758	0.585	8.362e-07	1.931e-02
14	Glial cells	*MT3*	0.8510	0.212	0.024	2.460e-23	5.681e-19
14	Glial cells	*DOCK5*	0.8506	0.182	0.045	3.853e-08	8.897e-04
14	Glial cells	*CNTN4*	0.8491	0.182	0.003	1.448e-120	3.344e-116
14	Glial cells	*SLC27A1*	0.8433	0.258	0.044	1.150e-17	2.656e-13
14	Glial cells	*EDNRB*	0.8429	0.227	0.039	2.222e-15	5.131e-11
14	Glial cells	*DUSP6*	0.8410	0.227	0.027	1.749e-23	4.039e-19
14	Glial cells	*GRIK3*	0.8378	0.197	0.003	7.430e-121	1.716e-116
14	Glial cells	*VIM*	0.8315	0.939	0.934	1.155e-11	2.668e-07
14	Glial cells	*MAP1LC3A*	0.8203	0.576	0.402	1.884e-06	4.350e-02
14	Glial cells	*FAM234A*	0.8193	0.197	0.059	6.466e-07	1.493e-02
14	Glial cells	*FAM181B*	0.8113	0.318	0.102	6.087e-10	1.406e-05
14	Glial cells	*SPARC*	0.8046	0.712	0.516	6.530e-08	1.508e-03
14	Glial cells	*ATP10B*	0.7989	0.106	0.002	2.811e-59	6.490e-55
14	Glial cells	*PPP6R2*	0.7871	0.197	0.059	7.871e-07	1.818e-02
14	Glial cells	*FRMD8*	0.7864	0.227	0.067	4.952e-08	1.144e-03
14	Glial cells	*STK17A*	0.7861	0.288	0.112	9.138e-07	2.110e-02
14	Glial cells	*NTN4*	0.7833	0.227	0.075	7.675e-07	1.772e-02
14	Glial cells	*FAM89A*	0.7830	0.182	0.019	3.658e-21	8.446e-17
14	Glial cells	*LOC138845018*	0.7784	0.212	0.030	1.392e-18	3.214e-14
14	Glial cells	*HHAT*	0.7771	0.106	0.019	1.686e-07	3.894e-03
14	Glial cells	*PRDM16*	0.7762	0.136	0.006	2.309e-36	5.331e-32

Original cluster	Annotated cell type	Gene	avg_log2FC	pct.1	pct.2	Raw P value	Bonferroni-adjusted P value
14	Glial cells	*MEF2C*	0.7754	0.409	0.132	8.566e-11	1.978e-06
14	Glial cells	*CDK15*	0.7644	0.152	0.004	1.011e-69	2.335e-65
14	Glial cells	*SRRM2*	0.7638	0.833	0.737	1.018e-06	2.350e-02
14	Glial cells	*NGFR*	0.7623	0.136	0.001	1.411e-111	3.259e-107
14	Glial cells	*RELN*	0.7620	0.227	0.053	4.059e-10	9.372e-06
14	Glial cells	*CDK2AP2*	0.7612	0.273	0.103	1.097e-06	2.534e-02
14	Glial cells	*SEMA4B*	0.7579	0.212	0.058	1.965e-08	4.537e-04
14	Glial cells	*SLITRK5*	0.7530	0.167	0.003	6.361e-104	1.469e-99
14	Glial cells	*SH3TC2*	0.7476	0.152	0.014	2.972e-21	6.863e-17
14	Glial cells	*LOC108176990*	0.7460	0.227	0.073	7.648e-07	1.766e-02
14	Glial cells	*CORO2B*	0.7436	0.197	0.057	2.040e-07	4.711e-03
14	Glial cells	*RAP2A*	0.7349	0.197	0.060	1.095e-06	2.529e-02
14	Glial cells	*ARHGEF40*	0.7319	0.212	0.071	2.033e-06	4.695e-02
14	Glial cells	*DAGLA*	0.7296	0.152	0.014	4.038e-20	9.325e-16
14	Glial cells	*LOC127493187*	0.7255	0.182	0.001	2.509e-257	5.793e-253
14	Glial cells	*WNT6*	0.7227	0.288	0.089	2.713e-09	6.264e-05
14	Glial cells	*ARPC1B*	0.7227	0.333	0.128	8.643e-08	1.996e-03
14	Glial cells	*LOC100346561*	0.7218	0.212	0.068	7.233e-07	1.670e-02
14	Glial cells	*CPXM2*	0.7185	0.152	0.023	5.806e-12	1.341e-07
14	Glial cells	*NLGN3*	0.7183	0.136	0.006	1.060e-38	2.448e-34
14	Glial cells	*P2RX7*	0.7168	0.136	0.029	9.335e-08	2.156e-03
14	Glial cells	*AFAP1L1*	0.7070	0.182	0.012	7.420e-34	1.713e-29
14	Glial cells	*FEZ1*	0.7035	0.197	0.063	2.125e-06	4.908e-02
14	Glial cells	*TAFA5*	0.7020	0.136	0.001	8.097e-145	1.870e-140
14	Glial cells	*CPEB1*	0.6988	0.136	0.017	1.149e-13	2.652e-09
14	Glial cells	*CADM4*	0.6953	0.152	0.009	4.040e-32	9.329e-28
14	Glial cells	*PAX3*	0.6950	0.136	0.000	1.817e-224	4.197e-220
14	Glial cells	*LOC103345435*	0.6949	0.106	0.000	5.480e-214	1.265e-209
14	Glial cells	*GFRA1*	0.6916	0.152	0.009	4.294e-32	9.916e-28
14	Glial cells	*LOC100354197*	0.6893	0.121	0.009	9.914e-21	2.289e-16
14	Glial cells	*ITGB4*	0.6892	0.152	0.013	7.134e-23	1.647e-18
14	Glial cells	*LOC138847690*	0.6887	0.182	0.041	3.876e-09	8.951e-05
14	Glial cells	*DNM3*	0.6845	0.318	0.124	8.181e-07	1.889e-02
14	Glial cells	*TMSB4X*	0.6824	0.970	0.975	1.365e-11	3.151e-07
14	Glial cells	*FSTL3*	0.6800	0.136	0.017	2.841e-13	6.561e-09
14	Glial cells	*CD99*	0.6771	0.894	0.858	1.249e-06	2.885e-02
14	Glial cells	*ST8SIA1*	0.6766	0.167	0.010	1.421e-32	3.281e-28
14	Glial cells	*ADGRE5*	0.6664	0.167	0.029	1.352e-11	3.121e-07
14	Glial cells	*SCHIP1*	0.6641	0.242	0.086	2.090e-06	4.826e-02
14	Glial cells	*LOC138845008*	0.6636	0.167	0.042	2.885e-07	6.662e-03

Original cluster	Annotated cell type	Gene	avg_log2FC	pct.1	pct.2	Raw P value	Bonferroni-adjusted P value
14	Glial cells	*ADAM23*	0.6607	0.136	0.001	6.061e-117	1.400e-112
14	Glial cells	*KANK4*	0.6568	0.197	0.056	2.813e-07	6.496e-03
14	Glial cells	*P3H2*	0.6563	0.242	0.069	1.158e-08	2.673e-04
14	Glial cells	*CUEDC1*	0.6541	0.182	0.049	2.710e-07	6.258e-03
14	Glial cells	*ITGA4*	0.6503	0.182	0.048	3.050e-07	7.044e-03
14	Glial cells	*MYO10*	0.6429	0.197	0.061	1.530e-06	3.533e-02
14	Glial cells	*CDH2*	0.6372	0.121	0.026	1.500e-06	3.465e-02
14	Glial cells	*FZD8*	0.6326	0.152	0.029	2.511e-09	5.799e-05
14	Glial cells	*IPCEF1*	0.6271	0.167	0.046	1.964e-06	4.535e-02
14	Glial cells	*MGAT3*	0.6230	0.106	0.014	2.618e-10	6.046e-06
14	Glial cells	*MARCHF10*	0.6212	0.152	0.009	9.223e-33	2.130e-28
14	Glial cells	*INSC*	0.6211	0.152	0.003	1.133e-83	2.616e-79
14	Glial cells	*DEPDC7*	0.6189	0.152	0.014	4.336e-21	1.001e-16
14	Glial cells	*BICD1*	0.6166	0.182	0.039	1.676e-09	3.871e-05
14	Glial cells	*LOC138849261*	0.6156	0.152	0.001	1.099e-131	2.537e-127
14	Glial cells	*RUNX1*	0.6136	0.167	0.042	3.928e-07	9.071e-03
14	Glial cells	*HMGA2*	0.6123	0.136	0.027	4.237e-08	9.783e-04
14	Glial cells	*LOC103350037*	0.6112	0.121	0.012	2.969e-15	6.857e-11
14	Glial cells	*TUBB4A*	0.6101	0.106	0.013	7.229e-11	1.669e-06
14	Glial cells	*FBXO2*	0.6089	0.167	0.033	6.399e-10	1.478e-05
14	Glial cells	*NGF*	0.5954	0.197	0.053	8.560e-08	1.977e-03
14	Glial cells	*LRATD2*	0.5775	0.152	0.039	1.656e-06	3.824e-02
14	Glial cells	*RXRG*	0.5772	0.152	0.001	1.253e-131	2.893e-127
14	Glial cells	*NFKBID*	0.5730	0.167	0.027	5.434e-12	1.255e-07
14	Glial cells	*CAPG*	0.5708	0.152	0.035	1.723e-07	3.979e-03
14	Glial cells	*LOC127483689*	0.5624	0.106	0.002	5.684e-55	1.312e-50
14	Glial cells	*LOC103350166*	0.5602	0.106	0.000	2.631e-166	6.075e-162
14	Glial cells	*GPR37L1*	0.5597	0.167	0.000	2.395e-283	5.531e-279
14	Glial cells	*LOC138843693*	0.5567	0.121	0.000	1.507e-162	3.481e-158
14	Glial cells	*FA2H*	0.5562	0.121	0.002	2.783e-84	6.425e-80
14	Glial cells	*HEPACAM*	0.5460	0.121	0.000	3.098e-195	7.155e-191
14	Glial cells	*NIBAN1*	0.5430	0.136	0.017	9.056e-14	2.091e-09
14	Glial cells	*LOC138844036*	0.5273	0.106	0.015	1.112e-09	2.569e-05
14	Glial cells	*PCDH1*	0.5246	0.121	0.012	5.198e-15	1.200e-10
14	Glial cells	*ZBTB47*	0.5166	0.152	0.038	7.609e-07	1.757e-02
14	Glial cells	*DTX4*	0.5100	0.136	0.013	2.469e-17	5.702e-13
14	Glial cells	*LOC103350167*	0.5055	0.106	0.000	1.215e-149	2.805e-145
14	Glial cells	*FAM78B*	0.5044	0.106	0.013	4.727e-11	1.092e-06
14	Glial cells	*LOC100359306*	0.5041	0.121	0.008	2.481e-22	5.729e-18
14	Glial cells	*CLIP2*	0.4947	0.136	0.033	1.389e-06	3.207e-02

Original cluster	Annotated cell type	Gene	avg_log2FC	pct.1	pct.2	Raw P value	Bonferroni-adjusted P value
14	Glial cells	*AGPAT2*	0.4907	0.136	0.025	1.130e-08	2.609e-04
14	Glial cells	*MBP*	0.4769	0.152	0.024	2.145e-11	4.953e-07
14	Glial cells	*SCN4B*	0.4733	0.121	0.001	9.988e-130	2.306e-125
14	Glial cells	*LOC108176883*	0.4676	0.136	0.015	6.510e-15	1.503e-10
14	Glial cells	*PRIMA1*	0.4589	0.121	0.010	1.809e-18	4.177e-14
14	Glial cells	*HES1*	0.4497	0.409	0.181	2.104e-06	4.859e-02
14	Glial cells	*EFNA2*	0.4420	0.106	0.015	3.840e-09	8.867e-05
14	Glial cells	*PAQR8*	0.4247	0.121	0.023	1.113e-07	2.570e-03
14	Glial cells	*LOC138849599*	0.4240	0.121	0.008	3.763e-24	8.691e-20
14	Glial cells	*SMOC1*	0.4223	0.136	0.022	4.481e-10	1.035e-05
14	Glial cells	*COL5A3*	0.4191	0.106	0.005	2.096e-27	4.841e-23
14	Glial cells	*MAL*	0.4037	0.121	0.013	8.503e-14	1.963e-09
14	Glial cells	*MMP17*	0.4022	0.106	0.001	1.186e-106	2.738e-102
14	Glial cells	*ARHGAP15*	0.3457	0.152	0.036	8.668e-07	2.002e-02

Note: Positive markers with Bonferroni-adjusted $P < 0.05$ were retained. Within each annotated cell type, genes occurring in more than one constituent cluster were represented by the record with the highest avg_log2FC. All resulting nonredundant markers were ranked in descending order by avg_log2FC and are reported for each cell type. The 14-cell unclassified cluster was excluded. pct.1, proportion of cells expressing the gene in the focal cluster; pct.2, proportion of cells expressing the gene in all remaining cells.
