# Supplementary material for: Single-Cell RNA Sequencing of Uterine Tissue from a 70-Day-Old Female Rabbit: Cellular Composition and Fibroblast-Associated Networks
Source: Genes (Basel). 2026 Sep 16;17(9):1131. doi: 10.3390/genes17091131 (PMC13606278; doi:10.3390/genes17091131)
Supplement: Supplementary file 1 [file genes-17-01131-s001.zip › Supplementary Figures & Tables S1-S6.pdf]

Supplementary Materials

Single-Cell RNA Sequencing of Uterine Tissue from a 70-Day-Old Female Rabbit: Cellular Composition and Fibroblast-Associated Networks

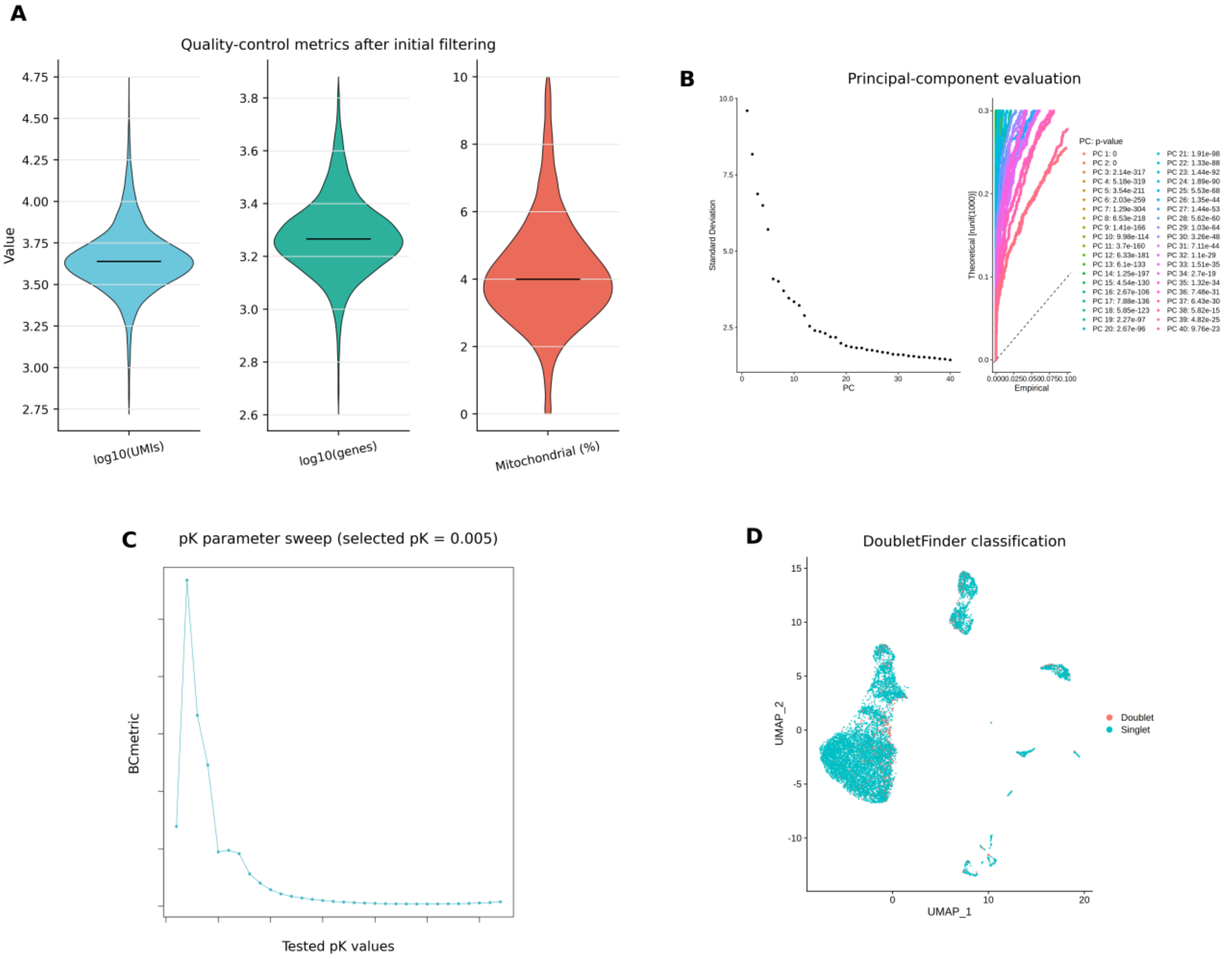

Figure S1. Quality control, principal-component evaluation, and doublet identification in the single rabbit uterine-body dataset. (A) Violin plots of total UMI counts, detected genes, and mitochondrial transcript proportions after initial quality filtering; each point represents one cell. (B) PCA elbow and JackStraw plots used to evaluate principal components, with component number on the x-axis and standard-deviation or significance measures on the y-axis. (C) Mean-variance-normalized bimodality coefficient across tested pK values; pK = 0.005 was selected. (D) UMAP of predicted doublets and retained singlets, with colors indicating classification. A total of 682 predicted doublets were removed, leaving 9,251 singlets from one biological specimen.

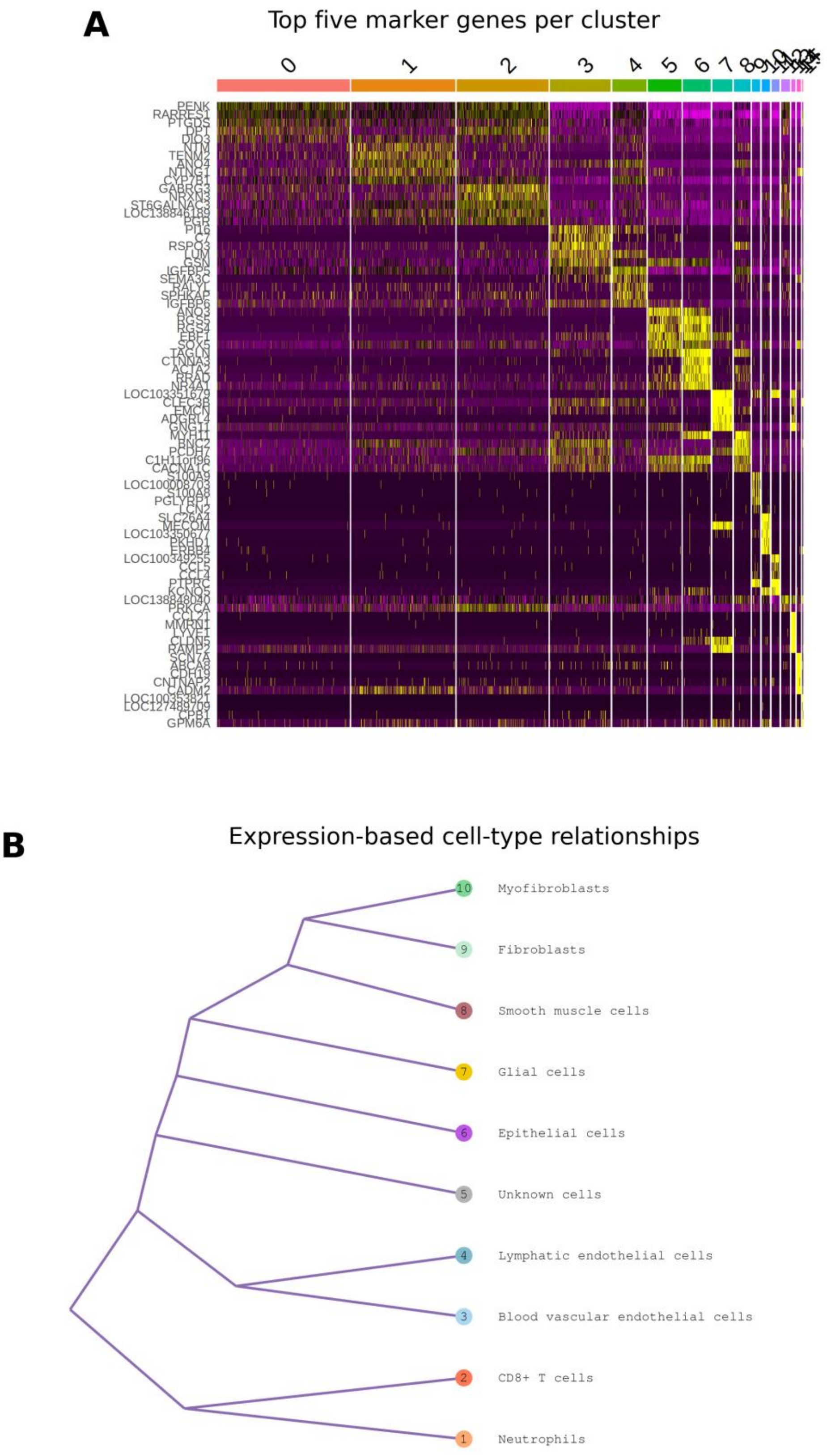

Figure S2. Marker-gene evidence supporting cell-type annotation. (A) Heatmap of the top five positive marker candidates per preliminary Seurat cluster; columns represent cells grouped by cluster, rows represent genes, the upper color bar denotes cluster identity, and heatmap color represents scaled expression. (B) Expression-based hierarchical tree of the nine annotated cell types and the small unclassified population, shown as 'Unknown cells'; branch proximity summarizes transcriptomic similarity and does not represent lineage ancestry.

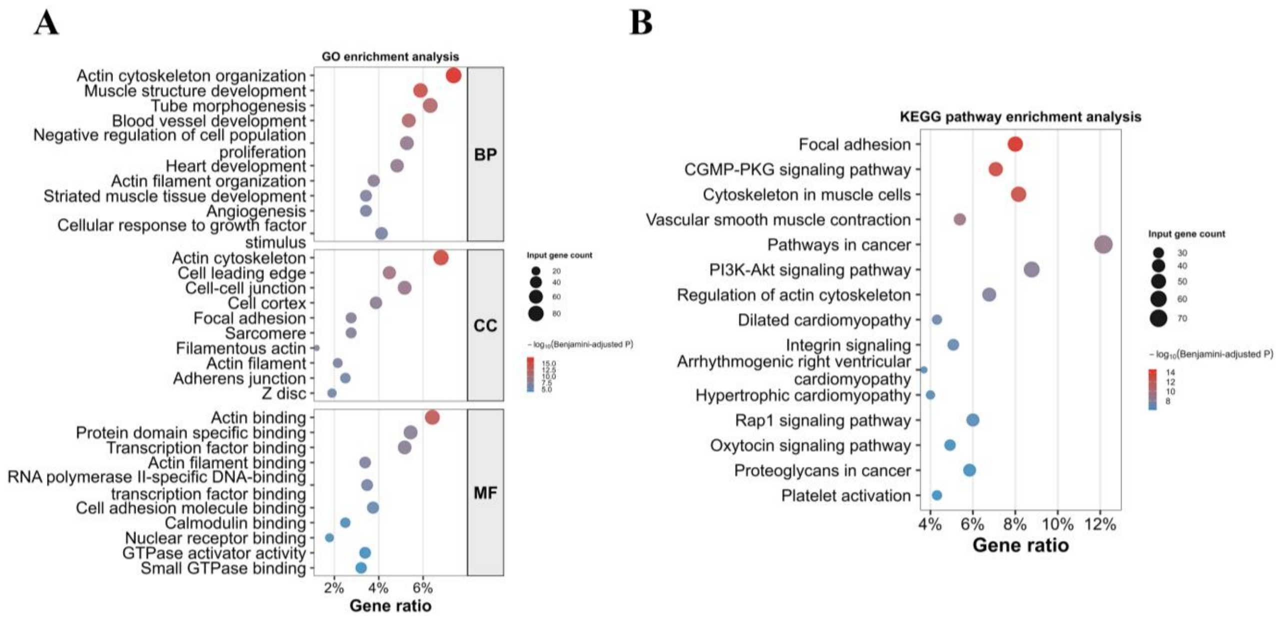

Figure S3. GO and KEGG enrichment analyses of smooth muscle cell marker genes. (A) GO terms separated into Biological Process (BP), Cellular Component (CC), and Molecular Function (MF); (B) KEGG pathways. The x-axis shows gene ratio and the y-axis lists terms or pathways. Bubble size represents input-gene count, and color represents -log10(Benjamini-adjusted P).

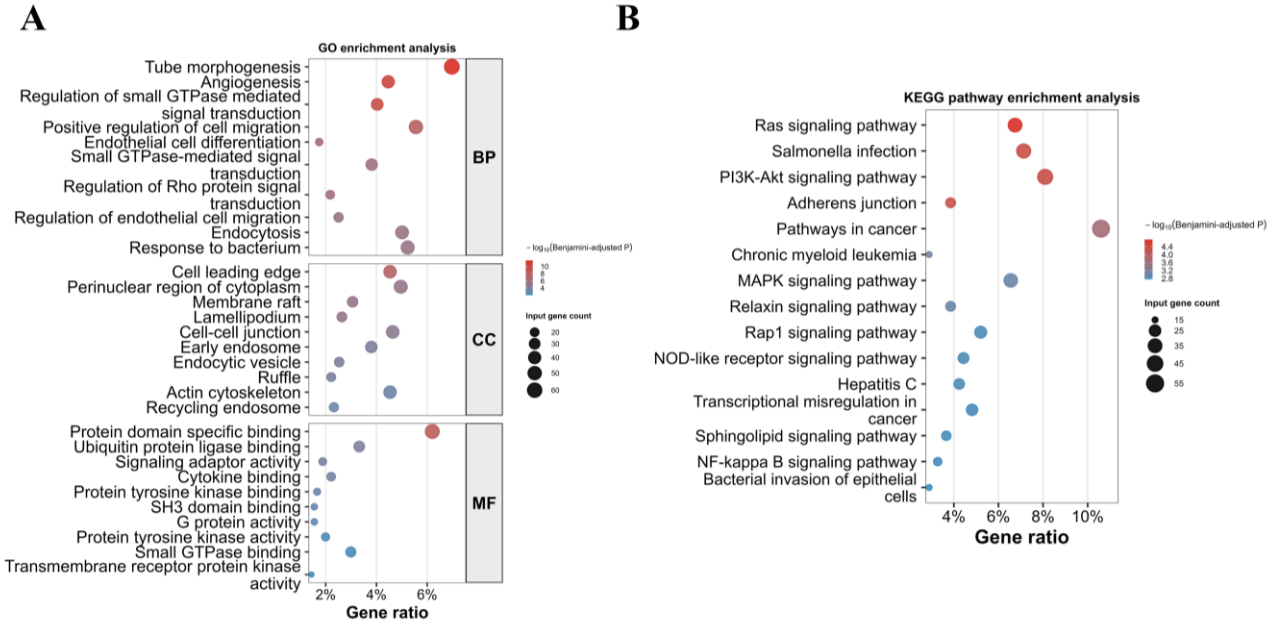

Figure S4. GO and KEGG enrichment analyses of blood vascular endothelial cell marker genes. (A) GO terms separated into Biological Process (BP), Cellular Component (CC), and Molecular Function (MF); (B) KEGG pathways. The x-axis shows gene ratio and the y-axis lists terms or pathways. Bubble size represents input-gene count, and color represents -log10(Benjamini-adjusted P).

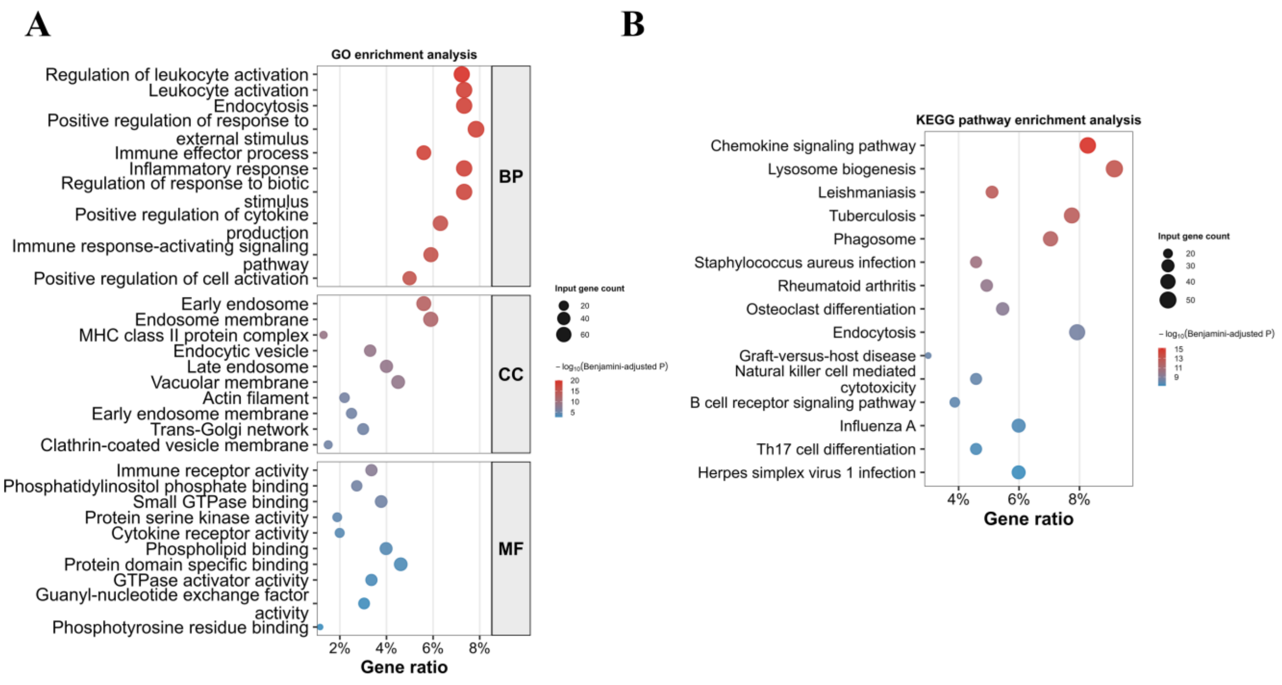

Figure S5. GO and KEGG enrichment analyses of neutrophil marker genes. (A) GO terms separated into Biological Process (BP), Cellular Component (CC), and Molecular Function (MF); (B) KEGG pathways. The x-axis shows gene ratio and the y-axis lists terms or pathways. Bubble size represents input-gene count, and color represents -log10(Benjamini-adjusted P).

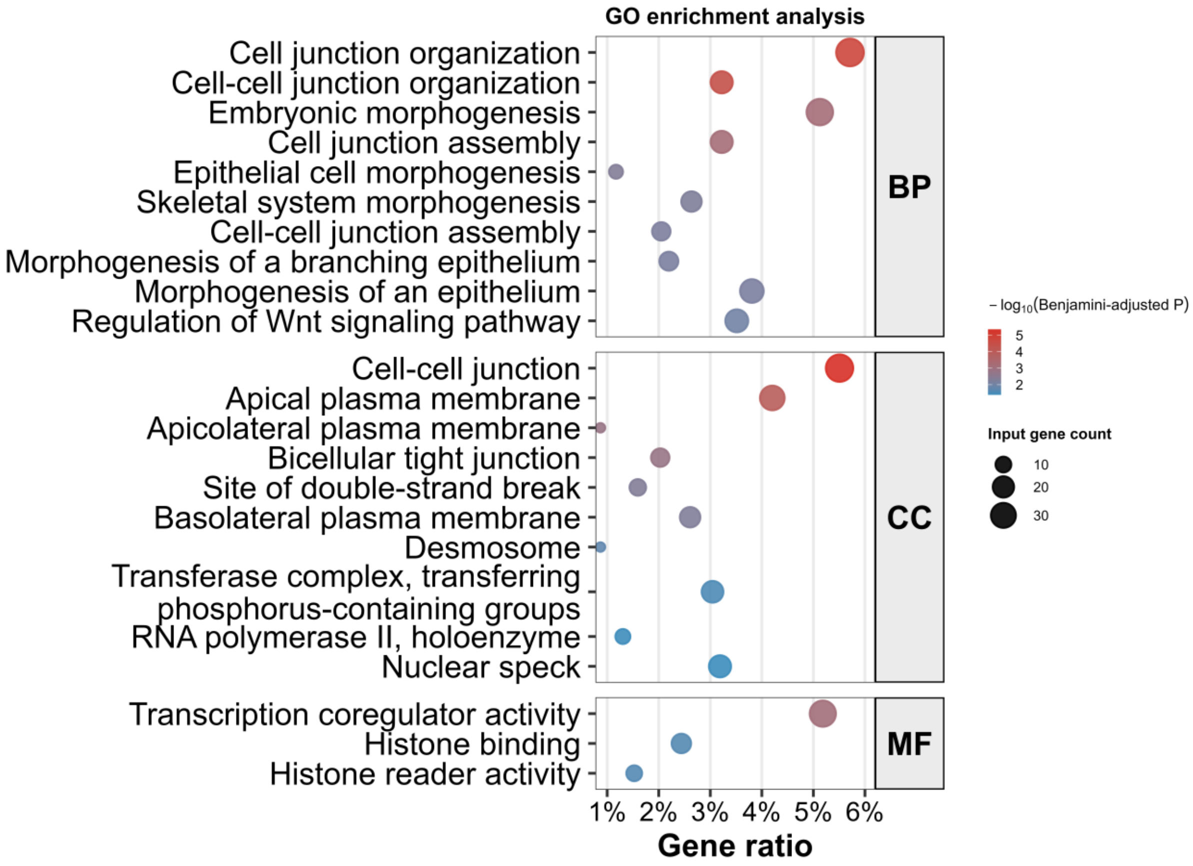

Figure S6. GO enrichment analysis of epithelial cell marker genes. Terms are separated into Biological Process (BP), Cellular Component (CC), and Molecular Function (MF). The x-axis shows gene ratio and the y-axis lists GO terms. Bubble size represents input-gene count, and color represents -log10(Benjamini-adjusted P).

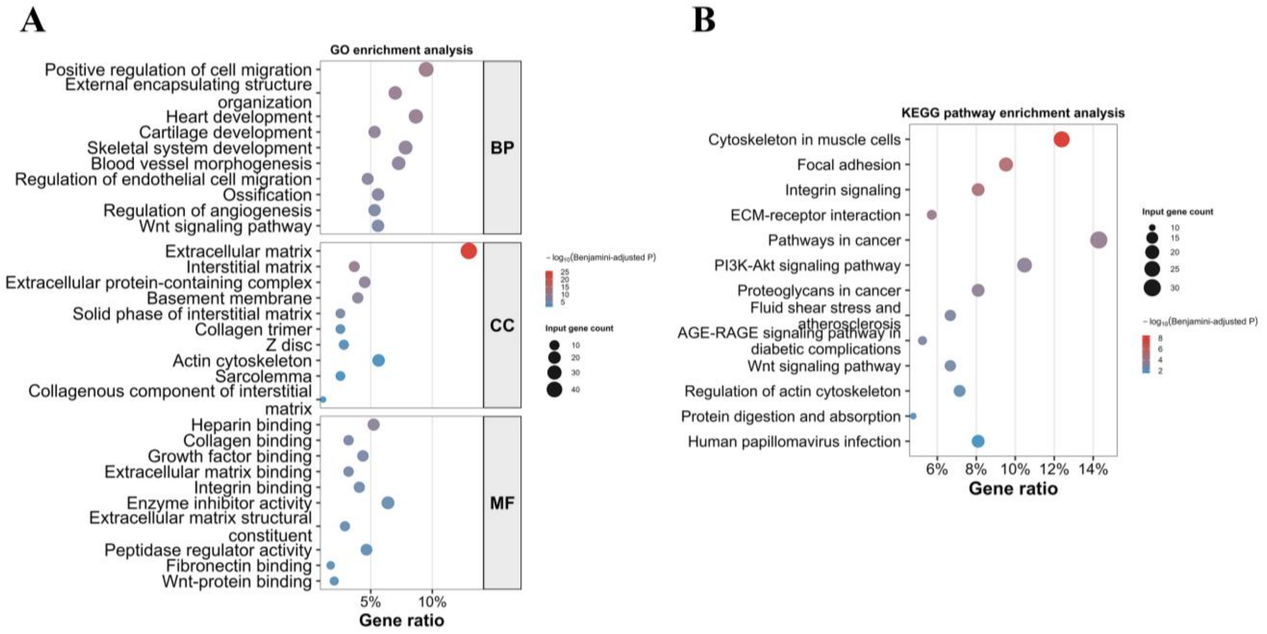

Figure S7. GO and KEGG enrichment analyses of communication-associated fibroblast co-expression modules. Genes from Fibroblasts-M2, Fibroblasts-M4, and Fibroblasts-M5 were combined because only these modules overlapped CellChat ligand‑receptor-related genes retained at an unadjusted empirical permutation P value < 0.05. (A) GO terms separated into Biological Process (BP), Cellular Component (CC), and Molecular Function (MF); (B) KEGG pathways. The x-axis shows gene ratio, the y-axis lists terms or pathways, bubble size represents input-gene count, and color represents -log10(Benjamini-adjusted P).

Supplementary Tables

Table S1. Signaling pathways predicted by CellChat using an unadjusted empirical permutation *p* value < 0.05.

Pathway	Ligand–receptor records	Source–target pairs	Total probability	Maximum probability	Highest-probability source → target
COLLAGEN	432	48	9.6696	0.4481	Glial cells → Epithelial cells
LAMININ	506	48	7.7239	0.4337	Glial cells → Lymphatic endothelial cells
CD99	24	24	2.4651	0.1909	Glial cells → Glial cells
MK	52	29	1.4114	0.2384	Fibroblasts → Epithelial cells
MIF	36	18	1.0832	0.1488	Fibroblasts → Neutrophils
PTPRM	21	21	1.0345	0.1228	Blood vascular endothelial cells → Blood vascular endothelial cells
PTN	18	10	0.9175	0.2215	Fibroblasts → Epithelial cells
NCAM	34	21	0.8501	0.2599	Glial cells → Glial cells
FN1	69	36	0.6986	0.0684	Blood vascular endothelial cells → CD8+ T cells
PECAM1	9	9	0.6870	0.0917	Lymphatic endothelial cells → Lymphatic endothelial cells
VEGF	30	13	0.5935	0.2087	Fibroblasts → Blood vascular endothelial cells
CADM	10	9	0.4809	0.1163	Glial cells → Glial cells
NOTCH	60	27	0.4387	0.0603	Blood vascular endothelial cells → Smooth muscle cells
NRG	10	4	0.3937	0.2007	Epithelial cells → Epithelial cells
APP	7	7	0.3320	0.0755	Lymphatic endothelial cells → Neutrophils
MPZ	12	12	0.3284	0.2137	Glial cells → Glial cells
CDH5	4	4	0.3185	0.0810	Lymphatic endothelial cells → Lymphatic endothelial cells
JAM	44	26	0.3048	0.0461	Blood vascular endothelial cells → Blood vascular endothelial cells
BMP	34	11	0.2636	0.1215	Epithelial cells → Epithelial cells
NEGR	1	1	0.2059	0.2059	Fibroblasts → Fibroblasts
ITGB2	10	10	0.1781	0.0375	Neutrophils → CD8+ T cells
ANGPTL	24	14	0.1642	0.0222	Lymphatic endothelial cells → Blood vascular endothelial cells
PDGF	11	8	0.1516	0.0620	Epithelial cells → Fibroblasts
THY1	4	4	0.1447	0.1010	Lymphatic endothelial cells → Neutrophils
EPHA	18	15	0.1419	0.0193	Epithelial cells → Smooth muscle cells
HSPG	20	20	0.1277	0.0230	Lymphatic endothelial cells → Epithelial cells
NRXN	2	2	0.1041	0.0737	Epithelial cells → Glial cells
ANNEXIN	8	8	0.1022	0.0391	Neutrophils → Neutrophils
CD45	4	4	0.0976	0.0398	CD8+ T cells → Lymphatic endothelial cells
ICAM	12	6	0.0910	0.0497	Blood vascular endothelial cells → Neutrophils
VISTA	3	3	0.0874	0.0617	Neutrophils → Glial cells
WNT	56	12	0.0860	0.0205	Glial cells → Epithelial cells
CXCL	12	9	0.0856	0.0471	Neutrophils → Neutrophils
ESAM	4	4	0.0827	0.0506	Blood vascular endothelial cells → Blood vascular endothelial cells
TGFb	24	16	0.0783	0.0192	CD8+ T cells → Blood vascular endothelial cells
ANGPT	16	10	0.0728	0.0219	Smooth muscle cells → Blood vascular endothelial cells
SEMA3	17	6	0.0712	0.0411	Glial cells → Blood vascular endothelial cells
IGF	8	8	0.0693	0.0474	Lymphatic endothelial cells → Epithelial cells
SELL	4	3	0.0657	0.0461	Neutrophils → Blood vascular endothelial cells
FGF	13	11	0.0652	0.0095	Myofibroblasts → Smooth muscle cells
CCL	6	5	0.0581	0.0346	CD8+ T cells → CD8+ T cells
CALCR	3	3	0.0559	0.0333	Fibroblasts → Blood vascular endothelial cells
GRN	16	16	0.0500	0.0103	Neutrophils → Smooth muscle cells

Pathway	Ligand–receptor records	Source–target pairs	Total probability	Maximum probability	Highest-probability source → target
CDH1	3	3	0.0463	0.0204	Epithelial cells → Epithelial cells
EPHB	12	6	0.0444	0.0131	Blood vascular endothelial cells → Blood vascular endothelial cells
THBS	9	9	0.0406	0.0114	Smooth muscle cells → Epithelial cells
GAS	8	8	0.0405	0.0124	Lymphatic endothelial cells → Smooth muscle cells
OSM	4	3	0.0404	0.0253	Neutrophils → Blood vascular endothelial cells
AGRN	15	15	0.0385	0.0061	Lymphatic endothelial cells → Epithelial cells
GDF	6	6	0.0366	0.0139	Epithelial cells → Lymphatic endothelial cells
NECTIN	9	9	0.0347	0.0113	Lymphatic endothelial cells → CD8+ T cells
CDH	5	4	0.0287	0.0235	Epithelial cells → Epithelial cells
CD226	3	3	0.0261	0.0113	CD8+ T cells → Lymphatic endothelial cells
VISFATIN	7	6	0.0246	0.0093	Blood vascular endothelial cells → Blood vascular endothelial cells
SELPLG	4	4	0.0238	0.0174	Neutrophils → Neutrophils
IL1	1	1	0.0234	0.0234	Neutrophils → Neutrophils
OCLN	1	1	0.0196	0.0196	Epithelial cells → Epithelial cells
LCK	1	1	0.0183	0.0183	CD8+ T cells → CD8+ T cells
ADIPONECTIN	7	4	0.0172	0.0047	Smooth muscle cells → Blood vascular endothelial cells
HGF	2	2	0.0150	0.0087	Fibroblasts → Epithelial cells
COMPLEMENT	1	1	0.0140	0.0140	Neutrophils → Neutrophils
IL2	1	1	0.0125	0.0125	Lymphatic endothelial cells → CD8+ T cells
SEMA6	3	3	0.0122	0.0069	Lymphatic endothelial cells → Blood vascular endothelial cells
BAG	1	1	0.0112	0.0112	Blood vascular endothelial cells → CD8+ T cells
SEMA4	5	3	0.0091	0.0078	CD8+ T cells → Epithelial cells
EDN	2	2	0.0085	0.0067	Blood vascular endothelial cells → Smooth muscle cells
L1CAM	1	1	0.0073	0.0073	Glial cells → Glial cells
CD80	3	2	0.0066	0.0051	Neutrophils → CD8+ T cells
PERIOSTIN	1	1	0.0051	0.0051	Glial cells → Epithelial cells
SELE	9	7	0.0047	0.0018	Blood vascular endothelial cells → CD8+ T cells
PROS	2	2	0.0047	0.0025	Lymphatic endothelial cells → Smooth muscle cells
CD46	2	2	0.0019	0.0011	Epithelial cells → Smooth muscle cells
TWEAK	2	2	0.0016	0.0011	Lymphatic endothelial cells → Epithelial cells
KIT	1	1	0.0009	0.0009	Blood vascular endothelial cells → Epithelial cells
FASLG	1	1	0.0007	0.0007	CD8+ T cells → Glial cells
CLDN	1	1	0.0005	0.0005	Epithelial cells → Epithelial cells

Note: Probabilities are model-derived CellChat communication scores. Counts and probabilities summarize the complete pathway-level output; they are descriptive predictions rather than experimentally validated interaction rates.

Table S2. Summary of fibroblast hdWGCNA modules.

Module	Color	Assigned genes	Median assigned-module kME	Maximum assigned-module kME	Top three hub genes (kME)
Fibroblasts-M1	blue	625	0.119	0.794	RPS12 (0.794); RPL9 (0.791); RPS4X (0.788)
Fibroblasts-M2	yellow	159	0.125	0.390	DLK1 (0.390); FBLN1 (0.372); FCGRT (0.355)
Fibroblasts-M3	turquoise	3229	0.123	0.585	ZBTB20 (0.585); ATRNL1 (0.534); PRKG1 (0.521)
Fibroblasts-M4	brown	185	0.236	0.652	PI16 (0.652); SLIT3 (0.651); C7 (0.609)
Fibroblasts-M5	green	58	0.244	0.668	PENK (0.668); RARRES1 (0.636); SFRP2 (0.559)

Note: The module summary is based on the complete 4,256-gene membership table. The grey set was not included because it represents unassigned genes rather than a biological co-expression module.

Table S3. Top 10 hub genes in each fibroblast hdWGCNA module.

Rank	Fibroblasts-M1	Fibroblasts-M2	Fibroblasts-M3	Fibroblasts-M4	Fibroblasts-M5
1	RPS12 (0.794)	DLK1 (0.390)	ZBTB20 (0.585)	PI16 (0.652)	PENK (0.668)
2	RPL9 (0.791)	FBLN1 (0.372)	ATRNL1 (0.534)	SLIT3 (0.651)	RARRES1 (0.636)
3	RPS4X (0.788)	FCGRT (0.355)	PRKG1 (0.521)	C7 (0.609)	SFRP2 (0.559)
4	RPS5 (0.761)	TIMP2 (0.316)	NEGR1 (0.516)	GSN (0.602)	DPT (0.555)
5	RPL11 (0.760)	SELENOP (0.313)	LOC103350594 (0.504)	LUM (0.583)	TPBG (0.506)
6	RPS3A (0.756)	IGFBP4 (0.309)	LOC127484872 (0.491)	CXCL12 (0.542)	COX6C (0.464)
7	EEF1A1 (0.756)	C1S (0.295)	PTPRD (0.474)	ZFPM2 (0.541)	COL3A1 (0.454)
8	RPL8 (0.753)	HTRA3 (0.293)	TCF12 (0.473)	OGN (0.536)	G0S2 (0.425)
9	RPL23 (0.752)	SERPINF1 (0.292)	SFPQ (0.471)	MFAP5 (0.535)	APCDD1 (0.405)
10	RPS7 (0.749)	RRAS (0.292)	ST6GALNAC3 (0.467)	MGP (0.523)	PTGDS (0.387)

Note: Values in parentheses are kME scores within the corresponding module.

Table S4. Communication-associated candidate genes identified by integrating CellChat interactions retained at an unadjusted empirical permutation *P* value < 0.05 with fibroblast hdWGCNA results. Part A: module and communication summary.

Gene	Module	kME	Interactions	Pathways	Max. probability	Role
CD44	Fibroblasts-M4	0.168	10	4	0.0755	sender
ITGA1	Fibroblasts-M4	0.324	8	2	0.0649	sender
FGFR1	Fibroblasts-M4	0.175	2	2	0.0199	receiver; sender
LAMC1	Fibroblasts-M4	0.236	8	1	0.0170	sender
COL1A1	Fibroblasts-M4	0.246	6	1	0.0712	sender
COL6A2	Fibroblasts-M2	0.259	6	1	0.0685	sender
COL1A2	Fibroblasts-M4	0.228	6	1	0.0684	sender
PTN	Fibroblasts-M5	0.321	4	1	0.1002	sender
BMP2	Fibroblasts-M5	0.359	4	1	0.0118	sender
PDGFRA	Fibroblasts-M5	0.225	3	1	0.0559	receiver
CD99	Fibroblasts-M2	0.259	2	1	0.1604	receiver; sender
FGF2	Fibroblasts-M4	0.208	2	1	0.0071	receiver; sender
VEGFB	Fibroblasts-M2	0.270	1	1	0.0679	sender
NCAM1	Fibroblasts-M4	0.380	1	1	0.0199	receiver
NLGN1	Fibroblasts-M4	0.254	1	1	0.0304	sender
EFNA1	Fibroblasts-M4	0.151	1	1	0.0128	receiver
THY1	Fibroblasts-M4	0.257	1	1	0.0116	sender
GAS6	Fibroblasts-M4	0.260	1	1	0.0104	receiver
IGF1	Fibroblasts-M4	0.142	1	1	0.0054	receiver; sender
EPHA3	Fibroblasts-M4	0.512	1	1	0.0076	sender
ANXA1	Fibroblasts-M4	0.227	1	1	0.0045	sender

Note: kME denotes module eigengene-based connectivity. Candidate genes were obtained by intersecting ligand-receptor-related genes retained at an unadjusted empirical permutation P value < 0.05 with genes assigned to all five fibroblast hdWGCNA modules. Overlaps were found only in Fibroblasts-M2, Fibroblasts-M4, and Fibroblasts-M5.

Table S4 (continued). Part B: signaling pathways and partner cell types for the integrated candidate genes.

Gene	Signaling pathways	Partner cell types
CD44	COLLAGEN; FN1; LAMININ; MIF	CD8+ T cells; Epithelial cells; Neutrophils
ITGA1	COLLAGEN; LAMININ	Blood vascular endothelial cells; Glial cells; Lymphatic endothelial cells; Myofibroblasts; Smooth muscle cells
FGFR1	FGF; NCAM	Blood vascular endothelial cells; Epithelial cells; Fibroblasts; Glial cells; Lymphatic endothelial cells; Myofibroblasts; Smooth muscle cells
LAMC1	LAMININ	Blood vascular endothelial cells; CD8+ T cells; Epithelial cells; Glial cells; Lymphatic endothelial cells; Myofibroblasts; Neutrophils; Smooth muscle cells
COL1A1	COLLAGEN	Blood vascular endothelial cells; CD8+ T cells; Epithelial cells; Glial cells; Lymphatic endothelial cells; Myofibroblasts; Neutrophils; Smooth muscle cells
COL6A2	COLLAGEN	Blood vascular endothelial cells; CD8+ T cells; Epithelial cells; Glial cells; Lymphatic endothelial cells; Myofibroblasts; Neutrophils; Smooth muscle cells
COL1A2	COLLAGEN	Blood vascular endothelial cells; CD8+ T cells; Epithelial cells; Glial cells; Lymphatic endothelial cells; Myofibroblasts; Neutrophils; Smooth muscle cells
PTN	PTN	Blood vascular endothelial cells; CD8+ T cells; Epithelial cells; Fibroblasts; Glial cells; Lymphatic endothelial cells; Smooth muscle cells
BMP2	BMP	Epithelial cells; Fibroblasts; Glial cells; Myofibroblasts; Smooth muscle cells
PDGFRA	PDGF	Blood vascular endothelial cells; Epithelial cells; Glial cells; Smooth muscle cells
CD99	CD99	Fibroblasts; Glial cells; Lymphatic endothelial cells; Neutrophils; Smooth muscle cells
FGF2	FGF	Blood vascular endothelial cells; Epithelial cells; Fibroblasts; Lymphatic endothelial cells; Myofibroblasts; Smooth muscle cells
VEGFB	VEGF	Blood vascular endothelial cells
NCAM1	NCAM	Epithelial cells; Glial cells; Myofibroblasts
NLGN1	NRXN	Glial cells
EFNA1	EPHA	Blood vascular endothelial cells; Epithelial cells; Lymphatic endothelial cells
THY1	THY1	Neutrophils
GAS6	GAS	Blood vascular endothelial cells; Lymphatic endothelial cells
IGF1	IGF	Epithelial cells; Fibroblasts; Lymphatic endothelial cells; Myofibroblasts; Smooth muscle cells
EPHA3	EPHA	Smooth muscle cells
ANXA1	ANNEXIN	Neutrophils

Note: Partner cell types include all cell populations linked to the corresponding candidate gene in the integrated CellChat output.

Table S5. Gene sets used for descriptive functional scoring with Seurat AddModuleScore.

Functional score	Number of genes	Genes
ECM remodeling	18	COL1A1, COL1A2, COL3A1, COL5A1, COL5A2, COL6A1, COL6A2, COL6A3, DCN, LUM, OGN, MFAP5, FN1, MMP2, MMP9, MMP14, TIMP1, TIMP2
Collagen	12	COL1A1, COL1A2, COL3A1, COL4A1, COL4A2, COL5A1, COL5A2, COL6A1, COL6A2, COL6A3, COL14A1, COL15A1
Cell adhesion	16	ITGA1, ITGA2, ITGA5, ITGAV, ITGB1, CD44, THY1, VCAM1, ICAM1, LAMC1, LAMB1, LAMA2, FN1, NCAM1, CDH1, CDH2
Angiogenesis	14	VWF, PECAM1, KDR, FLT1, FLT4, VEGFA, VEGFB, ANGPT1, ANGPT2, TEK, AQP1, PLVAP, EMCN, ESAM
Smooth muscle contraction	11	ACTA2, TAGLN, MYL9, MYH11, LMOD1, TPM1, CNN1, CALD1, DES, RGS5, NOTCH3
Epithelial barrier	13	EPCAM, KRT7, KRT8, KRT18, KRT19, CDH1, CLDN1, CLDN3, CLDN4, OCLN, TJP1, TJP2, MUC1
Inflammatory response	13	CXCL8, CXCL10, CCL2, CCL4, IL1B, IL6, TNF, NFKBIA, S100A8, S100A9, CSF3R, MIF, LST1
Cell cycle	12	MKI67, TOP2A, PCNA, MCM2, MCM3, MCM4, MCM5, MCM6, CDK1, CCNB1, CCNB2, UBE2C
Hormone response	12	ESR1, ESR2, PGR, GREB1, WNT4, WNT5A, IGF1, IGF1R, FOXO1, HAND2, BMP2, PTGS2

Note: The nine functional gene sets were manually assembled from commonly recognized mammalian genes with established roles in the corresponding biological processes, based on published functional knowledge and GO/KEGG annotations.

Table S6. Mean descriptive functional module scores for each uterine cell type. Part A: extracellular matrix, adhesion, angiogenesis, and smooth muscle contraction scores.

Cell type	ECM	Collagen	Adhesion	Angiogenesis	SM contraction	Cells
Fibroblasts	0.182	0.054	-0.059	-0.099	-0.182	7088
Smooth muscle cells	-0.147	-0.077	0.015	-0.058	0.816	1007
Blood vascular endothelial cells	-0.465	-0.445	-0.085	0.673	-0.237	332
Myofibroblasts	-0.080	-0.166	-0.039	-0.093	0.550	273
Neutrophils	-0.329	-0.424	-0.073	-0.076	-0.235	134
Epithelial cells	-0.514	-0.614	0.019	-0.139	-0.298	138
CD8+ T cells	-0.515	-0.571	-0.097	-0.222	-0.312	132
Lymphatic endothelial cells	-0.384	-0.418	0.127	0.335	-0.174	67
Glial cells	0.052	0.204	0.302	-0.153	-0.128	66

Note: Scores are descriptive cell-level means from the single profiled uterus and are not biological-replicate statistics. ECM, extracellular matrix; SM, smooth muscle.

Table S6 (continued). Part B: epithelial barrier, inflammatory response, cell-cycle, and hormone-response scores.

Cell type	Epithelial barrier	Inflammation	Cell cycle	Hormone response	Cells
Fibroblasts	-0.019	0.028	-0.003	0.065	7088
Smooth muscle cells	-0.006	0.005	-0.004	-0.169	1007
Blood vascular endothelial cells	0.082	0.039	0.009	-0.314	332
Myofibroblasts	-0.023	-0.020	0.003	0.023	273
Neutrophils	-0.067	0.908	0.018	-0.185	134
Epithelial cells	0.862	0.024	-0.049	-0.027	138
CD8+ T cells	-0.063	0.054	-0.008	-0.239	132
Lymphatic endothelial cells	0.021	-0.004	-0.032	-0.191	67
Glial cells	-0.011	0.010	-0.017	-0.289	66

Note: Scores are descriptive cell-level means from the single profiled uterus and are not biological-replicate statistics.
